# Independent Prognostic Potential of GNPNAT1 in Lung Adenocarcinoma

**DOI:** 10.1155/2020/8851437

**Published:** 2020-10-29

**Authors:** Xiangyu Zheng, Yongwei Li, Chao Ma, Jinjun Zhang, Yanmin Zhang, Zongqiang Fu, Huan Luo

**Affiliations:** ^1^Department of Laboratory Medicine, The Second Affiliated Hospital of Henan University of Chinese Medicine, Zhengzhou, China; ^2^Charité-Universitätsmedizin Berlin, Corporate Member of Freie Universität Berlin, Humboldt-Universität zu Berlin, and The Berlin Institute of Health, Berlin, Germany

## Abstract

**Background:**

Glucosamine-Phosphate N-Acetyltransferase 1 (GNPNAT1) is a critical enzyme in the biosynthesis of uridine diphosphate-N-acetylglucosamine. It has many important functions, such as protein binding, monosaccharide binding, and embryonic development and growth. However, the role of GNPNAT1 in lung adenocarcinoma (LUAD) remains unclear.

**Methods:**

In this study, we explored the expression pattern and prognostic value of GNPNAT1 in LUAD across TCGA and GEO databases and assessed its independent prognostic value via Cox analysis. LinkedOmics and GEPIA2 were applied to investigate coexpression and functional networks associated with GNPNAT1. The TIMER web tool was deployed to assess the correlation between GNPNAT1 and the main six types of tumor-infiltrating immune cells. Besides, the correlations between GNPNAT1 and the LUAD common genetic mutations, TMB, and immune signatures were examined.

**Results:**

GNPNAT1 was validated upregulated in tumor tissues in TCGA-LUAD and GEO cohorts. Moreover, in both TCGA and GEO cohorts, high GNPNAT1 expression was found to be associated with poor overall survival. Cox analysis showed that high GNPNAT1 expression was an independent risk factor for LUAD. Functional network analysis suggested that GNPNAT1 regulates cell cycle, ribosome, proteasome, RNA transport, and spliceosome signaling through pathways involving multiple cancer-related kinases and E2F family. In addition, GNPNAT1 correlated with infiltrating levels of B cells, CD4+ T cells, and dendritic cells. B cells and dendritic cells could predict the outcome of LUAD, and B cells and CD4+ T cells were significant independent risk factors. The TMB and mutations of KRAS, EGFR, STK11, and TP53 were correlated with GNPNAT1. At last, the correlation analysis showed GNPNAT1 correlated with most of the immune signatures we performed.

**Conclusion:**

Our findings showed that GNPNAT1 was correlated to the prognosis and immune infiltration of LUAD. In particular, the tight relationship between GNPNAT1 and B cell marker genes may be the epicenter of the immune response and one of the key factors affecting the prognosis. Our findings laid the foundation for further research on the immunomodulatory role of GNPNAT1 in LUAD.

## 1. Introduction

Lung cancer is still the most common cancer (accounting for 11.6% of all cancers) and the leading cause of cancer death, with over 1.7 million deaths worldwide in 2018 [1–3]. The survival rate of lung cancer depends mainly on the stage of diagnosis. In general, the current 5-year survival rate is about 18%, but if found early, the prognosis can be improved [4]. Unfortunately, at the time of diagnosis, only about 15% of cases were at the early stage, while the vast majority (57%) were already at the advanced stage [4]. Lung adenocarcinoma (LUAD) is a subclass of non-small-cell lung cancer (NSCLC), which develops along the outer edge of the lungs within glandular cells in the small airways. LUAD accounts for approximately 40% of all lung cancer cases being the most common type of histology [5].

However, due to the combination of adverse factors that span a range of different biological and clinical behaviors and the increased resistance to antilung cancer drugs, existing targeted drugs have shown unsatisfactory efficacy [6]. In NSCLC, little is known about the genomic and host factors that drive the progression of preinvasive lesions. Investigating these factors can enhance our understanding of lung cancer biology, help to develop better screening strategies, and improve patient prognosis [7]. Furthermore, the lack of specific markers for disease stages or tumor types represents a key gap in the current understanding and treatment of LUAD.

Glucosamine-Phosphate N-Acetyltransferase 1 (GNPNAT1), a member of the GCN5-related N-acetyltransferase superfamily, is a key enzyme in the pathway toward biosynthesis of uridine diphosphate-N-acetylglucosamine (UDP-GlcNAc), an important donor substrate for N-linked glycosylation [8]. The gene encoding GNPNAT1 has been characterized in many eukaryotes, such as the murine gene EMeg32 [9]. It is worth noting that EMeg32 is essential for embryonic development, and the level of UDP-GlcNAc that depends on EMeg32 affects sensitivity to apoptosis stimulation and cell cycle progression [10]. One recent study indicated that the expression of GNPNAT1 is associated with the progression of castration-resistant prostate cancer via the phosphatidylinositol3-kinase/protein kinase B signaling pathway [11]. However, whether GNPNAT1 is a biomarker of LUAD and the biological function of GNPNAT1 in LUAD remains to be determined.

In this study, we examined the expression and prognostic value of GNPNAT1 in LUAD patients in the Cancer Genome Atlas (TCGA) and GEO cohorts. Moreover, using multidimensional analysis, we assessed the coexpression and functional network associated with GNPNAT1 in LUAD and learned its role in tumor immunity. The present study may potentially reveal new biological targets and strategies for the diagnosis, treatment, and prognosis assessment of LUAD.

## 2. Materials and Methods

### 2.1. Data Mining from TCGA and GEO Databases

513 LUAD patients' gene expression profiles, along with their clinical data, and survival status were downloaded from the GDC Xena Hub (v2019-08-28, https://gdc.xenahubs.net) with cohort ID: GDC TCGA Lung Adenocarcinoma (TCGA-LUAD). For finding a suitable cohort in GEO for differential gene express validation, the inclusion criteria of the dataset were as follows: (I) expression level of GNPNAT1 in both LUAD and healthy tissues should be available, (II) the size of the dataset should be greater than 100 samples (of which the normal tissue sample should be greater than 50), and (III) the dataset should be released later than the year 2010. GSE19188 (*n* = 45 for LUAD, *n* = 65 for normal) and GSE32863 (*n* = 58 for LUAD, *n* = 58 for normal) were chosen. As for survival validation, the inclusion criteria were (I) expression level of GNPNAT1 of LUAD tissues should be available; (II) the numbers of samples with survival data should be higher than 200; (III) each of the four tumor stages should be available; (IV) the dataset should be released later than 2010. Finally, GSE72094 (*n* = 442) was chosen for survival validation. In our research, TCGA-LUAD, GSE19188, and GSE32863 cohorts were applied to evaluate the difference in expression of GNPNAT1 in normal tissues and tumor tissues. TCGA-LUAD and GSE72094 were used for survival analysis to value how GNPNAT1 affects the prognosis of LUAD.

### 2.2. Differential Expression of GNPNAT1

The distributions of expression of GNPNAT1 in tumor and healthy tissues were examined by unpaired and paired *t*-test in TCGA-LUAD cohort and unpaired *t*-test in GSE19188 and GSE32863 cohorts. R package “beeswarm” was for visualization.

### 2.3. Survival Analysis

Survival analysis was conducted between high and low GNPNAT1 expression groups in cohorts of TCGA-LUAD and GSE72094 through Kaplan–Meier analysis with log-rank test, using “survminer” and “survival” packages in R. In addition, univariate and multivariate Cox analyses were performed on GNPNAT1 and clinical characteristics to assess the potential independent prognostic value of GNPNAT1 in LUAD.

### 2.4. LinkedOmics and GEPIA2 Databases Analysis

LinkedOmics (http://www.linkedomics.org) is a publicly available portal that includes multiomics data from all 32 TCGA cancer types [12]. In the LinkFinder module, the Pearson test was applied to perform statistical analysis on GNPNAT1 coexpression. LinkInterpreter module was used to conducted analyses of Gene Ontology (Biological Process), Kyoto Encyclopedia of Genes and Genomes (KEGG) pathways, kinase-target enrichment, miRNA-target enrichment, and transcription factor-target enrichment through Gene Set Enrichment Analysis (GSEA). The rank criterion was false discovery rate (FDR) < 0.05, and simulation was set as 500. The Gene Expression Profiling Interactive Analysis (GEPIA2) (http://gepia2.cancer-pku.cn/) is a web server for analyzing the RNA sequencing expression data of 9,736 tumors and 8,587 normal samples from the TCGA and the GTEx projects, using a standard processing pipeline [13]. GEPIA2 was applied to plot survival heat maps and survival curves.

### 2.5. The Correlation between GNPNAT1 and Six Types of Infiltrating Immune Cells

The Tumor Immune Estimation Resource (TIMER, https://cistrome.shinyapps.io/timer/) is a comprehensive resource for systematical analysis of immune infiltrates across diverse cancer types [14, 15]. Gene module was applied to explore the correlation between GNPNAT1 expression and abundance of six types of immune cells infiltrates, including B cells, CD4+ T cells, CD8+ T cells, neutrophils, macrophages, and dendritic cells, by tumor purity-corrected partial Spearman's correlation. The Kaplan-Meier analysis was conducted to assess the prognostic capacity of each immune infiltrate. Multivariate Cox analysis was used to evaluate how GNPNAT1 and these six types of immune cells together affect outcomes. *p* value < 0.05 is the threshold of a significant correlation.

### 2.6. Correlation between GNPNAT1 and KRAS Mutation, EGFR Mutation, STK11 Mutation, TP53 Mutation, TMB, and Immune Signatures

The mutations of KRAS, EGFR, STK11, and TP53 were detailed in GSE72094 cohort. The correlation between GNPNAT1 and the above mutations was tested using Spearman's correlation. TMB is defined as the total number of somatic gene coding errors, base substitutions, insertions, or deletions detected per million bases [16]. In our research, the somatic mutation data of the TCGA-LUAD cohort was downloaded from the GDC Data Portal (https://portal.gdc.cancer.gov/). The mutation frequency with the number of variants/the length of exons (38 million) for each sample was calculated via Perl scripts based on the JAVA8 platform (Table S1) [16]. Spearman's rank correlation coefficient was applied to examine the correlation between TMB and GNPNAT1 in TCGA-LUAD cohort. TISIDB (http://cis.hku.hk/TISIDB/) is a central portal for tumor and immune system interactions, which integrates multiple heterogeneous data types [17], and contained various immune gene signatures categorized by type of immune or their function. Gene signatures of chemokine, receptor, major histocompatibility complex (MHC), immunoinhibitor, immunostimulator, and 28 tumor-infiltrating lymphocytes (TILs) [18] were downloaded. The correlations between GNPNAT1 and these gene signatures above were calculated via the “Correlation” module of TIMER with tumor purity-corrected partial Spearman's correlation. The cutoff of *p* value < 0.05 indicates the significance of correlation.

## 3. Results

### 3.1. Clinical Characteristics

**Figure 1:**
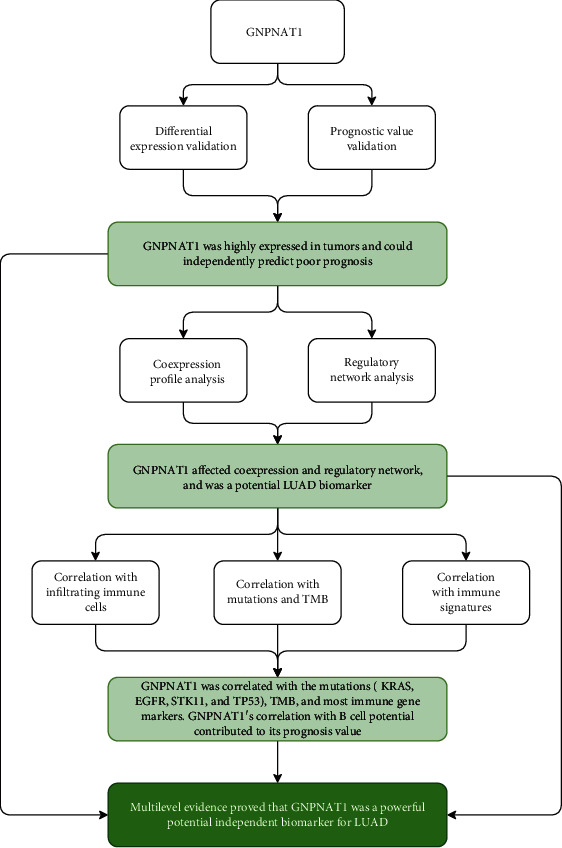
Flow chart of the study. LUAD: lung adenocarcinoma; TMB: tumor mutational burden.

The flowchart of the present research is shown in Figure 1. TCGA-LUAD (*n* = 513 for LUAD, *n* = 59 for normal), GSE19188 (*n* = 45 for LUAD, *n* = 65 for normal), and GSE32863 (*n* = 58 for LUAD, *n* = 58 for normal) were chosen for GNPNAT1 differential expression comparison between tumor and healthy tissues. 513 LUAD cases that came from the TCGA-LUAD cohort and 442 LUADs of the GSE72094 dataset were used as the survival validation purpose (Table 1).

### 3.2. High GNPNAT1 Expression in LUAD

**Figure 2:**
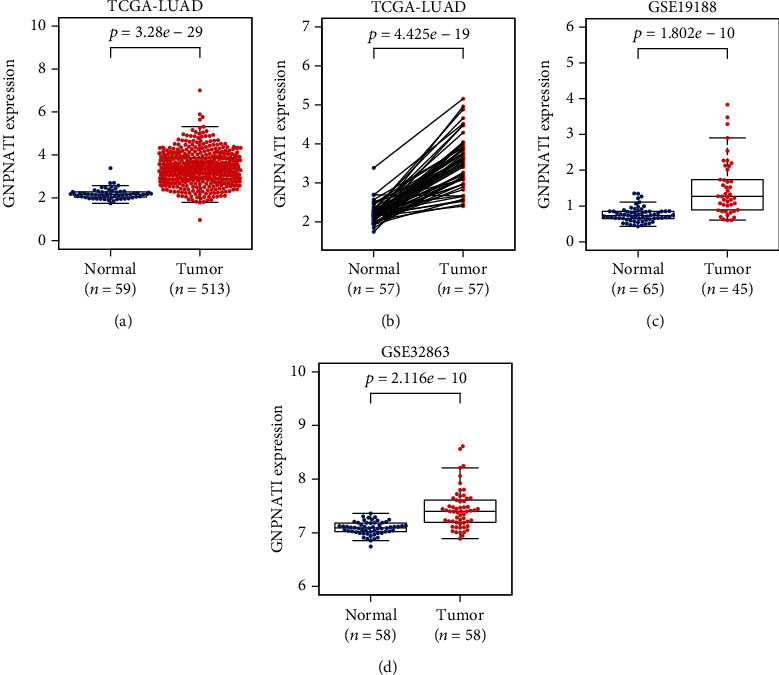
GNPNAT1 was highly expressed in LUAD: (a, b) expression comparisons in TCGA-LUAD cohort; (c) expression comparison in GSE19188 cohort; (d) expression comparison in GSE32863 cohort. TCGA: The Cancer Genome Atlas; LUAD: lung adenocarcinoma.

In TCGA-LUAD cohort, we compared the expression of GNPNAT1 in tumor and adjacent tissues, and the unpaired (*p* value = 3.28*e*−29, Figure 2(a)) and paired (*p* value = 4.425*e*−19, Figure 2(b)) tests both indicated that the expression of GNPNAT1 in the tumor was elevated. Moreover, we examined GNPNAT1 differential expression in independent datasets of GSE19188 (*p* value = 1.802*e*−10, Figure 2(c)) and GSE32863 (*p* value = 2.116*e*−10, Figure 2(d)), finding the consistent results with that in TCGA-LUAD cohort.

### 3.3. High GNPNAT1 Expression Indicated Worse Survival an Acted as an Independent Risk Factor in LUAD

**Figure 3:**
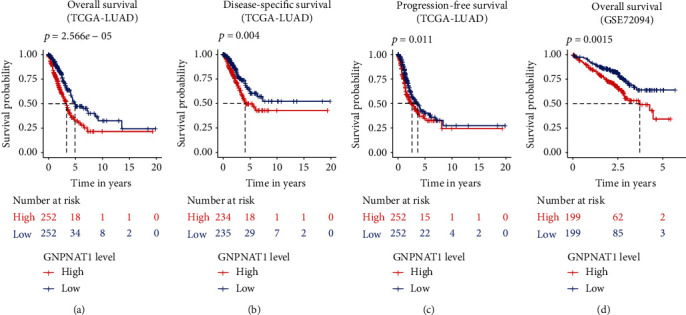
GNPNAT1 was associated with outcome (Kaplan–Meier estimator): (a) overall survival in TCGA-LUAD cohort; (b) disease-specific survival in TCGA-LUAD cohort; (c) progression-free survival in TCGA-LUAD cohort; (d) overall survival of GNPNAT1 in GSE72094 cohort. The numbers below the figures denote the number of patients at risk in each group. TCGA: The Cancer Genome Atlas; LUAD: lung adenocarcinoma.

Then, to understand the correlation between GNPNAT1 expression and patients' outcomes, we used the Kaplan-Meier curves to evaluate and compare the survival differences between patients with high and low expression of GNPNAT1 (Figure 3). In TCGA-LUAD cohort, the high GNPNAT1 expression group had significantly shorter overall survival, and the median overall survival of group of the high GNPNAT1 expression vs. the low expression was 3.33 years vs. 4.93 years (log-rank test, *p* value = 2.566*e*−05, Figure 3(a)). In addition, we also checked how GNPNAT1 performing in disease-specific survival (Figure 3(b)) and progression-free survival (Figure 3(c)) in TCGA-LUAD, finding the high expression of GNPNAT1 predicted a worse prognosis. Consistently, in GSE72094 cohort, the high expression group had significantly unfavorable overall outcomes than the low expression group (*p* value = 0.0015, Figure 3(d)). To assess the risk potential of GNPNAT1 in LUAD, the Cox proportional-hazards model was constructed. In TCGA-LUAD cohort, the overall survival-based Cox analysis showed GNPNAT1 having potential predict value in univariate (HR = 1.68, 95% CI: 1.38-2.05, *p* value = 3.60*E*-07) and multivariate (HR = 2.81, 95% CI: 1.48-5.36, *p* value = 0.00166) test (Table 2). Similarly, the independent prognosis capacity of GNPNAT1 was also confirmed in the disease-specific survival and progression-free survival Cox model (Table S2, Table S3). Additionally, in the GSE72094 cohort, Cox analyses identified the important value of GNPNAT1 in independently predicting the overall survival (Table 3; univariate analysis: HR = 2.02, 95%CI = 1.36‐3, *p* value = 0.000521; multivariate analysis: HR = 1.76, 95%CI = 1.17‐2.65, *p* value = 0.00667).

### 3.4. GNPNAT1 Coexpression and Regulatory Networks in LUAD

**Figure 4:**
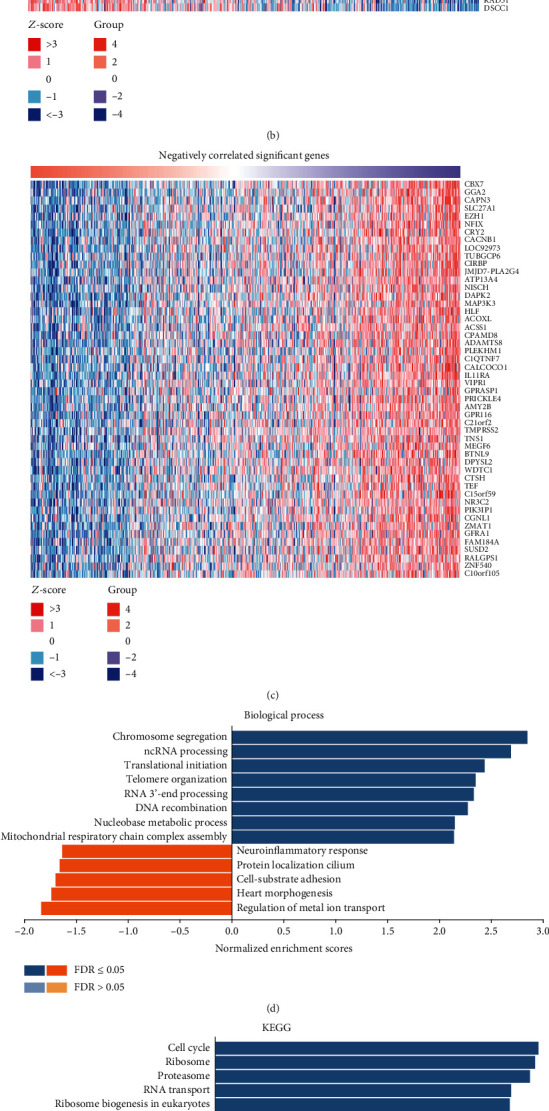
GNPNAT1 coexpression genes in TCGA-LUAD cohort (LinkedOmics). (a) The global GNPNAT1 highly correlated genes identified by Pearson test in the TCGA-LUAD cohort. Red and green dots represent positively and negatively significantly correlated genes with GNPNAT1, respectively. (b, c) Heat maps showing top 50 genes positively and negatively correlated with GNPNAT1 in TCGA-LUAD. (d, e) Significantly enriched GO: Biological Process annotations and KEGG pathways of GNPNAT1 in TCGA-LUAD cohort. (f, g) Survival heat map of the top 50 genes positively and negatively correlated with GNPNAT1 in TCGA-LUAD. The survival heat map shows the hazard ratios in logarithmic scale (log10) for different genes. The red and blue blocks denote higher and lower risks, respectively. The rectangles with frames mean the significant unfavorable and favorable results in prognostic analyses (*p* value < 0.05). FDR: false discovery rate; KEGG: Kyoto Encyclopedia of Genes and Genomes; LUAD: lung adenocarcinoma; TCGA: The Cancer Genome Atlas; GO: Gene Ontology.

In order to better understand the biological meaning of GNPNAT1 in LUAD, the LinkFinder module in the LinkedOmics web portal was deployed to check the coexpression pattern of GNPNAT1 in TCGA-LUAD. As is plotted in Figure 4(a), it showed that 4825 genes (red dots) positively correlated with GNPNAT1, and 7679 genes (green dots) negatively correlated (FDR < 0.05). Figures 4(b) and 4(c) show the heat maps of the top 50 genes positively and negatively associated with GNPNAT1, respectively. Moreover, Table S4 detailed lists the coexpressed genes. Significant Gene Ontology term annotation by GSEA showed that GNPNAT1 coexpressed genes involved mainly in chromosome segregation, ncRNA processing, translational initiation, telomere organization, and RNA 3′-end processing. In contrast, the activities like regulation of metal ion transport, heart morphogenesis, cell-substrate adhesion, and protein localization to cilium were inhibited (Figure 4(d) and Table S5). KEGG analysis showed genes were primarily enriched in the cell cycle, ribosome, proteasome, RNA transport, ribosome biogenesis in eukaryotes, spliceosome pathways, etc. (Figure 4(e) and Table S6). These results indicated the broad impact of GNPNAT1 on global transcriptome. GNPNAT1 displayed a strong positive association with the expression of CDKN3 (positive rank #1, *r* = 0.603, *p* value = 2.08*E*-52), CCNB1 (*r* = 0.599, *p* value = 2.17*E*-51), DLGAP5 (*r* = 0.598, *p* value = 2.78*E*-51), etc. Remarkably, the top 50 positively genes highly owned probability of becoming high-risk markers in LUAD, of which 48/50 genes had unfavorable HR (*p* value < 0.05) (Figure 4(f)). In contrast, still accounted for a high proportion, 32 of the top 50 negatively genes had protective HR (*p* value < 0.05) (Figure 4(g)).

To understand the regulatory factors of GNPNAT1 in LUAD, we further analyzed the enrichment of kinases, miRNAs, and transcription factors of GNPNAT1 coexpressed genes. The top 5 kinases related mainly to CDK1, PLK1, AURKB, CDK2, and ATM (Table 4 and Table S7). In fact, 3 of top 5 kinase genes include CDK1, PLK1, and AURKB were significantly highly expressed in tumor tissues and significantly related to the overall survival of LUAD (Figure S1). Interestingly, the coexpressed genes of GNPNAT1 were not enriched on any miRNA targets significantly (Table S8). Transcription factor enrichment results showed that the coexpressed genes of GNPNAT1 were mainly enriched in the E2F transcription factor family (Table S9), including V$E2F1_Q6, V$E2F_Q6, V$E2F_Q4, V$E2F4DP1_01, and V$E2F1DP1_01. A recent study revealed the biological function of the E2F family gene in the development of cancer, and the possibility of this family gene becoming a potential biomarker of further therapeutic studies in patients with LUAD [19].

### 3.5. Correlation Analysis between GNPNAT1 and Six Kinds of Main Infiltrating Immune Cells

**Figure 5:**
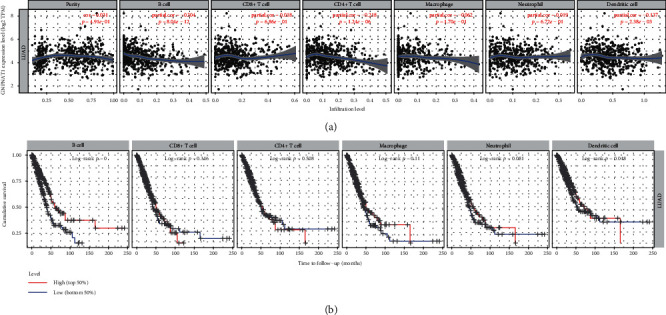
Correlation analysis between the GNPNAT1 expression and six types of infiltrating immune cells in TCGA-LUAD cohort. (a) Correlation of the GNPNAT1 expression with immune infiltration level in the TIMER database. (b) Overall survival curves of each of the six types of immune cells via Kaplan-Meier analysis. LUAD: lung adenocarcinoma; TIMER: The Tumor Immune Estimation Resource.

Then, we investigated whether GNPNAT1 was correlated with six main infiltrating immune cells (B cells, CD4 T cells, CD8+ T cells, neutrophils, macrophages, and dendritic cells) in LUAD using TIMER database. The analysis showed that GNPNAT1 expression levels negatively correlated with B cells (*r* = −0.304, *p* value = 8.04*e*−12), CD4+ T cells (*r* = −0.218, *p* value = 1.24*e*−06), and dendritic cells (*r* = −0.137, *p* value = 2.38*e*−03) (Figure 5(a)). Moreover, we evaluated the prognostic ability of each of the six types of immune cells via the Kaplan-Meier analysis; finding B cells (*p* value = 0 in log−rank test) and dendritic cells (*p* value = 0.048 in log−rank test) can predict the outcome of LUAD (Figure 5(b)). Then, multivariable hazards models were applied to assess the impacts of the GNPNAT1 expression in the presence of six main immune cells. GNPNAT1 showed 1.659 times higher risk on overall survival and can predict tumor outcomes independently of the other six immune cells (HR = 1.659, 95%CI = 1.343‐2.049, *p* value = 0). Interestingly, B cells (HR = 0.009, 95%CI = 0.001‐0.113, *p* value = 0) and CD4+ T cells (HR = 30.567, 95%CI = 1.994‐468.636, *p* value = 0.014) were also significant independent risk factors among all variables (Table 5). Taking together, the significantly infiltrating with B cells seemed like one of the critical factors that GNPNAT1 holds to influence the outcome of LUAD pronounced.

### 3.6. Correlation between GNPNAT1 and the Mutations of KRAS, EGFR, STK11, and TP53, Tumor Mutation Burden (TMB), and Immune Signatures

The mutations of KRAS, EGFR, STK11, and TP53 are correlated with the expression of GNPNAT1 in GSE72094 cohort based on our results. And TMB also had a significant correlation with GNPNAT1 (Table 6).

To expand the understanding of the crosstalk between GNPNAT1 and multiple immune marker genes of 28 TILs, immune inhibitory or stimulatory, cytokine-related, cancer-testis antigen, and MHC, we did correlation analysis between them. The analysis showed that GNPNAT1 was significantly correlated with 66.14% (582/880) immune marker genes (Table S10). Among the significant correlated immune markers, 246/582 (42.27%) were positively; 336/582 (57.73%) were negatively related. On the whole, the top 5 positively correlated marker genes were CDKN3 (*r* = 0.626, *p* value = 4.29*E*-55), CCNB1 (*r* = 0.622, *p* value = 3.87*E*-54), CCNA2 (*r* = 0.616, *p* value = 7.89*E*-53), EXO1 (*r* = 0.594, *p* value = 2.45*E*-48), and KIF11 (*r* = 0.570, *p* value = 8.76*E*-44). Besides, the top 5 negatively correlated markers with GNPNAT1 were LTC4S (*r* = −0.478, *p* value = 1.92*E*-29), DAPK2 (*r* = −0.468, *p* value = 3.68*E*-28), ABTB1 (*r* = −0.460, *p* value = 3.58*E*-27), GNG7 (*r* = −0.448, *p* value = 9.65*E*-26), and HLA-DPB1 (*r* = −0.427, *p* value = 2.56*E*-23). As for immunoinhibitory genes, results showed LGALS9, TGFB1, CD160, CSF1R, and CD96 have negative correlations with GNPNAT1. CD40LG, CD48, IL6R, CD27, CD40, CXCR4, LTA, CXCL12, and CD28 have negative correlations with GNPNAT1 in immunostimulatory genes.

In the previous section, B cell infiltration was found potential to be one of the key reasons that caused GNPNAT1 to become a prognostic factor. Thus, the correlation between GNPNAT1 and B cell marker genes was notable.  Table 7, which was extracted from Table S10, shows the purity-corrected partial Spearman's correlation between GNPNAT1 and B cell markers. In B cells, GNPNAT1 is highly correlated with CDKN3 (#1, *r* = 0.626, *p* value = 4.29*E*-55), CCNA2 (#2, *r* = 0.616, *p* value = 7.89*E*-53), and GNG7 (#3, *r* = −0.448, *p* value = 9.65*E*-26). In total, 38/57 of the B cell marker genes associated significantly to GNPNAT1, of which the number of positive correlations was 8/38 (21.05%) and the negative was 30/38 (78.95%). We plotted the survival heat maps of the significant B cell markers correlated significantly with the GNPNAT1 expression on Figure S2. Interestingly, all the positively markers showed a high probability of becoming high-risk factors in LUAD, of which 3/8 markers had elevated HR (*p* value < 0.05) (Figure S2A). In comparison, there were 22/30 genes with low HR (*p* value < 0.05) in the negatively markers (Figure S2B).

## 4. Discussion

The present study found that GNPNAT1 was highly expressed in LUAD tumor tissue and significantly predicts a poor prognosis. Univariate and multivariate Cox analyses indicated the GNPNAT1 might be a potential independent biomarker for LUAD prognosis. Then, the profiles of coexpression and regulator networks of GNPNAT1 were analyzed. At last, we conducted a correlation analysis between GNPNAT1 and immune infiltration, common gene mutations, TMB, and immune signatures, finding that GNPNAT1 was related to gene mutations, TMB, most of the immune marker genes. The infiltration of GNPNAT1 in B cells may be one of the contributions of GNPNAT1 prognostic ability. Such work we have done aimed to guide future research in LUAD.

Early studies have shown that GNPNAT1 deficiency may reduce insulin secretion associated with type 2 diabetes [20]. In prostate cancer, both GNPNAT1 and UAP1 are highly expressed at the RNA and protein levels. In addition, high UDP-GlcNAc levels correlate with increased UAP1 levels in prostate cancer cells [21]. A recent study showed that prostate cancer contains higher levels of GNPNAT1 and UAP1 transcripts than benign tissue [11]. In addition, there were few other studies on GNPNAT1. In our story, the investigation of differential expression in LUAD indicated that GNPNAT1 was highly expressed in tumor tissues, which was subsequently validated in two independent GEO datasets. Thus, we carried out overall survival analysis in TCGA-LUAD, revealing that the high GNPNAT1 expression was associated with poor outcomes, which was also examined in GSE72094 cohorts. Besides, the Cox analyses further proved GNPNAT1 was an independent risk factor in LUAD. Therefore, our results indicate that GNPNAT1 upregulation occurs in LUAD, and as a potential diagnostic and prognostic marker, it is worthy of further clinical verification.

We explored the regulators responsible for GNPNAT1 dysregulation and found that GNPNAT1 was related to kinase networks, such as CDK1, PLK1, AURKB, CDK2, and ATM. These kinases mainly regulated mitosis, genome stability, and cell cycle and showed survival prognosis value and differential expression in LUAD. CDK1 is a prototype kinase, a central regulator that drives cells through G2 phase and mitosis [22]. CDK1 orchestrates the transition from the G2 phase into mitosis, and as cancer cells often display enhanced CDK1 activity, it has been proposed as a tumor-specific anticancer target [23]. Data mining from different databases (TCGA and GEO) demonstrated CDK1 upregulation in LUAD. Furthermore, CDK1 upregulation is associated with poor prognosis [24]. However, the molecular mechanism and potential application of CDK1 in lung cancer have not been determined [25]. PLK1 is indispensable for finely regulating cell division and maintenance of genomic stability in mitosis, spindle assembly, and DNA damage response [26]. Studies have shown that PLK1 is highly expressed in most human carcinoma, and its overexpression is associated with unfavorable prognosis [27–29]. In human tumors, the overexpression of AURKB is associated with poor prognosis. AURKB inhibitors are in clinical trials for stages I-II leukemia [30]. AURKB is also involved in resistance to certain antitumor agents, such as paclitaxel in NSCLC [31]. Bertran-Alamillo et al. revealed that AURKB is related to acquire resistance to EGFR TKIs, and AURKB can become a potential biological target for anti-EGFR therapy of NSCLC without carrying resistance mutations [32].

In this study, we found that the E2F family was the main transcription factor constituting GNPNAT1 dysregulation. E2F is a group of genes that encodes a family of transcription factors in advanced eukaryotes. They participate regulating the cell cycle and DNA synthesis in mammalian cells [33]. Our analysis did not find miRNAs that are significantly associated with GNPNAT1, which may be due to the fact that GNPNAT1 is mainly involved in the role of mRNA spliceosomes and is far away from miRNA cellular. Our results indicate that E2F1 is a vital regulator of GNPNAT1, and GNPNAT1 may play a role in regulating the cell cycle and proliferation ability of LUAD through this factor.

KRAS mutation is the most common gain-of-function alteration, accounting for ~30% of lung adenocarcinomas in Western countries and about 10% of Asian lung adenocarcinomas [34]. An EGFR mutation causes rapid cell growth, which helps lung cancer spread. Gene testing can identify it and help tailor lung cancer treatment [35]. STK11, mutated or deleted in a third of non-small-cell lung cancer patients, fosters an immunologically “cold” tumor microenvironment, with minimal penetration of tumors by T cells, rendering anti-PD1/PDL1 drugs ineffective [36]. The tumor suppressor gene TP53 is frequently mutated in human cancers. Abnormality of the TP53 gene is one of the most significant events in lung cancers and plays an important role in the tumorigenesis of lung epithelial cells [37]. TMB is defined as the number of mutations per DNA megabases. It was first assessed as a biomarker for ICI based on the observation of successful immune checkpoint inhibition in solid tumors with high TMB in NSCLC [38]. In our research, the above mutations and TMB were all correlated with GNPNAT1, which suggested that GNPNAT1 has the potential to utilize these correlations to obtain the possibility of biological treatment.

This study found that the GNPNAT1 expression had significant negative correlations with B cells infiltrating. Moreover, the subsequent analysis found that B cells could independently predict the outcome of LUAD. These findings indicated that B cell infiltration may be one of the important contributors to GNPNAT1 with prognostic value. Notable, we detailed analyzed the correlation between GNPNAT1 and B cell signatures finding 38/57 (66.67%) of the B cell marker genes associated significantly to GNPNAT1, including CDKN3, CCNA2, and GNG7. It is well known that CDKN3 is overexpressed in multiple human tumor tissues and cell lines [39, 40]. The highly expression of CDKN3 in human cancer tissue may reflect the increased proportion of mitotic cells in the tumor [41]. The elevated CDKN3 expression is associated with the adverse outcome of LUAD. CCNA2, also known as cyclin A2, belongs to the highly conserved cyclin family and plays a key role in cell cycle control [42]. A recent study demonstrated that CCNA2 is a crucial regulator of NSCLC cell metastasis promoting invasion. It has been speculated that GNG7 may be involved in cell contact-induced growth arrest and thus block uncontrolled cell proliferation in multicellular organisms [43]. Correlate analysis provides an exhaustive characterization of the association between GNPNAT1 and immune signatures in LUAD patients, indicating that GNPNAT1 is a crucial player in immune escape in the tumor microenvironment. In addition, the correlation between GNPNAT1 and B cell markers is particularly vital to the prognosis of LUAD patients. It is worth noting that GNPNAT1 may be a key factor mediating B cell therapy, which is needed to be further studied in further research.

The present research also has some limitations. In this study, the cohorts included come from TCGA and GEO databases, which own undoubted academic recognition by most scholars. However, such sample distribution in these cohorts may not be consistent with the clinical population. Therefore, our research may have a selection bias for database selection. Besides, there is currently no wet experimental data explaining the relationship between GNPNAT1 and its mechanism in LUAD samples. Therefore, between GNPNAT1 and the prognosis of LUAD, more effort is needed to clarify the potential relationship.

## 5. Conclusion

This study provided multiple levels of evidence for the importance of GNPNAT1 in the development of lung cancer and its potential as a biomarker and prognostic predictor of LUAD. Our results indicate that the upregulation of GNPNAT1 in LUAD indicates a poor prognosis, which may be caused by multiple steps that affect RNA splicing and genomic stability and cell cycle. Besides, we found that GNPNAT1 has a significant correlation with most immune signatures. In particular, the relationship between GNPNAT1 and B cell marker genes needs to be noted, which might be a new target for future LUAD research.

## Figures and Tables

**Table 1:** Clinical characteristics of cohorts involved in the study for prognostic validation.

Characteristics	TCGA-LUAD cohort, *n* = 513	GSE72094 cohort, *n* = 442
Age		
< 65	220 (42.88%)	115 (26.02%)
≥65	274 (53.41%)	306 (69.23%)
Unknown	19 (3.7%)	21 (4.75%)
Gender		
Female	276 (53.8%)	240 (54.3%)
Male	237 (46.2%)	202 (45.7%)
T classification		
T1	168 (32.75%)	NA
T2	276 (53.8%)	NA
T3	47 (9.16%)	NA
T4	19 (3.7%)	NA
Unknown	3 (0.58%)	NA
N classification		
N0	330 (64.33%)	NA
N1	95 (18.52%)	NA
N2	74 (14.42%)	NA
N3	2 (0.39%)	NA
Unknown	12 (2.34%)	NA
M classification		
M0	344 (67.06%)	NA
M1	25 (4.87%)	NA
Unknown	144 (28.07%)	NA
Tumor stage		
Stage I	274 (53.41%)	265 (59.95%)
Stage II	121 (23.59%)	69 (15.61%)
Stage III	84 (16.37%)	63 (14.25%)
Stage IV	26 (5.07%)	17 (3.85%)
Unknown	8 (1.56%)	28 (6.33%)
Race		
American Indian or Alaska native	1 (0.19%)	0
Asian	7 (1.36%)	1 (0.23%)
Black or African American	52 (10.14%)	13 (2.94%)
Unknown	66 (12.87%)	25 (5.66%)
White	387 (75.44%)	399 (90.27%)
Vietnamese	0	1 (0.23%)
Thai	0	1 (0.23%)
Other	0	2 (0.45%)
Ethnicity		
Hispanic or Latino	7 (1.36%)	10 (2.26%)
Not Hispanic or Latino	382 (74.46%)	402 (90.95%)
Unknown	124 (24.17%)	30 (6.79%)
Vital status		
Alive	326 (63.55%)	298 (67.42%)
Dead	187 (36.45%)	122 (27.6%)
Unknown	0	22 (4.98%)
Tobacco smoking history		
Ever	425 (82.85%)	335 (75.79%)
Never	74 (14.42%)	33 (7.47%)
Unknown	14 (2.73%)	74 (16.74%)
Number pack years smoked		
< 30	119 (23.2%)	NA
≥30	232 (45.22%)	NA
Unknown	162 (31.58%)	NA
Radiation therapy		
Yes	58 (11.31%)	NA
No	370 (72.12%)	NA
Unknown	85 (16.57%)	NA
Additional radiation therapy		
Yes	62 (12.09%)	NA
No	78 (15.2%)	NA
Unknown	373 (72.71%)	NA
Additional pharmaceutical therapy		
YES	61 (11.89%)	NA
NO	76 (14.81%)	NA
Unknown	376 (73.29%)	NA
KRAS mutation		
Yes	23 (4.48%)	154 (34.84%)
No	39 (7.6%)	288 (65.16%)
Unknown	451 (87.91%)	0
EGFR mutation		
Yes	80 (15.59%)	47 (10.63%)
No	193 (37.62%)	395 (89.37%)
Unknown	240 (46.78%)	0
STK11 mutation		
Yes	NA	68 (15.38%)
No	NA	374 (84.62%)
TP53 mutation		
Yes	NA	111 (25.11%)
No	NA	331 (74.89%)
EML4-ALK translocation		
Yes	34 (6.63%)	NA
No	209 (40.74%)	NA
Unknown	270 (52.63%)	NA
Location in lung parenchyma		
Central lung	62 (12.09%)	NA
Peripheral lung	127 (24.76%)	NA
Unknown	324 (63.16%)	NA
Tumor intermediate dimension		
< 1	300 (58.48%)	NA
≥1	88 (17.15%)	NA
Unknown	125 (24.37%)	NA
Person neoplasm cancer status		
Tumor free	243 (47.37%)	NA
With tumor	139 (27.1%)	NA
Unknown	131 (25.54%)	NA

TCGA: The Cancer Genome Atlas; LUAD: lung adenocarcinoma.

**Table 2:** Univariate analysis and multivariate analysis of the correlation of the GNPNAT1 expression with overall survival among lung adenocarcinoma patients in TCGA-LUAD cohort.

Variable	Univariate Cox analysis				Multivariate Cox analysis^∗^			
	Coef	HR (95% CI)	*z*	*p* value	Coef	HR (95% CI)	*z*	*p* value
Age	0.00865	1.01 (0.993-1.02)	1.12	0.264				
Gender (male vs. female)	0.0584	1.06 (0.792-1.42)	0.393	0.694				
Stage II (vs. stage I)	0.904	2.47 (1.72-3.55)	4.87	1.13*E*-06	1.06	2.88 (1.11-7.48)	2.17	0.03
Stage III (vs. stage I)	1.27	3.57 (2.44-5.22)	6.55	5.72*E*-11	0.5	1.65 (0.442-6.14)	0.745	0.456
Stage IV (vs. stage I)	1.34	3.81 (2.2-6.62)	4.76	1.95*E*-06	-0.167	0.846 (0.24-2.99)	-0.259	0.796
Race (white vs. nonwhite)	0.359	1.43 (0.875-2.34)	1.43	0.154				
Ethnicity (Hispanic and Latino vs. non-Hispanic and Latino)	0.384	1.47 (0.464-4.64)	0.654	0.513				
Tobacco smoking history (ever vs. never)	-0.12	0.887 (0.587-1.34)	-0.571	0.568				
Number pack years smoked	0.00327	1 (0.996-1.01)	0.936	0.349				
Radiation therapy (yes vs. no)	0.762	2.14 (1.44-3.19)	3.75	0.000175	0.289	1.34 (0.368-4.84)	0.439	0.66
Additional radiation therapy (yes vs. no)	-0.0197	0.981 (0.622-1.55)	-0.0847	0.932				
Additional pharmaceutical therapy (yes vs. no)	-0.5	0.607 (0.383-0.962)	-2.13	0.0334	-0.814	0.443 (0.21-0.933)	-2.14	0.0322
KRAS mutation (yes vs. no)	0.492	1.63 (0.672-3.98)	1.08	0.278				
EGFR mutation (yes vs. no)	0.268	1.31 (0.828-2.06)	1.15	0.25				
EML4-ALK translocation (yes vs. no)	0.592	1.81 (1.01-3.24)	1.98	0.0473	0.771	2.16 (0.833-5.61)	1.58	0.113
Location in lung parenchyma (central lung vs. peripheral lung)	0.0908	1.09 (0.684-1.75)	0.378	0.706				
Tumor intermediate dimension	0.411	1.51 (0.895-2.54)	1.54	0.122				
GNPNAT1	0.519	1.68 (1.38-2.05)	5.09	3.60*E*-07	1.03	2.81 (1.48-5.36)	3.15	0.00166

^∗^Concordance = 0.746 (se = 0.042), likelihood ratio test = 24.58 on 7 df, *p* = 9*e* − 04, Wald test = 22.07 on 7 df, *p* = 0.002, score (logrank) test = 24.16 on 7 df, *p* = 0.001.

**Table 3:** Univariate analysis and multivariate analysis of the correlation of the GNPNAT1 expression with overall survival among lung adenocarcinoma patients in GSE72094 cohort.

Variable	Univariate Cox analysis				Multivariate Cox analysis^∗^			
	Coef	HR (95% CI)	*z*	*p* value	Coef	HR (95% CI)	*z*	*p* value
Age	0.00696	1.01 (0.988-1.03)	0.702	0.483				
Gender (male vs. female)	0.44	1.55 (1.07-2.25)	2.33	0.0198	0.485	1.62 (1.11-2.37)	2.51	0.0122
Stage II (vs. stage I)	0.758	2.13 (1.32-3.44)	3.11	0.00185	0.771	2.16 (1.34-3.49)	3.16	0.00158
Stage III (vs. stage I)	1.13	3.09 (1.93-4.97)	4.67	3.00*E*-06	1.17	3.22 (1.99-5.22)	4.75	2.00*E*-06
Stage IV (vs. stage I)	1.21	3.35 (1.59-7.06)	3.18	0.00148	1.31	3.7 (1.75-7.84)	3.42	0.000622
Race (white vs. nonwhite)	-0.0694	0.933 (0.38-2.29)	-0.151	0.88				
Ethnicity (Hispanic and Latino vs. non-Hispanic and Latino)	-0.642	0.526 (0.0733-3.78)	-0.638	0.524				
Tobacco smoking history (ever vs. never)	0.314	1.37 (0.597-3.14)	0.741	0.459				
KRAS mutation (yes vs. no)	0.376	1.46 (1-2.12)	1.97	0.0492	0.148	1.16 (0.786-1.71)	0.744	0.457
EGFR mutation (yes vs. no)	-1.34	0.262 (0.0965-0.71)	-2.63	0.00849	-1.07	0.344 (0.125-0.951)	-2.06	0.0397
STK11 mutation (yes vs. no)	-0.0393	0.961 (0.58-1.59)	-0.153	0.879				
TP53 mutation (yes vs. no)	0.211	1.23 (0.82-1.86)	1.01	0.313				
GNPNAT1	0.701	2.02 (1.36-3)	3.47	0.000521	0.565	1.76 (1.17-2.65)	2.71	0.00667

^∗^Concordance = 0.699 (se = 0.026), likelihood ratio test = 52.73 on 7 df, *p* = 4*e* − 09, Wald test = 50.37 on 7 df, *p* = 1*e* − 08, score (logrank) test = 54.68 on 7 df, *p* = 2*e* − 09.

**Table 4:** The kinases, miRNAs, and transcription factors-target networks of *GNPNAT1* in LUAD.

Enriched category	Gene set	Leading edge number	NES	FDR
Kinase target	Kinase_CDK1	84	2.5367	0
	Kinase_PLK1	30	2.4679	0
	Kinase_AURKB	34	2.2754	0
	Kinase_CDK2	90	2.1616	0
	Kinase_ATM	38	2.0821	0
miRNA target	GGGGCCC,MIR-296	27	-1.5696	0.099967
	CCTGTGA,MIR-513	47	-1.579	0.11175
	AGCGCTT,MIR-518F,MIR-518E,MIR-518A	7	-1.5396	0.12257
	GAGCCTG,MIR-484	40	-1.6427	0.13212
	CCCAGAG,MIR-326	49	-1.5866	0.13432
Transcription factor	V$E2F1_Q6	85	2.1908	0
	V$E2F_Q6	81	2.1879	0
	V$E2F_Q4	81	2.1486	0
	V$E2F4DP1_01	82	2.147	0
	V$E2F1DP1_01	82	2.1346	0

LUAD: lung adenocarcinoma; NES: normalized enrichment score; FDR: false discovery rate.

**Table 5:** Multivariate analysis^∗^ of the correlation of the *GNPNAT1* expression and six types of immune cells among lung adenocarcinoma patients.

Variable	Coef	HR	95% CI	*p* value
B cell	-4.732	0.009	0.001-0.113	**0**
CD8+ T cell	0.325	1.384	0.225-8.52	0.726
CD4+ T cell	3.42	30.567	1.994-468.636	**0.014**
Macrophage	0.406	1.501	0.115-19.662	0.757
Neutrophil	-2.161	0.115	0.002-6.365	0.291
Dendritic	0.023	1.023	0.274-3.825	0.973
*GNPNAT1*	0.506	1.659	1.343-2.049	**0**

coef: regression coefficient; HR: hazard ratio; CI: confidence interval; Bold values indicate *p* value < 0.05; ^∗^*R*square = 0.09 (max possible = 9.78*e* − 01), likelihood ratio test *p* = 1.22*e* − 07, Wald test *p* = 4.39*e* − 07, score (logrank) test *p* = 3.74*e* − 07.

**Table 6:** Correlations between GNPNAT1 and the mutations of KRAS, EGFR, STK11, and TP53 and tumor mutational burden in GSE72094 cohort.

Variable	Pearson test		Spearman test	
	*r*	*p* value	*r*	*p* value
KRAS mutation	0.203011986	1.70*E*-05	0.213909277	5.70*E*-06
EGFR mutation	-0.124674225	0.008691702	-0.136064535	0.004159154
STK11 mutation	0.173553017	0.000246114	0.185866334	8.46*E*-05
TP53 mutation	0.118804885	0.012436346	0.12550517	0.008252137
Tumor mutational burden	0.171396115	0.000111969	0.284225295	8.41*E*-11

**Table 7:** Correlation analysis between *GNPNAT1* and B cell markers in TCGA-LUAD cohort.

Variables	None adjusted		Tumor purity adjusted	
	Cor	*p* value	Cor	*p* value
Activated B cell				
GNG7	-0.444	2.46*E*-26	-0.448	**9.65** **E** **-26**
HLA-DOB	-0.306	1.35*E*-12	-0.338	**1.23** **E** **-14**
CLEC9A	-0.315	2.65*E*-13	-0.320	**3.64** **E** **-13**
BLK	-0.249	1.06*E*-08	-0.274	**6.34** **E** **-10**
CLECL1	-0.241	3.15*E*-08	-0.254	**1.13** **E** **-08**
SPIB	-0.218	6.06*E*-07	-0.236	**1.22** **E** **-07**
MS4A1	-0.200	4.59*E*-06	-0.217	**1.22** **E** **-06**
AKNA	-0.210	1.53*E*-06	-0.210	**2.45** **E** **-06**
ARHGAP25	-0.185	2.42*E*-05	-0.199	**8.67** **E** **-06**
MICAL3	0.169	1.16*E*-04	0.181	**5.40** **E** **-05**
CD79B	-0.165	1.76*E*-04	-0.179	**6.67** **E** **-05**
CLEC17A	-0.161	2.44*E*-04	-0.170	**1.54** **E** **-04**
CD19	-0.145	9.52*E*-04	-0.163	**2.72** **E** **-04**
CD27	-0.149	6.75*E*-04	-0.159	**4.00** **E** **-04**
FCRL2	-0.143	1.18*E*-03	-0.148	**1.01** **E** **-03**
CR2	-0.146	9.28*E*-04	-0.146	**1.19** **E** **-03**
TNFRSF17	-0.085	5.52*E*-02	-0.080	7.68*E*-02
TCL1A	-0.088	4.64*E*-02	-0.076	9.07*E*-02
CD180	-0.077	8.01*E*-02	-0.061	1.75*E*-01
CCL21	0.035	4.29*E*-01	0.060	1.80*E*-01
BACH2	0.039	3.77*E*-01	0.060	1.87*E*-01
PNOC	-0.054	2.22*E*-01	-0.053	2.44*E*-01
ADAM28	-0.059	1.80*E*-01	-0.052	2.45*E*-01
CD38	0.027	5.36*E*-01	0.039	3.92*E*-01
Immature B cell				
CD22	-0.311	5.28*E*-13	-0.338	**1.19** **E** **-14**
TXNIP	-0.297	5.54*E*-12	-0.288	**7.59** **E** **-11**
FCRL1	-0.254	5.30*E*-09	-0.273	**7.28** **E** **-10**
HLA-DQA1	-0.255	4.31*E*-09	-0.269	**1.28** **E** **-09**
FAM129C	-0.242	2.55*E*-08	-0.260	**4.97** **E** **-09**
STAP1	-0.234	7.78*E*-08	-0.254	**1.02** **E** **-08**
HVCN1	-0.189	1.54*E*-05	-0.194	**1.49** **E** **-05**
FCRL3	-0.174	6.95*E*-05	-0.179	**6.62** **E** **-05**
TAGAP	-0.166	1.58*E*-04	-0.170	**1.45** **E** **-04**
NCF1B	-0.126	4.07*E*-03	-0.143	**1.49** **E** **-03**
FCRLA	-0.117	7.73*E*-03	-0.131	**3.47** **E** **-03**
NCF1	-0.126	4.09*E*-03	-0.131	**3.51** **E** **-03**
KIAA0226	0.094	3.29*E*-02	0.101	**2.42** **E** **-02**
ZCCHC2	0.102	2.09*E*-02	0.101	**2.55** **E** **-02**
HDAC9	-0.062	1.59*E*-01	-0.052	2.52*E*-01
CYBB	-0.061	1.67*E*-01	-0.039	3.90*E*-01
P2RY10	-0.042	3.39*E*-01	-0.028	5.31*E*-01
FCRL5	0.014	7.48*E*-01	0.020	6.51*E*-01
SP100	-0.009	8.44*E*-01	0.012	7.85*E*-01
Memory B cell				
CDKN3	0.626	1.70*E*-57	0.626	**4.29** **E** **-55**
CCNA2	0.614	8.63*E*-55	0.616	**7.89** **E** **-53**
FCER1A	-0.422	1.09*E*-23	-0.418	**2.59** **E** **-22**
MYC	0.287	3.45*E*-11	0.291	**4.30** **E** **-11**
ENPP1	0.283	6.52*E*-11	0.289	**6.23** **E** **-11**
RUNX2	0.138	1.71*E*-03	0.147	**1.04** **E** **-03**
STAT5A	-0.152	5.37*E*-04	-0.141	**1.69** **E** **-03**
FCRL4	-0.121	5.96*E*-03	-0.132	**3.25** **E** **-03**
SOX5	-0.061	1.65*E*-01	-0.071	1.15*E*-01
SORL1	-0.033	4.54*E*-01	-0.034	4.47*E*-01
STAT5B	-0.041	3.54*E*-01	-0.033	4.66*E*-01
CLCN5	-0.029	5.06*E*-01	-0.032	4.80*E*-01
AICDA	-0.027	5.36*E*-01	-0.015	7.39*E*-01
TLR9	-0.009	8.37*E*-01	-0.006	8.99*E*-01

Cor: correlation coefficient. Bold values indicate *p* value < 0.05.

## Data Availability

Publicly available datasets were analyzed in this study. These data can be found here: TCGA: https://portal.gdc.cancer.gov/; GEO: https://www.ncbi.nlm.nih.gov/geo/.

## Supplementary Materials

Supplementary 1 Figure S1: expression and survival outcome of top 5 kinase regulators of GNPNAT1 coexpressed genes in TCGA-LUAD cohort. CDK1 (A), PLK1 (B), and AURKB (C) were significantly highly expressed in tumor tissues and had significant associations with overall survival while CDK2 (D) and ATM (E) not. TCGA: The Cancer Genome Atlas; LUAD: lung adenocarcinoma. Figure S2: survival heat maps of B cell markers positively and negatively correlated with GNPNAT1 in TCGA-LUAD cohort. The survival heat maps show the hazard ratios in logarithmic scale (log10) for different genes. The red and blue boxes indicate high and low risks, respectively. Framed rectangles indicate significantly adverse and favorable results in prognostic analysis (*p* value < 0.05). LUAD: Lung adenocarcinoma; TCGA: The Cancer Genome Atlas;

Supplementary 2 Table S1: tumor mutation burden (TMB) scores of each sample in the TCGA-LUAD cohort. Table S2: univariate analysis and multivariate analysis of the correlation of GNPNAT1 expression with disease-specific survival among lung adenocarcinoma patients in TCGA-LUAD cohort. Table S3: univariate analysis and multivariate analysis of the correlation of GNPNAT1 expression with progression-free survival among lung adenocarcinoma patients in TCGA-LUAD cohort. Table S4: GNPNAT1 coexpressed genes. Table S5: Gene Ontology term annotation of GNPNAT1 coexpressed genes. Table S6: KEGG annotation of GNPNAT1 coexpressed genes. Table S7: kinases enrichment of GNPNAT1 coexpressed genes. Table S8: miRNA enrichment of GNPNAT1 coexpressed genes. Table S9: transcription factor enrichment of GNPNAT1 coexpressed genes. Table S10: correlation of GNPNAT1 with various immune signatures in TCGA-LUAD cohort.

## References

[1] Bray F., Ferlay J., Soerjomataram I., Siegel R. L., Torre L. A., Jemal A. (2018). Global cancer statistics 2018: GLOBOCAN estimates of incidence and mortality worldwide for 36 cancers in 185 countries. *CA: A Cancer Journal for Clinicians*.

[2] Siegel R. L., Miller K. D., Jemal A. (2018). Cancer statistics, 2019. *CA: A Cancer Journal for Clinicians*.

[3] The Lancet (2019). Lung cancer: some progress, but still a lot more to do. *The Lancet*.

[4] Siegel R. L., Miller K. D., Jemal A. (2016). Cancer statistics, 2016. *CA: a Cancer Journal for Clinicians*.

[5] Senosain M. F., Massion P. P. (2020). Intratumor heterogeneity in early lung adenocarcinoma. *Frontiers in Oncology*.

[6] Yuan M., Huang L. L., Chen J. H., Wu J., Xu Q. (2019). The emerging treatment landscape of targeted therapy in non-small-cell lung cancer. *Signal Transduction and Targeted Therapy*.

[7] Devarakonda S., Govindan R. (2019). Untangling the evolutionary roots of lung cancer. *Nature Communications*.

[8] Wang J., Liu X., Liang Y. H., Li L. F., Su X. D. (2008). Acceptor substrate binding revealed by crystal structure of human glucosamine-6-phosphate N-acetyltransferase 1. *FEBS Letters*.

[9] Mio T., Yamada-Okabe T., Arisawa M., Yamada-Okabe H. (1999). Saccharomyces cerevisiae GNA1, an essential gene encoding a novel acetyltransferase involved in UDP-N-acetylglucosamine synthesis. *The Journal of Biological Chemistry*.

[10] Boehmelt G., Wakeham A., Elia A. (2000). Decreased UDP-GlcNAc levels abrogate proliferation control in EMeg32-deficient cells. *The EMBO Journal*.

[11] Kaushik A. K., Shojaie A., Panzitt K. (2016). Inhibition of the hexosamine biosynthetic pathway promotes castration-resistant prostate cancer. *Nature Communications*.

[12] Vasaikar S. V., Straub P., Wang J., Zhang B. (2018). LinkedOmics: analyzing multi-omics data within and across 32 cancer types. *Nucleic Acids Research*.

[13] Tang Z., Kang B., Li C., Chen T., Zhang Z. (2019). GEPIA2: an enhanced web server for large-scale expression profiling and interactive analysis. *Nucleic Acids Research*.

[14] Li T., Fan J., Wang B. (2017). TIMER: a web server for comprehensive analysis of tumor-infiltrating immune cells. *Cancer Research*.

[15] Li B., Severson E., Pignon J. C. (2016). Comprehensive analyses of tumor immunity: implications for cancer immunotherapy. *Genome Biology*.

[16] Zhang C., Li Z., Qi F., Hu X., Luo J. (2019). Exploration of the relationships between tumor mutation burden with immune infiltrates in clear cell renal cell carcinoma. *Annals of Translational Medicine*.

[17] Ru B., Wong C. N., Tong Y. (2019). TISIDB: an integrated repository portal for tumor-immune system interactions. *Bioinformatics*.

[18] Chen X., Xu C., Hong S. (2019). Immune cell types and secreted factors contributing to inflammation-to-cancer transition and immune therapy response. *Cell Reports*.

[19] Wang H., Wang X., Xu L., Zhang J., Cao H. (2020). Integrated analysis of the E2F transcription factors across cancer types. *Oncology Reports*.

[20] Zhao M., Li H., Ma Y. (2017). Nanoparticle abraxane possesses impaired proliferation in A549 cells due to the underexpression of glucosamine 6-phosphate N-acetyltransferase 1 (GNPNAT1/GNA1). *International Journal of Nanomedicine*.

[21] Itkonen H. M., Engedal N., Babaie E. (2015). UAP1 is overexpressed in prostate cancer and is protective against inhibitors of N-linked glycosylation. *Oncogene*.

[22] Diril M. K., Ratnacaram C. K., Padmakumar V. C. (2012). Cyclin-dependent kinase 1 (Cdk1) is essential for cell division and suppression of DNA re-replication but not for liver regeneration. *Proceedings of the National Academy of Sciences of the United States of America*.

[23] Prevo R., Pirovano G., Puliyadi R. (2018). CDK1 inhibition sensitizes normal cells to DNA damage in a cell cycle dependent manner. *Cell Cycle*.

[24] Shi Y. X., Zhu T., Zou T. (2016). Prognostic and predictive values of CDK1 and MAD2L1 in lung adenocarcinoma. *Oncotarget*.

[25] Kuang Y., Guo W., Ling J. (2019). Iron-dependent CDK1 activity promotes lung carcinogenesis via activation of the GP130/STAT3 signaling pathway. *Cell Death & Disease*.

[26] Lens S. M., Voest E. E., Medema R. H. (2010). Shared and separate functions of polo-like kinases and aurora kinases in cancer. *Nature Reviews Cancer*.

[27] Ramani P., Nash R., Sowa-Avugrah E., Rogers C. (2015). High levels of polo-like kinase 1 and phosphorylated translationally controlled tumor protein indicate poor prognosis in neuroblastomas. *Journal of Neuro-Oncology*.

[28] Tut T. G., Lim S. H. S., Dissanayake I. U. (2015). Upregulated polo-like kinase 1 expression correlates with inferior survival outcomes in rectal cancer. *PLoS One*.

[29] Zhang R., Shi H., Ren F. (2015). Misregulation of polo-like protein kinase 1, P53 and P21WAF1 in epithelial ovarian cancer suggests poor prognosis. *Oncology Reports*.

[30] Tang A., Gao K., Chu L., Zhang R., Yang J., Zheng J. (2017). Aurora kinases: novel therapy targets in cancers. *Oncotarget*.

[31] Al-Khafaji A. S. K., Davies M. P. A., Risk J. M. (2017). Aurora B expression modulates paclitaxel response in non-small cell lung cancer. *British Journal of Cancer*.

[32] Bertran-Alamillo J., Cattan V., Schoumacher M. (2019). AURKB as a target in non-small cell lung cancer with acquired resistance to anti-EGFR therapy. *Nature Communications*.

[33] Kent L. N., Leone G. (2019). The broken cycle: E2F dysfunction in cancer. *Nature Reviews. Cancer*.

[34] Yang H., Liang S. Q., Schmid R. A., Peng R. W. (2019). New horizons in KRAS-mutant lung cancer: dawn after darkness. *Frontiers in Oncology*.

[35] Kobayashi S., Boggon T. J., Dayaram T. (2005). EGFR mutation and resistance of non-small-cell lung cancer to gefitinib. *The New England Journal of Medicine*.

[36] Skoulidis F., Goldberg M. E., Greenawalt D. M. (2018). STK11/LKB1 mutations and PD-1 inhibitor resistance in KRAS-mutant lung adenocarcinoma. *Cancer Discovery*.

[37] Mogi A., Kuwano H. (2011). TP53 mutations in nonsmall cell lung cancer. *Journal of Biomedicine & Biotechnology*.

[38] Greillier L., Tomasini P., Barlesi F. (2018). The clinical utility of tumor mutational burden in non-small cell lung cancer. *Translational Lung Cancer Research*.

[39] Espinosa A. M., Alfaro A., Roman-Basaure E. (2013). Mitosis is a source of potential markers for screening and survival and therapeutic targets in cervical cancer. *PLoS One*.

[40] Yu Y., Jiang X., Schoch B. S., Carroll R. S., Black P. M., Johnson M. D. (2007). Aberrant splicing of cyclin-dependent kinase-associated protein phosphatase KAP increases proliferation and migration in glioblastoma. *Cancer Research*.

[41] Fan C., Chen L., Huang Q. (2015). Overexpression of major CDKN3 transcripts is associated with poor survival in lung adenocarcinoma. *British Journal of Cancer*.

[42] Gao T., Han Y., Yu L., Ao S., Li Z., Ji J. (2014). CCNA2 is a prognostic biomarker for ER+ breast cancer and tamoxifen resistance. *PLoS One*.

[43] Shibata K., Tanaka S., Shiraishi T., Kitano S., Mori M. (1999). G-protein gamma 7 is down-regulated in cancers and associated with p 27kip1-induced growth arrest. *Cancer Research*.

