# Supplementary material for: Independent Prognostic Potential of GNPNAT1 in Lung Adenocarcinoma
Source: Biomed Res Int. 2020 Oct 29;2020:8851437. doi: 10.1155/2020/8851437 (PMC7648248; doi:10.1155/2020/8851437)

Figure S1

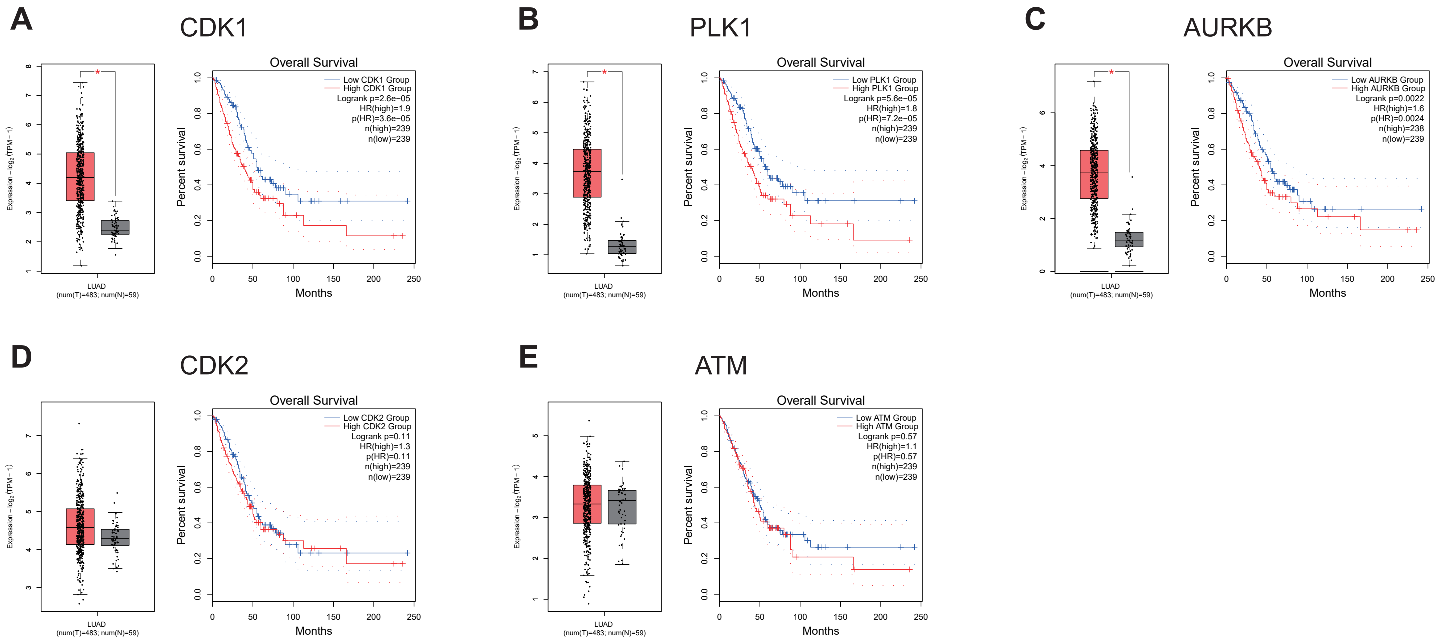

A
CDK1
B
PLK1
C
AURKB
D
CDK2
E
ATM
Overall Survival
Low CDK1 Group
High CDK1 Group
Logrank p=2.6e−05
HR(high)=1.9
p(HR)=3.6e−05
n(high)=239
n(low)=239
Low PLK1 Group
High PLK1 Group
Logrank p=5.6e−05
HR(high)=1.8
p(HR)=7.2e−05
n(high)=239
n(low)=239
Low AURKB Group
High AURKB Group
Logrank p=0.0022
HR(high)=1.6
p(HR)=0.0024
n(high)=238
n(low)=239
Low CDK2 Group
High CDK2 Group
Logrank p=0.11
HR(high)=1.3
p(HR)=0.11
n(high)=239
n(low)=239
Low ATM Group
High ATM Group
Logrank p=0.57
HR(high)=1.1
p(HR)=0.57
n(high)=239
n(low)=239
Percent survival
Months
Expression −log2 (TPM + 1)
LUAD
(num(T)=483; num(N)=59)

Figure S2

A

Survival heat map of B cell markers positively correlated with GNPNAT1

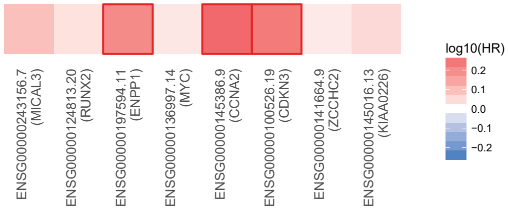

B

Survival heat map of B cell markers negatively correlated with GNPNAT1

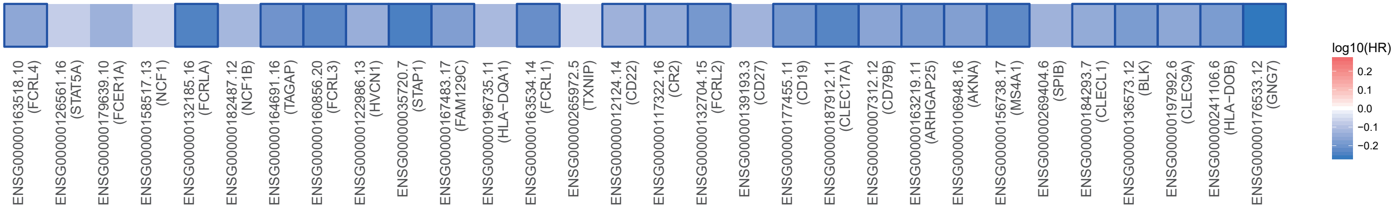

Supplement: Supplementary 1 — Figure S1: expression and survival outcome of top 5 kinase regulators of GNPNAT1 coexpressed genes in TCGA-LUAD cohort. CDK1 (A), PLK1 (B), and AURKB (C) were significantly highly expressed in tumor tissues and had significant associations with overall survival while CDK2 (D) and ATM (E) not. TCGA: The Cancer Genome Atlas; LUAD: lung adenocarcinoma. Figure S2: survival heat maps of B cell markers positively and negatively correlated with GNPNAT1 in TCGA-LUAD cohort. The survival heat maps show the hazard ratios in logarithmic scale (log10) for different genes. The red and blue boxes indicate high and low risks, respectively. Framed rectangles indicate significantly adverse and favorable results in prognostic analysis (p value < 0.05). LUAD: Lung adenocarcinoma; TCGA: The Cancer Genome Atlas; [file 8851437.f1.pdf]
