# Supplementary material for: Independent Prognostic Potential of GNPNAT1 in Lung Adenocarcinoma
Source: Biomed Res Int. 2020 Oct 29;2020:8851437. doi: 10.1155/2020/8851437 (PMC7648248; doi:10.1155/2020/8851437)
Supplement: Supplementary 2 — Table S1: tumor mutation burden (TMB) scores of each sample in the TCGA-LUAD cohort. Table S2: univariate analysis and multivariate analysis of the correlation of GNPNAT1 expression with disease-specific survival among lung adenocarcinoma patients in TCGA-LUAD cohort. Table S3: univariate analysis and multivariate analysis of the correlation of GNPNAT1 expression with progression-free survival among lung adenocarcinoma patients in TCGA-LUAD cohort. Table S4: GNPNAT1 coexpressed genes. Table S5: Gene Ontology term annotation of GNPNAT1 coexpressed genes. Table S6: KEGG annotation of GNPNAT1 coexpressed genes. Table S7: kinases enrichment of GNPNAT1 coexpressed genes. Table S8: miRNA enrichment of GNPNAT1 coexpressed genes. Table S9: transcription factor enrichment of GNPNAT1 coexpressed genes. Table S10: correlation of GNPNAT1 with various immune signatures in TCGA-LUAD cohort. [file 8851437.f2.pdf]

Table S1. Tumor mutation burden (TMB) scores of each sample in the TCGA-LUAD cohort.

id	TMB
TCGA-78-7153-01A-11D-2036-08	2.184210526
TCGA-73-4670-01A-01D-1265-08	11.65789474
TCGA-78-7145-01A-11D-2036-08	6.052631579
TCGA-49-4506-01A-01D-1265-08	2.710526316
TCGA-17-Z055-01A-01W-0747-08	11.92105263
TCGA-55-A491-01A-11D-A24D-08	10.5
TCGA-55-7913-01B-11D-2238-08	9.657894737
TCGA-05-4402-01A-01D-1265-08	1.710526316
TCGA-55-8203-01A-11D-2238-08	12.86842105
TCGA-91-6829-01A-21D-1855-08	18.34210526
TCGA-97-A4M7-01A-11D-A24P-08	8
TCGA-44-7659-01A-11D-2063-08	4.421052632
TCGA-75-6214-01A-41D-1945-08	19.65789474
TCGA-62-A46P-01A-11D-A24D-08	8.105263158
TCGA-05-4398-01A-01D-1265-08	18.13157895
TCGA-78-7540-01A-11D-2063-08	0.973684211
TCGA-L9-A50W-01A-12D-A397-08	0.815789474
TCGA-64-1681-01A-11D-2063-08	1.368421053
TCGA-17-Z028-01A-01W-0746-08	12.94736842

TCGA-55-8507-01A-11D-2393-08	29.63157895
TCGA-NJ-A7XG-01A-12D-A397-08	0.894736842
TCGA-49-4490-01A-21D-1855-08	0.710526316
TCGA-55-8506-01A-11D-2393-08	51.84210526
TCGA-44-5644-01A-21D-2036-08	30
TCGA-86-8054-01A-11D-2238-08	6.815789474
TCGA-44-A4SS-01A-11D-A24P-08	10.07894737
TCGA-49-4494-01A-01D-1265-08	5.394736842
TCGA-55-8092-01A-11D-2238-08	16.07894737
TCGA-69-7764-01A-11D-2167-08	2.447368421
TCGA-86-7713-01A-11D-2063-08	4.868421053
TCGA-44-2662-01A-01D-A271-08	4.657894737
TCGA-91-6828-01A-11D-1855-08	7.342105263
TCGA-55-8094-01A-11D-2238-08	14.36842105
TCGA-17-Z025-01A-01W-0746-08	6.789473684
TCGA-97-A4LX-01A-11D-A24P-08	2.789473684
TCGA-44-2659-01A-01D-0969-08	12.34210526
TCGA-53-7624-01A-11D-2063-08	25.60526316
TCGA-75-5126-01A-01D-1753-08	14.39473684
TCGA-78-7537-01A-11D-2063-08	4.315789474
TCGA-55-6983-01A-11D-1945-08	2.289473684
TCGA-35-3615-01A-01D-1040-01	3.736842105

TCGA-97-7941-01A-11D-2184-08	2.184210526
TCGA-05-5425-01A-02D-1625-08	14.42105263
TCGA-78-7161-01A-11D-2036-08	5.473684211
TCGA-69-7760-01A-11D-2167-08	2.026315789
TCGA-71-6725-01A-11D-1855-08	1.5
TCGA-50-5044-01A-21D-1855-08	2.394736842
TCGA-55-8208-01A-11D-2238-08	3.236842105
TCGA-75-6212-01A-11D-1753-08	0.526315789
TCGA-50-6595-01A-12D-1855-08	1.394736842
TCGA-55-A57B-01A-12D-A397-08	0.894736842
TCGA-86-8075-01A-11D-2238-08	0.973684211
TCGA-L9-A8F4-01A-11D-A397-08	21.60526316
TCGA-55-6642-01A-11D-1855-08	3.815789474
TCGA-05-4384-01A-01D-1753-08	3.5
TCGA-55-A48X-01A-11D-A24D-08	8.5
TCGA-17-Z016-01A-01W-0746-08	9.815789474
TCGA-44-3917-01A-01D-A271-08	6.026315789
TCGA-05-4249-01A-01D-1105-08	9.789473684
TCGA-17-Z057-01A-01W-0747-08	10.76315789
TCGA-49-4505-01A-01D-1931-08	5.815789474
TCGA-69-8254-01A-11D-2284-08	2.289473684
TCGA-49-4512-01A-21D-1855-08	0.921052632

TCGA-J2-A4AD-01A-11D-A24D-08	7.973684211
TCGA-91-A4BD-01A-11D-A24D-08	0.736842105
TCGA-55-6969-01A-11D-1945-08	11.21052632
TCGA-86-7954-01A-11D-2184-08	4.473684211
TCGA-97-8172-01A-11D-2284-08	7.289473684
TCGA-97-8547-01A-11D-2393-08	1.605263158
TCGA-05-5423-01A-01D-1625-08	4.5
TCGA-55-8097-01A-11D-2238-08	3.026315789
TCGA-NJ-A4YF-01A-12D-A25L-08	18.57894737
TCGA-86-8279-01A-11D-2284-08	15.76315789
TCGA-55-7903-01A-11D-2167-08	4.026315789
TCGA-05-4397-01A-01D-1265-08	25.86842105
TCGA-J2-A4AG-01A-11D-A24D-08	1.789473684
TCGA-17-Z009-01A-01W-0746-08	0.184210526
TCGA-17-Z045-01A-01W-0746-08	19.89473684
TCGA-62-A46O-01A-11D-A24D-08	27.28947368
TCGA-44-6774-01A-21D-1855-08	8.578947368
TCGA-55-8621-01A-11D-2393-08	0.184210526
TCGA-17-Z002-01A-01W-0746-08	0.105263158
TCGA-49-AARO-01A-12D-A410-08	15.76315789
TCGA-67-3770-01A-01D-0969-08	6.736842105
TCGA-NJ-A4YI-01A-11D-A25L-08	14.13157895

TCGA-17-Z010-01A-01W-0746-08	8.157894737
TCGA-MP-A4TF-01A-11D-A25L-08	11.81578947
TCGA-44-2655-01A-01D-1553-08	3.605263158
TCGA-95-A4VK-01A-11D-A25L-08	7.789473684
TCGA-67-3772-01A-01W-0928-08	1.368421053
TCGA-55-6986-01A-11D-1945-08	0.605263158
TCGA-NJ-A55O-01A-11D-A25L-08	2.289473684
TCGA-64-5775-01A-01D-1625-08	15.47368421
TCGA-78-7535-01A-11D-2063-08	5.289473684
TCGA-73-4668-01A-01D-1265-08	17.71052632
TCGA-62-8397-01A-11D-2323-08	0.894736842
TCGA-05-4432-01A-01D-1265-08	14.76315789
TCGA-MP-A4T2-01A-11D-A24P-08	1.710526316
TCGA-91-6830-01A-11D-1945-08	4.526315789
TCGA-91-A4BC-01A-11D-A24D-08	10.65789474
TCGA-78-7155-01A-11D-2036-08	48.10526316
TCGA-S2-AA1A-01A-12D-A397-08	3.157894737
TCGA-05-4410-01A-21D-1855-08	28.07894737
TCGA-75-5122-01A-01D-1753-08	2.368421053
TCGA-49-6744-01A-11D-1855-08	3.842105263
TCGA-4B-A93V-01A-11D-A397-08	4.368421053
TCGA-05-4395-01A-01D-1265-08	8.578947368

TCGA-50-5932-01A-11D-1753-08	2.342105263
TCGA-97-A4M2-01A-12D-A24P-08	0.131578947
TCGA-69-7973-01A-11D-2184-08	8.052631579
TCGA-55-8090-01A-11D-2238-08	2.815789474
TCGA-78-8648-01A-11D-2393-08	0.026315789
TCGA-38-6178-01A-11D-1753-08	1.052631579
TCGA-NJ-A4YQ-01A-11D-A25L-08	28.97368421
TCGA-05-4250-01A-01D-1105-08	10.97368421
TCGA-78-7220-01A-11D-2036-08	26.5
TCGA-78-7150-01A-21D-2036-08	12.31578947
TCGA-64-5774-01A-01D-1625-08	4.289473684
TCGA-J2-8192-01A-11D-2238-08	1.736842105
TCGA-78-7162-01A-21D-2063-08	1.526315789
TCGA-44-6778-01A-11D-1855-08	11.13157895
TCGA-05-5428-01A-01D-1625-08	11.28947368
TCGA-55-A48Z-01A-12D-A24P-08	1.315789474
TCGA-91-7771-01A-11D-2167-08	4.052631579
TCGA-NJ-A55A-01A-11D-A25L-08	1.052631579
TCGA-62-A46R-01A-11D-A24D-08	4.552631579
TCGA-86-8672-01A-21D-2393-08	8.236842105
TCGA-73-4666-01A-01D-1265-08	11.92105263
TCGA-97-7552-01A-11D-2036-08	0.552631579

TCGA-86-A4JF-01A-11D-A24P-08	34.92105263
TCGA-44-7670-01A-11D-2063-08	33.42105263
TCGA-55-6982-01A-11D-1945-08	4.657894737
TCGA-62-A46Y-01A-11D-A24D-08	1.026315789
TCGA-17-Z020-01A-01W-0746-08	2.078947368
TCGA-91-6840-01A-11D-1945-08	3.447368421
TCGA-17-Z005-01A-01W-0746-08	3.710526316
TCGA-49-4507-01A-01D-1265-08	5.684210526
TCGA-55-7907-01A-11D-2167-08	35.28947368
TCGA-62-8399-01A-21D-2323-08	15.15789474
TCGA-49-4487-01A-21D-1855-08	6.842105263
TCGA-86-8673-01A-11D-2393-08	13.47368421
TCGA-67-6217-01A-11D-1753-08	4.578947368
TCGA-55-6980-01A-11D-1945-08	0.315789474
TCGA-55-A4DF-01A-11D-A24D-08	21.28947368
TCGA-49-AAR3-01A-11D-A410-08	1.947368421
TCGA-44-A47A-01A-21D-A24D-08	5
TCGA-55-6984-01A-11D-1945-08	0.657894737
TCGA-L9-A444-01A-21D-A24D-08	14.92105263
TCGA-93-A4JQ-01A-11D-A24P-08	1.289473684
TCGA-MP-A4TE-01A-22D-A25L-08	4.289473684
TCGA-05-4426-01A-01D-1265-08	1.368421053

TCGA-05-4425-01A-01D-1753-08	1.210526316
TCGA-55-7227-01A-11D-2036-08	5.052631579
TCGA-97-8176-01A-11D-2393-08	4.263157895
TCGA-73-4675-01A-01D-1265-08	1.921052632
TCGA-55-6975-01A-11D-1945-08	2.342105263
TCGA-44-6147-01A-31D-A27T-08	1.447368421
TCGA-78-7633-01A-11D-2063-08	3.552631579
TCGA-44-A47F-01A-11D-A24D-08	2.105263158
TCGA-MP-A4T4-01A-11D-A25L-08	8.605263158
TCGA-17-Z033-01A-01W-0746-08	3.210526316
TCGA-17-Z017-01A-01W-0746-08	6.605263158
TCGA-55-6979-01A-11D-1945-08	2.657894737
TCGA-93-A4JP-01A-11D-A24P-08	0.552631579
TCGA-L4-A4E5-01A-11D-A24P-08	10.47368421
TCGA-55-6985-01A-11D-1945-08	12.28947368
TCGA-44-3918-01A-01D-A271-08	22.42105263
TCGA-86-8358-01A-11D-2323-08	34.34210526
TCGA-62-A46U-01A-11D-A24D-08	1.078947368
TCGA-86-7955-01A-11D-2184-08	9.394736842
TCGA-49-AAR9-01A-21D-A410-08	20.71052632
TCGA-86-A4P7-01A-11D-A24P-08	1.052631579
TCGA-93-8067-01A-11D-2284-08	14.94736842

TCGA-78-7167-01A-11D-2063-08	5.552631579
TCGA-91-8497-01A-11D-2393-08	1.210526316
TCGA-67-6216-01A-11D-1753-08	0.5
TCGA-97-A4M5-01A-11D-A24P-08	3.210526316
TCGA-49-AAR0-01A-21D-A397-08	2.736842105
TCGA-55-A4DG-01A-11D-A24D-08	19.26315789
TCGA-17-Z062-01A-01W-0747-08	8.947368421
TCGA-78-7149-01A-11D-2036-08	3.815789474
TCGA-50-5051-01A-21D-1855-08	3.184210526
TCGA-O1-A52J-01A-11D-A25L-08	5.078947368
TCGA-55-8616-01A-11D-2393-08	14.18421053
TCGA-35-4122-01A-01D-1105-08	6.973684211
TCGA-MP-A4T8-01A-11D-A24P-08	3.631578947
TCGA-44-2665-01A-01D-A271-08	0.921052632
TCGA-55-8505-01A-11D-2393-08	1
TCGA-50-5068-01A-01D-1625-08	2.263157895
TCGA-17-Z051-01A-01W-0747-08	7.736842105
TCGA-99-8032-01A-11D-2238-08	10.84210526
TCGA-86-8073-01A-11D-2238-08	39.76315789
TCGA-55-7995-01A-11D-2184-08	20.34210526
TCGA-17-Z049-01A-01W-0746-08	19.05263158
TCGA-MN-A4N1-01A-11D-A24P-08	13.02631579

TCGA-17-Z014-01A-01W-0746-08	11.39473684
TCGA-86-8055-01A-11D-2238-08	0.815789474
TCGA-55-6981-01A-11D-1945-08	1.342105263
TCGA-78-7163-01A-12D-2063-08	0.578947368
TCGA-55-8091-01A-11D-2238-08	0.552631579
TCGA-05-4396-01A-21D-1855-08	7.894736842
TCGA-50-5946-01A-11D-1753-08	19.76315789
TCGA-38-A44F-01A-11D-A24D-08	0.157894737
TCGA-86-8669-01A-11D-2393-08	3.736842105
TCGA-NJ-A4YP-01A-11D-A25L-08	14.84210526
TCGA-44-2668-01A-01D-A271-08	8.368421053
TCGA-55-A493-01A-11D-A24D-08	5.236842105
TCGA-50-5045-01A-01D-1625-08	6.315789474
TCGA-95-A4VN-01A-11D-A25L-08	9.947368421
TCGA-93-7347-01A-11D-2184-08	2.789473684
TCGA-44-2657-01A-01D-1105-08	10.23684211
TCGA-67-3771-01A-01D-1040-01	30.97368421
TCGA-55-8204-01A-11D-2238-08	5.868421053
TCGA-38-4630-01A-01D-1265-08	4
TCGA-75-5125-01A-01D-1753-08	4.236842105
TCGA-86-6851-01A-11D-1945-08	25.23684211
TCGA-38-7271-01A-11D-2036-08	0.815789474

TCGA-80-5607-01A-31D-1945-08	3.763157895
TCGA-17-Z021-01A-01W-0746-08	2.631578947
TCGA-99-8033-01A-11D-2238-08	4.763157895
TCGA-86-8074-01A-11D-2238-08	1.921052632
TCGA-86-8585-01A-11D-2393-08	16.55263158
TCGA-MP-A4SV-01A-11D-A24P-08	6.5
TCGA-50-6592-01A-11D-1753-08	7.210526316
TCGA-17-Z056-01A-01W-0747-08	8.578947368
TCGA-49-4510-01A-01D-1265-08	1.342105263
TCGA-L9-A443-01A-12D-A24D-08	7.078947368
TCGA-50-6597-01A-11D-1855-08	1.578947368
TCGA-49-AAR4-01A-12D-A410-08	9
TCGA-97-7547-01A-11D-2036-08	4.789473684
TCGA-38-4626-01A-01D-1553-08	2.263157895
TCGA-75-6205-01A-11D-1753-08	0.605263158
TCGA-67-4679-01B-01D-1753-08	8.763157895
TCGA-55-1596-01A-01D-1040-01	5.684210526
TCGA-MP-A4TC-01A-11D-A24P-08	7.526315789
TCGA-05-5420-01A-01D-1625-08	1.894736842
TCGA-97-8552-01A-11D-2393-08	0.631578947
TCGA-86-7701-01A-11D-2167-08	5.394736842
TCGA-44-7671-01A-11D-2063-08	7.368421053

TCGA-55-1594-01A-01D-1040-01	6.157894737
TCGA-86-7953-01A-11D-2184-08	1.315789474
TCGA-50-5933-01A-11D-1753-08	13.07894737
TCGA-78-7146-01A-11D-2036-08	11.52631579
TCGA-MP-A4TA-01A-21D-A24P-08	9.131578947
TCGA-78-7152-01A-11D-2036-08	4.947368421
TCGA-55-8615-01A-11D-2393-08	3.842105263
TCGA-91-8499-01A-11D-2393-08	10.28947368
TCGA-53-7626-01A-12D-2063-08	11.21052632
TCGA-38-4625-01A-01D-1553-08	17.13157895
TCGA-55-7574-01A-11D-2036-08	3.315789474
TCGA-97-7553-01A-21D-2036-08	0.789473684
TCGA-50-5931-01A-11D-1753-08	9.184210526
TCGA-05-4415-01A-22D-1855-08	6.184210526
TCGA-97-7937-01A-11D-2167-08	13.39473684
TCGA-62-A470-01A-11D-A24D-08	3.5
TCGA-17-Z030-01A-01W-0746-08	13.13157895
TCGA-95-7947-01A-11D-2184-08	16.60526316
TCGA-55-8206-01A-11D-2238-08	0.789473684
TCGA-MP-A5C7-01A-11D-A25L-08	2.315789474
TCGA-91-6836-01A-21D-1855-08	15.07894737
TCGA-17-Z050-01A-01W-0747-08	4.657894737

TCGA-44-A4SU-01A-11D-A24P-08	3.552631579
TCGA-78-7159-01A-11D-2036-08	9.184210526
TCGA-05-4405-01A-21D-1855-08	9.289473684
TCGA-73-A9RS-01A-11D-A410-08	28.92105263
TCGA-73-7498-01A-12D-2184-08	3.552631579
TCGA-05-4434-01A-01D-1265-08	0.921052632
TCGA-55-8302-01A-11D-2323-08	16.86842105
TCGA-86-8674-01A-21D-2393-08	9.210526316
TCGA-55-8299-01A-11D-2284-08	0.421052632
TCGA-55-A48Y-01A-11D-A24D-08	8.394736842
TCGA-17-Z023-01A-01W-0746-08	11.23684211
TCGA-50-5930-01A-11D-1753-08	32.02631579
TCGA-55-8207-01A-11D-2238-08	9.921052632
TCGA-J2-8194-01A-11D-2238-08	4.5
TCGA-95-7944-01A-11D-2184-08	7.342105263
TCGA-MP-A4TI-01A-21D-A24P-08	4.210526316
TCGA-55-8512-01A-11D-2393-08	1.157894737
TCGA-99-AA5R-01A-11D-A397-08	0.131578947
TCGA-93-A4JN-01A-11D-A24P-08	1.815789474
TCGA-64-5779-01A-01D-1625-08	10.55263158
TCGA-55-5899-01A-11D-1625-08	13.55263158
TCGA-05-5429-01A-01D-1625-08	0.947368421

TCGA-55-8619-01A-11D-2393-08	0.131578947
TCGA-73-4659-01A-01D-1265-08	6.526315789
TCGA-49-AAR2-01A-11D-A397-08	12.31578947
TCGA-97-7546-01A-11D-2036-08	8.184210526
TCGA-55-7911-01A-11D-2167-08	6.052631579
TCGA-17-Z013-01A-01W-0746-08	1.789473684
TCGA-44-3919-01A-02D-1458-08	1.026315789
TCGA-17-Z058-01A-01W-0747-08	7.657894737
TCGA-49-AAQV-01A-11D-A397-08	0.894736842
TCGA-86-7714-01A-12D-2167-08	0.131578947
TCGA-64-5815-01A-01D-1625-08	4.894736842
TCGA-64-1677-01A-01W-0928-08	5.289473684
TCGA-78-7158-01A-11D-2036-08	7.184210526
TCGA-44-2656-01A-41D-A27T-08	20.63157895
TCGA-17-Z043-01A-01W-0746-08	6.236842105
TCGA-91-6847-01A-11D-1945-08	2.052631579
TCGA-55-8614-01A-11D-2393-08	6.447368421
TCGA-64-1679-01A-21D-2063-08	13.71052632
TCGA-NJ-A55R-01A-11D-A25L-08	7.473684211
TCGA-55-6978-01A-11D-1945-08	0.526315789
TCGA-78-7154-01A-11D-2036-08	7.763157895
TCGA-17-Z036-01A-01W-0746-08	2.289473684

TCGA-17-Z048-01A-01W-0746-08	0.842105263
TCGA-50-6593-01A-11D-1753-08	8.263157895
TCGA-17-Z054-01A-01W-0747-08	0.210526316
TCGA-50-8460-01A-11D-2323-08	0.736842105
TCGA-80-5608-01A-31D-1945-08	5.184210526
TCGA-50-6594-01A-11D-1753-08	16.63157895
TCGA-55-6712-01A-11D-1855-08	2.973684211
TCGA-55-7284-01B-11D-2238-08	1.131578947
TCGA-17-Z000-01A-01W-0746-08	3.421052632
TCGA-93-7348-01A-21D-2036-08	2.078947368
TCGA-17-Z022-01A-01W-0746-08	38.15789474
TCGA-55-7727-01A-11D-2167-08	6.236842105
TCGA-69-8255-01A-11D-2284-08	8.657894737
TCGA-MN-A4N5-01A-11D-A24P-08	13.36842105
TCGA-17-Z060-01A-01W-0747-08	8.578947368
TCGA-53-A4EZ-01A-12D-A24P-08	16.76315789
TCGA-64-5778-01A-01D-1625-08	14.55263158
TCGA-55-8514-01A-11D-2393-08	8.578947368
TCGA-50-6591-01A-11D-1753-08	1.315789474
TCGA-67-3774-01A-01D-1040-01	3.473684211
TCGA-17-Z007-01A-01W-0746-08	1.894736842
TCGA-55-7573-01A-11D-2036-08	1.421052632

TCGA-55-6971-01A-11D-1945-08	2.394736842
TCGA-78-7166-01A-12D-2063-08	6.868421053
TCGA-MP-A4SY-01A-21D-A24P-08	3.105263158
TCGA-17-Z037-01A-01W-0746-08	3.736842105
TCGA-49-6745-01A-11D-1855-08	3
TCGA-97-A4M1-01A-11D-A24P-08	0.921052632
TCGA-38-4631-01A-01D-1753-08	21.92105263
TCGA-55-7910-01A-11D-2167-08	8.894736842
TCGA-97-8174-01A-11D-2284-08	4.078947368
TCGA-62-A472-01A-11D-A24D-08	1.657894737
TCGA-55-6543-01A-11D-1753-08	0.578947368
TCGA-86-7711-01A-11D-2063-08	6.157894737
TCGA-17-Z026-01A-01W-0746-08	21.42105263
TCGA-69-7763-01A-11D-2167-08	1.947368421
TCGA-69-7979-01A-11D-2184-08	43.23684211
TCGA-95-7948-01A-11D-2184-08	4
TCGA-50-5049-01A-01D-1625-08	16.78947368
TCGA-78-7156-01A-11D-2036-08	8.131578947
TCGA-05-4390-01A-02D-1753-08	16.68421053
TCGA-50-7109-01A-11D-2036-08	7
TCGA-78-7160-01A-11D-2036-08	1.026315789
TCGA-67-3773-01A-01D-1040-01	3.210526316

TCGA-62-8394-01A-11D-2323-08	2.342105263
TCGA-62-A471-01A-12D-A24D-08	3.236842105
TCGA-55-A494-01A-11D-A24P-08	7.473684211
TCGA-44-7661-01A-11D-2063-08	6.105263158
TCGA-55-8096-01A-11D-2238-08	3.131578947
TCGA-99-8025-01A-11D-2238-08	14.02631579
TCGA-44-2661-01A-01D-1105-08	1
TCGA-44-5645-01A-51D-A27T-08	1.105263158
TCGA-73-4658-01A-01D-1753-08	5.052631579
TCGA-05-4417-01A-22D-1855-08	8.763157895
TCGA-86-8671-01A-11D-2393-08	0.078947368
TCGA-44-6144-01A-11D-1753-08	6.105263158
TCGA-44-6776-01A-11D-1855-08	6.105263158
TCGA-17-Z044-01A-01W-0746-08	3.894736842
TCGA-78-7148-01A-11D-2036-08	6.105263158
TCGA-MP-A4TJ-01A-51D-A25L-08	1.736842105
TCGA-50-5944-01A-11D-1753-08	2.078947368
TCGA-95-7043-01A-11D-1945-08	34.05263158
TCGA-78-7147-01A-11D-2036-08	10.23684211
TCGA-78-8655-01A-11D-2393-08	3.289473684
TCGA-05-4403-01A-01D-1265-08	4.184210526
TCGA-64-5781-01A-01D-1625-08	31.07894737

TCGA-93-A4JO-01A-21D-A24P-08	0.657894737
TCGA-50-5066-01A-01D-1625-08	6.342105263
TCGA-05-4433-01A-22D-1855-08	1.131578947
TCGA-73-4662-01A-01D-1265-08	5.973684211
TCGA-55-7815-01A-11D-2167-08	3.684210526
TCGA-55-8510-01A-11D-2393-08	8.289473684
TCGA-44-8117-01A-11D-2238-08	23.52631579
TCGA-55-7576-01A-11D-2063-08	10.02631579
TCGA-78-7539-01A-11D-2063-08	13.31578947
TCGA-55-1592-01A-01D-0969-08	15.68421053
TCGA-17-Z003-01A-01W-0746-08	6.263157895
TCGA-50-6590-01A-12D-1855-08	22.44736842
TCGA-97-8175-01A-11D-2284-08	0.763157895
TCGA-17-Z015-01A-01W-0746-08	15.81578947
TCGA-86-6562-01A-11D-1753-08	1.131578947
TCGA-78-7536-01A-11D-2063-08	19.36842105
TCGA-55-8089-01A-11D-2238-08	32.39473684
TCGA-44-7667-01A-31D-2063-08	12.86842105
TCGA-55-7283-01A-11D-2036-08	5.578947368
TCGA-49-AARQ-01A-11D-A410-08	20.78947368
TCGA-44-6775-01A-31D-A27T-08	1.368421053
TCGA-44-8119-01A-11D-2238-08	18.5

TCGA-62-8395-01A-11D-2323-08	1.552631579
TCGA-62-8402-01A-11D-2323-08	1.736842105
TCGA-97-7554-01A-11D-2036-08	12.44736842
TCGA-MP-A4T9-01A-11D-A24P-08	0.394736842
TCGA-17-Z052-01A-01W-0747-08	1.421052632
TCGA-17-Z001-01A-01W-0746-08	6.342105263
TCGA-J2-A4AE-01A-21D-A24D-08	1.052631579
TCGA-75-6207-01A-11D-1753-08	1.552631579
TCGA-75-6203-01A-11D-1753-08	0.289473684
TCGA-78-8640-01A-11D-2393-08	20.15789474
TCGA-05-4422-01A-01D-1265-08	1.210526316
TCGA-17-Z061-01A-01W-0747-08	5.710526316
TCGA-05-4430-01A-02D-1265-08	5.894736842
TCGA-49-4501-01A-01D-1265-08	1.184210526
TCGA-38-4628-01A-01D-1265-08	3.210526316
TCGA-17-Z004-01A-01W-0746-08	0.605263158
TCGA-75-6206-01A-11D-1753-08	2.526315789
TCGA-17-Z027-01A-01W-0746-08	2.736842105
TCGA-55-7728-01A-11D-2184-08	2.789473684
TCGA-44-6779-01A-11D-1855-08	2.947368421
TCGA-44-8120-01A-11D-2238-08	18.94736842
TCGA-35-4123-01A-01D-1105-08	8.657894737

TCGA-69-7978-01A-11D-2184-08	12.55263158
TCGA-49-4488-01A-01D-1753-08	8.868421053
TCGA-91-6835-01A-11D-1855-08	1.263157895
TCGA-91-8496-01A-11D-2393-08	0.710526316
TCGA-49-4514-01A-21D-1855-08	9.342105263
TCGA-75-5147-01A-01D-1625-08	1
TCGA-50-5072-01A-21D-1855-08	4.684210526
TCGA-50-8457-01A-11D-2323-08	1.394736842
TCGA-95-7567-01A-11D-2063-08	24.31578947
TCGA-69-A59K-01A-11D-A25L-08	14.55263158
TCGA-69-8253-01A-11D-2284-08	4.552631579
TCGA-86-8668-01A-11D-2393-08	0.842105263
TCGA-67-6215-01A-11D-1753-08	2.605263158
TCGA-97-8177-01A-11D-2284-08	0.605263158
TCGA-MP-A4TD-01A-32D-A25L-08	1.947368421
TCGA-49-AARN-01A-21D-A410-08	13.63157895
TCGA-86-A456-01A-11D-A24D-08	8.342105263
TCGA-62-A46S-01A-11D-A24D-08	3.263157895
TCGA-44-A47G-01A-21D-A24D-08	0.815789474
TCGA-MP-A4TK-01A-11D-A24P-08	8.763157895
TCGA-78-7542-01A-21D-2063-08	11.15789474
TCGA-80-5611-01A-01D-1625-08	5.736842105

TCGA-91-6848-01A-11D-1945-08	17.13157895
TCGA-38-4632-01A-01D-1753-08	13.71052632
TCGA-55-1595-01A-01D-0969-08	9.473684211
TCGA-49-6743-01A-11D-1855-08	20
TCGA-86-8056-01A-11D-2238-08	7.105263158
TCGA-17-Z053-01A-01W-0747-08	10.07894737
TCGA-97-A4M6-01A-11D-A24P-08	0.815789474
TCGA-55-7725-01A-11D-2167-08	4.157894737
TCGA-86-8076-01A-31D-2238-08	1.315789474
TCGA-69-7765-01A-11D-2167-08	15.39473684
TCGA-MP-A4T6-01A-32D-A25L-08	1.947368421
TCGA-55-7726-01A-11D-2167-08	3.026315789
TCGA-17-Z059-01A-01W-0747-08	4.736842105
TCGA-MP-A4SW-01A-21D-A24P-08	1.447368421
TCGA-55-7724-01A-11D-2167-08	3.289473684
TCGA-69-7761-01A-11D-2167-08	0.684210526
TCGA-44-A47B-01A-11D-A24D-08	1.5
TCGA-91-6849-01A-11D-1945-08	3.789473684
TCGA-L9-A7SV-01A-11D-A397-08	48.42105263
TCGA-64-1676-01A-01D-0969-08	18.21052632
TCGA-97-7938-01A-11D-2167-08	11.55263158
TCGA-97-8171-01A-11D-2284-08	1.578947368

TCGA-05-4424-01A-22D-1855-08	17.23684211
TCGA-55-7914-01A-11D-2167-08	5.052631579
TCGA-75-7025-01A-12D-1945-08	0.763157895
TCGA-L9-A5IP-01A-21D-A397-08	9.105263158
TCGA-49-4486-01A-01D-1265-08	3.631578947
TCGA-75-7031-01A-11D-1945-08	6.394736842
TCGA-44-2666-01A-41D-A27T-08	0.657894737
TCGA-55-6968-01A-11D-1945-08	13.5
TCGA-75-7027-01A-11D-1945-08	7.763157895
TCGA-73-7499-01A-11D-2184-08	1.552631579
TCGA-NJ-A4YG-01A-22D-A25L-08	3.210526316
TCGA-95-7039-01A-11D-1945-08	35.92105263
TCGA-55-7281-01A-11D-2036-08	13.23684211
TCGA-50-5936-01A-11D-1625-08	3.131578947
TCGA-97-A4M0-01A-11D-A24P-08	11.86842105
TCGA-95-8494-01A-11D-2323-08	3
TCGA-55-8511-01A-11D-2393-08	15.02631579
TCGA-95-7562-01A-11D-2238-08	12.42105263
TCGA-62-8398-01A-11D-2323-08	2.526315789
TCGA-49-AARE-01A-11D-A410-08	42.86842105
TCGA-55-8087-01A-11D-2238-08	0.763157895
TCGA-44-7669-01A-21D-2063-08	13.15789474

TCGA-86-8281-01A-11D-2284-08	7.236842105
TCGA-50-8459-01A-11D-2323-08	2.421052632
TCGA-69-7974-01A-11D-2184-08	13.81578947
TCGA-55-6987-01A-11D-1945-08	4
TCGA-MN-A4N4-01A-12D-A24P-08	36.71052632
TCGA-44-5643-01A-01D-1625-08	4.026315789
TCGA-55-8301-01A-11D-2284-08	6.578947368
TCGA-78-8662-01A-11D-2393-08	36.31578947
TCGA-MP-A4TH-01A-31D-A25L-08	3.105263158
TCGA-17-Z041-01A-01W-0746-08	3.026315789
TCGA-73-4677-01A-01D-1265-08	7.710526316
TCGA-49-6761-01A-31D-1945-08	5.026315789
TCGA-44-7660-01A-11D-2063-08	9.868421053
TCGA-55-8620-01A-11D-2393-08	15.23684211
TCGA-99-7458-01A-11D-2036-08	12.89473684
TCGA-05-4389-01A-01D-1265-08	5.947368421
TCGA-35-5375-01A-01D-1625-08	12.65789474
TCGA-44-6777-01A-11D-1855-08	8.710526316
TCGA-50-5935-01A-11D-1753-08	1.105263158
TCGA-44-A479-01A-31D-A24D-08	7
TCGA-17-Z042-01A-01W-0746-08	13.84210526
TCGA-50-5055-01A-01D-1625-08	0.131578947

TCGA-95-A4VP-01A-21D-A25L-08	3.342105263
TCGA-97-8179-01A-11D-2284-08	5.394736842
TCGA-91-6831-01A-11D-1855-08	8.184210526
TCGA-95-8039-01A-11D-2238-08	2.736842105
TCGA-44-4112-01A-31D-A27T-08	12.92105263
TCGA-17-Z032-01A-01W-0746-08	2.973684211
TCGA-55-6970-01A-11D-1945-08	5.157894737
TCGA-53-7813-01A-11D-2167-08	4.263157895
TCGA-55-A492-01A-11D-A24D-08	7.447368421
TCGA-75-5146-01A-01D-1625-08	5.710526316
TCGA-99-8028-01A-11D-2238-08	4.078947368
TCGA-86-8280-01A-11D-2284-08	1
TCGA-38-4627-01A-01D-1553-08	0.5
TCGA-05-4244-01A-01D-1105-08	6.657894737
TCGA-17-Z018-01A-01W-0746-08	7.473684211
TCGA-44-3398-01A-01D-1105-08	3.026315789
TCGA-55-6972-01A-11D-1945-08	7.868421053
TCGA-55-A490-01A-11D-A24D-08	38.05263158
TCGA-44-3396-01A-01D-1553-08	3.789473684
TCGA-55-8085-01A-11D-2238-08	8.368421053
TCGA-44-7662-01A-11D-2063-08	15.42105263
TCGA-55-7994-01A-11D-2184-08	47.05263158

TCGA-05-4382-01A-01D-1931-08	38.65789474
TCGA-64-1680-01A-02D-0969-08	1.184210526
TCGA-MP-A4T7-01A-11D-A24P-08	3.5
TCGA-86-8278-01A-11D-2284-08	1.789473684
TCGA-71-8520-01A-11D-2393-08	2.552631579
TCGA-L9-A743-01A-43D-A397-08	1.763157895
TCGA-17-Z019-01A-01W-0746-08	0.105263158
TCGA-55-8205-01A-11D-2238-08	12.18421053
TCGA-73-4676-01A-01D-1753-08	2.815789474
TCGA-05-5715-01A-01D-1625-08	3.605263158
TCGA-50-6673-01A-11D-1945-08	1.5
TCGA-17-Z047-01A-01W-0747-08	0.421052632
TCGA-05-4427-01A-21D-1855-08	28.63157895
TCGA-49-6767-01A-11D-1855-08	13.52631579
TCGA-05-4418-01A-01D-1265-08	8.236842105
TCGA-78-8660-01A-11D-2393-08	6.394736842
TCGA-50-5942-01A-21D-1753-08	0.921052632
TCGA-86-8359-01A-11D-2323-08	4.736842105
TCGA-49-6742-01A-11D-1855-08	7.5
TCGA-44-6146-01A-31D-A27T-08	0.815789474
TCGA-97-A4M3-01A-11D-A24P-08	5.921052632
TCGA-05-4420-01A-01D-1265-08	8.763157895

TCGA-55-7570-01A-11D-2036-08	11.63157895
TCGA-69-7980-01A-11D-2184-08	23.07894737
TCGA-50-5939-01A-11D-1625-08	2.105263158
TCGA-83-5908-01A-21D-2284-08	5.973684211
TCGA-55-8508-01A-11D-2393-08	5.184210526
TCGA-44-7672-01A-11D-2063-08	3.342105263
TCGA-50-5941-01A-11D-1753-08	7.815789474
TCGA-75-6211-01A-11D-1753-08	10.52631579
TCGA-17-Z011-01A-01W-0746-08	3.315789474
TCGA-38-4629-01A-02D-1265-08	8.605263158
TCGA-17-Z031-01A-01W-0746-08	73.57894737
TCGA-62-A46V-01A-11D-A24D-08	1.447368421
TCGA-44-6148-01A-11D-1753-08	0.131578947
TCGA-44-6145-01A-11D-1753-08	8.421052632

Table S2. Univariate analysis and multivariate analysis of the correlation of GNPNAT1 expression with disease-specific survival among lung adenocarcinoma patients in TCGA-LUAD cohort.

Variable	Univariate Cox analysis				Multivariate Cox analysis*			
	coef	HR (95% CI)	z	p-value	coef	HR (95% CI)	z	p-value
Age	-0.0104	0.99 (0.972-1.01)	-1.11	0.268				
Gender (male vs. female)	-0.0445	0.956 (0.658-1.39)	-0.234	0.815				
Stage II (vs. stage I)	1.18	3.25 (2.05-5.16)	5	**5.71E-07**	1.07	2.91 (1.54-5.47)	3.3	**0.00095**
Stage III (vs. stage I)	1.26	3.52 (2.12-5.86)	4.85	**1.24E-06**	1.36	3.89 (1.8-8.42)	3.46	**0.00055**
Stage IV (vs. stage I)	1.59	4.91 (2.48-9.69)	4.58	**4.61E-06**	0.108	1.11 (0.38-3.27)	0.197	0.844
Race (white vs. non-white)	0.0929	1.1 (0.635-1.9)	0.333	0.739				
Ethnicity (Hispanic and Latino vs. non-Hispanic and Latino)	0.667	1.95 (0.612-6.2)	1.13	0.259				
Tobacco smoking history (ever vs. never)	0.0133	1.01 (0.585-1.75)	0.0474	0.962				
Number pack years smoked	0.00231	1 (0.993-1.01)	0.482	0.63				
Radiation therapy (yes vs. no)	0.792	2.21 (1.39-3.51)	3.35	**0.000794**	0.0298	1.03 (0.514-2.06)	0.0842	0.933
Additional radiation therapy (yes vs. no)	-0.0354	0.965 (0.594-1.57)	-0.143	0.886				
Additional pharmaceutical therapy (yes vs. no)	-0.627	0.534 (0.328-0.869)	-2.52	**0.0116**	-0.815	0.443 (0.265-0.74)	-3.11	**0.00187**

KRAS mutation (yes vs. no)	0.254	1.29 (0.408-4.07)	0.433	0.665				
EGFR mutation (yes vs. no)	0.218	1.24 (0.693-2.23)	0.731	0.465				
EML4-ALK translocation (yes vs. no)	0.669	1.95 (0.967-3.94)	1.87	0.0621				
Location in lung parenchyma (central lung vs. peripheral lung)	-0.188	0.828 (0.433-1.59)	-0.568	0.57				
Tumor intermediate dimension	0.0649	1.07 (0.545-2.09)	0.189	0.85				
GNPNAT1	0.534	1.71 (1.32-2.2)	4.11	**3.91E-05**	0.498	1.65 (1.11-2.44)	2.47	**0.0135**

[*]Concordance = 0.703 (se = 0.034), Likelihood ratio test = 33.03 on 6 df, p = 1e-05, Wald test = 32.11 on 6 df, p = 2e-05, Score (logrank) test = 34.58 on 6 df, p = 5e-06.

Table S3. Univariate analysis and multivariate analysis of the correlation of GNPNAT1 expression with progression-free survival among lung adenocarcinoma patients in TCGA-LUAD cohort.

Variable	Univariate Cox analysis				Multivariate Cox analysis*			
	coef	HR (95% CI)	z	p-value	coef	HR (95% CI)	z	p-value
Age	-0.001	0.999 (0.985-1.01)	-0.141	0.888				
Gender (male vs. female)	0.07	1.07 (0.815-1.41)	0.499	0.617				
Stage II (vs. stage I)	0.798	2.22 (1.61-3.07)	4.85	**1.22E-06**	1.2	3.31 (1.92-5.72)	4.3	**1.68E-05**
Stage III (vs. stage I)	0.709	2.03 (1.38-2.99)	3.61	**0.00031**	0.98	2.66 (1.37-5.2)	2.87	**0.00406**
Stage IV (vs. stage I)	0.849	2.34 (1.32-4.13)	2.93	**0.0034**	0.914	2.49 (1.09-5.71)	2.16	**0.0306**
Race (white vs. non-white)	0.0557	1.06 (0.703-1.59)	0.268	0.789				
Ethnicity (Hispanic and Latino vs. non-Hispanic and Latino)	0.291	1.34 (0.495-3.62)	0.574	0.566				
Tobacco smoking history (ever vs. never)	-0.0557	0.946 (0.637-1.4)	-0.276	0.782				
Number pack years smoked	-0.0006	0.999 (0.993-1.01)	-0.165	0.869				
Radiation therapy (yes vs. no)	0.707	2.03 (1.42-2.89)	3.92	**9.01E-05**	0.343	1.41 (0.758-2.62)	1.08	0.278
Additional radiation therapy (yes vs. no)	0.16	1.17 (0.825-1.67)	0.89	0.373				
Additional pharmaceutical therapy (yes vs. no)	0.27	1.31 (0.914-1.88)	1.47	0.141				

KRAS mutation (yes vs. no)	-0.275	0.759 (0.316-1.82)	-0.616	0.538				
EGFR mutation (yes vs. no)	0.483	1.62 (1.06-2.49)	2.21	**0.0271**	0.316	1.37 (0.843-2.23)	1.27	0.204
EML4-ALK translocation (yes vs. no)	0.509	1.66 (0.965-2.87)	1.83	0.0672				
Location in lung parenchyma (central lung vs. peripheral lung)	-0.0709	0.932 (0.584-1.49)	-0.298	0.766				
Tumor intermediate dimension	0.328	1.39 (0.84-2.29)	1.28	0.201				
GNPNAT1	0.327	1.39 (1.15-1.68)	3.36	**0.000777**	0.543	1.72 (1.19-2.49)	2.9	**0.00373**

*Concordance = 0.733 (se = 0.032), Likelihood ratio test = 44.44 on 6 df, p = 6e-08, Wald test = 41.44 on 6 df, p = 2e-07, Score (logrank) test = 46.24 on 6 df, p = 3e-08.

Table S4. *GNPNAT1* co-expressed genes.

Query	Correlation coefficient	P-value	FDR (BH)
A1BG	-0.073329107	0.096453	0.137727
A1CF	0.144260373	0.001027	0.002181
A2BP1	-0.084609426	0.055001	0.08336
A2LD1	0.041511797	0.347131	0.421509
A2ML1	0.03885401	0.378899	0.453661
A2M	-0.37658219	8.50E-19	1.87E-17
A4GALT	0.032846964	0.456994	0.53107
A4GNT	-0.213617454	9.95E-07	3.50E-06
AAA1	0.0789296	0.073512	0.108121
AAAS	0.105652204	0.016461	0.027917
AACSL	-0.108419775	0.013827	0.023836
AACS	0.254661778	4.57E-09	2.29E-08
AADACL2	-0.119822936	0.006481	0.011914
AADACL3	0.075055202	0.088842	0.127984
AADACL4	-0.081349728	0.065082	0.096964
AADAC	-0.180733406	3.70E-05	0.000101
AADAT	-0.219649637	4.80E-07	1.78E-06
AAGAB	0.38305059	1.92E-19	4.65E-18
AAK1	-0.208271628	1.87E-06	6.28E-06
AAMP	0.032396757	0.463189	0.537082
AANAT	-0.146240115	0.000873	0.001875
AARS2	-0.108945876	0.013371	0.023107
AARSD1	0.075881328	0.085375	0.123559
AARS	0.174286369	7.01E-05	0.000183
AASDHPPT	0.248920572	1.03E-08	4.93E-08
AASDH	-0.062306368	0.157982	0.213332
AASS	-0.297932077	5.12E-12	3.99E-11
AATF	0.142709288	0.001165	0.002449
AATK	-0.244684125	1.86E-08	8.53E-08
ABAT	-0.308817125	7.66E-13	6.83E-12
ABCA10	-0.448152503	8.27E-27	5.90E-25
ABCA11P	0.040385444	0.360382	0.43514
ABCA12	-0.011476907	0.794996	0.835808
ABCA13	-0.224060796	2.78E-07	1.07E-06
ABCA17P	-0.130976617	0.002902	0.005681
ABCA1	-0.104195758	0.018017	0.03031
ABCA2	-0.302770488	2.22E-12	1.84E-11
ABCA3	-0.353434414	1.34E-16	2.09E-15
ABCA4	-0.218258385	5.69E-07	2.08E-06
ABCA5	-0.378424609	5.58E-19	1.26E-17
ABCA6	-0.329636443	1.61E-14	1.85E-13
ABCA7	-0.311718544	4.56E-13	4.19E-12

ABCA8	-0.400258463	3.09E-21	9.76E-20
ABCA9	-0.36722352	6.92E-18	1.33E-16
ABCB10	0.167722497	0.000131	0.000328
ABCB11	-0.007996954	0.856335	0.886723
ABCB1	-0.173087057	7.87E-05	0.000203
ABCB4	-0.037307863	0.398174	0.472803
ABCB5	-0.088717469	0.044178	0.068373
ABCB6	0.181744665	3.34E-05	9.18E-05
ABCB7	0.150591667	0.000606	0.00134
ABCB8	-0.00013075	0.997638	0.998519
ABCB9	-0.084612258	0.054993	0.083354
ABCC10	-0.16202216	0.000222	0.000533
ABCC11	-0.021215948	0.630978	0.692776
ABCC12	-0.379898753	3.98E-19	9.20E-18
ABCC13	-0.189231403	1.54E-05	4.49E-05
ABCC1	0.081987051	0.063	0.094205
ABCC2	0.304713091	1.58E-12	1.34E-11
ABCC3	-0.259171719	2.38E-09	1.24E-08
ABCC4	-0.153508839	0.000472	0.001063
ABCC5	-0.172827928	8.07E-05	0.000208
ABCC6P1	-0.260251233	2.03E-09	1.07E-08
ABCC6P2	-0.298004333	5.06E-12	3.95E-11
ABCC6	-0.39529518	1.04E-20	3.02E-19
ABCC8	-0.119272003	0.006732	0.012347
ABCC9	-0.032726101	0.458653	0.532471
ABCD1	-0.004024324	0.927409	0.943218
ABCD2	-0.107896067	0.014295	0.024575
ABCD3	-0.103508185	0.018795	0.031489
ABCD4	-0.220360436	4.40E-07	1.64E-06
ABCE1	0.48134611	3.17E-31	4.40E-29
ABCF1	0.097091126	0.027581	0.044638
ABCF2	0.361152836	2.60E-17	4.55E-16
ABCF3	0.068786914	0.118978	0.165955
ABCG1	-0.124908731	0.004527	0.008563
ABCG2	-0.069646437	0.114426	0.160378
ABCG4	-0.004269237	0.923005	0.939827
ABCG5	0.062658455	0.155641	0.210526
ABCG8	0.145704787	0.000912	0.001953
ABHD10	0.163861064	0.000188	0.000456
ABHD11	-0.012268452	0.781205	0.824083
ABHD12B	-0.084803366	0.054444	0.082642
ABHD12	-0.031989852	0.468828	0.54252
ABHD13	0.019490221	0.659017	0.718157
ABHD14A	-0.202073589	3.79E-06	1.21E-05

ABHD14B	-0.198336895	5.76E-06	1.79E-05
ABHD15	-0.121820816	0.005637	0.010491
ABHD1	-0.09547749	0.030279	0.04857
ABHD2	-0.065203996	0.13949	0.191217
ABHD3	0.148009754	0.000754	0.00164
ABHD4	0.107039312	0.015091	0.025822
ABHD5	0.091679791	0.037537	0.058939
ABHD6	-0.107105639	0.015028	0.025729
ABHD8	-0.106953084	0.015173	0.025935
ABI1	0.184279002	2.58E-05	7.22E-05
ABI2	-0.161440492	0.000234	0.000559
ABI3BP	-0.394686023	1.21E-20	3.45E-19
ABI3	-0.230205147	1.27E-07	5.17E-07
ABL1	-0.016413706	0.710186	0.764292
ABL2	0.096241409	0.028975	0.046664
ABLIM1	-0.07107349	0.107173	0.151197
ABLIM2	-0.134389596	0.002241	0.004478
ABLIM3	-0.020450801	0.643348	0.70396
ABO	-0.251539308	7.13E-09	3.48E-08
ABP1	-0.048954222	0.267467	0.337041
ABRA	-0.191644517	1.19E-05	3.53E-05
ABR	-0.247707242	1.22E-08	5.76E-08
ABT1	0.172827146	8.07E-05	0.000208
ABTB1	-0.452004807	2.69E-27	2.06E-25
ABTB2	0.024875737	0.573274	0.641301
ACAA1	-0.249882045	9.01E-09	4.34E-08
ACAA2	0.003969981	0.928387	0.943828
ACACA	-0.011697302	0.79115	0.832465
ACACB	-0.393878286	1.47E-20	4.12E-19
ACAD10	-0.134000067	0.002309	0.004603
ACAD11	-0.240772434	3.16E-08	1.40E-07
ACAD8	-0.354692719	1.03E-16	1.63E-15
ACAD9	0.025332297	0.566254	0.634967
ACADL	-0.324294912	4.47E-14	4.78E-13
ACADM	0.074474306	0.091348	0.131197
ACADSB	-0.239768777	3.62E-08	1.59E-07
ACADS	-0.250112418	8.72E-09	4.21E-08
ACADVL	-0.175027895	6.52E-05	0.000171
ACAN	0.128278959	0.003544	0.006827
ACAP1	-0.257927452	2.85E-09	1.47E-08
ACAP2	0.012758171	0.772706	0.817274
ACAP3	-0.213424674	1.02E-06	3.57E-06
ACAT1	0.074699201	0.090371	0.129934
ACAT2	0.180910328	3.63E-05	9.93E-05

ACBD3	0.163576608	0.000193	0.000468
ACBD4	-0.322184116	6.65E-14	6.92E-13
ACBD5	0.219527938	4.87E-07	1.80E-06
ACBD6	0.177731791	4.99E-05	0.000133
ACBD7	0.019358349	0.66118	0.720161
ACCN1	-0.090377873	0.040344	0.062965
ACCN2	0.030795183	0.485602	0.559136
ACCN3	-0.203278805	3.31E-06	1.07E-05
ACCN4	-0.203745732	3.14E-06	1.02E-05
ACCN5	-0.027082409	0.539733	0.610606
ACCSL	-0.11578899	0.008535	0.015332
ACCS	-0.402578041	1.74E-21	5.75E-20
ACD	-0.089210789	0.043008	0.066706
ACE2	-0.169715311	0.000109	0.000275
ACER1	-0.18264772	3.05E-05	8.43E-05
ACER2	-0.138596633	0.001617	0.00331
ACER3	0.06286921	0.154252	0.208874
ACE	-0.230370034	1.25E-07	5.07E-07
ACHE	-0.246148823	1.52E-08	7.06E-08
ACIN1	-0.141493655	0.001285	0.00268
ACLY	0.308576765	8.00E-13	7.12E-12
ACMSD	0.128125309	0.003585	0.006897
ACN9	0.321247605	7.93E-14	8.14E-13
ACO1	0.044295247	0.315731	0.388096
ACO2	-0.064670089	0.142767	0.195239
ACOT11	-0.040080359	0.364025	0.438691
ACOT12	0.128346149	0.003527	0.006798
ACOT13	0.17574442	6.08E-05	0.00016
ACOT1	-0.019207874	0.663652	0.722223
ACOT2	0.053160269	0.228466	0.294182
ACOT4	0.132613814	0.002566	0.005072
ACOT6	0.158604845	0.000302	0.000705
ACOT7	0.252171588	6.52E-09	3.20E-08
ACOT8	0.010785541	0.807095	0.845726
ACOT9	0.204250921	2.96E-06	9.62E-06
ACOX1	-0.076873121	0.081356	0.118352
ACOX2	-0.143456624	0.001096	0.002316
ACOX3	-0.170430914	0.000102	0.000258
ACOXL	-0.490071228	1.81E-32	3.12E-30
ACP1	0.298194533	4.90E-12	3.82E-11
ACP2	-0.032264648	0.465016	0.538857
ACP5	-0.248342125	1.12E-08	5.30E-08
ACP6	-0.008303865	0.850884	0.882138
ACPL2	0.077031228	0.08073	0.117603

ACPP	-0.031801924	0.471446	0.545201
ACPT	0.080708361	0.067235	0.099858
ACRBP	-0.116253505	0.008272	0.014914
ACRC	-0.122171587	0.0055	0.010257
ACRV1	0.260834841	1.86E-09	9.90E-09
ACR	-0.229371733	1.42E-07	5.70E-07
ACSBG1	-0.272428632	3.24E-10	1.93E-09
ACSBG2	-0.117820136	0.007437	0.013529
ACSF2	-0.189215857	1.54E-05	4.49E-05
ACSF3	-0.172509499	8.32E-05	0.000214
ACSL1	-0.147488173	0.000787	0.001705
ACSL3	0.223810216	2.87E-07	1.10E-06
ACSL4	0.165937156	0.000155	0.000382
ACSL5	-0.204149322	3.00E-06	9.72E-06
ACSL6	-0.234725914	7.07E-08	2.98E-07
ACSM1	-0.387605024	6.58E-20	1.68E-18
ACSM2A	-0.101526761	0.021202	0.035166
ACSM2B	-0.047043923	0.286608	0.357576
ACSM3	-0.242326608	2.56E-08	1.15E-07
ACSM4	-0.001521893	0.972516	0.979425
ACSM5	-0.361582231	2.37E-17	4.16E-16
ACSS1	-0.488674517	2.88E-32	4.79E-30
ACSS2	-0.291618012	1.49E-11	1.09E-10
ACSS3	-0.095468355	0.030295	0.048591
ACTA1	-0.034565683	0.433775	0.508403
ACTA2	-0.03949585	0.371068	0.445915
ACTBL2	-0.044729597	0.311004	0.38304
ACTB	0.078808126	0.073958	0.108717
ACTC1	-0.142939018	0.001143	0.002407
ACTG1	0.236380616	5.69E-08	2.42E-07
ACTG2	-0.138232692	0.001664	0.0034
ACTL6A	0.367031619	7.22E-18	1.38E-16
ACTL6B	0.030618287	0.488113	0.561422
ACTL7A	-0.057476383	0.19283	0.253838
ACTL7B	-0.119062561	0.00683	0.012513
ACTL8	0.268877242	5.59E-10	3.23E-09
ACTL9	0.039846428	0.366834	0.441517
ACTN1	0.158149846	0.000315	0.000732
ACTN2	-0.378004349	6.14E-19	1.38E-17
ACTN3	0.029252637	0.50773	0.580312
ACTN4	-0.052034193	0.238489	0.305258
ACTR10	0.248072044	1.16E-08	5.49E-08
ACTR1A	0.101906342	0.020721	0.034428
ACTR1B	-0.165573731	0.00016	0.000395

ACTR2	0.227041899	1.91E-07	7.52E-07
ACTR3B	0.220660675	4.24E-07	1.58E-06
ACTR3C	0.031621467	0.473966	0.547702
ACTR3	0.392775832	1.92E-20	5.30E-19
ACTR5	0.062969168	0.153597	0.208099
ACTR6	0.301313711	2.86E-12	2.32E-11
ACTR8	-0.01703178	0.699792	0.755343
ACTRT1	-0.005800261	0.89553	0.918224
ACTRT2	-0.005211115	0.906089	0.926627
ACVR1B	-0.099655356	0.023717	0.038886
ACVR1C	0.025100115	0.569819	0.638034
ACVR1	-0.099871579	0.023414	0.038451
ACVR2A	-0.216819653	6.78E-07	2.44E-06
ACVR2B	-0.023260973	0.598427	0.663954
ACVRL1	-0.223437793	3.00E-07	1.15E-06
ACY1	0.131822806	0.002723	0.005359
ACY3	0.031888974	0.470232	0.543987
ACYP1	0.123849578	0.004884	0.009193
ACYP2	-0.149131392	0.000686	0.001504
ADAD1	0.045495146	0.302788	0.374466
ADAD2	-0.059966709	0.174217	0.232569
ADAL	-0.11176336	0.011145	0.019588
ADAM10	0.185581889	2.25E-05	6.37E-05
ADAM11	-0.12315712	0.00513	0.009622
ADAM12	0.244779621	1.83E-08	8.42E-08
ADAM15	-0.018971606	0.66754	0.725892
ADAM17	0.232895751	8.99E-08	3.73E-07
ADAM18	0.063110061	0.152677	0.206965
ADAM19	0.05601238	0.20443	0.267156
ADAM20	-0.162484052	0.000213	0.000513
ADAM21P1	0.08364655	0.057834	0.087172
ADAM21	0.136764272	0.001866	0.00378
ADAM22	0.053654126	0.224167	0.289298
ADAM23	-0.061923931	0.160554	0.216396
ADAM28	-0.161275645	0.000238	0.000567
ADAM29	-0.106730573	0.015387	0.026254
ADAM2	0.142965497	0.001141	0.002403
ADAM30	-0.047541468	0.281536	0.352171
ADAM32	0.152297943	0.000524	0.001171
ADAM33	-0.330974409	1.25E-14	1.45E-13
ADAM3A	0.017344072	0.694561	0.750508
ADAM5P	0.040462705	0.359463	0.434162
ADAM6	-0.070582711	0.109625	0.154331
ADAM7	0.03443659	0.435495	0.51006

ADAM8	-0.169243817	0.000114	0.000287
ADAM9	0.164477802	0.000177	0.000434
ADAMDEC1	0.061013729	0.166804	0.223764
ADAMTS10	-0.286056063	3.73E-11	2.55E-10
ADAMTS12	0.174655037	6.76E-05	0.000177
ADAMTS13	-0.313386499	3.37E-13	3.17E-12
ADAMTS14	-0.09201664	0.036839	0.057943
ADAMTS15	-0.107856888	0.014331	0.024626
ADAMTS16	0.003501981	0.93681	0.950313
ADAMTS17	-0.42153638	1.33E-23	6.01E-22
ADAMTS18	0.022529521	0.609982	0.674317
ADAMTS19	-0.002080556	0.962433	0.97113
ADAMTS1	-0.203340045	3.29E-06	1.06E-05
ADAMTS20	0.183915396	2.68E-05	7.48E-05
ADAMTS2	0.031086811	0.481478	0.555293
ADAMTS3	-0.01560584	0.723853	0.775868
ADAMTS4	0.240635416	3.22E-08	1.43E-07
ADAMTS5	0.277299864	1.52E-10	9.50E-10
ADAMTS6	0.159034462	0.000291	0.000681
ADAMTS7	0.003600649	0.935034	0.948992
ADAMTS8	-0.487566735	4.15E-32	6.75E-30
ADAMTS9	-0.052554376	0.233821	0.300187
ADAMTSL1	-0.056062067	0.204028	0.266648
ADAMTSL2	-0.374912559	1.24E-18	2.66E-17
ADAMTSL3	-0.29680661	6.21E-12	4.79E-11
ADAMTSL4	-0.237406913	4.96E-08	2.13E-07
ADAMTSL5	-0.078075081	0.076693	0.112394
ADAP1	-0.039829156	0.367041	0.441661
ADAP2	-0.092966388	0.034929	0.055233
ADARB1	-0.293406421	1.10E-11	8.19E-11
ADARB2	-0.142367815	0.001197	0.002511
ADAR	0.066958417	0.129131	0.17841
ADAT1	0.048199977	0.274917	0.345243
ADAT2	-0.146690075	0.000841	0.001812
ADAT3	-0.051551416	0.242879	0.310202
ADA	0.277652801	1.43E-10	9.03E-10
ADCK1	-0.055815005	0.206032	0.268968
ADCK2	-0.039709396	0.368485	0.443184
ADCK4	-0.168393	0.000123	0.000309
ADCK5	-0.053311612	0.227143	0.292606
ADCY10	-0.096388932	0.028728	0.046308
ADCY1	-0.1533667	0.000478	0.001076
ADCY2	-0.23471075	7.09E-08	2.98E-07
ADCY3	0.039971617	0.365329	0.439997

ADCY4	-0.300678739	3.20E-12	2.57E-11
ADCY5	-0.157843373	0.000323	0.00075
ADCY6	-0.304315519	1.70E-12	1.43E-11
ADCY7	-0.137142764	0.001812	0.00368
ADCY8	-0.094008625	0.03293	0.052346
ADCY9	-0.436238807	2.44E-25	1.43E-23
ADCYAP1R1	-0.13500915	0.002137	0.004292
ADCYAP1	-0.185701343	2.23E-05	6.30E-05
ADC	-0.291823614	1.44E-11	1.05E-10
ADD1	-0.256334756	3.59E-09	1.82E-08
ADD2	-0.129692641	0.003193	0.006204
ADD3	-0.130444481	0.00302	0.005894
ADH1A	-0.366597437	7.94E-18	1.51E-16
ADH1B	-0.404450351	1.09E-21	3.69E-20
ADH1C	-0.185810872	2.20E-05	6.24E-05
ADH4	0.083238187	0.059071	0.088869
ADH5	0.110760948	0.011896	0.020762
ADH6	-0.060668633	0.169221	0.226727
ADH7	-0.103843449	0.018412	0.030907
ADHFE1	-0.411565723	1.79E-22	6.71E-21
ADI1	-0.024718331	0.575704	0.643362
ADIG	-0.040440022	0.359733	0.434435
ADIPOQ	0.087309916	0.047665	0.073191
ADIPOR1	-0.022919105	0.603815	0.668831
ADIPOR2	0.305458406	1.39E-12	1.19E-11
ADK	0.054018446	0.221032	0.285787
ADM2	-0.011247451	0.799006	0.839186
ADM	0.24038981	3.33E-08	1.47E-07
ADNP2	0.132317658	0.002624	0.005178
ADNP	0.141329677	0.001302	0.002713
ADORA1	-0.17124171	9.40E-05	0.00024
ADORA2A	-0.288083675	2.67E-11	1.87E-10
ADORA2B	-0.035982043	0.415163	0.489891
ADORA3	-0.142792712	0.001157	0.002433
ADO	0.213896757	9.63E-07	3.39E-06
ADPGK	-0.027359821	0.535587	0.606705
ADPRHL1	-0.045907326	0.298424	0.369801
ADPRHL2	-0.111417913	0.011399	0.020001
ADPRH	-0.28176614	7.46E-11	4.89E-10
ADRA1A	-0.345908426	6.39E-16	9.08E-15
ADRA1B	-0.129145346	0.003325	0.006443
ADRA1D	-0.212690728	1.11E-06	3.87E-06
ADRA2A	-0.216109304	7.38E-07	2.65E-06
ADRA2B	-0.19757103	6.26E-06	1.93E-05

ADRA2C	-0.161414358	0.000235	0.00056
ADRB1	-0.34734693	4.76E-16	6.92E-15
ADRB2	-0.411283464	1.93E-22	7.17E-21
ADRB3	-0.202101626	3.78E-06	1.21E-05
ADRBK1	-0.103564698	0.01873	0.031401
ADRBK2	-0.186022844	2.15E-05	6.12E-05
ADRM1	0.133229393	0.002449	0.004859
ADSL	0.317445349	1.60E-13	1.58E-12
ADSSL1	0.136969237	0.001837	0.003726
ADSS	0.128950197	0.003373	0.006524
AEBP1	-0.126281087	0.004101	0.007816
AEBP2	0.141997376	0.001234	0.002582
AEN	0.217390744	6.32E-07	2.29E-06
AES	-0.224676781	2.57E-07	9.93E-07
AFAP1L1	-0.050634919	0.251368	0.319493
AFAP1L2	-0.042240814	0.338722	0.412827
AFAP1	-0.014770092	0.738085	0.788121
AFARP1	0.180304097	3.86E-05	0.000105
AFF1	-0.219399631	4.95E-07	1.83E-06
AFF2	-0.237084974	5.18E-08	2.22E-07
AFF3	-0.355351525	8.97E-17	1.43E-15
AFF4	-0.040339605	0.360928	0.435668
AFG3L1	-0.193719556	9.53E-06	2.86E-05
AFG3L2	0.104794467	0.017363	0.029274
AFMID	0.095347842	0.030506	0.048886
AFM	0.111051611	0.011674	0.02042
AFP	0.198835246	5.45E-06	1.70E-05
AFTPH	-0.051241441	0.245727	0.313379
AG2	-0.265416463	9.43E-10	5.27E-09
AGAP11	-0.376729785	8.22E-19	1.81E-17
AGAP1	0.019030613	0.666568	0.725081
AGAP2	-0.20490144	2.75E-06	8.98E-06
AGAP3	-0.234929511	6.89E-08	2.90E-07
AGAP4	-0.244263829	1.97E-08	9.01E-08
AGAP5	-0.341667177	1.51E-15	2.04E-14
AGAP6	-0.216006935	7.47E-07	2.67E-06
AGAP7	-0.154011315	0.000452	0.001022
AGAP8	-0.229273334	1.44E-07	5.76E-07
AGA	0.046840112	0.288703	0.359741
AGBL1	-0.15916307	0.000287	0.000674
AGBL2	-0.302343747	2.40E-12	1.96E-11
AGBL3	-0.172950134	7.98E-05	0.000206
AGBL4	-0.118955939	0.00688	0.012597
AGBL5	0.070228048	0.111425	0.156567

AGER	-0.357018304	6.30E-17	1.04E-15
AGFG1	0.331389283	1.15E-14	1.34E-13
AGFG2	-0.215722765	7.73E-07	2.76E-06
AGGF1	-0.142881386	0.001149	0.002417
AGK	0.013002379	0.768478	0.813836
AGL	-0.005427042	0.902217	0.923517
AGMAT	0.426000087	4.04E-24	1.98E-22
AGPAT1	-0.036917684	0.40313	0.477808
AGPAT2	-0.100216879	0.022937	0.037758
AGPAT3	-0.262180261	1.53E-09	8.25E-09
AGPAT4	-0.147965109	0.000756	0.001645
AGPAT5	0.277396844	1.49E-10	9.38E-10
AGPAT6	0.037340418	0.397762	0.472511
AGPAT9	0.102579205	0.019892	0.033164
AGPHD1	0.006646875	0.880389	0.906232
AGPS	0.33328423	7.96E-15	9.52E-14
AGR2	0.034786717	0.430839	0.505554
AGR3	-0.236194826	5.83E-08	2.48E-07
AGRN	-0.138420575	0.001639	0.003353
AGRP	-0.226438525	2.06E-07	8.08E-07
AGTPBP1	-0.061681644	0.1622	0.218277
AGTR1	-0.157071138	0.000346	0.000799
AGTR2	-0.167907544	0.000129	0.000323
AGTRAP	0.021312021	0.629432	0.691653
AGT	0.063375452	0.150955	0.204992
AGXT2L1	0.233807283	7.98E-08	3.33E-07
AGXT2L2	-0.435116481	3.34E-25	1.93E-23
AGXT2	-0.089249773	0.042917	0.066585
AGXT	0.031682503	0.473113	0.546844
AHCTF1	0.219105107	5.13E-07	1.89E-06
AHCYL1	-0.127875735	0.003651	0.007013
AHCYL2	-0.138990513	0.001567	0.003217
AHCY	0.411207135	1.97E-22	7.30E-21
AHDC1	-0.320649158	8.87E-14	9.04E-13
AHI1	-0.141116591	0.001324	0.002754
AHNAK2	0.151451899	0.000564	0.001252
AHNAK	-0.222319864	3.45E-07	1.31E-06
AHRR	-0.032672471	0.45939	0.533264
AHR	-0.179106884	4.35E-05	0.000117
AHSA1	0.437852165	1.56E-25	9.46E-24
AHSA2	-0.321224387	7.96E-14	8.17E-13
AHSG	0.201196233	4.19E-06	1.33E-05
AHSP	0.002559582	0.953792	0.964407
AICDA	-0.008307997	0.850811	0.882138

AIDA	-0.011824575	0.788931	0.830654
AIF1L	-0.119058817	0.006832	0.012515
AIF1	-0.149479899	0.000666	0.001464
AIFM1	0.052783741	0.231784	0.297859
AIFM2	0.166011776	0.000154	0.00038
AIFM3	-0.153888566	0.000457	0.001032
AIG1	0.134317311	0.002254	0.004501
AIM1L	0.081529243	0.06449	0.096146
AIM1	-0.154205006	0.000445	0.001007
AIM2	0.078300465	0.075844	0.111312
AIMP1	0.317516517	1.58E-13	1.56E-12
AIMP2	0.455678426	9.08E-28	7.32E-26
AIPL1	0.004156848	0.925026	0.941512
AIP	0.021154908	0.631961	0.693703
AIRE	-0.156700539	0.000358	0.000823
AJAP1	-0.199261148	5.20E-06	1.63E-05
AK1	-0.336637055	4.12E-15	5.19E-14
AK2	0.028710073	0.515637	0.58787
AK3L1	0.30655824	1.14E-12	9.93E-12
AK3	0.00599153	0.892106	0.91559
AK5	0.041715785	0.344765	0.418966
AK7	-0.071027737	0.107399	0.151475
AKAP10	-0.033870525	0.443083	0.51728
AKAP11	-0.087897065	0.046183	0.071184
AKAP12	0.197624511	6.23E-06	1.92E-05
AKAP13	-0.399207851	4.01E-21	1.25E-19
AKAP14	-0.192628028	1.07E-05	3.20E-05
AKAP1	-0.241220091	2.97E-08	1.32E-07
AKAP2	-0.009451045	0.830577	0.86513
AKAP3	-0.206977747	2.17E-06	7.20E-06
AKAP4	-0.022927355	0.603685	0.668724
AKAP5	0.037309597	0.398152	0.472803
AKAP6	-0.156847706	0.000353	0.000813
AKAP7	-0.295136062	8.24E-12	6.26E-11
AKAP8L	-0.208333032	1.85E-06	6.24E-06
AKAP8	-0.17093905	9.68E-05	0.000247
AKAP9	-0.156482735	0.000365	0.000837
AKD1	-0.258680201	2.56E-09	1.33E-08
AKIRIN1	0.181906252	3.28E-05	9.04E-05
AKIRIN2	0.136916667	0.001844	0.00374
AKNAD1	0.097204565	0.027399	0.044355
AKNA	-0.37643851	8.78E-19	1.93E-17
AKR1A1	-0.049361578	0.263502	0.332673
AKR1B10	0.216549027	7.00E-07	2.52E-06

AKR1B15	0.26761973	6.77E-10	3.86E-09
AKR1B1	0.084683117	0.054789	0.083083
AKR1C1	0.155625909	0.000393	0.000897
AKR1C2	0.211056574	1.35E-06	4.63E-06
AKR1C3	0.156902874	0.000351	0.000809
AKR1C4	0.251248954	7.43E-09	3.63E-08
AKR1CL1	0.130162212	0.003084	0.006009
AKR1D1	0.139322842	0.001527	0.003143
AKR1E2	0.138429136	0.001638	0.003351
AKR7A2	-0.063559614	0.149769	0.203565
AKR7A3	0.183537467	2.78E-05	7.75E-05
AKR7L	0.063195236	0.152122	0.206284
AKT1S1	0.089809244	0.041624	0.064791
AKT1	-0.104909957	0.017239	0.029087
AKT2	-0.010273438	0.816087	0.853314
AKT3	0.027795768	0.529103	0.601057
AKTIP	-0.141470906	0.001287	0.002685
ALAD	-0.285725935	3.93E-11	2.69E-10
ALAS1	-0.081488113	0.064625	0.096326
ALAS2	-0.052864193	0.231072	0.297145
ALB	-0.101437795	0.021316	0.035341
ALCAM	-0.131991556	0.002689	0.0053
ALDH16A1	-0.197355619	6.41E-06	1.98E-05
ALDH18A1	0.342297257	1.33E-15	1.81E-14
ALDH1A1	-0.170481505	0.000101	0.000257
ALDH1A2	-0.11378788	0.009755	0.017362
ALDH1A3	-0.018125022	0.681544	0.738639
ALDH1B1	0.299829063	3.70E-12	2.94E-11
ALDH1L1	-0.090185682	0.040772	0.063584
ALDH1L2	0.242424059	2.53E-08	1.14E-07
ALDH2	-0.286883324	3.25E-11	2.25E-10
ALDH3A1	-0.076884911	0.08131	0.118301
ALDH3A2	-0.102300597	0.020232	0.033671
ALDH3B1	-0.132686735	0.002551	0.005048
ALDH3B2	0.10463174	0.017538	0.029558
ALDH4A1	-0.19791902	6.03E-06	1.87E-05
ALDH5A1	-0.231803264	1.04E-07	4.25E-07
ALDH6A1	-0.071374249	0.105691	0.149297
ALDH7A1	-0.022898713	0.604138	0.669114
ALDH8A1	-0.16364382	0.000192	0.000465
ALDH9A1	-0.103255269	0.019088	0.031943
ALDOA	0.255981081	3.78E-09	1.91E-08
ALDOB	-0.217833618	5.99E-07	2.18E-06
ALDOC	-0.007812466	0.859614	0.88952

ALG10B	0.056678792	0.199088	0.260943
ALG10	0.323322818	5.37E-14	5.65E-13
ALG11	0.064150442	0.146011	0.199107
ALG12	-0.208258277	1.87E-06	6.28E-06
ALG13	-0.111940152	0.011017	0.01938
ALG14	0.212923336	1.08E-06	3.77E-06
ALG1L2	0.111990341	0.010981	0.01932
ALG1L	0.106342237	0.015767	0.026843
ALG1	-0.122072451	0.005539	0.010325
ALG2	0.072183644	0.101785	0.144446
ALG3	0.24473049	1.84E-08	8.48E-08
ALG5	0.068117334	0.122621	0.170576
ALG6	0.157567818	0.000331	0.000766
ALG8	0.336197654	4.49E-15	5.63E-14
ALG9	0.070249346	0.111316	0.156436
ALKBH1	0.185699346	2.23E-05	6.30E-05
ALKBH2	0.332260286	9.72E-15	1.15E-13
ALKBH3	-0.104958342	0.017187	0.029017
ALKBH4	0.072262203	0.101412	0.144026
ALKBH5	-0.046497146	0.292252	0.363506
ALKBH6	-0.121762992	0.00566	0.010529
ALKBH7	-0.078397621	0.07548	0.110811
ALKBH8	-0.043199953	0.327858	0.401177
ALK	-0.17952504	4.18E-05	0.000113
ALLC	0.024216767	0.583478	0.650305
ALMS1P	-0.191708786	1.18E-05	3.51E-05
ALMS1	-0.13556379	0.002048	0.004125
ALOX12B	-0.008449793	0.848295	0.880177
ALOX12P2	0.003286172	0.940697	0.953239
ALOX12	-0.097834762	0.026408	0.042911
ALOX15B	-0.30513792	1.47E-12	1.25E-11
ALOX15	-0.245197528	1.73E-08	7.98E-08
ALOX5AP	-0.169673554	0.000109	0.000276
ALOX5	-0.283735808	5.43E-11	3.63E-10
ALOXE3	0.157652103	0.000329	0.000762
ALPI	0.096813467	0.02803	0.045295
ALPK1	-0.283041767	6.08E-11	4.03E-10
ALPK2	0.119038981	0.006841	0.01253
ALPK3	-0.015187075	0.730973	0.782073
ALPL	-0.333294744	7.94E-15	9.51E-14
ALPPL2	-0.051149746	0.246574	0.314299
ALPP	-0.079363659	0.071938	0.10611
ALS2CL	-0.2980374	5.03E-12	3.93E-11
ALS2CR11	-0.099821327	0.023484	0.038556

ALS2CR12	-0.300435143	3.33E-12	2.67E-11
ALS2CR4	0.094299875	0.032389	0.051577
ALS2CR8	-0.423901764	7.09E-24	3.31E-22
ALS2	-0.01319134	0.765211	0.811021
ALX1	0.133024459	0.002487	0.004928
ALX3	-0.093678533	0.033552	0.053246
ALX4	-0.0654649	0.137911	0.189337
AMAC1L2	-0.109153648	0.013194	0.022832
AMAC1L3	-0.17633949	5.73E-05	0.000151
AMAC1	-0.07274032	0.099165	0.141226
AMACR	0.040831677	0.355095	0.429643
AMBN	0.095281679	0.030622	0.049052
AMBP	0.035457679	0.421998	0.49657
AMBRA1	-0.230277652	1.26E-07	5.12E-07
AMD1	0.240865041	3.12E-08	1.39E-07
AMDHD1	-0.014568201	0.741537	0.790835
AMDHD2	-0.175657738	6.13E-05	0.000161
AMELX	0.066211069	0.133468	0.183882
AMELY	0.113124321	0.010193	0.018067
AMFR	0.116599165	0.008081	0.014607
AMHR2	-0.108925253	0.013389	0.02313
AMH	-0.111533836	0.011314	0.019864
AMICA1	-0.362940234	1.77E-17	3.16E-16
AMIGO1	-0.351433502	2.04E-16	3.10E-15
AMIGO2	-5.49E-05	0.999008	0.999408
AMIGO3	-0.138847815	0.001585	0.00325
AMMECR1L	0.047501505	0.281941	0.352612
AMMECR1	0.187559379	1.83E-05	5.28E-05
AMN1	0.131870173	0.002714	0.005345
AMN	-0.097841342	0.026398	0.042901
AMOTL1	-0.182149058	3.20E-05	8.83E-05
AMOTL2	-0.200447999	4.55E-06	1.44E-05
AMOT	-0.311802167	4.49E-13	4.14E-12
AMPD1	-0.207999078	1.93E-06	6.46E-06
AMPD2	-0.137353493	0.001782	0.003623
AMPD3	-0.212161574	1.18E-06	4.10E-06
AMPH	0.017169153	0.697489	0.753224
AMTN	-0.107281869	0.014862	0.025477
AMT	-0.371749127	2.53E-18	5.15E-17
AMY1A	-0.355805234	8.15E-17	1.32E-15
AMY2A	-0.35582539	8.12E-17	1.32E-15
AMY2B	-0.481679015	2.85E-31	4.01E-29
AMZ1	-0.004421844	0.920262	0.93757
AMZ2P1	-0.203605885	3.19E-06	1.03E-05

AMZ2	0.112548459	0.010587	0.018698
ANAPC10	0.19221932	1.12E-05	3.33E-05
ANAPC11	0.172274712	8.51E-05	0.000219
ANAPC13	0.059434451	0.178078	0.237106
ANAPC16	-0.056279604	0.202276	0.264808
ANAPC1	0.232717696	9.20E-08	3.81E-07
ANAPC2	-0.266705016	7.77E-10	4.39E-09
ANAPC4	-0.096343487	0.028804	0.046412
ANAPC5	0.30355775	1.94E-12	1.62E-11
ANAPC7	0.363973118	1.41E-17	2.57E-16
ANGEL1	-0.004031259	0.927284	0.943139
ANGEL2	-0.132150473	0.002657	0.005241
ANGPT1	-0.242975009	2.34E-08	1.06E-07
ANGPT2	0.240663577	3.21E-08	1.42E-07
ANGPT4	-0.239673003	3.66E-08	1.61E-07
ANGPTL1	-0.153770297	0.000462	0.001042
ANGPTL2	0.064053537	0.146623	0.199761
ANGPTL3	0.100728023	0.022246	0.03673
ANGPTL4	0.249176855	9.95E-09	4.77E-08
ANGPTL5	-0.35418913	1.15E-16	1.81E-15
ANGPTL6	-0.325011432	3.91E-14	4.23E-13
ANGPTL7	-0.301169224	2.94E-12	2.37E-11
ANG	-0.078161532	0.076366	0.11198
ANK1	-0.011148929	0.80073	0.840422
ANK2	-0.177154752	5.29E-05	0.00014
ANK3	-0.291789772	1.45E-11	1.06E-10
ANKAR	-0.259607349	2.23E-09	1.17E-08
ANKDD1A	-0.263866533	1.19E-09	6.53E-09
ANKFN1	-0.324702361	4.14E-14	4.46E-13
ANKFY1	-0.254996987	4.36E-09	2.19E-08
ANKHD1-EIF4EBP3	-0.286032402	3.74E-11	2.56E-10
ANKHD1	-0.281610941	7.65E-11	5.01E-10
ANKH	-0.109215839	0.013142	0.022759
ANKIB1	0.279126066	1.14E-10	7.25E-10
ANKK1	-0.381349416	2.85E-19	6.76E-18
ANKLE1	0.128321579	0.003533	0.006808
ANKLE2	0.221142918	3.99E-07	1.50E-06
ANKMY1	-0.395282377	1.05E-20	3.02E-19
ANKMY2	-0.15111329	0.00058	0.001286
ANKRA2	-0.133367604	0.002423	0.004812
ANKRD10	-0.294485314	9.20E-12	6.93E-11
ANKRD11	-0.186022713	2.15E-05	6.12E-05
ANKRD12	-0.184075553	2.63E-05	7.37E-05
ANKRD13A	-0.225920448	2.20E-07	8.57E-07

ANKRD13B	-0.009953276	0.821721	0.857858
ANKRD13C	-0.082911747	0.060076	0.090245
ANKRD13D	-0.136529427	0.0019	0.003844
ANKRD16	0.032215369	0.465698	0.539491
ANKRD17	0.025731688	0.560147	0.629497
ANKRD19	0.06161419	0.162661	0.218852
ANKRD1	-0.092133971	0.036598	0.0576
ANKRD20A3	-0.186163965	2.12E-05	6.04E-05
ANKRD20A4	-0.147938268	0.000758	0.001648
ANKRD20B	-0.172954158	7.97E-05	0.000206
ANKRD22	-0.063064443	0.152974	0.20734
ANKRD23	-0.243438072	2.20E-08	9.99E-08
ANKRD24	-0.406345552	6.77E-22	2.36E-20
ANKRD26P1	0.018157164	0.681011	0.73814
ANKRD26	-0.011924338	0.787192	0.829086
ANKRD27	0.053807932	0.222839	0.287789
ANKRD28	-0.005631508	0.898553	0.920662
ANKRD29	-0.156314117	0.00037	0.000848
ANKRD2	0.073080903	0.097589	0.1392
ANKRD30A	0.0092173	0.834706	0.868646
ANKRD30B	0.11238499	0.010701	0.018877
ANKRD31	-0.001060691	0.980843	0.985775
ANKRD32	0.22883525	1.52E-07	6.07E-07
ANKRD33	0.074637133	0.09064	0.130264
ANKRD34A	-0.150659049	0.000603	0.001334
ANKRD34B	-0.096067137	0.029268	0.047095
ANKRD34C	-0.163969971	0.000186	0.000452
ANKRD35	-0.130784395	0.002944	0.005758
ANKRD36BP1	-0.066277013	0.133081	0.183389
ANKRD36B	-0.094622527	0.031799	0.05074
ANKRD36	-0.21047292	1.44E-06	4.93E-06
ANKRD37	-0.014509404	0.742543	0.791732
ANKRD39	0.112220148	0.010817	0.019068
ANKRD40	-0.047085504	0.286182	0.357199
ANKRD42	-0.185881706	2.18E-05	6.20E-05
ANKRD43	-0.033544124	0.447493	0.521757
ANKRD44	-0.417098012	4.28E-23	1.78E-21
ANKRD45	-0.150158957	0.000629	0.001386
ANKRD46	0.076533294	0.082716	0.120119
ANKRD49	0.026639822	0.546381	0.61687
ANKRD50	-0.164494141	0.000177	0.000433
ANKRD52	0.087650576	0.0468	0.072029
ANKRD53	-0.11950174	0.006626	0.012166
ANKRD54	-0.058892173	0.182077	0.241465

ANKRD55	-0.282988418	6.13E-11	4.06E-10
ANKRD56	-0.041222699	0.350502	0.425163
ANKRD57	0.206909192	2.19E-06	7.25E-06
ANKRD58	-0.25778081	2.91E-09	1.50E-08
ANKRD5	0.01407778	0.749943	0.798182
ANKRD6	-0.369021531	4.65E-18	9.14E-17
ANKRD7	0.142176804	0.001216	0.002547
ANKRD9	0.023339966	0.597185	0.662829
ANKS1A	-0.091023609	0.03893	0.060925
ANKS1B	-0.240033877	3.49E-08	1.54E-07
ANKS3	-0.329564425	1.64E-14	1.88E-13
ANKS4B	0.092198123	0.036467	0.057421
ANKS6	-0.218371111	5.61E-07	2.06E-06
ANKZF1	-0.165326126	0.000164	0.000403
ANLN	0.510763809	1.45E-35	4.68E-33
ANO10	0.193544444	9.71E-06	2.91E-05
ANO1	-0.090617299	0.039815	0.062192
ANO2	-0.290202012	1.88E-11	1.35E-10
ANO3	0.120090732	0.006361	0.011714
ANO4	-0.106635555	0.015479	0.026397
ANO5	-0.284857068	4.53E-11	3.07E-10
ANO6	0.06355927	0.149771	0.203565
ANO7	0.178014907	4.86E-05	0.00013
ANO8	-0.19989067	4.84E-06	1.52E-05
ANO9	-0.276981832	1.59E-10	9.95E-10
ANP32A	0.146875476	0.000828	0.001787
ANP32B	0.279452345	1.08E-10	6.90E-10
ANP32C	0.134630062	0.0022	0.004405
ANP32D	0.012641051	0.774736	0.819
ANP32E	0.245592775	1.64E-08	7.58E-08
ANPEP	-0.118304742	0.007195	0.01312
ANTXR1	0.017738245	0.68798	0.744644
ANTXR2	-0.003345673	0.939625	0.952395
ANTXRL	-0.230073696	1.30E-07	5.25E-07
ANUBL1	-0.089562938	0.042189	0.065558
ANXA10	0.070701819	0.109026	0.153574
ANXA11	-0.169345078	0.000113	0.000284
ANXA13	0.107285149	0.014859	0.025473
ANXA1	-0.062873306	0.154225	0.208852
ANXA2P1	0.135265189	0.002096	0.004213
ANXA2P2	0.085719916	0.051878	0.079077
ANXA2P3	0.113133951	0.010186	0.018057
ANXA2	0.078182626	0.076287	0.111897
ANXA3	-0.025175269	0.568664	0.637062

ANXA4	-0.102208535	0.020345	0.033846
ANXA5	0.056215784	0.202789	0.265393
ANXA6	-0.173569611	7.51E-05	0.000195
ANXA7	0.26711287	7.31E-10	4.15E-09
ANXA8L1	7.88E-06	0.999858	0.999963
ANXA8L2	-0.01663411	0.706473	0.761198
ANXA8	-0.032002112	0.468658	0.542373
ANXA9	-0.158920987	0.000294	0.000687
AOAH	-0.133257314	0.002443	0.00485
AOC2	0.006634326	0.880613	0.906394
AOC3	-0.314949239	2.54E-13	2.43E-12
AOX1	-0.020684972	0.639551	0.70065
AOX2P	0.209408509	1.64E-06	5.54E-06
AP1AR	0.331655175	1.09E-14	1.28E-13
AP1B1	-0.196632629	6.94E-06	2.13E-05
AP1G1	0.019843334	0.653239	0.712949
AP1G2	-0.214165639	9.32E-07	3.29E-06
AP1M1	-0.19282575	1.05E-05	3.13E-05
AP1M2	-0.107980347	0.014219	0.024459
AP1S1	0.205647019	2.53E-06	8.31E-06
AP1S2	-0.16179321	0.000227	0.000543
AP1S3	0.217272186	6.41E-07	2.32E-06
AP2A1	0.080740332	0.067126	0.099712
AP2A2	-0.242917369	2.36E-08	1.07E-07
AP2B1	0.039958099	0.365491	0.440166
AP2M1	0.10823087	0.013995	0.024104
AP2S1	0.248354424	1.12E-08	5.30E-08
AP3B1	-0.000689902	0.987539	0.99069
AP3B2	-0.205320762	2.62E-06	8.59E-06
AP3D1	-0.042084961	0.340508	0.414626
AP3M1	0.119378617	0.006683	0.012266
AP3M2	-0.052651008	0.232961	0.299179
AP3S1	0.229302819	1.43E-07	5.74E-07
AP3S2	-0.084492492	0.055339	0.083821
AP4B1	-0.26460019	1.07E-09	5.90E-09
AP4E1	-0.045306177	0.304802	0.376568
AP4M1	0.141204528	0.001315	0.002737
AP4S1	0.061862023	0.160974	0.216859
APAF1	0.051683063	0.241676	0.308883
APBA1	-0.264266782	1.12E-09	6.18E-09
APBA2	-0.030381567	0.491484	0.5648
APBA3	-0.094101355	0.032757	0.052113
APBB1IP	-0.221738539	3.71E-07	1.40E-06
APBB1	-0.361036805	2.67E-17	4.66E-16

APBB2	-0.075220339	0.08814	0.127101
APBB3	-0.333417203	7.75E-15	9.31E-14
APC2	-0.019919099	0.652002	0.711754
APCDD1L	0.315079898	2.48E-13	2.37E-12
APCDD1	-0.253303927	5.55E-09	2.75E-08
APCS	0.134016801	0.002306	0.004598
APC	-0.200031752	4.77E-06	1.50E-05
APEH	0.003379495	0.939016	0.951947
APEX1	0.365250594	1.07E-17	1.99E-16
APEX2	0.177241187	5.24E-05	0.000139
APH1A	0.082253567	0.062145	0.093074
APH1B	-0.210732497	1.40E-06	4.80E-06
API5	0.172644925	8.22E-05	0.000212
APIP	-0.049067993	0.266356	0.335852
APITD1	0.077716089	0.078063	0.114108
APLF	-0.034613709	0.433136	0.507803
APLNR	-0.077134335	0.080324	0.117071
APLN	0.034529233	0.43426	0.508852
APLP1	-0.15813512	0.000315	0.000732
APLP2	-0.3246122	4.21E-14	4.53E-13
APOA1BP	0.224494689	2.63E-07	1.01E-06
APOA1	-0.064864987	0.141564	0.193754
APOA2	0.177183015	5.27E-05	0.00014
APOA4	0.069118185	0.117207	0.163782
APOA5	0.03459413	0.433397	0.508048
APOB48R	-0.376003378	9.70E-19	2.11E-17
APOBEC1	0.203922125	3.08E-06	9.96E-06
APOBEC2	-0.196394898	7.13E-06	2.18E-05
APOBEC3A	0.009822838	0.824019	0.859889
APOBEC3B	0.139713554	0.00148	0.003055
APOBEC3C	-0.23055863	1.22E-07	4.95E-07
APOBEC3D	-0.167344367	0.000136	0.000338
APOBEC3F	-0.253572782	5.34E-09	2.66E-08
APOBEC3G	-0.136681014	0.001878	0.003802
APOBEC3H	-0.2455829	1.64E-08	7.59E-08
APOBEC4	-0.208744285	1.77E-06	5.96E-06
APOB	0.032773344	0.458004	0.531872
APOC1P1	-0.025576617	0.562515	0.631837
APOC1	-0.098343077	0.025632	0.04176
APOC2	-0.147261643	0.000802	0.001735
APOC3	0.141388297	0.001295	0.002701
APOC4	-0.169210493	0.000114	0.000287
APOD	-0.280957985	8.49E-11	5.53E-10
APOE	-0.120801985	0.006054	0.01119

APOF	0.241239398	2.97E-08	1.32E-07
APOH	-0.223248924	3.08E-07	1.17E-06
APOL1	0.064273469	0.145238	0.198212
APOL2	0.004093912	0.926158	0.94228
APOL3	-0.161388581	0.000235	0.000561
APOL4	-0.052863358	0.231079	0.297145
APOL5	0.039881006	0.366418	0.441149
APOL6	0.026581326	0.547263	0.617621
APOLD1	-0.042095925	0.340382	0.414498
APOM	-0.070792954	0.108569	0.152995
APOOL	0.106184432	0.015923	0.027073
APOO	0.230867812	1.17E-07	4.76E-07
APPBP2	-0.040998118	0.353135	0.42776
APPL1	0.025207337	0.568172	0.636653
APPL2	0.065730183	0.136318	0.187383
APP	-0.054518254	0.216783	0.281144
APRT	0.136800425	0.001861	0.00377
APTX	0.237677661	4.79E-08	2.06E-07
AQP10	-0.288218632	2.61E-11	1.83E-10
AQP11	0.15628973	0.000371	0.00085
AQP12A	0.065585001	0.137188	0.188436
AQP12B	0.052470116	0.234573	0.301036
AQP1	-0.321345469	7.79E-14	8.02E-13
AQP2	-0.24159713	2.83E-08	1.26E-07
AQP3	-0.256850235	3.33E-09	1.70E-08
AQP4	-0.300729842	3.17E-12	2.55E-11
AQP5	-0.305444135	1.39E-12	1.19E-11
AQP6	-0.207715027	1.99E-06	6.65E-06
AQP7P1	-0.108834015	0.013467	0.023257
AQP7P3	-0.064209434	0.14564	0.198707
AQP7	-0.176327637	5.74E-05	0.000151
AQP8	-0.157128817	0.000344	0.000795
AQP9	0.102874482	0.019537	0.032629
AQPEP	0.127662808	0.003708	0.007118
AQR	0.108090329	0.01412	0.024303
ARAF	-0.180217294	3.90E-05	0.000106
ARAP1	-0.365484422	1.01E-17	1.90E-16
ARAP2	-0.194040235	9.21E-06	2.77E-05
ARAP3	-0.331121686	1.21E-14	1.41E-13
ARCN1	0.24530783	1.70E-08	7.87E-08
ARC	-0.26712026	7.30E-10	4.14E-09
AREG	0.021720111	0.622883	0.685885
ARF1	0.129657979	0.003201	0.006219
ARF3	0.092720644	0.035415	0.055953

ARF4	0.193454804	9.81E-06	2.94E-05
ARF5	0.086088976	0.050873	0.077713
ARF6	0.373873123	1.57E-18	3.32E-17
ARFGAP1	-0.001976	0.96432	0.972493
ARFGAP2	-0.324436277	4.36E-14	4.68E-13
ARFGAP3	0.115935484	0.008451	0.015199
ARFGEF1	0.092349556	0.036159	0.057008
ARFGEF2	0.126080759	0.004161	0.00792
ARFIP1	0.055771228	0.206388	0.269364
ARFIP2	0.170792183	9.81E-05	0.00025
ARFRP1	-0.208188739	1.88E-06	6.33E-06
ARG1	0.011202322	0.799796	0.83975
ARG2	0.229626783	1.37E-07	5.53E-07
ARGFXP2	-0.227366646	1.83E-07	7.23E-07
ARGFX	0.028350115	0.520917	0.593011
ARGLU1	-0.341824295	1.46E-15	1.97E-14
ARHGAP10	-0.08305812	0.059624	0.089613
ARHGAP11A	0.494980902	3.48E-33	6.89E-31
ARHGAP11B	0.334168676	6.69E-15	8.15E-14
ARHGAP12	0.012602407	0.775406	0.819331
ARHGAP15	-0.225963424	2.19E-07	8.53E-07
ARHGAP17	-0.218175302	5.75E-07	2.10E-06
ARHGAP18	-0.156448111	0.000366	0.000839
ARHGAP19	-0.037949232	0.390108	0.464881
ARHGAP1	-0.239489618	3.76E-08	1.65E-07
ARHGAP20	-0.332976987	8.45E-15	1.01E-13
ARHGAP21	0.123631521	0.00496	0.009325
ARHGAP22	-0.05624856	0.202525	0.265082
ARHGAP23	-0.28950559	2.11E-11	1.51E-10
ARHGAP24	-0.38430241	1.43E-19	3.55E-18
ARHGAP25	-0.293734095	1.04E-11	7.79E-11
ARHGAP26	0.020518022	0.642257	0.703036
ARHGAP27	-0.323356122	5.34E-14	5.62E-13
ARHGAP28	-0.168371788	0.000123	0.00031
ARHGAP29	-0.090859025	0.039286	0.061421
ARHGAP30	-0.329001885	1.82E-14	2.07E-13
ARHGAP31	-0.4003649	3.01E-21	9.52E-20
ARHGAP32	-0.03186857	0.470517	0.544241
ARHGAP33	-0.222828316	3.24E-07	1.23E-06
ARHGAP36	0.029001077	0.511388	0.583626
ARHGAP39	0.08358096	0.058031	0.087423
ARHGAP42	-0.072441859	0.100563	0.142995
ARHGAP4	-0.138578812	0.001619	0.003314
ARHGAP5	0.16466485	0.000174	0.000427

ARHGAP6	-0.316017266	2.09E-13	2.02E-12
ARHGAP8	-0.266744397	7.73E-10	4.37E-09
ARHGAP9	-0.256950416	3.29E-09	1.67E-08
ARHGDIA	-0.017226715	0.696525	0.752293
ARHGDIB	-0.323769597	4.94E-14	5.24E-13
ARHGDIG	0.00982087	0.824053	0.859889
ARHGEF10L	-0.279649662	1.05E-10	6.72E-10
ARHGEF10	-0.095408398	0.0304	0.048732
ARHGEF11	-0.211633805	1.26E-06	4.35E-06
ARHGEF12	-0.17835891	4.69E-05	0.000126
ARHGEF15	-0.329259253	1.74E-14	1.98E-13
ARHGEF16	-0.051951084	0.23924	0.306063
ARHGEF17	-0.424128971	6.67E-24	3.15E-22
ARHGEF18	-0.187136804	1.92E-05	5.50E-05
ARHGEF19	-0.3042115	1.73E-12	1.46E-11
ARHGEF1	-0.378884818	5.02E-19	1.14E-17
ARHGEF2	-0.377039862	7.66E-19	1.69E-17
ARHGEF33	-0.051577286	0.242642	0.309939
ARHGEF35	0.041157002	0.351271	0.425812
ARHGEF37	-0.240466022	3.29E-08	1.46E-07
ARHGEF38	-0.122477275	0.005383	0.010057
ARHGEF3	-0.170477462	0.000101	0.000257
ARHGEF4	0.183611401	2.76E-05	7.69E-05
ARHGEF5	-0.026629307	0.54654	0.616979
ARHGEF6	-0.308899258	7.55E-13	6.74E-12
ARHGEF7	-0.259982936	2.11E-09	1.11E-08
ARHGEF9	-0.337882697	3.22E-15	4.13E-14
ARID1A	-0.164795204	0.000172	0.000422
ARID1B	-0.165866858	0.000156	0.000385
ARID2	0.003889118	0.929842	0.945114
ARID3A	0.168984829	0.000117	0.000293
ARID3B	-0.047709529	0.279837	0.350396
ARID3C	0.106438897	0.015671	0.026695
ARID4A	-0.24704835	1.34E-08	6.27E-08
ARID4B	-0.146076577	0.000885	0.001899
ARID5A	-0.196926728	6.72E-06	2.06E-05
ARID5B	-0.188200645	1.72E-05	4.96E-05
ARIH1	0.041176479	0.351043	0.425613
ARIH2	-0.068454448	0.120776	0.168216
ARL10	-0.080304384	0.068621	0.101689
ARL11	-0.030019951	0.496657	0.56984
ARL13A	-0.128973943	0.003368	0.006516
ARL13B	-0.076631777	0.08232	0.119623
ARL14	0.08524441	0.053196	0.080895

ARL15	-0.173852169	7.31E-05	0.00019
ARL16	-0.024245848	0.583026	0.649946
ARL17A	-0.175237702	6.39E-05	0.000167
ARL17B	-0.237308406	5.03E-08	2.16E-07
ARL1	0.264583998	1.07E-09	5.92E-09
ARL2BP	-0.048516824	0.271771	0.341731
ARL2	0.102257468	0.020285	0.033751
ARL3	0.018021766	0.68326	0.740097
ARL4A	0.143253863	0.001115	0.002352
ARL4C	0.08973138	0.041802	0.065042
ARL4D	0.097080046	0.027598	0.044663
ARL5A	0.265437537	9.40E-10	5.26E-09
ARL5B	0.32750584	2.43E-14	2.70E-13
ARL5C	-0.132411011	0.002605	0.005143
ARL6IP1	0.357069505	6.23E-17	1.03E-15
ARL6IP4	0.016613637	0.706818	0.761405
ARL6IP5	-0.053137953	0.228662	0.294396
ARL6IP6	0.298743272	4.46E-12	3.49E-11
ARL6	0.034461359	0.435165	0.509732
ARL8A	-0.106194763	0.015913	0.02706
ARL8B	0.002608586	0.952909	0.96366
ARL9	0.186949482	1.96E-05	5.60E-05
ARMC10	0.244286594	1.96E-08	8.98E-08
ARMC1	0.437381667	1.78E-25	1.07E-23
ARMC2	-0.243492485	2.18E-08	9.92E-08
ARMC3	-0.075771038	0.085831	0.124112
ARMC4	-0.167516351	0.000134	0.000333
ARMC5	-0.314171886	2.92E-13	2.77E-12
ARMC6	0.047804642	0.278878	0.349415
ARMC7	-0.172666467	8.20E-05	0.000211
ARMC8	0.058865453	0.182276	0.241696
ARMC9	-0.313720482	3.17E-13	2.99E-12
ARMCX1	-0.181795665	3.32E-05	9.13E-05
ARMCX2	-0.126500731	0.004036	0.007701
ARMCX3	-0.118609206	0.007046	0.01287
ARMCX5	-0.04856815	0.271263	0.341222
ARMCX6	-0.090682005	0.039673	0.06199
ARMS2	-0.11938131	0.006681	0.012265
ARNT2	-0.250901902	7.80E-09	3.80E-08
ARNTL2	0.379164739	4.71E-19	1.08E-17
ARNTL	-0.160515243	0.000255	0.000603
ARNT	-0.091249082	0.038447	0.060254
ARPC1A	0.331059012	1.23E-14	1.43E-13
ARPC1B	0.108665797	0.013612	0.023492

ARPC2	0.093262928	0.03435	0.054374
ARPC3	0.349340646	3.15E-16	4.70E-15
ARPC4	0.135981064	0.001983	0.004003
ARPC5L	0.183798204	2.71E-05	7.56E-05
ARPC5	0.185031897	2.39E-05	6.72E-05
ARPM1	0.074751236	0.090147	0.129657
ARPP19	0.222994719	3.17E-07	1.21E-06
ARPP21	-0.062478458	0.156834	0.21194
ARR3	-0.034758863	0.431209	0.50581
ARRB1	-0.167860261	0.00013	0.000324
ARRB2	-0.300232042	3.45E-12	2.76E-11
ARRDC1	-0.198208537	5.84E-06	1.81E-05
ARRDC2	-0.199046481	5.32E-06	1.66E-05
ARRDC3	-0.200994426	4.28E-06	1.36E-05
ARRDC4	-0.222352407	3.44E-07	1.30E-06
ARRDC5	-0.21713269	6.52E-07	2.36E-06
ARSA	-0.188538742	1.66E-05	4.80E-05
ARSB	-0.02779415	0.529127	0.601057
ARSD	-0.245469361	1.67E-08	7.70E-08
ARSE	-0.0632642	0.151675	0.205774
ARSF	-0.058915528	0.181903	0.241267
ARSG	-0.208316116	1.86E-06	6.25E-06
ARSH	0.032506914	0.461669	0.535537
ARSI	0.036020313	0.414666	0.489392
ARSJ	0.010151457	0.818232	0.854931
ARSK	-0.037657215	0.393768	0.468657
ART1	-0.05776317	0.190615	0.251287
ART3	-0.009976134	0.821318	0.857527
ART4	-0.328135902	2.15E-14	2.41E-13
ART5	-0.090192584	0.040757	0.063565
ARTN	0.221689429	3.73E-07	1.40E-06
ARV1	-0.172695994	8.18E-05	0.000211
ARVCF	-0.339305517	2.42E-15	3.17E-14
ARX	-0.143684045	0.001076	0.002277
AR	-0.217295274	6.40E-07	2.32E-06
AS3MT	-0.005049484	0.908989	0.928737
ASAH1	-0.207730023	1.99E-06	6.65E-06
ASAH2B	0.125963051	0.004196	0.007981
ASAH2	0.153934788	0.000455	0.001028
ASAM	0.1544082	0.000437	0.000991
ASAP1IT1	0.012523909	0.776768	0.820268
ASAP1	0.160227372	0.000261	0.000618
ASAP2	-0.001263541	0.97718	0.983131
ASAP3	-0.384015454	1.53E-19	3.78E-18

ASB10	0.100249672	0.022892	0.037694
ASB11	-0.136803728	0.00186	0.00377
ASB12	-0.227368375	1.83E-07	7.23E-07
ASB13	0.044990176	0.308191	0.380095
ASB14	-0.229452039	1.40E-07	5.64E-07
ASB15	-0.061731416	0.161861	0.217864
ASB16	-0.239610696	3.70E-08	1.62E-07
ASB17	0.065705071	0.136469	0.187537
ASB18	0.052507088	0.234243	0.30069
ASB1	0.04097309	0.35343	0.428065
ASB2	-0.182733231	3.02E-05	8.36E-05
ASB3	-0.148474511	0.000725	0.001582
ASB4	-0.073874328	0.093995	0.134515
ASB5	-0.0327572	0.458226	0.532099
ASB6	-0.022728569	0.606828	0.671461
ASB7	0.107737944	0.014439	0.024795
ASB8	0.031041759	0.482114	0.555898
ASB9	0.099670303	0.023696	0.038858
ASCC1	0.236567653	5.55E-08	2.37E-07
ASCC2	-0.184981002	2.40E-05	6.75E-05
ASCC3	0.115029782	0.008981	0.01607
ASCL1	0.095231171	0.030711	0.049175
ASCL2	-0.058046273	0.188447	0.248888
ASCL3	-0.144866645	0.000977	0.002082
ASCL4	-0.29218107	1.35E-11	9.95E-11
ASF1A	0.176984207	5.38E-05	0.000143
ASF1B	0.395900136	9.00E-21	2.63E-19
ASFMR1	-0.113381042	0.010022	0.017794
ASGR1	-0.01878425	0.67063	0.728508
ASGR2	0.033509606	0.447961	0.522242
ASH1L	-0.200508475	4.52E-06	1.43E-05
ASH2L	0.042538121	0.33533	0.409236
ASIP	-0.034464833	0.435118	0.509708
ASL	-0.04358579	0.323552	0.396661
ASMTL	-0.271608417	3.68E-10	2.18E-09
ASMT	0.085898695	0.051389	0.078421
ASNA1	0.122974887	0.005197	0.009735
ASNSD1	0.226373376	2.08E-07	8.14E-07
ASNS	0.336819844	3.97E-15	5.02E-14
ASPA	-0.331688961	1.09E-14	1.27E-13
ASPDH	-0.206131213	2.39E-06	7.88E-06
ASPG	-0.014827014	0.737113	0.787209
ASPHD1	-0.114277511	0.009443	0.016844
ASPHD2	0.035406673	0.422666	0.497219

ASPH	0.18291697	2.96E-05	8.22E-05
ASPM	0.488815214	2.75E-32	4.65E-30
ASPN	-0.018471866	0.675793	0.73344
ASPRV1	-0.299673858	3.80E-12	3.01E-11
ASPSCR1	-0.044229801	0.316447	0.388833
ASRGL1	-0.143638232	0.00108	0.002284
ASS1	-0.10319867	0.019154	0.032038
ASTE1	-0.166118109	0.000152	0.000376
ASTL	-0.112019378	0.01096	0.019295
ASTN1	-0.144046494	0.001045	0.002215
ASTN2	-0.185773889	2.21E-05	6.26E-05
ASXL1	-0.04742016	0.282767	0.353534
ASXL2	0.048713852	0.269827	0.339628
ASXL3	-0.083577966	0.05804	0.08743
ASZ1	-0.041936437	0.342217	0.416376
ATAD1	0.249022961	1.02E-08	4.87E-08
ATAD2B	-0.115507318	0.008698	0.015601
ATAD2	0.396020985	8.74E-21	2.56E-19
ATAD3A	0.117695172	0.007501	0.013635
ATAD3B	0.05517419	0.211295	0.274923
ATAD3C	-0.256570014	3.47E-09	1.77E-08
ATAD5	0.237958571	4.61E-08	1.99E-07
ATCAY	-0.024025991	0.586448	0.652996
ATE1	0.217911414	5.94E-07	2.17E-06
ATF1	0.376132498	9.42E-19	2.06E-17
ATF2	0.162675248	0.000209	0.000505
ATF3	-0.102271657	0.020267	0.033725
ATF4	0.218525119	5.51E-07	2.02E-06
ATF5	0.111805603	0.011115	0.019536
ATF6B	-0.046021467	0.297223	0.368512
ATF6	0.077094129	0.080482	0.117276
ATF7IP2	-0.260103432	2.08E-09	1.09E-08
ATF7IP	-0.000988374	0.982149	0.98669
ATF7	-0.13753259	0.001758	0.003575
ATG10	0.052149589	0.237448	0.30412
ATG12	0.176201264	5.81E-05	0.000153
ATG16L1	0.149777301	0.00065	0.001429
ATG16L2	-0.402394489	1.82E-21	6.01E-20
ATG2A	-0.194601805	8.66E-06	2.62E-05
ATG2B	-0.171469291	9.20E-05	0.000235
ATG3	0.09808024	0.026031	0.042349
ATG4A	0.036621268	0.406919	0.481671
ATG4B	-0.174777555	6.68E-05	0.000175
ATG4C	0.163175422	0.0002	0.000484

ATG4D	-0.025992723	0.556173	0.625593
ATG5	0.213332592	1.03E-06	3.61E-06
ATG7	0.00716122	0.87121	0.899657
ATG9A	-0.037124627	0.400497	0.475054
ATG9B	-0.058458286	0.185324	0.245157
ATHL1	-0.169782011	0.000108	0.000274
ATIC	0.334674891	6.06E-15	7.45E-14
ATL1	0.133064335	0.002479	0.004913
ATL2	0.050734598	0.250435	0.318469
ATL3	0.323058298	5.65E-14	5.92E-13
ATMIN	0.22752519	1.79E-07	7.10E-07
ATM	-0.2352458	6.61E-08	2.79E-07
ATN1	-0.135057772	0.002129	0.004277
ATOH1	0.045329918	0.304549	0.37634
ATOH7	0.096410333	0.028693	0.046267
ATOH8	-0.244928489	1.79E-08	8.26E-08
ATOX1	0.155224693	0.000407	0.000927
ATP10A	-0.197121398	6.58E-06	2.02E-05
ATP10B	-0.175998935	5.93E-05	0.000156
ATP10D	-0.148353047	0.000732	0.001597
ATP11A	-0.238651827	4.20E-08	1.83E-07
ATP11B	0.03984096	0.366899	0.441569
ATP11C	0.013273487	0.763792	0.809946
ATP12A	-0.072504293	0.100269	0.142636
ATP13A1	-0.120450234	0.006204	0.011447
ATP13A2	-0.142400932	0.001194	0.002505
ATP13A3	0.264891588	1.02E-09	5.67E-09
ATP13A4	-0.498962662	8.98E-34	1.97E-31
ATP13A5	-0.24496394	1.79E-08	8.23E-08
ATP1A1	-0.294745246	8.81E-12	6.64E-11
ATP1A2	-0.433286264	5.54E-25	3.10E-23
ATP1A3	-0.124641652	0.004615	0.008722
ATP1A4	-0.284747093	4.61E-11	3.11E-10
ATP1B1	-0.03651166	0.408326	0.482993
ATP1B2	-0.354418246	1.09E-16	1.73E-15
ATP1B3	0.242612011	2.46E-08	1.11E-07
ATP1B4	-0.0071579	0.87127	0.899671
ATP2A1	-0.003662335	0.933923	0.948299
ATP2A2	0.28504691	4.39E-11	2.98E-10
ATP2A3	-0.059989074	0.174056	0.232401
ATP2B1	0.257707188	2.95E-09	1.51E-08
ATP2B2	-0.189127001	1.56E-05	4.53E-05
ATP2B3	-0.224785933	2.54E-07	9.80E-07
ATP2B4	-0.111976931	0.010991	0.019335

ATP2C1	0.123892075	0.004869	0.009171
ATP2C2	-0.145500274	0.000928	0.001984
ATP4A	0.010102175	0.819099	0.855658
ATP4B	-0.106563389	0.015549	0.026503
ATP5A1	0.134395164	0.00224	0.004477
ATP5B	0.435251571	3.22E-25	1.87E-23
ATP5C1	0.347344052	4.76E-16	6.92E-15
ATP5D	0.034303068	0.437278	0.511608
ATP5EP2	0.14792439	0.000759	0.00165
ATP5E	0.168954932	0.000117	0.000294
ATP5F1	0.245800719	1.59E-08	7.38E-08
ATP5G1	0.205630049	2.53E-06	8.32E-06
ATP5G2	0.2103523	1.46E-06	5.00E-06
ATP5G3	0.359517622	3.70E-17	6.33E-16
ATP5H	0.196556287	7.00E-06	2.14E-05
ATP5I	0.130700421	0.002962	0.005792
ATP5J2	0.311917405	4.39E-13	4.05E-12
ATP5J	0.16166509	0.00023	0.000549
ATP5L2	0.058130233	0.187807	0.248191
ATP5L	0.174284488	7.01E-05	0.000183
ATP5O	0.089114565	0.043234	0.067005
ATP5SL	0.096743916	0.028143	0.045468
ATP5S	0.267341704	7.06E-10	4.02E-09
ATP6AP1L	-0.232681898	9.24E-08	3.83E-07
ATP6AP1	-0.122485601	0.00538	0.010052
ATP6AP2	0.110464221	0.012127	0.021131
ATP6V0A1	-0.450110508	4.68E-27	3.45E-25
ATP6V0A2	0.174739969	6.70E-05	0.000175
ATP6V0A4	0.053559711	0.224984	0.29024
ATP6V0B	0.094638619	0.03177	0.050708
ATP6V0C	-0.106824691	0.015296	0.026116
ATP6V0D1	-0.217190435	6.48E-07	2.35E-06
ATP6V0D2	-0.206036641	2.42E-06	7.96E-06
ATP6V0E1	-0.109249564	0.013114	0.022717
ATP6V0E2	-0.121873144	0.005617	0.010458
ATP6V1A	0.060897035	0.167619	0.224811
ATP6V1B1	-0.105851928	0.016258	0.027588
ATP6V1B2	-0.166138532	0.000152	0.000376
ATP6V1C1	0.214201517	9.28E-07	3.28E-06
ATP6V1C2	0.062529578	0.156494	0.211566
ATP6V1D	0.207944392	1.94E-06	6.50E-06
ATP6V1E1	0.047187131	0.285142	0.356058
ATP6V1E2	0.104816049	0.017339	0.029237
ATP6V1F	0.154104293	0.000449	0.001015

ATP6V1G1	0.140094101	0.001436	0.00297
ATP6V1G2	-0.11734323	0.007683	0.013936
ATP6V1G3	-0.118510631	0.007094	0.01295
ATP6V1H	0.097236569	0.027348	0.044283
ATP7A	-0.182863599	2.98E-05	8.26E-05
ATP7B	0.039268986	0.373824	0.448715
ATP8A1	-0.332218666	9.80E-15	1.16E-13
ATP8A2	-0.270557255	4.33E-10	2.54E-09
ATP8B1	0.083544733	0.05814	0.087568
ATP8B2	-0.257341792	3.11E-09	1.59E-08
ATP8B3	0.100898073	0.02202	0.036384
ATP8B4	-0.24416717	1.99E-08	9.12E-08
ATP8B5P	-0.13934683	0.001524	0.003138
ATP9A	-0.222468721	3.39E-07	1.28E-06
ATP9B	-0.207763135	1.98E-06	6.62E-06
ATPAF1	-0.103545605	0.018751	0.03143
ATPAF2	-0.037945591	0.390153	0.464881
ATPBD4	0.240655607	3.21E-08	1.42E-07
ATPIF1	-0.158742539	0.000298	0.000697
ATRIP	-0.013214628	0.764809	0.810723
ATRNL1	-0.20731493	2.09E-06	6.94E-06
ATRN	-0.091177482	0.0386	0.06046
ATRX	-0.162961015	0.000204	0.000493
ATR	0.048043825	0.276477	0.34695
ATXN10	0.006897268	0.875918	0.903072
ATXN1L	-0.233623051	8.17E-08	3.41E-07
ATXN1	-0.229483479	1.40E-07	5.62E-07
ATXN2L	-0.012615362	0.775182	0.819199
ATXN2	0.016056119	0.716224	0.76901
ATXN3L	0.006240947	0.887643	0.911915
ATXN3	-0.091988572	0.036897	0.058024
ATXN7L1	-0.192733394	1.06E-05	3.16E-05
ATXN7L2	-0.184277578	2.58E-05	7.22E-05
ATXN7L3B	0.218708811	5.39E-07	1.98E-06
ATXN7L3	0.108841546	0.01346	0.023248
ATXN7	-0.30413486	1.75E-12	1.47E-11
ATXN8OS	0.065408885	0.138249	0.189736
AUH	-0.029354295	0.506255	0.57898
AUP1	0.141521735	0.001282	0.002675
AURKAIP1	0.08864458	0.044353	0.068606
AURKAPS1	0.215643645	7.81E-07	2.79E-06
AURKA	0.546669009	1.81E-41	1.91E-38
AURKB	0.505380581	9.72E-35	2.62E-32
AURKC	-0.093946785	0.033046	0.052497

AUTS2	-0.379476711	4.38E-19	1.01E-17
AVEN	0.355489677	8.72E-17	1.40E-15
AVIL	-0.222532249	3.36E-07	1.28E-06
AVL9	0.317844061	1.49E-13	1.48E-12
AVPI1	0.023243075	0.598708	0.664131
AVPR1A	0.133627613	0.002376	0.004727
AVPR1B	-0.161488264	0.000233	0.000557
AVPR2	-0.200364106	4.59E-06	1.45E-05
AVP	0.043535794	0.324108	0.397172
AWAT1	0.103084626	0.019288	0.032246
AWAT2	-0.046451506	0.292727	0.363983
AXIN1	-0.082148433	0.062481	0.093493
AXIN2	-0.279216651	1.12E-10	7.15E-10
AXL	-0.140127877	0.001432	0.002962
AZGP1	-0.101698195	0.020984	0.034833
AZI1	-0.146859781	0.000829	0.001789
AZI2	0.047406977	0.282901	0.353679
AZIN1	0.345895743	6.41E-16	9.10E-15
AZU1	-0.332300316	9.64E-15	1.14E-13
B2M	-0.050739411	0.25039	0.31845
B3GALNT1	0.13030038	0.003052	0.005952
B3GALNT2	0.207439872	2.06E-06	6.85E-06
B3GALT1	0.10816301	0.014055	0.0242
B3GALT2	-0.258704748	2.55E-09	1.32E-08
B3GALT4	-0.154997864	0.000415	0.000944
B3GALT5	0.032543312	0.461167	0.535017
B3GALT6	-0.036564304	0.40765	0.482422
B3GALTL	-0.093101415	0.034664	0.054841
B3GAT1	-0.370615384	3.26E-18	6.53E-17
B3GAT2	-0.196660943	6.92E-06	2.12E-05
B3GAT3	-0.02160448	0.624736	0.687471
B3GNT1	-0.273615039	2.70E-10	1.63E-09
B3GNT2	0.032041747	0.468107	0.54181
B3GNT3	0.161535945	0.000232	0.000555
B3GNT4	0.23218979	9.85E-08	4.06E-07
B3GNT5	0.340348055	1.97E-15	2.61E-14
B3GNT6	0.051251675	0.245633	0.313279
B3GNT7	-0.289331228	2.18E-11	1.54E-10
B3GNT8	-0.415995224	5.71E-23	2.33E-21
B3GNT9	-0.323786483	4.92E-14	5.23E-13
B3GNTL1	-0.057638346	0.191576	0.252421
B4GALNT1	0.139325505	0.001527	0.003142
B4GALNT2	0.192796466	1.05E-05	3.14E-05
B4GALNT3	-0.186273037	2.10E-05	5.97E-05

B4GALNT4	0.049241853	0.264663	0.33397
B4GALT1	0.356321773	7.31E-17	1.19E-15
B4GALT2	0.201767118	3.93E-06	1.25E-05
B4GALT3	0.180316042	3.86E-05	0.000105
B4GALT4	0.145327651	0.000941	0.00201
B4GALT5	0.121711451	0.005681	0.010563
B4GALT6	0.171488983	9.18E-05	0.000235
B4GALT7	-0.101178051	0.021652	0.035845
B9D1	-0.012070173	0.784653	0.827031
B9D2	-0.191863509	1.16E-05	3.45E-05
BAALC	0.023937214	0.587833	0.654275
BAAT	-0.195281103	8.05E-06	2.44E-05
BACE1	-0.200354197	4.60E-06	1.45E-05
BACE2	0.055551807	0.208182	0.271438
BACH1	0.131708091	0.002747	0.005403
BACH2	-0.095341395	0.030517	0.0489
BAD	-0.125986909	0.004189	0.007969
BAG1	0.052794727	0.231686	0.297791
BAG2	0.406433693	6.62E-22	2.31E-20
BAG3	0.100533243	0.022507	0.037106
BAG4	0.184102034	2.63E-05	7.35E-05
BAG5	0.151314767	0.00057	0.001266
BAGE2	0.040817607	0.355261	0.429736
BAGE	0.14483835	0.00098	0.002086
BAHCC1	-0.289653679	2.06E-11	1.47E-10
BAHD1	-0.192346574	1.10E-05	3.29E-05
BAI1	-0.163264031	0.000198	0.00048
BAI2	-0.086260082	0.050413	0.077114
BAI3	-0.289643738	2.07E-11	1.47E-10
BAIAP2L1	0.100702134	0.02228	0.036771
BAIAP2L2	0.101903749	0.020724	0.034431
BAIAP2	-0.29475949	8.79E-12	6.63E-11
BAIAP3	-0.192842242	1.05E-05	3.13E-05
BAK1	0.103462632	0.018847	0.031567
BAMBI	-0.089356933	0.042666	0.066223
BANF1	0.312138671	4.22E-13	3.91E-12
BANF2	0.159735465	0.000273	0.000643
BANK1	-0.294941665	8.52E-12	6.45E-11
BANP	0.097420397	0.027056	0.043864
BAP1	-0.222592817	3.34E-07	1.27E-06
BARD1	0.152529113	0.000514	0.00115
BARHL1	-0.011437812	0.795679	0.836383
BARHL2	-0.02143315	0.627485	0.690078
BARX1	0.163765106	0.000189	0.00046

BARX2	0.044908097	0.309076	0.38097
BASE	0.020451861	0.643331	0.70396
BASP1	0.262213581	1.52E-09	8.21E-09
BAT1	-0.088424263	0.044886	0.069382
BAT2L1	-0.109220716	0.013138	0.022755
BAT2L2	0.02113018	0.63236	0.694026
BAT2	0.020554262	0.641669	0.702547
BAT3	-0.03812102	0.387964	0.462769
BAT4	0.013829271	0.754213	0.801703
BAT5	-0.087483552	0.047222	0.072602
BATF2	0.035713987	0.418649	0.493364
BATF3	0.144417472	0.001014	0.002156
BATF	-0.023194319	0.599476	0.664835
BAX	-0.00360135	0.935021	0.948992
BAZ1A	0.161389115	0.000235	0.000561
BAZ1B	0.03812247	0.387946	0.462769
BAZ2A	0.048852169	0.268467	0.338151
BAZ2B	-0.229544263	1.39E-07	5.58E-07
BBC3	-0.164178565	0.000182	0.000445
BBOX1	-0.1900313	1.41E-05	4.15E-05
BBS10	0.079161794	0.072667	0.10701
BBS12	-0.004869798	0.912215	0.931366
BBS1	-0.413071369	1.22E-22	4.71E-21
BBS2	-0.297164241	5.84E-12	4.52E-11
BBS4	-0.139428858	0.001514	0.003119
BBS5	-0.377605185	6.73E-19	1.50E-17
BBS7	0.195049764	8.25E-06	2.50E-05
BBS9	-0.187385123	1.87E-05	5.37E-05
BBX	0.030675334	0.487302	0.560684
BCAM	-0.365159425	1.09E-17	2.02E-16
BCAN	0.198560928	5.61E-06	1.75E-05
BCAP29	0.083398489	0.058583	0.088201
BCAP31	0.040279828	0.36164	0.436287
BCAR1	0.001070938	0.980658	0.985674
BCAR3	0.167707786	0.000131	0.000328
BCAR4	0.087232131	0.047864	0.073457
BCAS1	-0.005773081	0.896017	0.918581
BCAS2	0.163595564	0.000192	0.000467
BCAS3	-0.211787643	1.24E-06	4.27E-06
BCAS4	-0.103668649	0.01861	0.031215
BCAT1	-0.058333815	0.186263	0.246298
BCAT2	-0.216874885	6.73E-07	2.43E-06
BCCIP	0.417659255	3.70E-23	1.56E-21
BCDIN3D	0.048998523	0.267034	0.336622

BCHE	-0.199939683	4.82E-06	1.51E-05
BCKDHA	-0.249223954	9.88E-09	4.74E-08
BCKDHB	-0.145013446	0.000966	0.002059
BCKDK	-0.013830248	0.754197	0.801703
BCL10	0.115850281	0.0085	0.015276
BCL11A	-0.209537914	1.61E-06	5.47E-06
BCL11B	-0.179071103	4.37E-05	0.000118
BCL2A1	0.03817141	0.387337	0.462134
BCL2L10	0.19095129	1.28E-05	3.78E-05
BCL2L11	0.046050999	0.296912	0.368271
BCL2L12	0.181880409	3.29E-05	9.06E-05
BCL2L13	0.172838889	8.06E-05	0.000208
BCL2L14	-0.16683434	0.000143	0.000354
BCL2L15	-0.170435005	0.000102	0.000258
BCL2L1	-0.069113216	0.117234	0.163808
BCL2L2	-0.200923666	4.32E-06	1.37E-05
BCL2	-0.21889686	5.26E-07	1.94E-06
BCL3	0.091740641	0.03741	0.058763
BCL6B	-0.059545489	0.177268	0.236199
BCL6	-0.276409781	1.75E-10	1.08E-09
BCL7A	-0.100618831	0.022392	0.036937
BCL7B	0.065157112	0.139776	0.191557
BCL7C	-0.045897129	0.298531	0.369911
BCL8	-0.058261274	0.186812	0.246975
BCL9L	-0.006201124	0.888356	0.912459
BCL9	0.017830091	0.686449	0.743028
BCLAF1	-0.080927428	0.066493	0.098889
BCMO1	-0.12787745	0.00365	0.007012
BCO2	-0.319060233	1.19E-13	1.20E-12
BCORL1	-0.118332789	0.007181	0.013096
BCORL2	0.106337418	0.015771	0.026848
BCOR	-0.095052586	0.031027	0.049629
BCR	-0.142207272	0.001213	0.002542
BCS1L	0.086104602	0.050831	0.077665
BCYRN1	0.011125354	0.801142	0.840704
BDH1	-0.00870325	0.843803	0.87638
BDH2	-0.2855621	4.04E-11	2.75E-10
BDKRB1	0.209505192	1.62E-06	5.48E-06
BDKRB2	0.101657387	0.021035	0.034913
BDNFOS	-0.364214013	1.34E-17	2.45E-16
BDNF	-0.00636142	0.885489	0.910263
BDP1	-0.027032102	0.540487	0.611285
BEAN	-0.013981764	0.751592	0.799554
BECN1	-0.062917389	0.153936	0.208474

BEGAIN	-0.072820085	0.098794	0.140748
BEND2	-0.046756668	0.289564	0.360678
BEND3	0.13143331	0.002804	0.005504
BEND4	-0.075706433	0.0861	0.124437
BEND5	-0.333692045	7.35E-15	8.88E-14
BEND6	0.210303859	1.47E-06	5.02E-06
BEND7	-0.216029981	7.45E-07	2.67E-06
BEST1	-0.125580567	0.004314	0.00819
BEST2	0.069490468	0.115242	0.161408
BEST3	0.248541999	1.09E-08	5.17E-08
BEST4	-0.111879944	0.011061	0.019453
BET1L	-0.093634538	0.033636	0.053362
BET1	0.075275322	0.087908	0.126793
BET3L	0.026513137	0.548291	0.618642
BEX1	-0.10385556	0.018398	0.030887
BEX2	-0.265489438	9.33E-10	5.22E-09
BEX4	-0.339947408	2.13E-15	2.81E-14
BEX5	-0.193018871	1.03E-05	3.07E-05
BEYLA	-0.038480733	0.383499	0.458082
BFAR	-0.021024411	0.634065	0.695592
BFSP1	0.058001065	0.188792	0.249245
BFSP2	0.023283008	0.59808	0.663619
BGLAP	-0.039008668	0.377002	0.451797
BGN	-0.073631233	0.095085	0.135909
BHLHA15	0.210688919	1.41E-06	4.82E-06
BHLHB9	-0.057248005	0.194607	0.255792
BHLHE22	-0.199168876	5.25E-06	1.64E-05
BHLHE23	0.002339215	0.957767	0.967689
BHLHE40	-0.126358055	0.004078	0.007775
BHLHE41	-0.212283619	1.17E-06	4.04E-06
BHMT2	0.034466281	0.435099	0.509708
BHMT	-0.068363384	0.121272	0.168849
BICC1	-0.110032769	0.01247	0.021674
BICD1	0.130288414	0.003055	0.005956
BICD2	-0.209199235	1.68E-06	5.67E-06
BID	0.147353971	0.000796	0.001723
BIK	0.164200934	0.000182	0.000444
BIN1	-0.004949219	0.910789	0.93029
BIN2	-0.210477906	1.44E-06	4.93E-06
BIN3	-0.147542808	0.000784	0.001698
BIRC2	0.151304067	0.000571	0.001267
BIRC3	-0.01425341	0.746929	0.795397
BIRC5	0.504031941	1.56E-34	3.99E-32
BIRC6	-0.075385611	0.087442	0.126213

BIRC7	-0.251873567	6.80E-09	3.33E-08
BIRC8	0.010636439	0.809711	0.847888
BIVM	-0.126409335	0.004063	0.007747
BLCAP	-0.187963394	1.76E-05	5.08E-05
BLID	0.167539741	0.000134	0.000333
BLK	-0.263938916	1.18E-09	6.46E-09
BLMH	0.033556015	0.447332	0.5216
BLM	0.411943513	1.63E-22	6.12E-21
BLNK	-0.318715539	1.27E-13	1.27E-12
BLOC1S1	0.048401719	0.272911	0.343035
BLOC1S2	0.180244362	3.89E-05	0.000106
BLOC1S3	-0.026403016	0.549955	0.620176
BLVRA	-0.128679455	0.003442	0.006647
BLVRB	0.005102599	0.908036	0.928095
BLZF1	0.183661126	2.75E-05	7.66E-05
BMF	-0.23540868	6.46E-08	2.74E-07
BMI1	0.098158313	0.025912	0.042179
BMP10	-0.091225651	0.038497	0.060327
BMP15	-0.100146498	0.023033	0.037899
BMP1	0.041904674	0.342583	0.416615
BMP2K	-0.063896054	0.14762	0.200942
BMP2	-0.10213132	0.020441	0.033993
BMP3	-0.334495923	6.28E-15	7.70E-14
BMP4	-0.120231798	0.006299	0.011611
BMP5	-0.180743579	3.69E-05	0.000101
BMP6	0.06455272	0.143495	0.196114
BMP7	-0.184679007	2.47E-05	6.95E-05
BMP8A	0.089173525	0.043095	0.066816
BMP8B	-0.001638818	0.970405	0.977693
BMPER	-0.231025595	1.15E-07	4.68E-07
BMPR1A	0.128021213	0.003612	0.006946
BMPR1B	0.10509823	0.017039	0.028803
BMPR2	-0.180314411	3.86E-05	0.000105
BMS1P4	-0.225241258	2.40E-07	9.29E-07
BMS1P5	-0.272329766	3.29E-10	1.96E-09
BMS1	0.216139413	7.36E-07	2.64E-06
BMX	-0.231466143	1.08E-07	4.44E-07
BNC1	0.037165504	0.399978	0.47456
BNC2	-0.052014555	0.238666	0.305446
BNIP1	0.168654374	0.00012	0.000302
BNIP2	-0.076541453	0.082683	0.120098
BNIP3L	-0.141790283	0.001254	0.002623
BNIP3	0.209054694	1.70E-06	5.76E-06
BNIPL	-0.303021141	2.13E-12	1.77E-11

BOC	-0.246773121	1.39E-08	6.51E-08
BOD1L	-0.11439501	0.00937	0.016719
BOD1	0.134818897	0.002169	0.004347
BOK	-0.229170196	1.45E-07	5.83E-07
BOLA1	0.106846369	0.015275	0.026083
BOLA2	0.302299239	2.41E-12	1.98E-11
BOLA3	0.41303913	1.23E-22	4.73E-21
BOLL	0.077737891	0.077979	0.113994
BOP1	0.289827948	2.00E-11	1.43E-10
BPESC1	0.060240333	0.172257	0.230275
BPGM	-0.071947943	0.10291	0.145884
BPHL	0.208269591	1.87E-06	6.28E-06
BPIL1	0.01949819	0.658886	0.718054
BPIL2	-0.055525508	0.208398	0.271684
BPIL3	0.026982073	0.541237	0.611898
BPI	-0.120321379	0.00626	0.011543
BPNT1	0.161231385	0.000239	0.000568
BPTF	-0.143447835	0.001097	0.002318
BPY2	-0.017145062	0.697893	0.753617
BRAF	0.043419579	0.325402	0.398563
BRAP	0.22320703	3.09E-07	1.18E-06
BRCA1	0.338722082	2.72E-15	3.54E-14
BRCA2	0.247378081	1.28E-08	6.01E-08
BRCC3	0.162674977	0.000209	0.000505
BRD1	-0.349090082	3.32E-16	4.93E-15
BRD2	-0.02985101	0.499084	0.572165
BRD3	-0.022239206	0.614596	0.678329
BRD4	-0.004904542	0.911591	0.930914
BRD7P3	0.006892988	0.875995	0.903104
BRD7	0.045849408	0.299034	0.37042
BRD8	-0.173135246	7.84E-05	0.000202
BRD9	-0.031012378	0.482528	0.55628
BRDT	0.048091834	0.275997	0.346435
BREA2	-0.176528454	5.63E-05	0.000149
BRE	-0.059352933	0.178675	0.237742
BRF1	-0.127048228	0.003878	0.007422
BRF2	0.127340297	0.003797	0.007276
BRI3BP	0.397979677	5.42E-21	1.64E-19
BRI3	-0.013801028	0.754699	0.801979
BRIP1	0.324307467	4.46E-14	4.77E-13
BRIX1	0.373352261	1.77E-18	3.68E-17
BRMS1L	0.188696534	1.63E-05	4.72E-05
BRMS1	0.147972391	0.000756	0.001644
BRP44L	-0.157012113	0.000348	0.000802

BRP44	0.046020217	0.297236	0.368512
BRPF1	-0.10466704	0.0175	0.029498
BRPF3	-0.069248708	0.116515	0.162963
BRS3	0.029023592	0.51106	0.583351
BRSK1	-0.041915908	0.342453	0.416584
BRSK2	0.037331978	0.397869	0.472582
BRWD1	-0.303548604	1.94E-12	1.62E-11
BRWD3	-0.01554092	0.724955	0.776882
BSCL2	-0.126943522	0.003908	0.007474
BSDC1	-0.289024267	2.29E-11	1.62E-10
BSG	0.075082665	0.088725	0.127825
BSND	0.047861765	0.278303	0.348854
BSN	-0.20516081	2.67E-06	8.73E-06
BSPRY	-0.018484379	0.675586	0.733255
BST1	-0.077599413	0.078512	0.114698
BST2	-0.064805265	0.141932	0.194204
BSX	0.033365378	0.449918	0.524279
BTAF1	-0.177008447	5.36E-05	0.000142
BTBD10	0.183975059	2.66E-05	7.44E-05
BTBD11	0.089605806	0.04209	0.065435
BTBD12	-0.137706064	0.001734	0.003533
BTBD16	0.188718306	1.62E-05	4.71E-05
BTBD17	0.035065573	0.427152	0.501758
BTBD18	-0.19509385	8.21E-06	2.49E-05
BTBD19	-0.217718411	6.08E-07	2.21E-06
BTBD1	0.170156962	0.000104	0.000265
BTBD2	0.000670459	0.98789	0.990964
BTBD3	-0.007974535	0.856733	0.88709
BTBD6	0.052168667	0.237276	0.303978
BTBD7	-0.022178121	0.615569	0.679215
BTBD8	0.000688969	0.987556	0.99069
BTBD9	-0.401797492	2.11E-21	6.88E-20
BTC	-0.160823798	0.000248	0.000588
BTD	-0.164214169	0.000182	0.000443
BTF3L1	0.034173217	0.439016	0.513311
BTF3L4	0.212987203	1.07E-06	3.75E-06
BTF3	0.136723993	0.001872	0.003791
BTG1	0.007183998	0.870804	0.899284
BTG2	-0.358990831	4.14E-17	7.03E-16
BTG3	-0.192110368	1.13E-05	3.36E-05
BTG4	-0.10816487	0.014053	0.024199
BTK	-0.264025974	1.16E-09	6.39E-09
BTLA	-0.204355509	2.93E-06	9.52E-06
BTN1A1	-0.141687318	0.001265	0.002644

BTN2A1	-0.250222199	8.59E-09	4.15E-08
BTN2A2	-0.398413585	4.87E-21	1.49E-19
BTN2A3	-0.264290774	1.12E-09	6.16E-09
BTN3A1	-0.218250601	5.70E-07	2.08E-06
BTN3A2	-0.199946338	4.81E-06	1.51E-05
BTN3A3	-0.243502489	2.18E-08	9.91E-08
BTNL2	-0.085471562	0.052563	0.08003
BTNL3	-0.076213177	0.084013	0.121746
BTNL8	-0.193994887	9.25E-06	2.78E-05
BTNL9	-0.48000762	4.88E-31	6.38E-29
BTRC	-0.081804998	0.063589	0.094958
BUB1B	0.507759501	4.21E-35	1.22E-32
BUB1	0.474353926	2.96E-30	3.44E-28
BUB3	0.473943881	3.37E-30	3.90E-28
BUD13	0.120375134	0.006237	0.011501
BUD31	0.275601077	1.98E-10	1.22E-09
BVES	0.038929609	0.377971	0.452781
BYSL	0.421142239	1.48E-23	6.58E-22
BZRAP1	-0.443797862	2.90E-26	1.95E-24
BZW1	0.468943289	1.61E-29	1.65E-27
BZW2	0.403009109	1.56E-21	5.20E-20
C10orf105	-0.46867111	1.76E-29	1.77E-27
C10orf107	-0.315817882	2.16E-13	2.09E-12
C10orf108	0.06075907	0.168585	0.225956
C10orf10	-0.172784685	8.11E-05	0.000209
C10orf110	-0.150485316	0.000612	0.001351
C10orf111	-0.168899312	0.000117	0.000296
C10orf113	-0.058149581	0.18766	0.24803
C10orf114	0.086713239	0.049211	0.075397
C10orf116	-0.247890526	1.19E-08	5.63E-08
C10orf118	-0.103723342	0.018548	0.031121
C10orf119	0.359365397	3.82E-17	6.52E-16
C10orf11	-0.243872709	2.07E-08	9.46E-08
C10orf120	0.023840424	0.589344	0.655562
C10orf122	0.005544153	0.900118	0.921966
C10orf125	-0.01115237	0.80067	0.840403
C10orf128	-0.226542536	2.03E-07	7.98E-07
C10orf129	-0.001296585	0.976583	0.982729
C10orf12	0.1735437	7.53E-05	0.000195
C10orf131	-0.045305653	0.304808	0.376568
C10orf137	-0.095999369	0.029383	0.047268
C10orf140	-0.148991198	0.000694	0.00152
C10orf18	0.164311388	0.00018	0.00044
C10orf25	0.01902452	0.666668	0.725151

C10orf26	-0.133574764	0.002385	0.004745
C10orf27	0.070724664	0.108911	0.153434
C10orf28	-0.14106563	0.001329	0.002764
C10orf2	0.386392237	8.77E-20	2.20E-18
C10orf32	-0.176929754	5.41E-05	0.000143
C10orf35	-0.01785838	0.685978	0.742719
C10orf40	0.05027564	0.254752	0.323287
C10orf41	-0.020182947	0.647702	0.707949
C10orf46	0.207831931	1.96E-06	6.58E-06
C10orf47	0.050729368	0.250484	0.318511
C10orf4	-0.067711648	0.12487	0.173122
C10orf50	-0.269893327	4.79E-10	2.79E-09
C10orf53	-0.162239453	0.000218	0.000524
C10orf54	-0.330439741	1.38E-14	1.60E-13
C10orf55	-0.002476938	0.955283	0.96567
C10orf57	0.091277479	0.038386	0.060163
C10orf58	0.065191636	0.139566	0.191307
C10orf62	0.07430636	0.092083	0.132043
C10orf67	-0.26136606	1.72E-09	9.20E-09
C10orf68	-0.314518212	2.74E-13	2.61E-12
C10orf71	0.028712972	0.515594	0.58787
C10orf72	-0.295253788	8.08E-12	6.14E-11
C10orf75	0.011233059	0.799258	0.839362
C10orf76	-0.290404333	1.82E-11	1.31E-10
C10orf78	0.22517369	2.42E-07	9.36E-07
C10orf79	-0.231679006	1.05E-07	4.32E-07
C10orf81	-0.169834606	0.000108	0.000273
C10orf82	0.080567152	0.067717	0.100484
C10orf84	0.221712889	3.72E-07	1.40E-06
C10orf88	0.258077986	2.79E-09	1.44E-08
C10orf90	0.294051259	9.90E-12	7.41E-11
C10orf91	0.081316	0.065194	0.097116
C10orf93	-0.178871972	4.46E-05	0.00012
C10orf95	-0.177085305	5.32E-05	0.000141
C10orf96	0.06511752	0.140017	0.191807
C10orf99	-0.00967671	0.826595	0.861956
C11orf10	0.255840091	3.86E-09	1.95E-08
C11orf16	-0.217759546	6.05E-07	2.20E-06
C11orf17	0.07163657	0.104412	0.147665
C11orf1	-0.025188572	0.56846	0.636904
C11orf20	0.102703539	0.019742	0.032944
C11orf21	-0.389289475	4.41E-20	1.16E-18
C11orf24	0.321420712	7.68E-14	7.92E-13
C11orf2	-0.156973479	0.000349	0.000805

C11orf30	0.015699652	0.722261	0.774411
C11orf31	0.144738801	0.000988	0.002102
C11orf34	-0.057015377	0.196429	0.257948
C11orf35	-0.325807289	3.36E-14	3.67E-13
C11orf36	0.075305353	0.087781	0.126656
C11orf41	0.037041163	0.401557	0.47617
C11orf42	-0.17307345	7.88E-05	0.000204
C11orf45	-0.20066202	4.44E-06	1.40E-05
C11orf46	-0.003230187	0.941705	0.953778
C11orf48	0.286979202	3.20E-11	2.22E-10
C11orf49	-0.151460731	0.000563	0.001252
C11orf51	0.134977439	0.002142	0.004301
C11orf52	-0.252944733	5.84E-09	2.89E-08
C11orf53	0.031849003	0.470789	0.544505
C11orf54	-0.081167631	0.065688	0.097819
C11orf57	0.121696508	0.005687	0.010573
C11orf58	0.139977126	0.00145	0.002995
C11orf59	0.172412546	8.40E-05	0.000216
C11orf61	-0.326712742	2.83E-14	3.12E-13
C11orf63	-0.211624369	1.26E-06	4.35E-06
C11orf64	0.049632088	0.260891	0.329689
C11orf65	-0.189216345	1.54E-05	4.49E-05
C11orf66	-0.317659703	1.54E-13	1.52E-12
C11orf67	-0.105861028	0.016248	0.027575
C11orf68	-0.109700266	0.01274	0.022104
C11orf70	-0.115450961	0.008731	0.015655
C11orf71	-0.110024262	0.012477	0.02168
C11orf73	0.223314651	3.05E-07	1.17E-06
C11orf74	-0.102339813	0.020184	0.033601
C11orf75	-0.094333196	0.032328	0.051496
C11orf80	0.105085492	0.017052	0.028821
C11orf82	0.437764532	1.60E-25	9.66E-24
C11orf83	0.205675193	2.52E-06	8.28E-06
C11orf84	0.168718086	0.00012	0.0003
C11orf85	0.028893542	0.512956	0.585281
C11orf86	0.211165407	1.33E-06	4.58E-06
C11orf87	-0.023719984	0.591226	0.657254
C11orf88	-0.22622352	2.12E-07	8.28E-07
C11orf90	0.082221926	0.062246	0.093211
C11orf92	-0.415131674	7.15E-23	2.86E-21
C11orf93	-0.365454978	1.02E-17	1.91E-16
C11orf94	0.003804542	0.931364	0.946228
C11orf95	-0.092514629	0.035826	0.056541
C11orf9	-0.095021366	0.031082	0.049706

C12orf10	0.185192573	2.35E-05	6.61E-05
C12orf11	0.444439775	2.41E-26	1.65E-24
C12orf12	-0.028717138	0.515533	0.587858
C12orf23	0.432209245	7.45E-25	4.06E-23
C12orf24	0.269271825	5.27E-10	3.05E-09
C12orf26	0.070863017	0.108219	0.152566
C12orf27	-0.125493773	0.004341	0.008239
C12orf29	0.405215898	9.00E-22	3.08E-20
C12orf32	0.344020999	9.39E-16	1.30E-14
C12orf34	0.247889489	1.19E-08	5.63E-08
C12orf35	-0.043902267	0.320048	0.392823
C12orf36	0.101172806	0.021659	0.03585
C12orf39	0.248262363	1.13E-08	5.36E-08
C12orf40	-0.029992649	0.497049	0.570126
C12orf41	0.241682809	2.79E-08	1.25E-07
C12orf42	-0.033938653	0.442166	0.516481
C12orf43	0.292689732	1.24E-11	9.18E-11
C12orf44	0.298516531	4.64E-12	3.63E-11
C12orf45	0.324875967	4.01E-14	4.33E-13
C12orf47	0.16400279	0.000185	0.000451
C12orf48	0.547575294	1.26E-41	1.48E-38
C12orf49	0.006201563	0.888348	0.912459
C12orf4	0.221322537	3.91E-07	1.46E-06
C12orf50	-0.009052762	0.837615	0.870949
C12orf51	-0.144202561	0.001032	0.002189
C12orf52	0.239059878	3.98E-08	1.74E-07
C12orf53	-0.062383309	0.157468	0.212724
C12orf54	-0.053732612	0.223489	0.288572
C12orf56	0.214437155	9.03E-07	3.19E-06
C12orf57	0.12029009	0.006274	0.011567
C12orf59	-0.171012395	9.61E-05	0.000245
C12orf5	0.296676056	6.35E-12	4.89E-11
C12orf60	0.121503132	0.005765	0.0107
C12orf61	0.023334202	0.597275	0.662873
C12orf62	0.007759595	0.860555	0.890309
C12orf63	-0.191640143	1.19E-05	3.53E-05
C12orf65	0.10543558	0.016685	0.028246
C12orf66	0.287402729	2.99E-11	2.08E-10
C12orf68	-0.151570746	0.000558	0.00124
C12orf69	-0.324232616	4.53E-14	4.83E-13
C12orf70	0.236831989	5.36E-08	2.29E-07
C12orf71	-0.244941592	1.79E-08	8.25E-08
C12orf72	-0.238001995	4.58E-08	1.98E-07
C12orf73	0.323962238	4.76E-14	5.07E-13

C12orf74	-0.153285047	0.000482	0.001083
C12orf75	0.07666348	0.082193	0.119455
C12orf76	-0.16083441	0.000247	0.000588
C12orf77	-0.043549025	0.323961	0.397065
C13orf15	-0.31815488	1.41E-13	1.40E-12
C13orf16	0.070219865	0.111467	0.156614
C13orf18	-0.175154978	6.44E-05	0.000169
C13orf1	0.131652687	0.002758	0.005423
C13orf23	0.105355897	0.016768	0.028379
C13orf26	-0.151595904	0.000557	0.001238
C13orf27	0.344739408	8.11E-16	1.13E-14
C13orf29	0.213182115	1.05E-06	3.67E-06
C13orf30	-0.274278571	2.43E-10	1.48E-09
C13orf31	-0.055435658	0.209136	0.272522
C13orf33	0.129614255	0.003212	0.006238
C13orf34	0.403220828	1.48E-21	4.94E-20
C13orf35	-0.061940816	0.16044	0.216257
C13orf36	-0.222132386	3.53E-07	1.34E-06
C13orf37	0.395480374	9.97E-21	2.89E-19
C13orf38	0.152945892	0.000496	0.001112
C13orf39	-0.143922004	0.001056	0.002235
C14orf101	0.137448625	0.001769	0.003597
C14orf102	-0.115641762	0.00862	0.015468
C14orf104	0.478159754	8.84E-31	1.10E-28
C14orf105	-0.075842648	0.085535	0.123745
C14orf106	0.27126885	3.88E-10	2.29E-09
C14orf109	0.136902544	0.001846	0.003743
C14orf115	-0.096529655	0.028495	0.045969
C14orf118	0.207073838	2.14E-06	7.12E-06
C14orf119	0.299657266	3.81E-12	3.02E-11
C14orf126	0.337137285	3.73E-15	4.74E-14
C14orf128	0.134052514	0.0023	0.004587
C14orf129	0.430150712	1.31E-24	6.77E-23
C14orf132	-0.294778147	8.76E-12	6.61E-11
C14orf135	0.141258153	0.001309	0.002726
C14orf138	0.113270648	0.010095	0.017909
C14orf139	-0.341938946	1.43E-15	1.93E-14
C14orf142	0.141922131	0.001241	0.002597
C14orf143	0.19750255	6.31E-06	1.95E-05
C14orf145	0.204308637	2.94E-06	9.57E-06
C14orf147	0.318666666	1.28E-13	1.28E-12
C14orf148	-0.136666029	0.00188	0.003806
C14orf149	0.16326417	0.000198	0.00048
C14orf153	0.286106942	3.70E-11	2.54E-10

C14orf156	0.370485238	3.36E-18	6.72E-17
C14orf159	-0.271892009	3.52E-10	2.09E-09
C14orf162	0.040713935	0.356485	0.431034
C14orf165	0.037544911	0.395181	0.470087
C14orf166B	-0.092162993	0.036539	0.057516
C14orf166	0.519727972	5.66E-37	2.26E-34
C14orf167	0.061436912	0.163876	0.220324
C14orf169	0.243454192	2.20E-08	9.97E-08
C14orf174	-0.095195754	0.030773	0.049259
C14orf176	-0.156211726	0.000373	0.000855
C14orf178	-0.180975064	3.61E-05	9.87E-05
C14orf179	0.134530083	0.002217	0.004436
C14orf180	-0.260784778	1.88E-09	9.96E-09
C14orf181	-0.150818674	0.000595	0.001316
C14orf182	-0.059171907	0.180006	0.23925
C14orf183	-0.017465617	0.692529	0.748514
C14orf184	0.024086761	0.585501	0.652123
C14orf19	0.094280859	0.032424	0.051629
C14orf1	0.140008429	0.001446	0.002988
C14orf21	0.064317873	0.14496	0.197873
C14orf23	0.165044044	0.000168	0.000413
C14orf28	-0.027895908	0.52762	0.599719
C14orf2	0.314510859	2.75E-13	2.61E-12
C14orf33	0.409658983	2.92E-22	1.06E-20
C14orf34	0.225295694	2.38E-07	9.23E-07
C14orf37	0.136799922	0.001861	0.00377
C14orf39	-0.080431367	0.068183	0.101123
C14orf43	-0.124209076	0.00476	0.008982
C14orf45	-0.106700964	0.015416	0.0263
C14orf48	-0.088688287	0.044248	0.068465
C14orf49	-0.188738371	1.62E-05	4.70E-05
C14orf4	-0.097339648	0.027184	0.044039
C14orf50	0.042632055	0.334263	0.40804
C14orf53	0.138310719	0.001654	0.003381
C14orf64	-0.33796982	3.16E-15	4.07E-14
C14orf68	0.044749082	0.310793	0.382851
C14orf70	0.034403478	0.435937	0.510457
C14orf72	0.012807448	0.771852	0.816501
C14orf73	-0.291122681	1.62E-11	1.17E-10
C14orf79	-0.045710034	0.300507	0.372014
C14orf80	0.17001946	0.000106	0.000268
C14orf86	-0.092359454	0.036139	0.056981
C14orf93	-0.079102231	0.072883	0.107274
C15orf17	-0.387750537	6.36E-20	1.63E-18

C15orf21	0.123546174	0.00499	0.009376
C15orf23	0.500146477	5.98E-34	1.39E-31
C15orf24	0.158904128	0.000294	0.000688
C15orf26	-0.177163276	5.28E-05	0.00014
C15orf27	-0.267400748	7.00E-10	3.98E-09
C15orf28	-0.231525462	1.07E-07	4.40E-07
C15orf29	0.146521223	0.000853	0.001835
C15orf2	-0.148196201	0.000742	0.001617
C15orf32	-0.028311326	0.521488	0.593492
C15orf33	-0.087716064	0.046636	0.071804
C15orf34	-0.357118923	6.17E-17	1.02E-15
C15orf37	-0.189225222	1.54E-05	4.49E-05
C15orf38	-0.153618244	0.000468	0.001054
C15orf39	-0.061838335	0.161134	0.217032
C15orf40	-0.021975101	0.618806	0.682148
C15orf41	0.222515844	3.37E-07	1.28E-06
C15orf42	0.395769331	9.29E-21	2.71E-19
C15orf43	-0.021785337	0.621839	0.684887
C15orf44	0.273819914	2.61E-10	1.58E-09
C15orf48	0.23413596	7.64E-08	3.20E-07
C15orf50	-0.083109805	0.059465	0.089381
C15orf51	-0.345677684	6.70E-16	9.48E-15
C15orf52	-0.245139256	1.74E-08	8.04E-08
C15orf53	-0.012209337	0.782232	0.824864
C15orf54	-0.092761974	0.035332	0.055832
C15orf55	-0.049428167	0.262858	0.331922
C15orf56	-0.269891588	4.79E-10	2.79E-09
C15orf57	-0.061789364	0.161467	0.217421
C15orf58	-0.026148726	0.553805	0.623455
C15orf59	-0.476385597	1.56E-30	1.89E-28
C15orf5	-0.068958348	0.118059	0.164788
C15orf60	0.105321417	0.016804	0.028435
C15orf61	0.129223875	0.003306	0.006407
C15orf62	-0.334061388	6.84E-15	8.30E-14
C15orf63	0.290415042	1.82E-11	1.31E-10
C16orf11	0.058933555	0.18177	0.241153
C16orf13	-0.019500817	0.658843	0.718046
C16orf3	-0.175266921	6.37E-05	0.000167
C16orf42	-0.071894366	0.103168	0.146187
C16orf45	-0.072767247	0.09904	0.141083
C16orf46	0.003626068	0.934576	0.94872
C16orf48	-0.321264976	7.90E-14	8.12E-13
C16orf52	-0.158008086	0.000319	0.00074
C16orf53	7.39E-05	0.998665	0.999165

C16orf54	-0.299517057	3.91E-12	3.09E-11
C16orf55	-0.137543402	0.001756	0.003573
C16orf57	0.102532056	0.019949	0.033242
C16orf58	-0.219000993	5.20E-07	1.92E-06
C16orf59	0.332055133	1.01E-14	1.19E-13
C16orf5	-0.389905418	3.81E-20	1.02E-18
C16orf61	0.34748068	4.63E-16	6.74E-15
C16orf62	-0.076531649	0.082722	0.12012
C16orf63	0.194532139	8.73E-06	2.64E-05
C16orf68	0.036244702	0.411763	0.486426
C16orf70	0.011817521	0.789053	0.83074
C16orf71	-0.279342134	1.10E-10	7.01E-10
C16orf72	0.052754994	0.232038	0.298128
C16orf73	0.147115829	0.000812	0.001755
C16orf74	0.087385912	0.047471	0.072926
C16orf75	0.22717264	1.88E-07	7.40E-07
C16orf78	-0.002149003	0.961198	0.970325
C16orf79	-0.245962193	1.56E-08	7.23E-08
C16orf7	-0.08171577	0.06388	0.095357
C16orf80	0.116086561	0.008366	0.015067
C16orf81	-0.188172913	1.72E-05	4.97E-05
C16orf82	-0.015906292	0.71876	0.771442
C16orf86	-0.327589256	2.39E-14	2.66E-13
C16orf87	0.282617369	6.51E-11	4.30E-10
C16orf88	0.307537147	9.62E-13	8.45E-12
C16orf89	-0.430237942	1.28E-24	6.64E-23
C16orf90	0.037770381	0.392347	0.467257
C16orf91	0.159357304	0.000283	0.000664
C16orf92	-0.000102985	0.99814	0.998889
C16orf93	-0.319050029	1.19E-13	1.20E-12
C17orf100	0.028278015	0.521978	0.59394
C17orf101	-0.129381743	0.003268	0.006336
C17orf102	-0.170554223	0.0001	0.000255
C17orf103	-0.451304084	3.30E-27	2.51E-25
C17orf104	0.116860194	0.007939	0.014369
C17orf105	-0.10614269	0.015965	0.027138
C17orf106	0.003254919	0.94126	0.953617
C17orf107	-0.185625	2.24E-05	6.35E-05
C17orf108	-0.425490898	4.63E-24	2.24E-22
C17orf28	-0.156558014	0.000362	0.000832
C17orf37	0.126115646	0.00415	0.007901
C17orf39	-0.141822004	0.001251	0.002617
C17orf42	0.215742326	7.72E-07	2.75E-06
C17orf44	-0.197728133	6.16E-06	1.90E-05

C17orf46	-0.150848024	0.000593	0.001313
C17orf47	-0.240789363	3.15E-08	1.40E-07
C17orf48	-0.00876954	0.842628	0.875433
C17orf49	0.057172755	0.195195	0.256513
C17orf50	-0.396178173	8.41E-21	2.47E-19
C17orf51	0.062014997	0.159939	0.215669
C17orf53	0.44418036	2.60E-26	1.76E-24
C17orf54	-0.20038178	4.59E-06	1.45E-05
C17orf55	-0.200831248	4.36E-06	1.38E-05
C17orf56	-0.271807953	3.57E-10	2.12E-09
C17orf57	-0.199944685	4.81E-06	1.51E-05
C17orf58	0.277082705	1.57E-10	9.80E-10
C17orf59	-0.120205049	0.006311	0.011631
C17orf60	-0.14061664	0.001378	0.002858
C17orf61	0.188989455	1.58E-05	4.59E-05
C17orf62	-0.014903234	0.735812	0.786072
C17orf63	0.058663561	0.183783	0.243436
C17orf64	0.076978434	0.080939	0.117856
C17orf65	-0.324396531	4.39E-14	4.71E-13
C17orf66	-0.157103391	0.000345	0.000796
C17orf67	-0.013210998	0.764872	0.810747
C17orf68	-0.24180966	2.75E-08	1.23E-07
C17orf69	-0.260553365	1.94E-09	1.03E-08
C17orf70	-0.143562395	0.001087	0.002298
C17orf71	0.196970333	6.69E-06	2.06E-05
C17orf72	-0.290643473	1.75E-11	1.26E-10
C17orf73	0.086016696	0.051069	0.077974
C17orf74	0.014638974	0.740326	0.789839
C17orf75	0.218452448	5.56E-07	2.04E-06
C17orf76	-0.130496463	0.003008	0.005872
C17orf77	0.060416092	0.171006	0.228756
C17orf78	0.033139257	0.452997	0.527252
C17orf79	0.181470248	3.43E-05	9.42E-05
C17orf80	0.147956897	0.000757	0.001646
C17orf81	0.148448268	0.000726	0.001586
C17orf82	-0.110994675	0.011717	0.020482
C17orf85	0.03780606	0.3919	0.466795
C17orf86	-0.189581169	1.48E-05	4.33E-05
C17orf87	-0.1901986	1.39E-05	4.07E-05
C17orf88	-0.122217214	0.005482	0.010229
C17orf89	0.14529634	0.000944	0.002014
C17orf90	-0.029009768	0.511261	0.583515
C17orf91	-0.188984682	1.58E-05	4.59E-05
C17orf93	0.12897649	0.003367	0.006515

C17orf95	0.056124087	0.203527	0.26615
C17orf96	0.248231534	1.14E-08	5.38E-08
C17orf97	-0.131449259	0.002801	0.005498
C17orf98	-0.025112573	0.569628	0.637855
C17orf99	-0.095158545	0.030839	0.049356
C18orf10	-0.047978626	0.27713	0.347634
C18orf16	-0.306753625	1.11E-12	9.63E-12
C18orf18	-0.157851255	0.000323	0.00075
C18orf19	0.337900373	3.21E-15	4.12E-14
C18orf1	-0.389438851	4.26E-20	1.13E-18
C18orf20	-0.094216359	0.032543	0.05181
C18orf21	0.158375976	0.000308	0.000719
C18orf22	0.080652944	0.067424	0.100116
C18orf25	0.039797462	0.367423	0.442067
C18orf26	0.007729169	0.861096	0.890777
C18orf2	0.169083197	0.000115	0.000291
C18orf32	0.159421192	0.000281	0.00066
C18orf34	-0.099254959	0.024288	0.039743
C18orf45	0.025322543	0.566404	0.635092
C18orf54	0.318817333	1.25E-13	1.25E-12
C18orf55	0.239053149	3.98E-08	1.74E-07
C18orf56	0.245983982	1.55E-08	7.21E-08
C18orf62	0.110828776	0.011844	0.020674
C18orf8	-0.039425359	0.371923	0.446782
C19orf10	0.248470675	1.10E-08	5.22E-08
C19orf12	0.08842134	0.044893	0.069383
C19orf18	-0.200015463	4.78E-06	1.50E-05
C19orf20	-0.038287494	0.385894	0.46063
C19orf21	0.168047448	0.000127	0.000319
C19orf22	0.067509349	0.126003	0.1745
C19orf23	0.004768921	0.914026	0.932786
C19orf24	0.026127014	0.554134	0.623791
C19orf25	-0.103233978	0.019113	0.03198
C19orf26	0.115777786	0.008541	0.015341
C19orf28	0.173234499	7.76E-05	0.000201
C19orf29	-0.129717345	0.003187	0.006194
C19orf2	0.130730533	0.002956	0.005781
C19orf30	0.002989457	0.946043	0.957395
C19orf33	0.069684564	0.114228	0.160145
C19orf34	-0.183402197	2.82E-05	7.84E-05
C19orf35	-0.242143445	2.62E-08	1.18E-07
C19orf36	-0.260953985	1.83E-09	9.74E-09
C19orf38	-0.096525017	0.028503	0.045978
C19orf39	-0.134870773	0.00216	0.004331

C19orf40	0.301925595	2.58E-12	2.10E-11
C19orf41	0.036385707	0.409945	0.484622
C19orf42	-0.054305066	0.218588	0.283048
C19orf43	0.044917662	0.308972	0.380866
C19orf44	-0.270673172	4.25E-10	2.50E-09
C19orf45	0.165858655	0.000156	0.000385
C19orf46	-0.144958177	0.00097	0.002068
C19orf47	0.138396189	0.001643	0.003359
C19orf48	0.391663407	2.50E-20	6.89E-19
C19orf50	0.064339197	0.144826	0.197718
C19orf51	-0.148135175	0.000746	0.001625
C19orf52	0.11791272	0.007391	0.013455
C19orf53	0.132463728	0.002595	0.005125
C19orf54	-0.083899933	0.057077	0.086141
C19orf55	-0.048109164	0.275824	0.346239
C19orf56	-0.077950558	0.077166	0.112988
C19orf57	-0.057997242	0.188821	0.249267
C19orf59	-0.179426207	4.22E-05	0.000114
C19orf60	-0.079558699	0.07124	0.105165
C19orf61	0.024875196	0.573283	0.641301
C19orf62	0.055160682	0.211407	0.275033
C19orf63	-0.045662179	0.301014	0.372595
C19orf66	-0.334661704	6.08E-15	7.46E-14
C19orf69	-0.138827647	0.001588	0.003255
C19orf6	-0.215796051	7.67E-07	2.74E-06
C19orf70	0.039209593	0.374548	0.449422
C19orf71	-0.207733708	1.99E-06	6.64E-06
C19orf73	-0.023940798	0.587777	0.654275
C19orf75	-0.050526055	0.25239	0.320606
C19orf76	-0.147853879	0.000763	0.001659
C19orf77	0.127334845	0.003798	0.007277
C1D	0.204462899	2.89E-06	9.42E-06
C1GALT1C1	0.189110944	1.56E-05	4.54E-05
C1GALT1	0.015679687	0.7226	0.774732
C1QA	-0.105181023	0.016951	0.028663
C1QBP	0.405591517	8.19E-22	2.81E-20
C1QB	-0.089784361	0.041681	0.064874
C1QC	-0.070213391	0.1115	0.15665
C1QL1	0.049942316	0.257919	0.326656
C1QL2	-0.221669054	3.74E-07	1.41E-06
C1QL3	-0.056906213	0.197289	0.258958
C1QL4	0.179246303	4.29E-05	0.000116
C1QTNF1	-0.077159534	0.080225	0.116961
C1QTNF2	-0.323695224	5.01E-14	5.31E-13

C1QTNF3	-0.188800063	1.61E-05	4.67E-05
C1QTNF4	-0.189635851	1.47E-05	4.31E-05
C1QTNF6	0.224495341	2.63E-07	1.01E-06
C1QTNF7	-0.48464741	1.08E-31	1.64E-29
C1QTNF8	-0.143955385	0.001053	0.002229
C1QTNF9B	-0.209880986	1.55E-06	5.26E-06
C1QTNF9	-0.156660762	0.000359	0.000825
C1RL	-0.025238396	0.567695	0.636226
C1R	-0.081096496	0.065925	0.098124
C1S	-0.011541474	0.793869	0.834922
C1orf100	-0.008001198	0.856259	0.886691
C1orf101	-0.417544324	3.81E-23	1.60E-21
C1orf103	0.271500063	3.74E-10	2.21E-09
C1orf104	-0.234575079	7.21E-08	3.03E-07
C1orf105	0.193389218	9.88E-06	2.96E-05
C1orf106	-0.03774685	0.392642	0.467512
C1orf107	0.171663241	9.03E-05	0.000231
C1orf109	0.199610446	5.00E-06	1.57E-05
C1orf110	-0.158371074	0.000308	0.000719
C1orf111	-0.14656426	0.00085	0.00183
C1orf112	0.384820383	1.27E-19	3.16E-18
C1orf113	-0.180571924	3.76E-05	0.000102
C1orf114	-0.057484601	0.192766	0.253771
C1orf115	-0.140297933	0.001413	0.002925
C1orf116	-0.416156496	5.48E-23	2.24E-21
C1orf122	0.118010007	0.007342	0.013374
C1orf123	-0.08153003	0.064488	0.096146
C1orf124	0.284950174	4.46E-11	3.02E-10
C1orf125	-0.070032013	0.11243	0.15789
C1orf126	-0.249270832	9.82E-09	4.71E-08
C1orf127	-0.321523236	7.53E-14	7.78E-13
C1orf128	-0.145992238	0.000891	0.001911
C1orf129	-0.230248787	1.27E-07	5.14E-07
C1orf130	-0.308355526	8.32E-13	7.38E-12
C1orf131	0.254809243	4.48E-09	2.24E-08
C1orf133	-0.34203813	1.40E-15	1.90E-14
C1orf135	0.493301803	6.14E-33	1.15E-30
C1orf141	-0.203521287	3.22E-06	1.04E-05
C1orf144	0.113744991	0.009783	0.017408
C1orf146	-0.106303522	0.015805	0.026897
C1orf14	-0.00011965	0.997839	0.998638
C1orf150	-0.288003849	2.71E-11	1.89E-10
C1orf151	0.12501397	0.004493	0.008509
C1orf152	-0.381441058	2.79E-19	6.63E-18

C1orf156	0.065947262	0.135026	0.185836
C1orf157	0.063352881	0.151101	0.205163
C1orf158	-0.242227811	2.59E-08	1.17E-07
C1orf159	-0.06654946	0.131491	0.18137
C1orf161	-0.020518889	0.642243	0.703036
C1orf162	-0.236544236	5.56E-08	2.38E-07
C1orf163	0.442947855	3.69E-26	2.43E-24
C1orf168	-0.251287358	7.39E-09	3.61E-08
C1orf170	-0.122753961	0.005279	0.009875
C1orf172	-0.068907317	0.118332	0.165123
C1orf173	-0.252122673	6.57E-09	3.22E-08
C1orf174	0.18009337	3.94E-05	0.000107
C1orf175	-0.421333187	1.40E-23	6.30E-22
C1orf177	-0.077360791	0.079438	0.11594
C1orf180	0.102858765	0.019556	0.032658
C1orf182	0.258353203	2.68E-09	1.39E-08
C1orf183	-0.343045157	1.14E-15	1.57E-14
C1orf185	-0.074528196	0.091113	0.130906
C1orf186	-0.413077056	1.22E-22	4.71E-21
C1orf187	0.083429526	0.058489	0.088066
C1orf189	-0.189331351	1.52E-05	4.44E-05
C1orf190	-0.3149909	2.52E-13	2.41E-12
C1orf192	-0.216894698	6.71E-07	2.42E-06
C1orf194	-0.203306321	3.30E-06	1.06E-05
C1orf198	-0.303909271	1.82E-12	1.53E-11
C1orf200	-0.172361449	8.44E-05	0.000217
C1orf201	-0.21499607	8.44E-07	3.00E-06
C1orf203	-0.188006054	1.75E-05	5.06E-05
C1orf204	-0.319322964	1.13E-13	1.14E-12
C1orf210	-0.209167036	1.68E-06	5.69E-06
C1orf212	0.181622559	3.38E-05	9.28E-05
C1orf213	-0.263637769	1.23E-09	6.73E-09
C1orf216	-0.063271676	0.151626	0.205737
C1orf21	-0.415275669	6.89E-23	2.77E-21
C1orf220	-0.082361262	0.061803	0.092602
C1orf223	-0.150192264	0.000627	0.001383
C1orf226	-0.110251388	0.012295	0.021402
C1orf227	-0.086203622	0.050564	0.077316
C1orf228	-0.275180768	2.12E-10	1.30E-09
C1orf229	-0.147491148	0.000787	0.001705
C1orf230	-0.176067059	5.89E-05	0.000155
C1orf25	0.038717663	0.380575	0.455178
C1orf26	0.0323577	0.463729	0.537596
C1orf27	0.099983486	0.023259	0.038237

C1orf31	0.398904433	4.32E-21	1.33E-19
C1orf35	-0.036538696	0.407978	0.482696
C1orf38	-0.148008951	0.000754	0.00164
C1orf43	0.260677155	1.91E-09	1.01E-08
C1orf49	-0.031174288	0.480244	0.554094
C1orf50	-0.025097117	0.569865	0.63805
C1orf51	-0.080568258	0.067713	0.100484
C1orf52	0.076313449	0.083605	0.121216
C1orf53	-0.094073865	0.032808	0.052186
C1orf54	-0.092860333	0.035138	0.055542
C1orf55	0.166437334	0.000148	0.000367
C1orf56	0.022357632	0.612712	0.676699
C1orf57	0.116325026	0.008232	0.014849
C1orf58	0.126187353	0.004129	0.007864
C1orf59	0.025570788	0.562604	0.631866
C1orf61	0.218725212	5.37E-07	1.97E-06
C1orf63	-0.335895969	4.77E-15	5.94E-14
C1orf64	0.048046329	0.276452	0.346941
C1orf65	-0.084718242	0.054688	0.082955
C1orf66	-0.10775759	0.014421	0.024768
C1orf68	0.044718919	0.31112	0.383136
C1orf69	-0.131831114	0.002722	0.005357
C1orf70	-0.219588068	4.84E-07	1.79E-06
C1orf74	0.292169512	1.36E-11	9.97E-11
C1orf77	0.022562934	0.609452	0.673843
C1orf83	-0.004577551	0.917464	0.935196
C1orf84	-0.083792746	0.057396	0.08659
C1orf85	0.172805007	8.09E-05	0.000208
C1orf86	-0.049643179	0.260784	0.329596
C1orf87	-0.231265513	1.11E-07	4.55E-07
C1orf88	-0.335886191	4.78E-15	5.95E-14
C1orf89	-0.23817848	4.48E-08	1.94E-07
C1orf91	-0.108827572	0.013472	0.023264
C1orf92	-0.261143607	1.78E-09	9.49E-09
C1orf93	-0.157765909	0.000326	0.000755
C1orf94	0.088576293	0.044518	0.068845
C1orf95	-0.029794563	0.499896	0.572899
C1orf96	0.140696363	0.001369	0.002841
C1orf97	0.008361583	0.84986	0.881389
C1orf9	0.140504815	0.00139	0.002881
C20orf103	-0.202125338	3.77E-06	1.21E-05
C20orf106	-0.126486589	0.00404	0.007708
C20orf107	-0.177555173	5.08E-05	0.000136
C20orf108	-0.257475375	3.05E-09	1.56E-08

C20orf111	0.221886812	3.64E-07	1.37E-06
C20orf112	-0.159038153	0.000291	0.000681
C20orf114	-0.104993778	0.017149	0.028963
C20orf117	-0.070616517	0.109455	0.154113
C20orf118	-0.077755771	0.07791	0.113927
C20orf11	0.103938514	0.018304	0.03075
C20orf123	-0.145963277	0.000893	0.001914
C20orf12	-0.277662874	1.43E-10	9.02E-10
C20orf132	-0.33049148	1.37E-14	1.58E-13
C20orf134	-0.126166377	0.004135	0.007875
C20orf135	-0.177631558	5.04E-05	0.000135
C20orf141	0.302644616	2.27E-12	1.87E-11
C20orf144	-0.048658885	0.270368	0.340245
C20orf151	-0.192887523	1.04E-05	3.11E-05
C20orf152	-0.058315578	0.186401	0.246464
C20orf160	-0.32473946	4.11E-14	4.43E-13
C20orf165	-0.118882291	0.006915	0.012648
C20orf166	-0.082418528	0.061621	0.092365
C20orf173	-0.008212948	0.852498	0.883437
C20orf177	0.037269891	0.398655	0.473318
C20orf185	-0.017426249	0.693187	0.749144
C20orf186	0.083607144	0.057952	0.087317
C20orf191	0.112137214	0.010876	0.019165
C20orf194	-0.435098136	3.36E-25	1.94E-23
C20orf195	-0.13463809	0.002199	0.004403
C20orf196	0.061545858	0.163129	0.219407
C20orf197	-0.125184477	0.004439	0.00841
C20orf199	0.255900186	3.83E-09	1.94E-08
C20orf200	-0.32309324	5.61E-14	5.88E-13
C20orf201	-0.102425603	0.020079	0.033447
C20orf202	-0.102717043	0.019726	0.032921
C20orf203	-0.119829107	0.006478	0.011911
C20orf20	0.323384943	5.31E-14	5.60E-13
C20orf24	0.425514879	4.60E-24	2.23E-22
C20orf26	-0.200701266	4.42E-06	1.40E-05
C20orf27	0.113151923	0.010174	0.018037
C20orf29	0.028414975	0.519964	0.592191
C20orf30	0.153717817	0.000464	0.001046
C20orf3	0.005435377	0.902068	0.923412
C20orf43	0.046462191	0.292615	0.363867
C20orf46	-0.116934679	0.007899	0.014301
C20orf4	-0.001159791	0.979053	0.98457
C20orf54	-0.0801755	0.069067	0.102291
C20orf56	-0.319117321	1.18E-13	1.18E-12

C20orf70	0.108778744	0.013514	0.023331
C20orf71	0.013822438	0.754331	0.801785
C20orf72	0.16270531	0.000209	0.000504
C20orf7	0.216854808	6.75E-07	2.43E-06
C20orf85	-0.202095368	3.78E-06	1.21E-05
C20orf94	-0.003531119	0.936285	0.949973
C20orf96	-0.197292753	6.46E-06	1.99E-05
C21orf119	-0.006476202	0.883438	0.908622
C21orf121	-0.135298901	0.00209	0.004204
C21orf122	-0.22638156	2.07E-07	8.13E-07
C21orf125	0.190792842	1.30E-05	3.84E-05
C21orf128	-0.118616854	0.007043	0.012865
C21orf129	-0.183686139	2.74E-05	7.64E-05
C21orf130	-0.1111391	0.011608	0.020317
C21orf131	0.011268629	0.798636	0.838929
C21orf15	-0.284247899	5.00E-11	3.35E-10
C21orf29	-0.263809957	1.20E-09	6.58E-09
C21orf2	-0.48093502	3.62E-31	4.92E-29
C21orf33	-0.138496135	0.00163	0.003335
C21orf34	-0.355289505	9.09E-17	1.45E-15
C21orf45	0.37708128	7.59E-19	1.68E-17
C21orf49	-0.269397195	5.17E-10	2.99E-09
C21orf54	-0.015204511	0.730676	0.781797
C21orf56	0.018060064	0.682624	0.739527
C21orf57	0.017525988	0.691521	0.747667
C21orf58	-0.192562028	1.08E-05	3.22E-05
C21orf59	-0.00866466	0.844486	0.876862
C21orf62	-0.287134452	3.12E-11	2.17E-10
C21orf63	-0.273447064	2.77E-10	1.67E-09
C21orf67	-0.215476613	7.97E-07	2.84E-06
C21orf70	0.09881161	0.024933	0.040713
C21orf71	-0.166855679	0.000142	0.000353
C21orf7	-0.077272366	0.079783	0.116376
C21orf81	-0.195697813	7.69E-06	2.34E-05
C21orf82	-0.178673295	4.55E-05	0.000122
C21orf84	0.146401895	0.000861	0.001852
C21orf88	-0.00038008	0.993135	0.994906
C21orf90	-0.1769668	5.39E-05	0.000143
C21orf91	-0.097588635	0.026791	0.043492
C21orf94	-0.066870296	0.129636	0.179084
C21orf96	-0.171486104	9.18E-05	0.000235
C21orf99	0.047253403	0.284465	0.355324
C22orf13	-0.220958085	4.09E-07	1.53E-06
C22orf15	-0.236815105	5.37E-08	2.30E-07

C22orf23	-0.168240755	0.000125	0.000313
C22orf24	-0.061919795	0.160582	0.21642
C22orf25	-0.216134272	7.36E-07	2.64E-06
C22orf26	-0.1251168	0.00446	0.008449
C22orf27	0.001025078	0.981486	0.98622
C22orf28	0.137667675	0.001739	0.003542
C22orf29	-0.265914688	8.75E-10	4.91E-09
C22orf30	-0.079450334	0.071627	0.105706
C22orf31	-0.125616354	0.004303	0.008171
C22orf32	-0.132655492	0.002557	0.005059
C22orf33	-0.114793519	0.009124	0.016309
C22orf34	-0.165653226	0.000159	0.000392
C22orf36	-0.383683525	1.65E-19	4.06E-18
C22orf39	-0.16104509	0.000243	0.000577
C22orf40	-0.182487491	3.10E-05	8.56E-05
C22orf41	-0.042004635	0.341432	0.415623
C22orf42	-0.089735452	0.041792	0.065033
C22orf43	-0.153616378	0.000468	0.001054
C22orf45	-0.270555791	4.33E-10	2.54E-09
C22orf46	-0.234909226	6.90E-08	2.91E-07
C22orf9	-0.037287634	0.39843	0.473079
C2CD2L	-0.293674914	1.05E-11	7.86E-11
C2CD2	-0.255852705	3.85E-09	1.95E-08
C2CD3	-0.060522418	0.170253	0.227903
C2CD4A	0.127021174	0.003886	0.007435
C2CD4B	-0.142410787	0.001193	0.002503
C2CD4C	-0.190723667	1.31E-05	3.87E-05
C2CD4D	0.105549637	0.016567	0.028072
C2orf14	0.012057328	0.784877	0.827171
C2orf15	0.027739222	0.529942	0.601674
C2orf16	0.065134032	0.139916	0.191735
C2orf18	0.083269893	0.058974	0.088744
C2orf24	-0.216851093	6.75E-07	2.43E-06
C2orf27A	0.046032385	0.297108	0.36849
C2orf28	0.129714142	0.003188	0.006195
C2orf29	0.222718097	3.29E-07	1.25E-06
C2orf34	-0.182461765	3.10E-05	8.58E-05
C2orf39	-0.187537732	1.84E-05	5.29E-05
C2orf3	0.255942031	3.80E-09	1.92E-08
C2orf40	-0.386785757	7.99E-20	2.03E-18
C2orf42	-0.144638416	0.000996	0.002119
C2orf43	0.232922155	8.96E-08	3.72E-07
C2orf44	0.167470332	0.000134	0.000335
C2orf47	0.355828885	8.11E-17	1.32E-15

C2orf48	0.081729805	0.063834	0.095295
C2orf49	0.407294476	5.33E-22	1.87E-20
C2orf50	-0.137170157	0.001808	0.003672
C2orf51	0.110849883	0.011828	0.02065
C2orf52	-0.05451693	0.216794	0.281144
C2orf53	-0.005561237	0.899812	0.921763
C2orf54	-0.277665002	1.43E-10	9.02E-10
C2orf55	-0.285469568	4.10E-11	2.79E-10
C2orf56	0.020740122	0.638659	0.699825
C2orf57	-0.153195892	0.000485	0.00109
C2orf58	-0.391430967	2.65E-20	7.25E-19
C2orf60	0.0371011	0.400795	0.47538
C2orf61	-0.044814192	0.310089	0.382125
C2orf62	-0.22304316	3.16E-07	1.20E-06
C2orf63	-0.342716304	1.22E-15	1.67E-14
C2orf64	0.038324287	0.385437	0.460139
C2orf65	-0.171574058	9.11E-05	0.000233
C2orf66	-0.208885548	1.74E-06	5.87E-06
C2orf67	-0.222109002	3.54E-07	1.34E-06
C2orf68	-0.159845994	0.00027	0.000638
C2orf69	0.278326699	1.29E-10	8.17E-10
C2orf70	-0.03117031	0.4803	0.554104
C2orf71	-0.20495441	2.74E-06	8.93E-06
C2orf72	-0.029834103	0.499327	0.572312
C2orf73	-0.272467939	3.22E-10	1.92E-09
C2orf74	-0.153133702	0.000488	0.001096
C2orf76	0.190478814	1.35E-05	3.96E-05
C2orf77	-0.201157536	4.20E-06	1.33E-05
C2orf78	0.156608136	0.000361	0.000829
C2orf79	0.069547903	0.114941	0.161055
C2orf7	0.096539407	0.028479	0.045951
C2orf80	0.1003865	0.022705	0.037399
C2orf81	-0.232991304	8.88E-08	3.69E-07
C2orf82	0.166372044	0.000149	0.000368
C2orf83	0.096975499	0.027767	0.044907
C2orf84	0.052993613	0.22993	0.295877
C2orf85	-0.250949754	7.75E-09	3.77E-08
C2orf86	-0.207268992	2.10E-06	6.97E-06
C2orf88	0.033113728	0.453345	0.527596
C2orf89	-0.195178016	8.14E-06	2.47E-05
C2	-0.129510304	0.003237	0.006283
C3AR1	-0.112546685	0.010588	0.018698
C3P1	0.176408652	5.69E-05	0.00015
C3orf10	0.07057238	0.109677	0.154387

C3orf14	0.001726404	0.968824	0.976445
C3orf15	-0.345229249	7.34E-16	1.03E-14
C3orf16	-0.146019729	0.000889	0.001907
C3orf17	0.13732376	0.001786	0.003631
C3orf18	-0.279923753	1.00E-10	6.45E-10
C3orf19	-0.154792266	0.000423	0.00096
C3orf1	0.202891751	3.46E-06	1.11E-05
C3orf20	0.003250475	0.94134	0.953649
C3orf21	0.14147509	0.001286	0.002684
C3orf22	-0.035817679	0.417298	0.491976
C3orf23	0.033146235	0.452902	0.527172
C3orf24	-0.019972061	0.651138	0.710966
C3orf26	0.371239148	2.84E-18	5.74E-17
C3orf27	-0.060331504	0.171607	0.229452
C3orf30	0.033243871	0.451571	0.525899
C3orf31	-0.084352725	0.055745	0.084347
C3orf32	-0.12387728	0.004874	0.009177
C3orf33	0.111049301	0.011676	0.020422
C3orf34	-0.069149672	0.11704	0.163583
C3orf35	-0.235007307	6.82E-08	2.88E-07
C3orf36	-0.208296082	1.86E-06	6.26E-06
C3orf37	0.141423197	0.001292	0.002694
C3orf38	0.191760686	1.18E-05	3.49E-05
C3orf39	-0.069765579	0.113806	0.159621
C3orf42	-0.353744032	1.26E-16	1.97E-15
C3orf43	0.006816682	0.877357	0.904182
C3orf45	-0.05398747	0.221297	0.286075
C3orf47	-0.336092521	4.59E-15	5.73E-14
C3orf48	-0.188986011	1.58E-05	4.59E-05
C3orf49	-0.054519652	0.216771	0.281144
C3orf50	-0.159765926	0.000272	0.000642
C3orf51	-0.049698167	0.260256	0.329074
C3orf52	-0.161351703	0.000236	0.000563
C3orf54	-0.229072331	1.47E-07	5.90E-07
C3orf55	-0.062223829	0.158534	0.214006
C3orf57	-0.002372971	0.957158	0.967174
C3orf58	-0.089884678	0.041452	0.064556
C3orf59	0.065423362	0.138161	0.189655
C3orf62	-0.42606266	3.97E-24	1.95E-22
C3orf63	-0.013862658	0.753639	0.80122
C3orf64	-0.099936169	0.023324	0.03833
C3orf65	-0.004513529	0.918614	0.936169
C3orf66	0.042709403	0.333386	0.407118
C3orf67	0.117814212	0.00744	0.013533

C3orf70	-0.121344435	0.005829	0.010809
C3orf71	-0.216049989	7.44E-07	2.66E-06
C3orf72	0.092318071	0.036223	0.057104
C3orf74	-0.042122883	0.340073	0.414222
C3orf75	0.099195348	0.024374	0.039868
C3orf77	0.024807156	0.574332	0.642009
C3orf79	-0.02931555	0.506817	0.579501
C3	-0.224908054	2.50E-07	9.66E-07
C4A	-0.414828444	7.74E-23	3.08E-21
C4BPA	-0.293778936	1.04E-11	7.73E-11
C4BPB	-0.015799753	0.720564	0.772964
C4orf10	-0.181040944	3.59E-05	9.81E-05
C4orf12	-0.234071787	7.71E-08	3.22E-07
C4orf14	0.158189694	0.000313	0.000729
C4orf17	0.040260302	0.361873	0.436414
C4orf19	-0.231646242	1.06E-07	4.34E-07
C4orf21	0.064114958	0.146235	0.199341
C4orf22	-0.158953223	0.000293	0.000685
C4orf23	-0.261041762	1.81E-09	9.63E-09
C4orf26	0.21398742	9.52E-07	3.36E-06
C4orf27	0.123643611	0.004956	0.009318
C4orf29	0.002287228	0.958704	0.968394
C4orf31	-0.334050808	6.85E-15	8.31E-14
C4orf32	0.062552142	0.156345	0.211393
C4orf33	-0.022639242	0.608243	0.672766
C4orf34	0.109542247	0.01287	0.022314
C4orf35	0.034360645	0.436509	0.51092
C4orf36	0.005922495	0.893341	0.916497
C4orf37	-0.19158632	1.20E-05	3.55E-05
C4orf38	-0.106928178	0.015197	0.025973
C4orf39	-0.195519947	7.84E-06	2.38E-05
C4orf3	0.022112017	0.616622	0.680152
C4orf40	0.047556833	0.28138	0.352043
C4orf41	-0.0876342	0.046842	0.072082
C4orf42	-0.005343532	0.903715	0.924624
C4orf43	0.403292919	1.46E-21	4.87E-20
C4orf44	-0.239296134	3.86E-08	1.69E-07
C4orf45	-0.049975403	0.257603	0.326318
C4orf46	0.346670137	5.47E-16	7.88E-15
C4orf47	-0.096963867	0.027786	0.04493
C4orf48	0.087764722	0.046514	0.071638
C4orf49	-0.055921841	0.205164	0.267974
C4orf50	0.000710529	0.987166	0.990437
C4orf51	0.094094973	0.032769	0.052128

C4orf52	0.211791185	1.24E-06	4.27E-06
C4orf6	0.143965063	0.001052	0.002228
C4orf7	-0.212708604	1.11E-06	3.86E-06
C5AR1	-0.127145792	0.003851	0.007371
C5orf13	-0.035323918	0.423752	0.498379
C5orf15	0.175728224	6.09E-05	0.00016
C5orf20	-0.292324139	1.32E-11	9.73E-11
C5orf22	0.180903366	3.64E-05	9.94E-05
C5orf23	-0.151810521	0.000547	0.001217
C5orf24	0.100883281	0.022039	0.036413
C5orf25	-0.158494397	0.000305	0.000712
C5orf27	-0.051643265	0.242039	0.309288
C5orf28	0.218198887	5.73E-07	2.10E-06
C5orf30	0.196255035	7.24E-06	2.21E-05
C5orf32	-0.151443847	0.000564	0.001253
C5orf33	0.161792491	0.000227	0.000543
C5orf34	0.290530521	1.78E-11	1.28E-10
C5orf35	0.067968126	0.123444	0.171479
C5orf36	-0.025171134	0.568728	0.637097
C5orf38	-0.400723887	2.76E-21	8.77E-20
C5orf39	-0.169935667	0.000106	0.00027
C5orf40	-0.030158733	0.494668	0.567917
C5orf41	-0.338384824	2.91E-15	3.77E-14
C5orf42	-0.246100281	1.53E-08	7.10E-08
C5orf43	0.291563271	1.50E-11	1.10E-10
C5orf44	0.047876689	0.278153	0.348704
C5orf45	-0.354120926	1.16E-16	1.83E-15
C5orf46	0.325803271	3.36E-14	3.67E-13
C5orf47	-0.043044472	0.329604	0.403017
C5orf48	0.012747259	0.772895	0.817431
C5orf49	-0.30110248	2.97E-12	2.40E-11
C5orf4	-0.159223114	0.000286	0.000671
C5orf51	0.023129442	0.600498	0.665857
C5orf52	-0.0412164	0.350576	0.425172
C5orf53	-0.412646826	1.36E-22	5.18E-21
C5orf54	-0.083292636	0.058905	0.088653
C5orf55	-0.085245203	0.053194	0.080895
C5orf56	-0.287359587	3.01E-11	2.09E-10
C5orf58	-0.196330253	7.18E-06	2.19E-05
C5orf60	-0.053480236	0.225674	0.290941
C5orf62	-0.052433359	0.234901	0.301361
C5	-0.047668095	0.280255	0.350898
C6orf103	-0.262472302	1.46E-09	7.91E-09
C6orf105	-0.058570599	0.18448	0.244246

C6orf106	-0.117413961	0.007646	0.013878
C6orf108	0.076385384	0.083313	0.120864
C6orf10	0.027551724	0.532728	0.60418
C6orf114	-0.082197613	0.062324	0.093307
C6orf115	0.230161471	1.28E-07	5.19E-07
C6orf118	-0.236971738	5.26E-08	2.25E-07
C6orf120	0.12389989	0.004866	0.009166
C6orf122	-0.008802643	0.842042	0.874917
C6orf123	-0.305793723	1.31E-12	1.12E-11
C6orf124	-0.332527149	9.22E-15	1.09E-13
C6orf125	0.099958668	0.023293	0.038288
C6orf126	0.068102461	0.122703	0.17065
C6orf127	0.069524387	0.115064	0.161205
C6orf129	0.334362145	6.45E-15	7.88E-14
C6orf130	0.047323101	0.283755	0.354588
C6orf132	-0.047859983	0.278321	0.348854
C6orf134	-0.14161688	0.001272	0.002656
C6orf136	0.080632396	0.067494	0.100198
C6orf138	-0.269247364	5.29E-10	3.06E-09
C6orf141	-0.036653972	0.4065	0.481289
C6orf142	-0.031506275	0.475579	0.54925
C6orf145	-0.205191925	2.66E-06	8.71E-06
C6orf146	-0.002686057	0.951512	0.962345
C6orf147	-0.146056975	0.000886	0.001902
C6orf150	0.293471732	1.09E-11	8.11E-11
C6orf153	0.353544977	1.31E-16	2.05E-15
C6orf154	-0.063288755	0.151516	0.205656
C6orf155	-0.34270887	1.22E-15	1.67E-14
C6orf15	0.060228798	0.172339	0.230369
C6orf162	0.079942527	0.069881	0.103337
C6orf163	-0.192991786	1.03E-05	3.08E-05
C6orf164	-0.302345436	2.39E-12	1.96E-11
C6orf165	-0.151682328	0.000553	0.00123
C6orf167	0.197015194	6.66E-06	2.05E-05
C6orf168	-0.196007733	7.43E-06	2.27E-05
C6orf170	-0.17799986	4.86E-05	0.00013
C6orf174	-0.277786015	1.41E-10	8.86E-10
C6orf176	0.281429795	7.87E-11	5.15E-10
C6orf182	0.220323513	4.42E-07	1.65E-06
C6orf186	-0.113318435	0.010063	0.017859
C6orf191	0.050105631	0.256364	0.324995
C6orf192	-0.051000538	0.247957	0.3158
C6orf195	0.132649179	0.002559	0.005061
C6orf1	-0.138615888	0.001614	0.003305

C6orf201	-0.312292868	4.11E-13	3.81E-12
C6orf203	-0.144809888	0.000982	0.002091
C6orf204	-0.098170694	0.025893	0.042152
C6orf208	-0.087305726	0.047675	0.073201
C6orf211	0.027555088	0.532678	0.604163
C6orf217	-0.267953672	6.43E-10	3.68E-09
C6orf218	0.225496003	2.32E-07	9.01E-07
C6orf221	-0.038436732	0.384043	0.458667
C6orf222	-0.173555422	7.52E-05	0.000195
C6orf223	-0.025372869	0.565633	0.634484
C6orf225	-0.168921141	0.000117	0.000295
C6orf226	-0.124746524	0.00458	0.008662
C6orf227	-0.138941554	0.001574	0.003227
C6orf25	-0.174672919	6.75E-05	0.000176
C6orf26	-0.129435343	0.003255	0.006314
C6orf27	-0.042181871	0.339397	0.413492
C6orf35	0.040990318	0.353227	0.427845
C6orf41	-0.271374823	3.82E-10	2.25E-09
C6orf47	-0.190752447	1.31E-05	3.86E-05
C6orf48	0.06456179	0.143438	0.19605
C6orf52	0.07662677	0.08234	0.119643
C6orf57	0.107829142	0.014356	0.024667
C6orf58	-0.095437393	0.030349	0.04867
C6orf59	-0.179576525	4.15E-05	0.000112
C6orf62	-0.021932403	0.619488	0.682749
C6orf64	-0.076971932	0.080965	0.117876
C6orf70	-0.282456132	6.68E-11	4.41E-10
C6orf72	-0.030679606	0.487242	0.560646
C6orf81	-0.078248196	0.07604	0.111551
C6orf89	-0.184314684	2.57E-05	7.20E-05
C6orf94	0.00416671	0.924848	0.941379
C6orf97	-0.286332551	3.56E-11	2.45E-10
C6	-0.287909835	2.75E-11	1.92E-10
C7orf10	0.10898574	0.013337	0.023052
C7orf11	0.311699572	4.57E-13	4.20E-12
C7orf13	-0.010754862	0.807633	0.846201
C7orf16	-0.086534665	0.049682	0.076077
C7orf23	-0.424903588	5.42E-24	2.59E-22
C7orf25	0.174598307	6.80E-05	0.000177
C7orf26	-0.069666316	0.114323	0.160256
C7orf27	-0.096498037	0.028547	0.046043
C7orf28A	0.197187136	6.53E-06	2.01E-05
C7orf28B	0.228748865	1.54E-07	6.13E-07
C7orf29	0.012683536	0.773999	0.818382

C7orf30	0.136438093	0.001914	0.00387
C7orf31	-0.140190793	0.001425	0.002949
C7orf33	0.021474025	0.626829	0.689508
C7orf34	-0.091366301	0.038197	0.059877
C7orf36	0.185794204	2.20E-05	6.25E-05
C7orf40	0.132182661	0.00265	0.00523
C7orf41	-0.336186408	4.50E-15	5.64E-14
C7orf42	-0.038803572	0.379518	0.454262
C7orf43	-0.062081814	0.159488	0.215134
C7orf44	0.223420385	3.01E-07	1.15E-06
C7orf45	-0.14592063	0.000896	0.001921
C7orf46	-0.185473035	2.28E-05	6.44E-05
C7orf47	0.133209607	0.002452	0.004866
C7orf49	0.326566918	2.91E-14	3.20E-13
C7orf4	-0.117824965	0.007435	0.013526
C7orf50	-0.002031785	0.963313	0.971772
C7orf51	0.000535775	0.990323	0.992856
C7orf52	0.000943975	0.98295	0.987247
C7orf53	-0.118851672	0.00693	0.012673
C7orf54	-0.176480445	5.65E-05	0.000149
C7orf55	-0.018182812	0.680585	0.737759
C7orf57	-0.243531386	2.17E-08	9.87E-08
C7orf58	-0.248992874	1.02E-08	4.89E-08
C7orf59	0.031523713	0.475335	0.549079
C7orf60	-0.066444079	0.132104	0.182166
C7orf61	0.095478879	0.030277	0.04857
C7orf63	-0.235360739	6.51E-08	2.75E-07
C7orf64	-0.001587891	0.971324	0.978373
C7orf65	0.063343444	0.151162	0.205232
C7orf66	0.043981054	0.319179	0.391829
C7orf68	0.379075467	4.81E-19	1.10E-17
C7orf69	-0.00020003	0.996387	0.997535
C7orf70	0.267957572	6.43E-10	3.68E-09
C7orf71	-0.009084349	0.837057	0.870595
C7orf72	-0.039876768	0.366468	0.441184
C7	-0.371056021	2.96E-18	5.96E-17
C8A	0.086075542	0.050909	0.077755
C8B	-0.071914547	0.103071	0.14607
C8G	0.063417663	0.150682	0.204678
C8ORFK29	-0.038598697	0.382041	0.456658
C8orf12	-0.105827644	0.016282	0.027627
C8orf22	-0.050456492	0.253044	0.321378
C8orf30A	0.175933141	5.96E-05	0.000157
C8orf31	-0.053719926	0.223598	0.288695

C8orf33	0.313165813	3.51E-13	3.29E-12
C8orf34	-0.35247172	1.64E-16	2.54E-15
C8orf37	0.084866372	0.054265	0.08242
C8orf38	0.165189356	0.000166	0.000408
C8orf39	0.006910668	0.875679	0.903012
C8orf40	-0.002815208	0.949184	0.960379
C8orf41	0.132894887	0.002512	0.004974
C8orf42	-0.252813542	5.95E-09	2.94E-08
C8orf44	-0.043658095	0.322749	0.395774
C8orf45	0.053303672	0.227212	0.292661
C8orf46	-0.183851291	2.69E-05	7.52E-05
C8orf47	0.070492704	0.11008	0.154851
C8orf48	-0.225141553	2.43E-07	9.40E-07
C8orf4	-0.000549893	0.990068	0.9927
C8orf51	0.067423358	0.126488	0.175122
C8orf55	-0.026505664	0.548404	0.618735
C8orf56	0.056336409	0.20182	0.26428
C8orf58	-0.118548289	0.007076	0.012921
C8orf59	0.339823915	2.19E-15	2.88E-14
C8orf71	0.052792615	0.231705	0.297796
C8orf73	-0.146895216	0.000827	0.001785
C8orf74	0.123551022	0.004989	0.009374
C8orf75	0.198773659	5.48E-06	1.71E-05
C8orf76	0.486718267	5.49E-32	8.57E-30
C8orf77	-0.158229987	0.000312	0.000727
C8orf79	-0.332818302	8.72E-15	1.04E-13
C8orf80	-0.082416428	0.061628	0.092368
C8orf83	0.125360047	0.004383	0.008313
C8orf84	-0.237752765	4.74E-08	2.04E-07
C8orf85	-0.324801692	4.06E-14	4.38E-13
C8orf86	0.030469084	0.490236	0.56354
C9orf100	0.378226718	5.84E-19	1.32E-17
C9orf102	-0.061567705	0.162979	0.219232
C9orf103	-0.201241194	4.17E-06	1.32E-05
C9orf106	-0.22151115	3.82E-07	1.43E-06
C9orf109	0.039398057	0.372255	0.447055
C9orf110	0.035842146	0.41698	0.491774
C9orf114	-0.072138755	0.101998	0.144697
C9orf116	-0.072645816	0.099606	0.141774
C9orf117	-0.261381866	1.72E-09	9.19E-09
C9orf119	0.154761348	0.000424	0.000963
C9orf11	-0.178850618	4.47E-05	0.00012
C9orf122	-0.052942371	0.230382	0.296362
C9orf123	0.089672796	0.041936	0.065236

C9orf125	-0.045328252	0.304566	0.37634
C9orf128	-0.36668537	7.79E-18	1.48E-16
C9orf129	0.031644493	0.473644	0.547395
C9orf130	-0.209106763	1.69E-06	5.73E-06
C9orf131	-0.262631802	1.43E-09	7.74E-09
C9orf135	-0.213303714	1.03E-06	3.62E-06
C9orf139	-0.338057809	3.11E-15	4.01E-14
C9orf140	0.445414056	1.82E-26	1.25E-24
C9orf142	-0.079178116	0.072607	0.106941
C9orf144B	-0.216960352	6.66E-07	2.41E-06
C9orf144	-0.154831434	0.000421	0.000957
C9orf150	-0.17363401	7.47E-05	0.000194
C9orf152	-0.23937386	3.82E-08	1.67E-07
C9orf153	-0.005804559	0.895453	0.918192
C9orf156	-0.167997158	0.000128	0.00032
C9orf163	-0.13526396	0.002096	0.004213
C9orf167	-0.106877355	0.015246	0.026041
C9orf169	0.086197042	0.050582	0.077338
C9orf16	-0.029308997	0.506912	0.579544
C9orf170	-0.083185311	0.059233	0.089066
C9orf171	-0.214274647	9.20E-07	3.25E-06
C9orf172	0.040926741	0.353975	0.428621
C9orf173	-0.175414045	6.28E-05	0.000165
C9orf21	0.10894744	0.01337	0.023107
C9orf23	0.21037218	1.46E-06	4.98E-06
C9orf24	-0.238849466	4.09E-08	1.78E-07
C9orf25	-0.064991017	0.14079	0.1928
C9orf27	0.095052355	0.031027	0.049629
C9orf30	0.463365276	8.98E-29	8.20E-27
C9orf37	-0.113724612	0.009796	0.01743
C9orf3	-0.059050922	0.1809	0.240158
C9orf40	0.35060697	2.43E-16	3.64E-15
C9orf41	0.214856436	8.58E-07	3.04E-06
C9orf43	-0.051967904	0.239088	0.305908
C9orf44	-0.21155523	1.27E-06	4.38E-06
C9orf45	-0.405050549	9.38E-22	3.20E-20
C9orf46	0.218515715	5.51E-07	2.02E-06
C9orf47	-0.080102325	0.069322	0.102638
C9orf4	0.051739438	0.241162	0.308305
C9orf50	-0.199497099	5.06E-06	1.59E-05
C9orf53	0.016872824	0.70246	0.757773
C9orf57	0.027697183	0.530566	0.60211
C9orf5	-0.166329634	0.00015	0.00037
C9orf64	0.107034144	0.015096	0.025827

C9orf66	-0.285673453	3.97E-11	2.71E-10
C9orf68	-0.337477775	3.49E-15	4.45E-14
C9orf69	0.137917258	0.001705	0.00348
C9orf6	0.098714921	0.025076	0.040929
C9orf70	-0.170799361	9.81E-05	0.00025
C9orf71	-0.125868259	0.004225	0.008032
C9orf72	-0.147972942	0.000756	0.001644
C9orf78	0.014910612	0.735686	0.785979
C9orf79	0.052510806	0.23421	0.300667
C9orf7	-0.282546004	6.58E-11	4.35E-10
C9orf80	0.155829495	0.000386	0.000883
C9orf82	0.165948494	0.000155	0.000382
C9orf84	0.029084465	0.510174	0.582573
C9orf85	0.173321918	7.70E-05	0.000199
C9orf86	-0.081415882	0.064864	0.09666
C9orf89	0.023819524	0.58967	0.655816
C9orf91	-0.144331722	0.001021	0.00217
C9orf93	-0.149124713	0.000686	0.001505
C9orf95	-0.121454296	0.005784	0.010734
C9orf96	-0.297234023	5.77E-12	4.47E-11
C9orf98	-0.234092485	7.69E-08	3.22E-07
C9orf9	-0.247689556	1.22E-08	5.78E-08
C9	0.111856883	0.011077	0.01948
CA10	-0.334949509	5.74E-15	7.08E-14
CA11	-0.131668537	0.002755	0.005418
CA12	0.178975595	4.41E-05	0.000119
CA13	-0.265400065	9.46E-10	5.28E-09
CA14	-0.050860415	0.24926	0.317197
CA1	-0.18768005	1.81E-05	5.22E-05
CA2	-0.111999122	0.010975	0.019314
CA3	-0.411136563	2.00E-22	7.40E-21
CA4	-0.232283076	9.73E-08	4.02E-07
CA5A	0.167697431	0.000132	0.000328
CA5BP	-0.099493649	0.023946	0.039226
CA5B	-0.337788606	3.28E-15	4.20E-14
CA6	0.098413288	0.025526	0.041602
CA7	-0.069186803	0.116843	0.163342
CA8	-0.051080679	0.247214	0.314974
CA9	0.060774169	0.168479	0.225829
CAB39L	-0.198557577	5.62E-06	1.75E-05
CAB39	0.027283084	0.536732	0.607624
CABC1	-0.277957608	1.37E-10	8.63E-10
CABIN1	-0.358663176	4.44E-17	7.49E-16
CABLES1	-0.267492483	6.90E-10	3.93E-09

CABLES2	-0.032777179	0.457951	0.531872
CABP1	-0.011173207	0.800305	0.840108
CABP2	0.005037472	0.909205	0.928862
CABP4	0.026513488	0.548286	0.618642
CABP5	-0.038854824	0.378889	0.453661
CABP7	-0.142848315	0.001152	0.002424
CABYR	0.264078871	1.15E-09	6.34E-09
CACHD1	-0.356332074	7.29E-17	1.19E-15
CACNA1A	-0.022397341	0.612081	0.676124
CACNA1B	-0.091478204	0.037961	0.059563
CACNA1C	-0.313194753	3.49E-13	3.27E-12
CACNA1D	-0.298839181	4.39E-12	3.44E-11
CACNA1E	0.054943161	0.213217	0.277098
CACNA1F	-0.381311274	2.87E-19	6.81E-18
CACNA1G	-0.326907687	2.72E-14	3.01E-13
CACNA1H	-0.128462109	0.003497	0.006744
CACNA1I	-0.148561261	0.00072	0.001572
CACNA1S	-0.065639266	0.136862	0.18804
CACNA2D1	0.056122171	0.203543	0.266153
CACNA2D2	-0.387604147	6.58E-20	1.68E-18
CACNA2D3	-0.264232869	1.13E-09	6.21E-09
CACNA2D4	-0.198634482	5.57E-06	1.73E-05
CACNB1	-0.508055468	3.79E-35	1.12E-32
CACNB2	0.005545305	0.900098	0.921966
CACNB3	-0.177560223	5.08E-05	0.000135
CACNB4	-0.305748215	1.32E-12	1.13E-11
CACNG1	-0.066728135	0.130455	0.180104
CACNG2	0.019283463	0.66241	0.721186
CACNG3	0.030272376	0.493043	0.566311
CACNG4	-0.169266394	0.000113	0.000286
CACNG5	-0.0138013	0.754695	0.801979
CACNG6	-0.156967938	0.000349	0.000805
CACNG7	0.164294241	0.000181	0.00044
CACNG8	0.133540323	0.002391	0.004756
CACYBP	0.427230515	2.90E-24	1.46E-22
CADM1	-0.187755337	1.80E-05	5.18E-05
CADM2	0.026223666	0.552669	0.622527
CADM3	-0.340734102	1.82E-15	2.43E-14
CADM4	-0.108441186	0.013808	0.023806
CADPS2	-0.248548645	1.09E-08	5.17E-08
CADPS	-0.21185143	1.23E-06	4.24E-06
CAD	0.19356552	9.69E-06	2.91E-05
CAGE1	0.166130441	0.000152	0.000376
CALB1	0.139672921	0.001485	0.003063

CALB2	0.034269669	0.437725	0.512037
CALCA	0.174735814	6.71E-05	0.000175
CALCB	0.17658355	5.59E-05	0.000148
CALCOCO1	-0.484380507	1.18E-31	1.78E-29
CALCOCO2	-0.208737015	1.77E-06	5.97E-06
CALCRL	-0.135788011	0.002013	0.004059
CALCR	0.099820624	0.023485	0.038556
CALD1	-0.007065416	0.872919	0.900955
CALHM1	-0.083679729	0.057734	0.087035
CALHM2	-0.075447477	0.087182	0.125883
CALHM3	0.055583716	0.207921	0.27115
CALM1	-0.024403965	0.580571	0.64757
CALM2	0.159876143	0.00027	0.000636
CALM3	0.036094624	0.413704	0.488343
CALML3	0.125284957	0.004407	0.008354
CALML4	-0.222022047	3.58E-07	1.35E-06
CALML5	-0.064951628	0.141032	0.193078
CALML6	-0.222473856	3.39E-07	1.28E-06
CALN1	-0.086347438	0.050179	0.076804
CALR3	0.049187853	0.265188	0.334464
CALR	0.248729727	1.06E-08	5.05E-08
CALU	0.404319852	1.13E-21	3.80E-20
CALY	0.121134132	0.005915	0.010957
CAMK1D	-0.312903298	3.68E-13	3.44E-12
CAMK1G	-0.176643998	5.56E-05	0.000147
CAMK1	-0.240336522	3.35E-08	1.48E-07
CAMK2A	-0.262070952	1.55E-09	8.37E-09
CAMK2B	-0.085730968	0.051847	0.079045
CAMK2D	-0.279511277	1.07E-10	6.85E-10
CAMK2G	-0.175247806	6.38E-05	0.000167
CAMK2N1	0.00940475	0.831394	0.865741
CAMK2N2	0.033431111	0.449025	0.5233
CAMK4	-0.190242386	1.38E-05	4.06E-05
CAMKK1	-0.30650442	1.16E-12	1.00E-11
CAMKK2	0.131825026	0.002723	0.005359
CAMKV	-0.037769578	0.392357	0.467257
CAMLG	0.009651952	0.827031	0.862322
CAMP	-0.218790566	5.33E-07	1.96E-06
CAMSAP1L1	0.077758427	0.0779	0.113921
CAMSAP1	-0.009431436	0.830923	0.865386
CAMTA1	-0.183647757	2.75E-05	7.67E-05
CAMTA2	-0.335246536	5.42E-15	6.69E-14
CAND1	0.249856544	9.04E-09	4.36E-08
CAND2	-0.220436094	4.36E-07	1.63E-06

CANT1	0.212682333	1.11E-06	3.87E-06
CANX	0.110163077	0.012365	0.021511
CAP1	0.070628469	0.109395	0.15406
CAP2	0.018324921	0.678228	0.735642
CAPG	-0.118982735	0.006868	0.012577
CAPN10	-0.166106043	0.000153	0.000377
CAPN11	-0.224114051	2.76E-07	1.06E-06
CAPN12	-0.196515924	7.03E-06	2.15E-05
CAPN13	-0.216962384	6.66E-07	2.41E-06
CAPN14	0.054468183	0.217206	0.281551
CAPN1	-0.03772258	0.392947	0.467819
CAPN2	-0.127265299	0.003817	0.007309
CAPN3	-0.522188531	2.29E-37	9.93E-35
CAPN5	-0.026657045	0.546122	0.616682
CAPN6	-0.139604208	0.001493	0.003078
CAPN7	-0.048971465	0.267299	0.336873
CAPN8	-0.326314929	3.05E-14	3.35E-13
CAPN9	-0.118915214	0.0069	0.012627
CAPNS1	-0.012154152	0.783192	0.825743
CAPNS2	-0.171072985	9.55E-05	0.000244
CAPRIN1	0.202116753	3.77E-06	1.21E-05
CAPRIN2	-0.131755872	0.002737	0.005385
CAPS2	-0.10068769	0.0223	0.0368
CAPSL	-0.181621175	3.38E-05	9.28E-05
CAPS	-0.312729457	3.80E-13	3.54E-12
CAPZA1	0.289802418	2.01E-11	1.44E-10
CAPZA2	0.19664349	6.93E-06	2.13E-05
CAPZA3	0.031780595	0.471743	0.545482
CAPZB	0.003541882	0.936092	0.949825
CARD10	-0.034632052	0.432892	0.507546
CARD11	-0.150569772	0.000608	0.001342
CARD14	0.215430568	8.01E-07	2.85E-06
CARD16	-0.232194159	9.85E-08	4.06E-07
CARD17	0.026636227	0.546435	0.616896
CARD18	0.099647463	0.023728	0.038901
CARD6	-0.141555057	0.001278	0.002668
CARD8	-0.359643708	3.60E-17	6.18E-16
CARD9	-0.29533387	7.97E-12	6.06E-11
CARHSP1	0.134881318	0.002158	0.004329
CARKD	-0.089141076	0.043172	0.066924
CARM1	0.183797159	2.71E-05	7.56E-05
CARNS1	-0.256445541	3.54E-09	1.80E-08
CARS2	-0.050237631	0.255111	0.323613
CARS	0.284377896	4.90E-11	3.29E-10

CARTPT	-0.072685615	0.09942	0.141559
CASC1	-0.302897861	2.17E-12	1.80E-11
CASC2	-0.27894902	1.17E-10	7.45E-10
CASC3	-0.03098445	0.482923	0.556645
CASC4	0.054956109	0.213109	0.276976
CASC5	0.438963273	1.14E-25	7.10E-24
CASD1	-0.179770379	4.07E-05	0.00011
CASKIN1	0.102360067	0.020159	0.033567
CASKIN2	-0.299125337	4.18E-12	3.29E-11
CASK	-0.015268961	0.729579	0.780916
CASP10	-0.290473157	1.80E-11	1.30E-10
CASP12	-0.287088231	3.15E-11	2.18E-10
CASP14	0.117674285	0.007512	0.013651
CASP1	-0.260420977	1.98E-09	1.05E-08
CASP2	0.032840303	0.457085	0.531114
CASP3	0.248723724	1.06E-08	5.05E-08
CASP4	-0.011082181	0.801898	0.84125
CASP5	0.094736154	0.031593	0.050446
CASP6	0.189662046	1.47E-05	4.30E-05
CASP7	0.020384252	0.644428	0.705065
CASP8AP2	-0.019399178	0.66051	0.719487
CASP8	-0.089576752	0.042157	0.065519
CASP9	0.034399847	0.435985	0.510484
CASQ1	-0.380341473	3.59E-19	8.36E-18
CASQ2	-0.373317952	1.78E-18	3.69E-17
CASR	-0.318660668	1.28E-13	1.28E-12
CASS4	-0.348818889	3.51E-16	5.20E-15
CAST	-0.122776361	0.00527	0.009863
CASZ1	-0.398057676	5.31E-21	1.61E-19
CATSPER1	0.04622476	0.295092	0.366422
CATSPER2P1	0.13582619	0.002007	0.004048
CATSPER2	-0.294193387	9.67E-12	7.26E-11
CATSPER3	-0.078160163	0.076372	0.11198
CATSPER4	0.014030276	0.750758	0.798837
CATSPERB	0.050294447	0.254574	0.323115
CATSPERG	-0.28804148	2.69E-11	1.88E-10
CAT	-0.290991355	1.65E-11	1.20E-10
CAV1	-0.068297319	0.121633	0.169304
CAV2	-0.067625895	0.125349	0.173678
CAV3	-0.36546036	1.02E-17	1.91E-16
CBARA1	0.091089437	0.038789	0.060723
CBFA2T2	-0.162499231	0.000213	0.000513
CBFA2T3	-0.394934923	1.14E-20	3.26E-19
CBFB	0.081470507	0.064683	0.096405

CBLB	-0.080132477	0.069217	0.102498
CBLC	0.141211358	0.001314	0.002735
CBLL1	0.184309855	2.57E-05	7.20E-05
CBLN1	-0.081822844	0.063531	0.094883
CBLN2	0.014800828	0.73756	0.787645
CBLN3	-0.204594721	2.85E-06	9.28E-06
CBLN4	-0.159207837	0.000286	0.000672
CBL	-0.063505081	0.150119	0.203982
CBR1	0.117994399	0.007349	0.013387
CBR3	0.077219151	0.079991	0.116654
CBR4	-0.199926387	4.82E-06	1.52E-05
CBS	0.17894096	4.43E-05	0.000119
CBWD1	0.109099817	0.01324	0.022899
CBWD2	-0.010475539	0.812535	0.850267
CBWD3	0.145167467	0.000954	0.002035
CBWD5	0.101037734	0.021836	0.03611
CBWD6	0.091134676	0.038691	0.060594
CBX1	0.187560743	1.83E-05	5.28E-05
CBX2	0.242689416	2.44E-08	1.10E-07
CBX3	0.408489264	3.94E-22	1.42E-20
CBX4	0.015711499	0.722061	0.774237
CBX5	0.091080869	0.038807	0.060747
CBX6	-0.18339461	2.82E-05	7.85E-05
CBX7	-0.610103406	7.88E-54	7.87E-50
CBX8	0.04090489	0.354232	0.428855
CBY1	-0.081794301	0.063624	0.094996
CC2D1A	-0.239438398	3.78E-08	1.66E-07
CC2D1B	-0.257678118	2.96E-09	1.52E-08
CC2D2A	-0.198226483	5.83E-06	1.81E-05
CC2D2B	-0.330838636	1.28E-14	1.48E-13
CCAR1	0.253590791	5.33E-09	2.65E-08
CCBE1	-0.022596436	0.608921	0.673442
CCBL1	-0.081803194	0.063595	0.09496
CCBL2	-0.025295855	0.566813	0.635416
CCBP2	-0.026677024	0.545821	0.616411
CCDC101	-0.328767961	1.91E-14	2.16E-13
CCDC102A	-0.259298516	2.34E-09	1.22E-08
CCDC102B	-0.042741638	0.333021	0.406737
CCDC103	-0.049395849	0.26317	0.332275
CCDC104	-0.057794588	0.190373	0.251001
CCDC105	0.038727727	0.380451	0.455104
CCDC106	-0.160304162	0.00026	0.000614
CCDC107	-0.091698424	0.037498	0.058888
CCDC108	-0.229295434	1.43E-07	5.75E-07

CCDC109A	0.186654466	2.02E-05	5.76E-05
CCDC109B	0.053640285	0.224286	0.289434
CCDC110	0.103064828	0.019311	0.032282
CCDC111	-0.055180783	0.211241	0.27487
CCDC112	0.047730655	0.279624	0.350151
CCDC113	-0.14714258	0.00081	0.001752
CCDC114	-0.350169837	2.66E-16	3.97E-15
CCDC115	-0.318585708	1.30E-13	1.30E-12
CCDC116	-0.180631718	3.74E-05	0.000102
CCDC117	0.04039612	0.360255	0.435039
CCDC11	-0.185998347	2.16E-05	6.13E-05
CCDC120	-0.252182049	6.51E-09	3.20E-08
CCDC121	-0.16230119	0.000217	0.000521
CCDC122	0.009456375	0.830483	0.86513
CCDC123	0.080685726	0.067312	0.099965
CCDC124	0.052230437	0.23672	0.303383
CCDC125	-0.111204518	0.011559	0.020245
CCDC126	0.150500484	0.000611	0.00135
CCDC127	0.052361503	0.235544	0.302108
CCDC129	-0.156455617	0.000365	0.000839
CCDC12	-0.113638398	0.009852	0.017524
CCDC130	-0.278830717	1.19E-10	7.58E-10
CCDC132	0.120842374	0.006037	0.011164
CCDC134	0.049204093	0.26503	0.334307
CCDC135	-0.212668045	1.11E-06	3.87E-06
CCDC136	-0.219470318	4.91E-07	1.82E-06
CCDC137	0.139099136	0.001554	0.003193
CCDC138	0.363418826	1.59E-17	2.87E-16
CCDC13	-0.294417891	9.31E-12	7.00E-11
CCDC140	0.009138365	0.836101	0.869737
CCDC141	-0.268974274	5.51E-10	3.18E-09
CCDC142	-0.033925315	0.442346	0.51663
CCDC144A	-0.143361043	0.001105	0.002333
CCDC144B	-0.160568101	0.000253	0.000601
CCDC144C	-0.019227301	0.663332	0.722033
CCDC144NL	0.087873086	0.046243	0.071259
CCDC146	-0.401796112	2.11E-21	6.88E-20
CCDC147	-0.278526737	1.25E-10	7.94E-10
CCDC148	-0.165534439	0.000161	0.000396
CCDC149	-0.101331584	0.021453	0.035538
CCDC14	-0.208697825	1.78E-06	5.99E-06
CCDC150	0.176637302	5.57E-05	0.000147
CCDC151	-0.143304429	0.00111	0.002343
CCDC152	-0.313489959	3.31E-13	3.12E-12

CCDC153	-0.263648437	1.23E-09	6.72E-09
CCDC154	-0.205182434	2.67E-06	8.71E-06
CCDC155	-0.110279222	0.012273	0.021365
CCDC157	-0.288201219	2.62E-11	1.83E-10
CCDC158	-0.067943109	0.123583	0.171599
CCDC159	-0.425637018	4.45E-24	2.16E-22
CCDC15	0.045628566	0.30137	0.37299
CCDC160	-0.077986057	0.077031	0.112798
CCDC163P	0.056249713	0.202516	0.265082
CCDC17	-0.273657031	2.68E-10	1.62E-09
CCDC18	0.075656944	0.086306	0.124689
CCDC19	-0.219080893	5.15E-07	1.90E-06
CCDC21	0.115777823	0.008541	0.015341
CCDC22	-0.106032572	0.016075	0.027301
CCDC23	0.047840465	0.278518	0.349029
CCDC24	-0.23954103	3.73E-08	1.64E-07
CCDC25	-0.023837359	0.589392	0.655579
CCDC27	-0.08748326	0.047223	0.072602
CCDC28A	-0.299492486	3.92E-12	3.10E-11
CCDC28B	-0.085147728	0.053468	0.081284
CCDC30	-0.390160095	3.59E-20	9.61E-19
CCDC33	-0.221687252	3.73E-07	1.40E-06
CCDC34	0.378032964	6.10E-19	1.37E-17
CCDC36	-0.127650665	0.003712	0.007124
CCDC37	-0.286942461	3.22E-11	2.23E-10
CCDC38	-0.062401784	0.157345	0.212572
CCDC39	-0.325589495	3.50E-14	3.81E-13
CCDC3	-0.133781207	0.002348	0.004674
CCDC40	-0.2889036	2.34E-11	1.65E-10
CCDC41	-0.004406082	0.920545	0.937715
CCDC42B	-0.276278057	1.78E-10	1.11E-09
CCDC42	-0.240246137	3.39E-08	1.50E-07
CCDC43	0.303750992	1.87E-12	1.57E-11
CCDC45	-0.133631197	0.002375	0.004727
CCDC46	-0.348423133	3.81E-16	5.61E-15
CCDC47	-0.023771015	0.590428	0.656477
CCDC48	-0.458469761	3.95E-28	3.25E-26
CCDC50	-0.046668254	0.290478	0.361591
CCDC51	0.287831344	2.79E-11	1.94E-10
CCDC52	0.081822007	0.063534	0.094883
CCDC53	-0.051256352	0.24559	0.313244
CCDC54	-0.033270401	0.45121	0.52557
CCDC55	-0.067649397	0.125218	0.17352
CCDC56	0.030766967	0.486002	0.559445

CCDC57	-0.328785933	1.90E-14	2.16E-13
CCDC58	0.44825644	8.02E-27	5.75E-25
CCDC59	0.412831976	1.30E-22	4.96E-21
CCDC60	-0.227468273	1.81E-07	7.15E-07
CCDC61	-0.278815271	1.19E-10	7.60E-10
CCDC62	-0.033079741	0.453809	0.52789
CCDC63	0.035720859	0.418559	0.493317
CCDC64B	-0.397689448	5.81E-21	1.75E-19
CCDC64	-0.006758499	0.878395	0.904926
CCDC65	-0.205551585	2.55E-06	8.39E-06
CCDC66	-0.300396217	3.36E-12	2.69E-11
CCDC67	-0.04150076	0.347259	0.421625
CCDC68	-0.165690655	0.000159	0.00039
CCDC69	-0.286840136	3.28E-11	2.26E-10
CCDC6	0.030085031	0.495724	0.568904
CCDC70	0.105659916	0.016454	0.027906
CCDC71	-0.034278399	0.437608	0.511934
CCDC72	0.076096936	0.084488	0.122398
CCDC73	-0.097432329	0.027037	0.04384
CCDC74A	-0.108131431	0.014083	0.024244
CCDC74B	-0.130169732	0.003082	0.006006
CCDC75	0.091707991	0.037478	0.058865
CCDC76	-0.004381868	0.92098	0.938111
CCDC77	0.322960612	5.75E-14	6.02E-13
CCDC78	-0.240878769	3.12E-08	1.38E-07
CCDC79	-0.087727193	0.046608	0.071766
CCDC7	0.013810567	0.754535	0.801917
CCDC80	0.035423849	0.422441	0.496984
CCDC81	-0.289488803	2.12E-11	1.51E-10
CCDC82	-0.112590386	0.010558	0.018652
CCDC83	0.027370642	0.535425	0.606591
CCDC84	-0.298754953	4.45E-12	3.49E-11
CCDC85A	-0.265501383	9.31E-10	5.21E-09
CCDC85B	0.083249309	0.059037	0.088825
CCDC85C	0.165350892	0.000164	0.000402
CCDC86	0.381519115	2.74E-19	6.53E-18
CCDC87	0.029352995	0.506274	0.57898
CCDC88A	-0.001595256	0.971191	0.978288
CCDC88B	-0.334358023	6.45E-15	7.89E-14
CCDC88C	-0.192527196	1.08E-05	3.23E-05
CCDC89	-0.226168216	2.13E-07	8.33E-07
CCDC8	-0.229795357	1.34E-07	5.42E-07
CCDC90A	0.022038897	0.617788	0.681176
CCDC90B	0.228433252	1.60E-07	6.36E-07

CCDC91	0.174030013	7.18E-05	0.000187
CCDC92	-0.302492347	2.33E-12	1.92E-11
CCDC93	-0.260461708	1.97E-09	1.04E-08
CCDC94	-0.143675878	0.001077	0.002278
CCDC96	-0.272643964	3.14E-10	1.88E-09
CCDC97	-0.17666694	5.55E-05	0.000147
CCDC99	0.41829966	3.13E-23	1.34E-21
CCDC9	-0.097517287	0.026903	0.043655
CCHCR1	-0.112954537	0.010308	0.018252
CCIN	0.082702862	0.060727	0.091168
CCKAR	-0.034682698	0.432219	0.506906
CCKBR	-0.089659425	0.041967	0.065263
CCK	0.053067676	0.229279	0.295114
CCL11	0.093273695	0.034329	0.054347
CCL13	-0.110923388	0.011772	0.020562
CCL14-CCL15	-0.017020385	0.699983	0.755508
CCL14	-0.311957228	4.36E-13	4.03E-12
CCL15	0.024161895	0.584332	0.651111
CCL16	-0.318238977	1.39E-13	1.38E-12
CCL17	-0.290549115	1.78E-11	1.28E-10
CCL18	-0.00615742	0.889137	0.913121
CCL19	-0.245178141	1.73E-08	8.00E-08
CCL1	0.033126117	0.453176	0.52743
CCL20	0.217753785	6.05E-07	2.20E-06
CCL21	0.034242078	0.438094	0.512353
CCL22	-0.179513825	4.18E-05	0.000113
CCL23	-0.195230073	8.09E-06	2.46E-05
CCL24	0.081176627	0.065658	0.097785
CCL25	0.025793461	0.559206	0.62858
CCL26	0.287718972	2.84E-11	1.98E-10
CCL27	-0.097262312	0.027307	0.04422
CCL28	-0.238491452	4.29E-08	1.87E-07
CCL2	-0.059066347	0.180786	0.240039
CCL3L1	0.03506926	0.427103	0.501758
CCL3L3	-0.119346612	0.006698	0.012289
CCL3	0.067473274	0.126206	0.174769
CCL4L2	0.063274844	0.151606	0.205736
CCL4	0.0996863	0.023674	0.038824
CCL5	-0.01574825	0.721437	0.773717
CCL7	0.199579286	5.01E-06	1.57E-05
CCL8	0.166153113	0.000152	0.000375
CCM2	0.024973126	0.571773	0.6399
CCNA1	-0.035701928	0.418806	0.49352
CCNA2	0.561194523	4.57E-44	7.03E-41

CCNB1IP1	0.224432085	2.65E-07	1.02E-06
CCNB1	0.598637682	2.17E-51	1.09E-47
CCNB2	0.565093446	8.73E-45	1.59E-41
CCNB3	-0.034433814	0.435532	0.510073
CCNC	0.212448639	1.14E-06	3.97E-06
CCND1	-0.037410301	0.396879	0.471822
CCND2	-0.229220874	1.45E-07	5.80E-07
CCND3	-0.194142219	9.11E-06	2.74E-05
CCNDBP1	-0.157030957	0.000347	0.000801
CCNE1	0.331316058	1.17E-14	1.36E-13
CCNE2	0.34315991	1.12E-15	1.53E-14
CCNF	0.32637213	3.02E-14	3.31E-13
CCNG1	-0.072029523	0.10252	0.145351
CCNG2	0.126577364	0.004014	0.007661
CCNH	-0.037080381	0.401059	0.475636
CCNI2	0.063080806	0.152867	0.207209
CCNI	0.129016749	0.003357	0.006499
CCNJL	-0.241940421	2.70E-08	1.21E-07
CCNJ	0.232532805	9.42E-08	3.89E-07
CCNK	0.194303766	8.95E-06	2.70E-05
CCNL1	-0.277166697	1.55E-10	9.68E-10
CCNL2	-0.307445897	9.78E-13	8.58E-12
CCNO	-0.030798897	0.485549	0.559117
CCNT1	0.118917093	0.006899	0.012626
CCNT2	-0.167719099	0.000131	0.000328
CCNYL1	0.055419734	0.209267	0.272622
CCNY	0.153135665	0.000488	0.001096
CCPG1	-0.154037011	0.000451	0.00102
CCR10	0.094159053	0.03265	0.051967
CCR1	-0.043049232	0.32955	0.402996
CCR2	-0.181045781	3.58E-05	9.80E-05
CCR3	-0.044021162	0.318738	0.391359
CCR4	-0.262036931	1.56E-09	8.41E-09
CCR5	-0.139930716	0.001455	0.003005
CCR6	-0.377647349	6.67E-19	1.49E-17
CCR7	-0.282379594	6.76E-11	4.46E-10
CCR8	-0.079304502	0.072151	0.106392
CCR9	-0.164494004	0.000177	0.000433
CCRL1	-0.169305755	0.000113	0.000285
CCRL2	-0.202851086	3.47E-06	1.12E-05
CCRN4L	0.426415418	3.61E-24	1.80E-22
CCS	-0.10483929	0.017314	0.029203
CCT2	0.399333492	3.88E-21	1.21E-19
CCT3	0.468259718	2.00E-29	2.00E-27

CCT4	0.46226084	1.26E-28	1.13E-26
CCT5	0.458373997	4.06E-28	3.33E-26
CCT6A	0.450749111	3.88E-27	2.92E-25
CCT6B	-0.317301788	1.65E-13	1.62E-12
CCT6P1	-0.121575695	0.005735	0.010653
CCT7	0.48686943	5.22E-32	8.22E-30
CCT8L2	-0.102586133	0.019883	0.033154
CCT8	0.392802649	1.90E-20	5.27E-19
CD101	-0.263026276	1.35E-09	7.32E-09
CD109	0.206603513	2.26E-06	7.49E-06
CD14	-0.076040797	0.084718	0.122692
CD151	-0.101222416	0.021595	0.035752
CD160	-0.188687941	1.63E-05	4.72E-05
CD163L1	0.063945485	0.147307	0.200583
CD163	-0.043600807	0.323385	0.396505
CD164L2	-0.31283593	3.72E-13	3.48E-12
CD164	0.027055818	0.540131	0.611022
CD177	0.070565162	0.109714	0.154412
CD180	-0.13164677	0.00276	0.005424
CD19	-0.139702354	0.001482	0.003057
CD1A	-0.331632042	1.10E-14	1.28E-13
CD1B	-0.346541881	5.61E-16	8.06E-15
CD1C	-0.398807399	4.42E-21	1.36E-19
CD1D	-0.216019195	7.46E-07	2.67E-06
CD1E	-0.423984964	6.93E-24	3.25E-22
CD200R1L	-0.07774445	0.077954	0.113966
CD200R1	-0.259934016	2.13E-09	1.12E-08
CD200	-0.009792502	0.824553	0.860276
CD207	-0.383834012	1.60E-19	3.94E-18
CD209	0.007480385	0.865524	0.894478
CD226	-0.089234521	0.042952	0.066636
CD22	-0.375455362	1.10E-18	2.37E-17
CD244	-0.090502663	0.040067	0.062562
CD247	-0.129014671	0.003357	0.006499
CD248	-0.015044714	0.733398	0.784122
CD24	-0.035925476	0.415897	0.490642
CD274	0.092672985	0.035509	0.056072
CD276	0.195324183	8.01E-06	2.43E-05
CD27	-0.155019828	0.000414	0.000943
CD28	-0.187498961	1.85E-05	5.31E-05
CD2AP	0.195891049	7.53E-06	2.29E-05
CD2BP2	-0.037983379	0.389681	0.464485
CD2	-0.135404027	0.002073	0.004173
CD300A	-0.024625867	0.577134	0.644707

CD300C	-0.198467132	5.67E-06	1.76E-05
CD300E	-0.016433706	0.709849	0.764053
CD300LB	-0.272240038	3.34E-10	1.99E-09
CD300LD	0.017080829	0.698969	0.754568
CD300LF	-0.258749701	2.53E-09	1.31E-08
CD300LG	-0.374951959	1.23E-18	2.64E-17
CD302	-0.423497203	7.90E-24	3.66E-22
CD320	0.077479902	0.078974	0.115331
CD33	-0.270979285	4.05E-10	2.39E-09
CD34	-0.299741887	3.76E-12	2.98E-11
CD36	-0.174164598	7.09E-05	0.000185
CD37	-0.339342652	2.41E-15	3.15E-14
CD38	0.023306821	0.597706	0.66324
CD3D	-0.059571186	0.17708	0.235981
CD3EAP	0.111387162	0.011422	0.020039
CD3E	-0.160552957	0.000254	0.000601
CD3G	-0.062844851	0.154412	0.209062
CD40LG	-0.35993227	3.38E-17	5.85E-16
CD40	-0.20501975	2.72E-06	8.87E-06
CD44	-0.184891083	2.42E-05	6.81E-05
CD46	0.093791815	0.033337	0.052931
CD47	-0.238121081	4.51E-08	1.95E-07
CD48	-0.178431225	4.66E-05	0.000125
CD4	-0.269837364	4.83E-10	2.81E-09
CD52	-0.256234241	3.65E-09	1.85E-08
CD53	-0.138050805	0.001688	0.003448
CD55	-0.080887729	0.066627	0.099059
CD58	-0.010249956	0.8165	0.85361
CD59	-0.185149502	2.36E-05	6.64E-05
CD5L	-0.147649784	0.000777	0.001685
CD5	-0.250822454	7.89E-09	3.84E-08
CD63	-0.023932897	0.5879	0.654284
CD68	-0.097205104	0.027398	0.044355
CD69	-0.22685476	1.95E-07	7.69E-07
CD6	-0.241608819	2.82E-08	1.26E-07
CD70	0.067182391	0.127852	0.176839
CD72	-0.066813683	0.129962	0.179507
CD74	-0.383530792	1.71E-19	4.18E-18
CD79A	-0.105715526	0.016397	0.027817
CD79B	-0.141442849	0.00129	0.00269
CD7	0.002011589	0.963677	0.971993
CD80	-0.163759818	0.00019	0.00046
CD81	-0.373961122	1.54E-18	3.25E-17
CD82	-0.347235213	4.87E-16	7.07E-15

CD83	-0.397723127	5.77E-21	1.74E-19
CD84	-0.167881123	0.000129	0.000323
CD86	-0.118431431	0.007133	0.013018
CD8A	0.01726412	0.695899	0.751831
CD8B	0.024341237	0.581545	0.648475
CD93	-0.179714076	4.10E-05	0.000111
CD96	-0.179538859	4.17E-05	0.000113
CD97	-0.253102975	5.71E-09	2.83E-08
CD99L2	-0.023244983	0.598678	0.664131
CD99	0.145364243	0.000938	0.002004
CD9	-0.103085296	0.019287	0.032246
CDADC1	-0.205486308	2.57E-06	8.45E-06
CDAN1	-0.189568144	1.49E-05	4.34E-05
CDA	0.218991514	5.20E-07	1.92E-06
CDC123	0.387953279	6.06E-20	1.57E-18
CDC14A	-0.190026484	1.41E-05	4.15E-05
CDC14B	-0.183918736	2.67E-05	7.48E-05
CDC14C	-0.035105771	0.426622	0.501223
CDC16	-0.035443456	0.422184	0.496711
CDC20B	-0.080723072	0.067185	0.099791
CDC20	0.53314987	3.67E-39	2.62E-36
CDC23	0.066710874	0.130555	0.180217
CDC25A	0.461807405	1.44E-28	1.29E-26
CDC25B	-0.034487624	0.434815	0.509442
CDC25C	0.500856046	4.68E-34	1.10E-31
CDC26	0.118939915	0.006888	0.01261
CDC27	0.344422028	8.65E-16	1.20E-14
CDC34	0.186220442	2.11E-05	6.01E-05
CDC37L1	0.055429728	0.209185	0.272568
CDC37	0.000560646	0.989873	0.992555
CDC40	-0.059059823	0.180834	0.240087
CDC42BPA	-0.247753902	1.21E-08	5.73E-08
CDC42BPB	0.050863164	0.249235	0.317185
CDC42BPG	-0.290266799	1.86E-11	1.34E-10
CDC42EP1	-0.244034916	2.03E-08	9.27E-08
CDC42EP2	0.12875771	0.003422	0.006612
CDC42EP3	-0.22632583	2.09E-07	8.18E-07
CDC42EP4	-0.248286372	1.13E-08	5.34E-08
CDC42EP5	0.074340192	0.091934	0.131878
CDC42SE1	0.088072232	0.045749	0.070563
CDC42SE2	-0.113436168	0.009985	0.017734
CDC42	0.081948699	0.063124	0.094382
CDC45	0.460568905	2.10E-28	1.79E-26
CDC5L	0.27962685	1.05E-10	6.74E-10

CDC6	0.494597122	3.97E-33	7.68E-31
CDC73	0.304404305	1.67E-12	1.41E-11
CDC7	0.288931237	2.32E-11	1.64E-10
CDCA2	0.482186474	2.41E-31	3.42E-29
CDCA3	0.501647651	3.56E-34	8.47E-32
CDCA4	0.443057132	3.58E-26	2.38E-24
CDCA5	0.507526917	4.57E-35	1.30E-32
CDCA7L	-0.17741277	5.15E-05	0.000137
CDCA7	0.156589511	0.000361	0.00083
CDCA8	0.48809563	3.49E-32	5.71E-30
CDCP1	0.112651833	0.010515	0.018578
CDCP2	-0.11200491	0.010971	0.01931
CDH10	-0.078087427	0.076647	0.11235
CDH11	-0.097784319	0.026486	0.043027
CDH12	0.011726939	0.790633	0.832052
CDH13	-0.006519755	0.882659	0.908053
CDH15	-0.066304093	0.132922	0.183206
CDH16	-0.130099806	0.003098	0.006034
CDH17	0.150015142	0.000637	0.001402
CDH18	0.207247699	2.10E-06	6.99E-06
CDH19	-0.189662657	1.47E-05	4.30E-05
CDH1	-0.158284032	0.000311	0.000724
CDH20	-0.352197785	1.74E-16	2.68E-15
CDH22	-0.148646645	0.000715	0.001561
CDH23	-0.40990497	2.75E-22	9.99E-21
CDH24	0.212326858	1.16E-06	4.02E-06
CDH26	-0.15737934	0.000337	0.000778
CDH2	0.145242975	0.000948	0.002023
CDH3	0.028468442	0.519178	0.591436
CDH4	-0.159188885	0.000287	0.000673
CDH5	-0.224472001	2.64E-07	1.02E-06
CDH6	-0.130913938	0.002915	0.005706
CDH7	0.148951588	0.000696	0.001525
CDH8	-0.06864242	0.119757	0.166936
CDH9	-0.015492922	0.725771	0.777554
CDHR1	-0.271660448	3.65E-10	2.16E-09
CDHR2	0.088812085	0.043952	0.068043
CDHR3	-0.186471147	2.05E-05	5.86E-05
CDHR4	-0.219549745	4.86E-07	1.80E-06
CDHR5	0.025571825	0.562588	0.631866
CDIPT	-0.261293948	1.74E-09	9.29E-09
CDK10	-0.303486677	1.96E-12	1.64E-11
CDK11A	-0.245366193	1.69E-08	7.81E-08
CDK11B	-0.206099596	2.40E-06	7.91E-06

CDK12	0.125385554	0.004375	0.008299
CDK13	-0.04215856	0.339664	0.413749
CDK14	-0.057709465	0.191028	0.251782
CDK15	-0.153700804	0.000465	0.001048
CDK16	0.173387586	7.65E-05	0.000198
CDK17	0.184499779	2.52E-05	7.07E-05
CDK18	-0.418797214	2.74E-23	1.18E-21
CDK19	-0.214947469	8.49E-07	3.01E-06
CDK1	0.524044698	1.15E-37	5.59E-35
CDK20	-0.239501939	3.75E-08	1.64E-07
CDK2AP1	0.207212511	2.11E-06	7.01E-06
CDK2AP2	0.047248556	0.284515	0.355363
CDK2	0.339095966	2.53E-15	3.30E-14
CDK3	-0.30050354	3.29E-12	2.64E-11
CDK4	0.265135063	9.84E-10	5.49E-09
CDK5R1	0.186630521	2.02E-05	5.77E-05
CDK5R2	0.146849943	0.00083	0.00179
CDK5RAP1	0.123882387	0.004872	0.009175
CDK5RAP2	-0.044471896	0.313803	0.385987
CDK5RAP3	-0.288616359	2.45E-11	1.72E-10
CDK5	0.125840263	0.004234	0.008045
CDK6	0.208652822	1.79E-06	6.02E-06
CDK7	0.246084612	1.53E-08	7.11E-08
CDK8	0.376973996	7.77E-19	1.72E-17
CDK9	-0.194009124	9.24E-06	2.78E-05
CDKAL1	0.04107413	0.352243	0.426834
CDKL1	-0.0746737	0.090482	0.130058
CDKL2	-0.362189401	2.08E-17	3.68E-16
CDKL3	-0.176048875	5.90E-05	0.000155
CDKL4	-0.066998543	0.128901	0.178117
CDKL5	-0.130370364	0.003036	0.005923
CDKN1A	-0.074226869	0.092432	0.132478
CDKN1B	-0.03369933	0.445393	0.519673
CDKN1C	-0.180531013	3.77E-05	0.000103
CDKN2AIPNL	0.217637491	6.14E-07	2.23E-06
CDKN2AIP	-0.024608302	0.577405	0.644919
CDKN2A	0.086294466	0.050321	0.076991
CDKN2BAS	-0.025350862	0.56597	0.634791
CDKN2B	-0.071310342	0.106005	0.149687
CDKN2C	0.018981684	0.667374	0.72576
CDKN2D	0.131860668	0.002716	0.005348
CDKN3	0.603483893	2.08E-52	1.38E-48
CDNF	-0.170259994	0.000103	0.000262
CDO1	-0.267674639	6.71E-10	3.83E-09

CDON	-0.191995457	1.15E-05	3.41E-05
CDR1	-0.070313536	0.110989	0.156009
CDR2L	0.052707947	0.232456	0.298626
CDR2	0.086566347	0.049598	0.075961
CDRT15P	-0.210149044	1.50E-06	5.11E-06
CDRT15	-0.061377893	0.164282	0.220766
CDRT1	0.021305302	0.62954	0.691653
CDRT4	-0.213052925	1.06E-06	3.72E-06
CDS1	-0.21995428	4.62E-07	1.72E-06
CDS2	-0.057461071	0.192948	0.253978
CDSN	-0.150608869	0.000606	0.001339
CDT1	0.410607113	2.29E-22	8.43E-21
CDV3	0.162707024	0.000209	0.000504
CDX1	0.048926141	0.267742	0.337345
CDX2	0.136422173	0.001916	0.003874
CDY2B	-0.067968352	0.123443	0.171479
CDYL2	0.006345601	0.885772	0.910388
CDYL	0.21197163	1.21E-06	4.18E-06
CEACAM16	0.102614389	0.019849	0.033106
CEACAM18	0.00064391	0.98837	0.991395
CEACAM19	0.124420898	0.004688	0.008856
CEACAM1	-0.107068803	0.015063	0.025779
CEACAM20	0.042386703	0.337054	0.410996
CEACAM21	-0.228643657	1.56E-07	6.21E-07
CEACAM22P	0.045253963	0.305361	0.377111
CEACAM3	-0.027488338	0.533671	0.604992
CEACAM4	-0.232424336	9.56E-08	3.95E-07
CEACAM5	0.009860077	0.823362	0.859393
CEACAM6	-0.181323726	3.48E-05	9.55E-05
CEACAM7	-0.117523861	0.007589	0.013785
CEACAM8	-0.308000116	8.86E-13	7.82E-12
CEBPA	-0.348220301	3.98E-16	5.84E-15
CEBPB	0.158070259	0.000317	0.000736
CEBPD	-0.142063485	0.001227	0.002569
CEBPE	-0.063923842	0.147444	0.200757
CEBPG	0.244914618	1.80E-08	8.28E-08
CEBPZ	0.292368976	1.31E-11	9.66E-11
CECR1	-0.269406453	5.16E-10	2.99E-09
CECR2	-0.222779954	3.26E-07	1.24E-06
CECR4	-0.191821512	1.17E-05	3.47E-05
CECR5	0.103305018	0.01903	0.031859
CECR6	-0.193410436	9.86E-06	2.95E-05
CECR7	-0.026788088	0.54415	0.614871
CELA1	-0.064200652	0.145695	0.198769

CELA2A	0.003167284	0.942839	0.954587
CELA2B	-0.088152203	0.045552	0.070281
CELA3A	-0.02045271	0.643317	0.70396
CELA3B	-0.060776476	0.168463	0.225822
CELF1	-0.110036559	0.012467	0.02167
CELF2	-0.381702987	2.62E-19	6.27E-18
CELF3	0.039529178	0.370664	0.445457
CELF4	-0.129353071	0.003275	0.006348
CELF5	-0.042536752	0.335345	0.409236
CELF6	-0.386864385	7.84E-20	1.99E-18
CELP	0.043545339	0.324002	0.397071
CELSR1	-0.254807685	4.48E-09	2.24E-08
CELSR2	-0.239558717	3.72E-08	1.63E-07
CELSR3	0.089521007	0.042286	0.065688
CEL	-0.098356229	0.025612	0.041735
CEMP1	-0.362624914	1.89E-17	3.37E-16
CEND1	0.02225577	0.614332	0.678128
CENPA	0.54496671	3.58E-41	3.58E-38
CENPBD1	0.115855759	0.008497	0.015273
CENPB	-0.086138746	0.050739	0.077542
CENPC1	-0.277188591	1.54E-10	9.66E-10
CENPE	0.445487477	1.78E-26	1.23E-24
CENPF	0.422790296	9.54E-24	4.36E-22
CENPH	0.400670918	2.79E-21	8.86E-20
CENPI	0.471807108	6.61E-30	7.13E-28
CENPJ	0.122051423	0.005547	0.010338
CENPK	0.398171831	5.17E-21	1.57E-19
CENPL	0.479031584	6.68E-31	8.51E-29
CENPM	0.346805255	5.32E-16	7.69E-15
CENPN	0.517179396	1.44E-36	5.53E-34
CENPO	0.454823895	1.17E-27	9.28E-26
CENPP	0.193594405	9.66E-06	2.90E-05
CENPQ	0.317714715	1.53E-13	1.51E-12
CENPT	-0.202850961	3.47E-06	1.12E-05
CENPV	0.021202701	0.631191	0.692972
CENPW	0.447500479	9.99E-27	7.08E-25
CEP110	-0.287915727	2.75E-11	1.92E-10
CEP120	-0.208327475	1.85E-06	6.24E-06
CEP135	0.075089263	0.088697	0.127803
CEP152	0.117413535	0.007646	0.013878
CEP164	-0.121373798	0.005817	0.01079
CEP170L	0.023884319	0.588658	0.654914
CEP170	0.080592248	0.067631	0.100379
CEP192	-0.152125559	0.000532	0.001187

CEP250	-0.024212218	0.583549	0.650347
CEP290	-0.08003577	0.069555	0.102944
CEP350	-0.05059104	0.25178	0.319914
CEP55	0.543240825	7.12E-41	6.78E-38
CEP57	0.134056771	0.002299	0.004586
CEP63	-0.062814117	0.154614	0.209279
CEP68	-0.301989628	2.55E-12	2.08E-11
CEP70	-0.101144124	0.021697	0.035897
CEP72	0.083701488	0.057669	0.086956
CEP76	0.293419857	1.10E-11	8.17E-11
CEP78	0.299709174	3.78E-12	3.00E-11
CEP97	0.062310201	0.157956	0.213311
CEPT1	-0.064122956	0.146185	0.199286
CER1	-0.039602791	0.369773	0.444573
CERCAM	0.12277234	0.005272	0.009865
CERKL	-0.258886051	2.48E-09	1.29E-08
CERK	-0.140522441	0.001388	0.002878
CES1	-0.158297913	0.00031	0.000723
CES2	-0.319282445	1.14E-13	1.15E-12
CES3	-0.204325138	2.94E-06	9.55E-06
CES4	-0.139329916	0.001526	0.003142
CES7	-0.092945396	0.03497	0.055286
CES8	-0.325668068	3.45E-14	3.76E-13
CETN1	0.054236431	0.219171	0.283712
CETN2	0.008145755	0.853691	0.884394
CETN3	-0.048632177	0.270631	0.340498
CETN4P	0.028162682	0.523678	0.595679
CETP	-0.064400995	0.14444	0.197271
CFB	0.026084559	0.554778	0.624305
CFC1B	-0.118238377	0.007228	0.013179
CFDP1	-0.020299075	0.645812	0.706232
CFD	-0.273425848	2.78E-10	1.67E-09
CFHR1	0.123992031	0.004834	0.009111
CFHR2	0.120244515	0.006294	0.011602
CFHR3	0.090525993	0.040016	0.062492
CFHR4	0.165019005	0.000169	0.000414
CFHR5	0.148743201	0.000709	0.00155
CFH	-0.11117178	0.011583	0.020283
CFI	-0.203877496	3.09E-06	1.00E-05
CFL1	0.349980595	2.76E-16	4.13E-15
CFL2	0.195641876	7.74E-06	2.35E-05
CFLAR	-0.161130614	0.000241	0.000573
CFLP1	-0.248938903	1.03E-08	4.92E-08
CFP	-0.292949061	1.19E-11	8.81E-11

CFTR	-0.29665025	6.38E-12	4.91E-11
CG030	-0.33079943	1.29E-14	1.49E-13
CGA	0.181467958	3.43E-05	9.42E-05
CGB1	0.106099885	0.016008	0.027201
CGB2	-0.042023313	0.341217	0.415387
CGB5	-0.005669539	0.897871	0.9202
CGB7	-0.26146882	1.70E-09	9.08E-09
CGB8	0.068039041	0.123052	0.171041
CGB	0.039558333	0.370311	0.445139
CGGBP1	-0.03222683	0.46554	0.53937
CGNL1	-0.475116166	2.33E-30	2.75E-28
CGN	-0.195417186	7.93E-06	2.41E-05
CGREF1	0.294803667	8.72E-12	6.59E-11
CGRRF1	0.191007643	1.27E-05	3.76E-05
CH25H	-0.317652259	1.54E-13	1.52E-12
CHAC1	0.226257702	2.11E-07	8.25E-07
CHAC2	0.441507376	5.56E-26	3.61E-24
CHADL	-0.404853734	9.85E-22	3.36E-20
CHAD	-0.405919297	7.54E-22	2.61E-20
CHAF1A	0.250835376	7.88E-09	3.83E-08
CHAF1B	0.293841466	1.03E-11	7.65E-11
CHAT	-0.112751779	0.010446	0.01847
CHCHD10	0.128726876	0.00343	0.006626
CHCHD1	0.232200991	9.84E-08	4.06E-07
CHCHD2	0.337823799	3.26E-15	4.18E-14
CHCHD3	0.410027009	2.66E-22	9.71E-21
CHCHD4	0.222132091	3.53E-07	1.34E-06
CHCHD5	0.104069211	0.018158	0.030525
CHCHD6	-0.052954173	0.230278	0.296266
CHCHD7	0.11123474	0.011536	0.020209
CHCHD8	0.363420952	1.59E-17	2.87E-16
CHD1L	0.26970437	4.93E-10	2.86E-09
CHD1	-0.043094698	0.329039	0.402499
CHD2	-0.31012704	6.06E-13	5.50E-12
CHD3	-0.175625944	6.15E-05	0.000161
CHD4	-0.030823711	0.485198	0.558859
CHD5	-0.221452753	3.84E-07	1.44E-06
CHD6	-0.205483351	2.58E-06	8.45E-06
CHD7	0.16450631	0.000177	0.000433
CHD8	0.048134217	0.275574	0.345947
CHD9	-0.273073472	2.94E-10	1.76E-09
CHDH	-0.160649208	0.000252	0.000597
CHEK1	0.468065924	2.12E-29	2.12E-27
CHEK2	0.345038989	7.63E-16	1.07E-14

CHERP	-0.02923187	0.508031	0.580524
CHFR	0.130091995	0.0031	0.006037
CHGA	0.008216109	0.852442	0.883436
CHGB	0.067086534	0.128398	0.177521
CHI3L1	-0.187657178	1.82E-05	5.23E-05
CHI3L2	-0.194976764	8.32E-06	2.52E-05
CHIA	-0.313475948	3.32E-13	3.13E-12
CHIC1	-0.238742334	4.15E-08	1.81E-07
CHIC2	0.139657054	0.001487	0.003066
CHID1	-0.011797738	0.789398	0.831059
CHIT1	-0.28433352	4.93E-11	3.31E-10
CHKA	-0.205134248	2.68E-06	8.76E-06
CHKB-CPT1B	-0.341156389	1.67E-15	2.24E-14
CHKB	-0.144247214	0.001028	0.002183
CHL1	0.008413421	0.848941	0.880658
CHML	0.218238641	5.70E-07	2.09E-06
CHMP1A	0.099911597	0.023358	0.038373
CHMP1B	0.173625998	7.47E-05	0.000194
CHMP2A	-0.073586595	0.095286	0.136187
CHMP2B	0.205351781	2.61E-06	8.56E-06
CHMP4A	0.16343935	0.000195	0.000473
CHMP4B	-0.007664695	0.862243	0.89164
CHMP4C	0.327253586	2.55E-14	2.83E-13
CHMP5	0.282596627	6.53E-11	4.31E-10
CHMP6	-0.199413875	5.11E-06	1.60E-05
CHMP7	-0.080002827	0.06967	0.103093
CHM	0.062319624	0.157893	0.213255
CHN1	0.052500754	0.234299	0.300743
CHN2	-0.239709082	3.65E-08	1.60E-07
CHODL	0.032088341	0.46746	0.541249
CHORDC1	0.404648215	1.04E-21	3.53E-20
CHP2	-0.135262548	0.002096	0.004213
CHPF2	0.071917778	0.103055	0.146069
CHPF	0.026471389	0.548922	0.619214
CHPT1	0.007303896	0.868668	0.897355
CHP	0.005077112	0.908494	0.92842
CHRAC1	0.290868055	1.69E-11	1.22E-10
CHRDL1	-0.376082449	9.52E-19	2.08E-17
CHRDL2	-0.001033413	0.981335	0.986121
CHRD	-0.028135043	0.524086	0.596041
CHRFAM7A	-0.082024796	0.062878	0.094045
CHRM1	-0.307609849	9.50E-13	8.35E-12
CHRM2	-0.146972619	0.000822	0.001775
CHRM3	0.003198677	0.942273	0.954159

CHRM4	0.007919353	0.857714	0.887922
CHRM5	-0.004109207	0.925882	0.942144
CHRNA10	-0.268296839	6.11E-10	3.51E-09
CHRNA1	-0.080948029	0.066424	0.098793
CHRNA2	-0.291368509	1.55E-11	1.13E-10
CHRNA3	0.013565353	0.758757	0.805419
CHRNA4	-0.101430999	0.021325	0.035352
CHRNA5	0.302866802	2.19E-12	1.81E-11
CHRNA6	-0.230142292	1.28E-07	5.20E-07
CHRNA7	-0.08088783	0.066627	0.099059
CHRNA9	0.306526612	1.15E-12	9.99E-12
CHRNB1	-0.039034996	0.37668	0.451465
CHRNB2	-0.0225554	0.609572	0.673901
CHRNB3	-0.288749375	2.40E-11	1.69E-10
CHRNB4	0.117233883	0.00774	0.014033
CHRND	0.025633375	0.561648	0.631041
CHRNE	-0.089669066	0.041945	0.065244
CHRNG	-0.056157714	0.203256	0.265852
CHST10	-0.229536746	1.39E-07	5.59E-07
CHST11	0.161886217	0.000225	0.000539
CHST12	-0.152943757	0.000496	0.001112
CHST13	-0.030109601	0.495372	0.568578
CHST14	-0.107157644	0.014979	0.025657
CHST15	0.023372094	0.59668	0.66247
CHST1	0.145894114	0.000898	0.001925
CHST2	-0.179652801	4.12E-05	0.000112
CHST3	-0.013219109	0.764731	0.810684
CHST4	-0.019848955	0.653147	0.712887
CHST5	-0.274652515	2.30E-10	1.40E-09
CHST6	-0.010530229	0.811575	0.849574
CHST7	-0.222807888	3.25E-07	1.24E-06
CHST8	-0.237849971	4.68E-08	2.02E-07
CHST9	-0.223322181	3.05E-07	1.16E-06
CHSY1	0.052691521	0.232601	0.298794
CHSY3	-0.095754004	0.029802	0.047895
CHTF18	0.003829791	0.930909	0.945955
CHTF8	0.013634721	0.757562	0.80445
CHUK	0.370983674	3.00E-18	6.05E-17
CHURC1	0.072598661	0.099827	0.142037
CIAO1	0.196126754	7.34E-06	2.24E-05
CIAPIN1	0.326288183	3.07E-14	3.36E-13
CIB1	0.050881672	0.249062	0.316986
CIB2	0.177344687	5.19E-05	0.000138
CIB3	0.005989808	0.892136	0.91559

CIB4	0.080115976	0.069275	0.102575
CIC	-0.24646461	1.45E-08	6.78E-08
CIDEA	0.085387329	0.052797	0.080331
CIDEB	-0.141195704	0.001316	0.002738
CIDECP	0.066789185	0.130103	0.179667
CIDEC	0.163156927	0.0002	0.000485
CIITA	-0.414285726	8.90E-23	3.51E-21
CILP2	0.011481092	0.794923	0.835775
CILP	-0.01187024	0.788135	0.829948
CINP	0.209867965	1.55E-06	5.27E-06
CIR1	-0.111376248	0.01143	0.020049
CIRBP	-0.501824713	3.35E-34	8.27E-32
CIRH1A	0.324338038	4.44E-14	4.75E-13
CISD1	0.359412894	3.78E-17	6.46E-16
CISD2	0.397031408	6.83E-21	2.04E-19
CISD3	-0.008898552	0.840344	0.873332
CISH	-0.373251918	1.81E-18	3.74E-17
CITED1	-0.105062019	0.017077	0.028858
CITED2	-0.35958904	3.64E-17	6.25E-16
CITED4	-0.060777297	0.168457	0.225822
CIT	-0.091794657	0.037298	0.058601
CIZ1	-0.009806034	0.824315	0.860072
CKAP2L	0.505558595	9.13E-35	2.53E-32
CKAP2	0.46074296	1.99E-28	1.72E-26
CKAP4	0.359248844	3.92E-17	6.67E-16
CKAP5	0.245574953	1.64E-08	7.60E-08
CKB	-0.132428362	0.002602	0.005137
CKLF	0.047051526	0.28653	0.357504
CKMT1A	0.063896657	0.147616	0.200942
CKMT1B	0.102371366	0.020145	0.033553
CKMT2	-0.261583358	1.67E-09	8.94E-09
CKM	0.08476394	0.054557	0.082788
CKS1B	0.499238535	8.17E-34	1.81E-31
CKS2	0.483836776	1.41E-31	2.09E-29
CLASP1	-0.106276274	0.015832	0.026936
CLASP2	-0.012394735	0.779011	0.822246
CLCA1	0.010321407	0.815243	0.852744
CLCA2	-0.136779295	0.001864	0.003776
CLCA3P	0.122743513	0.005283	0.009881
CLCA4	-0.104634699	0.017535	0.029555
CLCC1	-0.261461478	1.70E-09	9.09E-09
CLCF1	0.070848728	0.10829	0.152634
CLCN1	-0.010356307	0.81463	0.852236
CLCN2	0.061405015	0.164095	0.220574

CLCN3	0.043663572	0.322689	0.395748
CLCN4	-0.108939588	0.013376	0.023112
CLCN5	-0.200480427	4.54E-06	1.43E-05
CLCN6	-0.386776464	8.01E-20	2.03E-18
CLCN7	-0.255549371	4.02E-09	2.03E-08
CLCNKA	-0.270492495	4.37E-10	2.56E-09
CLCNKB	-0.224179933	2.74E-07	1.05E-06
CLC	-0.030072143	0.495909	0.569079
CLDN10	0.160518702	0.000255	0.000603
CLDN11	-0.126131044	0.004146	0.007894
CLDN12	0.255296308	4.17E-09	2.10E-08
CLDN14	0.238927893	4.05E-08	1.77E-07
CLDN15	-0.227052735	1.91E-07	7.51E-07
CLDN16	-0.252396725	6.32E-09	3.10E-08
CLDN17	-0.010694785	0.808687	0.847038
CLDN18	-0.292518204	1.28E-11	9.43E-11
CLDN19	0.088606721	0.044444	0.068737
CLDN1	-0.177648907	5.04E-05	0.000134
CLDN20	-0.212550905	1.13E-06	3.92E-06
CLDN22	-0.01426775	0.746683	0.79522
CLDN23	-0.291555109	1.50E-11	1.10E-10
CLDN25	-0.036158061	0.412883	0.487576
CLDN2	-0.226553628	2.03E-07	7.97E-07
CLDN3	-0.112929679	0.010325	0.018276
CLDN4	-0.114980412	0.009011	0.016118
CLDN5	-0.130130438	0.003091	0.006022
CLDN6	0.054527212	0.216707	0.281123
CLDN7	0.079534764	0.071325	0.105283
CLDN8	-0.133844661	0.002337	0.004654
CLDN9	-0.198498902	5.65E-06	1.76E-05
CLDND1	0.177264058	5.23E-05	0.000139
CLDND2	-0.131884774	0.002711	0.00534
CLEC10A	-0.280976985	8.46E-11	5.52E-10
CLEC11A	-0.084458418	0.055437	0.083958
CLEC12A	-0.183022388	2.93E-05	8.14E-05
CLEC12B	-0.313870226	3.09E-13	2.92E-12
CLEC14A	-0.290297794	1.85E-11	1.33E-10
CLEC16A	-0.380650002	3.34E-19	7.81E-18
CLEC17A	-0.203054962	3.40E-06	1.09E-05
CLEC18A	-0.15758266	0.000331	0.000766
CLEC18B	-0.152594047	0.000511	0.001144
CLEC18C	0.016567465	0.707595	0.762078
CLEC1A	-0.270263611	4.53E-10	2.64E-09
CLEC1B	-0.128333903	0.00353	0.006804

CLEC2A	0.02029319	0.645908	0.706297
CLEC2B	-0.060336595	0.171571	0.229428
CLEC2D	-0.083939554	0.056959	0.085996
CLEC2L	0.125001991	0.004497	0.008515
CLEC3A	-0.149982486	0.000639	0.001406
CLEC3B	-0.312178891	4.19E-13	3.88E-12
CLEC4A	-0.15810832	0.000316	0.000734
CLEC4C	-0.02370783	0.591417	0.657429
CLEC4D	0.064387116	0.144527	0.197376
CLEC4E	0.033213787	0.451981	0.526345
CLEC4F	-0.464180745	7.00E-29	6.54E-27
CLEC4GP1	-0.200736977	4.41E-06	1.39E-05
CLEC4G	0.011113711	0.801346	0.840804
CLEC4M	-0.082539417	0.06124	0.091869
CLEC5A	-0.157910383	0.000321	0.000746
CLEC6A	0.061909078	0.160655	0.216503
CLEC7A	-0.180215055	3.90E-05	0.000106
CLEC9A	-0.310040043	6.16E-13	5.57E-12
CLECL1	-0.254457075	4.71E-09	2.35E-08
CLGN	0.183366288	2.83E-05	7.87E-05
CLIC1	0.312574854	3.90E-13	3.63E-12
CLIC2	-0.159500685	0.000279	0.000656
CLIC3	-0.266937172	7.50E-10	4.25E-09
CLIC4	0.145656731	0.000916	0.00196
CLIC5	-0.389214935	4.49E-20	1.18E-18
CLIC6	-0.322250512	6.57E-14	6.84E-13
CLINT1	-0.154649256	0.000428	0.000972
CLIP1	0.172945864	7.98E-05	0.000206
CLIP2	-0.118712151	0.006997	0.012787
CLIP3	-0.193564264	9.69E-06	2.91E-05
CLIP4	-0.077560616	0.078662	0.1149
CLK1	-0.311916724	4.40E-13	4.05E-12
CLK2P	-0.049658745	0.260635	0.329469
CLK2	-0.158762197	0.000298	0.000696
CLK3	-0.121857539	0.005623	0.010468
CLK4	-0.421444419	1.36E-23	6.13E-22
CLLU1OS	0.041725892	0.344648	0.418875
CLLU1	0.022186102	0.615442	0.679113
CLMN	-0.035009404	0.427893	0.502481
CLN3	-0.210729745	1.40E-06	4.80E-06
CLN5	-0.179357287	4.25E-05	0.000115
CLN6	0.021954988	0.619128	0.682389
CLN8	-0.158286419	0.000311	0.000724
CLNK	-0.106651932	0.015463	0.026374

CLNS1A	0.396539312	7.70E-21	2.28E-19
CLOCK	0.11033612	0.012228	0.021298
CLP1	0.218803243	5.32E-07	1.96E-06
CLPB	0.293228718	1.14E-11	8.42E-11
CLPP	0.083982726	0.056831	0.085835
CLPS	0.003527122	0.936357	0.949998
CLPTM1L	-0.197596313	6.25E-06	1.93E-05
CLPTM1	0.002774493	0.949917	0.960878
CLPX	0.209516178	1.61E-06	5.48E-06
CLRN1OS	-0.076973295	0.080959	0.117876
CLRN1	-0.084216112	0.056144	0.084919
CLRN2	-0.060887153	0.167688	0.224889
CLRN3	0.010477147	0.812507	0.850267
CLSPN	0.360400831	3.06E-17	5.32E-16
CLSTN1	-0.081913307	0.063238	0.094512
CLSTN2	-0.054619175	0.215932	0.280226
CLSTN3	-0.051953627	0.239217	0.306054
CLTA	0.142786435	0.001158	0.002434
CLTB	-0.054031437	0.220921	0.285692
CLTCL1	0.042531388	0.335406	0.409286
CLTC	0.088820846	0.043931	0.068016
CLUAP1	-0.278201397	1.32E-10	8.32E-10
CLUL1	-0.400702388	2.77E-21	8.80E-20
CLU	-0.312034468	4.30E-13	3.97E-12
CLVS1	0.020089472	0.649224	0.709264
CLVS2	0.096713649	0.028193	0.045536
CLYBL	-0.138937289	0.001574	0.003228
CMA1	-0.289918806	1.97E-11	1.41E-10
CMAH	-0.325345214	3.67E-14	3.98E-13
CMAS	0.320335836	9.40E-14	9.55E-13
CMBL	0.170284275	0.000103	0.000262
CMC1	0.27122323	3.91E-10	2.30E-09
CMIP	0.067838687	0.124162	0.172272
CMKLR1	-0.153903917	0.000457	0.001031
CMPK1	0.068168513	0.122339	0.170216
CMPK2	-0.083283098	0.058934	0.08869
CMTM1	0.001575365	0.97155	0.978551
CMTM2	-0.077838564	0.077593	0.11353
CMTM3	-0.013957204	0.752014	0.799918
CMTM4	-0.037526074	0.395418	0.470333
CMTM5	-0.288707129	2.41E-11	1.70E-10
CMTM6	0.072225482	0.101586	0.144232
CMTM7	-0.302137779	2.48E-12	2.03E-11
CMTM8	-0.050025169	0.257129	0.325862

CMYA5	-0.390507356	3.30E-20	8.90E-19
CN5H6.4	-0.076089317	0.084519	0.122418
CNBD1	0.044220586	0.316548	0.388917
CNBP	0.190582493	1.33E-05	3.92E-05
CNDP1	0.016242236	0.713079	0.766586
CNDP2	-0.135913646	0.001993	0.004023
CNFN	-0.098674653	0.025136	0.04101
CNGA1	-0.131657917	0.002757	0.005421
CNGA2	-0.027347602	0.535769	0.606843
CNGA3	-0.023765282	0.590518	0.65654
CNGA4	-0.297120888	5.89E-12	4.55E-11
CNGB1	-0.033961422	0.44186	0.516183
CNGB3	0.264545473	1.07E-09	5.95E-09
CNIH2	-0.005490118	0.901087	0.922671
CNIH3	-0.054338894	0.218301	0.282713
CNIH4	0.066960205	0.12912	0.178408
CNIH	0.518008801	1.06E-36	4.17E-34
CNKSR1	-0.345478791	6.98E-16	9.85E-15
CNKSR2	-0.365550385	9.99E-18	1.88E-16
CNKSR3	-0.087607858	0.046908	0.072167
CNN1	-0.107748659	0.014429	0.02478
CNN2	-0.10386733	0.018385	0.03087
CNN3	-0.045461605	0.303144	0.374884
CNNM1	-0.15548821	0.000398	0.000907
CNNM2	-0.129983159	0.003125	0.006084
CNNM3	-0.365367276	1.04E-17	1.94E-16
CNNM4	-0.08774932	0.046552	0.071686
CNOT10	0.177054467	5.34E-05	0.000142
CNOT1	0.007206445	0.870404	0.898917
CNOT2	0.173462113	7.59E-05	0.000197
CNOT3	-0.045219288	0.305732	0.377476
CNOT4	-0.052778088	0.231834	0.297904
CNOT6L	-0.160003652	0.000267	0.000629
CNOT6	-0.027727217	0.53012	0.601774
CNOT7	0.178028731	4.85E-05	0.00013
CNOT8	-0.083195147	0.059203	0.089034
CNO	0.310831796	5.34E-13	4.87E-12
CNPY1	-0.042740546	0.333033	0.406737
CNPY2	0.313427794	3.34E-13	3.15E-12
CNPY3	-0.042867146	0.331602	0.405212
CNPY4	-0.012007992	0.785736	0.827857
CNP	0.095408478	0.0304	0.048732
CNR1	-0.353512483	1.32E-16	2.06E-15
CNR2	-0.256258785	3.63E-09	1.84E-08

CNRIP1	-0.210431458	1.45E-06	4.95E-06
CNST	-0.045244067	0.305466	0.377195
CNTD1	-0.269407567	5.16E-10	2.99E-09
CNTD2	0.21560466	7.85E-07	2.80E-06
CNTFR	-0.372745205	2.02E-18	4.17E-17
CNTF	-0.008208725	0.852573	0.883469
CNTLN	-0.077912432	0.077311	0.113151
CNTN1	0.000844193	0.984752	0.988461
CNTN2	-0.287546924	2.92E-11	2.03E-10
CNTN3	-0.179484151	4.19E-05	0.000113
CNTN4	-0.26822801	6.17E-10	3.54E-09
CNTN5	0.011985719	0.786123	0.828146
CNTN6	-0.336306722	4.40E-15	5.52E-14
CNTNAP1	-0.176601802	5.58E-05	0.000148
CNTNAP2	0.052949616	0.230318	0.296299
CNTNAP3	-0.077341981	0.079511	0.11603
CNTNAP4	0.120943827	0.005995	0.011092
CNTNAP5	0.075422281	0.087288	0.126008
CNTROB	-0.291538178	1.51E-11	1.10E-10
COASY	0.156457212	0.000365	0.000839
COBLL1	-0.302035812	2.53E-12	2.07E-11
COBL	-0.136406759	0.001919	0.003878
COBRA1	-0.022891267	0.604255	0.669207
COCH	0.247243539	1.30E-08	6.12E-08
COG1	-0.092258895	0.036343	0.057267
COG2	0.136251853	0.001942	0.003924
COG3	0.028974501	0.511775	0.584034
COG4	-0.105010706	0.017131	0.02894
COG5	0.216913338	6.70E-07	2.42E-06
COG6	0.173706167	7.41E-05	0.000192
COG7	-0.21018729	1.49E-06	5.09E-06
COG8	-0.054520159	0.216767	0.281144
COIL	0.186150963	2.12E-05	6.05E-05
COL10A1	1.40E-05	0.999747	0.999963
COL11A1	0.264462668	1.09E-09	6.02E-09
COL11A2	-0.244621976	1.87E-08	8.60E-08
COL12A1	0.186096075	2.14E-05	6.08E-05
COL13A1	-0.314818432	2.60E-13	2.48E-12
COL14A1	-0.288346932	2.56E-11	1.79E-10
COL15A1	0.110716093	0.011931	0.020819
COL16A1	-0.220037984	4.58E-07	1.70E-06
COL17A1	-0.111884751	0.011057	0.019449
COL18A1	-0.041325209	0.349304	0.423839
COL19A1	-0.28479276	4.58E-11	3.09E-10

COL1A1	0.115226688	0.008863	0.015876
COL1A2	0.112928198	0.010326	0.018276
COL20A1	-0.033197059	0.452209	0.526519
COL21A1	-0.38040045	3.54E-19	8.25E-18
COL22A1	-0.013046308	0.767718	0.813203
COL23A1	-0.199701491	4.95E-06	1.55E-05
COL24A1	-0.128656556	0.003447	0.006657
COL25A1	0.066067303	0.134316	0.184958
COL27A1	-0.266830017	7.63E-10	4.32E-09
COL28A1	-0.208385774	1.84E-06	6.20E-06
COL29A1	-0.363753086	1.48E-17	2.68E-16
COL2A1	0.024394906	0.580712	0.647691
COL3A1	0.157760882	0.000326	0.000755
COL4A1	0.079120924	0.072815	0.107182
COL4A2	-0.018303053	0.67859	0.735916
COL4A3BP	-0.312036078	4.30E-13	3.97E-12
COL4A3	-0.430687085	1.13E-24	5.92E-23
COL4A4	-0.381950099	2.48E-19	5.94E-18
COL4A5	-0.130083546	0.003102	0.00604
COL4A6	-0.129094663	0.003338	0.006466
COL5A1	0.057366049	0.193687	0.254765
COL5A2	0.179820634	4.05E-05	0.00011
COL5A3	0.072289194	0.101284	0.143885
COL6A1	0.007000642	0.874074	0.901682
COL6A2	-0.043897237	0.320103	0.392867
COL6A3	-0.015498865	0.72567	0.777554
COL6A4P2	-0.226976953	1.92E-07	7.58E-07
COL6A6	-0.362957092	1.76E-17	3.15E-16
COL7A1	0.043215162	0.327688	0.400993
COL8A1	-0.117627511	0.007536	0.013692
COL8A2	-0.189766743	1.45E-05	4.26E-05
COL9A1	-0.141746233	0.001259	0.002632
COL9A2	-0.337915499	3.20E-15	4.11E-14
COL9A3	-0.053775318	0.22312	0.288134
COLEC10	-0.101715228	0.020962	0.034803
COLEC11	-0.00919347	0.835127	0.86882
COLEC12	-0.350686678	2.39E-16	3.58E-15
COLQ	-0.323668019	5.03E-14	5.33E-13
COMMD10	0.114877619	0.009073	0.016223
COMMD1	0.102799683	0.019627	0.032768
COMMD2	0.159854143	0.00027	0.000637
COMMD3	0.106123146	0.015984	0.027168
COMMD4	0.015467429	0.726204	0.777803
COMMD5	0.185783423	2.21E-05	6.26E-05

COMMD6	-0.016149315	0.714649	0.767814
COMMD7	0.171856484	8.86E-05	0.000227
COMMD8	0.26204745	1.56E-09	8.40E-09
COMMD9	0.042716946	0.3333	0.407039
COMP	-0.093638746	0.033628	0.053358
COMTD1	0.154949344	0.000417	0.000948
COMT	-0.076008076	0.084852	0.122838
COPA	0.161978346	0.000223	0.000535
COPB1	0.283184338	5.94E-11	3.95E-10
COPB2	0.280598539	8.99E-11	5.83E-10
COPE	0.113010433	0.01027	0.018192
COPG2	0.108366429	0.013874	0.023909
COPG	0.192583418	1.08E-05	3.21E-05
COPS2	0.252432657	6.28E-09	3.09E-08
COPS3	0.300954763	3.05E-12	2.46E-11
COPS4	0.169720015	0.000109	0.000275
COPS5	0.370243703	3.54E-18	7.06E-17
COPS6	0.285483309	4.09E-11	2.78E-10
COPS7A	0.13114758	0.002865	0.005611
COPS7B	0.028653922	0.516459	0.588673
COPS8	0.275465092	2.02E-10	1.24E-09
COPZ1	0.371798823	2.50E-18	5.10E-17
COPZ2	-0.035052849	0.42732	0.501925
COQ10A	-0.174822011	6.65E-05	0.000174
COQ10B	0.133165358	0.002461	0.00488
COQ2	0.240513804	3.27E-08	1.45E-07
COQ3	0.223444169	3.00E-07	1.15E-06
COQ4	-0.19215412	1.13E-05	3.35E-05
COQ5	0.286781447	3.31E-11	2.28E-10
COQ6	0.138031778	0.00169	0.003453
COQ7	-0.155792	0.000387	0.000885
COQ9	0.128030244	0.00361	0.006942
CORIN	-0.019694248	0.655676	0.715218
CORO1A	-0.252767599	5.99E-09	2.96E-08
CORO1B	0.019644578	0.656489	0.715909
CORO1C	0.205408756	2.60E-06	8.51E-06
CORO2A	-0.19360526	9.65E-06	2.90E-05
CORO2B	-0.356259574	7.41E-17	1.21E-15
CORO6	-0.069792016	0.113669	0.159496
CORO7	-0.301309218	2.87E-12	2.32E-11
CORT	-0.100410653	0.022673	0.037348
COTL1	-0.030649175	0.487674	0.561014
COX10	0.156208575	0.000373	0.000856
COX11	0.07880192	0.07398	0.10873

COX15	0.228473758	1.59E-07	6.33E-07
COX16	0.206805661	2.21E-06	7.33E-06
COX17	0.165440396	0.000162	0.000399
COX18	0.078334826	0.075715	0.111139
COX19	-0.091926751	0.037024	0.058216
COX4I1	0.105286575	0.01684	0.02849
COX4I2	-0.243534354	2.17E-08	9.87E-08
COX4NB	0.350319923	2.58E-16	3.86E-15
COX5A	0.293931572	1.01E-11	7.54E-11
COX5B	0.202134085	3.77E-06	1.20E-05
COX6A1	0.262997012	1.35E-09	7.35E-09
COX6A2	-0.003273247	0.94093	0.953427
COX6B1	0.186513334	2.05E-05	5.83E-05
COX6B2	0.09396456	0.033012	0.052457
COX6C	0.227922473	1.71E-07	6.77E-07
COX7A1	-0.16009393	0.000264	0.000624
COX7A2L	0.29536463	7.93E-12	6.04E-11
COX7A2	0.247149896	1.32E-08	6.19E-08
COX7B2	0.187281581	1.89E-05	5.42E-05
COX7B	0.226171639	2.13E-07	8.32E-07
COX7C	0.077349377	0.079482	0.115996
COX8A	0.19597809	7.46E-06	2.27E-05
COX8C	0.051974147	0.239032	0.305855
CP110	-0.237308103	5.03E-08	2.16E-07
CPA1	-0.042051585	0.340892	0.415042
CPA2	0.057645204	0.191524	0.252368
CPA3	-0.306349145	1.19E-12	1.03E-11
CPA4	0.160278467	0.00026	0.000615
CPA5	0.020853996	0.636817	0.698037
CPA6	-0.016812663	0.703471	0.758699
CPAMD8	-0.488388835	3.16E-32	5.23E-30
CPB1	0.013878728	0.753363	0.801012
CPB2	-0.186822935	1.98E-05	5.66E-05
CPD	0.249054746	1.01E-08	4.85E-08
CPEB1	-0.174827592	6.65E-05	0.000174
CPEB2	-0.018823538	0.669981	0.727962
CPEB3	-0.326018696	3.23E-14	3.53E-13
CPEB4	-0.287025198	3.18E-11	2.20E-10
CPE	0.287635673	2.88E-11	2.00E-10
CPLX1	0.061725725	0.1619	0.217902
CPLX2	0.228589693	1.57E-07	6.25E-07
CPLX3	-0.081942751	0.063143	0.094394
CPLX4	0.104112279	0.01811	0.030454
CPM	-0.117902276	0.007396	0.013463

CPN1	0.222260914	3.48E-07	1.32E-06
CPN2	0.014278827	0.746493	0.79506
CPNE1	0.096720788	0.028181	0.045525
CPNE2	-0.372454252	2.16E-18	4.44E-17
CPNE3	0.182004	3.25E-05	8.95E-05
CPNE4	-0.057991135	0.188868	0.249312
CPNE5	-0.088636924	0.044372	0.06863
CPNE6	-0.016613955	0.706812	0.761405
CPNE7	-0.124691259	0.004599	0.008694
CPNE8	0.068726046	0.119306	0.166365
CPNE9	-0.286184118	3.65E-11	2.50E-10
CPOX	0.148295609	0.000736	0.001604
CPO	-0.200702258	4.42E-06	1.40E-05
CPPED1	-0.091802274	0.037282	0.058584
CPS1	0.309724095	6.52E-13	5.88E-12
CPSF1	-0.03161963	0.473992	0.547702
CPSF2	0.393804208	1.50E-20	4.19E-19
CPSF3L	-0.168215403	0.000125	0.000314
CPSF3	0.37027489	3.52E-18	7.03E-17
CPSF4L	-0.006259345	0.887314	0.911624
CPSF4	0.203230819	3.33E-06	1.07E-05
CPSF6	0.120185904	0.006319	0.011644
CPSF7	-0.068895402	0.118396	0.1652
CPT1A	0.04950159	0.262148	0.331099
CPT1B	-0.249775319	9.15E-09	4.40E-08
CPT1C	0.095498851	0.030242	0.048529
CPT2	-0.142182885	0.001215	0.002546
CPVL	-0.218234208	5.71E-07	2.09E-06
CPXCR1	-0.05547989	0.208772	0.272083
CPXM1	0.106121418	0.015986	0.027169
CPXM2	-0.161284031	0.000238	0.000566
CPZ	-0.102889064	0.01952	0.032603
CP	-0.009214864	0.834749	0.868646
CR1L	0.049876218	0.25855	0.32731
CR1	-0.179577094	4.15E-05	0.000112
CR2	-0.176775706	5.49E-05	0.000145
CRABP1	0.144698403	0.000991	0.002109
CRABP2	-0.024113237	0.585089	0.651773
CRADD	0.103222381	0.019126	0.032
CRAMP1L	-0.302448611	2.35E-12	1.93E-11
CRAT	-0.132117925	0.002663	0.005253
CRB1	-0.122147368	0.005509	0.010273
CRB2	-0.232101154	9.97E-08	4.10E-07
CRB3	-0.148025403	0.000753	0.001638

CRBN	-0.107860608	0.014327	0.024622
CRCP	0.16232181	0.000216	0.00052
CRCT1	0.019556137	0.657937	0.717136
CREB1	-0.090474654	0.040129	0.062649
CREB3L1	-0.142410568	0.001193	0.002503
CREB3L2	0.05209728	0.237919	0.304607
CREB3L3	0.002274908	0.958927	0.968521
CREB3L4	0.083642664	0.057846	0.087183
CREB3	0.115081406	0.00895	0.016023
CREB5	-0.103272204	0.019068	0.031913
CREBBP	-0.259953296	2.12E-09	1.12E-08
CREBL2	-0.196884919	6.75E-06	2.07E-05
CREBZF	-0.14092578	0.001344	0.002792
CREG1	-0.051764994	0.24093	0.308031
CREG2	0.241848002	2.73E-08	1.22E-07
CRELD1	-0.179872289	4.03E-05	0.000109
CRELD2	0.016642835	0.706326	0.76116
CREM	0.262007436	1.57E-09	8.44E-09
CRHBP	-0.268102574	6.29E-10	3.60E-09
CRHR1	-0.092153401	0.036558	0.057542
CRHR2	-0.066414802	0.132275	0.182376
CRH	-0.015135054	0.731859	0.782853
CRIM1	-0.164546042	0.000176	0.000431
CRIP1	-0.001443833	0.973925	0.980417
CRIP2	-0.123343363	0.005063	0.009504
CRIP3	-0.027653261	0.531218	0.602747
CRIPAK	-0.175248296	6.38E-05	0.000167
CRIPT	0.296262215	6.81E-12	5.23E-11
CRISP1	0.008928169	0.83982	0.872878
CRISP2	-0.095269581	0.030643	0.049078
CRISP3	0.009996356	0.820962	0.85729
CRISPLD1	-0.092948989	0.034963	0.055279
CRISPLD2	0.012254382	0.781449	0.824297
CRKL	0.071775646	0.103739	0.146831
CRK	0.122265305	0.005464	0.010196
CRLF1	-0.0527888	0.231739	0.29782
CRLF2	-0.059472652	0.177799	0.236765
CRLF3	0.048544383	0.271498	0.341453
CRLS1	-0.084024583	0.056707	0.085687
CRMP1	-0.188367762	1.69E-05	4.88E-05
CRNKL1	0.137648291	0.001742	0.003547
CRNN	0.004321772	0.92206	0.938972
CROCCL1	-0.347518964	4.59E-16	6.69E-15
CROCCL2	-0.432612166	6.67E-25	3.67E-23

CROCC	-0.412453036	1.43E-22	5.39E-21
CROT	-0.179788938	4.07E-05	0.00011
CRP	0.114651695	0.009211	0.01646
CRTAC1	-0.348051397	4.12E-16	6.03E-15
CRTAM	-0.122674945	0.005308	0.009924
CRTAP	-0.129910218	0.003142	0.006113
CRTC1	-0.327791816	2.30E-14	2.57E-13
CRTC2	-0.065156983	0.139777	0.191557
CRTC3	-0.089929982	0.041349	0.064413
CRX	-0.021656139	0.623908	0.686682
CRY1	0.175451779	6.25E-05	0.000164
CRY2	-0.509665008	2.14E-35	6.70E-33
CRYAA	0.068219823	0.122058	0.169836
CRYAB	-0.039397411	0.372262	0.447055
CRYBA1	-0.055424912	0.209225	0.272584
CRYBA2	0.134965167	0.002144	0.004304
CRYBA4	-0.104336536	0.017861	0.030059
CRYBB1	-0.087355075	0.047549	0.073036
CRYBB2	-0.083680751	0.057731	0.087035
CRYBB3	-0.171264643	9.38E-05	0.00024
CRYBG3	-0.134475438	0.002227	0.004454
CRYGA	-0.003544217	0.93605	0.949825
CRYGB	0.079929552	0.069927	0.103388
CRYGC	0.098684513	0.025121	0.040993
CRYGD	0.039099178	0.375896	0.450717
CRYGN	-0.076025251	0.084782	0.122754
CRYGS	-0.310563668	5.61E-13	5.11E-12
CRYL1	-0.079628185	0.070992	0.104823
CRYM	-0.32826218	2.10E-14	2.36E-13
CRYZL1	-0.111227341	0.011542	0.020217
CRYZ	-0.03413452	0.439535	0.513858
CSAD	-0.346156193	6.07E-16	8.67E-15
CSAG1	0.194891319	8.40E-06	2.54E-05
CSAG2	0.148003324	0.000754	0.00164
CSAG3	0.198665816	5.55E-06	1.73E-05
CSDAP1	0.15418104	0.000446	0.001009
CSDA	0.105025427	0.017116	0.028916
CSDC2	-0.242444036	2.52E-08	1.14E-07
CSDE1	-0.015494443	0.725745	0.777554
CSE1L	0.450491434	4.19E-27	3.11E-25
CSF1R	-0.229035693	1.48E-07	5.93E-07
CSF1	-0.22932709	1.43E-07	5.73E-07
CSF2RA	-0.179736057	4.09E-05	0.000111
CSF2RB	-0.222046701	3.57E-07	1.35E-06

CSF2	-0.133907704	0.002325	0.004634
CSF3R	-0.246398637	1.46E-08	6.83E-08
CSF3	0.007678063	0.862005	0.89151
CSGALNACT1	0.143836205	0.001063	0.00225
CSGALNACT2	-0.047763514	0.279292	0.349824
CSH1	0.029079861	0.510241	0.582596
CSH2	0.029144522	0.5093	0.581808
CSK	-0.091146746	0.038666	0.060558
CSMD1	-0.143160609	0.001123	0.002367
CSMD2	0.052154982	0.237399	0.304096
CSMD3	0.039702745	0.368565	0.443254
CSN1S1	0.018950501	0.667888	0.726042
CSN2	0.086559946	0.049615	0.075981
CSN3	-0.108354389	0.013885	0.023925
CSNK1A1L	0.058855232	0.182352	0.241781
CSNK1A1P	0.028822928	0.513987	0.586391
CSNK1A1	-0.013488237	0.760087	0.806702
CSNK1D	-0.143127334	0.001126	0.002373
CSNK1E	0.14321278	0.001118	0.002359
CSNK1G1	0.020344998	0.645066	0.70557
CSNK1G2	-0.088331239	0.045113	0.069684
CSNK1G3	0.062544237	0.156397	0.211449
CSNK2A1P	0.206162535	2.38E-06	7.86E-06
CSNK2A1	0.211459319	1.29E-06	4.43E-06
CSNK2A2	0.103496509	0.018808	0.031509
CSNK2B	0.204072704	3.02E-06	9.80E-06
CSPG4PY2	0.001855939	0.966486	0.974334
CSPG4	-0.047879308	0.278127	0.348692
CSPG5	-0.214102136	9.39E-07	3.32E-06
CSPP1	0.06463621	0.142976	0.195486
CSRNP1	-0.280798046	8.71E-11	5.66E-10
CSRNP2	0.097465329	0.026985	0.043763
CSRNP3	0.057374332	0.193622	0.254714
CSRP1	-0.169970601	0.000106	0.000269
CSRP2BP	0.001022477	0.981533	0.98622
CSRP2	0.200762916	4.39E-06	1.39E-05
CSRP3	0.070622468	0.109425	0.154081
CST11	-0.012398556	0.778944	0.822219
CST1	-0.039195271	0.374723	0.44955
CST2	-0.121449325	0.005786	0.010737
CST3	-0.249446392	9.58E-09	4.60E-08
CST4	-0.006964359	0.874721	0.902197
CST5	-0.367651722	6.30E-18	1.21E-16
CST6	-0.078261274	0.075991	0.111496

CST7	-0.079269915	0.072276	0.106545
CST9L	0.090618345	0.039812	0.062192
CST9	0.068019945	0.123158	0.171152
CSTA	-0.091012282	0.038955	0.060954
CSTB	0.067358943	0.126851	0.175613
CSTF1	0.22111811	4.01E-07	1.50E-06
CSTF2T	0.054877687	0.213763	0.277737
CSTF2	0.294035651	9.93E-12	7.42E-11
CSTF3	0.329397084	1.69E-14	1.93E-13
CSTL1	0.038667747	0.38119	0.455709
CSTT	0.042437261	0.336478	0.410443
CS	0.316786422	1.81E-13	1.77E-12
CT45A1	0.021655624	0.623916	0.686682
CT45A2	0.076922195	0.081162	0.118137
CT45A3	0.06050554	0.170372	0.227999
CT45A4	0.121216239	0.005882	0.010901
CT45A5	0.064748894	0.142279	0.194626
CT45A6	0.043127561	0.32867	0.402072
CT47A11	0.054342716	0.218268	0.28269
CT47A1	0.020549904	0.64174	0.702547
CT47A2	0.022059135	0.617465	0.680932
CT47A6	0.055974129	0.20474	0.267543
CT47B1	0.028616513	0.517006	0.589197
CT62	-0.159162377	0.000287	0.000674
CTAG1B	0.134438491	0.002233	0.004465
CTAG2	0.160604115	0.000253	0.000599
CTAGE1	0.209454586	1.63E-06	5.51E-06
CTAGE4	-0.092224733	0.036413	0.057354
CTAGE5	0.242941084	2.35E-08	1.06E-07
CTAGE6	-0.090858801	0.039287	0.061421
CTAGE9	-0.075147226	0.088451	0.127493
CTBP1	-0.101441215	0.021312	0.035336
CTBP2	0.291199157	1.60E-11	1.16E-10
CTBS	0.157663364	0.000328	0.000761
CTCFL	0.170965328	9.65E-05	0.000246
CTCF	0.069311109	0.116186	0.162548
CTDP1	-0.084996727	0.053894	0.081876
CTDSP1	-0.294940044	8.52E-12	6.45E-11
CTDSP2	-0.190630276	1.33E-05	3.90E-05
CTDSPL2	0.192414659	1.10E-05	3.26E-05
CTDSPL	-0.359853172	3.44E-17	5.94E-16
CTF1	-0.34002401	2.10E-15	2.77E-14
CTGF	-0.099742172	0.023595	0.038715
CTHRC1	0.332460447	9.34E-15	1.11E-13

CTH	0.160606384	0.000253	0.000599
CTLA4	-0.111039006	0.011684	0.020432
CTNNA1	-0.067257102	0.127428	0.176301
CTNNA2	-0.02606477	0.555079	0.624608
CTNNA3	-0.09147235	0.037973	0.059576
CTNNAL1	0.101619443	0.021084	0.034985
CTNNB1	0.031255277	0.479104	0.552938
CTNNBIP1	-0.309034677	7.37E-13	6.59E-12
CTNNBL1	0.111834532	0.011094	0.019504
CTNND1	-0.045794467	0.299614	0.371047
CTNND2	-0.01870503	0.671938	0.72977
CTNS	-0.120485157	0.006189	0.011421
CTPS2	0.044815571	0.310074	0.382125
CTPS	0.317473923	1.60E-13	1.57E-12
CTR9	-0.072603386	0.099805	0.142016
CTRB1	0.025844203	0.558433	0.627887
CTRB2	-0.050262122	0.25488	0.32336
CTRC	-0.148274716	0.000737	0.001607
CTRL	-0.111428336	0.011392	0.019991
CTSA	-0.012945312	0.769465	0.814672
CTSB	0.057963383	0.18908	0.249529
CTSC	0.078957156	0.073411	0.107988
CTSD	-0.338637571	2.77E-15	3.60E-14
CTSE	-0.221310541	3.91E-07	1.47E-06
CTSF	-0.292163171	1.36E-11	9.98E-11
CTSG	-0.336835148	3.96E-15	5.01E-14
CTSH	-0.478355877	8.30E-31	1.04E-28
CTSK	-0.014599458	0.741002	0.790349
CTSL1	0.30389051	1.83E-12	1.53E-11
CTSL2	0.338056481	3.11E-15	4.01E-14
CTSL3	0.04197327	0.341792	0.416031
CTSO	-0.329606413	1.62E-14	1.86E-13
CTSS	-0.180664204	3.72E-05	0.000102
CTSW	-0.139299645	0.00153	0.003148
CTSZ	-0.204335175	2.94E-06	9.54E-06
CTTNBP2NL	-0.099090396	0.024526	0.040106
CTTNBP2	-0.302663374	2.27E-12	1.87E-11
CTTN	-0.011539548	0.793903	0.834922
CTU1	0.12817173	0.003572	0.006876
CTU2	0.141053639	0.001331	0.002766
CTXN1	0.104934775	0.017212	0.029052
CTXN2	-0.033107442	0.453431	0.527635
CTXN3	-0.103593985	0.018696	0.031353
CUBN	-0.305029517	1.50E-12	1.27E-11

CUEDC1	-0.192473255	1.09E-05	3.24E-05
CUEDC2	0.097386223	0.02711	0.043937
CUL1	0.125708805	0.004274	0.00812
CUL2	0.349158722	3.28E-16	4.88E-15
CUL3	0.002321675	0.958083	0.967864
CUL4A	0.048823325	0.26875	0.338444
CUL4B	0.223373547	3.03E-07	1.16E-06
CUL5	0.084447008	0.05547	0.083977
CUL7	-0.069049457	0.117573	0.164201
CUL9	-0.370135739	3.63E-18	7.23E-17
CUTA	-0.124287506	0.004733	0.008933
CUTC	0.11605346	0.008384	0.015097
CUX1	-0.154196969	0.000445	0.001008
CUX2	-0.156849126	0.000353	0.000813
CUZD1	0.052998521	0.229887	0.295859
CWC15	0.160331643	0.000259	0.000612
CWC22	0.097028266	0.027682	0.044784
CWC25	-0.259319935	2.33E-09	1.22E-08
CWC27	0.120116941	0.00635	0.011694
CWF19L1	0.266423276	8.11E-10	4.57E-09
CWF19L2	-0.006652875	0.880281	0.906215
CWH43	-0.108463742	0.013789	0.02378
CX3CL1	-0.319515891	1.09E-13	1.11E-12
CX3CR1	-0.415395852	6.68E-23	2.69E-21
CXADRP2	-0.080344164	0.068483	0.101516
CXADRP3	-0.038886293	0.378502	0.453296
CXADR	-0.102147363	0.020421	0.033969
CXCL10	0.120646583	0.00612	0.011304
CXCL11	0.079777148	0.070464	0.104133
CXCL12	-0.198204402	5.84E-06	1.81E-05
CXCL13	-0.012801229	0.77196	0.816572
CXCL14	-0.210494354	1.44E-06	4.92E-06
CXCL16	-0.301339587	2.85E-12	2.31E-11
CXCL17	-0.299376613	4.00E-12	3.16E-11
CXCL1	0.040919556	0.354059	0.428697
CXCL2	-0.108288236	0.013944	0.024018
CXCL3	0.043316006	0.326559	0.399832
CXCL5	0.252467765	6.25E-09	3.08E-08
CXCL6	0.047284423	0.284149	0.354973
CXCL9	0.024837853	0.573859	0.641694
CXCR1	-0.00535358	0.903534	0.924501
CXCR2P1	-0.09671831	0.028185	0.045528
CXCR2	-0.15836201	0.000309	0.000719
CXCR3	-0.121630174	0.005713	0.010614

CXCR4	-0.177581473	5.07E-05	0.000135
CXCR5	-0.212614032	1.12E-06	3.90E-06
CXCR6	-0.040159122	0.363082	0.437613
CXCR7	0.155611684	0.000393	0.000898
CXXC1	-0.127020127	0.003886	0.007435
CXXC4	-0.004325238	0.921998	0.938956
CXXC5	-0.186118646	2.13E-05	6.07E-05
CXorf1	-0.17736154	5.18E-05	0.000138
CXorf21	-0.248107922	1.15E-08	5.47E-08
CXorf22	-0.243613695	2.15E-08	9.77E-08
CXorf23	-0.300718908	3.17E-12	2.55E-11
CXorf26	0.155382591	0.000401	0.000915
CXorf27	0.040880393	0.354521	0.429126
CXorf30	-0.276255827	1.79E-10	1.11E-09
CXorf36	-0.186582049	2.03E-05	5.80E-05
CXorf38	-0.085788687	0.051689	0.078837
CXorf40A	0.03904371	0.376574	0.451365
CXorf40B	0.152326723	0.000523	0.001169
CXorf41	-0.211045556	1.35E-06	4.64E-06
CXorf42	-0.293496766	1.09E-11	8.09E-11
CXorf48	0.04798247	0.277092	0.347612
CXorf49B	-0.05375843	0.223266	0.288303
CXorf50B	-0.274973496	2.18E-10	1.34E-09
CXorf51	0.069652604	0.114394	0.160345
CXorf56	0.055761138	0.206471	0.269453
CXorf57	-0.260348057	2.00E-09	1.06E-08
CXorf58	-0.013269433	0.763862	0.809978
CXorf59	-0.246528073	1.44E-08	6.72E-08
CXorf61	0.204198068	2.98E-06	9.68E-06
CXorf64	-0.174101793	7.13E-05	0.000186
CXorf65	-0.222282889	3.47E-07	1.31E-06
CXorf66	-8.19E-06	0.999852	0.999963
CYB561D1	-0.245029496	1.77E-08	8.15E-08
CYB561D2	0.029154738	0.509152	0.581684
CYB561	-0.056919445	0.197184	0.258855
CYB5A	-0.236866118	5.33E-08	2.28E-07
CYB5B	0.182604502	3.06E-05	8.46E-05
CYB5D1	-0.174007534	7.20E-05	0.000187
CYB5D2	-0.212030623	1.20E-06	4.16E-06
CYB5R1	-0.241776881	2.76E-08	1.23E-07
CYB5R2	-0.139200526	0.001542	0.003169
CYB5R3	-0.272393073	3.26E-10	1.94E-09
CYB5R4	0.152111397	0.000533	0.001189
CYB5RL	-0.229213564	1.45E-07	5.80E-07

CYBASC3	-0.198497262	5.65E-06	1.76E-05
CYBA	-0.038928713	0.377982	0.452781
CYBB	-0.160912318	0.000246	0.000584
CYBRD1	-0.378244483	5.82E-19	1.31E-17
CYC1	0.366592155	7.95E-18	1.51E-16
CYCSP52	-0.118912154	0.006901	0.012628
CYCS	0.476622695	1.44E-30	1.76E-28
CYFIP1	0.044684381	0.311494	0.383502
CYFIP2	-0.37118993	2.87E-18	5.80E-17
CYGB	-0.207311855	2.09E-06	6.94E-06
CYHR1	-0.047063509	0.286407	0.357414
CYLC1	0.004081701	0.926377	0.942416
CYLC2	-0.032954525	0.455521	0.529481
CYLD	-0.322692007	6.05E-14	6.32E-13
CYMP	-0.187501595	1.85E-05	5.31E-05
CYP11A1	-0.029694267	0.501341	0.574291
CYP11B1	-0.038390443	0.384617	0.45927
CYP11B2	-0.014430666	0.743891	0.792923
CYP17A1	-0.22448689	2.63E-07	1.02E-06
CYP19A1	0.06340816	0.150744	0.204747
CYP1A1	-0.055162809	0.21139	0.275028
CYP1A2	-0.183449853	2.81E-05	7.81E-05
CYP1B1	-0.086047536	0.050985	0.077864
CYP20A1	-0.261627302	1.66E-09	8.88E-09
CYP21A2	-0.399639923	3.60E-21	1.13E-19
CYP24A1	0.307019148	1.05E-12	9.20E-12
CYP26A1	0.019324473	0.661736	0.720531
CYP26B1	0.00758452	0.86367	0.892839
CYP26C1	-0.040071234	0.364134	0.438796
CYP27A1	-0.365248149	1.07E-17	1.99E-16
CYP27B1	0.011770482	0.789873	0.831384
CYP27C1	0.071884473	0.103215	0.146227
CYP2A13	-0.174679584	6.74E-05	0.000176
CYP2A6	-0.145693256	0.000913	0.001955
CYP2A7	-0.074449638	0.091456	0.131323
CYP2B6	-0.114540545	0.009279	0.016575
CYP2B7P1	-0.375775713	1.02E-18	2.22E-17
CYP2C18	-0.078802512	0.073978	0.10873
CYP2C19	-0.047175488	0.285261	0.356184
CYP2C8	-0.174196248	7.07E-05	0.000184
CYP2C9	-0.103955812	0.018285	0.03072
CYP2D6	-0.143646602	0.00108	0.002283
CYP2D7P1	-0.249365691	9.69E-09	4.65E-08
CYP2E1	-0.076794063	0.081671	0.118749

CYP2F1	-0.242287698	2.57E-08	1.16E-07
CYP2J2	-0.069789301	0.113683	0.159505
CYP2R1	-0.16699643	0.00014	0.000349
CYP2S1	-0.238285273	4.41E-08	1.91E-07
CYP2U1	-0.323135113	5.57E-14	5.84E-13
CYP2W1	-0.11029264	0.012262	0.021348
CYP39A1	-0.181705222	3.35E-05	9.21E-05
CYP3A43	-0.072216136	0.10163	0.144264
CYP3A4	-0.061148802	0.165865	0.222714
CYP3A5	-0.077388977	0.079328	0.115814
CYP3A7	-0.250226615	8.58E-09	4.15E-08
CYP46A1	-0.26071266	1.90E-09	1.01E-08
CYP4A11	-0.252737837	6.02E-09	2.97E-08
CYP4A22	-0.315789298	2.18E-13	2.10E-12
CYP4B1	-0.467196229	2.77E-29	2.72E-27
CYP4F11	0.076362133	0.083407	0.120965
CYP4F12	-0.229465321	1.40E-07	5.64E-07
CYP4F22	-0.164513919	0.000177	0.000432
CYP4F2	0.110923465	0.011772	0.020562
CYP4F3	0.19259039	1.08E-05	3.21E-05
CYP4F8	-0.043930119	0.319741	0.392494
CYP4V2	-0.300295664	3.41E-12	2.73E-11
CYP4X1	-0.432754117	6.42E-25	3.54E-23
CYP4Z1	-0.436891062	2.04E-25	1.22E-23
CYP4Z2P	-0.458587517	3.81E-28	3.16E-26
CYP51A1	0.164027182	0.000185	0.00045
CYP7A1	-0.274927973	2.20E-10	1.35E-09
CYP7B1	-0.110294754	0.012261	0.021347
CYP8B1	-0.039002397	0.377079	0.451862
CYR61	-0.163844989	0.000188	0.000457
CYS1	-0.304502409	1.64E-12	1.39E-11
CYSLTR1	-0.269215104	5.31E-10	3.07E-09
CYSLTR2	-0.154155644	0.000447	0.001011
CYTH1	-0.366522735	8.07E-18	1.53E-16
CYTH2	-0.186110965	2.13E-05	6.07E-05
CYTH3	-0.2844605	4.83E-11	3.25E-10
CYTH4	-0.178624193	4.57E-05	0.000123
CYTIP	-0.14802846	0.000752	0.001638
CYTL1	-0.024345113	0.581484	0.648444
CYTSA	-0.186053204	2.15E-05	6.10E-05
CYTSB	0.17229548	8.50E-05	0.000218
CYYR1	-0.238633125	4.21E-08	1.83E-07
CYorf15A	0.160915136	0.000246	0.000584
CYorf15B	0.109242956	0.013119	0.022725

D2HGDH	-0.264159269	1.14E-09	6.27E-09
D4S234E	-0.306250337	1.21E-12	1.04E-11
DAAM1	-0.001904936	0.965602	0.973688
DAAM2	-0.3973622	6.30E-21	1.88E-19
DAB1	-0.052906914	0.230695	0.296707
DAB2IP	-0.275826697	1.91E-10	1.18E-09
DAB2	-0.226626168	2.01E-07	7.90E-07
DACH1	-0.290765542	1.72E-11	1.24E-10
DACH2	-0.171971092	8.77E-05	0.000225
DACT1	0.065885339	0.135394	0.186253
DACT2	-0.111995904	0.010977	0.019316
DACT3	-0.177525682	5.10E-05	0.000136
DAD1L	-0.119594928	0.006584	0.012094
DAD1	0.348488968	3.76E-16	5.54E-15
DAG1	0.05215338	0.237413	0.304096
DAGLA	-0.04774903	0.279438	0.349963
DAGLB	-0.230856729	1.17E-07	4.77E-07
DAK	-0.057520989	0.192484	0.253433
DALRD3	-0.1041137	0.018108	0.030454
DAND5	-0.106970499	0.015156	0.025911
DAO	-0.152143609	0.000531	0.001186
DAP3	0.300001686	3.59E-12	2.86E-11
DAPK1	-0.327549234	2.41E-14	2.68E-13
DAPK2	-0.496255256	2.26E-33	4.66E-31
DAPK3	-0.063115435	0.152642	0.206932
DAPL1	-0.09051614	0.040037	0.062521
DAPP1	-0.174400437	6.93E-05	0.000181
DAP	-0.046027463	0.29716	0.368509
DARC	-0.355668756	8.39E-17	1.35E-15
DARS2	0.471927003	6.36E-30	6.91E-28
DARS	0.422572465	1.01E-23	4.59E-22
DAXX	0.074420262	0.091584	0.131442
DAZ1	0.027221143	0.537657	0.60843
DAZ2	0.033194961	0.452237	0.526521
DAZ3	0.020708462	0.639171	0.70031
DAZAP1	0.080585035	0.067656	0.100408
DAZAP2	0.110917075	0.011777	0.020569
DAZL	0.005442196	0.901946	0.923334
DBC1	-0.234434298	7.35E-08	3.08E-07
DBF4B	0.173230721	7.76E-05	0.000201
DBF4	0.5230277	1.67E-37	7.61E-35
DBH	-0.2924712	1.29E-11	9.50E-11
DBI	0.021669319	0.623697	0.686554
DBN1	0.20448404	2.89E-06	9.40E-06

DBNDD1	0.193202732	1.01E-05	3.02E-05
DBNDD2	-0.05542589	0.209217	0.272584
DBNL	-0.112991856	0.010282	0.018211
DBP	-0.368595672	5.11E-18	9.99E-17
DBR1	0.095551569	0.030151	0.048406
DBT	-0.026037668	0.55549	0.625036
DBX1	-0.002496694	0.954926	0.965359
DBX2	0.070440401	0.110345	0.155201
DCAF10	0.19275447	1.06E-05	3.16E-05
DCAF11	-0.180375354	3.83E-05	0.000104
DCAF12L1	0.03430827	0.437209	0.511557
DCAF12L2	0.046949544	0.287577	0.358538
DCAF12	0.07462301	0.090701	0.130343
DCAF13	0.479957475	4.96E-31	6.44E-29
DCAF15	-0.14750833	0.000786	0.001703
DCAF16	0.064601376	0.143192	0.195755
DCAF17	0.178637572	4.56E-05	0.000123
DCAF4L1	-0.098524741	0.025359	0.04135
DCAF4L2	0.104168883	0.018047	0.030358
DCAF4	0.054520208	0.216766	0.281144
DCAF5	-0.195874162	7.54E-06	2.30E-05
DCAF6	-0.142949244	0.001142	0.002405
DCAF7	0.001017052	0.981631	0.986269
DCAF8L1	0.078991459	0.073286	0.107828
DCAF8L2	0.121503725	0.005764	0.0107
DCAF8	-0.278561522	1.24E-10	7.89E-10
DCAKD	-0.221597548	3.78E-07	1.42E-06
DCBLD1	-0.036782744	0.404852	0.479651
DCBLD2	0.087319584	0.04764	0.073158
DCC	-0.132479161	0.002592	0.00512
DCDC1	-0.060544786	0.170095	0.227735
DCDC2B	-0.268851653	5.61E-10	3.24E-09
DCDC2	-0.11693575	0.007899	0.014301
DCD	-0.058981707	0.181412	0.240759
DCHS1	-0.157974671	0.00032	0.000742
DCHS2	-0.213507784	1.01E-06	3.54E-06
DCI	0.053627658	0.224396	0.289556
DCK	0.282902547	6.21E-11	4.12E-10
DCLK1	-0.034996379	0.428065	0.502624
DCLK2	-0.291617937	1.49E-11	1.09E-10
DCLK3	0.137392664	0.001777	0.003613
DCLRE1A	0.270895011	4.11E-10	2.42E-09
DCLRE1B	0.13590977	0.001994	0.004023
DCLRE1C	0.093774792	0.03337	0.052969

DCN	-0.195815954	7.59E-06	2.31E-05
DCP1A	-0.032278497	0.464824	0.538666
DCP1B	-0.2290377	1.48E-07	5.93E-07
DCP2	-0.217444634	6.28E-07	2.28E-06
DCPS	0.102337756	0.020186	0.033601
DCST1	-0.089597689	0.042109	0.065459
DCST2	-0.177196879	5.27E-05	0.00014
DCTD	-0.026664289	0.546013	0.616593
DCTN1	-0.169451679	0.000111	0.000282
DCTN2	0.23543007	6.45E-08	2.73E-07
DCTN3	0.064869926	0.141533	0.193725
DCTN4	-0.097755249	0.026531	0.043094
DCTN5	0.203545698	3.21E-06	1.04E-05
DCTN6	0.12942672	0.003257	0.006317
DCTPP1	0.28968582	2.05E-11	1.47E-10
DCT	-0.211909496	1.22E-06	4.21E-06
DCUN1D1	0.259860494	2.15E-09	1.13E-08
DCUN1D2	-0.202718872	3.53E-06	1.13E-05
DCUN1D3	-0.15909155	0.000289	0.000678
DCUN1D4	0.007241461	0.86978	0.898412
DCUN1D5	0.449497254	5.59E-27	4.07E-25
DCXR	-0.080966128	0.066363	0.09871
DCX	-0.048968036	0.267332	0.336892
DDA1	0.052173385	0.237233	0.303943
DDAH1	-0.261519498	1.69E-09	9.02E-09
DDAH2	0.019244854	0.663044	0.721758
DDB1	0.147339631	0.000797	0.001725
DDB2	-0.240027294	3.49E-08	1.54E-07
DDC	0.166533642	0.000147	0.000363
DDHD1	-0.008906233	0.840208	0.873236
DDHD2	0.067694731	0.124964	0.173241
DDI1	-0.100085978	0.023117	0.038026
DDI2	-0.00821549	0.852453	0.883436
DDIT3	0.139353756	0.001523	0.003137
DDIT4L	0.131481726	0.002794	0.005486
DDIT4	0.105554471	0.016562	0.028069
DDN	0.238684842	4.18E-08	1.82E-07
DDOST	0.204540284	2.87E-06	9.34E-06
DDO	-0.23914231	3.94E-08	1.72E-07
DDR1	-0.235818157	6.12E-08	2.60E-07
DDR2	-0.149316684	0.000675	0.001482
DDRGK1	-0.139108924	0.001553	0.003191
DDTL	-0.031691365	0.472989	0.546733
DDT	0.059140502	0.180238	0.239486

DDX10		0.321253816	7.92E-14	8.13E-13
DDX11L2		-0.156532465	0.000363	0.000834
DDX11		0.143052265	0.001133	0.002387
DDX12		0.107736736	0.01444	0.024795
DDX17		-0.301686251	2.68E-12	2.18E-11
DDX18		0.344464559	8.58E-16	1.19E-14
DDX19A		0.047022179	0.286831	0.357742
DDX19B		-0.072097579	0.102195	0.144935
DDX1		0.31845277	1.33E-13	1.33E-12
DDX20		0.102337783	0.020186	0.033601
DDX21		0.390618646	3.22E-20	8.67E-19
DDX23		0.288113548	2.66E-11	1.86E-10
DDX24		-0.178869799	4.46E-05	0.00012
DDX25		0.055288237	0.210352	0.273856
DDX26B		-0.297867108	5.18E-12	4.03E-11
DDX27		0.170456908	0.000101	0.000258
DDX28		0.079368641	0.07192	0.106091
DDX31		-0.023196195	0.599446	0.664835
DDX39		0.336010299	4.66E-15	5.82E-14
DDX3X		-0.054220337	0.219308	0.283853
DDX3Y		0.155522939	0.000397	0.000905
DDX41		-0.014674911	0.739712	0.789268
DDX42		-0.218280566	5.67E-07	2.08E-06
DDX43		0.063975555	0.147116	0.200378
DDX46		-0.023443657	0.595556	0.66159
DDX47		0.401653781	2.19E-21	7.10E-20
DDX49		0.107115442	0.015019	0.025719
DDX4		-0.021528534	0.625954	0.68866
DDX50		0.287957582	2.73E-11	1.91E-10
DDX51		-0.071517552	0.104991	0.148445
DDX52		0.361065774	2.65E-17	4.64E-16
DDX53		0.14280632	0.001156	0.002431
DDX54		0.145038789	0.000964	0.002055
DDX55		0.241039407	3.05E-08	1.35E-07
DDX56		0.263618691	1.23E-09	6.74E-09
DDX58		0.006589743	0.881409	0.906955
DDX59		0.146200692	0.000876	0.00188
DDX5		-0.322361887	6.44E-14	6.71E-13
DDX60L		-0.073727898	0.09465	0.135366
DDX60		-0.058232659	0.187029	0.247229
DDX6		-0.086007238	0.051094	0.078007
DEAF1		-0.18995484	1.43E-05	4.18E-05
	1-Dec	0.010828434	0.806343	0.845026
DECR1		0.19664674	6.93E-06	2.12E-05

DECR2	-0.010274242	0.816073	0.853314
DEDD2	0.027377203	0.535327	0.606549
DEDD	0.171247768	9.40E-05	0.00024
DEF6	-0.308480782	8.14E-13	7.23E-12
DEF8	-0.121755783	0.005663	0.010533
DEFA1B	0.037558301	0.395012	0.469915
DEFA4	-0.003289781	0.940632	0.953222
DEFA5	0.121516749	0.005759	0.010694
DEFA6	0.094827653	0.031429	0.050219
DEFB103B	0.191553193	1.20E-05	3.56E-05
DEFB109P1B	0.068369836	0.121237	0.168811
DEFB115	-0.008742101	0.843114	0.875801
DEFB118	0.027224961	0.5376	0.6084
DEFB119	-0.0026943	0.951363	0.962292
DEFB124	-0.135264611	0.002096	0.004213
DEFB125	0.055959312	0.20486	0.267665
DEFB126	0.120263977	0.006285	0.011587
DEFB131	0.027487704	0.533681	0.604992
DEFB132	0.05862361	0.184082	0.2438
DEFB1	0.192217828	1.12E-05	3.33E-05
DEFB4A	0.084085773	0.056527	0.085427
DEGS1	0.059499644	0.177602	0.236519
DEGS2	-0.157063389	0.000346	0.000799
DEK	0.265082131	9.92E-10	5.53E-09
DEM1	-0.067781435	0.124481	0.172618
DENND1A	0.149796748	0.000649	0.001427
DENND1B	-0.034233544	0.438208	0.512457
DENND1C	-0.344355521	8.77E-16	1.22E-14
DENND2A	-0.31053147	5.64E-13	5.13E-12
DENND2C	-0.06653491	0.131575	0.181462
DENND2D	-0.174648212	6.76E-05	0.000177
DENND3	-0.33811502	3.07E-15	3.97E-14
DENND4A	-0.08957481	0.042161	0.06552
DENND4B	-0.13842284	0.001639	0.003353
DENND4C	-0.112745596	0.01045	0.018475
DENND5A	-0.110214031	0.012325	0.021446
DENND5B	0.049846752	0.258832	0.327605
DENR	0.495073104	3.38E-33	6.75E-31
DEPDC1B	0.495981588	2.48E-33	5.01E-31
DEPDC1	0.572998306	2.84E-46	6.30E-43
DEPDC4	0.146912943	0.000826	0.001783
DEPDC5	-0.228204697	1.65E-07	6.54E-07
DEPDC6	-0.200186655	4.69E-06	1.48E-05
DEPDC7	0.077406688	0.079259	0.11573

DERA	0.36802288	5.80E-18	1.13E-16
DERL1	0.32096433	8.36E-14	8.55E-13
DERL2	0.185022384	2.39E-05	6.72E-05
DERL3	-0.084604687	0.055014	0.083375
DES	-0.334298489	6.53E-15	7.97E-14
DET1	-0.153393055	0.000477	0.001073
DEXI	-0.251312747	7.36E-09	3.60E-08
DFFA	0.270222666	4.55E-10	2.66E-09
DFFB	-0.115718551	0.008576	0.015398
DFNA5	-0.11269749	0.010484	0.018527
DFNB31	-0.266921922	7.52E-10	4.26E-09
DFNB59	-0.237484878	4.91E-08	2.11E-07
DGAT1	-0.106101087	0.016006	0.027201
DGAT2L6	0.013283261	0.763623	0.809853
DGAT2	-0.034668563	0.432407	0.507037
DGCR10	-0.156262075	0.000372	0.000852
DGCR11	-0.048773636	0.269238	0.338952
DGCR14	-0.117985386	0.007354	0.013394
DGCR2	-0.254042293	5.00E-09	2.49E-08
DGCR5	-0.071667275	0.104263	0.14751
DGCR6L	-0.173316179	7.70E-05	0.000199
DGCR6	-0.223723548	2.90E-07	1.11E-06
DGCR8	-0.187301855	1.88E-05	5.41E-05
DGCR9	-0.131219731	0.002849	0.005585
DGKA	-0.156365307	0.000368	0.000845
DGKB	0.045193254	0.30601	0.377727
DGKD	-0.223372933	3.03E-07	1.16E-06
DGKE	-0.115249442	0.00885	0.015855
DGKG	0.122014877	0.005561	0.010361
DGKH	-0.012300841	0.780642	0.823532
DGKI	0.170689915	9.91E-05	0.000252
DGKK	0.027614844	0.531789	0.603258
DGKQ	-0.149530341	0.000663	0.001458
DGKZ	-0.079303021	0.072156	0.106393
DGUOK	0.247579417	1.24E-08	5.86E-08
DHCR24	-0.19866173	5.55E-06	1.73E-05
DHCR7	0.184710672	2.47E-05	6.93E-05
DHDDS	-0.144532326	0.001004	0.002136
DHDH	-0.260587501	1.93E-09	1.02E-08
DHDPSL	-0.027361019	0.535569	0.606705
DHFRL1	-0.152804753	0.000502	0.001125
DHFR	0.229248669	1.44E-07	5.78E-07
DHH	-0.216073508	7.41E-07	2.66E-06
DHODH	0.01841418	0.676749	0.734237

DHPS	0.008143502	0.853731	0.884394
DHRS11	-0.075434045	0.087238	0.125946
DHRS12	-0.26814101	6.25E-10	3.59E-09
DHRS13	-0.051136203	0.246699	0.314408
DHRS1	-0.294494518	9.19E-12	6.92E-11
DHRS2	0.04711454	0.285884	0.356873
DHRS3	-0.249974528	8.89E-09	4.29E-08
DHRS4L1	-0.06147128	0.16364	0.220036
DHRS4L2	0.011077239	0.801985	0.841297
DHRS4	-0.026106353	0.554448	0.624074
DHRS7B	-0.135002473	0.002138	0.004293
DHRS7C	0.017856164	0.686015	0.742719
DHRS7	0.035526934	0.421091	0.495717
DHRS9	-0.104365454	0.017829	0.030015
DHRSX	0.14340552	0.001101	0.002325
DHTKD1	0.117212668	0.007752	0.014051
DHX15	0.226538208	2.03E-07	7.99E-07
DHX16	-0.015894226	0.718964	0.77162
DHX29	-0.042041612	0.341006	0.415156
DHX30	-0.016560989	0.707704	0.762146
DHX32	0.051572477	0.242686	0.309976
DHX33	0.182984231	2.94E-05	8.17E-05
DHX34	-0.01368899	0.756628	0.803647
DHX35	-0.004809257	0.913302	0.932238
DHX36	0.295955584	7.18E-12	5.50E-11
DHX37	0.242445342	2.52E-08	1.14E-07
DHX38	-0.089620642	0.042056	0.065392
DHX40P1	0.020889057	0.63625	0.697547
DHX40	-0.110857815	0.011822	0.020641
DHX57	0.16200083	0.000223	0.000534
DHX58	-0.343728219	9.96E-16	1.37E-14
DHX8	0.00448958	0.919044	0.936521
DHX9	0.185214702	2.34E-05	6.60E-05
DIABLO	0.364022039	1.40E-17	2.55E-16
DIAPH1	-0.029808383	0.499697	0.572704
DIAPH2	-0.127316787	0.003803	0.007285
DIAPH3	0.438154702	1.43E-25	8.77E-24
DICER1	-0.016129523	0.714983	0.76804
DIDO1	-0.26010969	2.07E-09	1.09E-08
DIMT1L	0.232620574	9.32E-08	3.85E-07
DIO1	-0.216996509	6.63E-07	2.40E-06
DIO2	0.18873721	1.62E-05	4.70E-05
DIO3OS	-0.246403351	1.46E-08	6.83E-08
DIO3	-0.181290523	3.50E-05	9.58E-05

DIP2A	-0.267447599	6.95E-10	3.96E-09
DIP2B	0.192174565	1.13E-05	3.34E-05
DIP2C	-0.166165776	0.000152	0.000375
DIRAS1	-0.088283854	0.045229	0.069836
DIRAS2	-0.075288251	0.087853	0.126742
DIRAS3	-0.325893477	3.30E-14	3.62E-13
DIRC1	0.172857089	8.05E-05	0.000208
DIRC2	-0.073981117	0.093519	0.133882
DIRC3	0.003243577	0.941464	0.953679
DIS3L2	-0.151154563	0.000578	0.001282
DIS3L	-0.121364164	0.005821	0.010795
DIS3	0.09532672	0.030543	0.048937
DISC1	-0.150936062	0.000589	0.001304
DISC2	-0.010875269	0.805522	0.844448
DISP1	-0.365300583	1.06E-17	1.97E-16
DISP2	-0.000505126	0.990876	0.993311
DIXDC1	-0.195763855	7.64E-06	2.32E-05
DKC1	0.470635191	9.53E-30	1.00E-27
DKFZP434H168	-0.029358309	0.506197	0.578958
DKFZP434K028	-0.052225513	0.236764	0.30342
DKFZP434L187	-0.030850557	0.484817	0.558532
DKFZP586I1420	-0.263965532	1.17E-09	6.44E-09
DKFZP686I15217	-0.235742452	6.19E-08	2.62E-07
DKFZp434J0226	-0.073257885	0.096778	0.138122
DKFZp434L192	-0.011701027	0.791085	0.832451
DKFZp566F0947	-0.065251157	0.139204	0.19089
DKFZp686A1627	0.113348771	0.010043	0.017826
DKFZp686O24166	0.017226636	0.696527	0.752293
DKFZp761E198	-0.112273843	0.010779	0.019008
DKFZp779M0652	-0.06933262	0.116072	0.162412
DKK1	0.144207623	0.001031	0.002189
DKK2	-0.152935826	0.000496	0.001113
DKK3	-0.167608106	0.000133	0.000331
DKK4	-0.002375795	0.957107	0.967172
DKKL1	-0.066812398	0.129969	0.179507
DLAT	0.357898787	5.23E-17	8.74E-16
DLC1	-0.425266305	4.92E-24	2.37E-22
DLD	0.298837614	4.39E-12	3.44E-11
DLEC1	-0.427565266	2.65E-24	1.34E-22
DLEU1	0.247603128	1.24E-08	5.84E-08
DLEU2L	-0.164309832	0.00018	0.00044
DLEU2	0.094145889	0.032674	0.051998
DLEU7	-0.010917656	0.804779	0.84383
DLG1	-0.03514609	0.42609	0.500746

DLG2	-0.112482861	0.010632	0.018766
DLG3	-0.151145044	0.000579	0.001283
DLG4	-0.2148008	8.64E-07	3.06E-06
DLG5	-0.003378151	0.93904	0.951947
DLGAP1	-0.161368392	0.000236	0.000562
DLGAP2	-0.265960819	8.69E-10	4.88E-09
DLGAP3	-0.115104352	0.008936	0.016001
DLGAP4	-0.030375888	0.491565	0.56484
DLGAP5	0.598126416	2.78E-51	1.11E-47
DLK1	-0.097357161	0.027156	0.043997
DLK2	0.061944982	0.160412	0.216234
DLL1	-0.155315256	0.000404	0.00092
DLL3	0.229996546	1.31E-07	5.30E-07
DLL4	-0.0422287	0.33886	0.412921
DLST	0.225172394	2.42E-07	9.36E-07
DLX1	0.079273544	0.072262	0.106534
DLX2	0.144246806	0.001028	0.002183
DLX3	-0.302840561	2.20E-12	1.82E-11
DLX4	-0.196134574	7.33E-06	2.24E-05
DLX5	-0.017651144	0.689432	0.745893
DLX6AS	-0.004005702	0.927744	0.943414
DLX6	0.033804393	0.443975	0.51823
DMAP1	-0.213246609	1.04E-06	3.64E-06
DMBT1	-0.307699646	9.35E-13	8.23E-12
DMBX1	-0.028648123	0.516543	0.588736
DMC1	0.148912085	0.000699	0.00153
DMD	-0.260608617	1.93E-09	1.02E-08
DMGDH	0.00473229	0.914684	0.933311
DMKN	-0.12701919	0.003887	0.007435
DMP1	0.105048492	0.017091	0.02888
DMPK	-0.115521335	0.00869	0.015588
DMRT1	0.055572722	0.208011	0.271232
DMRT2	-0.275827123	1.91E-10	1.18E-09
DMRT3	0.000194905	0.996479	0.997577
DMRTA1	-0.151050132	0.000583	0.001292
DMRTA2	-0.102821212	0.019601	0.03273
DMRTB1	0.03640442	0.409705	0.484423
DMRTC1B	-0.312713157	3.81E-13	3.55E-12
DMRTC1	-0.135091443	0.002124	0.004267
DMRTC2	0.09141737	0.038089	0.059723
DMTF1	-0.227988286	1.69E-07	6.71E-07
DMWD	-0.028931605	0.512401	0.584681
DMXL1	-0.304866784	1.54E-12	1.31E-11
DMXL2	-0.140131172	0.001432	0.002961

DNA2	0.393030552	1.80E-20	5.01E-19
DNAH10	-0.26690835	7.54E-10	4.27E-09
DNAH11	-0.282589301	6.53E-11	4.32E-10
DNAH12	-0.286251776	3.61E-11	2.48E-10
DNAH14	0.217317722	6.38E-07	2.31E-06
DNAH17	-0.149620912	0.000658	0.001447
DNAH1	-0.385182104	1.16E-19	2.91E-18
DNAH2	-0.244601111	1.88E-08	8.62E-08
DNAH3	-0.125273233	0.00441	0.008361
DNAH5	-0.239969872	3.52E-08	1.55E-07
DNAH6	-0.374981697	1.22E-18	2.63E-17
DNAH7	-0.289402899	2.15E-11	1.53E-10
DNAH8	-0.258283834	2.71E-09	1.40E-08
DNAH9	-0.274133537	2.49E-10	1.51E-09
DNAI1	-0.218844373	5.30E-07	1.95E-06
DNAI2	-0.265042845	9.98E-10	5.55E-09
DNAJA1	0.347314494	4.79E-16	6.96E-15
DNAJA2	0.155019871	0.000414	0.000943
DNAJA3	0.175745896	6.08E-05	0.00016
DNAJA4	-0.128915619	0.003382	0.00654
DNAJB11	0.26082205	1.87E-09	9.91E-09
DNAJB12	-0.102563136	0.019911	0.033188
DNAJB13	-0.202194177	3.74E-06	1.20E-05
DNAJB14	0.058457898	0.185327	0.245157
DNAJB1	0.07778116	0.077813	0.11381
DNAJB2	-0.266491221	8.03E-10	4.53E-09
DNAJB3	0.102436303	0.020066	0.033428
DNAJB4	0.227392405	1.83E-07	7.21E-07
DNAJB5	0.003593519	0.935162	0.94906
DNAJB6	0.186006353	2.16E-05	6.12E-05
DNAJB7	-0.129948242	0.003133	0.006097
DNAJB8	-0.049294602	0.264151	0.333408
DNAJB9	0.084014992	0.056736	0.085711
DNAJC10	0.353118706	1.44E-16	2.23E-15
DNAJC11	0.075282906	0.087876	0.126756
DNAJC12	0.135440608	0.002067	0.004162
DNAJC13	0.01017954	0.817738	0.854548
DNAJC14	0.28700027	3.19E-11	2.21E-10
DNAJC15	0.133228047	0.002449	0.004859
DNAJC16	-0.070505722	0.110014	0.154769
DNAJC17	-0.151108353	0.00058	0.001286
DNAJC18	-0.096520953	0.02851	0.045985
DNAJC19	0.059754341	0.17575	0.234365
DNAJC1	0.156085669	0.000377	0.000864

DNAJC21	-0.021488166	0.626602	0.689296
DNAJC22	0.193033134	1.03E-05	3.07E-05
DNAJC24	0.076487758	0.082899	0.120342
DNAJC25-GNG10	0.109090284	0.013248	0.022911
DNAJC25	0.173626802	7.47E-05	0.000194
DNAJC27	-0.344556145	8.42E-16	1.18E-14
DNAJC28	-0.282881283	6.23E-11	4.13E-10
DNAJC2	0.278493949	1.26E-10	7.97E-10
DNAJC30	-0.05213289	0.237598	0.304274
DNAJC3	-0.006661482	0.880128	0.906113
DNAJC4	-0.212218862	1.18E-06	4.07E-06
DNAJC5B	-0.141663397	0.001267	0.002647
DNAJC5G	-0.123124784	0.005142	0.009643
DNAJC5	-0.073802794	0.094315	0.134915
DNAJC6	0.102048784	0.020543	0.034158
DNAJC7	0.12260637	0.005334	0.009972
DNAJC8	0.065869802	0.135486	0.186341
DNAJC9	0.423435455	8.03E-24	3.72E-22
DNAL1	-0.00127201	0.977027	0.983027
DNAL4	-0.130861454	0.002927	0.005727
DNALI1	-0.268228642	6.17E-10	3.54E-09
DNASE1L1	-0.063298624	0.151452	0.205611
DNASE1L2	-0.00844386	0.848401	0.880234
DNASE1L3	-0.281582829	7.68E-11	5.03E-10
DNASE1	0.051386641	0.24439	0.311793
DNASE2B	-0.27972069	1.03E-10	6.65E-10
DNASE2	-0.189801856	1.45E-05	4.24E-05
DND1	-0.144669351	0.000993	0.002113
DNER	0.158328457	0.00031	0.000721
DNHD1	-0.326372789	3.02E-14	3.31E-13
DNLZ	0.063142862	0.152463	0.206717
DNM1L	0.365191479	1.08E-17	2.01E-16
DNM1P35	-0.369268382	4.40E-18	8.69E-17
DNM1	-0.019661631	0.65621	0.71569
DNM2	-0.28339946	5.73E-11	3.82E-10
DNM3	-0.214473797	8.99E-07	3.18E-06
DNMBP	0.040607344	0.357747	0.432272
DNMT1	0.085651252	0.052066	0.079346
DNMT3A	0.163318658	0.000197	0.000478
DNMT3B	0.289067998	2.27E-11	1.61E-10
DNMT3L	0.076141474	0.084306	0.122144
DNPEP	0.06066349	0.169257	0.22675
DNTTIP1	0.135728988	0.002022	0.004076
DNTTIP2	0.321173526	8.04E-14	8.24E-13

DNTT	0.054768921	0.214674	0.278793
DOC2A	-0.19153877	1.20E-05	3.57E-05
DOC2B	0.042172601	0.339503	0.413578
DOCK10	-0.294101441	9.82E-12	7.35E-11
DOCK11	-0.160118483	0.000264	0.000623
DOCK1	-0.153640217	0.000467	0.001052
DOCK2	-0.272450933	3.23E-10	1.93E-09
DOCK3	-0.151905473	0.000542	0.001207
DOCK4	-0.23702823	5.22E-08	2.24E-07
DOCK5	0.032301944	0.4645	0.538352
DOCK6	-0.177630437	5.04E-05	0.000135
DOCK7	0.151134961	0.000579	0.001284
DOCK8	-0.336385315	4.33E-15	5.44E-14
DOCK9	-0.346304844	5.89E-16	8.44E-15
DOHH	-0.044304037	0.315635	0.388002
DOK1	-0.30819437	8.56E-13	7.57E-12
DOK2	-0.23843265	4.33E-08	1.88E-07
DOK3	-0.203929429	3.07E-06	9.96E-06
DOK4	-0.326354321	3.03E-14	3.32E-13
DOK5	-0.098946294	0.024736	0.040423
DOK6	-0.195189759	8.13E-06	2.47E-05
DOK7	-0.218756344	5.35E-07	1.97E-06
DOLK	0.107191014	0.014947	0.025608
DOLPP1	0.157694179	0.000328	0.000759
DOM3Z	-0.107933253	0.014262	0.024526
DONSON	0.27681989	1.64E-10	1.02E-09
DOPEY1	-0.304599985	1.62E-12	1.37E-11
DOPEY2	0.01004533	0.8201	0.856524
DOT1L	-0.108460763	0.013791	0.023782
DPAGT1	0.141277814	0.001307	0.002722
DPCD	0.134567339	0.002211	0.004424
DPCR1	-0.319635097	1.07E-13	1.08E-12
DPEP1	-0.064063497	0.14656	0.199702
DPEP2	-0.381010467	3.08E-19	7.25E-18
DPEP3	-0.002139065	0.961377	0.97041
DPF1	0.097946367	0.026236	0.042666
DPF2	0.015664485	0.722858	0.774926
DPF3	-0.102038397	0.020556	0.034177
DPH1	-0.25866303	2.56E-09	1.33E-08
DPH2	0.210245693	1.48E-06	5.05E-06
DPH3B	-0.085076702	0.053668	0.081557
DPH3	0.218024204	5.85E-07	2.14E-06
DPH5	0.225063124	2.45E-07	9.48E-07
DPM1	0.275105759	2.14E-10	1.31E-09

DPM2	-0.030890002	0.484259	0.557985
DPM3	-0.025279347	0.567066	0.635649
DPP10	-0.212221258	1.17E-06	4.07E-06
DPP3	0.238156998	4.49E-08	1.95E-07
DPP4	-0.225866156	2.21E-07	8.63E-07
DPP6	-0.25808137	2.79E-09	1.44E-08
DPP7	-0.261896703	1.59E-09	8.56E-09
DPP8	0.084730307	0.054653	0.082909
DPP9	0.053395847	0.226408	0.291776
DPPA2	0.142573069	0.001178	0.002473
DPPA3	0.012531643	0.776634	0.820213
DPPA4	0.030586081	0.488571	0.561851
DPPA5	0.058372014	0.185975	0.245981
DPRXP4	-0.221212652	3.96E-07	1.48E-06
DPRX	-0.097839305	0.026401	0.042903
DPT	-0.101617588	0.021086	0.034985
DPY19L1	0.143940452	0.001054	0.002232
DPY19L2P1	-0.042669003	0.333844	0.407628
DPY19L2P2	-0.207264339	2.10E-06	6.98E-06
DPY19L2P4	-0.178284597	4.73E-05	0.000127
DPY19L2	-0.077644554	0.078338	0.114469
DPY19L3	-0.147404341	0.000793	0.001717
DPY19L4	0.215987268	7.49E-07	2.68E-06
DPY30	0.222363301	3.43E-07	1.30E-06
DPYD	-0.209950194	1.53E-06	5.22E-06
DPYSL2	-0.479680503	5.42E-31	6.99E-29
DPYSL3	-0.013943416	0.752251	0.800127
DPYSL4	-0.029968087	0.497401	0.570465
DPYSL5	0.034940617	0.428802	0.503371
DPYS	-0.183446584	2.81E-05	7.81E-05
DQX1	0.054035768	0.220884	0.28567
DR1	0.234207414	7.57E-08	3.17E-07
DRAM1	-0.270856269	4.13E-10	2.43E-09
DRAM2	0.021790743	0.621753	0.684887
DRAP1	0.166504497	0.000147	0.000364
DRD1	-0.2358176	6.13E-08	2.60E-07
DRD2	-0.141256949	0.001309	0.002726
DRD3	-0.025362005	0.565799	0.634635
DRD4	-0.076367357	0.083386	0.120952
DRD5	-0.208092182	1.91E-06	6.40E-06
DRG1	0.336051412	4.62E-15	5.78E-14
DRG2	0.069696161	0.114167	0.160071
DRGX	-0.143450723	0.001097	0.002317
DRP2	0.274335636	2.41E-10	1.47E-09

DSC1	0.085392908	0.052782	0.08032
DSC2	0.189208922	1.54E-05	4.49E-05
DSC3	-0.011470159	0.795114	0.835888
DSCAML1	-0.23134156	1.10E-07	4.51E-07
DSCAM	0.177977297	4.87E-05	0.00013
DSCC1	0.515632656	2.52E-36	9.33E-34
DSCR10	0.021110658	0.632674	0.694257
DSCR3	-0.131712912	0.002746	0.005402
DSCR4	0.041768886	0.34415	0.418347
DSCR6	0.054563861	0.216398	0.28074
DSCR8	0.10606131	0.016046	0.027259
DSCR9	-0.000401802	0.992742	0.994633
DSEL	-0.063519561	0.150026	0.20387
DSE	0.07565	0.086335	0.124722
DSG1	0.156292795	0.000371	0.00085
DSG2	0.286740991	3.33E-11	2.30E-10
DSG3	0.048536343	0.271578	0.34151
DSG4	0.205623855	2.53E-06	8.32E-06
DSN1	0.270360399	4.46E-10	2.61E-09
DSPP	-0.030108583	0.495386	0.568578
DSP	0.228347631	1.62E-07	6.43E-07
DSTN	0.016890263	0.702167	0.757579
DSTYK	-0.17457673	6.81E-05	0.000178
DST	-0.15056726	0.000608	0.001343
DTD1	0.183472014	2.80E-05	7.80E-05
DTHD1	-0.238423825	4.33E-08	1.88E-07
DTL	0.450687816	3.95E-27	2.96E-25
DTNA	-0.097894255	0.026316	0.042775
DTNBP1	-0.216653546	6.91E-07	2.49E-06
DTNB	-0.203389592	3.27E-06	1.05E-05
DTWD1	0.068037452	0.123061	0.171041
DTWD2	-0.04469414	0.311389	0.383395
DTX1	-0.106677737	0.015438	0.026334
DTX2	-0.030451744	0.490483	0.563759
DTX3L	0.050740665	0.250378	0.31845
DTX3	-0.327067124	2.64E-14	2.93E-13
DTX4	-0.318178824	1.40E-13	1.39E-12
DTYMK	0.401147208	2.48E-21	7.99E-20
DULLARD	0.076439308	0.083095	0.120595
DUOX1	-0.361922425	2.20E-17	3.88E-16
DUOX2	-0.19271977	1.06E-05	3.17E-05
DUOXA1	-0.363964526	1.41E-17	2.57E-16
DUOXA2	-0.206873763	2.19E-06	7.28E-06
DUPD1	-0.097600673	0.026772	0.043471

DUS1L	0.007051125	0.873174	0.901074
DUS2L	0.016031981	0.716633	0.769366
DUS3L	0.064179501	0.145828	0.198909
DUS4L	0.301678956	2.69E-12	2.18E-11
DUSP10	-0.076886169	0.081305	0.118301
DUSP11	0.295714262	7.48E-12	5.72E-11
DUSP12	0.153937358	0.000455	0.001028
DUSP13	0.277253286	1.53E-10	9.57E-10
DUSP14	0.289445394	2.14E-11	1.52E-10
DUSP15	-0.223935027	2.82E-07	1.08E-06
DUSP16	-0.027799636	0.529046	0.601033
DUSP18	-0.206782036	2.22E-06	7.35E-06
DUSP19	-0.152645981	0.000509	0.001139
DUSP1	-0.199464197	5.08E-06	1.59E-05
DUSP21	-0.013938142	0.752341	0.800181
DUSP22	-0.243187777	2.28E-08	1.03E-07
DUSP23	0.029038648	0.510841	0.583134
DUSP26	-0.351143848	2.17E-16	3.28E-15
DUSP27	-0.293093914	1.16E-11	8.61E-11
DUSP28	-0.212880638	1.09E-06	3.79E-06
DUSP2	-0.140211496	0.001423	0.002944
DUSP3	-0.129958517	0.003131	0.006093
DUSP4	0.265281021	9.63E-10	5.37E-09
DUSP5P	0.133145545	0.002464	0.004887
DUSP5	0.041702393	0.34492	0.419129
DUSP6	-0.113557722	0.009905	0.01761
DUSP7	-0.12775454	0.003683	0.007072
DUSP8	-0.249269017	9.82E-09	4.71E-08
DUSP9	0.037512665	0.395587	0.470459
DUT	0.219228656	5.05E-07	1.87E-06
DUXA	-0.095777936	0.02976	0.047837
DVL1	0.014880683	0.736196	0.786399
DVL2	0.096485682	0.028568	0.046072
DVL3	-0.009910069	0.822482	0.858563
DVWA	0.166486753	0.000147	0.000365
DYDC1	-0.14254176	0.001181	0.002479
DYDC2	-0.060565749	0.169946	0.227567
DYM	0.187319172	1.88E-05	5.40E-05
DYNC1H1	0.006706718	0.87932	0.905511
DYNC1I1	0.065335734	0.138691	0.190278
DYNC1I2	0.279857504	1.01E-10	6.52E-10
DYNC1LI1	0.18557049	2.26E-05	6.38E-05
DYNC1LI2	-0.191571694	1.20E-05	3.55E-05
DYNC2H1	-0.335731161	4.93E-15	6.12E-14

DYNC2LI1	-0.189655517	1.47E-05	4.30E-05
DYNLL1	0.46116841	1.75E-28	1.55E-26
DYNLL2	-0.203391624	3.27E-06	1.05E-05
DYNLRB1	0.058922702	0.18185	0.241242
DYNLRB2	-0.242446344	2.52E-08	1.14E-07
DYNLT1	0.107704707	0.01447	0.024839
DYNLT3	0.115854492	0.008498	0.015273
DYRK1A	-0.345836913	6.48E-16	9.20E-15
DYRK1B	-0.142957893	0.001142	0.002404
DYRK2	0.076290159	0.083699	0.121327
DYRK3	-0.091000688	0.03898	0.060984
DYRK4	-0.015880243	0.719201	0.771755
DYSFIP1	0.097231409	0.027356	0.044293
DYSF	-0.062170693	0.158891	0.214414
DYTN	-0.013440759	0.760905	0.807399
DYX1C1	-0.062571498	0.156216	0.211233
DZIP1L	-0.193136159	1.02E-05	3.04E-05
DZIP1	-0.011652293	0.791935	0.833159
DZIP3	-0.220676089	4.23E-07	1.58E-06
E2F1	0.257041663	3.24E-09	1.65E-08
E2F2	0.289908397	1.98E-11	1.42E-10
E2F3	0.266492688	8.02E-10	4.53E-09
E2F4	0.119233141	0.00675	0.012378
E2F5	0.348600068	3.68E-16	5.42E-15
E2F6	0.233203773	8.63E-08	3.59E-07
E2F7	0.336741695	4.03E-15	5.09E-14
E2F8	0.358804906	4.31E-17	7.28E-16
E4F1	-0.132932556	0.002504	0.004961
EAF1	0.165408302	0.000163	0.0004
EAF2	-0.058905497	0.181978	0.241349
EAPP	0.121546846	0.005747	0.010674
EARS2	-0.017142881	0.697929	0.753617
EBAG9	0.123018499	0.005181	0.009706
EBF1	-0.133358001	0.002425	0.004815
EBF2	-0.139286738	0.001531	0.00315
EBF3	-0.203572223	3.20E-06	1.03E-05
EBF4	-0.413039269	1.23E-22	4.73E-21
EBI3	-0.147998057	0.000754	0.001641
EBNA1BP2	0.320058692	9.90E-14	1.00E-12
EBPL	0.211805637	1.23E-06	4.26E-06
EBP	0.188348203	1.69E-05	4.89E-05
ECD	0.185829895	2.20E-05	6.23E-05
ECE1	-0.09512771	0.030893	0.049439
ECE2	0.425628501	4.46E-24	2.16E-22

ECEL1	0.005789739	0.895718	0.91837
ECH1	-0.050956713	0.248364	0.316238
ECHDC1	-0.254265569	4.84E-09	2.42E-08
ECHDC2	-0.443370676	3.27E-26	2.19E-24
ECHDC3	-0.119779837	0.0065	0.011948
ECHS1	0.168645031	0.00012	0.000302
ECM1	-0.14218353	0.001215	0.002546
ECM2	-0.170341726	0.000102	0.00026
ECSCR	-0.128290068	0.003542	0.006823
ECSIT	0.03932932	0.37309	0.447914
ECT2L	-0.216183974	7.32E-07	2.62E-06
ECT2	0.514063159	4.45E-36	1.53E-33
EDA2R	-0.276126853	1.82E-10	1.13E-09
EDARADD	0.242368137	2.55E-08	1.15E-07
EDAR	-0.032979113	0.455184	0.529213
EDA	-0.267196339	7.22E-10	4.10E-09
EDC3	0.153442063	0.000475	0.001069
EDC4	-0.198165521	5.87E-06	1.82E-05
EDDM3A	-0.092313789	0.036232	0.057114
EDDM3B	-0.129019517	0.003356	0.006498
EDEM1	-0.157965798	0.00032	0.000743
EDEM2	0.094638608	0.03177	0.050708
EDEM3	0.111089909	0.011645	0.020374
EDF1	0.007543746	0.864396	0.893497
EDIL3	0.014608292	0.740851	0.79023
EDN1	-0.115101525	0.008938	0.016003
EDN2	-0.03901029	0.376983	0.451797
EDN3	-0.305834757	1.30E-12	1.12E-11
EDNRA	-0.092693429	0.035469	0.056012
EDNRB	-0.26701154	7.42E-10	4.21E-09
EEA1	-0.062867056	0.154266	0.208879
EED	0.266502661	8.01E-10	4.52E-09
EEF1A1P9	-0.008578959	0.846005	0.878165
EEF1A1	0.038740213	0.380297	0.455009
EEF1A2	0.047139604	0.285628	0.356575
EEF1B2	0.205397022	2.60E-06	8.52E-06
EEF1DP3	0.001011567	0.98173	0.986319
EEF1D	0.069430515	0.115557	0.161781
EEF1E1	0.445659452	1.70E-26	1.17E-24
EEF1G	0.216397136	7.13E-07	2.56E-06
EEF2K	-0.214170322	9.32E-07	3.29E-06
EEF2	-0.006512207	0.882794	0.908053
EEFSEC	-0.141327476	0.001302	0.002713
EEPD1	-0.072254764	0.101447	0.144055

EFCAB10	-0.14588976	0.000899	0.001925
EFCAB1	-0.22636006	2.08E-07	8.15E-07
EFCAB2	-0.050234319	0.255143	0.323632
EFCAB3	0.099765867	0.023562	0.038673
EFCAB4A	-0.182141761	3.21E-05	8.83E-05
EFCAB4B	0.100210296	0.022946	0.03777
EFCAB5	-0.176998364	5.37E-05	0.000142
EFCAB6	-0.42462894	5.84E-24	2.78E-22
EFCAB7	-0.082371262	0.061771	0.092569
EFEMP1	-0.285452249	4.11E-11	2.80E-10
EFEMP2	-0.148135129	0.000746	0.001625
EFHA1	0.129107389	0.003335	0.006461
EFHA2	-0.288969126	2.31E-11	1.63E-10
EFHB	-0.266726713	7.75E-10	4.38E-09
EFHC1	-0.296070784	7.04E-12	5.39E-11
EFHC2	-0.279116955	1.14E-10	7.25E-10
EFHD1	-0.164021754	0.000185	0.000451
EFHD2	0.068053816	0.122971	0.17094
EFNA1	-0.08372623	0.057595	0.086864
EFNA2	0.17481013	6.66E-05	0.000174
EFNA3	0.176373956	5.71E-05	0.000151
EFNA4	0.027467455	0.533982	0.605196
EFNA5	0.038998974	0.377121	0.451885
EFNB1	-0.158585813	0.000303	0.000706
EFNB2	-0.054408779	0.217708	0.282037
EFNB3	-0.112035244	0.010949	0.019277
EFR3A	0.103130273	0.019234	0.032169
EFR3B	-0.109068353	0.013267	0.022941
EFS	-0.194832574	8.45E-06	2.56E-05
EFTUD1	-0.04621731	0.295169	0.366496
EFTUD2	0.2913242	1.56E-11	1.14E-10
EGFL6	-0.110165256	0.012364	0.02151
EGFL7	-0.088973315	0.043568	0.067475
EGFL8	-0.111161242	0.011591	0.020291
EGFLAM	0.133012365	0.002489	0.004932
EGFR	-0.109413025	0.012977	0.022488
EGF	0.015384782	0.727609	0.779224
EGLN1	0.308327713	8.36E-13	7.41E-12
EGLN2	-0.242409766	2.53E-08	1.14E-07
EGLN3	0.291320748	1.56E-11	1.14E-10
EGOT	-0.214415487	9.05E-07	3.20E-06
EGR1	-0.243776386	2.10E-08	9.57E-08
EGR2	-0.201833193	3.90E-06	1.24E-05
EGR3	-0.24281627	2.40E-08	1.08E-07

EGR4	-0.079757739	0.070533	0.104222
EHBP1L1	-0.205693631	2.51E-06	8.27E-06
EHBP1	0.293136528	1.15E-11	8.55E-11
EHD1	-0.079798274	0.070389	0.104033
EHD2	-0.274307051	2.42E-10	1.48E-09
EHD3	0.024303511	0.58213	0.649092
EHD4	-0.110333895	0.01223	0.021299
EHF	-0.194798797	8.48E-06	2.57E-05
EHHADH	0.027352078	0.535702	0.606801
EHMT1	-0.06073112	0.168781	0.226173
EHMT2	0.014754594	0.73835	0.788362
EI24	0.190503247	1.35E-05	3.95E-05
EID1	-0.123410337	0.005039	0.009461
EID2B	-0.233794506	7.99E-08	3.34E-07
EID2	-0.026611961	0.546801	0.617239
EID3	0.119331805	0.006704	0.012299
EIF1AD	0.211212081	1.32E-06	4.55E-06
EIF1AX	0.115739206	0.008564	0.015379
EIF1AY	0.158700028	0.0003	0.0007
EIF1B	0.065758664	0.136148	0.187188
EIF1	0.141070884	0.001329	0.002763
EIF2AK1	0.248416885	1.11E-08	5.25E-08
EIF2AK2	0.231360635	1.10E-07	4.50E-07
EIF2AK3	0.113121335	0.010195	0.018069
EIF2AK4	0.108441171	0.013809	0.023806
EIF2A	0.220263887	4.45E-07	1.66E-06
EIF2B1	0.29899457	4.27E-12	3.36E-11
EIF2B2	0.321536884	7.51E-14	7.76E-13
EIF2B3	0.195440036	7.91E-06	2.40E-05
EIF2B4	0.066810918	0.129978	0.179507
EIF2B5	-0.020138376	0.648427	0.708611
EIF2C1	-0.250983519	7.72E-09	3.76E-08
EIF2C2	0.340009835	2.11E-15	2.78E-14
EIF2C3	0.076410717	0.08321	0.120741
EIF2C4	-0.367287054	6.82E-18	1.31E-16
EIF2S1	0.538536446	4.55E-40	3.95E-37
EIF2S2	0.431001608	1.04E-24	5.49E-23
EIF2S3	0.242081356	2.65E-08	1.19E-07
EIF3A	0.181618164	3.38E-05	9.28E-05
EIF3B	0.289151932	2.24E-11	1.59E-10
EIF3CL	-0.052898083	0.230773	0.296788
EIF3C	0.18651281	2.05E-05	5.83E-05
EIF3D	0.190705894	1.32E-05	3.87E-05
EIF3E	0.28093566	8.52E-11	5.55E-10

EIF3F	0.08983516	0.041565	0.064714
EIF3G	-0.052327916	0.235845	0.302436
EIF3H	0.322514199	6.25E-14	6.53E-13
EIF3IP1	0.152575	0.000512	0.001146
EIF3I	0.263709775	1.22E-09	6.67E-09
EIF3J	0.430779146	1.10E-24	5.80E-23
EIF3K	0.067013274	0.128816	0.178026
EIF3L	-0.015829069	0.720068	0.772473
EIF3M	0.329583523	1.63E-14	1.87E-13
EIF4A1	0.418789887	2.75E-23	1.18E-21
EIF4A2	-0.043372084	0.325932	0.399187
EIF4A3	0.460713859	2.01E-28	1.73E-26
EIF4B	0.153047529	0.000492	0.001103
EIF4E1B	-0.071368635	0.105719	0.149325
EIF4E2	0.067955569	0.123514	0.171541
EIF4E3	-0.263563891	1.24E-09	6.79E-09
EIF4EBP1	0.328625232	1.96E-14	2.22E-13
EIF4EBP2	-0.158367835	0.000309	0.000719
EIF4EBP3	-0.253181239	5.65E-09	2.80E-08
EIF4ENIF1	-0.179734288	4.09E-05	0.000111
EIF4E	0.401027825	2.56E-21	8.19E-20
EIF4G1	0.134412521	0.002237	0.004472
EIF4G2	0.153589307	0.000469	0.001056
EIF4G3	-0.027777343	0.529377	0.601233
EIF4H	0.161108617	0.000241	0.000574
EIF5A2	0.188971015	1.58E-05	4.60E-05
EIF5AL1	0.477113422	1.23E-30	1.51E-28
EIF5A	0.429817403	1.43E-24	7.39E-23
EIF5B	0.27830042	1.30E-10	8.20E-10
EIF5	0.149081808	0.000689	0.00151
EIF6	0.264238277	1.13E-09	6.20E-09
ELAC1	-0.149142814	0.000685	0.001503
ELAC2	0.061611056	0.162682	0.218866
ELANE	-0.34240018	1.30E-15	1.77E-14
ELAVL1	0.205109981	2.69E-06	8.78E-06
ELAVL2	-0.006699495	0.879449	0.905591
ELAVL3	-0.043424742	0.325345	0.398532
ELAVL4	-0.027335228	0.535954	0.606949
ELF1	-0.012949881	0.769386	0.814668
ELF2	-0.191573005	1.20E-05	3.55E-05
ELF3	-0.194919396	8.37E-06	2.53E-05
ELF4	-0.119488518	0.006632	0.012175
ELF5	-0.224906264	2.50E-07	9.66E-07
ELFN1	0.054062132	0.220658	0.285433

ELFN2	-0.201007871	4.28E-06	1.36E-05
ELK1	0.198217833	5.83E-06	1.81E-05
ELK3	0.062835908	0.154471	0.209099
ELK4	-0.000161247	0.997087	0.998086
ELL2	0.129415817	0.003259	0.006321
ELL3	0.012618907	0.77512	0.819177
ELL	-0.241255003	2.96E-08	1.32E-07
ELMO1	-0.237759308	4.73E-08	2.04E-07
ELMO2	-0.190100514	1.40E-05	4.12E-05
ELMO3	-0.1755868	6.17E-05	0.000162
ELMOD1	-0.12218477	0.005495	0.01025
ELMOD2	0.252588212	6.15E-09	3.03E-08
ELMOD3	-0.301122302	2.96E-12	2.39E-11
ELN	-0.406575934	6.39E-22	2.23E-20
ELOF1	-0.056586966	0.199818	0.261814
ELOVL1	-0.008489656	0.847588	0.879535
ELOVL2	0.073913952	0.093818	0.134291
ELOVL3	0.027931543	0.527092	0.599188
ELOVL4	0.15360277	0.000469	0.001055
ELOVL5	0.130303071	0.003052	0.005952
ELOVL6	0.285552001	4.04E-11	2.76E-10
ELOVL7	0.028264458	0.522178	0.594074
ELP2P	-0.123519716	0.005	0.009393
ELP2	0.121784501	0.005652	0.010515
ELP3	0.021648108	0.624037	0.686777
ELP4	0.107267081	0.014876	0.025498
ELSPBP1	0.062126451	0.159188	0.214772
ELTD1	0.019214299	0.663546	0.722147
EMB	-0.267262398	7.14E-10	4.06E-09
EMCN	-0.28995186	1.96E-11	1.41E-10
EMD	0.040200686	0.362585	0.437193
EME1	0.360130624	3.24E-17	5.62E-16
EME2	-0.206296167	2.35E-06	7.75E-06
EMG1	0.362160471	2.09E-17	3.70E-16
EMID1	-0.207891791	1.95E-06	6.53E-06
EMID2	-0.066017377	0.134611	0.185303
EMILIN1	-0.079960818	0.069817	0.103279
EMILIN2	-0.160095895	0.000264	0.000624
EMILIN3	-0.140934146	0.001343	0.002791
EML1	-0.132603609	0.002568	0.005075
EML2	-0.093567949	0.033763	0.053551
EML3	-0.301190677	2.93E-12	2.36E-11
EML4	-0.16531013	0.000164	0.000404
EML5	-0.205266435	2.64E-06	8.64E-06

EML6	-0.243566094	2.16E-08	9.83E-08
EMP1	-0.115140349	0.008915	0.015965
EMP2	-0.33428993	6.54E-15	7.98E-14
EMP3	-0.05779786	0.190348	0.250995
EMR1	-0.081188497	0.065618	0.09774
EMR2	0.033594005	0.446817	0.521122
EMR3	-0.206188426	2.37E-06	7.84E-06
EMR4P	-0.40799428	4.46E-22	1.59E-20
EMX1	-0.032909934	0.456131	0.530159
EMX2OS	-0.027279367	0.536788	0.607652
EMX2	0.027943908	0.526909	0.599014
EN1	0.077248402	0.079876	0.116504
EN2	-0.133492133	0.0024	0.004772
ENAH	0.157455334	0.000335	0.000774
ENAM	-0.134031916	0.002303	0.004593
ENC1	-0.253638342	5.29E-09	2.63E-08
ENDOD1	-0.228840932	1.52E-07	6.06E-07
ENDOG	0.064331419	0.144875	0.197771
ENDOU	-0.315328612	2.37E-13	2.27E-12
ENGASE	-0.377833424	6.39E-19	1.43E-17
ENG	-0.357983084	5.13E-17	8.60E-16
ENHO	-0.241199365	2.98E-08	1.33E-07
ENKUR	-0.152358609	0.000522	0.001166
ENO1	0.260895958	1.85E-09	9.82E-09
ENO2	-0.024592971	0.577643	0.645096
ENO3	0.051231797	0.245816	0.313453
ENOPH1	0.399801201	3.46E-21	1.09E-19
ENOSF1	-0.190631453	1.33E-05	3.90E-05
ENOX1	0.160020017	0.000266	0.000628
ENOX2	0.141407003	0.001293	0.002697
ENPEP	0.139296282	0.00153	0.003148
ENPP1	0.217822277	6.00E-07	2.19E-06
ENPP2	-0.129263923	0.003296	0.006389
ENPP3	-0.203511729	3.22E-06	1.04E-05
ENPP4	-0.261938292	1.58E-09	8.52E-09
ENPP5	-0.353847813	1.23E-16	1.93E-15
ENPP6	-0.218163174	5.76E-07	2.10E-06
ENPP7	-0.022707257	0.607165	0.671723
ENSA	0.060366002	0.171362	0.22917
ENTHD1	0.084429748	0.055521	0.084046
ENTPD1	-0.139439869	0.001513	0.003117
ENTPD2	-0.014741131	0.73858	0.788564
ENTPD3	-0.275968109	1.87E-10	1.16E-09
ENTPD4	-0.081632031	0.064153	0.095715

ENTPD5	0.121203154	0.005887	0.010909
ENTPD6	-0.046362856	0.29365	0.364949
ENTPD7	0.37761524	6.72E-19	1.49E-17
ENTPD8	0.050852818	0.249331	0.317267
ENY2	0.339047309	2.55E-15	3.33E-14
EOMES	-0.070178348	0.111679	0.156879
EP300	-0.156118313	0.000376	0.000861
EP400NL	-0.058919692	0.181873	0.241242
EP400	-0.021132594	0.632321	0.694022
EPAS1	-0.131740373	0.00274	0.005391
EPB41L1	-0.317464946	1.60E-13	1.57E-12
EPB41L2	-0.178026572	4.85E-05	0.00013
EPB41L3	-0.118979767	0.006869	0.012579
EPB41L4A	-0.460943376	1.87E-28	1.65E-26
EPB41L4B	-0.07776681	0.077868	0.113882
EPB41L5	-0.169778816	0.000108	0.000274
EPB41	-0.271150624	3.95E-10	2.33E-09
EPB42	-0.062011855	0.15996	0.215683
EPB49	-0.264491965	1.08E-09	5.99E-09
EPC1	-0.119052772	0.006835	0.012519
EPC2	-0.099053109	0.02458	0.040185
EPCAM	0.137904691	0.001707	0.003483
EPDR1	-0.238859591	4.09E-08	1.78E-07
EPGN	0.08173672	0.063811	0.095269
EPHA10	-0.199176675	5.24E-06	1.64E-05
EPHA1	-0.123770194	0.004911	0.00924
EPHA2	-0.166541443	0.000147	0.000363
EPHA3	-0.170535381	0.000101	0.000256
EPHA4	-0.335808068	4.85E-15	6.04E-14
EPHA5	-0.110927698	0.011768	0.020562
EPHA6	0.162705552	0.000209	0.000504
EPHA7	-0.005171871	0.906793	0.927252
EPHA8	-0.025310001	0.566596	0.635208
EPHB1	0.047270005	0.284296	0.355134
EPHB2	0.058057789	0.188359	0.248822
EPHB3	-0.1621473	0.00022	0.000528
EPHB4	-0.027824471	0.528678	0.600751
EPHB6	-0.315091753	2.47E-13	2.37E-12
EPHX1	-0.15458064	0.00043	0.000977
EPHX2	-0.299598056	3.85E-12	3.05E-11
EPHX3	-0.054209483	0.219401	0.283917
EPHX4	0.161286824	0.000237	0.000566
EPM2AIP1	-0.328646545	1.95E-14	2.21E-13
EPM2A	-0.243307437	2.24E-08	1.02E-07

EPN1	-0.053553433	0.225039	0.290273
EPN2	-0.215760796	7.70E-07	2.75E-06
EPN3	0.06381444	0.148139	0.20158
EPOR	-0.316136586	2.04E-13	1.98E-12
EPO	-0.017125636	0.698218	0.753889
EPPK1	0.004425875	0.920189	0.937544
EPR1	0.492763911	7.35E-33	1.31E-30
EPRS	0.264210702	1.13E-09	6.23E-09
EPS15L1	-0.082154097	0.062463	0.093473
EPS15	-0.188944354	1.59E-05	4.61E-05
EPS8L1	-0.180261024	3.88E-05	0.000105
EPS8L2	-0.274957243	2.19E-10	1.34E-09
EPS8L3	0.199376752	5.13E-06	1.61E-05
EPS8	-0.070520727	0.109938	0.154673
EPSTI1	0.081399222	0.064919	0.096727
EPT1	0.471395811	7.52E-30	8.03E-28
EPX	-0.289392204	2.15E-11	1.53E-10
EPYC	0.14283347	0.001153	0.002426
ERAL1	0.200959606	4.30E-06	1.36E-05
ERAP1	-0.085023631	0.053818	0.081772
ERAP2	-0.027829649	0.528601	0.600698
ERAS	-0.162998734	0.000203	0.000491
ERBB2IP	-0.085941452	0.051273	0.078268
ERBB2	-0.162429211	0.000214	0.000516
ERBB3	-0.17072307	9.88E-05	0.000252
ERBB4	-0.405768352	7.83E-22	2.70E-20
ERC1	0.061811359	0.161318	0.217235
ERC2	-0.11657947	0.008092	0.014624
ERCC1	0.069374267	0.115853	0.16215
ERCC2	0.015102679	0.73241	0.783359
ERCC3	0.090019773	0.041146	0.064127
ERCC4	-0.008411246	0.848979	0.880658
ERCC5	-0.345333335	7.19E-16	1.01E-14
ERCC6L	0.522392183	2.12E-37	9.42E-35
ERCC6	-0.078455317	0.075264	0.110511
ERCC8	0.210505677	1.44E-06	4.92E-06
EREG	0.13279322	0.002531	0.005009
ERF	0.010238242	0.816706	0.853731
ERGIC1	-0.047290185	0.28409	0.354922
ERGIC2	0.413131692	1.20E-22	4.67E-21
ERGIC3	0.114514346	0.009295	0.016598
ERG	-0.28336498	5.77E-11	3.84E-10
ERH	0.432509813	6.86E-25	3.76E-23
ERI1	0.212951521	1.08E-06	3.76E-06

ERI2	0.062688227	0.155444	0.210288
ERI3	0.058352591	0.186122	0.246159
ERICH1	-0.252132407	6.56E-09	3.22E-08
ERLEC1	0.084946923	0.054036	0.082078
ERLIN1	0.401209211	2.44E-21	7.88E-20
ERLIN2	0.093255385	0.034364	0.054393
ERMAP	-0.342555123	1.26E-15	1.72E-14
ERMN	-0.159342529	0.000283	0.000665
ERMP1	0.167774345	0.000131	0.000326
ERN1	-0.177014372	5.36E-05	0.000142
ERN2	-0.119930073	0.006433	0.011836
ERO1LB	-0.079443588	0.071651	0.105726
ERO1L	0.540691952	1.95E-40	1.77E-37
ERP27	-0.265055802	9.96E-10	5.54E-09
ERP29	0.07800944	0.076942	0.112701
ERP44	0.114099368	0.009556	0.017032
ERRFI1	0.1883327	1.69E-05	4.89E-05
ERVFRDE1	-0.421316228	1.41E-23	6.31E-22
ESAM	-0.302704835	2.25E-12	1.86E-11
ESCO1	0.093860393	0.033208	0.052738
ESCO2	0.493342547	6.05E-33	1.14E-30
ESD	0.075042303	0.088898	0.128054
ESF1	0.194683109	8.59E-06	2.60E-05
ESM1	0.259711235	2.20E-09	1.15E-08
ESPL1	0.446949181	1.17E-26	8.24E-25
ESPNL	-0.099498417	0.02394	0.039218
ESPNP	-0.202810504	3.49E-06	1.12E-05
ESPN	-0.057648537	0.191498	0.252351
ESR1	-0.099950533	0.023304	0.038303
ESR2	-0.141087645	0.001327	0.00276
ESRP1	0.113252625	0.010107	0.017927
ESRP2	-0.186490713	2.05E-05	5.85E-05
ESRRA	0.071530274	0.104929	0.148367
ESRRB	-0.13283662	0.002523	0.004994
ESRRG	-0.204979717	2.73E-06	8.90E-06
ESX1	0.143991503	0.00105	0.002224
ESYT1	0.012603962	0.775379	0.819331
ESYT2	0.009837329	0.823763	0.859721
ESYT3	-0.436978769	1.99E-25	1.19E-23
ETAA1	-0.172532112	8.31E-05	0.000214
ETF1	0.152082861	0.000534	0.001191
ETFA	0.295392089	7.89E-12	6.01E-11
ETFB	0.018447947	0.676189	0.733728
ETFDH	-0.101493022	0.021245	0.035235

ETHE1	0.149805706	0.000648	0.001426
ETNK1	0.140181089	0.001426	0.002951
ETNK2	-0.134612353	0.002203	0.00441
ETS1	-0.104971606	0.017173	0.028996
ETS2	-0.119706156	0.006533	0.012007
ETV1	-0.284316266	4.94E-11	3.32E-10
ETV2	-0.073306742	0.096555	0.137853
ETV3L	-0.050165541	0.255795	0.324377
ETV3	-0.098431094	0.025499	0.041562
ETV4	-0.055682038	0.207116	0.270207
ETV5	-0.246683668	1.41E-08	6.58E-08
ETV6	-0.033927723	0.442313	0.516622
ETV7	-0.089585053	0.042138	0.065494
EVC2	-0.128772179	0.003418	0.006606
EVC	-0.279525864	1.07E-10	6.84E-10
EVI2A	-0.110980089	0.011729	0.020499
EVI2B	-0.226894474	1.94E-07	7.65E-07
EVI5L	-0.28623763	3.62E-11	2.48E-10
EVI5	-0.179983081	3.99E-05	0.000108
EVL	-0.340218422	2.02E-15	2.67E-14
EVPLL	-0.284562965	4.75E-11	3.20E-10
EVPL	-0.318463434	1.33E-13	1.33E-12
EVX1	0.066743237	0.130368	0.180008
EVX2	-0.021957847	0.619082	0.682376
EWSR1	-0.137548496	0.001755	0.003572
EXD1	-0.092259397	0.036342	0.057267
EXD2	0.190703795	1.32E-05	3.87E-05
EXD3	-0.219597374	4.83E-07	1.79E-06
EXO1	0.527189126	3.53E-38	1.91E-35
EXOC1	0.080979288	0.066319	0.098659
EXOC2	0.067099045	0.128327	0.177434
EXOC3L2	-0.087043781	0.048349	0.074179
EXOC3L	-0.2650452	9.97E-10	5.55E-09
EXOC3	-0.101951949	0.020664	0.034342
EXOC4	0.132989371	0.002494	0.00494
EXOC5	0.394731077	1.20E-20	3.42E-19
EXOC6B	-0.026338158	0.550935	0.621135
EXOC6	0.169894546	0.000107	0.000271
EXOC7	-0.233340705	8.48E-08	3.53E-07
EXOC8	-0.150112996	0.000631	0.001392
EXOG	-0.113801958	0.009746	0.017347
EXOSC10	0.04730304	0.283959	0.354802
EXOSC1	0.218239055	5.70E-07	2.09E-06
EXOSC2	0.335520914	5.13E-15	6.37E-14

EXOSC3	0.368484009	5.24E-18	1.02E-16
EXOSC4	0.263443414	1.27E-09	6.90E-09
EXOSC5	0.140553538	0.001385	0.002872
EXOSC6	0.115936097	0.008451	0.015199
EXOSC7	0.043711299	0.322159	0.395147
EXOSC8	0.254996605	4.36E-09	2.19E-08
EXOSC9	0.164322396	0.00018	0.000439
EXPH5	-0.295611974	7.61E-12	5.81E-11
EXT1	0.09256482	0.035726	0.056391
EXT2	0.099893348	0.023384	0.038409
EXTL1	-0.112964929	0.010301	0.018241
EXTL2	0.037142717	0.400267	0.474809
EXTL3	-0.004135753	0.925405	0.941802
EYA1	-0.097742964	0.02655	0.043118
EYA2	-0.092360826	0.036136	0.056981
EYA3	-0.031589783	0.47441	0.54809
EYA4	-0.054900935	0.213569	0.27752
EYS	0.121673265	0.005696	0.010587
EZH1	-0.514775243	3.44E-36	1.23E-33
EZH2	0.327523882	2.42E-14	2.69E-13
EZR	-0.174085571	7.15E-05	0.000186
F10	-0.21401751	9.49E-07	3.35E-06
F11R	-0.189713627	1.46E-05	4.28E-05
F11	-0.390948555	2.97E-20	8.05E-19
F12	0.319188531	1.16E-13	1.17E-12
F13A1	-0.139750846	0.001476	0.003046
F13B	0.180096079	3.94E-05	0.000107
F2RL1	-0.015492567	0.725777	0.777554
F2RL2	-0.018920492	0.668382	0.726501
F2RL3	0.009600244	0.827944	0.863092
F2R	-0.022202361	0.615183	0.678902
F2	0.267476869	6.92E-10	3.94E-09
F3	0.080633962	0.067488	0.100197
F5	0.084199542	0.056192	0.084973
F7	0.174569388	6.82E-05	0.000178
F8A1	0.014098996	0.749578	0.797879
F8	-0.355659812	8.41E-17	1.36E-15
F9	0.056981467	0.196696	0.258264
FA2H	0.050956794	0.248363	0.316238
FAAH2	-0.014678053	0.739658	0.789268
FAAH	-0.412745999	1.33E-22	5.06E-21
FABP12	-0.022849292	0.604918	0.669719
FABP1	0.089483182	0.042373	0.065798
FABP2	-0.00916149	0.835692	0.869357

FABP3	-0.213242473	1.04E-06	3.64E-06
FABP4	-0.15702921	0.000347	0.000801
FABP5L3	0.013545818	0.759094	0.805734
FABP5	0.018692553	0.672144	0.729954
FABP6	-0.073749148	0.094555	0.135249
FABP7	0.03204945	0.468	0.541803
FABP9	0.011561334	0.793522	0.83461
FADD	0.356199039	7.50E-17	1.22E-15
FADS1	0.241824291	2.74E-08	1.23E-07
FADS2	0.12206762	0.005541	0.010328
FADS3	-0.154181076	0.000446	0.001009
FADS6	-0.248694254	1.06E-08	5.07E-08
FAF1	0.133253794	0.002444	0.004851
FAF2	0.005756987	0.896305	0.91883
FAHD1	0.11855381	0.007073	0.012917
FAHD2A	0.009035701	0.837917	0.871115
FAHD2B	-0.197414987	6.37E-06	1.96E-05
FAH	-0.122204881	0.005487	0.010237
FAIM2	-0.181565914	3.40E-05	9.33E-05
FAIM3	-0.319502226	1.10E-13	1.11E-12
FAIM	0.043903358	0.320036	0.392823
FAM100A	-0.184192227	2.60E-05	7.29E-05
FAM100B	0.071880972	0.103232	0.146227
FAM101A	0.204300157	2.95E-06	9.57E-06
FAM101B	-0.043507721	0.32442	0.397461
FAM102A	-0.063478319	0.150291	0.204189
FAM102B	0.011339873	0.797391	0.837885
FAM103A1	0.29912873	4.17E-12	3.29E-11
FAM104A	0.215490296	7.95E-07	2.83E-06
FAM104B	-0.059373775	0.178522	0.23757
FAM105A	-0.157861044	0.000323	0.000749
FAM105B	-0.018519529	0.675004	0.732663
FAM106A	-0.079619216	0.071024	0.104862
FAM106C	-0.134404078	0.002239	0.004474
FAM107A	-0.388411899	5.44E-20	1.41E-18
FAM107B	0.065425239	0.13815	0.189652
FAM108A1	-0.150571862	0.000607	0.001342
FAM108B1	0.139345751	0.001524	0.003138
FAM108C1	0.011939159	0.786934	0.828902
FAM109A	-0.093776362	0.033367	0.052969
FAM109B	0.037191552	0.399647	0.474328
FAM10A4	0.118362249	0.007167	0.013072
FAM110A	-0.141045087	0.001332	0.002767
FAM110B	-0.272887043	3.02E-10	1.81E-09

FAM110C	-0.097771291	0.026506	0.043057
FAM111A	-0.283831376	5.35E-11	3.58E-10
FAM111B	0.284532186	4.77E-11	3.22E-10
FAM113A	-0.261719265	1.64E-09	8.77E-09
FAM113B	-0.026015471	0.555828	0.625275
FAM114A1	0.268620048	5.81E-10	3.35E-09
FAM114A2	-0.087709817	0.046651	0.07182
FAM115A	-0.016669145	0.705884	0.760809
FAM115C	0.001615131	0.970833	0.978075
FAM116A	-0.021996815	0.61846	0.681803
FAM116B	-0.40217865	1.92E-21	6.30E-20
FAM117A	-0.462552238	1.15E-28	1.04E-26
FAM117B	-0.109099255	0.01324	0.022899
FAM118A	-0.232158036	9.89E-08	4.08E-07
FAM118B	-0.146856613	0.00083	0.001789
FAM119A	0.288835709	2.36E-11	1.67E-10
FAM119B	-0.027260533	0.537069	0.607902
FAM120AOS	-0.032119811	0.467023	0.540838
FAM120A	0.010692074	0.808734	0.847044
FAM120B	-0.164090679	0.000184	0.000448
FAM120C	-0.099912824	0.023357	0.038373
FAM122A	-0.242292666	2.57E-08	1.16E-07
FAM122B	-0.024784024	0.574689	0.642372
FAM122C	-0.185628055	2.24E-05	6.35E-05
FAM123A	-0.036160076	0.412857	0.487574
FAM123B	-0.005167262	0.906876	0.92726
FAM123C	-0.034427865	0.435611	0.510136
FAM124A	0.008503505	0.847343	0.879371
FAM124B	-0.185454661	2.28E-05	6.45E-05
FAM125A	-0.02781447	0.528826	0.600886
FAM125B	-0.341201509	1.66E-15	2.23E-14
FAM126A	0.099948309	0.023307	0.038305
FAM126B	-0.182894745	2.97E-05	8.23E-05
FAM127A	-0.026328064	0.551088	0.621179
FAM127B	0.088202678	0.045428	0.070111
FAM127C	-0.019410808	0.660319	0.719419
FAM128A	0.188119549	1.73E-05	5.00E-05
FAM128B	0.111311871	0.011478	0.020127
FAM129A	-0.279582651	1.06E-10	6.78E-10
FAM129B	-0.128199058	0.003565	0.006863
FAM129C	-0.291856039	1.43E-11	1.05E-10
FAM131A	-0.082173727	0.0624	0.093407
FAM131B	-0.138702531	0.001603	0.003284
FAM131C	0.103309323	0.019025	0.031854

FAM132A	0.086792005	0.049004	0.075115
FAM133A	0.18393052	2.67E-05	7.47E-05
FAM133B	0.062255591	0.158321	0.213762
FAM134A	-0.120014912	0.006395	0.011771
FAM134B	-0.158487511	0.000305	0.000712
FAM134C	-0.118672531	0.007016	0.012819
FAM135A	0.049952141	0.257825	0.326579
FAM135B	-0.176642169	5.56E-05	0.000147
FAM136A	0.430644165	1.14E-24	5.97E-23
FAM136B	0.134871798	0.00216	0.004331
FAM138B	-0.166274599	0.00015	0.000372
FAM138D	-0.115721829	0.008574	0.015396
FAM138E	-0.086469618	0.049854	0.07633
FAM138F	-0.278445289	1.27E-10	8.03E-10
FAM13AOS	-0.44585256	1.61E-26	1.11E-24
FAM13A	-0.176974714	5.38E-05	0.000143
FAM13B	-0.38382423	1.60E-19	3.94E-18
FAM13C	-0.235823198	6.12E-08	2.60E-07
FAM149A	-0.301848253	2.61E-12	2.12E-11
FAM149B1	-0.008303483	0.850891	0.882138
FAM150A	-0.197499155	6.31E-06	1.95E-05
FAM150B	-0.336899028	3.91E-15	4.95E-14
FAM151A	-0.158749509	0.000298	0.000697
FAM151B	0.007055984	0.873087	0.901074
FAM153A	-0.262527878	1.45E-09	7.85E-09
FAM153B	-0.296098176	7.00E-12	5.37E-11
FAM153C	-0.287892489	2.76E-11	1.92E-10
FAM154A	-0.174287687	7.01E-05	0.000183
FAM154B	-0.239752192	3.63E-08	1.59E-07
FAM155A	-0.074166423	0.092699	0.132793
FAM155B	-0.065718342	0.136389	0.187456
FAM156A	-0.344203438	9.04E-16	1.26E-14
FAM157A	-0.124945577	0.004515	0.008546
FAM157B	-0.172578502	8.27E-05	0.000213
FAM158A	0.273237194	2.86E-10	1.72E-09
FAM159A	-0.248542024	1.09E-08	5.17E-08
FAM160A1	-0.141345055	0.0013	0.00271
FAM160A2	-0.355780553	8.20E-17	1.32E-15
FAM160B1	0.018317888	0.678344	0.735729
FAM160B2	-0.273634817	2.69E-10	1.62E-09
FAM161A	0.005948027	0.892884	0.916169
FAM161B	-0.252584344	6.15E-09	3.03E-08
FAM162A	0.27711346	1.56E-10	9.76E-10
FAM162B	-0.225362124	2.36E-07	9.15E-07

FAM163A	0.116762672	0.007992	0.014459
FAM163B	-0.026981655	0.541243	0.611898
FAM164A	-0.057959295	0.189111	0.249551
FAM164C	-0.137299778	0.00179	0.003637
FAM165B	-0.076009721	0.084846	0.122837
FAM166A	-0.226762055	1.98E-07	7.77E-07
FAM166B	-0.156650628	0.000359	0.000826
FAM167A	-0.156266712	0.000372	0.000852
FAM167B	-0.178569639	4.59E-05	0.000123
FAM168A	0.057829188	0.190108	0.250717
FAM168B	-0.039345843	0.372889	0.447727
FAM169A	0.017482272	0.692251	0.748294
FAM169B	-0.026327333	0.551099	0.621179
FAM170A	-0.126332526	0.004085	0.007788
FAM170B	0.034082201	0.440237	0.514588
FAM171A1	-0.121366978	0.00582	0.010794
FAM171A2	0.040166638	0.362992	0.437552
FAM171B	0.071664701	0.104276	0.147517
FAM172A	-0.162858867	0.000206	0.000497
FAM173A	-0.08803667	0.045837	0.070693
FAM173B	0.076259474	0.083824	0.121491
FAM174A	-0.158063966	0.000317	0.000737
FAM174B	-0.231098004	1.14E-07	4.64E-07
FAM175A	0.055965117	0.204813	0.267621
FAM175B	0.294729832	8.83E-12	6.66E-11
FAM176A	-0.163519954	0.000194	0.00047
FAM176B	0.025461739	0.564272	0.633206
FAM177A1	0.100599597	0.022418	0.03697
FAM177B	0.084850461	0.05431	0.082463
FAM178A	-0.165332072	0.000164	0.000403
FAM178B	-0.030813159	0.485347	0.558949
FAM179A	-0.320478505	9.15E-14	9.32E-13
FAM179B	-0.000499922	0.99097	0.993356
FAM180A	-0.153221239	0.000484	0.001088
FAM180B	-0.237892109	4.65E-08	2.01E-07
FAM181A	-0.184486086	2.52E-05	7.08E-05
FAM181B	-0.201680358	3.96E-06	1.26E-05
FAM182A	-0.274444952	2.37E-10	1.45E-09
FAM182B	-0.367248818	6.88E-18	1.32E-16
FAM183A	-0.20161202	4.00E-06	1.27E-05
FAM183B	-0.275092044	2.14E-10	1.31E-09
FAM184A	-0.469241586	1.47E-29	1.53E-27
FAM184B	-0.007792182	0.859975	0.889801
FAM185A	-0.010715531	0.808323	0.846746

FAM186A	-0.205201791	2.66E-06	8.70E-06
FAM186B	-0.122398071	0.005413	0.010106
FAM187B	-0.17502107	6.52E-05	0.000171
FAM188A	-0.072307324	0.101198	0.143819
FAM188B	-0.19894006	5.38E-06	1.68E-05
FAM189A1	-0.138102546	0.001681	0.003434
FAM189A2	-0.460585274	2.09E-28	1.79E-26
FAM189B	0.234489836	7.30E-08	3.06E-07
FAM18A	-0.178565669	4.60E-05	0.000123
FAM18B2	0.087881247	0.046223	0.071233
FAM18B	0.192574141	1.08E-05	3.21E-05
FAM190A	-0.206214508	2.37E-06	7.82E-06
FAM190B	-0.002397313	0.956719	0.966975
FAM192A	0.001089513	0.980322	0.9854
FAM193A	-0.212505284	1.14E-06	3.94E-06
FAM193B	-0.422241201	1.10E-23	5.00E-22
FAM194A	-0.025767846	0.559596	0.628948
FAM194B	-0.016086869	0.715704	0.768658
FAM195A	0.052277926	0.236293	0.302953
FAM195B	-0.064447451	0.14415	0.196929
FAM196A	0.230193622	1.28E-07	5.17E-07
FAM196B	0.074482315	0.091313	0.131163
FAM197Y2	0.043091113	0.329079	0.402499
FAM198A	-0.353562208	1.31E-16	2.05E-15
FAM198B	0.085890766	0.051411	0.078442
FAM199X	0.278230633	1.31E-10	8.29E-10
FAM19A1	-0.143317233	0.001109	0.002341
FAM19A2	-0.213862915	9.67E-07	3.41E-06
FAM19A3	-0.010230012	0.81685	0.853799
FAM19A4	-0.060134469	0.173013	0.231162
FAM19A5	-0.048016726	0.276749	0.347269
FAM200A	0.098819426	0.024922	0.040698
FAM200B	-0.102546179	0.019932	0.033219
FAM20A	-0.237364104	4.99E-08	2.15E-07
FAM20B	0.170294881	0.000103	0.000262
FAM20C	-0.064004688	0.146932	0.200155
FAM21A	-0.07642618	0.083148	0.120659
FAM21B	-0.146786422	0.000834	0.001799
FAM21C	-0.145979726	0.000892	0.001912
FAM22A	-0.271215075	3.91E-10	2.31E-09
FAM22D	-0.214266746	9.21E-07	3.26E-06
FAM22F	-0.157690601	0.000328	0.000759
FAM22G	-0.142520007	0.001183	0.002483
FAM23A	-0.179151333	4.34E-05	0.000117

FAM24B	0.247308225	1.29E-08	6.07E-08
FAM25A	0.176802808	5.47E-05	0.000145
FAM25B	0.113464376	0.009967	0.017705
FAM26D	0.091425802	0.038071	0.059707
FAM26E	0.046715971	0.289984	0.361157
FAM26F	0.025666404	0.561144	0.630545
FAM27A	-0.089169294	0.043105	0.066826
FAM27B	0.031940548	0.469514	0.54325
FAM27C	-0.065896345	0.135328	0.186188
FAM27L	0.004511418	0.918652	0.936169
FAM32A	0.060403034	0.171099	0.228849
FAM35A	0.25717805	3.18E-09	1.62E-08
FAM35B2	0.162147401	0.00022	0.000528
FAM35B	0.218438834	5.57E-07	2.04E-06
FAM36A	0.090281343	0.040559	0.06327
FAM38A	-0.094148257	0.03267	0.051995
FAM38B	-0.232563016	9.39E-08	3.88E-07
FAM3A	-0.037337179	0.397803	0.472531
FAM3B	-0.161861505	0.000225	0.00054
FAM3C	0.148795065	0.000706	0.001544
FAM3D	-0.234566012	7.22E-08	3.03E-07
FAM40A	-0.197137126	6.57E-06	2.02E-05
FAM40B	0.362453357	1.96E-17	3.49E-16
FAM41AY1	-0.008103186	0.854448	0.88509
FAM41C	-0.144006564	0.001048	0.002222
FAM43A	-0.179178309	4.32E-05	0.000117
FAM43B	-0.097302423	0.027243	0.044124
FAM45A	0.284814809	4.56E-11	3.08E-10
FAM45B	0.242472926	2.51E-08	1.13E-07
FAM46A	-0.062511772	0.156613	0.211712
FAM46B	-0.175871297	6.00E-05	0.000158
FAM46C	-0.236479755	5.61E-08	2.40E-07
FAM46D	0.103210414	0.01914	0.032018
FAM47A	0.067007214	0.128851	0.178061
FAM47B	-0.019936019	0.651726	0.711492
FAM47C	-0.22934674	1.42E-07	5.71E-07
FAM47E	-0.288744857	2.40E-11	1.69E-10
FAM48A	-0.162970974	0.000204	0.000492
FAM48B1	-0.028740707	0.515188	0.587527
FAM48B2	0.011917811	0.787306	0.829158
FAM49A	-0.247208587	1.31E-08	6.15E-08
FAM49B	0.317752993	1.52E-13	1.50E-12
FAM50A	0.026902556	0.54243	0.613189
FAM50B	-0.072346235	0.101014	0.143593

FAM53A	-0.120231158	0.0063	0.011611
FAM53B	-0.22657751	2.02E-07	7.95E-07
FAM53C	0.00671425	0.879185	0.905459
FAM54A	0.461517721	1.57E-28	1.40E-26
FAM54B	-0.191206743	1.25E-05	3.68E-05
FAM55A	-0.074918566	0.089427	0.128715
FAM55B	0.013316757	0.763045	0.809412
FAM55C	-0.048722111	0.269745	0.339547
FAM55D	-0.116715468	0.008018	0.014503
FAM57A	0.038141405	0.38771	0.462522
FAM57B	-0.004234794	0.923624	0.940277
FAM58A	0.151169676	0.000577	0.001281
FAM58B	0.152043472	0.000536	0.001195
FAM59A	-0.118909614	0.006902	0.012628
FAM5B	-0.000867235	0.984336	0.9883
FAM5C	-0.164007472	0.000185	0.000451
FAM60A	0.39904708	4.17E-21	1.29E-19
FAM63A	-0.112510652	0.010613	0.018736
FAM63B	-0.066105662	0.134089	0.184687
FAM64A	0.48354001	1.55E-31	2.25E-29
FAM65A	-0.175369446	6.30E-05	0.000165
FAM65B	-0.31725723	1.66E-13	1.63E-12
FAM65C	-0.009578096	0.828334	0.863365
FAM66A	-0.26811411	6.28E-10	3.60E-09
FAM66C	-0.223724502	2.90E-07	1.11E-06
FAM66D	-0.227902762	1.71E-07	6.78E-07
FAM66E	-0.167590722	0.000133	0.000331
FAM69A	0.14179299	0.001254	0.002623
FAM69B	-0.048209936	0.274818	0.345172
FAM69C	-0.01362768	0.757684	0.804512
FAM70A	-0.202638283	3.56E-06	1.14E-05
FAM70B	-0.125976528	0.004192	0.007974
FAM71A	-0.162751527	0.000208	0.000502
FAM71B	0.018367667	0.677519	0.734953
FAM71C	-0.099221272	0.024336	0.039813
FAM71D	0.155816607	0.000386	0.000884
FAM71E1	-0.064987386	0.140813	0.192818
FAM71E2	0.045336096	0.304483	0.376283
FAM71F1	0.042543621	0.335267	0.409191
FAM71F2	-0.165783564	0.000157	0.000387
FAM72A	0.469351365	1.42E-29	1.49E-27
FAM72B	0.481125717	3.40E-31	4.66E-29
FAM72D	0.487095973	4.85E-32	7.69E-30
FAM73A	0.074294096	0.092137	0.132102

FAM73B	-0.262522344	1.45E-09	7.86E-09
FAM74A1	-0.191436066	1.22E-05	3.60E-05
FAM74A3	-0.197229464	6.50E-06	2.00E-05
FAM74A4	-0.114019131	0.009607	0.01712
FAM75A2	0.069445024	0.115481	0.161708
FAM75A3	0.066126696	0.133965	0.184528
FAM75A5	0.091035104	0.038905	0.060891
FAM75A6	0.012532591	0.776617	0.820213
FAM75C1	0.010959371	0.804049	0.843196
FAM76A	-0.29151574	1.51E-11	1.10E-10
FAM76B	-0.006131585	0.889599	0.913549
FAM78A	-0.260962498	1.83E-09	9.73E-09
FAM78B	-0.055129962	0.211662	0.275311
FAM7A2	-0.063855428	0.147878	0.201266
FAM7A3	-0.150309845	0.000621	0.00137
FAM81A	0.080523053	0.067868	0.100693
FAM81B	-0.258369063	2.67E-09	1.38E-08
FAM82A1	-0.346377985	5.80E-16	8.32E-15
FAM82A2	-0.281555749	7.71E-11	5.05E-10
FAM82B	0.142591533	0.001176	0.00247
FAM83A	0.342187081	1.36E-15	1.85E-14
FAM83B	0.132300681	0.002627	0.005184
FAM83C	0.035821925	0.417243	0.491969
FAM83D	0.513632189	5.19E-36	1.73E-33
FAM83E	-0.204419242	2.91E-06	9.46E-06
FAM83F	0.191070108	1.27E-05	3.74E-05
FAM83G	0.143956491	0.001053	0.002229
FAM83H	0.141538733	0.00128	0.002672
FAM84A	-0.027708864	0.530393	0.601981
FAM84B	-0.039548705	0.370428	0.445253
FAM86A	0.115843142	0.008504	0.015282
FAM86B1	-0.129670783	0.003198	0.006213
FAM86B2	-0.078372084	0.075575	0.110943
FAM86C	0.164551356	0.000176	0.000431
FAM86D	-0.054201564	0.219468	0.283986
FAM89A	0.096627505	0.028334	0.045743
FAM89B	-0.00827085	0.85147	0.882601
FAM8A1	-0.142434469	0.001191	0.002499
FAM90A1	-0.032046131	0.468046	0.541803
FAM90A7	0.014281133	0.746453	0.79506
FAM91A1	0.370281394	3.51E-18	7.02E-17
FAM92A1	0.150903884	0.000591	0.001307
FAM92A3	0.099499137	0.023939	0.039218
FAM92B	-0.177788533	4.97E-05	0.000133

FAM95B1	-0.362320898	2.02E-17	3.58E-16
FAM96A	0.233165445	8.68E-08	3.61E-07
FAM96B	0.101424947	0.021333	0.035361
FAM98A	0.328023391	2.20E-14	2.46E-13
FAM98B	0.181136857	3.55E-05	9.72E-05
FAM98C	-0.142416978	0.001193	0.002502
FAM99A	-0.026644103	0.546317	0.616832
FAM99B	0.045577126	0.301916	0.373619
FAM9A	0.113929991	0.009664	0.017216
FAM9B	0.227635807	1.77E-07	7.00E-07
FAM9C	0.033285111	0.45101	0.525368
FANCA	0.231000444	1.15E-07	4.69E-07
FANCB	0.399072191	4.14E-21	1.29E-19
FANCC	0.09692001	0.027857	0.045034
FANCD2	0.314652509	2.68E-13	2.55E-12
FANCE	0.039935487	0.365763	0.44044
FANCF	0.233007835	8.86E-08	3.68E-07
FANCG	0.35600584	7.81E-17	1.27E-15
FANCI	0.434978353	3.47E-25	1.99E-23
FANCL	0.040666262	0.357049	0.43154
FANCM	0.396113904	8.54E-21	2.50E-19
FANK1	-0.259844948	2.16E-09	1.13E-08
FAP	0.148688273	0.000712	0.001556
FAR1	0.054188269	0.219582	0.284114
FAR2	-0.087601121	0.046925	0.072186
FARP1	-0.154333087	0.00044	0.000997
FARP2	-0.319052052	1.19E-13	1.20E-12
FARS2	0.031570765	0.474676	0.548365
FARSA	0.198578779	5.60E-06	1.74E-05
FARSB	0.398104872	5.25E-21	1.60E-19
FASLG	0.017378505	0.693985	0.749966
FASN	0.026822975	0.543625	0.614348
FASTKD1	0.225811918	2.23E-07	8.68E-07
FASTKD2	0.237908643	4.64E-08	2.01E-07
FASTKD3	0.067955295	0.123515	0.171541
FASTKD5	-0.006470815	0.883534	0.908674
FASTK	-0.032216739	0.465679	0.539491
FAS	-0.166645981	0.000145	0.00036
FAT1	0.119199817	0.006766	0.012403
FAT2	-0.18835623	1.69E-05	4.88E-05
FAT3	-0.219814928	4.70E-07	1.75E-06
FAT4	-0.373966951	1.54E-18	3.25E-17
FATE1	0.227388024	1.83E-07	7.21E-07
FAU	0.087445159	0.04732	0.072712

FBF1	-0.225886226	2.21E-07	8.61E-07
FBLIM1	-0.026234211	0.552509	0.622382
FBLL1	-0.071166064	0.106715	0.150615
FBLN1	-0.241886454	2.72E-08	1.22E-07
FBLN2	-0.133623033	0.002376	0.004729
FBLN5	-0.283467043	5.67E-11	3.78E-10
FBLN7	-0.046935412	0.287722	0.358675
FBL	0.238068122	4.54E-08	1.97E-07
FBN1	0.033722934	0.445074	0.519392
FBN2	0.093310668	0.034257	0.054258
FBN3	-0.227131136	1.89E-07	7.44E-07
FBP1	-0.282220123	6.93E-11	4.57E-10
FBP2	-0.161839153	0.000226	0.000541
FBRSL1	0.022997036	0.602585	0.667728
FBRS	-0.196312168	7.19E-06	2.20E-05
FBXL12	-0.220337558	4.41E-07	1.64E-06
FBXL13	0.080040905	0.069537	0.102925
FBXL14	0.185325611	2.31E-05	6.53E-05
FBXL15	-0.152475972	0.000516	0.001155
FBXL16	-0.214865319	8.57E-07	3.04E-06
FBXL17	-0.112935647	0.010321	0.018272
FBXL18	0.130623708	0.002979	0.005822
FBXL19	0.022944458	0.603415	0.668491
FBXL20	0.009070849	0.837295	0.870707
FBXL21	0.038012968	0.389312	0.464155
FBXL22	-0.084526215	0.055241	0.083693
FBXL2	-0.017513057	0.691737	0.74786
FBXL3	-0.127526863	0.003745	0.007185
FBXL4	0.133405502	0.002416	0.004802
FBXL5	-0.021927516	0.619566	0.682797
FBXL6	-0.006649812	0.880336	0.906224
FBXL7	-0.258129041	2.77E-09	1.43E-08
FBXL8	-0.290273542	1.86E-11	1.34E-10
FBXO10	-0.01784838	0.686145	0.7428
FBXO11	-0.056855188	0.197691	0.259452
FBXO15	-0.250106995	8.73E-09	4.21E-08
FBXO16	-0.006553921	0.882049	0.90752
FBXO17	0.040804524	0.355415	0.429896
FBXO18	0.01223517	0.781783	0.824562
FBXO21	0.093845181	0.033237	0.05278
FBXO22OS	0.344433455	8.63E-16	1.20E-14
FBXO22	0.254907704	4.41E-09	2.22E-08
FBXO24	-0.291762338	1.45E-11	1.06E-10
FBXO25	-0.119633612	0.006566	0.012064

FBXO27	-0.060380517	0.171259	0.229048
FBXO28	0.203834533	3.11E-06	1.01E-05
FBXO2	-0.184531913	2.51E-05	7.05E-05
FBXO30	0.285649426	3.98E-11	2.72E-10
FBXO31	-0.227412582	1.82E-07	7.19E-07
FBXO32	0.229611033	1.37E-07	5.54E-07
FBXO33	0.15296784	0.000495	0.00111
FBXO34	0.046243149	0.294899	0.366241
FBXO36	-0.012069717	0.784661	0.827031
FBXO38	-0.291965377	1.40E-11	1.03E-10
FBXO39	-0.030419271	0.490946	0.564226
FBXO3	-0.152233317	0.000527	0.001177
FBXO40	-0.041752896	0.344335	0.418546
FBXO41	-0.072183091	0.101787	0.144446
FBXO42	-0.24788784	1.19E-08	5.63E-08
FBXO43	0.204667379	2.83E-06	9.21E-06
FBXO44	-0.383083018	1.90E-19	4.62E-18
FBXO45	0.406070543	7.26E-22	2.52E-20
FBXO46	-0.019087836	0.665626	0.724174
FBXO47	0.083588816	0.058007	0.087394
FBXO48	-0.129039748	0.003351	0.006491
FBXO4	-0.068066911	0.122899	0.170851
FBXO5	0.409374217	3.14E-22	1.14E-20
FBXO6	-0.020453976	0.643296	0.70396
FBXO7	-0.14661901	0.000846	0.001823
FBXO8	0.001448458	0.973841	0.980414
FBXO9	-0.12598604	0.004189	0.007969
FBXW10	-0.04537431	0.304075	0.375849
FBXW11	-0.185262892	2.33E-05	6.57E-05
FBXW12	0.040068725	0.364164	0.438806
FBXW2	-0.112517848	0.010608	0.018731
FBXW4	-0.420022478	1.99E-23	8.67E-22
FBXW5	0.018918166	0.668421	0.726503
FBXW7	-0.146588534	0.000848	0.001826
FBXW8	-0.026190294	0.553175	0.622991
FBXW9	-0.048971263	0.267301	0.336873
FCAMR	-0.301818027	2.62E-12	2.13E-11
FCAR	0.026997238	0.541009	0.611738
FCER1A	-0.401069	2.53E-21	8.12E-20
FCER1G	-0.049907579	0.258251	0.326993
FCER2	-0.269754706	4.89E-10	2.84E-09
FCF1	0.249643941	9.32E-09	4.48E-08
FCGBP	-0.427280046	2.86E-24	1.44E-22
FCGR1A	-0.026290931	0.55165	0.621625

FCGR1B	-0.030528594	0.489389	0.562663
FCGR1C	-0.050737868	0.250404	0.31845
FCGR2A	-0.099590452	0.023809	0.03903
FCGR2B	-0.068335749	0.121423	0.169047
FCGR2C	-0.131863499	0.002715	0.005347
FCGR3A	0.029334692	0.50654	0.57925
FCGR3B	0.078107603	0.07657	0.112247
FCGRT	-0.387674837	6.48E-20	1.66E-18
FCHO1	-0.029248411	0.507791	0.580316
FCHO2	-0.144137391	0.001037	0.0022
FCHSD1	-0.379449899	4.41E-19	1.01E-17
FCHSD2	-0.318269199	1.38E-13	1.37E-12
FCN1	-0.26732242	7.08E-10	4.03E-09
FCN2	-0.09897811	0.024689	0.040351
FCN3	-0.181643228	3.37E-05	9.27E-05
FCRL1	-0.289499829	2.12E-11	1.51E-10
FCRL2	-0.165511208	0.000161	0.000397
FCRL3	-0.247109817	1.33E-08	6.22E-08
FCRL4	-0.11907467	0.006824	0.012504
FCRL5	-0.004416335	0.920361	0.937623
FCRL6	-0.259179946	2.38E-09	1.24E-08
FCRLA	-0.110029599	0.012472	0.021674
FCRLB	0.014280605	0.746462	0.79506
FDFT1	-0.013248896	0.764217	0.810268
FDPSL2A	-0.169332808	0.000113	0.000285
FDPS	0.207846218	1.96E-06	6.57E-06
FDX1L	-0.041145977	0.3514	0.425942
FDX1	0.040046594	0.364429	0.439046
FDXACB1	-0.047817009	0.278754	0.349303
FDXR	-0.229486917	1.40E-07	5.62E-07
FECH	0.214452023	9.01E-07	3.19E-06
FEM1A	-0.112463033	0.010646	0.018787
FEM1B	0.163603345	0.000192	0.000467
FEM1C	-0.226251644	2.11E-07	8.25E-07
FEN1	0.413377705	1.13E-22	4.40E-21
FER1L4	0.022977822	0.602888	0.66799
FER1L5	-0.255609423	3.99E-09	2.01E-08
FER1L6	0.036206876	0.412252	0.486946
FERD3L	0.029637552	0.502159	0.574998
FERMT1	0.136694741	0.001876	0.003798
FERMT2	0.205422472	2.59E-06	8.50E-06
FERMT3	-0.215564555	7.88E-07	2.81E-06
FER	0.031215082	0.47967	0.553559
FES	-0.197213692	6.51E-06	2.01E-05

FETUB	0.126204672	0.004123	0.007856
FEV	0.060154452	0.17287	0.231002
FEZ1	-0.201837038	3.90E-06	1.24E-05
FEZ2	-0.057327026	0.19399	0.255097
FEZF1	-0.123604223	0.00497	0.009341
FEZF2	-0.065743835	0.136237	0.187284
FFAR1	-0.105505484	0.016613	0.02814
FFAR2	0.080612479	0.067562	0.100284
FFAR3	0.008583932	0.845917	0.878119
FGA	0.288352414	2.56E-11	1.79E-10
FGB	0.331748788	1.07E-14	1.26E-13
FGD1	0.166404287	0.000148	0.000367
FGD2	-0.306901304	1.08E-12	9.39E-12
FGD3	-0.361936974	2.19E-17	3.87E-16
FGD4	-0.190382184	1.36E-05	4.00E-05
FGD5	-0.308987524	7.43E-13	6.64E-12
FGD6	-0.004971548	0.910388	0.929928
FGF10	-0.196188894	7.29E-06	2.23E-05
FGF11	0.087983514	0.045968	0.07088
FGF12	0.190584853	1.33E-05	3.92E-05
FGF13	-0.117447112	0.007629	0.013849
FGF14	-0.346038695	6.22E-16	8.87E-15
FGF16	-0.008684223	0.84414	0.876639
FGF17	-0.280523854	9.10E-11	5.90E-10
FGF18	-0.236711436	5.44E-08	2.32E-07
FGF19	0.050700518	0.250754	0.318834
FGF1	-0.082847635	0.060275	0.090531
FGF20	-0.21503358	8.40E-07	2.99E-06
FGF21	0.184002849	2.65E-05	7.42E-05
FGF22	-0.16640282	0.000148	0.000368
FGF23	0.167740416	0.000131	0.000327
FGF2	-0.142410996	0.001193	0.002503
FGF3	0.102585164	0.019885	0.033154
FGF4	0.06111142	0.166125	0.222987
FGF5	0.303473965	1.97E-12	1.64E-11
FGF7	-0.093518804	0.033857	0.053692
FGF8	0.003317216	0.940138	0.952818
FGF9	-0.244051507	2.02E-08	9.25E-08
FGFBP1	0.040261896	0.361854	0.436414
FGFBP2	-0.218736301	5.37E-07	1.97E-06
FGFBP3	-0.098202271	0.025845	0.042077
FGFR1OP2	0.18532785	2.31E-05	6.53E-05
FGFR1OP	0.131177297	0.002859	0.005601
FGFR1	-0.112911381	0.010337	0.018293

FGFR2	-0.431722699	8.52E-25	4.59E-23
FGFR3	-0.356531461	6.99E-17	1.15E-15
FGFR4	-0.124989187	0.004501	0.008522
FGFRL1	0.028716844	0.515538	0.587858
FGGY	-0.201312924	4.13E-06	1.31E-05
FGG	0.23605134	5.94E-08	2.53E-07
FGL1	0.25668273	3.42E-09	1.74E-08
FGL2	-0.213054548	1.06E-06	3.72E-06
FGR	-0.267676116	6.71E-10	3.83E-09
FHAD1	-0.23643657	5.64E-08	2.41E-07
FHDC1	-0.360726621	2.85E-17	4.97E-16
FHIT	-0.213011617	1.07E-06	3.74E-06
FHL1	-0.340614604	1.87E-15	2.48E-14
FHL2	0.197621818	6.23E-06	1.92E-05
FHL3	0.028427649	0.519777	0.592064
FHL5	-0.27295771	2.99E-10	1.79E-09
FHOD1	-0.294890272	8.59E-12	6.50E-11
FHOD3	0.058756877	0.183085	0.242624
FH	0.279119638	1.14E-10	7.25E-10
FIBCD1	0.044606308	0.312341	0.384379
FIBIN	-0.09796983	0.0262	0.04261
FIBP	0.090894879	0.039209	0.061318
FICD	-0.019001023	0.667055	0.725532
FIG4	-0.27087862	4.12E-10	2.42E-09
FIGF	-0.407826498	4.66E-22	1.64E-20
FIGLA	-0.083974547	0.056856	0.085859
FIGNL1	0.208936912	1.73E-06	5.84E-06
FIGNL2	-0.167691827	0.000132	0.000329
FIGN	0.212261463	1.17E-06	4.05E-06
FILIP1L	-0.193720744	9.53E-06	2.86E-05
FILIP1	-0.176401536	5.70E-05	0.00015
FIP1L1	0.223208575	3.09E-07	1.18E-06
FIS1	0.062055407	0.159666	0.215345
FITM1	-0.305529298	1.37E-12	1.18E-11
FITM2	-0.049722764	0.26002	0.328859
FIZ1	-0.166901069	0.000142	0.000352
FJX1	0.124317002	0.004723	0.008917
FKBP10	0.276967085	1.60E-10	9.96E-10
FKBP11	0.115962234	0.008436	0.015176
FKBP14	0.231245519	1.11E-07	4.56E-07
FKBP15	-0.263547495	1.25E-09	6.81E-09
FKBP1AP1	-0.0136517	0.75727	0.804182
FKBP1A	0.131167164	0.002861	0.005604
FKBP1B	0.075474943	0.087067	0.125743

FKBP2	-0.016484214	0.708998	0.763301
FKBP3	0.460520871	2.13E-28	1.81E-26
FKBP4	0.391406823	2.66E-20	7.27E-19
FKBP5	0.05881286	0.182667	0.242087
FKBP6	-0.257221437	3.16E-09	1.62E-08
FKBP7	0.015486588	0.725878	0.777579
FKBP8	-0.181098072	3.56E-05	9.76E-05
FKBP9L	0.121289128	0.005852	0.01085
FKBP9	0.131377113	0.002816	0.005526
FKBPL	0.20607814	2.41E-06	7.93E-06
FKRP	-0.168313462	0.000124	0.000311
FKSG29	-0.073219563	0.096953	0.138352
FKSG83	-0.155514401	0.000397	0.000905
FKTN	0.103061985	0.019315	0.032285
FLAD1	0.309649152	6.60E-13	5.95E-12
FLCN	-0.278362187	1.28E-10	8.13E-10
FLG2	-0.084755748	0.054581	0.082818
FLG	-0.051423704	0.244049	0.311458
FLI1	-0.296148834	6.94E-12	5.33E-11
FLII	-0.126718017	0.003973	0.007591
FLJ10038	-0.388076681	5.89E-20	1.52E-18
FLJ10213	-0.226098	2.15E-07	8.40E-07
FLJ10357	-0.317928281	1.47E-13	1.45E-12
FLJ10661	-0.279846418	1.01E-10	6.53E-10
FLJ11235	-0.389577025	4.12E-20	1.10E-18
FLJ12825	-0.080314146	0.068587	0.101647
FLJ13197	-0.412465761	1.42E-22	5.38E-21
FLJ13224	-0.089502697	0.042328	0.065741
FLJ14107	-0.222552499	3.35E-07	1.27E-06
FLJ16779	0.036396479	0.409807	0.484487
FLJ22536	0.246294333	1.49E-08	6.92E-08
FLJ23867	-0.123783479	0.004907	0.009232
FLJ25363	0.155599779	0.000394	0.000899
FLJ25758	0.178178545	4.78E-05	0.000128
FLJ26850	-0.220313414	4.42E-07	1.65E-06
FLJ30679	0.093310462	0.034258	0.054258
FLJ32063	-0.035637125	0.419651	0.494309
FLJ33360	-0.411634315	1.76E-22	6.60E-21
FLJ33630	-0.165239049	0.000165	0.000406
FLJ34503	-0.250689974	8.04E-09	3.90E-08
FLJ35024	-0.094397168	0.03221	0.051329
FLJ35220	-0.139508833	0.001505	0.003101
FLJ35390	-0.151229891	0.000574	0.001274
FLJ35776	0.03144365	0.476457	0.550169

FLJ36000	0.062192003	0.158748	0.21425
FLJ36031	0.19118162	1.25E-05	3.69E-05
FLJ36777	-0.338724871	2.72E-15	3.54E-14
FLJ37201	-0.089862305	0.041503	0.064628
FLJ37307	-0.216989938	6.64E-07	2.40E-06
FLJ37453	-0.231276881	1.11E-07	4.54E-07
FLJ37543	-0.153598388	0.000469	0.001056
FLJ39582	-0.022016561	0.618145	0.681493
FLJ39609	-0.143148227	0.001124	0.00237
FLJ39653	-0.067238901	0.127531	0.176432
FLJ39739	0.149579613	0.000661	0.001452
FLJ40292	-0.112855573	0.010375	0.018354
FLJ40330	-0.091896906	0.037086	0.058308
FLJ40504	0.029596526	0.502751	0.575515
FLJ40852	-0.186048665	2.15E-05	6.10E-05
FLJ41941	-0.032972238	0.455278	0.529291
FLJ42289	-0.251876843	6.80E-09	3.33E-08
FLJ42393	-0.061738207	0.161815	0.217817
FLJ42627	-0.185673633	2.23E-05	6.32E-05
FLJ42709	-0.158603697	0.000302	0.000705
FLJ42875	-0.463683085	8.15E-29	7.47E-27
FLJ43390	-0.235856226	6.09E-08	2.59E-07
FLJ43663	-0.232601303	9.34E-08	3.86E-07
FLJ43859	0.126579068	0.004013	0.007661
FLJ43860	-0.13268456	0.002552	0.005048
FLJ43950	0.024105696	0.585207	0.651831
FLJ44054	0.086076021	0.050908	0.077755
FLJ44606	-0.27345802	2.77E-10	1.67E-09
FLJ44635	0.05805459	0.188383	0.248837
FLJ45079	-0.166660192	0.000145	0.000359
FLJ45244	-0.276018582	1.86E-10	1.15E-09
FLJ45340	-0.339395371	2.38E-15	3.13E-14
FLJ45445	-0.182652684	3.04E-05	8.43E-05
FLJ45983	-0.040831388	0.355098	0.429643
FLJ46111	0.134248922	0.002265	0.004523
FLJ46321	-0.029136322	0.50942	0.581878
FLJ46361	-0.026779756	0.544275	0.614978
FLJ90757	-0.373571302	1.68E-18	3.51E-17
FLNA	-0.219771461	4.73E-07	1.76E-06
FLNB	-0.070641891	0.109327	0.153976
FLNC	0.228884704	1.51E-07	6.03E-07
FLOT1	0.07316288	0.097213	0.138703
FLOT2	-0.116712644	0.008019	0.014504
FLRT1	-0.062337578	0.157773	0.213108

FLRT2	0.091781929	0.037324	0.058632
FLRT3	-0.128506803	0.003486	0.006725
FLT1	0.076363673	0.083401	0.120965
FLT3LG	-0.311588954	4.66E-13	4.28E-12
FLT3	-0.291932102	1.41E-11	1.04E-10
FLT4	-0.140471592	0.001394	0.002888
FLVCR1	0.237093656	5.17E-08	2.22E-07
FLVCR2	-0.045866671	0.298852	0.370263
FLYWCH1	-0.331303745	1.17E-14	1.36E-13
FLYWCH2	-0.14013747	0.001431	0.00296
FMN1	-0.21379453	9.75E-07	3.43E-06
FMN2	-0.148610718	0.000717	0.001565
FMNL1	-0.098702933	0.025094	0.040952
FMNL2	-0.197544281	6.28E-06	1.94E-05
FMNL3	-0.259116706	2.40E-09	1.25E-08
FMO1	0.042398328	0.336922	0.410871
FMO2	-0.302605786	2.29E-12	1.88E-11
FMO3	-0.264575309	1.07E-09	5.92E-09
FMO4	-0.442869093	3.77E-26	2.48E-24
FMO5	-0.237298151	5.03E-08	2.16E-07
FMO6P	-0.133314982	0.002433	0.00483
FMO9P	0.064666194	0.142791	0.195258
FMOD	-0.082699451	0.060737	0.091177
FMR1NB	0.007780914	0.860176	0.889963
FMR1	-0.116941229	0.007896	0.014297
FN1	0.055379176	0.209601	0.273003
FN3KRP	-0.030016187	0.496711	0.569869
FN3K	-0.121893135	0.005609	0.010444
FNBP1L	-0.033103077	0.453491	0.527673
FNBP1	-0.219465296	4.91E-07	1.82E-06
FNBP4	-0.289388286	2.16E-11	1.53E-10
FNDC1	0.096223388	0.029005	0.046698
FNDC3A	0.050833553	0.249511	0.317435
FNDC3B	0.203254149	3.32E-06	1.07E-05
FNDC4	0.133065357	0.002479	0.004913
FNDC5	-0.308259425	8.46E-13	7.49E-12
FNDC7	-0.065988668	0.134781	0.185511
FNDC8	-0.009541147	0.828986	0.863909
FNIP1	-0.132499022	0.002588	0.005112
FNIP2	-0.324424178	4.37E-14	4.68E-13
FNTA	0.230978227	1.15E-07	4.70E-07
FNTB	0.077888696	0.077402	0.113265
FOLH1B	0.104354911	0.017841	0.030027
FOLH1	0.113249221	0.010109	0.017929

FOLR1	-0.332638699	9.03E-15	1.07E-13
FOLR2	-0.19183085	1.17E-05	3.46E-05
FOLR3	-0.082449349	0.061524	0.09224
FOLR4	-0.140145201	0.001431	0.002959
FOSB	-0.263059339	1.34E-09	7.29E-09
FOSL1	0.294143775	9.75E-12	7.31E-11
FOSL2	0.026102007	0.554514	0.624113
FOS	-0.257194624	3.17E-09	1.62E-08
FOXA1	0.128525658	0.003481	0.006717
FOXA2	-0.297825847	5.22E-12	4.06E-11
FOXA3	0.096612668	0.028358	0.045778
FOXB1	0.006060335	0.890874	0.91467
FOXB2	0.037363544	0.39747	0.472248
FOXC1	-0.137331845	0.001785	0.003629
FOXC2	-0.063689591	0.148936	0.202526
FOXD1	0.070012343	0.112531	0.158021
FOXD2	-0.048390371	0.273024	0.343155
FOXD3	0.065244391	0.139245	0.190933
FOXD4L1	-0.197811148	6.10E-06	1.89E-05
FOXD4L2	-0.209457652	1.63E-06	5.51E-06
FOXD4L3	-0.154009655	0.000452	0.001022
FOXD4L5	-0.134074463	0.002296	0.00458
FOXD4L6	-0.237272133	5.05E-08	2.17E-07
FOXD4	-0.177366499	5.18E-05	0.000138
FOXE1	0.006324461	0.88615	0.910602
FOXE3	0.033715707	0.445172	0.519475
FOXF1	-0.334116357	6.76E-15	8.23E-14
FOXF2	-0.308508427	8.10E-13	7.20E-12
FOXG1	0.149968033	0.000639	0.001407
FOXH1	0.096794345	0.028061	0.045342
FOXI1	-0.192781567	1.05E-05	3.15E-05
FOXI2	-0.315358952	2.35E-13	2.26E-12
FOXI3	0.038838067	0.379094	0.45382
FOXJ1	-0.255743677	3.91E-09	1.98E-08
FOXJ2	-0.12637815	0.004072	0.007764
FOXJ3	-0.019045578	0.666322	0.724852
FOXK1	-0.003895118	0.929734	0.945053
FOXK2	0.142277779	0.001206	0.002528
FOXL1	-0.023023957	0.60216	0.667347
FOXL2	0.103525296	0.018775	0.031467
FOXM1	0.508047836	3.80E-35	1.12E-32
FOXN1	-0.183429976	2.81E-05	7.82E-05
FOXN2	0.156487449	0.000364	0.000837
FOXN3	-0.30782974	9.13E-13	8.05E-12

FOXN4	-0.024749196	0.575227	0.642865
FOXO1	-0.16979033	0.000108	0.000274
FOXO3B	-0.231896447	1.02E-07	4.21E-07
FOXO3	-0.27167714	3.64E-10	2.16E-09
FOXO4	-0.275957821	1.87E-10	1.16E-09
FOXP1	-0.117896961	0.007399	0.013467
FOXP2	0.112833745	0.01039	0.018377
FOXP3	-0.131856537	0.002716	0.005349
FOXP4	-0.189513521	1.49E-05	4.36E-05
FOXQ1	-0.22512602	2.43E-07	9.41E-07
FOXR1	0.016357352	0.711136	0.765109
FOXR2	0.131618467	0.002765	0.005434
FOXRED1	0.052329801	0.235828	0.302434
FOXRED2	-9.37E-05	0.998308	0.999007
FOXS1	-0.16818904	0.000126	0.000315
FPGS	-0.026329686	0.551064	0.621179
FPGT	-0.024883206	0.573159	0.641235
FPR1	-0.038113502	0.388058	0.462826
FPR2	0.032128301	0.466905	0.540733
FPR3	-0.114182499	0.009503	0.016942
FRAS1	-0.196873921	6.76E-06	2.08E-05
FRAT1	-0.240488877	3.28E-08	1.45E-07
FRAT2	0.095979417	0.029417	0.047315
FREM1	-0.232173579	9.87E-08	4.07E-07
FREM2	-0.299529563	3.90E-12	3.08E-11
FRG1B	-0.072287025	0.101294	0.143889
FRG1	0.09488885	0.031319	0.050056
FRG2B	0.062842765	0.154426	0.209067
FRG2C	0.038712725	0.380636	0.455223
FRG2	0.022451348	0.611223	0.675465
FRK	-0.043075019	0.32926	0.402672
FRMD1	0.009370085	0.832006	0.866289
FRMD3	-0.049532259	0.261853	0.330799
FRMD4A	-0.285924959	3.81E-11	2.60E-10
FRMD4B	-0.273710511	2.66E-10	1.61E-09
FRMD5	0.13461265	0.002203	0.00441
FRMD6	0.157609769	0.00033	0.000764
FRMD7	0.035962421	0.415417	0.490163
FRMD8	-0.076838774	0.081493	0.118533
FRMPD1	-0.144223992	0.00103	0.002186
FRMPD2L1	-0.081925904	0.063197	0.094465
FRMPD2	-0.094627006	0.031791	0.050733
FRMPD4	-0.194270485	8.98E-06	2.71E-05
FRRS1	0.059237069	0.179526	0.238694

FRS2	-0.034360482	0.436511	0.51092
FRS3	-0.270421185	4.42E-10	2.59E-09
FRYL	-0.1471881	0.000807	0.001745
FRY	-0.418060752	3.33E-23	1.41E-21
FRZB	-0.308493838	8.12E-13	7.22E-12
FSCB	0.05102608	0.24772	0.315554
FSCN1	0.181169507	3.54E-05	9.69E-05
FSCN2	-0.204058781	3.03E-06	9.82E-06
FSCN3	-0.140321964	0.001411	0.002921
FSD1L	0.11805867	0.007317	0.013336
FSD1	0.115986497	0.008422	0.015154
FSD2	-0.194913103	8.38E-06	2.54E-05
FSHB	0.004377255	0.921063	0.938148
FSHR	-0.128114537	0.003587	0.006902
FSIP1	-0.036610237	0.40706	0.48181
FSTL1	0.000438822	0.992074	0.994163
FSTL3	0.025158936	0.568915	0.637272
FSTL4	0.045552669	0.302176	0.373848
FSTL5	0.085432964	0.05267	0.080175
FST	0.207767153	1.98E-06	6.62E-06
FTCD	0.000845652	0.984726	0.988461
FTH1	0.042759281	0.332821	0.406553
FTHL17	0.07414271	0.092803	0.132895
FTHL3	0.09845524	0.025463	0.041506
FTLP10	-0.033338874	0.450278	0.524577
FTL	-0.002687796	0.951481	0.962345
FTMT	0.039340925	0.372949	0.447772
FTO	-0.177732661	4.99E-05	0.000133
FTSJ1	0.263725949	1.21E-09	6.66E-09
FTSJ2	0.045204655	0.305888	0.377623
FTSJ3	0.092503465	0.035849	0.056568
FTSJD1	0.01415376	0.748638	0.797048
FTSJD2	-0.142140327	0.00122	0.002554
FUBP1	0.23935857	3.82E-08	1.67E-07
FUBP3	0.222725273	3.28E-07	1.25E-06
FUCA1	-0.402191707	1.92E-21	6.29E-20
FUCA2	0.336342131	4.37E-15	5.48E-14
FUK	-0.259040751	2.43E-09	1.26E-08
FUNDC1	0.022049316	0.617622	0.681067
FUNDC2P2	0.184640549	2.48E-05	6.98E-05
FUNDC2	0.163984949	0.000186	0.000452
FURIN	0.234943444	6.87E-08	2.90E-07
FUS	-0.000241215	0.995643	0.99709
FUT10	0.021400322	0.628013	0.690582

FUT11	0.072299681	0.101234	0.143835
FUT1	-0.135770793	0.002015	0.004064
FUT2	0.034319178	0.437063	0.511446
FUT3	-0.145669983	0.000915	0.001958
FUT4	0.096232139	0.02899	0.046685
FUT5	0.031601174	0.47425	0.547937
FUT6	-0.03601653	0.414715	0.489421
FUT7	-0.259394751	2.30E-09	1.21E-08
FUT8	0.080828829	0.066826	0.099332
FUT9	-0.042452521	0.336304	0.410281
FUZ	-0.126441472	0.004053	0.007731
FXC1	-0.120781357	0.006063	0.011203
FXN	0.122434443	0.005399	0.010086
FXR1	0.201911467	3.86E-06	1.23E-05
FXR2	0.027741734	0.529905	0.601674
FXYD1	-0.426324511	3.70E-24	1.83E-22
FXYD2	-0.040174149	0.362902	0.43747
FXYD3	-0.085865758	0.051479	0.07854
FXYD4	0.129051899	0.003348	0.006486
FXYD5	-0.162499519	0.000213	0.000513
FXYD6	-0.262936068	1.37E-09	7.41E-09
FXYD7	-0.301855206	2.61E-12	2.12E-11
FYB	-0.103059225	0.019318	0.032288
FYCO1	-0.371643778	2.59E-18	5.26E-17
FYN	-0.266051987	8.57E-10	4.81E-09
FYTTD1	0.223981608	2.81E-07	1.08E-06
FZD10	0.178534704	4.61E-05	0.000124
FZD1	-0.205200134	2.66E-06	8.70E-06
FZD2	-0.134760608	0.002178	0.004366
FZD3	0.097918942	0.026278	0.04272
FZD4	-0.095797919	0.029726	0.04779
FZD5	-0.091427494	0.038068	0.059706
FZD6	0.108100142	0.014111	0.02429
FZD7	-0.122048945	0.005548	0.010338
FZD8	-0.218650703	5.42E-07	1.99E-06
FZD9	0.017550871	0.691105	0.747391
FZR1	-0.059314801	0.178955	0.238067
G0S2	0.057567969	0.19212	0.253038
G2E3	0.415905779	5.85E-23	2.38E-21
G3BP1	0.329380577	1.70E-14	1.94E-13
G3BP2	0.186030585	2.15E-05	6.11E-05
G6PC2	-0.092855031	0.035148	0.055554
G6PC3	0.02522371	0.56792	0.636443
G6PC	0.159056495	0.00029	0.00068

G6PD	0.215427338	8.01E-07	2.85E-06
GAA	-0.135815082	0.002009	0.004051
GAB1	-0.331535495	1.12E-14	1.31E-13
GAB2	-0.162315752	0.000216	0.00052
GAB3	-0.390987941	2.94E-20	8.00E-19
GAB4	0.01798956	0.683796	0.740637
GABARAPL1	0.058530278	0.184782	0.244566
GABARAPL2	-0.029662473	0.5018	0.574682
GABARAPL3	0.053439113	0.226032	0.291328
GABARAP	-0.043790857	0.321279	0.394164
GABBR1	-0.339712294	2.24E-15	2.94E-14
GABBR2	-0.192711152	1.06E-05	3.17E-05
GABPA	-0.008006098	0.856172	0.886647
GABPB1	0.418225479	3.19E-23	1.36E-21
GABPB2	-0.05694768	0.196962	0.258597
GABRA1	0.016640733	0.706362	0.76116
GABRA2	0.104587698	0.017586	0.029631
GABRA3	0.163040805	0.000202	0.00049
GABRA4	-0.133129146	0.002467	0.004892
GABRA5	0.130574767	0.00299	0.005841
GABRA6	0.009756828	0.825182	0.860752
GABRB1	-0.043020171	0.329877	0.4033
GABRB2	-0.123747304	0.004919	0.009252
GABRB3	-0.015035631	0.733553	0.784162
GABRD	-0.078577162	0.074811	0.109902
GABRE	-0.309462103	6.83E-13	6.13E-12
GABRG1	0.006660925	0.880138	0.906113
GABRG2	0.153927263	0.000456	0.001029
GABRG3	0.091201715	0.038548	0.060393
GABRP	-0.167080584	0.000139	0.000346
GABRQ	0.174194743	7.07E-05	0.000184
GABRR1	0.077012585	0.080804	0.117702
GABRR2	-0.207912835	1.95E-06	6.52E-06
GABRR3	0.030697135	0.486993	0.560424
GAD1	-0.007602142	0.863356	0.892607
GAD2	-0.042852201	0.33177	0.405369
GADD45A	-0.002184849	0.960551	0.96982
GADD45B	-0.149348081	0.000674	0.001478
GADD45GIP1	0.175685506	6.11E-05	0.000161
GADD45G	-0.172397112	8.41E-05	0.000216
GADL1	-0.154065348	0.00045	0.001018
GAGE10	-0.033993657	0.441426	0.515737
GAGE12D	0.078418888	0.0754	0.110702
GAGE12F	0.048334018	0.273583	0.343794

GAGE12J	0.084505198	0.055302	0.083772
GAGE13	0.037519378	0.395503	0.470386
GAGE1	0.091698323	0.037499	0.058888
GAGE2A	0.068778883	0.119021	0.165992
GAGE2B	0.064541508	0.143564	0.196184
GAGE2C	0.034453206	0.435273	0.50983
GAGE2D	0.093040134	0.034784	0.055018
GAGE2E	0.041745093	0.344426	0.41863
GAGE4	0.086903988	0.048712	0.074695
GAGE8	0.073395682	0.09615	0.137314
GAK	-0.072842864	0.098689	0.140618
GAL3ST1	-0.106301704	0.015807	0.026898
GAL3ST2	0.035339672	0.423545	0.498165
GAL3ST3	0.040893193	0.35437	0.428995
GAL3ST4	-0.080422048	0.068215	0.101163
GALC	-0.15365645	0.000466	0.001051
GALE	0.027739843	0.529933	0.601674
GALK1	0.062485335	0.156788	0.211893
GALK2	0.168867055	0.000118	0.000296
GALM	-0.036958528	0.402609	0.477276
GALNS	-0.017977397	0.683998	0.740816
GALNT10	-0.201528554	4.03E-06	1.28E-05
GALNT11	-0.254465748	4.70E-09	2.35E-08
GALNT12	-0.175801328	6.04E-05	0.000159
GALNT13	0.029840688	0.499232	0.572237
GALNT14	0.143254357	0.001115	0.002352
GALNT1	0.133871621	0.002332	0.004645
GALNT2	0.215940751	7.53E-07	2.69E-06
GALNT3	-0.005879347	0.894114	0.917148
GALNT4	0.284356564	4.91E-11	3.30E-10
GALNT5	-0.146859262	0.000829	0.001789
GALNT6	0.106819614	0.015301	0.026122
GALNT7	0.151933064	0.000541	0.001205
GALNT8	-0.025854266	0.55828	0.62775
GALNT9	0.004628644	0.916546	0.93469
GALNTL1	-0.362282168	2.04E-17	3.61E-16
GALNTL2	-0.039559795	0.370293	0.445139
GALNTL4	-0.321174375	8.04E-14	8.24E-13
GALNTL5	-0.02641619	0.549756	0.620049
GALNTL6	0.008802543	0.842044	0.874917
GALP	0.099335681	0.024172	0.039576
GALR1	0.024151412	0.584495	0.651184
GALR2	-0.032558773	0.460954	0.534863
GALR3	0.120382839	0.006234	0.011497

GALT	-0.21441477	9.05E-07	3.20E-06
GAL	0.414777967	7.84E-23	3.11E-21
GAMT	-0.074411507	0.091622	0.131477
GANAB	0.192414977	1.10E-05	3.26E-05
GANC	-0.384836249	1.26E-19	3.15E-18
GAN	0.224827588	2.52E-07	9.75E-07
GAP43	0.131682448	0.002752	0.005413
GAPDHS	-0.15715367	0.000344	0.000793
GAPDH	0.463787079	7.90E-29	7.27E-27
GAPT	-0.341246063	1.64E-15	2.21E-14
GAPVD1	-0.144941898	0.000971	0.00207
GAR1	0.308372716	8.29E-13	7.36E-12
GARNL3	-0.329780444	1.57E-14	1.81E-13
GARS	0.43502657	3.42E-25	1.97E-23
GART	0.360500583	2.99E-17	5.21E-16
GAS1	-0.02401221	0.586663	0.653139
GAS2L1	0.059282525	0.179192	0.238317
GAS2L2	-0.397394081	6.25E-21	1.87E-19
GAS2L3	0.274545769	2.34E-10	1.43E-09
GAS2	0.001540411	0.972181	0.979137
GAS5	0.120395275	0.006228	0.011488
GAS6	-0.339064366	2.54E-15	3.32E-14
GAS7	-0.299662891	3.81E-12	3.02E-11
GAS8	-0.222855222	3.23E-07	1.23E-06
GAST	0.222753178	3.27E-07	1.24E-06
GATA1	-0.377950727	6.22E-19	1.39E-17
GATA2	-0.204413219	2.91E-06	9.46E-06
GATA3	-0.111086106	0.011648	0.020376
GATA4	-0.040332549	0.361012	0.435743
GATA5	-0.380920855	3.14E-19	7.38E-18
GATA6	-0.328140533	2.15E-14	2.41E-13
GATAD1	-0.04620116	0.295338	0.366657
GATAD2A	-0.019612452	0.657015	0.716404
GATAD2B	-0.164289039	0.000181	0.000441
GATC	0.313801428	3.13E-13	2.95E-12
GATM	-0.110837155	0.011838	0.020665
GATSL1	0.204441277	2.90E-06	9.44E-06
GATSL2	0.11991944	0.006437	0.011844
GATSL3	-0.204517728	2.88E-06	9.36E-06
GATS	-0.037400793	0.396999	0.471885
GBA2	-0.164539948	0.000176	0.000432
GBA3	-0.09542187	0.030376	0.048706
GBAP1	0.048199696	0.27492	0.345243
GBAS	0.162492819	0.000213	0.000513

GBA	0.07783605	0.077603	0.113536
GBE1	0.179535628	4.17E-05	0.000113
GBF1	-0.074335105	0.091957	0.131892
GBGT1	-0.123122328	0.005143	0.009644
GBP1	0.106869537	0.015253	0.02605
GBP2	-0.02994477	0.497736	0.570718
GBP3	-0.049999562	0.257373	0.326109
GBP4	-0.05504946	0.212331	0.276037
GBP5	0.031359536	0.477638	0.551437
GBP6	-0.087976142	0.045987	0.070903
GBP7	0.02784224	0.528415	0.60052
GBX1	0.004238598	0.923556	0.940255
GBX2	0.272575889	3.17E-10	1.89E-09
GCAT	-0.072441569	0.100564	0.142995
GCA	-0.084197569	0.056198	0.084975
GCC1	0.02502703	0.570944	0.639078
GCC2	-0.234112205	7.67E-08	3.21E-07
GCDH	-0.176919016	5.41E-05	0.000143
GCET2	-0.363948625	1.42E-17	2.58E-16
GCFC1	-0.194398614	8.86E-06	2.67E-05
GCGR	-0.012075861	0.784554	0.827005
GCG	0.066536274	0.131567	0.181462
GCH1	0.337439719	3.51E-15	4.48E-14
GCHFR	-0.098092804	0.026011	0.042325
GCKR	0.09257759	0.0357	0.056355
GCK	-0.104831409	0.017323	0.029212
GCLC	0.289093488	2.26E-11	1.60E-10
GCLM	0.330570659	1.35E-14	1.56E-13
GCM1	-0.07224647	0.101486	0.144101
GCM2	-0.019909756	0.652155	0.711882
GCN1L1	0.194010991	9.24E-06	2.78E-05
GCNT1	-0.074274192	0.092224	0.132208
GCNT2	0.076981131	0.080928	0.117856
GCNT3	0.044347205	0.315163	0.387494
GCNT4	-0.307351568	9.94E-13	8.72E-12
GCNT7	-0.236780366	5.39E-08	2.30E-07
GCOM1	-0.180400738	3.82E-05	0.000104
GCSH	0.300759383	3.15E-12	2.54E-11
GC	-0.069702037	0.114137	0.16004
GDAP1L1	-0.17830721	4.72E-05	0.000126
GDAP1	0.016638455	0.7064	0.76116
GDAP2	-0.011214911	0.799576	0.839563
GDA	0.060219218	0.172407	0.230445
GDE1	-0.187076853	1.93E-05	5.53E-05

GDEP	-0.089177232	0.043087	0.066808
GDF10	-0.30253472	2.32E-12	1.90E-11
GDF11	-0.05814747	0.187676	0.248034
GDF15	-0.327813676	2.29E-14	2.56E-13
GDF1	0.104357483	0.017838	0.030025
GDF2	-0.147780489	0.000768	0.001668
GDF3	-0.106990699	0.015137	0.025882
GDF5	-0.239604451	3.70E-08	1.62E-07
GDF6	0.030561063	0.488926	0.562171
GDF7	-0.257528962	3.02E-09	1.55E-08
GDF9	-0.169238362	0.000114	0.000287
GDI1	-0.039382122	0.372448	0.447251
GDI2	0.34419358	9.06E-16	1.26E-14
GDNF	0.018276241	0.679035	0.736318
GDPD1	-0.054858081	0.213927	0.277913
GDPD2	-0.066277861	0.133076	0.183389
GDPD3	-0.015012828	0.733942	0.784536
GDPD4	-0.021708584	0.623068	0.685993
GDPD5	-0.260368376	2.00E-09	1.05E-08
GEFT	-0.117759011	0.007469	0.013577
GEMIN4	0.011219094	0.799502	0.839546
GEMIN5	0.056157311	0.20326	0.265852
GEMIN6	0.352241921	1.73E-16	2.66E-15
GEMIN7	0.306494189	1.16E-12	1.00E-11
GEMIN8P4	0.11223055	0.01081	0.019059
GEMIN8	-0.177340973	5.19E-05	0.000138
GEM	-0.006741428	0.8787	0.905193
GEN1	0.212416415	1.15E-06	3.98E-06
GET4	-0.005424375	0.902265	0.923519
GFAP	0.07913169	0.072776	0.107142
GFER	0.03664302	0.40664	0.481427
GFI1B	-0.3181882	1.40E-13	1.39E-12
GFI1	-0.057270711	0.19443	0.255608
GFM1	0.282147022	7.02E-11	4.62E-10
GFM2	0.171869308	8.85E-05	0.000227
GFOD1	-0.153723426	0.000464	0.001046
GFOD2	-0.014469454	0.743227	0.792257
GFPT1	0.162447083	0.000214	0.000515
GFPT2	0.1780379	4.84E-05	0.00013
GFRA1	-0.472871294	4.73E-30	5.31E-28
GFRA2	-0.303294606	2.03E-12	1.69E-11
GFRA3	-0.145776148	0.000907	0.001942
GFRA4	0.050904453	0.24885	0.316756
GFRAL	0.016327901	0.711633	0.765438

GGA1	-0.232655711	9.27E-08	3.84E-07
GGA2	-0.536533662	9.92E-40	7.94E-37
GGA3	-0.296298728	6.77E-12	5.20E-11
GGCT	0.105510085	0.016608	0.028137
GGCX	-0.099725111	0.023619	0.038748
GGH	0.414387934	8.67E-23	3.43E-21
GGNBP1	-0.041484673	0.347446	0.421815
GGNBP2	-0.109191683	0.013162	0.022786
GGN	-0.071792764	0.103657	0.146734
GGPS1	0.120043768	0.006382	0.011748
GGT1	-0.241334648	2.93E-08	1.30E-07
GGT3P	-0.399356069	3.86E-21	1.21E-19
GGT5	-0.279607051	1.05E-10	6.76E-10
GGT6	-0.367716734	6.21E-18	1.20E-16
GGT7	-0.303821839	1.85E-12	1.55E-11
GGT8P	-0.276735989	1.66E-10	1.03E-09
GGTA1	-0.262276197	1.51E-09	8.14E-09
GGTLC1	-0.444300964	2.51E-26	1.71E-24
GGTLC2	-0.256843586	3.34E-09	1.70E-08
GH1	-0.068557808	0.120215	0.167516
GH2	0.127676633	0.003704	0.007111
GHDC	-0.310620713	5.55E-13	5.06E-12
GHITM	0.313127039	3.53E-13	3.31E-12
GHRHR	0.004643816	0.916273	0.934507
GHRH	0.01442785	0.743939	0.792932
GHRLOS	-0.201706324	3.95E-06	1.26E-05
GHRL	-0.357632095	5.53E-17	9.21E-16
GHR	-0.340512703	1.90E-15	2.53E-14
GHSR	0.003365982	0.939259	0.952121
GIF	-0.022396739	0.612091	0.676124
GIGYF1	-0.288738565	2.40E-11	1.69E-10
GIGYF2	-0.146274053	0.000871	0.00187
GIMAP1	-0.294049355	9.90E-12	7.41E-11
GIMAP2	-0.161611788	0.000231	0.000552
GIMAP4	-0.149546639	0.000662	0.001456
GIMAP5	-0.145351018	0.000939	0.002006
GIMAP6	-0.184555326	2.51E-05	7.03E-05
GIMAP7	-0.212977492	1.07E-06	3.75E-06
GIMAP8	-0.26595025	8.71E-10	4.88E-09
GIN1	-0.094345318	0.032305	0.051464
GINS1	0.45954514	2.86E-28	2.41E-26
GINS2	0.431433411	9.22E-25	4.94E-23
GINS3	0.419859805	2.07E-23	9.03E-22
GINS4	0.391549734	2.57E-20	7.06E-19

GIPC1	-0.061711734	0.161995	0.218015
GIPC2	-0.134261513	0.002263	0.004519
GIPC3	-0.046435461	0.292894	0.364145
GIPR	-0.395629997	9.61E-21	2.80E-19
GIP	0.25070901	8.02E-09	3.89E-08
GIT1	0.067141409	0.128085	0.177113
GIT2	-0.172960064	7.97E-05	0.000206
GIYD2	-0.059519684	0.177456	0.236388
GJA10	0.074280468	0.092197	0.132178
GJA1	0.090272609	0.040578	0.063296
GJA3	0.050366147	0.253896	0.322419
GJA4	-0.129773834	0.003174	0.006171
GJA5	-0.062579554	0.156163	0.21118
GJA8	0.021560393	0.625443	0.688203
GJA9	-0.054657042	0.215613	0.279831
GJB1	-0.204328391	2.94E-06	9.55E-06
GJB2	0.240561091	3.25E-08	1.44E-07
GJB3	0.184406724	2.54E-05	7.14E-05
GJB4	0.027539111	0.532916	0.604295
GJB5	0.080391559	0.06832	0.101296
GJB6	0.027546295	0.532809	0.604209
GJB7	0.041202079	0.350743	0.425301
GJC1	0.122525566	0.005365	0.010026
GJC2	-0.233167726	8.67E-08	3.61E-07
GJC3	0.010931842	0.804531	0.843614
GJD2	-0.010493392	0.812222	0.850028
GJD3	-0.046567476	0.291522	0.362756
GJD4	0.007630244	0.862856	0.892182
GK2	-0.019661247	0.656216	0.71569
GK3P	0.074180695	0.092636	0.132731
GK5	-0.11142299	0.011396	0.019996
GKAP1	-0.095529738	0.030189	0.048459
GKN1	-0.045907489	0.298422	0.369801
GKN2	-0.171735603	8.97E-05	0.00023
GK	0.044932711	0.30881	0.380713
GLA	0.289321259	2.18E-11	1.55E-10
GLB1L2	-0.285606474	4.01E-11	2.74E-10
GLB1L3	-0.356827403	6.56E-17	1.08E-15
GLB1L	-0.252846939	5.92E-09	2.93E-08
GLB1	0.062087683	0.159449	0.21511
GLCCI1	-0.278262844	1.30E-10	8.25E-10
GLCE	0.18468204	2.47E-05	6.95E-05
GLDC	0.309844829	6.38E-13	5.76E-12
GLDN	-0.235624853	6.28E-08	2.66E-07

GLE1	0.001456384	0.973698	0.980319
GLG1	-0.028267952	0.522126	0.59405
GLI1	-0.244036488	2.03E-08	9.27E-08
GLI2	-0.086902578	0.048716	0.074695
GLI3	-0.148577239	0.000719	0.00157
GLI4	-0.06916154	0.116977	0.163518
GLIPR1L1	0.004527	0.918372	0.936027
GLIPR1L2	-0.253037081	5.77E-09	2.85E-08
GLIPR1	-0.041775751	0.344071	0.418276
GLIPR2	-0.158825384	0.000296	0.000693
GLIS1	-0.022773316	0.60612	0.670938
GLIS2	-0.239889739	3.56E-08	1.57E-07
GLIS3	-0.095749822	0.029809	0.047899
GLMN	0.225660891	2.27E-07	8.84E-07
GLO1	0.113370748	0.010028	0.017804
GLOD4	0.16398692	0.000186	0.000452
GLOD5	-0.266619716	7.87E-10	4.44E-09
GLP1R	-0.288081222	2.67E-11	1.87E-10
GLP2R	-0.027737219	0.529972	0.601674
GLRA1	0.040047195	0.364422	0.439046
GLRA2	-0.092097578	0.036673	0.057708
GLRA3	-0.063273385	0.151615	0.205736
GLRA4	-0.029102532	0.509911	0.582322
GLRB	-0.029040408	0.510815	0.583134
GLRX2	0.328599583	1.97E-14	2.23E-13
GLRX3	0.426212902	3.81E-24	1.88E-22
GLRX5	0.357688324	5.47E-17	9.11E-16
GLRX	0.090926066	0.039141	0.061222
GLS2	-0.316491602	1.91E-13	1.86E-12
GLS	-0.070294432	0.111086	0.156135
GLT1D1	-0.045154866	0.306422	0.378165
GLT25D1	0.142938859	0.001143	0.002407
GLT25D2	-0.293474977	1.09E-11	8.11E-11
GLT8D1	0.001097198	0.980183	0.98531
GLT8D2	0.008770624	0.842609	0.875433
GLTPD1	-0.03694176	0.402823	0.477501
GLTPD2	0.046278233	0.294533	0.365887
GLTP	-0.032993842	0.454983	0.52904
GLTSCR1	-0.127649525	0.003712	0.007124
GLTSCR2	-0.237988219	4.59E-08	1.99E-07
GLUD1	0.21452027	8.94E-07	3.16E-06
GLUD2	0.187309286	1.88E-05	5.40E-05
GLUL	-0.166017082	0.000154	0.00038
GLYATL1	0.094972638	0.031169	0.049829

GLYATL2	-0.15190505	0.000542	0.001207
GLYATL3	0.094032853	0.032885	0.052287
GLYAT	-0.004586152	0.917309	0.935196
GLYCTK	-0.006334247	0.885975	0.910528
GLYR1	-0.237710349	4.77E-08	2.06E-07
GM2A	-0.137552845	0.001755	0.003571
GMCL1L	0.079985477	0.069731	0.103174
GMCL1	0.150223607	0.000626	0.001379
GMDS	0.20893759	1.73E-06	5.84E-06
GMEB1	0.124135169	0.004785	0.009024
GMEB2	0.000201801	0.996355	0.997535
GMFB	0.494124447	4.65E-33	8.86E-31
GMFG	-0.188810304	1.61E-05	4.67E-05
GMIP	-0.295047485	8.37E-12	6.35E-11
GML	-0.077657436	0.078288	0.114413
GMNN	0.394629572	1.23E-20	3.49E-19
GMPPA	0.152064313	0.000535	0.001193
GMPPB	0.075603423	0.086529	0.124985
GMPR2	0.11071849	0.011929	0.020817
GMPR	-0.33226974	9.70E-15	1.14E-13
GMPS	0.380503424	3.46E-19	8.07E-18
GNA11	-0.102586751	0.019883	0.033154
GNA12	0.081688912	0.063967	0.095473
GNA13	0.142562985	0.001179	0.002475
GNA14	-0.258686872	2.55E-09	1.33E-08
GNA15	-0.082290969	0.062026	0.092909
GNAI1	-0.036238513	0.411843	0.486492
GNAI2	-0.226789404	1.97E-07	7.75E-07
GNAI3	0.297028595	5.98E-12	4.62E-11
GNAL	-0.073088437	0.097555	0.139161
GNAO1	-0.341418613	1.59E-15	2.14E-14
GNAQ	-0.252624824	6.11E-09	3.01E-08
GNASAS	-0.012942621	0.769512	0.814672
GNAS	-0.022279273	0.613958	0.67785
GNAT1	0.126013483	0.004181	0.007955
GNAT2	0.045883511	0.298675	0.370066
GNAT3	-0.027402687	0.534947	0.606153
GNAZ	-0.188001627	1.75E-05	5.06E-05
GNB1L	0.103452403	0.018859	0.031584
GNB1	0.174095361	7.14E-05	0.000186
GNB2L1	0.027550009	0.532753	0.60418
GNB2	0.062810844	0.154636	0.209294
GNB3	-0.152035459	0.000536	0.001195
GNB4	0.0846211	0.054967	0.083322

GNB5	-0.02840055	0.520176	0.592283
GNE	0.030333554	0.492169	0.565437
GNG10	0.129877779	0.00315	0.006126
GNG11	-0.015468306	0.726189	0.777803
GNG12	-0.03943189	0.371844	0.446767
GNG13	-0.025954649	0.556752	0.626174
GNG2	-0.207386016	2.07E-06	6.89E-06
GNG3	-0.060065607	0.173507	0.231744
GNG4	0.27397606	2.55E-10	1.55E-09
GNG5	0.128013586	0.003614	0.006948
GNG7	-0.45576775	8.84E-28	7.16E-26
GNG8	-0.053310221	0.227155	0.292606
GNGT1	0.177745698	4.99E-05	0.000133
GNGT2	-0.142537164	0.001181	0.00248
GNL1	-0.076038545	0.084727	0.122693
GNL2	0.095688968	0.029913	0.048059
GNL3L	0.105632589	0.016482	0.027939
GNL3	0.380018828	3.87E-19	8.97E-18
GNLY	0.082165179	0.062428	0.093441
GNMT	-0.333883853	7.08E-15	8.57E-14
GNPAT	0.1657959	0.000157	0.000387
GNPDA1	0.070464209	0.110224	0.155043
GNPDA2	0.042070351	0.340676	0.414805
GNPNAT1	1	1.00E-54	1.00E-50
GNPTAB	-0.203423427	3.26E-06	1.05E-05
GNPTG	-0.239903901	3.55E-08	1.56E-07
GNRH1	-0.317212676	1.68E-13	1.64E-12
GNRH2	0.053329845	0.226983	0.292442
GNRHR2	-0.299142	4.17E-12	3.28E-11
GNRHR	-0.179851808	4.04E-05	0.00011
GNS	0.00999062	0.821063	0.857306
GOLGA1	-0.152646372	0.000509	0.001139
GOLGA2B	-0.404177885	1.17E-21	3.93E-20
GOLGA2P3	-0.057722785	0.190926	0.251663
GOLGA2	-0.091477788	0.037962	0.059563
GOLGA3	-0.012469233	0.777717	0.821097
GOLGA4	-0.092662193	0.035531	0.056102
GOLGA5	0.364794624	1.18E-17	2.17E-16
GOLGA6A	-0.083922067	0.057011	0.086061
GOLGA6B	-0.119702934	0.006535	0.012008
GOLGA6C	0.059789656	0.175495	0.234086
GOLGA6D	0.057156678	0.195321	0.256661
GOLGA6L10	-0.328460193	2.02E-14	2.28E-13
GOLGA6L1	0.077055768	0.080633	0.11748

GOLGA6L5	-0.252711542	6.04E-09	2.98E-08
GOLGA6L6	0.046661928	0.290543	0.361638
GOLGA6L9	-0.358572659	4.53E-17	7.62E-16
GOLGA7B	-0.051180305	0.246292	0.313959
GOLGA7	0.206831136	2.21E-06	7.31E-06
GOLGA8A	-0.340411663	1.94E-15	2.58E-14
GOLGA8B	-0.30626226	1.21E-12	1.04E-11
GOLGA8C	0.117913447	0.00739	0.013455
GOLGA8DP	0.026565613	0.5475	0.617854
GOLGA8E	0.04126243	0.350038	0.424651
GOLGA8F	-0.000359473	0.993507	0.9952
GOLGA8G	0.062066336	0.159593	0.21526
GOLGA9P	-0.192031298	1.14E-05	3.39E-05
GOLGB1	-0.233180151	8.66E-08	3.60E-07
GOLIM4	-0.03259597	0.460442	0.534362
GOLM1	0.232768953	9.14E-08	3.79E-07
GOLPH3L	-0.07444807	0.091462	0.131323
GOLPH3	0.085548048	0.052351	0.079731
GOLT1A	-0.115365812	0.008781	0.01574
GOLT1B	0.423937729	7.02E-24	3.29E-22
GON4L	-0.142376903	0.001196	0.002509
GOPC	0.04355088	0.32394	0.397064
GORAB	-0.092504351	0.035847	0.056568
GORASP1	-0.227464408	1.81E-07	7.15E-07
GORASP2	0.225856857	2.22E-07	8.63E-07
GOSR1	0.093290246	0.034297	0.054307
GOSR2	0.135513564	0.002056	0.00414
GOT1L1	0.102991144	0.019399	0.032409
GOT1	0.246932558	1.36E-08	6.37E-08
GOT2	0.29692438	6.09E-12	4.69E-11
GP1BA	-0.350855315	2.30E-16	3.47E-15
GP2	0.062498665	0.1567	0.211815
GP5	-0.207065543	2.15E-06	7.13E-06
GP6	-0.182559433	3.07E-05	8.50E-05
GP9	-0.151238238	0.000574	0.001274
GPA33	-0.147367138	0.000795	0.001721
GPAA1	0.027621584	0.531689	0.603178
GPAM	0.01239191	0.77906	0.822254
GPAT2	-0.09331949	0.03424	0.054239
GPATCH1	-0.049508191	0.262085	0.331072
GPATCH2	0.165569062	0.00016	0.000395
GPATCH3	-0.118534219	0.007083	0.012931
GPATCH4	0.198135669	5.88E-06	1.83E-05
GPATCH8	-0.138291454	0.001656	0.003386

GPBAR1	-0.211294543	1.31E-06	4.51E-06
GPBP1L1	-0.213137116	1.05E-06	3.68E-06
GPBP1	-0.088994132	0.043519	0.067404
GPC1	-0.067070171	0.128491	0.177625
GPC2	0.074984047	0.089146	0.128367
GPC3	-0.299024521	4.25E-12	3.35E-11
GPC4	-0.430189701	1.30E-24	6.71E-23
GPC5	-0.194783275	8.50E-06	2.57E-05
GPC6	0.350842119	2.31E-16	3.47E-15
GPCPD1	-0.154399295	0.000437	0.000992
GPD1L	-0.376174226	9.33E-19	2.04E-17
GPD1	-0.285151207	4.32E-11	2.93E-10
GPD2	0.207641904	2.01E-06	6.70E-06
GPER	-0.225169181	2.42E-07	9.36E-07
GPHA2	-0.086761551	0.049084	0.075214
GPHN	0.182387218	3.13E-05	8.63E-05
GPIHBP1	-0.328839596	1.88E-14	2.14E-13
GPI	0.313785725	3.13E-13	2.96E-12
GPKOW	0.034819701	0.430402	0.505101
GPLD1	-0.297816375	5.23E-12	4.07E-11
GPM6A	-0.232954173	8.92E-08	3.70E-07
GPM6B	-0.103895958	0.018352	0.03082
GPN1	0.388165746	5.76E-20	1.49E-18
GPN2	-0.009633135	0.827363	0.862533
GPN3	0.358387814	4.71E-17	7.90E-16
GPNMB	-0.199948123	4.81E-06	1.51E-05
GPR101	-0.064215848	0.1456	0.198665
GPR107	0.012527869	0.776699	0.820239
GPR108	-0.284326903	4.94E-11	3.32E-10
GPR109A	0.025441907	0.564575	0.633475
GPR109B	0.052475025	0.234529	0.300999
GPR110	-0.254803806	4.48E-09	2.24E-08
GPR111	0.093037368	0.034789	0.055022
GPR112	-0.236895823	5.31E-08	2.27E-07
GPR113	-0.212878223	1.09E-06	3.79E-06
GPR114	-0.149411673	0.00067	0.001471
GPR115	0.273833754	2.61E-10	1.58E-09
GPR116	-0.481223781	3.30E-31	4.55E-29
GPR119	0.108344371	0.013894	0.023936
GPR120	-0.258157432	2.76E-09	1.42E-08
GPR123	-0.218035344	5.85E-07	2.14E-06
GPR124	-0.146377859	0.000863	0.001856
GPR125	-0.006852991	0.876709	0.9037
GPR126	0.047603343	0.28091	0.351564

GPR128	0.076512601	0.082799	0.120214
GPR12	-0.261017846	1.81E-09	9.66E-09
GPR132	-0.265341239	9.54E-10	5.33E-09
GPR133	-0.288862122	2.35E-11	1.66E-10
GPR135	-0.032415285	0.462933	0.53691
GPR137B	-0.035773963	0.417867	0.492559
GPR137C	0.156126784	0.000376	0.000861
GPR137	-0.176957929	5.39E-05	0.000143
GPR139	-0.005852123	0.894601	0.917591
GPR141	-0.045391228	0.303894	0.375672
GPR142	-0.007930183	0.857522	0.887768
GPR143	-0.209448354	1.63E-06	5.52E-06
GPR144	0.039404865	0.372172	0.447027
GPR146	-0.420308405	1.84E-23	8.08E-22
GPR148	-0.036778519	0.404906	0.479686
GPR149	-0.09243906	0.035978	0.05675
GPR150	-0.084862767	0.054275	0.082423
GPR151	-0.034348272	0.436674	0.511051
GPR152	0.037682198	0.393454	0.46834
GPR153	-0.067723331	0.124805	0.173044
GPR155	-0.223440217	3.00E-07	1.15E-06
GPR156	-0.076993378	0.08088	0.117795
GPR157	0.011016137	0.803054	0.842331
GPR158	0.071901215	0.103135	0.146151
GPR15	0.054511947	0.216836	0.281181
GPR160	-0.226215739	2.12E-07	8.28E-07
GPR161	-0.047740703	0.279522	0.350046
GPR162	-0.314830757	2.59E-13	2.48E-12
GPR171	-0.153396639	0.000477	0.001073
GPR172A	0.258816046	2.51E-09	1.30E-08
GPR172B	-0.337043572	3.80E-15	4.82E-14
GPR173	-0.149474985	0.000666	0.001464
GPR174	-0.154529213	0.000432	0.000981
GPR176	0.139031633	0.001562	0.003207
GPR179	-0.220301589	4.43E-07	1.65E-06
GPR17	-0.334671588	6.07E-15	7.45E-14
GPR180	0.305548574	1.37E-12	1.17E-11
GPR182	-0.133452536	0.002407	0.004786
GPR183	-0.168369289	0.000124	0.00031
GPR18	-0.160161643	0.000263	0.000621
GPR19	0.299843239	3.69E-12	2.94E-11
GPR1	0.167241073	0.000137	0.000341
GPR20	-0.161991528	0.000223	0.000534
GPR21	-0.018239515	0.679644	0.736898

GPR22	0.062928599	0.153862	0.208403
GPR25	-0.14901765	0.000693	0.001517
GPR26	0.001281312	0.976859	0.982957
GPR27	-0.139638765	0.001489	0.003071
GPR31	-0.131902203	0.002707	0.005335
GPR32	0.083685692	0.057716	0.087021
GPR34	-0.144967053	0.000969	0.002066
GPR35	0.178311989	4.71E-05	0.000126
GPR37L1	0.218550409	5.49E-07	2.02E-06
GPR37	0.114990892	0.009004	0.016108
GPR39	0.002509434	0.954697	0.965175
GPR3	0.041085006	0.352115	0.426731
GPR44	-0.35242511	1.66E-16	2.56E-15
GPR45	-0.123458406	0.005022	0.009432
GPR4	0.068664352	0.119638	0.166783
GPR50	0.023702821	0.591495	0.65748
GPR52	-0.021280579	0.629938	0.691906
GPR55	-0.29091395	1.67E-11	1.21E-10
GPR56	-0.052085841	0.238022	0.30472
GPR61	-0.074961965	0.089241	0.128475
GPR62	-0.12083421	0.006041	0.011168
GPR63	0.115647424	0.008617	0.015463
GPR64	-0.229896631	1.33E-07	5.36E-07
GPR65	-0.153569855	0.00047	0.001058
GPR68	-0.141299739	0.001305	0.002718
GPR6	-0.038848228	0.37897	0.453719
GPR75	-0.193992829	9.25E-06	2.78E-05
GPR77	-0.026847093	0.543263	0.614008
GPR78	0.181034529	3.59E-05	9.81E-05
GPR81	0.055113342	0.2118	0.275455
GPR82	-0.160912366	0.000246	0.000584
GPR83	0.011180967	0.800169	0.84001
GPR84	0.005636373	0.898466	0.92062
GPR85	-0.220037823	4.58E-07	1.70E-06
GPR87	0.070801381	0.108527	0.152946
GPR88	-0.074439707	0.091499	0.131357
GPR89A	0.151255827	0.000573	0.001272
GPR89B	0.049199756	0.265072	0.334339
GPR89C	0.046712895	0.290016	0.361174
GPR97	0.264867848	1.02E-09	5.68E-09
GPR98	-0.213983965	9.53E-07	3.36E-06
GPRASP1	-0.483345619	1.66E-31	2.38E-29
GPRASP2	-0.254824455	4.47E-09	2.24E-08
GPRC5A	-0.063016265	0.153289	0.207696

GPRC5B	-0.080002647	0.069671	0.103093
GPRC5C	-0.271885644	3.53E-10	2.09E-09
GPRC5D	-0.076585273	0.082507	0.11986
GPRC6A	-0.030314766	0.492437	0.56568
GPRIN1	0.144900889	0.000975	0.002076
GPRIN2	-0.304409784	1.67E-12	1.41E-11
GPRIN3	-0.015877698	0.719244	0.771755
GPS1	0.107080577	0.015052	0.025765
GPS2	-0.046150367	0.29587	0.367183
GPSM1	-0.178833167	4.48E-05	0.00012
GPSM2	0.161053993	0.000243	0.000577
GPSM3	-0.237089187	5.18E-08	2.22E-07
GPT2	0.207711595	1.99E-06	6.66E-06
GPT	-0.039402982	0.372195	0.447027
GPX1	-0.016713385	0.70514	0.76013
GPX2	0.160535258	0.000254	0.000602
GPX3	-0.1606725	0.000251	0.000596
GPX4	-0.079311419	0.072126	0.106364
GPX5	-0.035683832	0.419042	0.493711
GPX6	-0.025122401	0.569477	0.637798
GPX7	0.000611622	0.988953	0.991831
GPX8	0.214407816	9.06E-07	3.20E-06
GRAMD1A	-0.004827948	0.912966	0.93199
GRAMD1B	0.128129632	0.003583	0.006896
GRAMD1C	-0.074324922	0.092001	0.131936
GRAMD2	-0.299386907	3.99E-12	3.16E-11
GRAMD3	-0.203356845	3.28E-06	1.06E-05
GRAMD4	-0.12871201	0.003433	0.006632
GRAP2	-0.222026469	3.58E-07	1.35E-06
GRAPL	-0.161425736	0.000235	0.00056
GRAP	-0.143544997	0.001089	0.002301
GRASP	-0.353454381	1.34E-16	2.09E-15
GRB10	0.102743181	0.019694	0.032873
GRB14	0.046693611	0.290216	0.361332
GRB2	-0.059322464	0.178899	0.238008
GRB7	-0.162659301	0.00021	0.000506
GREB1L	0.235206254	6.64E-08	2.81E-07
GREB1	-0.162353605	0.000216	0.000519
GREM1	0.273740557	2.65E-10	1.60E-09
GREM2	-0.148425757	0.000728	0.001588
GRHL1	-0.186093111	2.14E-05	6.08E-05
GRHL2	-0.089220441	0.042985	0.066676
GRHL3	-0.115995892	0.008417	0.015148
GRHPR	0.174427474	6.91E-05	0.00018

GRIA1	-0.362912449	1.78E-17	3.18E-16
GRIA2	-0.03794645	0.390142	0.464881
GRIA3	-0.128533742	0.003479	0.006714
GRIA4	-0.109666211	0.012767	0.022149
GRID1	-0.321579444	7.45E-14	7.71E-13
GRID2IP	-0.297918455	5.14E-12	4.00E-11
GRID2	0.05942134	0.178174	0.237202
GRIK1	0.048685064	0.27011	0.339942
GRIK2	-0.027304354	0.536414	0.607333
GRIK3	-0.217566688	6.19E-07	2.25E-06
GRIK4	-0.275780604	1.93E-10	1.19E-09
GRIK5	-0.012726095	0.773262	0.817732
GRIN1	-0.001787076	0.967729	0.97539
GRIN2A	-0.19492818	8.36E-06	2.53E-05
GRIN2B	0.079266539	0.072288	0.106555
GRIN2C	-0.193579033	9.68E-06	2.90E-05
GRIN2D	0.217217197	6.46E-07	2.34E-06
GRIN3A	-0.05410896	0.220258	0.284953
GRIN3B	-0.095491074	0.030256	0.048547
GRINA	0.068222022	0.122046	0.169831
GRINL1A	0.184942621	2.41E-05	6.78E-05
GRIP1	-0.110123043	0.012397	0.021559
GRIP2	-0.072415815	0.100686	0.143137
GRIPAP1	-0.205432942	2.59E-06	8.49E-06
GRK1	-0.068997515	0.11785	0.164531
GRK4	0.120376772	0.006236	0.011501
GRK5	-0.151363683	0.000568	0.001261
GRK6	-0.105879549	0.01623	0.027545
GRK7	0.010190468	0.817546	0.854392
GRLF1	-0.20587369	2.46E-06	8.11E-06
GRM1	-0.04796541	0.277263	0.34774
GRM2	-0.156494511	0.000364	0.000836
GRM3	-0.251906763	6.77E-09	3.31E-08
GRM4	-0.037592936	0.394576	0.46948
GRM5	0.036002199	0.414901	0.489612
GRM6	-0.296995953	6.01E-12	4.64E-11
GRM7	-0.184403834	2.54E-05	7.14E-05
GRM8	0.163594352	0.000192	0.000467
GRN	-0.30905795	7.34E-13	6.57E-12
GRPEL1	0.318200943	1.40E-13	1.39E-12
GRPEL2	0.197263775	6.48E-06	2.00E-05
GRPR	-0.041079155	0.352184	0.426788
GRP	-0.124927065	0.004521	0.008555
GRRP1	-0.308746268	7.76E-13	6.91E-12

GRSF1	0.395177428	1.07E-20	3.09E-19
GRTP1	-0.023737074	0.590959	0.656994
GRWD1	0.190291876	1.38E-05	4.04E-05
GRXCR1	-0.035657622	0.419384	0.494056
GRXCR2	-0.011055106	0.802372	0.841659
GSC2	-0.012232784	0.781825	0.824563
GSC	0.055198822	0.211091	0.274711
GSDMA	-0.066048147	0.134429	0.185065
GSDMB	-0.257714922	2.94E-09	1.51E-08
GSDMC	0.071460181	0.105271	0.148777
GSDMD	-0.140212228	0.001423	0.002944
GSG1L	-0.28868743	2.42E-11	1.70E-10
GSG1	0.055269416	0.210507	0.274005
GSG2	0.412679055	1.35E-22	5.14E-21
GSK3A	0.184017475	2.65E-05	7.41E-05
GSK3B	0.046786873	0.289252	0.360312
GSN	-0.392826328	1.89E-20	5.25E-19
GSPT1	0.020137014	0.648449	0.708611
GSPT2	-0.22544042	2.34E-07	9.07E-07
GSR	0.231029645	1.15E-07	4.67E-07
GSS	0.182651632	3.04E-05	8.43E-05
GSTA1	-0.082109621	0.062606	0.093665
GSTA2	-0.064827488	0.141795	0.19403
GSTA3	-0.22286768	3.23E-07	1.23E-06
GSTA4	-0.13778999	0.001723	0.003512
GSTA5	-0.129755402	0.003178	0.006179
GSTCD	0.35543833	8.81E-17	1.41E-15
GSTK1	-0.117346143	0.007682	0.013935
GSTM1	-0.054490864	0.217014	0.281338
GSTM2P1	-0.005367879	0.903278	0.92434
GSTM2	-0.293604671	1.07E-11	7.95E-11
GSTM3	-0.033640418	0.446189	0.520572
GSTM4	-0.186040054	2.15E-05	6.11E-05
GSTM5	-0.355212183	9.24E-17	1.47E-15
GSTO1	0.213745754	9.80E-07	3.45E-06
GSTO2	0.093411954	0.034062	0.053982
GSTP1	0.08970383	0.041865	0.06513
GSTT1	-0.038960679	0.37759	0.452393
GSTT2	-0.09494732	0.031214	0.049897
GSTTP1	0.033264332	0.451292	0.525636
GSTTP2	0.037976628	0.389765	0.464507
GSTZ1	0.063046634	0.15309	0.20747
GSX2	-0.005122251	0.907684	0.92783
GTDC1	0.068636603	0.119788	0.166965

GTF2A1L	0.078525254	0.075004	0.110161
GTF2A1	0.147223211	0.000805	0.00174
GTF2A2	0.289403325	2.15E-11	1.53E-10
GTF2B	0.168388075	0.000123	0.000309
GTF2E1	0.295888899	7.26E-12	5.56E-11
GTF2E2	0.297303604	5.71E-12	4.43E-11
GTF2F1	-0.042653798	0.334016	0.40781
GTF2F2	0.280029187	9.85E-11	6.35E-10
GTF2H1	0.157795874	0.000325	0.000753
GTF2H2B	0.021138725	0.632222	0.693951
GTF2H2C	0.035183351	0.4256	0.500229
GTF2H2	0.113758947	0.009774	0.017394
GTF2H3	0.312664068	3.84E-13	3.58E-12
GTF2H4	0.113296684	0.010078	0.017881
GTF2H5	0.146435923	0.000859	0.001848
GTF2IP1	-0.185618325	2.25E-05	6.35E-05
GTF2IRD1	0.158150828	0.000315	0.000732
GTF2IRD2B	-0.326089899	3.18E-14	3.49E-13
GTF2IRD2P1	-0.374993043	1.22E-18	2.63E-17
GTF2IRD2	-0.353774711	1.25E-16	1.96E-15
GTF2I	-0.006948959	0.874996	0.9024
GTF3A	0.309137913	7.24E-13	6.49E-12
GTF3C1	-0.108607625	0.013663	0.023573
GTF3C2	0.212021773	1.20E-06	4.16E-06
GTF3C3	0.27182986	3.56E-10	2.11E-09
GTF3C4	0.147902724	0.00076	0.001652
GTF3C5	0.083955558	0.056912	0.085937
GTF3C6	0.261592326	1.67E-09	8.93E-09
GTPBP10	0.242493469	2.50E-08	1.13E-07
GTPBP1	-0.257449388	3.06E-09	1.57E-08
GTPBP2	-0.013906495	0.752885	0.800632
GTPBP3	-0.007253947	0.869558	0.898228
GTPBP4	0.417873978	3.50E-23	1.48E-21
GTPBP5	-0.01170041	0.791095	0.832451
GTPBP8	0.20364539	3.18E-06	1.03E-05
GTSE1	0.43686213	2.05E-25	1.23E-23
GTSF1L	-0.119365594	0.006689	0.012275
GTSF1	0.054213676	0.219365	0.283905
GUCA1A	0.336286355	4.41E-15	5.54E-14
GUCA1B	-0.015157048	0.731484	0.782578
GUCA1C	0.072725394	0.099235	0.141315
GUCA2A	-0.077887554	0.077406	0.113265
GUCA2B	0.134431636	0.002234	0.004466
GUCY1A2	-0.183927958	2.67E-05	7.47E-05

GUCY1A3	-0.274240349	2.45E-10	1.49E-09
GUCY1B2	-0.047813497	0.278789	0.349325
GUCY1B3	-0.144880732	0.000976	0.002079
GUCY2C	0.084448164	0.055467	0.083977
GUCY2D	-0.134023057	0.002305	0.004596
GUCY2E	0.095352259	0.030498	0.048877
GUCY2F	-0.173030102	7.92E-05	0.000204
GUCY2GP	0.11587277	0.008487	0.015257
GUF1	0.146226217	0.000874	0.001877
GUK1	-0.002797155	0.949509	0.960611
GULP1	0.181045235	3.58E-05	9.80E-05
GUSBL1	-0.23374944	8.04E-08	3.35E-07
GUSBL2	-0.086133523	0.050753	0.077557
GUSBP1	-0.200533642	4.51E-06	1.42E-05
GUSBP3	-0.16195188	0.000224	0.000536
GUSB	-0.161263383	0.000238	0.000567
GVIN1	-0.216971137	6.65E-07	2.40E-06
GXYLT1	0.067149571	0.128039	0.177061
GXYLT2	-0.064190118	0.145762	0.198845
GYG1	0.196389078	7.13E-06	2.18E-05
GYG2	-0.040753898	0.356013	0.430515
GYLTL1B	-0.075751106	0.085914	0.124204
GYPA	-0.041265772	0.349999	0.42463
GYPB	-0.120973798	0.005982	0.011072
GYPC	-0.250129731	8.70E-09	4.20E-08
GYPE	-0.289832635	2.00E-11	1.43E-10
GYS1	-0.097557972	0.026839	0.043562
GYS2	0.11102353	0.011695	0.020447
GZF1	-0.043551569	0.323933	0.397064
GZMA	0.062655055	0.155663	0.210542
GZMB	0.222715429	3.29E-07	1.25E-06
GZMH	0.025598259	0.562184	0.631572
GZMK	-0.124097654	0.004798	0.009046
GZMM	-0.125945818	0.004201	0.00799
H19	0.044861876	0.309574	0.381561
H1F0	0.176831881	5.46E-05	0.000145
H1FNT	-0.366534442	8.05E-18	1.53E-16
H1FOO	-0.00345668	0.937626	0.950802
H1FX	-0.024523937	0.578711	0.645928
H2AFB1	-0.015089448	0.732636	0.783517
H2AFJ	0.074171388	0.092677	0.132771
H2AFV	0.178142788	4.79E-05	0.000128
H2AFX	0.381048322	3.05E-19	7.19E-18
H2AFY2	-0.078053792	0.076774	0.112496

H2AFY	0.138847438	0.001585	0.00325
H2AFZ	0.494655628	3.89E-33	7.62E-31
H2BFM	-0.022322346	0.613273	0.677169
H2BFWT	0.029596269	0.502755	0.575515
H2BFXP	-0.157142019	0.000344	0.000794
H3F3A	0.100497519	0.022555	0.037173
H3F3B	-0.074963143	0.089236	0.128475
H3F3C	-0.07142154	0.10546	0.149012
H6PD	-0.268783492	5.67E-10	3.27E-09
HAAO	-0.359714662	3.54E-17	6.10E-16
HABP2	-0.315335956	2.36E-13	2.27E-12
HABP4	-0.175849562	6.01E-05	0.000158
HACE1	-0.127334549	0.003798	0.007277
HACL1	0.024825659	0.574047	0.641761
HADHA	0.168631966	0.00012	0.000303
HADHB	0.080867646	0.066695	0.099152
HADH	-0.128865243	0.003395	0.006563
HAGHL	-0.073668621	0.094916	0.135688
HAGH	-0.265151374	9.82E-10	5.47E-09
HAL	0.145155008	0.000955	0.002036
HAMP	0.02380627	0.589877	0.65601
HAND1	-0.148058802	0.000751	0.001634
HAND2	-0.138240194	0.001663	0.003398
HAO1	0.203122012	3.37E-06	1.08E-05
HAO2	-0.07170723	0.10407	0.147267
HAP1	-0.016845902	0.702912	0.758178
HAPLN1	0.099213944	0.024347	0.039827
HAPLN2	-0.064132922	0.146122	0.199223
HAPLN3	0.034557835	0.43388	0.508485
HAPLN4	-0.124922688	0.004523	0.008557
HAR1A	-0.13131211	0.00283	0.005551
HAR1B	0.020755458	0.63841	0.699626
HARBI1	0.158714699	0.000299	0.000699
HARS2	0.014714419	0.739036	0.788758
HARS	0.07630985	0.083619	0.121229
HAS1	-0.039226903	0.374337	0.449222
HAS2AS	0.119903219	0.006445	0.011855
HAS2	-0.023775753	0.590354	0.656431
HAS3	-0.317035475	1.73E-13	1.69E-12
HAT1	0.266443536	8.08E-10	4.56E-09
HAUS1	0.249264707	9.83E-09	4.72E-08
HAUS2	0.236360176	5.70E-08	2.43E-07
HAUS3	0.020129157	0.648577	0.708712
HAUS4	0.031026488	0.482329	0.556114

HAUS5	-0.162973758	0.000204	0.000492
HAUS6	0.33292522	8.54E-15	1.02E-13
HAUS7	0.15973795	0.000273	0.000643
HAUS8	0.127893473	0.003646	0.007006
HAVCR1	0.141647468	0.001269	0.00265
HAVCR2	-0.083716813	0.057623	0.0869
HAX1	0.210265956	1.48E-06	5.04E-06
HBA1	-0.201319792	4.13E-06	1.31E-05
HBA2	-0.201081727	4.24E-06	1.35E-05
HBBP1	0.11100715	0.011708	0.020467
HBB	-0.16020227	0.000262	0.000619
HBD	0.072609985	0.099774	0.141982
HBE1	0.071397186	0.105579	0.14916
HBEGF	-0.143246615	0.001115	0.002353
HBG1	-0.077102684	0.080449	0.117244
HBG2	-0.02176262	0.622203	0.685212
HBM	-0.08725152	0.047814	0.073386
HBP1	-0.079843858	0.070229	0.103811
HBQ1	0.192216427	1.12E-05	3.33E-05
HBS1L	0.249896955	8.99E-09	4.34E-08
HBXIP	0.185348424	2.31E-05	6.52E-05
HBZ	-0.215161815	8.27E-07	2.94E-06
HCCS	0.340081073	2.08E-15	2.74E-14
HCFC1R1	-0.083335219	0.058775	0.088477
HCFC1	-0.036470258	0.408858	0.483565
HCFC2	-0.06732526	0.127042	0.175852
HCG11	-0.080230962	0.068875	0.102036
HCG18	0.075272282	0.08792	0.126802
HCG22	-0.004697298	0.915312	0.933724
HCG26	-0.324530516	4.28E-14	4.60E-13
HCG27	-0.361934328	2.20E-17	3.87E-16
HCG2P7	-0.269248274	5.28E-10	3.06E-09
HCG4P6	-0.132542328	0.002579	0.005098
HCG4	-0.097468489	0.02698	0.043762
HCG9	-0.026319807	0.551213	0.621273
HCK	-0.214609904	8.84E-07	3.13E-06
HCLS1	-0.217134586	6.52E-07	2.36E-06
HCN1	-0.167077531	0.000139	0.000346
HCN2	0.077461832	0.079045	0.115425
HCN3	-0.098236121	0.025793	0.042004
HCN4	-0.189831227	1.44E-05	4.23E-05
HCP5	-0.082567361	0.061152	0.091757
HCRTR1	-0.23240039	9.59E-08	3.96E-07
HCRTR2	-0.155892997	0.000384	0.000878

HCRT	-0.042292473	0.338131	0.412233
HCST	-0.162334065	0.000216	0.00052
HDAC10	-0.357215041	6.04E-17	1.00E-15
HDAC11	-0.316014877	2.09E-13	2.02E-12
HDAC1	0.147911566	0.00076	0.001651
HDAC2	0.344147685	9.15E-16	1.27E-14
HDAC3	-0.081620402	0.064191	0.095764
HDAC4	-0.024861859	0.573488	0.641435
HDAC5	-0.432779029	6.37E-25	3.53E-23
HDAC6	-0.276164789	1.81E-10	1.12E-09
HDAC7	-0.275865485	1.90E-10	1.17E-09
HDAC8	0.010519835	0.811757	0.849676
HDAC9	-0.140340143	0.001409	0.002917
HDC	-0.387836845	6.23E-20	1.60E-18
HDDC2	0.144325491	0.001022	0.002171
HDDC3	0.141677469	0.001266	0.002644
HDGFL1	-0.043299072	0.326748	0.400015
HDGFRP2	-0.07857345	0.074825	0.109914
HDGFRP3	-0.019942571	0.651619	0.711414
HDGF	0.375871501	9.99E-19	2.17E-17
HDHD1A	-0.034831224	0.430249	0.504981
HDHD2	-0.182866465	2.98E-05	8.26E-05
HDHD3	0.010062551	0.819797	0.856252
HDLBP	0.108132775	0.014082	0.024244
HDX	-0.053979359	0.221367	0.286146
HEATR1	0.300082165	3.54E-12	2.83E-11
HEATR2	0.33706327	3.79E-15	4.80E-14
HEATR3	0.176533639	5.62E-05	0.000149
HEATR4	0.064442799	0.144179	0.196955
HEATR5A	0.208218444	1.88E-06	6.31E-06
HEATR5B	-0.229847658	1.33E-07	5.38E-07
HEATR6	-0.010874364	0.805538	0.844448
HEATR7A	-0.177028899	5.35E-05	0.000142
HEATR7B2	0.076093876	0.0845	0.1224
HEBP1	-0.050918813	0.248717	0.316606
HEBP2	0.240108607	3.46E-08	1.52E-07
HECA	-0.23400996	7.77E-08	3.25E-07
HECTD1	0.051213028	0.245989	0.313654
HECTD2	0.005333901	0.903887	0.924706
HECTD3	-0.193122294	1.02E-05	3.04E-05
HECW1	0.042344469	0.337537	0.411533
HECW2	-0.112853426	0.010377	0.018355
HEG1	-0.126576669	0.004014	0.007661
HELB	0.117808473	0.007443	0.013536

HELLS	0.374102624	1.49E-18	3.16E-17
HELQ	-0.158147628	0.000315	0.000732
HELT	-0.173235246	7.76E-05	0.000201
HELZ	-0.240171637	3.43E-08	1.51E-07
HEMGN	-0.269115254	5.39E-10	3.12E-09
HEMK1	-0.321114013	8.13E-14	8.32E-13
HEPACAM2	0.084180382	0.056249	0.085045
HEPACAM	-0.231355662	1.10E-07	4.50E-07
HEPHL1	0.072158568	0.101904	0.144591
HEPH	-0.039832882	0.366997	0.44166
HEPN1	-0.114529951	0.009286	0.016584
HERC1	-0.373338376	1.77E-18	3.69E-17
HERC2P2	-0.306126895	1.23E-12	1.07E-11
HERC2P4	0.071870562	0.103282	0.146266
HERC2	-0.163274075	0.000198	0.00048
HERC3	-0.251201479	7.48E-09	3.65E-08
HERC4	0.160128117	0.000264	0.000623
HERC5	-0.13041257	0.003027	0.005906
HERC6	-0.213060034	1.06E-06	3.72E-06
HERPUD1	-0.150252198	0.000624	0.001377
HERPUD2	0.125559887	0.00432	0.008201
HES1	-0.016797034	0.703733	0.7589
HES2	0.001215641	0.978045	0.983754
HES3	0.003492826	0.936975	0.950432
HES4	0.113865235	0.009705	0.017282
HES5	-0.056839997	0.197811	0.259532
HES6	0.104603462	0.017569	0.029604
HES7	0.137667952	0.001739	0.003542
HESRG	0.103520654	0.01878	0.031473
HESX1	-0.21895054	5.23E-07	1.93E-06
HEXA	-0.066092817	0.134165	0.184766
HEXB	-0.087453518	0.047299	0.07269
HEXDC	-0.268991255	5.50E-10	3.18E-09
HEXIM1	-0.056755298	0.198482	0.260284
HEXIM2	-0.045858064	0.298943	0.37033
HEY1	0.00097834	0.98233	0.986823
HEY2	-0.1707041	9.90E-05	0.000252
HEYL	-0.169510061	0.000111	0.00028
HFE2	-0.059763998	0.17568	0.234303
HFE	-0.036302852	0.411013	0.485683
HFM1	-0.130843368	0.002931	0.005734
HGC6.3	-0.020596559	0.640984	0.702065
HGD	0.160197731	0.000262	0.000619
HGFAC	-0.036764502	0.405085	0.479841

HGF	-0.154590844	0.00043	0.000977
HGSNAT	-0.063749377	0.148554	0.202087
HGS	-0.007020758	0.873715	0.901405
HHATL	-0.212568289	1.13E-06	3.92E-06
HHAT	-0.279870975	1.01E-10	6.51E-10
HHEX	-0.138959533	0.001571	0.003224
HHIPL1	-0.06810305	0.1227	0.17065
HHIPL2	0.28435831	4.91E-11	3.30E-10
HHIP	-0.285611326	4.01E-11	2.73E-10
HHLA1	0.057370012	0.193656	0.254741
HHLA2	-0.217409419	6.31E-07	2.29E-06
HHLA3	-0.116210899	0.008296	0.014952
HIAT1	0.110519245	0.012084	0.021066
HIATL1	0.07372077	0.094682	0.135392
HIATL2	-0.082650814	0.06089	0.091389
HIBADH	0.211858035	1.23E-06	4.24E-06
HIBCH	-0.076777772	0.081736	0.118826
HIC1	-0.066597023	0.131214	0.181051
HIC2	-0.077363096	0.079429	0.115935
HIF1AN	-0.034556595	0.433896	0.508485
HIF1A	0.295543331	7.69E-12	5.87E-11
HIF3A	-0.20136007	4.11E-06	1.31E-05
HIGD1A	0.207632119	2.01E-06	6.71E-06
HIGD1B	-0.277203716	1.54E-10	9.64E-10
HIGD1C	-0.085503436	0.052475	0.079913
HIGD2A	-0.004985407	0.910139	0.929722
HIGD2B	-0.071721192	0.104002	0.147182
HILS1	-0.058977155	0.181446	0.240788
HINFP	0.008661776	0.844537	0.876869
HINT1	0.173249079	7.75E-05	0.0002
HINT2	-0.02564432	0.561481	0.630888
HINT3	-0.139232625	0.001538	0.003163
HIP1R	-0.10919123	0.013163	0.022786
HIP1	-0.329301743	1.72E-14	1.96E-13
HIPK1	-0.228783924	1.53E-07	6.10E-07
HIPK2	0.024070721	0.585751	0.652329
HIPK3	-0.085091414	0.053627	0.0815
HIPK4	0.060406312	0.171075	0.228833
HIRA	-0.034784026	0.430875	0.505557
HIRIP3	-0.175806262	6.04E-05	0.000159
HIST1H1A	0.055701767	0.206955	0.270014
HIST1H1B	0.259510222	2.26E-09	1.19E-08
HIST1H1C	0.091418211	0.038087	0.059723
HIST1H1D	0.153707722	0.000464	0.001047

HIST1H1E	0.206288554	2.35E-06	7.76E-06
HIST1H1T	0.040700819	0.35664	0.431169
HIST1H2AA	0.056162988	0.203214	0.265839
HIST1H2AB	0.071089138	0.107095	0.151099
HIST1H2AC	0.116356086	0.008215	0.014821
HIST1H2AD	0.09635023	0.028793	0.046397
HIST1H2AE	0.163503104	0.000194	0.000471
HIST1H2AG	0.228903966	1.51E-07	6.02E-07
HIST1H2AH	0.163215803	0.000199	0.000482
HIST1H2AJ	0.177505394	5.11E-05	0.000136
HIST1H2AK	-0.020550323	0.641733	0.702547
HIST1H2AL	0.176722327	5.52E-05	0.000146
HIST1H2AM	0.210001935	1.53E-06	5.19E-06
HIST1H2BA	0.088025799	0.045864	0.070729
HIST1H2BB	0.082715331	0.060688	0.091116
HIST1H2BC	0.190239706	1.38E-05	4.06E-05
HIST1H2BD	0.200742468	4.40E-06	1.39E-05
HIST1H2BE	0.140077798	0.001438	0.002973
HIST1H2BF	0.172323517	8.47E-05	0.000218
HIST1H2BG	0.152947612	0.000496	0.001112
HIST1H2BH	0.198298583	5.78E-06	1.80E-05
HIST1H2BI	0.059519584	0.177456	0.236388
HIST1H2BJ	0.220165	4.51E-07	1.68E-06
HIST1H2BK	0.167829986	0.00013	0.000325
HIST1H2BL	0.173066525	7.89E-05	0.000204
HIST1H2BM	0.134191094	0.002276	0.004542
HIST1H2BN	0.187926821	1.77E-05	5.09E-05
HIST1H2BO	0.281092076	8.31E-11	5.42E-10
HIST1H3A	0.055062315	0.212224	0.275952
HIST1H3B	0.269286583	5.25E-10	3.04E-09
HIST1H3C	0.221702993	3.73E-07	1.40E-06
HIST1H3D	0.175719827	6.09E-05	0.00016
HIST1H3E	0.011348226	0.797245	0.837775
HIST1H3F	0.137358958	0.001782	0.003622
HIST1H3G	0.169114195	0.000115	0.00029
HIST1H3H	0.152741787	0.000505	0.00113
HIST1H3I	0.137688453	0.001736	0.003537
HIST1H3J	0.171141351	9.49E-05	0.000242
HIST1H4A	0.112505291	0.010617	0.018741
HIST1H4B	0.105502374	0.016616	0.028143
HIST1H4C	0.122358537	0.005428	0.010132
HIST1H4D	0.136524479	0.001901	0.003845
HIST1H4E	0.090289721	0.04054	0.063246
HIST1H4F	0.018551048	0.674483	0.732296

HIST1H4G	-0.007048848	0.873214	0.901074
HIST1H4H	0.117016892	0.007855	0.014227
HIST1H4I	0.026084589	0.554778	0.624305
HIST1H4J	0.069290795	0.116293	0.162664
HIST1H4K	0.025278123	0.567085	0.635649
HIST1H4L	0.105643214	0.016471	0.02793
HIST2H2AA3	0.167482434	0.000134	0.000334
HIST2H2AB	0.197695912	6.18E-06	1.91E-05
HIST2H2AC	0.178559331	4.60E-05	0.000124
HIST2H2BA	0.019341869	0.661451	0.720259
HIST2H2BE	0.080890168	0.066619	0.099059
HIST2H2BF	0.062803745	0.154682	0.209343
HIST2H3C	0.177263457	5.23E-05	0.000139
HIST2H3D	0.090410938	0.04027	0.06286
HIST2H4A	0.180329292	3.85E-05	0.000105
HIST3H2A	0.146563787	0.00085	0.00183
HIST3H2BB	0.002085024	0.962352	0.971098
HIST3H3	-0.139874345	0.001462	0.003017
HIST4H4	-0.159393971	0.000282	0.000662
HIVEP1	-0.144002554	0.001049	0.002222
HIVEP2	-0.080277245	0.068714	0.101814
HIVEP3	-0.267554519	6.84E-10	3.90E-09
HJURP	0.496461794	2.11E-33	4.39E-31
HK1	-0.050546759	0.252195	0.320381
HK2	0.112386355	0.0107	0.018877
HK3	-0.158746838	0.000298	0.000697
HKDC1	-0.058941444	0.181711	0.241091
HKR1	-0.201354819	4.11E-06	1.31E-05
HLA-A	-0.117785134	0.007455	0.013554
HLA-B	-0.189681401	1.47E-05	4.29E-05
HLA-C	-0.146548455	0.000851	0.001832
HLA-DMA	-0.397056384	6.79E-21	2.03E-19
HLA-DMB	-0.297235878	5.77E-12	4.47E-11
HLA-DOA	-0.381470191	2.77E-19	6.59E-18
HLA-DOB	-0.302175478	2.47E-12	2.02E-11
HLA-DPA1	-0.341191613	1.66E-15	2.23E-14
HLA-DPB1	-0.384109672	1.50E-19	3.70E-18
HLA-DPB2	-0.25260649	6.13E-09	3.02E-08
HLA-DQA1	-0.313268612	3.44E-13	3.23E-12
HLA-DQA2	-0.218127789	5.78E-07	2.11E-06
HLA-DQB1	-0.341991793	1.41E-15	1.91E-14
HLA-DQB2	-0.401749098	2.14E-21	6.95E-20
HLA-DRA	-0.303970099	1.80E-12	1.52E-11
HLA-DRB1	-0.357938856	5.18E-17	8.67E-16

HLA-DRB5	-0.324363842	4.42E-14	4.73E-13
HLA-DRB6	-0.178473272	4.64E-05	0.000124
HLA-E	-0.26619083	8.40E-10	4.72E-09
HLA-F	-0.238140255	4.50E-08	1.95E-07
HLA-G	-0.059761869	0.175696	0.234308
HLA-H	-0.089749668	0.04176	0.064992
HLA-J	-0.309536258	6.74E-13	6.05E-12
HLA-L	-0.295181748	8.18E-12	6.21E-11
HLCS	-0.128356518	0.003524	0.006794
HLF	-0.490880877	1.38E-32	2.42E-30
HLTF	0.205018221	2.72E-06	8.87E-06
HLX	-0.252302204	6.40E-09	3.14E-08
HM13	0.16846128	0.000122	0.000307
HMBOX1	0.003717464	0.932931	0.94758
HMBS	0.3019134	2.58E-12	2.10E-11
HMCN1	-0.33207697	1.01E-14	1.19E-13
HMG20A	-0.185709747	2.22E-05	6.30E-05
HMG20B	0.061227521	0.16532	0.222026
HMGA1	0.504551229	1.30E-34	3.41E-32
HMGA2	0.107902158	0.01429	0.024568
HMGB1	0.161682593	0.000229	0.000548
HMGB2	0.277384111	1.50E-10	9.39E-10
HMGB3L1	0.134630662	0.0022	0.004405
HMGB3	0.264207392	1.13E-09	6.23E-09
HMGB4	0.031423552	0.476739	0.550431
HMGCLL1	-0.318750821	1.26E-13	1.26E-12
HMGCL	-0.158958251	0.000293	0.000685
HMGCR	0.126574067	0.004014	0.007662
HMGCS1	0.056311614	0.202019	0.264506
HMGCS2	-0.100147252	0.023032	0.037899
HMGN1	0.072146053	0.101964	0.144666
HMGN2	0.09677218	0.028097	0.045397
HMGN3	-0.359697799	3.56E-17	6.11E-16
HMGN4	0.189114246	1.56E-05	4.54E-05
HMGN5	-0.103664122	0.018616	0.031221
HMGXB3	-0.069372001	0.115865	0.162155
HMGXB4	0.317666116	1.54E-13	1.52E-12
HMHA1	-0.355897987	7.99E-17	1.30E-15
HMHB1	0.073292185	0.096622	0.137925
HMMR	0.52311291	1.62E-37	7.54E-35
HMOX1	-0.007432165	0.866383	0.895319
HMOX2	-0.119536777	0.00661	0.012138
HMP19	0.021012395	0.634259	0.695729
HMSD	0.033181384	0.452422	0.526706

HMX1	-0.011517009	0.794296	0.83516
HMX2	0.188569858	1.65E-05	4.78E-05
HMX3	0.012103993	0.784065	0.826533
HN1L	0.194490446	8.77E-06	2.65E-05
HN1	0.365105165	1.10E-17	2.04E-16
HNF1A	0.121917808	0.005599	0.010427
HNF1B	-0.238837163	4.10E-08	1.79E-07
HNF4A	0.129455722	0.00325	0.006306
HNF4G	0.130198183	0.003075	0.005995
HNMT	-0.220935032	4.10E-07	1.53E-06
HNRNPA0	0.027305533	0.536397	0.607333
HNRNPA1L2	0.166220759	0.000151	0.000373
HNRNPA1	0.162681508	0.000209	0.000505
HNRNPA2B1	0.121843933	0.005628	0.010476
HNRNPA3P1	0.168597438	0.000121	0.000303
HNRNPA3	-0.005919128	0.893402	0.916512
HNRNPAB	0.218237705	5.70E-07	2.09E-06
HNRNPCL1	-0.019599301	0.65723	0.716561
HNRNPC	0.485508359	8.17E-32	1.27E-29
HNRNPD	0.124683296	0.004601	0.008698
HNRNPF	0.38414315	1.49E-19	3.68E-18
HNRNPH1	-0.196591001	6.97E-06	2.14E-05
HNRNPH2	0.025119108	0.569527	0.637798
HNRNPH3	0.022488672	0.610631	0.674884
HNRNPK	0.31501661	2.50E-13	2.40E-12
HNRNPL	0.310074065	6.12E-13	5.54E-12
HNRNPM	0.062246932	0.158379	0.213825
HNRNPR	0.300000917	3.59E-12	2.86E-11
HNRNPUL1	0.001389302	0.974909	0.981193
HNRNPUL2	-0.079177786	0.072609	0.106941
HNRNPU	0.100641863	0.022361	0.036892
HNRPDL	-0.114521649	0.009291	0.016591
HNRPLL	0.233318986	8.50E-08	3.54E-07
HOMER1	0.322823938	5.90E-14	6.17E-13
HOMER2	-0.197076366	6.61E-06	2.03E-05
HOMER3	0.056797768	0.198145	0.259877
HOMEZ	-0.025139845	0.569209	0.637565
HOOK1	-0.013246013	0.764267	0.810278
HOOK2	-0.267922856	6.46E-10	3.70E-09
HOOK3	-0.066066016	0.134323	0.184958
HOPX	-0.249440319	9.59E-09	4.61E-08
HORMAD1	0.11709817	0.007812	0.014154
HORMAD2	-0.008329111	0.850436	0.881895
HOTAIR	0.081519	0.064524	0.096189

HOXA10	0.14410375	0.00104	0.002205
HOXA11AS	0.142693406	0.001166	0.002451
HOXA11	0.207370642	2.07E-06	6.90E-06
HOXA13	0.096060196	0.02928	0.04711
HOXA1	0.15630818	0.00037	0.000849
HOXA2	-0.059300229	0.179062	0.238177
HOXA3	0.063190875	0.152151	0.206308
HOXA4	-0.071649058	0.104352	0.147614
HOXA5	-0.190349113	1.37E-05	4.01E-05
HOXA6	-0.079746036	0.070574	0.104267
HOXA7	-0.166112883	0.000153	0.000376
HOXA9	0.072743285	0.099151	0.141223
HOXB13	0.098560421	0.025306	0.04127
HOXB1	-0.018264633	0.679227	0.736487
HOXB2	0.009558934	0.828672	0.863672
HOXB3	-0.066760965	0.130266	0.179879
HOXB4	-0.088286776	0.045221	0.069836
HOXB5	0.037766168	0.3924	0.46728
HOXB6	0.047886498	0.278055	0.348624
HOXB7	0.156155745	0.000375	0.000859
HOXB8	0.107498605	0.01466	0.02515
HOXB9	0.183531076	2.78E-05	7.75E-05
HOXC10	0.124920263	0.004524	0.008557
HOXC11	0.105616909	0.016498	0.027964
HOXC12	0.099257047	0.024285	0.039742
HOXC13	0.103010889	0.019375	0.032375
HOXC4	0.079149586	0.072711	0.10706
HOXC5	0.138519303	0.001627	0.003329
HOXC6	0.18369309	2.74E-05	7.64E-05
HOXC8	0.188880583	1.60E-05	4.64E-05
HOXC9	0.185471555	2.28E-05	6.44E-05
HOXD10	0.150577636	0.000607	0.001342
HOXD11	0.175950993	5.95E-05	0.000157
HOXD12	0.135187447	0.002108	0.004237
HOXD13	0.146857327	0.000829	0.001789
HOXD1	-0.365496618	1.01E-17	1.89E-16
HOXD3	-0.159311161	0.000284	0.000666
HOXD4	-0.12297252	0.005198	0.009736
HOXD8	0.115457733	0.008727	0.015649
HOXD9	0.090498701	0.040076	0.062571
HP1BP3	-0.180136748	3.93E-05	0.000107
HPCAL1	-0.18154635	3.41E-05	9.35E-05
HPCAL4	-0.266976481	7.46E-10	4.23E-09
HPCA	-0.205485239	2.57E-06	8.45E-06

HPDL	0.347895657	4.25E-16	6.21E-15
HPD	0.05709231	0.195825	0.257273
HPGDS	-0.374841134	1.26E-18	2.70E-17
HPGD	-0.218306398	5.66E-07	2.07E-06
HPN	-0.189888939	1.44E-05	4.20E-05
HPRT1	0.353079551	1.45E-16	2.25E-15
HPR	-0.134905587	0.002154	0.004321
HPS1	-0.316698689	1.84E-13	1.80E-12
HPS3	0.125295159	0.004403	0.008349
HPS4	-0.217111924	6.54E-07	2.37E-06
HPS5	-0.02955826	0.503304	0.575979
HPS6	0.016433964	0.709845	0.764053
HPSE2	-0.332465622	9.34E-15	1.11E-13
HPSE	-0.055888643	0.205433	0.268291
HPVC1	0.032753484	0.458277	0.532127
HPX	0.016489109	0.708915	0.763253
HPYR1	-0.11164913	0.011229	0.019726
HP	-0.092621676	0.035612	0.056225
HRASLS2	-0.198312051	5.77E-06	1.79E-05
HRASLS5	-0.21319928	1.05E-06	3.66E-06
HRASLS	-0.036192883	0.412433	0.48713
HRAS	0.045363608	0.304189	0.375943
HRCT1	-0.106354262	0.015755	0.026828
HRC	-0.154464037	0.000435	0.000987
HRG	0.222659716	3.31E-07	1.26E-06
HRH1	0.07775171	0.077926	0.113933
HRH2	-0.010306946	0.815498	0.852965
HRH3	0.075031778	0.088942	0.12811
HRH4	-0.258308448	2.70E-09	1.39E-08
HRK	-0.029698847	0.501275	0.574248
HRNBP3	-0.095287746	0.030611	0.049039
HRNR	-0.115463761	0.008724	0.015644
HRSP12	0.256861722	3.33E-09	1.70E-08
HR	-0.279479281	1.07E-10	6.88E-10
HS1BP3	0.000873614	0.984221	0.9883
HS2ST1	0.215386317	8.05E-07	2.87E-06
HS3ST1	-0.109143729	0.013203	0.022844
HS3ST2	-0.189276225	1.53E-05	4.47E-05
HS3ST3A1	0.090054036	0.041069	0.064011
HS3ST3B1	-0.052115108	0.237758	0.304421
HS3ST4	-0.102367448	0.02015	0.033557
HS3ST5	-0.229373982	1.42E-07	5.70E-07
HS3ST6	-0.138973611	0.00157	0.003221
HS6ST1	-0.118010122	0.007342	0.013374

HS6ST2	-0.072123161	0.102073	0.144789
HS6ST3	-0.053456045	0.225884	0.291157
HSBP1L1	0.070684438	0.109113	0.153686
HSBP1	0.159925512	0.000268	0.000633
HSCB	0.152864062	0.000499	0.001119
HSD11B1L	-0.174948119	6.57E-05	0.000172
HSD11B1	0.004468014	0.919432	0.93682
HSD11B2	-0.152395409	0.00052	0.001162
HSD17B10	0.227929859	1.70E-07	6.76E-07
HSD17B11	-0.14002787	0.001444	0.002984
HSD17B12	0.058814918	0.182652	0.242083
HSD17B13	-0.273789652	2.63E-10	1.59E-09
HSD17B14	-0.176338009	5.73E-05	0.000151
HSD17B1	0.071344826	0.105835	0.149459
HSD17B2	-0.138806725	0.00159	0.003259
HSD17B3	-0.258492091	2.63E-09	1.36E-08
HSD17B4	-0.296571796	6.46E-12	4.97E-11
HSD17B6	-0.374335531	1.42E-18	3.02E-17
HSD17B7P2	-0.202425275	3.65E-06	1.17E-05
HSD17B7	-0.054418312	0.217628	0.281969
HSD17B8	-0.106868759	0.015254	0.02605
HSD3B1	0.089841917	0.041549	0.064695
HSD3B2	-0.077055737	0.080634	0.11748
HSD3B7	0.058460621	0.185307	0.245157
HSDL1	-6.78E-06	0.999878	0.999963
HSDL2	-0.182460809	3.10E-05	8.58E-05
HSF1	0.220974997	4.08E-07	1.53E-06
HSF2BP	0.324154761	4.59E-14	4.89E-13
HSF2	-0.006205697	0.888274	0.912459
HSF4	-0.247569129	1.24E-08	5.86E-08
HSF5	-0.202874175	3.47E-06	1.11E-05
HSFX2	-0.388062918	5.91E-20	1.53E-18
HSFY2	-0.033492067	0.448198	0.522428
HSFYL1	0.025496799	0.563735	0.632827
HSH2D	-0.150359547	0.000618	0.001365
HSN2	0.096371518	0.028757	0.046344
HSP90AA1	0.367878825	5.99E-18	1.16E-16
HSP90AB1	0.240950546	3.09E-08	1.37E-07
HSP90AB2P	0.192177337	1.13E-05	3.34E-05
HSP90AB4P	0.0298457	0.49916	0.572219
HSP90B1	0.308330974	8.36E-13	7.41E-12
HSP90B3P	0.18575044	2.21E-05	6.28E-05
HSPA12A	-0.113709412	0.009806	0.017446
HSPA12B	-0.262070175	1.55E-09	8.37E-09

HSPA13	0.337610065	3.40E-15	4.34E-14
HSPA14	0.334740591	5.98E-15	7.37E-14
HSPA1A	0.132867546	0.002517	0.004983
HSPA1B	0.17767681	5.02E-05	0.000134
HSPA1L	-0.136958781	0.001838	0.003728
HSPA2	0.053661807	0.2241	0.289231
HSPA4L	0.20911509	1.69E-06	5.73E-06
HSPA4	0.308212228	8.53E-13	7.55E-12
HSPA5	0.219939969	4.63E-07	1.72E-06
HSPA6	-0.049482561	0.262332	0.331301
HSPA7	-0.227648812	1.77E-07	6.99E-07
HSPA8	0.27402582	2.53E-10	1.54E-09
HSPA9	0.311597487	4.66E-13	4.28E-12
HSPB11	-0.03284277	0.457052	0.531106
HSPB1	-0.033589785	0.446875	0.521158
HSPB2	-0.160251511	0.000261	0.000616
HSPB3	-0.229485145	1.40E-07	5.62E-07
HSPB6	-0.266743897	7.73E-10	4.37E-09
HSPB7	-0.326979329	2.69E-14	2.98E-13
HSPB8	-0.274189129	2.47E-10	1.50E-09
HSPB9	-0.140230766	0.001421	0.00294
HSPBAP1	-0.12176229	0.005661	0.010529
HSPBP1	0.086783923	0.049025	0.075142
HSPC072	0.072653624	0.09957	0.141742
HSPC157	-0.147888332	0.000761	0.001654
HSPC159	-0.221215485	3.96E-07	1.48E-06
HSPD1	0.519779065	5.56E-37	2.26E-34
HSPE1	0.421004316	1.53E-23	6.81E-22
HSPG2	-0.294169341	9.70E-12	7.29E-11
HSPH1	0.173508289	7.56E-05	0.000196
HTATIP2	0.317310119	1.65E-13	1.61E-12
HTATSF1	-0.088236604	0.045344	0.069994
HTA	0.028391166	0.520314	0.592392
HTN1	-0.035117012	0.426474	0.501138
HTR1A	-0.03091857	0.483855	0.557551
HTR1B	0.098233343	0.025798	0.042007
HTR1D	0.222108897	3.54E-07	1.34E-06
HTR1E	-0.075707504	0.086095	0.124437
HTR1F	-0.011630541	0.792314	0.833515
HTR2A	-0.071635481	0.104418	0.147665
HTR2B	-0.085426179	0.052689	0.080197
HTR2C	0.16922925	0.000114	0.000287
HTR3A	0.03152273	0.475349	0.549079
HTR3B	0.107554165	0.014608	0.025068

HTR3C	-0.22766307	1.76E-07	6.98E-07
HTR3D	0.020584046	0.641186	0.702168
HTR3E	-0.009202616	0.834965	0.868793
HTR4	-0.261946712	1.58E-09	8.51E-09
HTR5A	-0.068779685	0.119017	0.165992
HTR6	-0.089008915	0.043484	0.067366
HTR7P1	-0.159634743	0.000276	0.000649
HTR7	-0.093761982	0.033394	0.053
HTRA1	-0.052497164	0.234331	0.300765
HTRA2	0.164277966	0.000181	0.000441
HTRA3	0.08911853	0.043225	0.066996
HTRA4	-0.199397376	5.12E-06	1.60E-05
HTT	-0.190753946	1.31E-05	3.86E-05
HULC	-0.057043729	0.196206	0.257689
HUNK	-0.246873177	1.37E-08	6.42E-08
HUS1B	0.099563949	0.023847	0.039081
HUS1	0.141303526	0.001304	0.002717
HUWE1	0.03799122	0.389583	0.464396
HVCN1	-0.247917033	1.19E-08	5.61E-08
HYAL1	-0.019403589	0.660438	0.71947
HYAL2	0.006764124	0.878295	0.90489
HYAL3	0.045858875	0.298934	0.37033
HYAL4	0.031697367	0.472905	0.546668
HYALP1	0.03716908	0.399932	0.47456
HYDIN	-0.201448976	4.07E-06	1.29E-05
HYI	-0.30649828	1.16E-12	1.00E-11
HYLS1	0.386521033	8.50E-20	2.14E-18
HYMAI	-0.137483501	0.001764	0.003588
HYOU1	0.164770201	0.000173	0.000423
IAH1	0.181190412	3.53E-05	9.68E-05
IAPP	0.120133276	0.006343	0.011682
IARS2	0.283644434	5.51E-11	3.68E-10
IARS	0.402306581	1.86E-21	6.13E-20
IBSP	0.180854234	3.65E-05	9.98E-05
IBTK	0.158244861	0.000312	0.000726
ICA1L	-0.271605253	3.68E-10	2.18E-09
ICA1	0.033356427	0.45004	0.524391
ICAM1	-0.283675311	5.48E-11	3.66E-10
ICAM2	-0.145652284	0.000916	0.00196
ICAM3	-0.160756052	0.000249	0.000591
ICAM4	-0.363657845	1.51E-17	2.74E-16
ICAM5	-0.4480585	8.50E-27	6.04E-25
ICK	-0.00505662	0.908861	0.928654
ICMT	0.118792825	0.006958	0.01272

ICOSLG	-0.223085293	3.14E-07	1.20E-06
ICOS	-0.115534269	0.008682	0.015576
ICT1	0.255027187	4.34E-09	2.18E-08
ID1	0.06031354	0.171735	0.229608
ID2B	-0.014160757	0.748518	0.796963
ID2	-0.091126845	0.038708	0.060616
ID3	0.03377038	0.444434	0.518705
ID4	-0.197012621	6.66E-06	2.05E-05
IDE	0.171072803	9.55E-05	0.000244
IDH1	0.192679371	1.07E-05	3.18E-05
IDH2	0.112679011	0.010496	0.018547
IDH3A	0.129423604	0.003257	0.006318
IDH3B	0.047450788	0.282456	0.353167
IDH3G	0.03908491	0.37607	0.450815
IDI1	0.157326412	0.000338	0.000782
IDI2	0.099748297	0.023587	0.038704
IDO1	-0.015879037	0.719221	0.771755
IDO2	-0.309015753	7.40E-13	6.61E-12
IDS	-0.132643279	0.00256	0.005062
IDUA	-0.339371462	2.39E-15	3.14E-14
IER2	-0.192816806	1.05E-05	3.14E-05
IER3IP1	0.169716005	0.000109	0.000275
IER3	0.11235357	0.010723	0.018912
IER5L	0.136093579	0.001966	0.00397
IER5	0.119345635	0.006698	0.012289
IFFO1	-0.255984324	3.78E-09	1.91E-08
IFFO2	-0.24891058	1.03E-08	4.93E-08
IFI16	-0.073702102	0.094766	0.135492
IFI27L1	0.054584606	0.216223	0.280568
IFI27L2	-0.13932147	0.001527	0.003143
IFI27	-0.04616202	0.295748	0.367063
IFI30	-0.069463283	0.115385	0.161585
IFI35	-0.006776282	0.878078	0.904738
IFI44L	-0.096244412	0.02897	0.04666
IFI44	-0.126612706	0.004003	0.007643
IFI6	-0.096583307	0.028407	0.045853
IFIH1	-0.069933078	0.112939	0.15855
IFIT1B	-0.023112467	0.600765	0.666032
IFIT1	-0.147694655	0.000774	0.001679
IFIT2	-0.147141486	0.00081	0.001752
IFIT3	-0.057856447	0.189898	0.250474
IFIT5	-0.155142136	0.00041	0.000933
IFITM1	-0.035572901	0.42049	0.495126
IFITM2	-0.080761354	0.067055	0.09962

IFITM3	0.005641933	0.898366	0.920591
IFITM4P	0.012571159	0.775948	0.819663
IFITM5	-0.009276208	0.833665	0.867789
IFLTD1	-0.201014196	4.27E-06	1.36E-05
IFNA10	0.003347794	0.939587	0.952395
IFNA13	0.072996459	0.097978	0.139705
IFNA14	-0.030767205	0.485998	0.559445
IFNA16	0.016979139	0.700675	0.756174
IFNA1	0.101422466	0.021336	0.035362
IFNA21	-0.084764505	0.054556	0.082788
IFNA2	0.102248181	0.020296	0.033767
IFNA4	0.069152934	0.117023	0.16357
IFNA5	-0.044426528	0.314298	0.386548
IFNA7	-0.087922812	0.046119	0.07109
IFNA8	0.043018481	0.329896	0.4033
IFNAR1	-0.215370298	8.07E-07	2.87E-06
IFNAR2	-0.182885936	2.97E-05	8.24E-05
IFNB1	-0.028399653	0.520189	0.592283
IFNE	0.097929313	0.026262	0.042698
IFNGR1	-0.113360272	0.010035	0.017814
IFNGR2	-0.041527779	0.346945	0.421334
IFNG	0.133428197	0.002412	0.004794
IFNK	-0.088267062	0.04527	0.069894
IFNW1	-0.083786918	0.057413	0.086603
IFRD1	0.229159015	1.46E-07	5.84E-07
IFRD2	0.123020613	0.00518	0.009705
IFT122	-0.236371141	5.69E-08	2.43E-07
IFT140	-0.426355428	3.67E-24	1.82E-22
IFT172	-0.431139943	1.00E-24	5.32E-23
IFT20	0.085451053	0.05262	0.080104
IFT27	-0.150924843	0.000589	0.001305
IFT46	-0.116358136	0.008214	0.014821
IFT52	0.072080866	0.102274	0.145034
IFT57	-0.333747086	7.27E-15	8.79E-14
IFT74	-0.105635661	0.016478	0.027936
IFT80	-0.30760958	9.50E-13	8.35E-12
IFT81	0.104082396	0.018143	0.030502
IFT88	-0.108572144	0.013694	0.023624
IGBP1	0.089497488	0.04234	0.065752
IGDCC3	0.017103845	0.698584	0.75421
IGDCC4	0.00642297	0.884389	0.909413
IGF1R	-0.032837544	0.457123	0.531127
IGF1	-0.055640935	0.207452	0.27061
IGF2AS	-0.029249439	0.507776	0.580316

IGF2BP1	0.280839003	8.65E-11	5.63E-10
IGF2BP2	0.139630574	0.00149	0.003072
IGF2BP3	0.319002646	1.20E-13	1.21E-12
IGF2R	-0.096710571	0.028198	0.045541
IGF2	-0.144206072	0.001032	0.002189
IGFALS	-0.306153014	1.23E-12	1.06E-11
IGFBP1	0.260770209	1.88E-09	9.98E-09
IGFBP2	-0.098674987	0.025135	0.04101
IGFBP3	0.16069713	0.00025	0.000594
IGFBP4	-0.207359419	2.07E-06	6.91E-06
IGFBP5	0.039091418	0.37599	0.450774
IGFBP6	-0.11391257	0.009675	0.017234
IGFBP7	-0.161057256	0.000242	0.000577
IGFBPL1	0.006835395	0.877023	0.903931
IGFL1	-0.010839554	0.806148	0.844999
IGFL2	-0.005640507	0.898392	0.920591
IGFL3	-0.067032089	0.128709	0.177889
IGFL4	0.04818994	0.275017	0.345335
IGFN1	-0.307758337	9.25E-13	8.15E-12
IGHMBP2	-0.072619149	0.099731	0.141941
IGJ	-0.091871172	0.037139	0.058378
IGLL1	-0.068436454	0.120874	0.168341
IGLL3	-0.208544172	1.81E-06	6.10E-06
IGLON5	0.173053573	7.90E-05	0.000204
IGSF10	-0.377942815	6.23E-19	1.39E-17
IGSF11	0.051639669	0.242072	0.30931
IGSF1	0.051064699	0.247362	0.315142
IGSF21	-0.123928664	0.004856	0.009149
IGSF22	-0.144413037	0.001014	0.002156
IGSF3	-0.276086419	1.84E-10	1.14E-09
IGSF5	-0.036339872	0.410536	0.485195
IGSF6	-0.18419187	2.60E-05	7.29E-05
IGSF8	-0.097073847	0.027608	0.044676
IGSF9B	-0.358423441	4.67E-17	7.85E-16
IGSF9	-0.003091642	0.944201	0.955725
IHH	-0.200227317	4.67E-06	1.47E-05
IKBIP	0.293303767	1.12E-11	8.33E-11
IKBKAP	-0.069998165	0.112604	0.158101
IKBKB	-0.314333063	2.84E-13	2.69E-12
IKBKE	-0.141679095	0.001266	0.002644
IKBKG	-0.085367745	0.052852	0.080396
IKZF1	-0.265619702	9.15E-10	5.12E-09
IKZF2	-0.27233244	3.29E-10	1.96E-09
IKZF3	-0.172208363	8.57E-05	0.00022

IKZF4	-0.219534932	4.87E-07	1.80E-06
IKZF5	-0.093158543	0.034552	0.054686
IK	-0.197242973	6.49E-06	2.00E-05
IL10RA	-0.234449159	7.33E-08	3.08E-07
IL10RB	-0.057387582	0.193519	0.254628
IL10	-0.06165876	0.162357	0.218472
IL11RA	-0.484229602	1.24E-31	1.85E-29
IL11	0.203010812	3.41E-06	1.10E-05
IL12A	-0.044040976	0.31852	0.391163
IL12B	-0.333587866	7.50E-15	9.03E-14
IL12RB1	-0.132074943	0.002672	0.005269
IL12RB2	0.187778825	1.79E-05	5.17E-05
IL13RA1	0.002060444	0.962796	0.971398
IL13RA2	-0.155741541	0.000389	0.000889
IL13	-0.069604266	0.114647	0.160676
IL15RA	0.058744713	0.183176	0.242696
IL15	0.04329293	0.326817	0.400075
IL16	-0.328180562	2.14E-14	2.40E-13
IL17A	0.010175919	0.817802	0.85457
IL17B	0.034652303	0.432623	0.507261
IL17C	0.203316681	3.30E-06	1.06E-05
IL17D	-0.054750506	0.214828	0.278975
IL17F	-0.125866668	0.004226	0.008032
IL17RA	-0.216756334	6.83E-07	2.46E-06
IL17RB	-0.050522862	0.25242	0.320606
IL17RC	-0.227491474	1.80E-07	7.13E-07
IL17RD	0.056259297	0.202439	0.265004
IL17REL	-0.161757486	0.000228	0.000545
IL17RE	-0.182437539	3.11E-05	8.60E-05
IL18BP	-0.100991609	0.021897	0.036198
IL18R1	-0.046640534	0.290765	0.361881
IL18RAP	-0.064180421	0.145823	0.198909
IL18	-0.071046935	0.107304	0.151351
IL19	-0.007675311	0.862054	0.89151
IL1A	0.085623654	0.052142	0.079438
IL1B	-0.015037063	0.733529	0.784162
IL1F10	0.004612182	0.916841	0.934885
IL1F5	0.112715614	0.010471	0.018509
IL1F6	-0.100241647	0.022903	0.037709
IL1F7	-0.278851426	1.19E-10	7.56E-10
IL1F8	0.103687473	0.018589	0.031184
IL1F9	-0.083249277	0.059037	0.088825
IL1R1	-0.212956894	1.08E-06	3.75E-06
IL1R2	0.346195755	6.03E-16	8.61E-15

IL1RAPL1	0.019768223	0.654466	0.714093
IL1RAPL2	0.008696923	0.843915	0.87645
IL1RAP	-0.024257787	0.582841	0.649775
IL1RL1	-0.041257417	0.350096	0.424696
IL1RL2	0.002241755	0.959525	0.968929
IL1RN	0.004097451	0.926094	0.942266
IL20RA	0.002271681	0.958985	0.968531
IL20RB	0.187525363	1.84E-05	5.29E-05
IL20	-0.13388378	0.00233	0.004641
IL21R	-0.109924395	0.012557	0.021812
IL21	0.084142459	0.05636	0.085194
IL22RA1	0.019319177	0.661823	0.720586
IL22RA2	-0.130530317	0.003	0.005858
IL22	-0.094508805	0.032006	0.05102
IL23A	0.069536632	0.115	0.161126
IL23R	-0.112191016	0.010838	0.019103
IL24	-0.076583703	0.082513	0.11986
IL25	-0.113429791	0.009989	0.01774
IL26	-0.051272426	0.245441	0.313075
IL27RA	-0.17115244	9.48E-05	0.000242
IL27	0.075454105	0.087154	0.125852
IL28A	-0.005813693	0.895289	0.918103
IL28B	0.046258437	0.294739	0.366098
IL28RA	-0.345678655	6.70E-16	9.48E-15
IL29	0.004267206	0.923041	0.939827
IL2RA	0.064981076	0.140851	0.192857
IL2RB	0.002000603	0.963876	0.972144
IL2RG	-0.154200485	0.000445	0.001007
IL2	-0.218773616	5.34E-07	1.96E-06
IL31RA	0.132843335	0.002521	0.004992
IL31	0.031697756	0.4729	0.546668
IL32	0.042931197	0.330879	0.404428
IL33	-0.308118825	8.68E-13	7.66E-12
IL34	-0.395484954	9.95E-21	2.89E-19
IL3RA	-0.268877816	5.59E-10	3.23E-09
IL3	-0.024826837	0.574029	0.641761
IL4I1	-0.024453892	0.579797	0.646884
IL4R	-0.1376988	0.001735	0.003534
IL4	-0.079243266	0.072372	0.106647
IL5RA	-0.224321481	2.69E-07	1.03E-06
IL5	-0.029310463	0.506891	0.579544
IL6R	-0.289440461	2.14E-11	1.52E-10
IL6ST	-0.134970129	0.002144	0.004302
IL6	0.167986796	0.000128	0.00032

IL7R	-0.176210333	5.80E-05	0.000153
IL7	-0.103299763	0.019036	0.031867
IL8	0.326628658	2.87E-14	3.17E-13
IL9R	-0.155122188	0.000411	0.000935
IL9	0.092055971	0.036758	0.057834
ILDR1	-0.328451447	2.03E-14	2.28E-13
ILDR2	-0.231905237	1.02E-07	4.20E-07
ILF2	0.397728217	5.76E-21	1.74E-19
ILF3	-0.0285443	0.518065	0.590269
ILKAP	0.035457035	0.422006	0.49657
ILK	-0.052566373	0.233714	0.300069
ILVBL	0.050682467	0.250923	0.319028
IMMP1L	0.102467101	0.020028	0.033368
IMMP2L	0.094707069	0.031646	0.050526
IMMT	0.325022926	3.90E-14	4.22E-13
IMP3	0.051475146	0.243577	0.310975
IMP4	0.22776785	1.74E-07	6.89E-07
IMP5	-0.013447836	0.760783	0.807313
IMPA1	0.310357304	5.82E-13	5.29E-12
IMPA2	0.097146864	0.027491	0.044501
IMPACT	0.030754078	0.486185	0.559623
IMPAD1	0.268120412	6.27E-10	3.60E-09
IMPDH1	0.049207941	0.264993	0.334281
IMPDH2	0.227316707	1.84E-07	7.27E-07
IMPG1	-0.303624571	1.92E-12	1.60E-11
IMPG2	-0.109930942	0.012552	0.021805
INADL	-0.152723253	0.000505	0.001132
INA	0.085735462	0.051835	0.079036
INCA1	-0.310264057	5.92E-13	5.37E-12
INCENP	0.314591843	2.71E-13	2.58E-12
INE1	-0.274582999	2.32E-10	1.42E-09
INE2	-0.230116911	1.29E-07	5.22E-07
INF2	0.026403986	0.54994	0.620176
ING1	0.034224538	0.438329	0.512538
ING2	0.084707802	0.054718	0.082995
ING3	-0.053554273	0.225031	0.290273
ING4	-0.135378749	0.002077	0.00418
ING5	-0.24573577	1.61E-08	7.44E-08
INGX	0.107779335	0.014401	0.024738
INHA	0.20225635	3.72E-06	1.19E-05
INHBA	0.189458106	1.50E-05	4.38E-05
INHBB	-0.121235886	0.005873	0.010887
INHBC	0.065532011	0.137506	0.188834
INHBE	0.277333785	1.51E-10	9.46E-10

INMT	-0.417778957	3.58E-23	1.51E-21
INO80B	-0.029282315	0.507299	0.57992
INO80C	0.072469891	0.100431	0.142856
INO80D	-0.210476468	1.44E-06	4.93E-06
INO80E	-0.083537144	0.058163	0.087595
INO80	-0.211919857	1.22E-06	4.21E-06
INPP1	-0.152207954	0.000528	0.00118
INPP4A	-0.280806959	8.70E-11	5.66E-10
INPP4B	0.182914855	2.96E-05	8.22E-05
INPP5A	-0.191366456	1.23E-05	3.63E-05
INPP5B	-0.451209691	3.39E-27	2.57E-25
INPP5D	-0.280246879	9.51E-11	6.15E-10
INPP5E	-0.228207427	1.65E-07	6.54E-07
INPP5F	-0.029601502	0.50268	0.575515
INPP5J	-0.379908938	3.97E-19	9.19E-18
INPP5K	-0.340710676	1.83E-15	2.44E-14
INPPL1	-0.137004539	0.001831	0.003716
INS-IGF2	-0.079646555	0.070927	0.10475
INSC	0.017191433	0.697116	0.752861
INSIG1	-0.031290171	0.478613	0.552467
INSIG2	-0.085500952	0.052482	0.079918
INSL3	-0.005664784	0.897957	0.92024
INSL4	0.288489853	2.50E-11	1.76E-10
INSL5	-0.126678711	0.003984	0.007612
INSL6	0.018664454	0.672608	0.730419
INSM1	-0.020063646	0.649645	0.709585
INSM2	-0.128502473	0.003487	0.006727
INSRR	-0.034023087	0.441031	0.515335
INSR	-0.09591325	0.029529	0.047488
INS	-0.005307909	0.904353	0.925047
INTS10	0.049781618	0.259456	0.32829
INTS12	0.074845147	0.089742	0.129103
INTS1	-0.006162003	0.889055	0.913084
INTS2	0.18017946	3.91E-05	0.000106
INTS3	-0.198244541	5.81E-06	1.81E-05
INTS4L1	-0.01755022	0.691116	0.747391
INTS4L2	0.00440845	0.920502	0.937715
INTS4	0.106230505	0.015877	0.027004
INTS5	-0.058026046	0.188601	0.249043
INTS6	0.2540385	5.00E-09	2.49E-08
INTS7	0.365511491	1.01E-17	1.89E-16
INTS8	0.417398689	3.96E-23	1.66E-21
INTS9	-0.137927067	0.001704	0.003478
INTU	-0.155060526	0.000413	0.00094

INVS	0.080009698	0.069646	0.103071
IP6K1	-0.149589771	0.00066	0.001451
IP6K2	-0.139037234	0.001562	0.003207
IP6K3	-0.050345529	0.254091	0.322605
IPCEF1	-0.287127632	3.13E-11	2.17E-10
IPMK	0.211338865	1.30E-06	4.49E-06
IPO11	0.186793216	1.99E-05	5.68E-05
IPO13	-0.185446546	2.29E-05	6.46E-05
IPO4	0.337363577	3.57E-15	4.54E-14
IPO5	0.340230695	2.02E-15	2.67E-14
IPO7	0.286688503	3.36E-11	2.32E-10
IPO8	0.226524496	2.04E-07	8.00E-07
IPO9	0.133567774	0.002386	0.004747
IPPK	0.17367636	7.44E-05	0.000193
IPP	-0.013883375	0.753283	0.800969
IPW	-0.170844279	9.77E-05	0.000249
IQCA1	-0.257524458	3.02E-09	1.55E-08
IQCB1	-0.094138567	0.032688	0.052015
IQCC	-0.15049409	0.000611	0.00135
IQCD	-0.011531283	0.794047	0.834942
IQCE	-0.102570101	0.019903	0.033176
IQCF1	-0.113561094	0.009903	0.017607
IQCF2	-0.040750264	0.356056	0.430541
IQCF3	-0.044325948	0.315395	0.387755
IQCF5	0.119154762	0.006787	0.012438
IQCF6	-0.00966848	0.82674	0.862063
IQCG	-0.213354172	1.03E-06	3.60E-06
IQCH	-0.067834956	0.124183	0.172288
IQCJ	0.111166309	0.011587	0.020288
IQCK	-0.193144418	1.01E-05	3.03E-05
IQGAP1	-0.153545764	0.000471	0.00106
IQGAP2	-0.288969917	2.31E-11	1.63E-10
IQGAP3	0.319523736	1.09E-13	1.10E-12
IQSEC1	-0.355613464	8.49E-17	1.37E-15
IQSEC2	-0.273544076	2.73E-10	1.64E-09
IQSEC3	-0.187643193	1.82E-05	5.24E-05
IQUB	-0.15851064	0.000305	0.000711
IRAK1BP1	-0.098604381	0.02524	0.041177
IRAK1	0.278694094	1.22E-10	7.74E-10
IRAK2	0.075666735	0.086265	0.124639
IRAK3	-0.164414503	0.000179	0.000436
IRAK4	-0.029116684	0.509705	0.582171
IREB2	0.062241635	0.158415	0.213859
IRF1	0.047593026	0.281014	0.35165

IRF2BP1	-0.021846857	0.620855	0.684029
IRF2BP2	-0.165740833	0.000158	0.000389
IRF2	-0.229927095	1.32E-07	5.34E-07
IRF3	-0.22064211	4.25E-07	1.59E-06
IRF4	-0.12799363	0.003619	0.006958
IRF5	-0.192278818	1.11E-05	3.31E-05
IRF6	-0.060520557	0.170266	0.227903
IRF7	-0.101144026	0.021697	0.035897
IRF8	-0.249109659	1.00E-08	4.81E-08
IRF9	-0.101128691	0.021717	0.035924
IRGC	0.006480375	0.883363	0.908592
IRGM	-0.107483729	0.014673	0.025171
IRGQ	-0.097940056	0.026246	0.042674
IRS1	0.009265462	0.833855	0.867897
IRS2	0.079487781	0.071493	0.105523
IRS4	-0.064091285	0.146384	0.199491
IRX1	-0.343130841	1.12E-15	1.54E-14
IRX2	-0.387646465	6.52E-20	1.67E-18
IRX3	-0.281781493	7.44E-11	4.88E-10
IRX4	0.069299023	0.116249	0.162614
IRX5	-0.309572675	6.70E-13	6.02E-12
IRX6	-0.330254289	1.43E-14	1.65E-13
ISCA1P1	0.278180123	1.32E-10	8.35E-10
ISCA1	0.182705816	3.03E-05	8.39E-05
ISCA2	0.163717452	0.00019	0.000462
ISCU	-0.287981105	2.72E-11	1.90E-10
ISG15	0.087450569	0.047306	0.072696
ISG20L2	0.283714918	5.45E-11	3.64E-10
ISG20	0.100993353	0.021894	0.036197
ISL1	-0.063859063	0.147855	0.201248
ISL2	0.162201907	0.000219	0.000525
ISLR2	-0.337203309	3.68E-15	4.68E-14
ISLR	-0.086763721	0.049078	0.075211
ISM1	-0.221781798	3.69E-07	1.39E-06
ISM2	0.17133245	9.32E-05	0.000238
ISOC1	0.014576426	0.741396	0.790727
ISOC2	0.045797786	0.299579	0.371026
ISPD	0.024668013	0.576482	0.644051
ISX	0.04578726	0.29969	0.371118
ISY1	0.157738828	0.000326	0.000757
ISYNA1	-0.120582774	0.006147	0.011351
ITCH	0.220134734	4.52E-07	1.68E-06
ITFG1	-0.010380724	0.814201	0.851877
ITFG2	-0.183519564	2.79E-05	7.76E-05

ITFG3	-0.155153529	0.00041	0.000933
ITGA10	-0.308862452	7.60E-13	6.78E-12
ITGA11	0.104628115	0.017542	0.029562
ITGA1	-0.031202077	0.479853	0.553738
ITGA2B	-0.243866214	2.08E-08	9.47E-08
ITGA2	-0.026286303	0.55172	0.621666
ITGA3	-0.128983166	0.003365	0.006513
ITGA4	-0.213483036	1.01E-06	3.55E-06
ITGA5	0.225657663	2.27E-07	8.84E-07
ITGA6	0.059984825	0.174087	0.232424
ITGA7	-0.301897871	2.59E-12	2.11E-11
ITGA8	-0.332866539	8.63E-15	1.03E-13
ITGA9	-0.369024734	4.65E-18	9.14E-17
ITGAD	-0.209042668	1.71E-06	5.77E-06
ITGAE	0.153877447	0.000458	0.001033
ITGAL	-0.27940875	1.09E-10	6.94E-10
ITGAM	-0.264627083	1.06E-09	5.88E-09
ITGAV	0.116123225	0.008345	0.015035
ITGAX	-0.286533839	3.45E-11	2.38E-10
ITGB1BP1	0.221197609	3.97E-07	1.49E-06
ITGB1BP2	-0.155111763	0.000411	0.000936
ITGB1BP3	-0.07531568	0.087737	0.126602
ITGB1	0.377400146	7.05E-19	1.57E-17
ITGB2	-0.25931025	2.33E-09	1.22E-08
ITGB3BP	0.187478427	1.85E-05	5.32E-05
ITGB3	-0.097294509	0.027255	0.044141
ITGB4	-0.097789807	0.026478	0.043017
ITGB5	0.036177433	0.412632	0.487337
ITGB6	-0.185306749	2.32E-05	6.54E-05
ITGB7	-0.107123024	0.015011	0.025709
ITGB8	-0.145816551	0.000904	0.001936
ITGBL1	-0.182236406	3.18E-05	8.75E-05
ITIH1	-0.07639284	0.083283	0.120837
ITIH2	0.006732677	0.878856	0.905219
ITIH3	-0.225109414	2.44E-07	9.43E-07
ITIH4	-0.184336501	2.56E-05	7.19E-05
ITIH5L	0.090375859	0.040348	0.062965
ITIH5	-0.396595308	7.60E-21	2.26E-19
ITK	-0.196120833	7.34E-06	2.24E-05
ITLN1	-0.196892404	6.75E-06	2.07E-05
ITLN2	-0.1906335	1.33E-05	3.90E-05
ITM2A	-0.217363419	6.34E-07	2.30E-06
ITM2B	-0.182394191	3.13E-05	8.63E-05
ITM2C	-0.055902194	0.205323	0.268165

ITPA	0.116171456	0.008318	0.014989
ITPK1	0.01183887	0.788681	0.830436
ITPKA	0.120661703	0.006114	0.011294
ITPKB	-0.263653066	1.23E-09	6.71E-09
ITPKC	-0.031791017	0.471598	0.545345
ITPR1	-0.148171601	0.000744	0.00162
ITPR2	-0.255975174	3.78E-09	1.92E-08
ITPR3	-0.2658484	8.84E-10	4.95E-09
ITPRIPL1	-0.055274285	0.210467	0.27397
ITPRIPL2	0.062695203	0.155398	0.21024
ITPRIP	-0.160815039	0.000248	0.000589
ITSN1	-0.033604822	0.446671	0.521035
ITSN2	-0.298635258	4.54E-12	3.56E-11
IVD	-0.33825436	2.99E-15	3.86E-14
IVL	-0.204835098	2.77E-06	9.05E-06
IVNS1ABP	-0.014722701	0.738895	0.788758
IWS1	0.121106003	0.005927	0.010977
IYD	-0.15457107	0.000431	0.000978
IZUMO1	-0.168149255	0.000126	0.000316
JAG1	0.005825404	0.89508	0.917998
JAG2	0.035066734	0.427136	0.501758
JAGN1	0.222045853	3.57E-07	1.35E-06
JAK1	-0.181851896	3.30E-05	9.08E-05
JAK2	-0.120635633	0.006125	0.011311
JAK3	-0.147605834	0.000779	0.00169
JAKMIP1	-0.009091541	0.836929	0.870508
JAKMIP2	-0.061082073	0.166329	0.223186
JAKMIP3	-0.031064304	0.481795	0.555627
JAM2	-0.324348708	4.43E-14	4.74E-13
JAM3	-0.141074559	0.001328	0.002763
JARID2	0.068292878	0.121658	0.169326
JAZF1	-0.206039023	2.42E-06	7.96E-06
JDP2	-0.205702025	2.51E-06	8.26E-06
JHDM1D	-0.193829834	9.42E-06	2.83E-05
JKAMP	0.222267671	3.47E-07	1.31E-06
JMJD1C	-0.054690487	0.215332	0.279502
JMJD4	0.15879789	0.000297	0.000694
JMJD5	-0.261964838	1.58E-09	8.49E-09
JMJD6	0.180002265	3.98E-05	0.000108
JMJD7-PLA2G4B	-0.499615384	7.18E-34	1.63E-31
JMJD8	-0.241705955	2.79E-08	1.25E-07
JMY	-0.248027234	1.17E-08	5.52E-08
JOSD1	0.058267492	0.186765	0.246929
JOSD2	-0.091953762	0.036968	0.058133

JPH1	-0.18685296	1.97E-05	5.65E-05
JPH2	-0.161263295	0.000238	0.000567
JPH3	-0.036670126	0.406293	0.481099
JPH4	-0.413190341	1.18E-22	4.60E-21
JRKL	-0.014392321	0.744548	0.793522
JRK	-0.036858196	0.403888	0.478594
JSRP1	-0.005542471	0.900148	0.921966
JTB	0.134614794	0.002203	0.00441
JUB	0.018637414	0.673055	0.730864
JUNB	-0.174313499	6.99E-05	0.000182
JUND	-0.252279058	6.42E-09	3.15E-08
JUN	-0.296194289	6.89E-12	5.29E-11
JUP	0.022063257	0.6174	0.680897
KAAG1	0.089531043	0.042263	0.065662
KAL1	-0.346550973	5.60E-16	8.05E-15
KALRN	-0.186451781	2.06E-05	5.87E-05
KANK1	-0.249044176	1.01E-08	4.85E-08
KANK2	-0.389170836	4.54E-20	1.19E-18
KANK3	-0.400876905	2.65E-21	8.47E-20
KANK4	-0.08966295	0.041959	0.065256
KARS	0.279433171	1.08E-10	6.92E-10
KAT2A	-0.020448017	0.643393	0.703971
KAT2B	-0.379327818	4.54E-19	1.04E-17
KAT5	-0.084527542	0.055237	0.083693
KATNA1	0.105268684	0.016859	0.028517
KATNAL1	-0.032728821	0.458615	0.532458
KATNAL2	-0.248950159	1.03E-08	4.91E-08
KATNB1	-0.128407801	0.003511	0.006771
KAZALD1	0.185042744	2.38E-05	6.71E-05
KAZ	-0.35496705	9.73E-17	1.55E-15
KBTBD10	-0.217305622	6.39E-07	2.32E-06
KBTBD11	-0.099880367	0.023402	0.038435
KBTBD12	-0.03877987	0.37981	0.454507
KBTBD13	-0.183787819	2.71E-05	7.57E-05
KBTBD2	0.118919883	0.006897	0.012625
KBTBD3	-0.147673533	0.000775	0.001682
KBTBD4	-0.14143473	0.001291	0.002692
KBTBD5	-0.072533089	0.100134	0.142454
KBTBD6	0.090557384	0.039946	0.062389
KBTBD7	-0.055940329	0.205014	0.267814
KBTBD8	-0.13240445	0.002606	0.005145
KC6	0.082937679	0.059996	0.090131
KCMF1	0.428665297	1.96E-24	1.00E-22
KCNA10	-0.199828395	4.88E-06	1.53E-05

KCNA1	-0.123827303	0.004891	0.009205
KCNA2	-0.212152329	1.18E-06	4.10E-06
KCNA3	-0.301499473	2.77E-12	2.25E-11
KCNA4	-0.31646268	1.92E-13	1.87E-12
KCNA5	-0.344543875	8.44E-16	1.18E-14
KCNA6	-0.234087248	7.69E-08	3.22E-07
KCNA7	0.00038237	0.993093	0.994906
KCNAB1	-0.218786507	5.33E-07	1.96E-06
KCNAB2	-0.318098947	1.42E-13	1.41E-12
KCNAB3	-0.28726871	3.06E-11	2.12E-10
KCNB1	-0.128493045	0.003489	0.00673
KCNB2	-0.110437471	0.012148	0.021166
KCNC1	-0.101021725	0.021857	0.036141
KCNC2	0.165146757	0.000167	0.000409
KCNC3	-0.359782891	3.49E-17	6.02E-16
KCNC4	-0.267388798	7.01E-10	3.99E-09
KCND1	-0.187823776	1.78E-05	5.15E-05
KCND2	0.336147786	4.54E-15	5.67E-14
KCND3	-0.277139758	1.56E-10	9.72E-10
KCNE1L	-0.117136279	0.007792	0.014122
KCNE1	-0.24753531	1.25E-08	5.89E-08
KCNE2	-0.109853252	0.012615	0.021905
KCNE3	0.131886034	0.00271	0.00534
KCNE4	0.056811259	0.198039	0.259788
KCNF1	0.293863385	1.02E-11	7.63E-11
KCNG1	0.058249056	0.186905	0.247081
KCNG2	0.027337728	0.535916	0.606941
KCNG3	-0.022721315	0.606943	0.671532
KCNG4	-0.00211342	0.96184	0.970826
KCNH1	-0.20295273	3.43E-06	1.10E-05
KCNH2	-0.115902544	0.00847	0.01523
KCNH3	-0.112803098	0.010411	0.018409
KCNH4	-0.164101048	0.000184	0.000448
KCNH5	0.111197874	0.011564	0.02025
KCNH6	0.034014538	0.441146	0.515439
KCNH7	0.040754228	0.356009	0.430515
KCNH8	-0.092236101	0.03639	0.057328
KCNIP1	-0.145488859	0.000929	0.001985
KCNIP2	-0.307305291	1.00E-12	8.78E-12
KCNIP3	-0.195588424	7.78E-06	2.37E-05
KCNIP4	-0.016883927	0.702273	0.757653
KCNJ10	-0.018218463	0.679993	0.737197
KCNJ11	-0.216674201	6.90E-07	2.48E-06
KCNJ12	-0.02777785	0.529369	0.601233

KCNJ13	-0.174294841	7.00E-05	0.000182
KCNJ14	0.058014259	0.188691	0.249145
KCNJ15	-0.322255411	6.57E-14	6.84E-13
KCNJ16	-0.091119656	0.038724	0.06063
KCNJ1	-0.101276102	0.021525	0.035648
KCNJ2	-0.15910257	0.000289	0.000678
KCNJ3	0.007395363	0.867038	0.895857
KCNJ4	0.055667043	0.207239	0.270349
KCNJ5	-0.32118142	8.03E-14	8.23E-13
KCNJ6	0.026703319	0.545425	0.616042
KCNJ8	0.054884133	0.213709	0.277685
KCNJ9	-0.045164581	0.306318	0.378083
KCNK10	0.023422548	0.595888	0.661884
KCNK12	0.106992836	0.015135	0.025882
KCNK13	-0.123379474	0.00505	0.009481
KCNK15	-0.099040714	0.024598	0.040211
KCNK16	-0.074981333	0.089158	0.128374
KCNK17	-0.279562299	1.06E-10	6.80E-10
KCNK18	0.041626615	0.345798	0.420043
KCNK1	-0.009220628	0.834647	0.868646
KCNK2	0.040820012	0.355232	0.429727
KCNK3	-0.189520708	1.49E-05	4.36E-05
KCNK4	-0.245112521	1.75E-08	8.07E-08
KCNK5	-0.342712247	1.22E-15	1.67E-14
KCNK6	-0.004543981	0.918067	0.935763
KCNK7	-0.034328581	0.436937	0.511329
KCNK9	0.134443547	0.002232	0.004464
KCNMA1	-0.118536232	0.007082	0.01293
KCNMB1	-0.210164075	1.50E-06	5.10E-06
KCNMB2	-0.154042494	0.000451	0.00102
KCNMB3	-0.069089393	0.11736	0.163939
KCNMB4	0.297088429	5.92E-12	4.58E-11
KCNN1	-0.063043538	0.15311	0.20747
KCNN2	-0.108466524	0.013786	0.023778
KCNN3	-0.053111994	0.22889	0.29467
KCNN4	-0.064150214	0.146013	0.199107
KCNQ1DN	-0.041179389	0.351009	0.425597
KCNQ1OT1	-0.194491763	8.77E-06	2.65E-05
KCNQ1	-0.337376227	3.56E-15	4.53E-14
KCNQ2	0.062162267	0.158947	0.214476
KCNQ3	-0.117358545	0.007675	0.013926
KCNQ4	-0.059587983	0.176958	0.235834
KCNQ5	-0.087786844	0.046458	0.071569
KCNRG	-0.232450835	9.52E-08	3.94E-07

KCNS1	-0.168448529	0.000123	0.000308
KCNS2	-0.195106658	8.20E-06	2.49E-05
KCNS3	-0.155036677	0.000414	0.000941
KCNT1	-0.062148606	0.159039	0.214586
KCNT2	-0.126309621	0.004092	0.0078
KCNU1	0.243105194	2.30E-08	1.04E-07
KCNV1	0.155375921	0.000402	0.000915
KCNV2	-0.007234753	0.8699	0.898442
KCP	0.08781688	0.046383	0.071464
KCTD10	-0.143467802	0.001095	0.002315
KCTD11	-0.060145582	0.172934	0.231071
KCTD12	-0.233772576	8.01E-08	3.35E-07
KCTD13	-0.082315996	0.061947	0.092797
KCTD14	-0.146046983	0.000887	0.001903
KCTD15	-0.036579878	0.40745	0.482214
KCTD16	-0.099486847	0.023956	0.039239
KCTD17	-0.075846587	0.085518	0.123734
KCTD18	-0.178721939	4.53E-05	0.000122
KCTD19	-0.295060298	8.35E-12	6.33E-11
KCTD1	-0.101242912	0.021568	0.035711
KCTD20	0.166715119	0.000144	0.000358
KCTD21	0.058574252	0.184452	0.244226
KCTD2	-0.276028575	1.85E-10	1.15E-09
KCTD3	0.260691659	1.90E-09	1.01E-08
KCTD4	0.000277416	0.994989	0.996535
KCTD5	0.164242791	0.000181	0.000442
KCTD6	0.186066361	2.14E-05	6.09E-05
KCTD7	-0.337117179	3.75E-15	4.75E-14
KCTD8	-0.099086011	0.024532	0.040114
KCTD9	0.103005379	0.019382	0.032383
KDELC1	0.147771937	0.000769	0.001669
KDELC2	0.137960888	0.0017	0.00347
KDELR1	0.16100322	0.000244	0.000579
KDELR2	0.237443798	4.94E-08	2.13E-07
KDELR3	0.125589647	0.004311	0.008185
KDM1A	0.282779131	6.34E-11	4.19E-10
KDM1B	0.102818775	0.019604	0.032732
KDM2A	-0.068717625	0.119351	0.166417
KDM2B	-0.025823546	0.558747	0.628206
KDM3A	0.017665549	0.689192	0.745673
KDM3B	-0.158465852	0.000306	0.000713
KDM4A	-0.212770389	1.10E-06	3.83E-06
KDM4B	-0.154917206	0.000418	0.00095
KDM4C	-0.221679201	3.74E-07	1.40E-06

KDM4DL	-0.033085569	0.45373	0.527828
KDM4D	-0.119075445	0.006824	0.012504
KDM5A	0.106857141	0.015265	0.026067
KDM5B	0.050946153	0.248462	0.316343
KDM5C	-0.200924703	4.32E-06	1.37E-05
KDM5D	0.139803303	0.00147	0.003034
KDM6A	-0.237129893	5.15E-08	2.21E-07
KDM6B	-0.233952486	7.83E-08	3.27E-07
KDR	-0.175706904	6.10E-05	0.00016
KDSR	0.012471017	0.777686	0.821097
KEAP1	-0.054284724	0.218761	0.283236
KEL	-0.232006107	1.01E-07	4.15E-07
KERA	-0.105316048	0.01681	0.028442
KGFLP1	-0.046177828	0.295583	0.366895
KGFLP2	-0.222481962	3.38E-07	1.28E-06
KHDC1L	0.271357273	3.83E-10	2.26E-09
KHDC1	-0.017225091	0.696552	0.752293
KHDRBS1	0.258029496	2.81E-09	1.45E-08
KHDRBS2	-0.2578243	2.90E-09	1.49E-08
KHDRBS3	-0.032055752	0.467913	0.541742
KHK	0.097906616	0.026297	0.042748
KHNYN	-0.023345169	0.597103	0.662829
KHSRP	0.131176857	0.002859	0.005601
KIAA0020	0.273184932	2.89E-10	1.73E-09
KIAA0040	-0.237781654	4.72E-08	2.04E-07
KIAA0087	-0.238324661	4.39E-08	1.91E-07
KIAA0090	0.239233092	3.89E-08	1.70E-07
KIAA0100	0.003078803	0.944433	0.955911
KIAA0101	0.530378916	1.06E-38	6.21E-36
KIAA0114	0.124175944	0.004771	0.009
KIAA0125	-0.127335384	0.003798	0.007277
KIAA0141	-0.309585786	6.68E-13	6.01E-12
KIAA0146	-0.035340113	0.423539	0.498165
KIAA0174	-0.051486864	0.24347	0.310858
KIAA0182	-0.100971377	0.021923	0.036239
KIAA0195	-0.246497684	1.44E-08	6.75E-08
KIAA0196	0.293472936	1.09E-11	8.11E-11
KIAA0226	-0.148706635	0.000711	0.001554
KIAA0232	-0.165327998	0.000164	0.000403
KIAA0240	-0.238130263	4.51E-08	1.95E-07
KIAA0247	-0.325605225	3.49E-14	3.80E-13
KIAA0284	-0.0973958	0.027095	0.04392
KIAA0317	0.189152517	1.55E-05	4.52E-05
KIAA0319L	-0.390184196	3.57E-20	9.58E-19

KIAA0319	0.06536368	0.138522	0.190072
KIAA0355	-0.242125588	2.63E-08	1.18E-07
KIAA0368	0.02308453	0.601205	0.666383
KIAA0391	0.293006551	1.18E-11	8.73E-11
KIAA0406	0.267512414	6.88E-10	3.92E-09
KIAA0408	-0.41092329	2.12E-22	7.79E-21
KIAA0415	-0.094516856	0.031991	0.051
KIAA0427	-0.284448295	4.84E-11	3.26E-10
KIAA0430	-0.331760339	1.07E-14	1.26E-13
KIAA0467	-0.41515381	7.11E-23	2.85E-21
KIAA0494	-0.394556296	1.25E-20	3.55E-19
KIAA0495	-0.408827558	3.61E-22	1.30E-20
KIAA0513	-0.355442298	8.80E-17	1.41E-15
KIAA0528	0.022868401	0.604616	0.669422
KIAA0556	-0.235301917	6.56E-08	2.77E-07
KIAA0562	-0.223656411	2.92E-07	1.12E-06
KIAA0564	0.014973636	0.73461	0.785166
KIAA0586	0.219137112	5.11E-07	1.89E-06
KIAA0649	-0.185891276	2.18E-05	6.19E-05
KIAA0652	0.035933619	0.415791	0.490546
KIAA0664P3	-0.352069809	1.79E-16	2.74E-15
KIAA0664	0.005482239	0.901228	0.922741
KIAA0748	-0.327690956	2.35E-14	2.62E-13
KIAA0753	-0.134456302	0.00223	0.00446
KIAA0754	-0.095359397	0.030486	0.048861
KIAA0776	-0.004016303	0.927554	0.943316
KIAA0802	-0.023783648	0.590231	0.656339
KIAA0831	0.013419801	0.761267	0.80774
KIAA0892	-0.370822654	3.11E-18	6.26E-17
KIAA0895L	-0.283819195	5.36E-11	3.58E-10
KIAA0895	0.06011661	0.173141	0.231318
KIAA0907	-0.164460619	0.000178	0.000434
KIAA0913	-0.323573332	5.13E-14	5.42E-13
KIAA0922	-0.13658243	0.001893	0.00383
KIAA0947	0.021419322	0.627707	0.690285
KIAA1009	-0.27302895	2.96E-10	1.77E-09
KIAA1012	0.035318711	0.42382	0.49843
KIAA1024	0.020944019	0.635363	0.696748
KIAA1033	0.184939314	2.41E-05	6.78E-05
KIAA1045	-0.146596305	0.000848	0.001825
KIAA1107	-0.088796295	0.043989	0.068091
KIAA1109	-0.33957964	2.30E-15	3.02E-14
KIAA1143	-0.058497072	0.185032	0.244848
KIAA1147	-0.146336433	0.000866	0.001862

KIAA1161	0.148935487	0.000697	0.001527
KIAA1191	-0.117353917	0.007678	0.013929
KIAA1199	0.109013272	0.013313	0.023016
KIAA1210	0.002249714	0.959381	0.968882
KIAA1211	0.027147155	0.538764	0.609578
KIAA1217	-0.165614973	0.00016	0.000393
KIAA1239	0.103279089	0.01906	0.031902
KIAA1244	-0.25282268	5.94E-09	2.94E-08
KIAA1257	-0.056803412	0.198101	0.259836
KIAA1267	-0.274401715	2.39E-10	1.45E-09
KIAA1274	-0.335288969	5.37E-15	6.64E-14
KIAA1279	0.179316904	4.26E-05	0.000115
KIAA1310	-0.214288093	9.19E-07	3.25E-06
KIAA1324L	-0.261959207	1.58E-09	8.50E-09
KIAA1324	-0.169511864	0.000111	0.00028
KIAA1328	-0.282086347	7.08E-11	4.66E-10
KIAA1370	-0.276526887	1.71E-10	1.06E-09
KIAA1377	-0.169448505	0.000112	0.000282
KIAA1383	-0.065241851	0.13926	0.190941
KIAA1407	-0.396245069	8.27E-21	2.44E-19
KIAA1409	-0.111102593	0.011636	0.02036
KIAA1429	0.338329702	2.94E-15	3.81E-14
KIAA1430	0.0319171	0.469841	0.543565
KIAA1432	-0.04222953	0.338851	0.412921
KIAA1462	-0.071881866	0.103228	0.146227
KIAA1467	-0.106966352	0.01516	0.025915
KIAA1468	-0.028102311	0.524569	0.596556
KIAA1486	-0.093962898	0.033016	0.052457
KIAA1522	-0.152120346	0.000532	0.001188
KIAA1524	0.457081939	5.98E-28	4.88E-26
KIAA1529	-0.437996382	1.50E-25	9.14E-24
KIAA1530	-0.181499699	3.42E-05	9.39E-05
KIAA1539	-0.070568759	0.109696	0.154397
KIAA1543	-0.301260007	2.89E-12	2.34E-11
KIAA1549	0.113802867	0.009746	0.017347
KIAA1586	0.115299734	0.00882	0.015804
KIAA1598	0.154527084	0.000432	0.000981
KIAA1609	0.24809322	1.16E-08	5.48E-08
KIAA1614	-0.229940671	1.32E-07	5.33E-07
KIAA1632	-0.187342955	1.88E-05	5.39E-05
KIAA1644	-0.022448243	0.611273	0.675482
KIAA1671	-0.307278047	1.01E-12	8.82E-12
KIAA1683	-0.407969437	4.49E-22	1.59E-20
KIAA1704	0.063494656	0.150186	0.20406

KIAA1712	0.016135761	0.714878	0.768011
KIAA1715	0.23755627	4.86E-08	2.10E-07
KIAA1731	-0.035879766	0.416491	0.491267
KIAA1737	-0.074155846	0.092745	0.132841
KIAA1751	-0.157420292	0.000336	0.000776
KIAA1755	-0.133124292	0.002468	0.004893
KIAA1797	0.003978106	0.928241	0.943749
KIAA1804	0.086157499	0.050688	0.077476
KIAA1826	-0.13720894	0.001803	0.003662
KIAA1841	0.015568915	0.72448	0.776414
KIAA1875	-0.170801684	9.81E-05	0.00025
KIAA1908	-0.326905886	2.73E-14	3.01E-13
KIAA1919	-0.016481774	0.709039	0.763304
KIAA1949	0.004905581	0.911572	0.930914
KIAA1958	-0.008762353	0.842756	0.87552
KIAA1967	-0.077390138	0.079323	0.115814
KIAA1984	-0.156529885	0.000363	0.000834
KIAA2013	0.029997417	0.49698	0.570113
KIAA2018	-0.235839527	6.11E-08	2.59E-07
KIAA2022	-0.422691687	9.79E-24	4.46E-22
KIAA2026	-0.20715143	2.13E-06	7.06E-06
KIDINS220	-0.280779743	8.73E-11	5.68E-10
KIF11	0.508612653	3.11E-35	9.58E-33
KIF12	-0.182000119	3.25E-05	8.95E-05
KIF13A	-0.283284524	5.84E-11	3.88E-10
KIF13B	-0.144104229	0.00104	0.002205
KIF14	0.499789493	6.76E-34	1.55E-31
KIF15	0.451525626	3.09E-27	2.36E-25
KIF16B	-0.282249175	6.90E-11	4.55E-10
KIF17	-0.230189876	1.28E-07	5.17E-07
KIF18A	0.472179744	5.88E-30	6.42E-28
KIF18B	0.399579009	3.66E-21	1.15E-19
KIF19	-0.137929372	0.001704	0.003478
KIF1A	0.022645305	0.608147	0.672697
KIF1B	-0.032821914	0.457338	0.531283
KIF1C	-0.228353574	1.61E-07	6.42E-07
KIF20A	0.496506246	2.08E-33	4.37E-31
KIF20B	0.428694583	1.95E-24	9.96E-23
KIF21A	0.206715402	2.24E-06	7.40E-06
KIF21B	-0.157633745	0.000329	0.000763
KIF22	0.112933806	0.010322	0.018272
KIF23	0.523721014	1.29E-37	6.16E-35
KIF24	0.232625682	9.31E-08	3.85E-07
KIF25	-0.106978106	0.015149	0.0259

KIF26A	-0.229911331	1.32E-07	5.35E-07
KIF26B	0.02986956	0.498817	0.571892
KIF27	-0.213835144	9.70E-07	3.42E-06
KIF2A	0.153031804	0.000492	0.001105
KIF2C	0.491127567	1.27E-32	2.25E-30
KIF3A	-0.150788664	0.000596	0.00132
KIF3B	0.079746294	0.070573	0.104267
KIF3C	0.200520704	4.52E-06	1.43E-05
KIF4A	0.504745721	1.21E-34	3.23E-32
KIF4B	0.423538038	7.81E-24	3.63E-22
KIF5A	-0.025337899	0.566169	0.634941
KIF5B	0.212177662	1.18E-06	4.09E-06
KIF5C	0.010295278	0.815703	0.853135
KIF6	-0.276999207	1.59E-10	9.93E-10
KIF7	-0.229949649	1.32E-07	5.33E-07
KIF9	-0.099449098	0.02401	0.039324
KIFAP3	-0.099232073	0.024321	0.039794
KIFC1	0.453454953	1.75E-27	1.36E-25
KIFC2	-0.063235985	0.151858	0.205981
KIFC3	-0.127366762	0.003789	0.007263
KILLIN	-0.18690217	1.96E-05	5.62E-05
KIN	0.213710613	9.84E-07	3.46E-06
KIR2DL1	0.054865349	0.213866	0.277853
KIR2DL3	0.097362094	0.027148	0.043988
KIR2DL4	0.206718561	2.23E-06	7.40E-06
KIR2DS4	0.066263602	0.13316	0.183483
KIR3DL1	0.061108101	0.166148	0.223003
KIR3DL2	0.059408591	0.178267	0.237263
KIR3DL3	0.113828972	0.009729	0.017321
KIR3DP1	0.116865701	0.007936	0.014365
KIR3DX1	0.137265964	0.001795	0.003646
KIRREL2	-0.110304692	0.012253	0.021337
KIRREL3	-0.114404303	0.009364	0.01671
KIRREL	-0.161114881	0.000241	0.000574
KISS1R	0.115178934	0.008892	0.015926
KISS1	-0.016338551	0.711454	0.765327
KITLG	-0.081074371	0.066	0.09822
KIT	-0.053991244	0.221265	0.286052
KLB	0.002100016	0.962082	0.970972
KLC1	-0.044563539	0.312806	0.384879
KLC2	0.125200758	0.004433	0.008401
KLC3	-0.002774821	0.949912	0.960878
KLC4	-0.275068268	2.15E-10	1.32E-09
KLF10	0.01750853	0.691812	0.747901

KLF11	-0.252398088	6.31E-09	3.10E-08
KLF12	-0.191323663	1.23E-05	3.64E-05
KLF13	-0.267150381	7.27E-10	4.13E-09
KLF14	0.059808604	0.175357	0.233935
KLF15	-0.341532658	1.55E-15	2.09E-14
KLF16	0.185253774	2.33E-05	6.58E-05
KLF17	0.000186481	0.996632	0.99768
KLF1	0.158439007	0.000307	0.000715
KLF2	-0.264679581	1.05E-09	5.84E-09
KLF3	0.021229902	0.630753	0.692568
KLF4	-0.057867822	0.189811	0.250409
KLF5	-0.003517145	0.936537	0.950132
KLF6	-0.083897274	0.057085	0.086146
KLF7	0.043346939	0.326213	0.399458
KLF8	-0.328313541	2.08E-14	2.34E-13
KLF9	-0.179624602	4.13E-05	0.000112
KLHDC10	-0.016414491	0.710173	0.764292
KLHDC1	-0.333622421	7.45E-15	8.98E-14
KLHDC2	0.088308981	0.045167	0.069757
KLHDC3	-0.044714806	0.311165	0.383152
KLHDC4	-0.076283264	0.083727	0.121359
KLHDC5	0.153893109	0.000457	0.001032
KLHDC7A	-0.395272678	1.05E-20	3.03E-19
KLHDC7B	0.027675709	0.530885	0.602403
KLHDC8A	-0.132512372	0.002585	0.005108
KLHDC8B	-0.289429796	2.14E-11	1.52E-10
KLHDC9	-0.172140262	8.62E-05	0.000221
KLHL10	-0.06073491	0.168755	0.226153
KLHL11	-0.040359533	0.36069	0.435434
KLHL12	0.0646856	0.142671	0.195135
KLHL13	-0.116536296	0.008115	0.014663
KLHL14	-0.184063458	2.64E-05	7.37E-05
KLHL15	0.065118451	0.140011	0.191807
KLHL17	-0.156104799	0.000377	0.000862
KLHL18	0.071060258	0.107238	0.151279
KLHL1	0.188730649	1.62E-05	4.71E-05
KLHL20	-0.206771417	2.22E-06	7.36E-06
KLHL21	-0.144244334	0.001028	0.002183
KLHL22	-0.201343973	4.12E-06	1.31E-05
KLHL23	0.123186183	0.00512	0.009604
KLHL24	-0.178718315	4.53E-05	0.000122
KLHL25	0.133064099	0.002479	0.004913
KLHL26	-0.276700768	1.67E-10	1.04E-09
KLHL28	0.170658743	9.94E-05	0.000253

KLHL29	-0.306001407	1.26E-12	1.09E-11
KLHL2	-0.016164292	0.714396	0.767687
KLHL30	-0.092220638	0.036421	0.057362
KLHL31	0.114244099	0.009464	0.016877
KLHL32	-0.196625415	6.95E-06	2.13E-05
KLHL33	-0.374398365	1.40E-18	2.98E-17
KLHL34	-0.146046617	0.000887	0.001903
KLHL35	-0.012027437	0.785397	0.827632
KLHL36	-0.032321171	0.464234	0.538138
KLHL38	-0.095850824	0.029636	0.047652
KLHL3	-0.222542627	3.36E-07	1.27E-06
KLHL4	-0.014711195	0.739091	0.788774
KLHL5	0.251045258	7.65E-09	3.73E-08
KLHL6	-0.243917894	2.06E-08	9.41E-08
KLHL7	0.169842556	0.000107	0.000272
KLHL8	0.058845646	0.182423	0.241844
KLHL9	-0.011263399	0.798728	0.838981
KLK10	0.030829699	0.485113	0.55884
KLK11	-0.087886652	0.046209	0.071218
KLK12	0.121862546	0.005621	0.010465
KLK13	0.064840753	0.141713	0.193931
KLK14	0.124185596	0.004768	0.008995
KLK15	0.074681127	0.09045	0.130028
KLK1	0.024902001	0.572869	0.641003
KLK2	0.111089275	0.011646	0.020374
KLK3	-0.042630234	0.334283	0.40804
KLK4	0.047936781	0.27755	0.348078
KLK5	0.032261934	0.465053	0.538869
KLK6	0.24105329	3.04E-08	1.35E-07
KLK7	0.011414487	0.796087	0.836691
KLK8	0.233124132	8.72E-08	3.63E-07
KLK9	0.170641292	9.96E-05	0.000253
KLKB1	-0.253839001	5.14E-09	2.56E-08
KLKP1	0.11804366	0.007325	0.013347
KLRA1	-0.196891742	6.75E-06	2.07E-05
KLRAQ1	-0.162792774	0.000207	0.0005
KLRB1	-0.198292876	5.78E-06	1.80E-05
KLRC1	0.129995306	0.003122	0.00608
KLRC2	0.180068169	3.95E-05	0.000107
KLRC3	0.169527589	0.000111	0.00028
KLRC4	0.022520165	0.610131	0.674432
KLRD1	0.114583497	0.009253	0.016532
KLRF1	-0.05721893	0.194834	0.256072
KLRG1	-0.171727844	8.97E-05	0.00023

KLRG2	-0.094452098	0.03211	0.051173
KLRK1	-0.094660873	0.031729	0.050652
KL	-0.25749591	3.04E-09	1.56E-08
KMO	-0.00149815	0.972944	0.979708
KNCN	-0.0534803	0.225673	0.290941
KNDC1	-0.250896744	7.81E-09	3.80E-08
KNG1	0.130585846	0.002988	0.005837
KNTC1	0.253580919	5.34E-09	2.65E-08
KPNA1	0.23205642	1.00E-07	4.12E-07
KPNA2	0.538140764	5.31E-40	4.42E-37
KPNA3	0.328916265	1.85E-14	2.11E-13
KPNA4	0.393205623	1.73E-20	4.82E-19
KPNA5	-0.127486184	0.003756	0.007204
KPNA6	-0.018543927	0.674601	0.732344
KPNA7	0.107520887	0.014639	0.025116
KPNB1	0.328427256	2.04E-14	2.29E-13
KPRP	0.031125546	0.480931	0.554758
KPTN	0.086492036	0.049795	0.076244
KRAS	0.209941757	1.54E-06	5.23E-06
KRBA1	-0.261365448	1.72E-09	9.20E-09
KRBA2	-0.173556722	7.52E-05	0.000195
KRCC1	-0.056894689	0.19738	0.25906
KREMEN1	-0.073831148	0.094188	0.134762
KREMEN2	0.158078454	0.000317	0.000736
KRI1	-0.080297378	0.068645	0.101718
KRIT1	-0.021366832	0.628551	0.690989
KRR1	0.39360463	1.57E-20	4.39E-19
KRT10	0.089884014	0.041454	0.064556
KRT12	0.028600401	0.517243	0.589432
KRT13	-0.131487829	0.002793	0.005484
KRT14	0.046018504	0.297254	0.368512
KRT15	-0.183635843	2.75E-05	7.68E-05
KRT16	0.11426043	0.009454	0.016861
KRT17	0.038451774	0.383857	0.458472
KRT18	0.321825932	7.12E-14	7.38E-13
KRT19	0.001896436	0.965755	0.973794
KRT1	-0.24574982	1.60E-08	7.43E-08
KRT20	-0.00603155	0.891389	0.915105
KRT222	-0.108663255	0.013614	0.023493
KRT23	-0.077136824	0.080314	0.117066
KRT24	-0.003442937	0.937873	0.950957
KRT25	0.099738645	0.0236	0.03872
KRT26	0.08851867	0.044657	0.06905
KRT27	-0.217748134	6.05E-07	2.20E-06

KRT28	0.05446516	0.217231	0.281551
KRT2	-0.105085394	0.017052	0.028821
KRT31	-0.047622363	0.280717	0.351389
KRT32	-0.184860567	2.43E-05	6.83E-05
KRT33A	-0.041908937	0.342534	0.416584
KRT33B	-0.081891709	0.063308	0.094595
KRT34	0.094326308	0.03234	0.051512
KRT35	-0.022123672	0.616436	0.679985
KRT36	-0.091864971	0.037152	0.058394
KRT37	0.010829546	0.806324	0.845026
KRT38	-0.024142173	0.584639	0.651308
KRT39	-0.161697571	0.000229	0.000548
KRT3	0.020304939	0.645717	0.706166
KRT40	-0.086255826	0.050424	0.077126
KRT4	-0.174820077	6.65E-05	0.000174
KRT5	-0.116460961	0.008157	0.014729
KRT6A	0.231248073	1.11E-07	4.55E-07
KRT6B	0.197763461	6.13E-06	1.90E-05
KRT6C	0.195617368	7.76E-06	2.36E-05
KRT71	-0.015746936	0.72146	0.773717
KRT72	-0.221592814	3.78E-07	1.42E-06
KRT73	-0.205932578	2.45E-06	8.05E-06
KRT74	-0.073530155	0.095541	0.136512
KRT75	-0.021310781	0.629452	0.691653
KRT76	0.005245462	0.905473	0.926091
KRT77	-0.054723401	0.215056	0.27918
KRT78	0.143465895	0.001096	0.002315
KRT79	-0.191640281	1.19E-05	3.53E-05
KRT7	0.137923337	0.001705	0.003479
KRT80	0.184019392	2.65E-05	7.41E-05
KRT81	0.115824441	0.008515	0.0153
KRT82	0.067940204	0.123599	0.171605
KRT83	0.210374675	1.46E-06	4.98E-06
KRT84	0.018544392	0.674593	0.732344
KRT85	0.000482679	0.991282	0.993568
KRT86	0.075284977	0.087867	0.126752
KRT8	0.240278544	3.38E-08	1.49E-07
KRT9	0.082520554	0.061299	0.091951
KRTAP1-1	0.022064823	0.617375	0.680897
KRTAP1-3	0.052216005	0.23685	0.303491
KRTAP1-5	0.008144837	0.853708	0.884394
KRTAP10-10	-0.044803943	0.3102	0.382191
KRTAP10-11	0.006632953	0.880637	0.906394
KRTAP10-12	0.000862867	0.984415	0.9883

KRTAP10-1	-0.038802082	0.379536	0.454262
KRTAP10-2	-0.071839501	0.103431	0.146457
KRTAP10-3	-0.140874921	0.00135	0.002803
KRTAP10-4	0.079638649	0.070955	0.104776
KRTAP10-5	-0.008574306	0.846088	0.878205
KRTAP10-6	0.019657355	0.65628	0.71572
KRTAP10-7	0.047345555	0.283526	0.354328
KRTAP10-9	0.034602548	0.433285	0.507947
KRTAP11-1	-0.046073257	0.296679	0.368027
KRTAP12-1	0.015256123	0.729797	0.781066
KRTAP12-2	-0.015669996	0.722765	0.774867
KRTAP12-4	-0.069102106	0.117293	0.163867
KRTAP13-1	-0.069121272	0.117191	0.16378
KRTAP13-2	0.009057655	0.837529	0.870904
KRTAP13-4	-0.092712164	0.035431	0.055967
KRTAP17-1	-0.01676202	0.704322	0.759343
KRTAP19-1	-0.031632199	0.473816	0.547562
KRTAP19-3	-0.05113507	0.24671	0.314408
KRTAP19-5	0.023678399	0.591877	0.657868
KRTAP19-8	0.005567917	0.899692	0.921688
KRTAP2-1	0.030682482	0.487201	0.560631
KRTAP2-2	0.007406077	0.866847	0.895706
KRTAP20-4	-0.020246691	0.646664	0.707047
KRTAP21-2	0.027162995	0.538527	0.609345
KRTAP26-1	-0.014702767	0.739235	0.788886
KRTAP3-1	-0.008441702	0.848439	0.880234
KRTAP3-2	0.009036238	0.837908	0.871115
KRTAP3-3	0.030724134	0.486609	0.560016
KRTAP4-11	-0.081600624	0.064256	0.095847
KRTAP4-12	-0.050625902	0.251453	0.319554
KRTAP4-1	0.159660977	0.000275	0.000647
KRTAP4-2	0.054052731	0.220739	0.285519
KRTAP4-3	-0.047989443	0.277022	0.347567
KRTAP4-4	0.082774191	0.060504	0.090854
KRTAP4-5	-0.028411197	0.520019	0.592191
KRTAP4-7	-0.039247721	0.374083	0.448999
KRTAP4-8	-0.053621437	0.22445	0.289607
KRTAP4-9	-0.061282394	0.164941	0.221562
KRTAP5-10	-0.257195665	3.17E-09	1.62E-08
KRTAP5-11	-0.110911176	0.011781	0.020575
KRTAP5-1	-0.10257558	0.019896	0.033168
KRTAP5-2	-0.136556364	0.001896	0.003837
KRTAP5-3	-0.036814454	0.404447	0.479199
KRTAP5-4	-0.007614948	0.863128	0.892417

KRTAP5-5	-0.00081884	0.98521	0.988871
KRTAP5-6	0.016318576	0.711791	0.765566
KRTAP5-7	-0.148670697	0.000713	0.001558
KRTAP5-8	-0.279158229	1.13E-10	7.21E-10
KRTAP5-9	-0.350489671	2.49E-16	3.73E-15
KRTAP6-3	0.068519826	0.120421	0.167757
KRTAP7-1	0.0131041	0.766719	0.812274
KRTAP8-1	0.014916387	0.735587	0.785958
KRTAP9-2	0.031776768	0.471797	0.545512
KRTAP9-4	0.053665735	0.224066	0.289206
KRTAP9-9	0.048481752	0.272118	0.342124
KRTCAP2	0.12307605	0.00516	0.009674
KRTCAP3	-0.001220301	0.977961	0.983719
KRTDAP	-0.05849182	0.185072	0.244884
KSR1	-0.046539484	0.291812	0.363072
KSR2	-0.2164967	7.05E-07	2.53E-06
KTELC1	-0.055542928	0.208255	0.271515
KTI12	0.113689634	0.009819	0.017467
KTN1	0.2456782	1.62E-08	7.50E-08
KYNU	0.265175404	9.78E-10	5.46E-09
KY	-0.122945992	0.005207	0.009752
L1CAM	0.011336053	0.797457	0.837911
L1TD1	-0.20844605	1.83E-06	6.16E-06
L2HGDH	0.468814587	1.68E-29	1.70E-27
L3MBTL2	-0.277611287	1.44E-10	9.08E-10
L3MBTL3	0.017684173	0.688881	0.745377
L3MBTL4	-0.242078614	2.65E-08	1.19E-07
L3MBTL	-0.304160935	1.74E-12	1.47E-11
LACE1	-0.044831981	0.309897	0.381935
LACRT	-0.05531957	0.210093	0.27359
LACTB2	0.132430319	0.002601	0.005137
LACTB	0.02816004	0.523717	0.595689
LAD1	-0.037779134	0.392237	0.467169
LAG3	-0.009527133	0.829234	0.864077
LAGE3	0.172176243	8.60E-05	0.000221
LAIR1	-0.162431023	0.000214	0.000516
LAIR2	0.049640698	0.260808	0.329605
LALBA	0.088562109	0.044552	0.068893
LAMA1	0.04058142	0.358054	0.432591
LAMA2	-0.324849944	4.03E-14	4.34E-13
LAMA3	0.05866894	0.183742	0.243399
LAMA4	-0.044047133	0.318452	0.391104
LAMA5	-0.10181999	0.02083	0.034597
LAMB1	0.071876318	0.103254	0.146237

LAMB2L	-0.316364683	1.96E-13	1.90E-12
LAMB2	-0.349977093	2.77E-16	4.13E-15
LAMB3	0.118974354	0.006872	0.012582
LAMB4	0.000573703	0.989638	0.992368
LAMC1	0.179278363	4.28E-05	0.000115
LAMC2	0.198973831	5.36E-06	1.67E-05
LAMC3	-0.172321757	8.48E-05	0.000218
LAMP1	-0.134593148	0.002207	0.004415
LAMP2	-0.01160164	0.792819	0.833957
LAMP3	-0.330877023	1.27E-14	1.47E-13
LANCL1	0.025780149	0.559409	0.628772
LANCL2	0.074880268	0.089591	0.128905
LANCL3	-0.271985258	3.47E-10	2.06E-09
LAP3	0.016189072	0.713977	0.767422
LAPTM4A	-0.097529316	0.026884	0.043628
LAPTM4B	0.211565952	1.27E-06	4.38E-06
LAPTM5	-0.140349792	0.001407	0.002916
LARGE	-0.268448897	5.97E-10	3.43E-09
LARP1B	0.262709613	1.41E-09	7.65E-09
LARP1	0.110901062	0.011789	0.020587
LARP4B	-0.063242224	0.151817	0.20594
LARP4	0.393690272	1.54E-20	4.30E-19
LARP6	0.025458041	0.564328	0.633234
LARP7	-0.027473281	0.533896	0.60516
LARS2	0.159345846	0.000283	0.000665
LARS	0.04703256	0.286724	0.357654
LAS1L	0.044665783	0.311696	0.383703
LASP1	-0.006005526	0.891855	0.915489
LASS1	0.058011053	0.188716	0.249161
LASS2	0.005386343	0.902947	0.924075
LASS3	-0.045096925	0.307044	0.378886
LASS4	-0.284911562	4.49E-11	3.04E-10
LASS5	-0.001106663	0.980013	0.985237
LASS6	0.072742313	0.099156	0.141223
LAT2	-0.110521192	0.012083	0.021065
LATS1	0.005687831	0.897544	0.919913
LATS2	-0.094054649	0.032844	0.052226
LAT	-0.203423048	3.26E-06	1.05E-05
LAX1	-0.076166349	0.084204	0.122006
LAYN	-0.002574707	0.95352	0.964215
LBH	-0.256075876	3.73E-09	1.89E-08
LBP	0.197859604	6.07E-06	1.88E-05
LBR	0.306445747	1.17E-12	1.01E-11
LBX1	0.021180477	0.631549	0.693327

LBX2	-0.020086908	0.649266	0.70927
LBXCOR1	-0.315189343	2.43E-13	2.33E-12
LCA5L	-0.261924493	1.59E-09	8.53E-09
LCA5	-0.375598507	1.06E-18	2.30E-17
LCAT	-0.426219379	3.81E-24	1.88E-22
LCE1B	-0.042190979	0.339292	0.413397
LCE1C	-0.038377326	0.384779	0.459429
LCE1E	-0.006732374	0.878862	0.905219
LCE1F	-0.053142922	0.228618	0.294359
LCE2A	-0.013314157	0.76309	0.809416
LCE2B	-0.008758898	0.842817	0.875538
LCE2C	-0.003924244	0.92921	0.944616
LCE2D	0.046907067	0.288014	0.35897
LCE3A	0.072262584	0.10141	0.144026
LCE3D	0.120790834	0.006059	0.011198
LCE3E	0.095846769	0.029643	0.047659
LCE5A	0.05509403	0.211961	0.275627
LCK	-0.119670674	0.006549	0.012034
LCLAT1	0.30372502	1.88E-12	1.58E-11
LCMT1	0.066427726	0.132199	0.182285
LCMT2	-0.098338681	0.025638	0.041765
LCN10	-0.215540167	7.91E-07	2.82E-06
LCN12	-0.181002611	3.60E-05	9.84E-05
LCN15	0.146961851	0.000822	0.001776
LCN1	0.194552107	8.71E-06	2.63E-05
LCN2	0.067886372	0.123897	0.171964
LCN6	-0.208913368	1.73E-06	5.85E-06
LCN8	-0.022513089	0.610243	0.674493
LCN9	0.006434534	0.884182	0.909247
LCNL1	-0.304950217	1.52E-12	1.29E-11
LCORL	0.050927337	0.248637	0.316525
LCOR	0.035761437	0.41803	0.492723
LCP1	-0.176525289	5.63E-05	0.000149
LCP2	-0.146591937	0.000848	0.001826
LCTL	0.169395455	0.000112	0.000283
LCT	-0.173672618	7.44E-05	0.000193
LDB1	-0.385348612	1.12E-19	2.81E-18
LDB2	-0.257461595	3.05E-09	1.56E-08
LDB3	-0.334388117	6.41E-15	7.85E-14
LDHAL6A	-0.023095692	0.601029	0.666225
LDHAL6B	0.056385686	0.201425	0.263781
LDHA	0.441452813	5.64E-26	3.64E-24
LDHB	0.254322102	4.80E-09	2.40E-08
LDHC	0.057299636	0.194204	0.255345

LDHD	-0.208929642	1.73E-06	5.84E-06
LDLRAD1	-0.038610214	0.381899	0.456516
LDLRAD2	-0.283845984	5.34E-11	3.57E-10
LDLRAD3	0.080147353	0.069165	0.102429
LDLRAP1	-0.408159242	4.28E-22	1.53E-20
LDLR	-0.102798383	0.019628	0.032768
LDOC1L	-0.028422134	0.519858	0.59211
LDOC1	-0.173598291	7.49E-05	0.000194
LEAP2	-0.120506837	0.00618	0.011406
LECT1	-0.112005808	0.01097	0.01931
LECT2	0.016166208	0.714364	0.767687
LEF1	-0.052233604	0.236692	0.303366
LEFTY1	-0.051441629	0.243885	0.311307
LEFTY2	-0.398580174	4.67E-21	1.43E-19
LEKR1	-0.180471408	3.80E-05	0.000103
LELP1	0.004518127	0.918531	0.936141
LEMD1	-0.161205626	0.000239	0.00057
LEMD2	-0.032515952	0.461544	0.535424
LEMD3	0.144135668	0.001038	0.0022
LENEP	-0.003668585	0.933811	0.948232
LENG1	-0.179354906	4.25E-05	0.000115
LENG8	-0.367564658	6.42E-18	1.24E-16
LENG9	0.027261133	0.53706	0.607902
LEO1	0.236010204	5.97E-08	2.54E-07
LEPRE1	0.061857948	0.161001	0.216882
LEPREL1	-0.235848486	6.10E-08	2.59E-07
LEPREL2	-0.033861448	0.443206	0.517393
LEPROTL1	0.059100866	0.18053	0.239764
LEPROT	0.094478114	0.032062	0.051101
LEPR	-0.040286616	0.361559	0.436272
LEP	0.156699617	0.000358	0.000823
LETM1	0.212474086	1.14E-06	3.96E-06
LETM2	0.101611755	0.021094	0.034995
LETMD1	-0.08149944	0.064588	0.096278
LEUTX	0.027034402	0.540452	0.611281
LFNG	-0.074987954	0.08913	0.128352
LGALS12	-0.121257722	0.005865	0.010872
LGALS13	0.035635456	0.419673	0.494309
LGALS14	0.094317002	0.032358	0.051535
LGALS1	0.146334478	0.000866	0.001862
LGALS2	-0.241497497	2.87E-08	1.28E-07
LGALS3BP	-0.165687236	0.000159	0.000391
LGALS3	-0.037937247	0.390258	0.464978
LGALS4	-0.162951606	0.000204	0.000493

LGALS7B	0.124344757	0.004714	0.0089
LGALS7	0.084556322	0.055154	0.08358
LGALS8	-0.080635032	0.067485	0.100197
LGALS9B	-0.149383625	0.000672	0.001474
LGALS9C	-0.287015012	3.19E-11	2.21E-10
LGALS9	-0.319514912	1.09E-13	1.11E-12
LGI1	-0.019453614	0.659617	0.718754
LGI2	0.079199539	0.07253	0.106842
LGI3	-0.309066642	7.33E-13	6.56E-12
LGI4	-0.279200163	1.12E-10	7.17E-10
LGMN	-0.126893595	0.003922	0.007501
LGR4	0.162801646	0.000207	0.0005
LGR5	-0.106580021	0.015533	0.026478
LGR6	-0.298607077	4.57E-12	3.57E-11
LGSN	0.129505731	0.003238	0.006284
LGTN	0.047483289	0.282126	0.352821
LHB	-0.053696068	0.223804	0.288923
LHCGR	-0.180445629	3.81E-05	0.000104
LHFPL1	-0.15689859	0.000351	0.00081
LHFPL2	-0.022341991	0.612961	0.676898
LHFPL3	-0.323254036	5.44E-14	5.72E-13
LHFPL4	-0.150348852	0.000619	0.001366
LHFPL5	0.174108738	7.13E-05	0.000186
LHFP	-0.311224393	4.98E-13	4.55E-12
LHPP	-0.079176187	0.072614	0.106942
LHX1	0.205772829	2.49E-06	8.20E-06
LHX2	0.102131611	0.02044	0.033993
LHX3	-0.029077045	0.510282	0.582596
LHX4	-0.178781743	4.50E-05	0.000121
LHX5	0.116358906	0.008213	0.014821
LHX6	-0.17311866	7.85E-05	0.000203
LHX8	0.028426851	0.519789	0.592064
LHX9	-0.230509932	1.22E-07	4.98E-07
LIAS	0.071631912	0.104435	0.147679
LIFR	-0.232837343	9.06E-08	3.76E-07
LIF	0.014940365	0.735178	0.785647
LIG1	0.03578286	0.417751	0.492452
LIG3	0.214911804	8.53E-07	3.03E-06
LIG4	-0.12413474	0.004785	0.009024
LILRA1	-0.156133674	0.000376	0.00086
LILRA2	-0.147099827	0.000813	0.001757
LILRA3	0.123627709	0.004961	0.009327
LILRA4	-0.216064222	7.42E-07	2.66E-06
LILRA5	0.093886769	0.033158	0.052664

LILRA6	-0.088668355	0.044296	0.068534
LILRB1	-0.128295023	0.00354	0.006821
LILRB2	-0.025057781	0.57047	0.63862
LILRB3	-0.084465429	0.055417	0.083934
LILRB4	-0.046373277	0.293541	0.364836
LILRB5	-0.165080603	0.000168	0.000412
LILRP2	0.181233863	3.52E-05	9.64E-05
LIM2	0.015942015	0.718155	0.770917
LIMA1	-0.079227573	0.072428	0.106715
LIMCH1	-0.203956989	3.07E-06	9.93E-06
LIMD1	-0.292308287	1.33E-11	9.75E-11
LIMD2	-0.137589619	0.00175	0.003561
LIME1	-0.123962215	0.004845	0.009129
LIMK1	0.037385638	0.39719	0.472028
LIMK2	-0.168137895	0.000126	0.000316
LIMS1	0.114557149	0.009269	0.016558
LIMS2	-0.408076164	4.37E-22	1.56E-20
LIMS3-LOC440895	-0.05410584	0.220285	0.284969
LIMS3	0.100483324	0.022574	0.037198
LIN28A	0.182024208	3.25E-05	8.93E-05
LIN28B	0.25023191	8.58E-09	4.15E-08
LIN37	0.0344994	0.434658	0.509288
LIN52	0.159111247	0.000289	0.000677
LIN54	0.300411306	3.35E-12	2.68E-11
LIN7A	0.012928613	0.769754	0.814842
LIN7B	-0.082807353	0.060401	0.090712
LIN7C	0.146829461	0.000831	0.001793
LIN9	0.417217731	4.15E-23	1.73E-21
LINGO1	0.035188677	0.42553	0.500176
LINGO2	0.159821468	0.000271	0.000639
LINGO3	-0.19898014	5.36E-06	1.67E-05
LINGO4	-0.185789718	2.21E-05	6.25E-05
LINS1	-0.113611464	0.00987	0.017553
LIPA	-0.211046553	1.35E-06	4.64E-06
LIPC	0.087220976	0.047893	0.073495
LIPE	-0.201928981	3.86E-06	1.23E-05
LIPF	-0.147690487	0.000774	0.00168
LIPG	0.032789767	0.457779	0.531703
LIPH	-0.202741853	3.52E-06	1.13E-05
LIPI	0.046928913	0.287789	0.358713
LIPJ	-0.131630633	0.002763	0.005429
LIPK	0.121994383	0.005569	0.010375
LIPM	-0.059509423	0.177531	0.236471
LIPN	-0.126454635	0.004049	0.007725

LIPT1	-0.100165387	0.023007	0.037862
LIPT2	0.115063358	0.008961	0.016039
LITAF	-0.212733882	1.11E-06	3.85E-06
LIX1L	-0.182343957	3.14E-05	8.67E-05
LIX1	0.000635607	0.988519	0.991496
LLGL1	-0.025808566	0.558976	0.628356
LLGL2	-0.20665651	2.25E-06	7.45E-06
LLPH	0.373774292	1.61E-18	3.37E-17
LMAN1L	-0.164676639	0.000174	0.000426
LMAN1	0.27698791	1.59E-10	9.94E-10
LMAN2L	-0.012625091	0.775013	0.819167
LMAN2	-0.013573824	0.758611	0.80535
LMBR1L	-0.077010102	0.080814	0.117708
LMBR1	0.243668942	2.13E-08	9.71E-08
LMBRD1	-0.170201801	0.000104	0.000264
LMBRD2	-0.044257039	0.316149	0.388538
LMCD1	-0.124011691	0.004828	0.009099
LMF1	-0.383671736	1.66E-19	4.06E-18
LMF2	-0.278380093	1.28E-10	8.11E-10
LMLN	-0.142592294	0.001176	0.00247
LMNA	-0.103313502	0.01902	0.031848
LMNB1	0.383317539	1.80E-19	4.38E-18
LMNB2	0.324556604	4.26E-14	4.58E-13
LMO1	0.148689909	0.000712	0.001556
LMO2	-0.305442122	1.39E-12	1.19E-11
LMO3	-0.309232806	7.11E-13	6.38E-12
LMO4	0.005146935	0.907241	0.927519
LMO7	-0.18234798	3.14E-05	8.67E-05
LMOD1	-0.316629732	1.86E-13	1.82E-12
LMOD2	-0.038589168	0.382159	0.456772
LMOD3	-0.31098366	5.20E-13	4.75E-12
LMTK2	0.006962519	0.874754	0.902197
LMTK3	-0.167658979	0.000132	0.000329
LMX1A	0.095195902	0.030773	0.049259
LMX1B	-0.092466019	0.035924	0.056673
LNP1	-0.02546456	0.564229	0.633193
LNPEP	0.04669639	0.290187	0.361319
LNX1	-0.123884279	0.004872	0.009175
LNX2	-0.14849275	0.000724	0.00158
LOC100009676	-0.189037521	1.57E-05	4.57E-05
LOC100101266	-0.216907559	6.70E-07	2.42E-06
LOC100101938	-0.034887093	0.42951	0.504142
LOC100124692	-0.10047896	0.02258	0.037202
LOC100125556	0.121585293	0.005731	0.010647

LOC100126784	0.002086348	0.962329	0.971098
LOC100127888	0.043770095	0.321508	0.394397
LOC100128023	-0.038698555	0.38081	0.45535
LOC100128076	-0.109972793	0.012518	0.02175
LOC100128164	-0.198797299	5.47E-06	1.71E-05
LOC100128191	0.289707585	2.04E-11	1.46E-10
LOC100128239	-0.30674165	1.11E-12	9.64E-12
LOC100128288	-0.283560516	5.59E-11	3.72E-10
LOC100128292	-0.053969777	0.221449	0.28622
LOC100128542	-0.024061346	0.585897	0.652455
LOC100128554	-0.160524731	0.000254	0.000603
LOC100128573	-0.189363254	1.52E-05	4.43E-05
LOC100128640	-0.095040959	0.031047	0.049658
LOC100128675	-0.205556067	2.55E-06	8.38E-06
LOC100128788	-0.1407661	0.001362	0.002827
LOC100128811	-0.021366544	0.628555	0.690989
LOC100128822	-0.072097021	0.102197	0.144935
LOC100128842	-0.277467541	1.48E-10	9.28E-10
LOC100128977	-0.012878652	0.770619	0.815455
LOC100129034	-0.284825011	4.55E-11	3.08E-10
LOC100129055	-0.067989737	0.123325	0.17136
LOC100129066	0.027313933	0.536272	0.60724
LOC100129387	-0.217854639	5.98E-07	2.18E-06
LOC100129534	-0.321731939	7.24E-14	7.50E-13
LOC100129550	-0.346117815	6.12E-16	8.73E-15
LOC100129637	-0.135526609	0.002054	0.004136
LOC100129716	-0.206838247	2.20E-06	7.31E-06
LOC100129726	-0.245531168	1.65E-08	7.64E-08
LOC100129935	0.016075454	0.715897	0.768824
LOC100130015	-0.205215297	2.66E-06	8.69E-06
LOC100130093	-0.250254163	8.55E-09	4.14E-08
LOC100130148	-0.01680482	0.703602	0.7588
LOC100130238	0.033449317	0.448778	0.523043
LOC100130264	0.02388402	0.588663	0.654914
LOC100130274	0.065886517	0.135387	0.186253
LOC100130331	0.125253071	0.004417	0.008371
LOC100130386	-0.053918955	0.221885	0.286723
LOC100130522	-0.14344389	0.001098	0.002318
LOC100130557	-0.343892861	9.63E-16	1.33E-14
LOC100130581	-0.182268167	3.17E-05	8.73E-05
LOC100130691	-0.094054782	0.032844	0.052226
LOC100130776	-0.057555903	0.192214	0.253127
LOC100130872	-0.115040563	0.008975	0.016059
LOC100130932	0.206257513	2.36E-06	7.78E-06

LOC100130933	-0.174225955	7.05E-05	0.000184
LOC100130987	-0.147760792	0.000769	0.00167
LOC100131193	-0.247085113	1.33E-08	6.24E-08
LOC100131434	-0.393904012	1.46E-20	4.10E-19
LOC100131496	-0.021157658	0.631917	0.693693
LOC100131551	0.257686471	2.95E-09	1.52E-08
LOC100131691	-0.225966497	2.19E-07	8.53E-07
LOC100131726	0.308024886	8.82E-13	7.79E-12
LOC100132111	0.159903184	0.000269	0.000635
LOC100132215	-0.131834132	0.002721	0.005357
LOC100132247	-0.286481423	3.48E-11	2.39E-10
LOC100132287	-0.266923773	7.52E-10	4.26E-09
LOC100132288	-0.107230398	0.01491	0.025549
LOC100132354	0.154538976	0.000432	0.000981
LOC100132707	-0.381614539	2.68E-19	6.39E-18
LOC100132724	0.139957362	0.001452	0.002999
LOC100132831	0.122694268	0.005301	0.009912
LOC100132832	-0.301236239	2.90E-12	2.35E-11
LOC100133050	-0.125318382	0.004396	0.008337
LOC100133161	-0.246049474	1.54E-08	7.14E-08
LOC100133308	0.011915675	0.787343	0.829158
LOC100133331	-0.318440752	1.34E-13	1.33E-12
LOC100133469	0.111856172	0.011078	0.01948
LOC100133545	-0.111248375	0.011526	0.020193
LOC100133612	-0.035877139	0.416525	0.491267
LOC100133669	-0.110051654	0.012455	0.021652
LOC100133893	-0.059228272	0.179591	0.238754
LOC100133920	0.039680795	0.36883	0.44352
LOC100133957	0.038837709	0.379099	0.45382
LOC100133985	0.177188505	5.27E-05	0.00014
LOC100133991	-0.045052941	0.307516	0.379408
LOC100134229	0.007122543	0.8719	0.900183
LOC100134259	-0.032597173	0.460426	0.534362
LOC100134368	-0.146051662	0.000887	0.001902
LOC100134713	-0.080084383	0.069385	0.102723
LOC100134868	-0.073131812	0.097355	0.138886
LOC100144603	0.079968272	0.069791	0.103248
LOC100144604	-0.2676893	6.70E-10	3.82E-09
LOC100170939	-0.298766838	4.44E-12	3.48E-11
LOC100188947	0.041214349	0.3506	0.425172
LOC100188949	-0.227590811	1.78E-07	7.04E-07
LOC100189589	-0.182672131	3.04E-05	8.41E-05
LOC100190938	-0.15413866	0.000447	0.001012
LOC100190939	-0.164806157	0.000172	0.000422

LOC100190940	0.205597154	2.54E-06	8.35E-06
LOC100190986	-0.316666189	1.85E-13	1.81E-12
LOC100192378	-0.010277096	0.816022	0.853314
LOC100192379	-0.040889405	0.354414	0.429023
LOC100192426	-0.044115	0.317706	0.390236
LOC100216001	0.186153516	2.12E-05	6.05E-05
LOC100216545	-0.118604442	0.007049	0.012873
LOC100233209	-0.121677337	0.005695	0.010585
LOC100240726	-0.225134179	2.43E-07	9.40E-07
LOC100240734	0.061126257	0.166022	0.222894
LOC100240735	-0.00705031	0.873188	0.901074
LOC100268168	-0.010279316	0.815983	0.853314
LOC100270710	0.07580386	0.085695	0.123948
LOC100270746	-0.140478419	0.001393	0.002887
LOC100270804	-0.323969521	4.76E-14	5.06E-13
LOC100271722	-0.283780687	5.39E-11	3.60E-10
LOC100271831	-0.029956001	0.497575	0.570631
LOC100271832	-0.047083815	0.286199	0.357199
LOC100271836	-0.162334096	0.000216	0.00052
LOC100272146	-0.142960458	0.001141	0.002404
LOC100272216	-0.269451444	5.12E-10	2.97E-09
LOC100272217	-0.170728527	9.87E-05	0.000252
LOC100272228	-0.34370318	1.00E-15	1.38E-14
LOC100286793	0.107662981	0.014508	0.024902
LOC100286844	-0.14355349	0.001088	0.002299
LOC100287227	-0.054353349	0.218178	0.282591
LOC100287704	-0.014041011	0.750574	0.798684
LOC100287718	-0.205609467	2.54E-06	8.34E-06
LOC100288778	-0.226090643	2.15E-07	8.40E-07
LOC100289341	-0.01635767	0.711131	0.765109
LOC100302401	0.18380142	2.71E-05	7.56E-05
LOC100302640	-0.161264407	0.000238	0.000567
LOC100302650	-0.26605463	8.57E-10	4.81E-09
LOC100303728	-0.316040864	2.08E-13	2.01E-12
LOC113230	-0.210919789	1.37E-06	4.70E-06
LOC115110	-0.450891302	3.72E-27	2.81E-25
LOC116437	0.14126519	0.001308	0.002725
LOC121838	-0.208502055	1.82E-06	6.12E-06
LOC121952	-0.35299713	1.47E-16	2.28E-15
LOC126536	-0.050340779	0.254136	0.322641
LOC127841	0.163641121	0.000192	0.000465
LOC134466	-0.105133911	0.017001	0.028742
LOC143188	-0.201974369	3.84E-06	1.22E-05
LOC143666	-0.172484197	8.34E-05	0.000215

LOC144438	0.212082258	1.19E-06	4.13E-06
LOC144486	-0.003476011	0.937278	0.950691
LOC144571	-0.237186452	5.11E-08	2.19E-07
LOC144742	0.005056694	0.90886	0.928654
LOC144776	0.102104141	0.020474	0.034047
LOC145474	-0.020582089	0.641218	0.702168
LOC145783	0.153556516	0.00047	0.001059
LOC145820	-0.21464783	8.80E-07	3.12E-06
LOC145837	-0.025762794	0.559673	0.628999
LOC145845	-0.059074692	0.180724	0.239985
LOC146336	-0.018800326	0.670364	0.728299
LOC146481	0.025564529	0.5627	0.631938
LOC146880	-0.399474112	3.75E-21	1.17E-19
LOC147727	-0.269939027	4.76E-10	2.77E-09
LOC147804	0.110502172	0.012097	0.021087
LOC148145	-0.247202469	1.31E-08	6.15E-08
LOC148189	0.026702848	0.545432	0.616042
LOC148413	-0.150638129	0.000604	0.001336
LOC148696	-0.388512476	5.31E-20	1.38E-18
LOC148709	0.095482966	0.03027	0.048566
LOC148824	-0.012010343	0.785695	0.827857
LOC149134	-0.091203062	0.038545	0.060393
LOC149620	-0.386645808	8.26E-20	2.09E-18
LOC149837	-0.081580804	0.064321	0.095922
LOC150185	-0.016467743	0.709275	0.763517
LOC150197	-0.148967359	0.000696	0.001523
LOC150381	-0.003652453	0.934101	0.948388
LOC150527	0.003460412	0.937559	0.950782
LOC150568	0.009511286	0.829513	0.864323
LOC150622	-0.20613332	2.39E-06	7.88E-06
LOC150776	-0.247381656	1.28E-08	6.01E-08
LOC150786	0.291735168	1.46E-11	1.07E-10
LOC151009	-0.246062125	1.53E-08	7.13E-08
LOC151162	-0.098149809	0.025924	0.042193
LOC151174	-0.121979469	0.005575	0.010385
LOC151534	-0.077292882	0.079703	0.116285
LOC151658	0.039743542	0.368073	0.442715
LOC152024	0.028711208	0.51562	0.58787
LOC152217	0.182202125	3.19E-05	8.78E-05
LOC152225	0.231080674	1.14E-07	4.65E-07
LOC153328	-0.065120349	0.14	0.191807
LOC153684	-0.356545243	6.97E-17	1.14E-15
LOC153910	0.03552166	0.42116	0.495769
LOC154449	-0.007968459	0.856841	0.887156

LOC154761	0.024629356	0.57708	0.644683
LOC154822	-0.060180101	0.172687	0.230772
LOC157381	-0.183871816	2.69E-05	7.51E-05
LOC157627	-0.005691844	0.897472	0.919913
LOC158376	-0.167515432	0.000134	0.000333
LOC158572	-0.245772082	1.60E-08	7.41E-08
LOC158696	-0.229858723	1.33E-07	5.38E-07
LOC162632	-0.133902986	0.002326	0.004635
LOC168474	-0.211250194	1.32E-06	4.53E-06
LOC200030	-0.29526324	8.07E-12	6.13E-11
LOC200726	-0.010121632	0.818757	0.85539
LOC201651	-0.024549447	0.578316	0.645632
LOC202181	-0.4368295	2.07E-25	1.23E-23
LOC202781	-0.15607944	0.000378	0.000864
LOC219347	-0.061102906	0.166184	0.223037
LOC220429	0.207528721	2.03E-06	6.79E-06
LOC220594	-0.116223085	0.008289	0.014941
LOC220729	-0.067851074	0.124093	0.172213
LOC220930	-0.005489784	0.901093	0.922671
LOC221122	-0.09204077	0.036789	0.057875
LOC221442	-0.283704673	5.46E-11	3.64E-10
LOC221710	0.374959265	1.23E-18	2.64E-17
LOC222699	0.117398102	0.007655	0.013891
LOC253039	-0.265079657	9.92E-10	5.53E-09
LOC253724	-0.023395854	0.596307	0.662202
LOC254312	-0.076813151	0.081595	0.118647
LOC254559	-0.047798236	0.278943	0.349452
LOC255025	0.02348887	0.594847	0.660838
LOC255167	-0.185339049	2.31E-05	6.52E-05
LOC256880	-0.1470251	0.000818	0.001767
LOC257358	-0.120809232	0.006051	0.011186
LOC25845	-0.031258602	0.479057	0.552924
LOC26102	-0.157710206	0.000327	0.000758
LOC282997	-0.147309541	0.000799	0.001729
LOC283050	-0.288471905	2.51E-11	1.76E-10
LOC283070	-0.414257762	8.97E-23	3.53E-21
LOC283174	-0.419443529	2.31E-23	1.00E-21
LOC283267	-0.097016394	0.027701	0.044807
LOC283314	-0.234559495	7.23E-08	3.04E-07
LOC283332	-0.033165963	0.452633	0.52692
LOC283392	-0.038259734	0.386238	0.461014
LOC283404	0.206141736	2.39E-06	7.88E-06
LOC283663	-0.306621279	1.13E-12	9.83E-12
LOC283731	-0.216267814	7.24E-07	2.60E-06

LOC283761	-0.062918696	0.153927	0.208474
LOC283856	-0.253407748	5.47E-09	2.71E-08
LOC283867	-0.105602849	0.016512	0.027987
LOC283914	-0.077684375	0.078185	0.114278
LOC283922	-0.335391655	5.27E-15	6.52E-14
LOC283999	-0.051392348	0.244338	0.311746
LOC284009	-0.139426964	0.001514	0.00312
LOC284023	-0.205430079	2.59E-06	8.49E-06
LOC284100	-0.053579889	0.224809	0.290052
LOC284232	0.038171263	0.387339	0.462134
LOC284233	-0.254022145	5.01E-09	2.50E-08
LOC284276	-0.271354231	3.83E-10	2.26E-09
LOC284379	-0.008376616	0.849594	0.881158
LOC284440	-0.464862236	5.68E-29	5.33E-27
LOC284441	0.10703857	0.015092	0.025822
LOC284551	-0.052463165	0.234635	0.301097
LOC284578	-0.328151003	2.15E-14	2.41E-13
LOC284632	-0.105920649	0.016188	0.027484
LOC284661	-0.063522667	0.150006	0.203857
LOC284688	0.101165754	0.021668	0.035859
LOC284749	-0.245561888	1.64E-08	7.61E-08
LOC284788	-0.003435065	0.938015	0.951052
LOC284798	-0.078296188	0.07586	0.111328
LOC284837	-0.401108138	2.51E-21	8.05E-20
LOC284900	-0.340738588	1.82E-15	2.43E-14
LOC285033	-0.051602202	0.242414	0.309668
LOC285045	-0.059598226	0.176884	0.235782
LOC285074	-0.035687053	0.419	0.493691
LOC285194	-0.024290103	0.582339	0.649288
LOC285205	-0.052246418	0.236576	0.303238
LOC285359	-0.164564675	0.000176	0.000431
LOC285370	-0.016840976	0.702995	0.758227
LOC285375	-0.003857354	0.930413	0.945599
LOC285401	0.047597536	0.280968	0.351615
LOC285419	-0.23531979	6.54E-08	2.77E-07
LOC285456	-0.128958859	0.003371	0.006521
LOC285501	0.111281071	0.011501	0.020159
LOC285548	0.080336005	0.068511	0.10155
LOC285593	-0.317710628	1.53E-13	1.51E-12
LOC285627	-0.032402749	0.463106	0.537042
LOC285629	-0.138958413	0.001571	0.003224
LOC285692	-0.028890804	0.512996	0.585294
LOC285696	0.163926142	0.000187	0.000454
LOC285733	0.060190166	0.172615	0.230692

LOC285735	-0.098773219	0.02499	0.040795
LOC285740	-0.076583543	0.082514	0.11986
LOC285768	-0.19431866	8.93E-06	2.69E-05
LOC285780	-0.124508164	0.004659	0.008803
LOC285796	-0.174037254	7.18E-05	0.000187
LOC285830	-0.229910647	1.32E-07	5.35E-07
LOC285847	-0.101994331	0.020611	0.03426
LOC285954	0.005948339	0.892879	0.916169
LOC286002	0.089194161	0.043047	0.066762
LOC286094	-0.031867312	0.470534	0.544241
LOC286135	0.021272172	0.630073	0.692011
LOC286238	0.068536648	0.12033	0.167653
LOC286359	0.029537032	0.503611	0.576264
LOC286367	-0.358299518	4.80E-17	8.04E-16
LOC286467	0.24747563	1.26E-08	5.94E-08
LOC29034	0.247704275	1.22E-08	5.77E-08
LOC338588	0.057131247	0.19552	0.256906
LOC338651	-0.165178982	0.000166	0.000408
LOC338758	-0.188397423	1.68E-05	4.86E-05
LOC338799	-0.243795507	2.10E-08	9.55E-08
LOC339047	-0.36720796	6.94E-18	1.33E-16
LOC339240	-0.102139934	0.02043	0.033981
LOC339290	-0.25548221	4.06E-09	2.05E-08
LOC339524	-0.277782356	1.41E-10	8.86E-10
LOC339535	0.078710696	0.074317	0.109199
LOC339568	0.097310563	0.02723	0.04411
LOC339674	0.19163665	1.19E-05	3.53E-05
LOC339788	-0.108351335	0.013888	0.023928
LOC340017	-0.00165299	0.970149	0.977583
LOC340074	-0.006902407	0.875827	0.903072
LOC340357	-0.144311159	0.001023	0.002173
LOC340508	-0.202359797	3.67E-06	1.18E-05
LOC341056	0.134742108	0.002182	0.004371
LOC342346	-0.345952737	6.33E-16	9.01E-15
LOC344595	-0.160846082	0.000247	0.000587
LOC344967	0.040158749	0.363087	0.437613
LOC347376	0.041663479	0.34537	0.4196
LOC348021	0.033062246	0.454048	0.528076
LOC348840	-0.065750257	0.136199	0.187244
LOC348926	0.025373321	0.565626	0.634484
LOC349114	-0.261586261	1.67E-09	8.93E-09
LOC349196	-0.280205427	9.57E-11	6.18E-10
LOC360030	0.035022915	0.427715	0.50236
LOC374443	-0.000869795	0.98429	0.9883

LOC374491	-0.029271918	0.50745	0.580026
LOC375190	-0.177771493	4.97E-05	0.000133
LOC387646	0.039098978	0.375898	0.450717
LOC387647	0.024601685	0.577508	0.644981
LOC388152	-0.359312566	3.86E-17	6.59E-16
LOC388242	0.003825921	0.930979	0.945955
LOC388387	-0.260841758	1.86E-09	9.89E-09
LOC388428	-0.014141159	0.748854	0.797193
LOC388588	-0.234122863	7.66E-08	3.20E-07
LOC388692	-0.127849752	0.003658	0.007025
LOC388789	0.1312558	0.002842	0.005573
LOC388796	0.159198737	0.000287	0.000672
LOC388946	0.026776717	0.54432	0.614995
LOC388955	0.130436273	0.003021	0.005897
LOC389033	-0.167072886	0.000139	0.000347
LOC389332	-0.029101538	0.509925	0.582322
LOC389333	0.03640971	0.409637	0.484371
LOC389458	-0.029985756	0.497148	0.570207
LOC389493	-0.075018221	0.089	0.128175
LOC389634	-0.216185371	7.32E-07	2.62E-06
LOC389705	-0.129977528	0.003126	0.006085
LOC389791	-0.120657859	0.006115	0.011296
LOC390595	-0.280013804	9.87E-11	6.37E-10
LOC390858	-0.022457008	0.611133	0.675403
LOC391322	-0.168420261	0.000123	0.000308
LOC392196	-0.01687802	0.702373	0.757719
LOC399744	-0.22567241	2.27E-07	8.83E-07
LOC399815	0.307577037	9.55E-13	8.39E-12
LOC399959	0.061566515	0.162987	0.219232
LOC400027	-0.295844926	7.31E-12	5.59E-11
LOC400043	-0.021551217	0.62559	0.688297
LOC400657	-0.195814951	7.59E-06	2.31E-05
LOC400696	0.030039728	0.496373	0.56958
LOC400752	-0.28490361	4.49E-11	3.04E-10
LOC400759	-0.033253408	0.451441	0.525778
LOC400794	-0.327354488	2.50E-14	2.78E-13
LOC400804	-0.15247042	0.000517	0.001155
LOC400891	-0.283749771	5.42E-11	3.62E-10
LOC400927	-0.261633573	1.66E-09	8.88E-09
LOC400931	-0.273182409	2.89E-10	1.73E-09
LOC400940	-0.117601663	0.007549	0.013715
LOC401010	0.187623099	1.82E-05	5.25E-05
LOC401052	-0.14043488	0.001398	0.002896
LOC401093	-0.356600698	6.89E-17	1.13E-15

LOC401127	-0.120949472	0.005992	0.011089
LOC401387	-0.098250752	0.025771	0.041971
LOC401397	0.04064285	0.357326	0.43179
LOC401431	-0.168113906	0.000127	0.000317
LOC401463	-0.210490307	1.44E-06	4.93E-06
LOC401588	-0.117100677	0.007811	0.014153
LOC402377	0.093095726	0.034675	0.054854
LOC402644	0.024576616	0.577896	0.64527
LOC407835	0.164854263	0.000171	0.00042
LOC415056	-0.071000273	0.107536	0.151656
LOC440040	-0.011687039	0.791329	0.832609
LOC440173	0.095184596	0.030793	0.049286
LOC440354	-0.091184052	0.038586	0.060443
LOC440356	0.068075109	0.122853	0.170812
LOC440461	-0.200560403	4.50E-06	1.42E-05
LOC440563	0.104866112	0.017286	0.029161
LOC440896	-0.291542551	1.51E-11	1.10E-10
LOC440905	0.069120064	0.117197	0.16378
LOC440925	-0.045550237	0.302202	0.373857
LOC440944	-0.198980201	5.36E-06	1.67E-05
LOC440957	-0.063371664	0.150979	0.205012
LOC441046	-0.066601947	0.131186	0.181025
LOC441089	0.315167068	2.44E-13	2.34E-12
LOC441177	0.276969356	1.60E-10	9.96E-10
LOC441204	-0.306189132	1.22E-12	1.05E-11
LOC441208	0.098326454	0.025657	0.041792
LOC441294	-0.044544791	0.31301	0.385083
LOC441454	-0.137569301	0.001753	0.003566
LOC441455	-0.042398192	0.336923	0.410871
LOC441601	0.038172139	0.387328	0.462134
LOC441666	0.123561757	0.004985	0.009368
LOC441869	-0.323186835	5.51E-14	5.79E-13
LOC442308	0.182269234	3.17E-05	8.73E-05
LOC442421	-0.034828377	0.430287	0.504996
LOC442454	0.259183008	2.38E-09	1.24E-08
LOC442459	0.096691309	0.028229	0.045588
LOC493754	-0.082999451	0.059805	0.089865
LOC494141	0.059413295	0.178233	0.237248
LOC541471	0.331358173	1.16E-14	1.35E-13
LOC541473	-0.242202074	2.60E-08	1.17E-07
LOC550112	0.094779656	0.031515	0.050341
LOC550643	0.150633763	0.000604	0.001336
LOC554202	0.187592623	1.83E-05	5.26E-05
LOC55908	-0.074223151	0.092449	0.132492

LOC572558	-0.230019138	1.30E-07	5.28E-07
LOC595101	-0.073131859	0.097355	0.138886
LOC606724	-0.19168549	1.19E-05	3.52E-05
LOC613037	-0.085385058	0.052803	0.080335
LOC619207	-0.250495015	8.27E-09	4.00E-08
LOC641298	-0.148658355	0.000714	0.00156
LOC641367	0.017645147	0.689532	0.745961
LOC642587	-0.075809049	0.085674	0.123929
LOC642597	-0.023373839	0.596653	0.66247
LOC642826	-0.205667138	2.52E-06	8.29E-06
LOC642846	0.060017973	0.173849	0.232139
LOC642852	-0.089648527	0.041992	0.065297
LOC642929	0.023019463	0.602231	0.667373
LOC643008	-0.242334602	2.56E-08	1.15E-07
LOC643387	0.001273317	0.977003	0.983027
LOC643486	0.14327024	0.001113	0.002349
LOC643677	-0.103037645	0.019344	0.032328
LOC643719	-0.262131835	1.54E-09	8.31E-09
LOC643763	0.086452058	0.049901	0.076395
LOC643837	-0.070151447	0.111817	0.157051
LOC643923	-0.019398225	0.660526	0.719487
LOC643955	0.088408919	0.044923	0.069419
LOC644145	0.11978438	0.006498	0.011945
LOC644165	-0.41249422	1.41E-22	5.35E-21
LOC644172	-0.191527107	1.21E-05	3.57E-05
LOC644538	-0.040782596	0.355674	0.430183
LOC644669	-0.026740133	0.544871	0.615512
LOC644936	0.010159718	0.818087	0.854824
LOC645166	0.318279919	1.38E-13	1.37E-12
LOC645323	-0.130621174	0.00298	0.005822
LOC645332	-0.091677881	0.037541	0.058941
LOC645431	-0.119573689	0.006593	0.012108
LOC645676	-0.16621459	0.000151	0.000373
LOC645752	-0.12163648	0.005711	0.010611
LOC646214	0.047503542	0.281921	0.352608
LOC646471	-0.329559339	1.64E-14	1.88E-13
LOC646498	-0.080495262	0.067963	0.10082
LOC646627	0.051086581	0.247159	0.314924
LOC646762	-0.120844972	0.006036	0.011163
LOC646813	0.055838654	0.20584	0.268769
LOC646851	-0.241895331	2.71E-08	1.22E-07
LOC646982	-0.157923415	0.000321	0.000745
LOC646999	0.107992427	0.014208	0.024445
LOC647121	-0.253451779	5.44E-09	2.70E-08

LOC647288	0.15896269	0.000293	0.000685
LOC647309	-0.034076229	0.440317	0.514652
LOC647859	-0.056161831	0.203223	0.265839
LOC647946	0.290599836	1.76E-11	1.27E-10
LOC647979	-0.126529456	0.004027	0.007686
LOC648691	0.164303096	0.00018	0.00044
LOC648740	-0.442285275	4.46E-26	2.91E-24
LOC649330	0.303032018	2.12E-12	1.76E-11
LOC650293	0.02212892	0.616353	0.679967
LOC650368	-0.132073187	0.002672	0.005269
LOC650623	-0.200280641	4.64E-06	1.46E-05
LOC651250	-0.303874605	1.83E-12	1.54E-11
LOC652276	-0.021359256	0.628673	0.69108
LOC653113	-0.133466943	0.002405	0.004781
LOC653501	-0.364440013	1.27E-17	2.33E-16
LOC653544	-0.051602941	0.242407	0.309668
LOC653566	0.248773177	1.05E-08	5.02E-08
LOC653653	-0.069226933	0.116631	0.163102
LOC653786	-0.161582175	0.000231	0.000553
LOC654342	0.203741395	3.14E-06	1.02E-05
LOC654433	-0.004935476	0.911036	0.930447
LOC678655	-0.109318936	0.013055	0.022619
LOC723809	-0.401593442	2.22E-21	7.20E-20
LOC723972	0.136057694	0.001971	0.00398
LOC727677	-0.035679706	0.419096	0.493745
LOC727896	0.275232021	2.10E-10	1.29E-09
LOC727924	0.006326986	0.886105	0.910602
LOC728024	-0.030560663	0.488932	0.562171
LOC728190	0.054305698	0.218583	0.283048
LOC728264	-0.290869294	1.69E-11	1.22E-10
LOC728276	-0.083609841	0.057944	0.087312
LOC728323	-0.062299147	0.15803	0.213382
LOC728392	-0.424397681	6.21E-24	2.95E-22
LOC728554	0.219149093	5.10E-07	1.88E-06
LOC728606	-0.051181241	0.246283	0.313959
LOC728613	-0.012899546	0.770257	0.815245
LOC728640	0.089888555	0.041443	0.06455
LOC728643	0.15749277	0.000333	0.000771
LOC728723	-0.149813842	0.000648	0.001425
LOC728743	-0.141224626	0.001313	0.002733
LOC728758	0.156676573	0.000358	0.000824
LOC728819	0.038699615	0.380797	0.45535
LOC728855	0.093428896	0.034029	0.053935
LOC728875	0.145412812	0.000935	0.001997

LOC728989	-0.312325497	4.08E-13	3.79E-12
LOC729020	0.306337436	1.19E-12	1.03E-11
LOC729082	0.190627715	1.33E-05	3.90E-05
LOC729156	-0.03501506	0.427818	0.502452
LOC729176	0.234429821	7.35E-08	3.09E-07
LOC729234	-0.317004147	1.74E-13	1.70E-12
LOC729375	-0.098902687	0.0248	0.040511
LOC729467	0.056914656	0.197222	0.258887
LOC729603	-0.254900495	4.42E-09	2.22E-08
LOC729609	-0.115961956	0.008436	0.015176
LOC729668	0.026374478	0.550386	0.62062
LOC729678	-0.151097217	0.000581	0.001287
LOC729799	-0.29654522	6.49E-12	4.99E-11
LOC729991-MEF2B	-0.054436482	0.217474	0.281788
LOC729991	-0.060335627	0.171578	0.229428
LOC730101	0.056925839	0.197134	0.258806
LOC730668	-0.152909882	0.000497	0.001115
LOC731779	-0.008161179	0.853418	0.884206
LOC731789	0.094562354	0.031908	0.05088
LOC732275	-0.002283682	0.958768	0.96841
LOC80054	-0.345174431	7.42E-16	1.04E-14
LOC80154	-0.394207022	1.36E-20	3.83E-19
LOC81691	0.119898882	0.006447	0.011857
LOC84740	-0.081887504	0.063322	0.094608
LOC84856	-0.041862566	0.343068	0.417133
LOC84931	0.011776334	0.789771	0.831321
LOC84989	-0.024159713	0.584366	0.651111
LOC90110	-0.337444542	3.51E-15	4.48E-14
LOC90246	-0.095526935	0.030194	0.048459
LOC90586	-0.221420345	3.86E-07	1.45E-06
LOC90784	-0.089088325	0.043296	0.067088
LOC90834	-0.348630455	3.65E-16	5.40E-15
LOC91149	0.038763335	0.380013	0.454723
LOC91316	-0.374324637	1.42E-18	3.02E-17
LOC91450	-0.278011924	1.36E-10	8.56E-10
LOC91948	-0.028181239	0.523404	0.595402
LOC92249	-0.240106174	3.46E-08	1.52E-07
LOC92659	0.118797132	0.006956	0.012717
LOC92973	-0.505502543	9.31E-35	2.55E-32
LOC93432	-0.044508808	0.313401	0.385517
LOC93622	-0.031723061	0.472546	0.546331
LOC96610	0.039853245	0.366751	0.441445
LOH12CR1	0.246521497	1.44E-08	6.73E-08
LOH12CR2	-0.061892502	0.160767	0.216625

LOH3CR2A	-0.191182184	1.25E-05	3.69E-05
LONP1	0.051624612	0.242209	0.309446
LONP2	-0.035280044	0.424328	0.498969
LONRF1	-0.162414227	0.000214	0.000516
LONRF2	-0.23209522	9.97E-08	4.10E-07
LONRF3	-0.016575227	0.707464	0.761979
LOR	-0.280601365	8.99E-11	5.83E-10
LOXHD1	-0.17093356	9.68E-05	0.000247
LOXL1	-0.106999434	0.015129	0.025874
LOXL2	0.281926899	7.27E-11	4.77E-10
LOXL3	-0.119593849	0.006584	0.012094
LOXL4	-0.294516925	9.15E-12	6.90E-11
LOX	0.208073434	1.91E-06	6.41E-06
LPAL2	-0.185616139	2.25E-05	6.35E-05
LPAR1	0.128223316	0.003559	0.006852
LPAR2	-0.095527035	0.030193	0.048459
LPAR3	-0.130699058	0.002963	0.005792
LPAR4	0.117844464	0.007425	0.01351
LPAR5	-0.094839	0.031408	0.050191
LPAR6	-0.172130795	8.63E-05	0.000222
LPA	-0.15641871	0.000367	0.000841
LPCAT1	-0.235936436	6.03E-08	2.56E-07
LPCAT2	-0.290264803	1.86E-11	1.34E-10
LPCAT3	-0.188100259	1.73E-05	5.01E-05
LPCAT4	0.039625488	0.369499	0.44427
LPGAT1	0.231256239	1.11E-07	4.55E-07
LPHN1	-0.252818022	5.95E-09	2.94E-08
LPHN2	-0.074850634	0.089718	0.129079
LPHN3	-0.077606997	0.078483	0.114664
LPIN1	-0.244052577	2.02E-08	9.25E-08
LPIN2	-0.163199607	0.0002	0.000483
LPIN3	-0.260353324	2.00E-09	1.06E-08
LPL	-0.240839792	3.13E-08	1.39E-07
LPO	-0.027239299	0.537386	0.608192
LPPR1	-0.207505745	2.04E-06	6.80E-06
LPPR2	-0.230235409	1.27E-07	5.15E-07
LPPR3	-0.111375618	0.011431	0.020049
LPPR4	-0.337631899	3.38E-15	4.33E-14
LPPR5	-0.109873213	0.012599	0.021878
LPP	0.083050242	0.059648	0.089643
LPXN	-0.208862947	1.74E-06	5.88E-06
LQK1	-0.024445862	0.579921	0.646954
LRAT	-0.191530316	1.21E-05	3.57E-05
LRBA	-0.078207188	0.076194	0.11177

LRCH1	-0.10230238	0.020229	0.03367
LRCH2	-0.106631326	0.015483	0.026401
LRCH3	-0.021077736	0.633205	0.694801
LRCH4	-0.366096062	8.87E-18	1.68E-16
LRDD	-0.247272239	1.30E-08	6.10E-08
LRFN1	-0.114341783	0.009403	0.016776
LRFN2	-0.036271136	0.411422	0.486138
LRFN3	-0.096560448	0.028444	0.045902
LRFN4	0.226311259	2.09E-07	8.20E-07
LRFN5	-0.089982454	0.04123	0.064248
LRG1	-0.088647944	0.044345	0.068605
LRGUK	-0.229664674	1.37E-07	5.51E-07
LRIG1	-0.32553282	3.54E-14	3.85E-13
LRIG2	-0.193643376	9.61E-06	2.89E-05
LRIG3	-0.331046737	1.23E-14	1.43E-13
LRIT1	0.131376575	0.002816	0.005526
LRIT2	-0.081637957	0.064134	0.0957
LRIT3	-0.31027633	5.90E-13	5.36E-12
LRMP	-0.247351853	1.28E-08	6.03E-08
LRP10	0.062930512	0.15385	0.208403
LRP11	0.118911394	0.006901	0.012628
LRP12	0.231937295	1.02E-07	4.19E-07
LRP1B	0.072304755	0.10121	0.143821
LRP1	-0.240226632	3.40E-08	1.50E-07
LRP2BP	-0.463814523	7.83E-29	7.25E-27
LRP2	-0.190015237	1.42E-05	4.15E-05
LRP3	-0.204219169	2.97E-06	9.66E-06
LRP4	0.0450522	0.307524	0.379408
LRP5L	-0.296409485	6.64E-12	5.11E-11
LRP5	0.019791275	0.65409	0.71376
LRP6	-0.146897557	0.000827	0.001784
LRP8	0.203266769	3.31E-06	1.07E-05
LRPAP1	-0.108808667	0.013489	0.02329
LRPPRC	0.364413132	1.28E-17	2.35E-16
LRRC10B	-0.119602746	0.00658	0.012088
LRRC10	-0.001097731	0.980174	0.98531
LRRC14B	0.018846734	0.669599	0.727585
LRRC14	-0.074399346	0.091675	0.131544
LRRC15	0.082489755	0.061396	0.092069
LRRC16A	0.045289014	0.304986	0.376742
LRRC16B	-0.138729848	0.0016	0.003278
LRRC17	-0.109769851	0.012683	0.022009
LRRC18	-0.254123961	4.94E-09	2.46E-08
LRRC19	-0.033199664	0.452173	0.526519

LRRC1	0.213104589	1.06E-06	3.70E-06
LRRC20	-0.073462834	0.095846	0.136899
LRRC23	-0.168708975	0.00012	0.000301
LRRC24	-0.06811226	0.122649	0.170599
LRRC25	-0.122418435	0.005405	0.010094
LRRC26	0.022309834	0.613472	0.677351
LRRC27	-0.299690458	3.79E-12	3.01E-11
LRRC28	0.132750464	0.002539	0.005025
LRRC29	-0.264413066	1.10E-09	6.06E-09
LRRC2	-0.17777035	4.97E-05	0.000133
LRRC31	-0.286768342	3.32E-11	2.29E-10
LRRC32	-0.105637979	0.016476	0.027935
LRRC33	-0.263247628	1.30E-09	7.10E-09
LRRC34	-0.027480716	0.533785	0.605075
LRRC36	-0.422983799	9.06E-24	4.15E-22
LRRC37A2	-0.27690851	1.61E-10	1.01E-09
LRRC37A3	-0.101162791	0.021672	0.035863
LRRC37A4	-0.315379608	2.34E-13	2.26E-12
LRRC37A	-0.317884509	1.48E-13	1.47E-12
LRRC37B2	0.03769362	0.39331	0.468197
LRRC37B	-0.314437257	2.78E-13	2.64E-12
LRRC39	-0.27303094	2.96E-10	1.77E-09
LRRC3B	-0.129152943	0.003323	0.00644
LRRC3	-0.244716305	1.85E-08	8.49E-08
LRRC40	0.305694092	1.33E-12	1.14E-11
LRRC41	-0.063775886	0.148385	0.201873
LRRC42	0.359761428	3.51E-17	6.04E-16
LRRC43	-0.127609911	0.003723	0.007143
LRRC45	-0.063954342	0.14725	0.200534
LRRC46	-0.204598805	2.85E-06	9.28E-06
LRRC47	-0.072460628	0.100475	0.142888
LRRC48	-0.309244318	7.10E-13	6.37E-12
LRRC49	-0.124405973	0.004693	0.008865
LRRC4B	-0.313263357	3.45E-13	3.24E-12
LRRC4C	-0.205501779	2.57E-06	8.43E-06
LRRC4	-0.258101157	2.78E-09	1.43E-08
LRRC50	-0.241039343	3.05E-08	1.35E-07
LRRC52	-0.25569365	3.94E-09	1.99E-08
LRRC55	-0.302716833	2.24E-12	1.85E-11
LRRC56	-0.28448236	4.81E-11	3.24E-10
LRRC57	0.035193898	0.425461	0.500124
LRRC58	0.033492297	0.448195	0.522428
LRRC59	0.473467467	3.92E-30	4.45E-28
LRRC61	0.245107955	1.75E-08	8.07E-08

LRRC66	0.060659492	0.169285	0.226773
LRRC67	-0.197582854	6.25E-06	1.93E-05
LRRC69	0.033838782	0.443511	0.517719
LRRC6	-0.074711765	0.090317	0.129865
LRRC70	-0.213144296	1.05E-06	3.68E-06
LRRC7	0.014149072	0.748719	0.797091
LRRC8A	-0.041096428	0.351981	0.426595
LRRC8B	-0.005352816	0.903548	0.924501
LRRC8C	-0.164692534	0.000174	0.000426
LRRC8D	0.058748721	0.183146	0.242682
LRRC8E	-0.112890547	0.010351	0.018316
LRRCC1	0.082316124	0.061946	0.092797
LRRFIP1	-0.099699647	0.023655	0.0388
LRRFIP2	-0.052458725	0.234674	0.301128
LRRIQ1	-0.147555788	0.000783	0.001697
LRRIQ3	-0.017278795	0.695653	0.751606
LRRIQ4	0.185186748	2.35E-05	6.62E-05
LRRK1	-0.25238262	6.33E-09	3.11E-08
LRRK2	-0.406713539	6.17E-22	2.16E-20
LRRN1	-0.094778047	0.031518	0.050342
LRRN2	-0.135791869	0.002012	0.004058
LRRN3	-0.331571006	1.11E-14	1.30E-13
LRRN4CL	-0.158321309	0.00031	0.000722
LRRN4	-0.234884889	6.93E-08	2.92E-07
LRRTM1	-0.225014381	2.47E-07	9.53E-07
LRRTM2	-0.122867432	0.005236	0.009804
LRRTM3	0.104153815	0.018063	0.030384
LRRTM4	-0.049943015	0.257912	0.326656
LRSAM1	-0.246353627	1.47E-08	6.87E-08
LRTM1	0.006595787	0.881301	0.906934
LRTM2	0.056716769	0.198787	0.260565
LRTOMT	-0.144195867	0.001032	0.00219
LRWD1	0.128959508	0.003371	0.006521
LSAMP	-0.304310948	1.70E-12	1.43E-11
LSG1	0.195172386	8.14E-06	2.47E-05
LSM10	0.079199324	0.072531	0.106842
LSM11	0.063748095	0.148562	0.202087
LSM12	0.388970421	4.76E-20	1.24E-18
LSM14A	0.119213813	0.006759	0.012392
LSM14B	-0.050141458	0.256023	0.324605
LSM1	0.238668354	4.19E-08	1.83E-07
LSM2	0.325273643	3.72E-14	4.03E-13
LSM3	0.25079246	7.93E-09	3.85E-08
LSM4	0.245242961	1.72E-08	7.93E-08

LSM5	0.35575488	8.24E-17	1.33E-15
LSM6	0.17730653	5.21E-05	0.000139
LSM7	0.099512696	0.023919	0.039192
LSMD1	0.024815972	0.574196	0.641892
LSP1	-0.281481246	7.81E-11	5.11E-10
LSR	-0.087286158	0.047725	0.073261
LSS	-0.260879317	1.85E-09	9.84E-09
LST-3TM12	0.012335042	0.780048	0.822992
LST1	-0.215720742	7.74E-07	2.76E-06
LTA4H	-0.18074888	3.69E-05	0.000101
LTA	-0.184971382	2.40E-05	6.76E-05
LTB4R2	-0.250070198	8.77E-09	4.23E-08
LTB4R	-0.21209558	1.19E-06	4.13E-06
LTBP1	-0.037408695	0.396899	0.471822
LTBP2	-0.311397039	4.83E-13	4.42E-12
LTBP3	-0.328558119	1.99E-14	2.24E-13
LTBP4	-0.360767647	2.83E-17	4.93E-16
LTBR	0.274427698	2.38E-10	1.45E-09
LTB	-0.251832727	6.84E-09	3.35E-08
LTC4S	-0.419288317	2.41E-23	1.04E-21
LTF	-0.170179897	0.000104	0.000264
LTK	-0.040971206	0.353452	0.428065
LTV1	0.280480574	9.16E-11	5.93E-10
LUC7L2	-0.008324261	0.850523	0.881938
LUC7L3	-0.294900504	8.58E-12	6.49E-11
LUC7L	-0.276111257	1.83E-10	1.13E-09
LUM	-0.067907002	0.123783	0.171818
LUZP1	-0.037388605	0.397153	0.472012
LUZP2	-0.292560548	1.27E-11	9.37E-11
LUZP4	0.034268005	0.437747	0.512037
LUZP6	0.299466461	3.94E-12	3.11E-11
LXN	0.008680749	0.844201	0.876657
LY6D	0.088145478	0.045568	0.070301
LY6E	-0.160411005	0.000257	0.000609
LY6G5B	-0.236155408	5.86E-08	2.49E-07
LY6G5C	-0.170221883	0.000104	0.000263
LY6G6C	0.039162352	0.375124	0.449951
LY6G6D	-0.0113076	0.797955	0.838345
LY6G6E	-0.00788087	0.858398	0.888538
LY6G6F	-0.094765477	0.03154	0.050374
LY6H	-0.040268943	0.36177	0.436342
LY6K	0.158960083	0.000293	0.000685
LY75	-0.155288164	0.000405	0.000922
LY86	-0.302458546	2.35E-12	1.93E-11

LY96	0.021281315	0.629926	0.691906
LY9	-0.174295027	7.00E-05	0.000182
LYAR	0.472299638	5.66E-30	6.25E-28
LYG1	0.013326357	0.762879	0.809279
LYG2	0.104831878	0.017322	0.029212
LYL1	-0.320428672	9.24E-14	9.40E-13
LYNX1	-0.313335227	3.40E-13	3.20E-12
LYN	0.052449337	0.234758	0.301197
LYPD1	0.13538142	0.002077	0.00418
LYPD2	-0.149716266	0.000653	0.001436
LYPD3	0.246693692	1.41E-08	6.57E-08
LYPD4	0.003246186	0.941417	0.953679
LYPD5	0.02093937	0.635438	0.696792
LYPD6B	0.037142881	0.400265	0.474809
LYPD6	0.034281798	0.437563	0.511911
LYPLA1	0.375269766	1.15E-18	2.47E-17
LYPLA2P1	-0.038003475	0.38943	0.464241
LYPLA2	-0.126043016	0.004172	0.00794
LYPLAL1	0.065127394	0.139957	0.191777
LYRM1	-0.066622569	0.131066	0.180872
LYRM2	-0.001047454	0.981082	0.985965
LYRM4	0.251290861	7.39E-09	3.61E-08
LYRM5	0.041374792	0.348726	0.423188
LYRM7	-0.095763304	0.029786	0.047873
LYSMD1	0.143196519	0.00112	0.002361
LYSMD2	-0.110488578	0.012108	0.0211
LYSMD3	0.036978557	0.402354	0.477002
LYSMD4	-0.282016584	7.16E-11	4.71E-10
LYST	-0.330810436	1.29E-14	1.49E-13
LYVE1	-0.00644564	0.883984	0.90909
LYZL1	0.084011002	0.056748	0.085722
LYZL2	0.18950599	1.50E-05	4.36E-05
LYZL4	-0.094366141	0.032267	0.051407
LYZL6	0.034538065	0.434143	0.508744
LYZ	-0.192505788	1.09E-05	3.23E-05
LZIC	0.248806554	1.05E-08	5.00E-08
LZTFL1	-0.187338121	1.88E-05	5.39E-05
LZTR1	-0.200385878	4.58E-06	1.45E-05
LZTS1	-0.035809447	0.417405	0.492073
LZTS2	-0.121641214	0.005709	0.010608
M6PR	0.134644301	0.002198	0.004402
MAB21L1	-0.262869314	1.38E-09	7.48E-09
MAB21L2	-0.174738709	6.70E-05	0.000175
MACC1	-0.265738838	8.99E-10	5.04E-09

MACF1	-0.382230007	2.32E-19	5.58E-18
MACROD1	-0.083564426	0.058081	0.087485
MACROD2	-0.343223231	1.10E-15	1.52E-14
MAD1L1	-0.082517988	0.061307	0.091956
MAD2L1BP	0.209995991	1.53E-06	5.19E-06
MAD2L1	0.578696263	2.26E-47	6.46E-44
MAD2L2	0.160777526	0.000249	0.00059
MADCAM1	-0.102663785	0.01979	0.033018
MADD	-0.333629725	7.44E-15	8.97E-14
MAEA	0.111105391	0.011633	0.020358
MAEL	-0.101782771	0.020877	0.034672
MAF1	0.149091998	0.000688	0.001509
MAFA	0.156701573	0.000358	0.000823
MAFB	-0.107806803	0.014376	0.0247
MAFF	0.018965588	0.667639	0.725892
MAFG	0.087777332	0.046482	0.0716
MAFK	0.032005235	0.468615	0.542366
MAF	-0.1587492	0.000298	0.000697
MAGEA10	0.073569505	0.095363	0.136288
MAGEA11	0.164828443	0.000172	0.000421
MAGEA12	0.197573375	6.26E-06	1.93E-05
MAGEA1	0.239903759	3.55E-08	1.56E-07
MAGEA2	0.228108727	1.67E-07	6.61E-07
MAGEA3	0.25098437	7.71E-09	3.76E-08
MAGEA4	0.099911753	0.023358	0.038373
MAGEA5	-0.003264337	0.94109	0.953493
MAGEA6	0.243045925	2.32E-08	1.05E-07
MAGEA8	0.111739204	0.011163	0.019615
MAGEA9B	0.107064646	0.015067	0.025784
MAGEB10	0.028038351	0.525513	0.597506
MAGEB16	0.084737434	0.054633	0.082891
MAGEB18	0.11838153	0.007157	0.013056
MAGEB1	0.1251675	0.004444	0.008419
MAGEB2	0.213716227	9.84E-07	3.46E-06
MAGEB3	0.07354461	0.095476	0.136429
MAGEB4	0.04992495	0.258085	0.326845
MAGEB6	0.2015401	4.03E-06	1.28E-05
MAGEC1	0.181666957	3.37E-05	9.25E-05
MAGEC2	0.190555046	1.34E-05	3.93E-05
MAGEC3	-0.032401192	0.463128	0.537042
MAGED1	0.068329884	0.121455	0.16908
MAGED2	0.035563997	0.420607	0.495234
MAGED4B	-0.004033662	0.927241	0.943139
MAGED4	-0.00046857	0.991536	0.993724

MAGEE1	-0.302930668	2.16E-12	1.79E-11
MAGEE2	-0.323520016	5.18E-14	5.47E-13
MAGEF1	0.033197847	0.452198	0.526519
MAGEH1	-0.116128348	0.008342	0.015031
MAGEL2	-0.067315777	0.127095	0.17589
MAGI1	-0.303014532	2.13E-12	1.77E-11
MAGI2	-0.305535795	1.37E-12	1.17E-11
MAGI3	-0.330606887	1.34E-14	1.55E-13
MAGIX	-0.122066206	0.005541	0.010328
MAGOHB	0.413877244	9.90E-23	3.88E-21
MAGOH	0.244201869	1.98E-08	9.08E-08
MAGT1	0.151765381	0.000549	0.001221
MAG	-0.193413122	9.85E-06	2.95E-05
MAK16	0.200848474	4.35E-06	1.38E-05
MAK	-0.213998176	9.51E-07	3.35E-06
MAL2	-0.086937373	0.048625	0.074574
MALAT1	-0.33350911	7.62E-15	9.15E-14
MALL	-0.30487344	1.54E-12	1.31E-11
MALT1	-0.087769047	0.046503	0.071627
MAL	-0.331397803	1.15E-14	1.34E-13
MAMDC2	-0.406214348	7.00E-22	2.43E-20
MAMDC4	-0.261913425	1.59E-09	8.54E-09
MAML1	-0.207233956	2.11E-06	7.00E-06
MAML2	-0.301295208	2.87E-12	2.33E-11
MAML3	-0.157124339	0.000345	0.000795
MAMLD1	-0.083109775	0.059465	0.089381
MAMSTR	0.145853127	0.000901	0.00193
MAN1A1	-0.027327805	0.536064	0.60704
MAN1A2	0.083126678	0.059413	0.08933
MAN1B1	-0.017259668	0.695974	0.751871
MAN1C1	-0.424200774	6.54E-24	3.09E-22
MAN2A1	0.148761136	0.000708	0.001548
MAN2A2	-0.211624175	1.26E-06	4.35E-06
MAN2B1	-0.296609919	6.42E-12	4.94E-11
MAN2B2	-0.21294439	1.08E-06	3.76E-06
MAN2C1	-0.332151018	9.92E-15	1.17E-13
MANBAL	0.150615047	0.000605	0.001338
MANBA	-0.179083524	4.36E-05	0.000118
MANEAL	-0.114111837	0.009548	0.01702
MANEA	0.037184472	0.399737	0.474378
MANF	0.331011463	1.24E-14	1.44E-13
MANSC1	0.001936477	0.965033	0.973164
MAOA	-0.196316891	7.19E-06	2.20E-05
MAOB	-0.323496738	5.20E-14	5.49E-13

MAP1A	-0.109142386	0.013204	0.022844
MAP1B	0.026291078	0.551648	0.621625
MAP1D	0.036399466	0.409768	0.48447
MAP1LC3A	-0.276289745	1.78E-10	1.10E-09
MAP1LC3B2	0.17828285	4.73E-05	0.000127
MAP1LC3B	0.060643308	0.169399	0.226865
MAP1LC3C	-0.217383634	6.33E-07	2.30E-06
MAP1S	-0.188017953	1.75E-05	5.05E-05
MAP2K1	0.196668415	6.92E-06	2.12E-05
MAP2K2	0.080444744	0.068137	0.101062
MAP2K3	-0.093780635	0.033359	0.05296
MAP2K4	0.13209731	0.002668	0.005261
MAP2K5	-0.123322834	0.00507	0.009517
MAP2K6	0.111199465	0.011563	0.02025
MAP2K7	-0.262075169	1.55E-09	8.37E-09
MAP2	0.039165943	0.37508	0.449925
MAP3K10	-0.013787406	0.754934	0.80217
MAP3K11	-0.160281196	0.00026	0.000615
MAP3K12	-0.283955211	5.24E-11	3.51E-10
MAP3K13	-0.273560296	2.72E-10	1.64E-09
MAP3K14	-0.321345563	7.78E-14	8.02E-13
MAP3K15	-0.221320147	3.91E-07	1.46E-06
MAP3K1	-0.28606234	3.72E-11	2.55E-10
MAP3K2	0.100499517	0.022552	0.037171
MAP3K3	-0.496085933	2.40E-33	4.89E-31
MAP3K4	-0.14427064	0.001026	0.00218
MAP3K5	-0.227716453	1.75E-07	6.94E-07
MAP3K6	-0.351891819	1.86E-16	2.84E-15
MAP3K7	0.108692862	0.013589	0.023455
MAP3K8	-0.153247302	0.000483	0.001086
MAP3K9	-0.00112202	0.979735	0.985008
MAP4K1	-0.2637164	1.22E-09	6.66E-09
MAP4K2	-0.133374654	0.002422	0.004811
MAP4K3	0.216383782	7.14E-07	2.56E-06
MAP4K4	0.219120264	5.12E-07	1.89E-06
MAP4K5	0.188630516	1.64E-05	4.75E-05
MAP4	-0.033572938	0.447103	0.521394
MAP6D1	0.256326464	3.60E-09	1.83E-08
MAP6	-0.363468091	1.57E-17	2.84E-16
MAP7D1	-0.138626348	0.001613	0.003303
MAP7D2	-0.161609303	0.000231	0.000552
MAP7D3	-0.032483668	0.46199	0.535878
MAP7	0.078826316	0.073891	0.10863
MAP9	-0.186370984	2.08E-05	5.92E-05

MAPK10		-0.466547673	3.39E-29	3.25E-27
MAPK11		-0.15925534	0.000285	0.000669
MAPK12		0.135419071	0.002071	0.004168
MAPK13		-0.011929172	0.787108	0.829041
MAPK14		0.023584142	0.593353	0.659289
MAPK15		-0.194363852	8.89E-06	2.68E-05
MAPK1IP1L		0.348827126	3.51E-16	5.19E-15
MAPK1		0.018108296	0.681822	0.738819
MAPK3		-0.130388184	0.003032	0.005915
MAPK4		-0.119276255	0.00673	0.012345
MAPK6		0.406908795	5.87E-22	2.06E-20
MAPK7		-0.141832125	0.00125	0.002615
MAPK8IP1		-0.259381513	2.31E-09	1.21E-08
MAPK8IP2		-0.057542885	0.192314	0.253243
MAPK8IP3		-0.348138553	4.04E-16	5.93E-15
MAPK8		0.309597393	6.67E-13	6.00E-12
MAPK9		-0.067294674	0.127215	0.176043
MAPKAP1		0.137613614	0.001746	0.003556
MAPKAPK2		-0.0778313	0.077621	0.113544
MAPKAPK3		-0.136706517	0.001874	0.003796
MAPKAPK5		0.410499094	2.36E-22	8.65E-21
MAPKBP1		-0.358758424	4.35E-17	7.34E-16
MAPKSP1		0.037755354	0.392536	0.467413
MAPRE1		0.374293504	1.43E-18	3.03E-17
MAPRE2		-0.139472111	0.001509	0.00311
MAPRE3		-0.355968546	7.88E-17	1.28E-15
MAPT		-0.204748807	2.80E-06	9.13E-06
	10-Mar	-0.094538936	0.031951	0.050944
	11-Mar	0.073705995	0.094749	0.135477
	1-Mar	-0.135512194	0.002056	0.00414
	2-Mar	-0.187736387	1.80E-05	5.19E-05
	3-Mar	-0.072954531	0.098172	0.139951
	4-Mar	0.047226446	0.28474	0.355623
	5-Mar	0.37217807	2.30E-18	4.71E-17
	6-Mar	0.037379572	0.397267	0.472088
	7-Mar	0.202398018	3.66E-06	1.17E-05
	8-Mar	-0.075087093	0.088707	0.127807
	9-Mar	-0.207183827	2.12E-06	7.04E-06
MARCKSL1		0.21935428	4.98E-07	1.84E-06
MARCKS		0.164968889	0.00017	0.000416
MARCO		-0.215151672	8.28E-07	2.94E-06
MARK1		0.024900818	0.572888	0.641003
MARK2		0.009644981	0.827154	0.862405
MARK3		0.069027644	0.117689	0.164341

MARK4	0.072300645	0.10123	0.143835
MARS2	0.29108187	1.63E-11	1.18E-10
MARS	0.378393928	5.62E-19	1.27E-17
MARVELD1	0.060506307	0.170367	0.227999
MARVELD2	-0.163869268	0.000188	0.000456
MARVELD3	0.047321576	0.28377	0.354588
MAS1L	-0.323551567	5.15E-14	5.44E-13
MAS1	-0.014849192	0.736734	0.786931
MASP1	-0.258878232	2.48E-09	1.29E-08
MASP2	-0.391408746	2.66E-20	7.27E-19
MAST1	0.130579222	0.002989	0.00584
MAST2	-0.015368889	0.727879	0.779472
MAST3	-0.384462793	1.38E-19	3.43E-18
MAST4	-0.285483935	4.09E-11	2.78E-10
MASTL	0.351268155	2.11E-16	3.20E-15
MAT1A	0.012571484	0.775943	0.819663
MAT2A	-0.247169953	1.32E-08	6.18E-08
MAT2B	-0.101139456	0.021703	0.035904
MATK	-0.227169541	1.88E-07	7.40E-07
MATN1	-0.106787607	0.015332	0.026172
MATN2	-0.144533634	0.001004	0.002136
MATN3	-0.09220509	0.036453	0.057403
MATN4	-0.198789412	5.47E-06	1.71E-05
MATR3	0.04498985	0.308195	0.380095
MAVS	-0.134481836	0.002225	0.004452
MAX	0.054288939	0.218725	0.283208
MAZ	0.197079009	6.61E-06	2.03E-05
MBD1	-0.056734153	0.198649	0.260429
MBD2	0.00210763	0.961945	0.970882
MBD3L1	0.044186623	0.31692	0.389319
MBD3L2	0.062443106	0.157069	0.212229
MBD3L5	0.052421932	0.235003	0.301473
MBD3	-0.085484021	0.052528	0.079983
MBD4	0.013168034	0.765614	0.811318
MBD5	-0.177829226	4.95E-05	0.000132
MBD6	-0.209809503	1.56E-06	5.30E-06
MBIP	-0.273091461	2.93E-10	1.76E-09
MBL1P	-0.443898397	2.81E-26	1.91E-24
MBL2	-0.000688297	0.987568	0.99069
MBLAC1	0.044251435	0.31621	0.38859
MBLAC2	-0.127505072	0.003751	0.007195
MBNL1	-0.222014838	3.59E-07	1.35E-06
MBNL2	-0.142067012	0.001227	0.002569
MBNL3	-0.141067806	0.001329	0.002764

MBOAT1	-0.189501069	1.50E-05	4.36E-05
MBOAT2	-0.031831343	0.471035	0.544758
MBOAT4	-0.127881346	0.003649	0.007011
MBOAT7	-0.063910443	0.147529	0.200845
MBP	-0.117505425	0.007599	0.0138
MBTD1	-0.134437349	0.002233	0.004465
MBTPS1	-0.032955975	0.455501	0.529481
MBTPS2	0.202686224	3.54E-06	1.14E-05
MB	0.134832974	0.002166	0.004344
MC1R	0.162162896	0.000219	0.000527
MC2R	-0.15599144	0.000381	0.000871
MC3R	0.018731763	0.671496	0.72933
MC4R	-0.111145221	0.011603	0.020311
MC5R	-0.260547346	1.94E-09	1.03E-08
MCAM	0.120515095	0.006176	0.0114
MCART1	0.115302478	0.008818	0.015803
MCART2	0.069837725	0.113432	0.159197
MCART3P	0.022750925	0.606474	0.671219
MCART6	-0.183903681	2.68E-05	7.49E-05
MCAT	0.041971367	0.341814	0.416031
MCCC1	-0.258374427	2.67E-09	1.38E-08
MCCC2	0.105440481	0.01668	0.02824
MCCD1	0.060109239	0.173194	0.231373
MCC	-0.273682197	2.67E-10	1.61E-09
MCEE	-0.038568689	0.382412	0.457027
MCF2L2	0.131327709	0.002827	0.005545
MCF2L	-0.163617465	0.000192	0.000466
MCF2	-0.030818183	0.485276	0.558899
MCFD2	0.231583108	1.07E-07	4.37E-07
MCHR1	0.111360729	0.011442	0.020067
MCHR2	0.147195422	0.000807	0.001744
MCL1	-0.05033861	0.254156	0.322647
MCM10	0.515123994	3.03E-36	1.10E-33
MCM2	0.322272523	6.55E-14	6.82E-13
MCM3APAS	0.014258409	0.746843	0.795349
MCM3AP	-0.24689347	1.37E-08	6.40E-08
MCM3	0.206183742	2.38E-06	7.84E-06
MCM4	0.439796894	9.01E-26	5.70E-24
MCM5	0.149404493	0.00067	0.001472
MCM6	0.39020421	3.55E-20	9.55E-19
MCM7	0.358924747	4.20E-17	7.12E-16
MCM8	0.257141587	3.20E-09	1.63E-08
MCM9	-0.213366839	1.03E-06	3.60E-06
MCOLN1	-0.276606669	1.69E-10	1.05E-09

MCOLN2	-0.187516975	1.84E-05	5.30E-05
MCOLN3	-0.219372569	4.97E-07	1.84E-06
MCPH1	0.03748244	0.395968	0.470856
MCRS1	0.249837693	9.07E-09	4.37E-08
MCTP1	-0.072466767	0.100446	0.142867
MCTP2	-0.184363735	2.56E-05	7.17E-05
MCTS1	0.176275576	5.77E-05	0.000152
MDC1	-0.010935531	0.804466	0.84359
MDFIC	0.017531483	0.691429	0.747649
MDFI	0.207266157	2.10E-06	6.98E-06
MDGA1	-0.234584985	7.21E-08	3.03E-07
MDGA2	0.178768184	4.50E-05	0.000121
MDH1B	-0.199682406	4.96E-06	1.55E-05
MDH1	0.254083843	4.97E-09	2.48E-08
MDH2	0.36256394	1.92E-17	3.41E-16
MDK	0.087492824	0.047199	0.07258
MDM1	-0.032133672	0.466831	0.540678
MDM2	0.01952683	0.658417	0.71762
MDM4	-0.272336405	3.29E-10	1.96E-09
MDN1	-0.182604623	3.06E-05	8.46E-05
MDP1	-0.002822687	0.949049	0.960291
MDS2	-0.166149301	0.000152	0.000375
ME1	0.238452886	4.32E-08	1.88E-07
ME2	0.056500667	0.200506	0.26268
ME3	-0.088499016	0.044705	0.069107
MEA1	0.153717837	0.000464	0.001046
MEAF6	-0.17563608	6.14E-05	0.000161
MECOM	-0.238222862	4.45E-08	1.93E-07
MECP2	-0.322097254	6.76E-14	7.03E-13
MECR	-0.08485792	0.054289	0.082437
MED10	0.120193812	0.006316	0.011639
MED11	-0.08367129	0.05776	0.087066
MED12L	-0.063640468	0.14925	0.20294
MED12	-0.114186117	0.009501	0.016939
MED13L	-0.030607525	0.488266	0.561566
MED13	-0.037840042	0.391474	0.466344
MED14	0.068088814	0.122778	0.170719
MED15	-0.185293049	2.32E-05	6.55E-05
MED16	-0.217969219	5.89E-07	2.15E-06
MED17	0.174099426	7.14E-05	0.000186
MED18	0.021321934	0.629273	0.691626
MED19	0.203577402	3.20E-06	1.03E-05
MED1	0.045047524	0.307574	0.379447
MED20	0.230330747	1.25E-07	5.09E-07

MED21	0.161513375	0.000233	0.000556
MED22	-0.109201312	0.013154	0.022778
MED23	-0.151189568	0.000576	0.001279
MED24	-0.001599229	0.97112	0.978265
MED25	-0.219641946	4.80E-07	1.78E-06
MED26	-0.337469631	3.49E-15	4.46E-14
MED27	0.264332211	1.11E-09	6.13E-09
MED28	0.213901684	9.62E-07	3.39E-06
MED29	-0.183126007	2.90E-05	8.06E-05
MED30	0.181245694	3.51E-05	9.63E-05
MED31	0.111127209	0.011617	0.020331
MED4	0.007831454	0.859277	0.889309
MED6	0.373223542	1.82E-18	3.76E-17
MED7	-0.049672725	0.2605	0.329341
MED8	0.145531879	0.000925	0.001979
MED9	-0.134417928	0.002236	0.004471
MEF2A	-0.34104932	1.71E-15	2.29E-14
MEF2B	-0.117296083	0.007708	0.013978
MEF2C	-0.365137738	1.09E-17	2.03E-16
MEF2D	-0.110928663	0.011768	0.020562
MEFV	-0.083022161	0.059735	0.089766
MEG3	-0.174489199	6.87E-05	0.000179
MEG8	-0.068389281	0.121131	0.168687
MEGF10	0.004081222	0.926386	0.942416
MEGF11	-0.379827605	4.04E-19	9.34E-18
MEGF6	-0.480015015	4.87E-31	6.38E-29
MEGF8	-0.110598695	0.012022	0.020961
MEGF9	-0.152315746	0.000523	0.00117
MEI1	-0.100600299	0.022417	0.03697
MEIG1	-0.08226388	0.062113	0.093032
MEIS1	-0.311389575	4.83E-13	4.42E-12
MEIS2	-0.055280889	0.210412	0.273917
MEIS3P1	-0.185983453	2.16E-05	6.14E-05
MEIS3	0.048644235	0.270512	0.340384
MELK	0.497891426	1.30E-33	2.78E-31
MEMO1	0.382705677	2.08E-19	5.01E-18
MEN1	-0.003175559	0.942689	0.954484
MEOX1	-0.357819257	5.31E-17	8.88E-16
MEOX2	-0.242369953	2.55E-08	1.15E-07
MEP1A	-0.089257109	0.0429	0.066567
MEP1B	0.048631637	0.270637	0.340498
MEPCE	0.009702315	0.826143	0.861553
MEPE	0.13279911	0.00253	0.005007
MERTK	-0.287043935	3.17E-11	2.20E-10

MESDC1	0.034375409	0.436312	0.510806
MESDC2	0.242202322	2.60E-08	1.17E-07
MESP1	-0.1452994	0.000943	0.002014
MESP2	-0.137057247	0.001824	0.003702
MESTIT1	-0.140341875	0.001408	0.002917
MEST	0.371165219	2.88E-18	5.82E-17
METAP1	0.341930982	1.43E-15	1.93E-14
METAP2	0.270604489	4.29E-10	2.52E-09
METRNL	-0.108388733	0.013855	0.023877
METRN	-0.106895316	0.015228	0.026017
METT10D	-0.114877164	0.009073	0.016223
METT11D1	-0.023553565	0.593832	0.659748
METT5D1	0.098322108	0.025663	0.041799
METTL10	0.266387816	8.15E-10	4.59E-09
METTL11A	0.305303727	1.43E-12	1.22E-11
METTL11B	0.079942255	0.069882	0.103337
METTL12	0.138615187	0.001615	0.003305
METTL13	0.110220276	0.01232	0.021443
METTL14	-0.091308736	0.03832	0.060064
METTL1	0.2594669	2.28E-09	1.19E-08
METTL2A	0.411978621	1.61E-22	6.08E-21
METTL2B	0.341996726	1.41E-15	1.91E-14
METTL3	0.020189071	0.647602	0.707879
METTL4	0.175975589	5.94E-05	0.000156
METTL5	0.354470617	1.08E-16	1.71E-15
METTL6	0.221094429	4.02E-07	1.50E-06
METTL7A	-0.414962436	7.47E-23	2.98E-21
METTL7B	-0.066873461	0.129618	0.179071
METTL8	0.301931696	2.57E-12	2.10E-11
METTL9	0.287188262	3.10E-11	2.15E-10
MET	-0.077753966	0.077917	0.113929
MEX3A	0.179391812	4.23E-05	0.000114
MEX3B	-0.000247394	0.995531	0.997028
MEX3C	0.09437685	0.032248	0.05138
MEX3D	0.124103148	0.004796	0.009043
MFAP1	0.137216772	0.001801	0.00366
MFAP2	0.052270137	0.236363	0.303023
MFAP3L	-0.011577131	0.793246	0.834364
MFAP3	0.063273743	0.151613	0.205736
MFAP4	-0.422699628	9.77E-24	4.46E-22
MFAP5	0.19521053	8.11E-06	2.46E-05
MFF	0.264619112	1.06E-09	5.89E-09
MFGE8	-0.128966924	0.003369	0.006519
MFHAS1	-0.067917938	0.123722	0.171757

MFI2	0.127347565	0.003795	0.007273
MFN1	0.209844272	1.55E-06	5.28E-06
MFN2	-0.182689419	3.03E-05	8.40E-05
MFNG	-0.263949261	1.17E-09	6.45E-09
MFRP	-0.142118426	0.001222	0.002559
MFSD10	-0.143254545	0.001115	0.002352
MFSD11	-0.024888513	0.573077	0.641179
MFSD1	-0.131826935	0.002722	0.005359
MFSD2A	-0.323552095	5.15E-14	5.44E-13
MFSD2B	0.477960708	9.42E-31	1.17E-28
MFSD3	0.117792853	0.007451	0.013548
MFSD4	-0.37606661	9.56E-19	2.08E-17
MFSD5	0.189783068	1.45E-05	4.25E-05
MFSD6L	-0.128259925	0.003549	0.006836
MFSD6	-0.150166459	0.000629	0.001386
MFSD7	-0.262829816	1.39E-09	7.52E-09
MFSD8	-0.034960332	0.428541	0.503094
MFSD9	0.2061836	2.38E-06	7.84E-06
MGAM	-0.196034495	7.41E-06	2.26E-05
MGAT1	-0.180276307	3.87E-05	0.000105
MGAT2	0.458510222	3.90E-28	3.22E-26
MGAT3	-0.32037621	9.33E-14	9.48E-13
MGAT4A	-0.045395998	0.303844	0.375632
MGAT4B	-0.04597911	0.297668	0.368933
MGAT4C	0.039414602	0.372054	0.446912
MGAT5B	0.066167013	0.133728	0.184227
MGAT5	-0.012696995	0.773766	0.818208
MGA	-0.032176935	0.466231	0.540045
MGC12916	-0.058549561	0.184638	0.244406
MGC12982	-0.006879137	0.876242	0.903312
MGC14436	0.20745161	2.05E-06	6.84E-06
MGC15885	0.013706934	0.756319	0.803428
MGC16025	0.059507043	0.177548	0.236476
MGC16121	-0.093992295	0.03296	0.052381
MGC16142	-0.001068937	0.980694	0.985674
MGC16275	-0.147398769	0.000793	0.001717
MGC16384	-0.199809525	4.89E-06	1.53E-05
MGC16703	-0.26366491	1.23E-09	6.71E-09
MGC21881	-0.182922467	2.96E-05	8.22E-05
MGC23270	0.010611127	0.810155	0.848226
MGC23284	-0.229349495	1.42E-07	5.71E-07
MGC26647	0.012345195	0.779871	0.82285
MGC27382	-0.251678538	6.99E-09	3.42E-08
MGC2752	-0.12741018	0.003777	0.007241

MGC2889	0.035147628	0.42607	0.500746
MGC29506	0.016170012	0.714299	0.767687
MGC34034	0.171746773	8.96E-05	0.00023
MGC3771	-0.272111463	3.41E-10	2.03E-09
MGC42105	-0.159097564	0.000289	0.000678
MGC4473	0.108331265	0.013905	0.023954
MGC45800	0.100034559	0.023188	0.038131
MGC57346	-0.026031428	0.555585	0.625066
MGC70857	0.070852741	0.10827	0.152617
MGC72080	0.269885962	4.79E-10	2.79E-09
MGC87042	0.342960488	1.16E-15	1.59E-14
MGEA5	-0.217012839	6.62E-07	2.39E-06
MGLL	-0.346604504	5.54E-16	7.97E-15
MGMT	-0.039458708	0.371518	0.44643
MGP	-0.374294338	1.43E-18	3.03E-17
MGRN1	-0.413815959	1.01E-22	3.93E-21
MGST1	0.142640532	0.001171	0.002462
MGST2	-0.09852953	0.025352	0.041342
MGST3	0.213441807	1.02E-06	3.57E-06
MIA2	0.000954498	0.98276	0.987156
MIA3	-0.113186656	0.010151	0.018
MIAT	-0.040376319	0.36049	0.435245
MIA	-0.149151083	0.000685	0.001502
MIB1	0.195703639	7.69E-06	2.34E-05
MIB2	-0.294712116	8.86E-12	6.68E-11
MICAL1	-0.370838233	3.10E-18	6.25E-17
MICAL2	-0.157267212	0.00034	0.000786
MICAL3	0.026255159	0.552192	0.62206
MICALCL	-0.285623047	4.00E-11	2.73E-10
MICALL1	0.072920099	0.098331	0.140138
MICALL2	-0.260318067	2.01E-09	1.06E-08
MICA	-0.116357726	0.008214	0.014821
MICB	0.055185829	0.211199	0.274834
MID1IP1	-0.219750001	4.74E-07	1.76E-06
MID1	0.028142087	0.523982	0.595957
MID2	-0.263041016	1.35E-09	7.30E-09
MIDN	-0.10111161	0.021739	0.035955
MIER1	0.051438106	0.243917	0.311309
MIER2	-0.150344999	0.000619	0.001366
MIER3	0.106777939	0.015341	0.026186
MIF4GD	-0.101644874	0.021051	0.034936
MIF	0.26370846	1.22E-09	6.67E-09
MIIP	-0.099295921	0.024229	0.039657
MIMT1	-0.012640415	0.774747	0.819

MINA	0.290137956	1.90E-11	1.37E-10
MINK1	-0.192720767	1.06E-05	3.17E-05
MINPP1	0.290986467	1.65E-11	1.20E-10
MIOS	-0.008273481	0.851424	0.882598
MIOX	0.018983681	0.667341	0.72576
MIPEP	0.080977566	0.066324	0.09866
MIPOL1	0.223813443	2.87E-07	1.10E-06
MIP	0.104398423	0.017793	0.029962
MIR155HG	-0.131307583	0.002831	0.005552
MIR17HG	-0.139195852	0.001542	0.00317
MIS12	0.158238414	0.000312	0.000726
MITD1	0.154374221	0.000438	0.000994
MITF	-0.182358943	3.14E-05	8.66E-05
MIXL1	0.01449364	0.742813	0.7919
MKI67IP	0.458864005	3.51E-28	2.93E-26
MKI67	0.416673791	4.79E-23	1.96E-21
MKKS	0.215388498	8.05E-07	2.87E-06
MKL1	-0.08925619	0.042902	0.066567
MKL2	-0.180880877	3.64E-05	9.96E-05
MKLN1	-0.019603657	0.657159	0.716522
MKNK1	-0.285119098	4.34E-11	2.94E-10
MKNK2	-0.187183123	1.91E-05	5.47E-05
MKRN1	-0.13403511	0.002303	0.004593
MKRN2	0.05016303	0.255819	0.324386
MKRN3	0.129674457	0.003197	0.006212
MKS1	-0.130929031	0.002912	0.0057
MKX	0.084763987	0.054557	0.082788
MLANA	-0.107917149	0.014276	0.024546
MLC1	-0.159328865	0.000283	0.000665
MLEC	0.045371243	0.304108	0.375866
MLF1IP	0.425042911	5.22E-24	2.50E-22
MLF1	-0.012633775	0.774862	0.819078
MLF2	0.27763948	1.44E-10	9.04E-10
MLH1	0.032714906	0.458806	0.532618
MLH3	0.012845253	0.771198	0.815981
MLKL	0.109105848	0.013235	0.022894
MLL2	-0.151156853	0.000578	0.001282
MLL3	-0.217588397	6.17E-07	2.24E-06
MLL4	-0.139683474	0.001484	0.003061
MLL5	-0.230499031	1.23E-07	4.98E-07
MLLT10	-0.029592011	0.502817	0.575553
MLLT11	0.298739093	4.46E-12	3.50E-11
MLLT1	-0.308151703	8.63E-13	7.62E-12
MLLT3	-0.131125635	0.00287	0.00562

MLLT4	-0.329314225	1.72E-14	1.96E-13
MLLT6	-0.340342755	1.97E-15	2.61E-14
MLL	-0.207456309	2.05E-06	6.84E-06
MLNR	0.06064344	0.169398	0.226865
MLN	0.005994991	0.892044	0.915588
MLPH	-0.252071198	6.61E-09	3.24E-08
MLST8	0.025516693	0.563431	0.632582
MLXIPL	-0.236927827	5.29E-08	2.27E-07
MLXIP	-0.040552431	0.358398	0.432954
MLX	0.094991487	0.031135	0.049783
MLYCD	-0.204656457	2.83E-06	9.22E-06
MMAA	-0.085637582	0.052104	0.079391
MMAB	0.025491385	0.563818	0.632839
MMACHC	0.16597645	0.000154	0.000381
MMADHC	0.354923344	9.82E-17	1.56E-15
MMD2	0.035133686	0.426254	0.500909
MMD	0.24918451	9.94E-09	4.77E-08
MMEL1	-0.169159509	0.000115	0.000289
MME	-0.019563467	0.657817	0.717044
MMGT1	0.055831678	0.205896	0.268826
MMP10	0.111835303	0.011093	0.019504
MMP11	0.094069152	0.032817	0.052196
MMP12	0.261816695	1.61E-09	8.65E-09
MMP13	0.003505543	0.936746	0.950296
MMP14	0.167281978	0.000137	0.00034
MMP15	-0.174038207	7.18E-05	0.000187
MMP16	-0.012939939	0.769558	0.814678
MMP17	0.08850687	0.044686	0.069083
MMP19	-0.275308952	2.07E-10	1.27E-09
MMP1	0.235222177	6.63E-08	2.80E-07
MMP20	0.042855341	0.331735	0.40535
MMP21	-0.244356249	1.94E-08	8.90E-08
MMP23A	-0.081023357	0.066171	0.098446
MMP23B	-0.261481466	1.69E-09	9.07E-09
MMP24	-0.299213717	4.11E-12	3.25E-11
MMP25	-0.103992484	0.018244	0.030659
MMP26	0.05280988	0.231552	0.297676
MMP27	-0.076661884	0.082199	0.119456
MMP28	-0.295444749	7.82E-12	5.96E-11
MMP2	-0.110031389	0.012471	0.021674
MMP3	0.077283175	0.07974	0.11633
MMP7	-0.118514178	0.007092	0.012948
MMP8	0.07149067	0.105122	0.148598
MMP9	0.037403727	0.396962	0.471869

MMRN1	-0.248345519	1.12E-08	5.30E-08
MMRN2	-0.21625737	7.25E-07	2.60E-06
MMS19	0.005821665	0.895147	0.918019
MN1	-0.012219148	0.782062	0.824769
MNAT1	0.321487038	7.58E-14	7.83E-13
MND1	0.43908695	1.10E-25	6.88E-24
MNDA	-0.217909752	5.94E-07	2.17E-06
MNS1	0.039541023	0.370521	0.445311
MNT	-0.076252034	0.083854	0.121526
MNX1	-0.049500805	0.262156	0.331099
MOAP1	-0.268931623	5.55E-10	3.20E-09
MOB2	-0.235129959	6.71E-08	2.83E-07
MOBKL1A	-0.033561089	0.447263	0.52155
MOBKL1B	0.282657462	6.46E-11	4.27E-10
MOBKL2A	-0.113290278	0.010082	0.017887
MOBKL2B	-0.159838744	0.000271	0.000638
MOBKL2C	-0.375661862	1.05E-18	2.27E-17
MOBKL3	0.31268665	3.82E-13	3.56E-12
MOBP	-0.098563665	0.025301	0.041266
MOCOS	0.264542232	1.08E-09	5.95E-09
MOCS1	-0.428255044	2.19E-24	1.11E-22
MOCS2	-0.004734482	0.914645	0.933311
MOCS3	0.205012137	2.72E-06	8.87E-06
MOGAT1	-0.005166217	0.906895	0.92726
MOGAT2	-0.163197006	0.0002	0.000483
MOGAT3	0.104921404	0.017227	0.029072
MOGS	0.015060746	0.733125	0.783872
MOG	-0.014131539	0.74902	0.797327
MON1A	-0.080360431	0.068427	0.10144
MON1B	0.008736826	0.843208	0.875807
MON2	-0.096933925	0.027834	0.045001
MORC1	0.029661439	0.501815	0.574682
MORC2	0.035002398	0.427986	0.50256
MORC3	-0.23346162	8.35E-08	3.48E-07
MORC4	0.109158871	0.01319	0.022826
MORF4L1	0.134301887	0.002256	0.004506
MORF4L2	0.340890976	1.77E-15	2.36E-14
MORF4	0.097744284	0.026548	0.043118
MORN1	-0.264091185	1.15E-09	6.33E-09
MORN2	0.091598655	0.037707	0.059192
MORN3	-0.229801212	1.34E-07	5.41E-07
MORN4	-0.190748898	1.31E-05	3.86E-05
MORN5	-0.194827486	8.45E-06	2.56E-05
MOSC1	0.015219014	0.730429	0.781575

MOSC2	-0.291097903	1.62E-11	1.18E-10
MOSPD1	0.151426588	0.000565	0.001254
MOSPD2	-0.037981385	0.389706	0.464487
MOSPD3	-0.05169937	0.241527	0.308713
MOS	0.086291991	0.050327	0.076995
MOV10L1	0.021034576	0.633901	0.695451
MOV10	-0.042892482	0.331316	0.404887
MOXD1	-0.211262339	1.32E-06	4.53E-06
MPDU1	0.161639184	0.00023	0.00055
MPDZ	-0.185134064	2.36E-05	6.65E-05
MPEG1	-0.231993323	1.01E-07	4.16E-07
MPG	-0.053458937	0.225859	0.291144
MPHOSPH10	0.201806772	3.91E-06	1.25E-05
MPHOSPH6	0.277262075	1.53E-10	9.56E-10
MPHOSPH8	-0.232441347	9.54E-08	3.94E-07
MPHOSPH9	0.276239626	1.79E-10	1.11E-09
MPI	-0.04972681	0.259981	0.328859
MPL	-0.294484202	9.20E-12	6.93E-11
MPND	-0.280253415	9.50E-11	6.14E-10
MPO	-0.020952631	0.635224	0.696634
MPP1	-0.045204927	0.305885	0.377623
MPP2	-0.005059383	0.908812	0.928654
MPP3	-0.121010041	0.005967	0.011048
MPP4	-0.016638616	0.706397	0.76116
MPP5	0.144629453	0.000997	0.00212
MPP6	0.268658547	5.78E-10	3.33E-09
MPP7	-0.024526905	0.578665	0.645913
MPPE1	-0.130564183	0.002993	0.005844
MPPED1	-0.142695813	0.001166	0.002451
MPPED2	-0.161299356	0.000237	0.000566
MPRIP	-0.295170568	8.20E-12	6.22E-11
MPST	-0.013007442	0.76839	0.813786
MPV17L2	0.074895695	0.089525	0.128844
MPV17L	-0.104940933	0.017206	0.029044
MPV17	0.0595917	0.176931	0.235829
MPZL1	0.097430914	0.027039	0.04384
MPZL2	-0.226418751	2.07E-07	8.10E-07
MPZL3	-0.040029973	0.364629	0.43926
MPZ	-0.076095691	0.084493	0.122398
MR1	-0.212824223	1.09E-06	3.81E-06
MRAP2	-0.092230603	0.036401	0.057339
MRAP	-0.045574541	0.301944	0.37363
MRAS	-0.19107528	1.27E-05	3.73E-05
MRC1	-0.209803995	1.56E-06	5.30E-06

MRC2	-0.241551314	2.84E-08	1.27E-07
MRE11A	0.158739939	0.000299	0.000697
MREG	0.006262251	0.887262	0.911617
MRFAP1L1	-0.036107224	0.413541	0.488237
MRFAP1	0.080823586	0.066844	0.099344
MRGPRD	-0.023339896	0.597186	0.662829
MRGPRE	-0.146605394	0.000847	0.001824
MRGPRF	-0.086193472	0.050592	0.077346
MRGPRX1	0.001877827	0.966091	0.974034
MRGPRX2	-0.166238187	0.000151	0.000373
MRGPRX3	0.111037229	0.011685	0.020432
MRGPRX4	0.14382627	0.001064	0.002251
MRI1	-0.187262129	1.89E-05	5.43E-05
MRM1	0.083920425	0.057016	0.086062
MRO	0.070403625	0.110531	0.155422
MRP63	0.130294425	0.003053	0.005954
MRPL10	0.221958253	3.61E-07	1.36E-06
MRPL11	0.348046515	4.12E-16	6.03E-15
MRPL12	0.366811903	7.58E-18	1.44E-16
MRPL13	0.446202684	1.45E-26	1.01E-24
MRPL14	0.103246055	0.019099	0.031959
MRPL15	0.520535234	4.21E-37	1.79E-34
MRPL16	0.235912166	6.05E-08	2.57E-07
MRPL17	0.327423867	2.47E-14	2.74E-13
MRPL18	0.175770027	6.06E-05	0.000159
MRPL19	0.432940814	6.09E-25	3.38E-23
MRPL1	0.326736647	2.81E-14	3.11E-13
MRPL20	0.07384515	0.094125	0.134682
MRPL21	0.373774414	1.61E-18	3.37E-17
MRPL22	0.238303279	4.40E-08	1.91E-07
MRPL23	0.038118656	0.387994	0.462777
MRPL24	0.174934928	6.58E-05	0.000172
MRPL27	0.216727304	6.85E-07	2.47E-06
MRPL28	0.142420843	0.001192	0.002502
MRPL2	0.200907407	4.32E-06	1.37E-05
MRPL30	0.392856298	1.88E-20	5.22E-19
MRPL32	0.238731717	4.16E-08	1.81E-07
MRPL33	0.180331442	3.85E-05	0.000105
MRPL34	0.099708205	0.023643	0.038783
MRPL35	0.37185623	2.47E-18	5.04E-17
MRPL36	0.229760446	1.35E-07	5.44E-07
MRPL37	0.348318397	3.90E-16	5.73E-15
MRPL38	0.06118212	0.165634	0.222419
MRPL39	0.215746806	7.71E-07	2.75E-06

MRPL3	0.471795963	6.63E-30	7.13E-28
MRPL40	0.094857447	0.031375	0.050142
MRPL41	0.070933768	0.107866	0.15209
MRPL42P5	-0.179185237	4.32E-05	0.000117
MRPL42	0.534579216	2.11E-39	1.57E-36
MRPL43	0.145916837	0.000897	0.001921
MRPL44	0.361884802	2.22E-17	3.91E-16
MRPL45	0.340441571	1.93E-15	2.57E-14
MRPL46	0.188072555	1.74E-05	5.02E-05
MRPL47	0.350953516	2.26E-16	3.40E-15
MRPL48	0.323684282	5.02E-14	5.32E-13
MRPL49	0.147325186	0.000798	0.001727
MRPL4	0.092181449	0.036501	0.057466
MRPL50	0.288470741	2.51E-11	1.76E-10
MRPL51	0.417605014	3.75E-23	1.58E-21
MRPL52	0.363534035	1.55E-17	2.80E-16
MRPL53	0.131615485	0.002766	0.005434
MRPL54	-0.00987471	0.823105	0.859168
MRPL55	-0.017626313	0.689846	0.746179
MRPL9	0.323461282	5.23E-14	5.52E-13
MRPS10	0.410027294	2.66E-22	9.71E-21
MRPS11	0.282543568	6.58E-11	4.35E-10
MRPS12	0.332812655	8.73E-15	1.04E-13
MRPS14	0.231069412	1.14E-07	4.65E-07
MRPS15	0.282959495	6.16E-11	4.08E-10
MRPS16	0.412941918	1.26E-22	4.84E-21
MRPS17	0.396183807	8.40E-21	2.47E-19
MRPS18A	0.182372311	3.13E-05	8.65E-05
MRPS18B	0.135049507	0.002131	0.004279
MRPS18C	0.217544233	6.21E-07	2.26E-06
MRPS21	0.069437407	0.115521	0.161742
MRPS22	0.27482701	2.24E-10	1.37E-09
MRPS23	0.288381169	2.55E-11	1.78E-10
MRPS24	0.304505998	1.64E-12	1.39E-11
MRPS25	-0.22251601	3.37E-07	1.28E-06
MRPS26	0.080182724	0.069042	0.102262
MRPS27	0.136407623	0.001918	0.003878
MRPS28	0.364533203	1.25E-17	2.29E-16
MRPS2	0.273553532	2.73E-10	1.64E-09
MRPS30	0.352878327	1.51E-16	2.34E-15
MRPS31	0.046022077	0.297216	0.368512
MRPS33	0.261964667	1.58E-09	8.49E-09
MRPS34	0.110613527	0.012011	0.020943
MRPS35	0.493256399	6.23E-33	1.15E-30

MRPS36	0.04060064	0.357826	0.432342
MRPS5	0.264938311	1.01E-09	5.63E-09
MRPS6	0.014956236	0.734907	0.785399
MRPS7	0.238673651	4.19E-08	1.82E-07
MRPS9	0.193228006	1.01E-05	3.01E-05
MRRF	0.107710315	0.014465	0.024832
MRS2P2	0.061434886	0.16389	0.220328
MRS2	0.164503943	0.000177	0.000433
MRTO4	0.380103359	3.79E-19	8.82E-18
MRVI1	-0.210592281	1.42E-06	4.87E-06
MS4A10	-0.08166344	0.06405	0.09559
MS4A12	0.037608024	0.394387	0.46931
MS4A13	0.042446421	0.336374	0.410341
MS4A14	-0.28432278	4.94E-11	3.32E-10
MS4A15	-0.363116348	1.70E-17	3.06E-16
MS4A1	-0.212963811	1.08E-06	3.75E-06
MS4A2	-0.394686902	1.21E-20	3.45E-19
MS4A3	-0.053876771	0.222247	0.287099
MS4A4A	-0.080417104	0.068232	0.101181
MS4A5	0.11130207	0.011486	0.020135
MS4A6A	-0.141023844	0.001334	0.002772
MS4A6E	-0.03151362	0.475476	0.549195
MS4A7	-0.242819546	2.39E-08	1.08E-07
MS4A8B	-0.234245578	7.53E-08	3.16E-07
MSC	0.032605841	0.460306	0.534266
MSGN1	-0.014387952	0.744623	0.793522
MSH2	0.30428759	1.71E-12	1.44E-11
MSH3	0.018063013	0.682575	0.739514
MSH4	-0.056520117	0.200351	0.262494
MSH5	-0.093300545	0.034277	0.05428
MSH6	0.27762804	1.44E-10	9.05E-10
MSI1	0.106329786	0.015779	0.026858
MSI2	0.100518677	0.022527	0.037132
MSL1	0.05685224	0.197715	0.259466
MSL2	-0.188864455	1.60E-05	4.65E-05
MSL3L2	0.11352265	0.009928	0.017647
MSL3	-0.086331009	0.050223	0.076865
MSLNL	0.005142742	0.907316	0.927549
MSLN	-0.069917322	0.113021	0.158642
MSMB	0.112689913	0.010489	0.018535
MSMP	-0.019730424	0.655084	0.714615
MSN	-0.177310226	5.21E-05	0.000139
MSR1	-0.092453154	0.03595	0.05671
MSRA	-0.218452029	5.56E-07	2.04E-06

MSRB2	0.030672212	0.487347	0.560702
MSRB3	-0.159826787	0.000271	0.000638
MST1P2	-0.33335943	7.84E-15	9.41E-14
MST1P9	-0.312623606	3.87E-13	3.60E-12
MST1R	-0.141740713	0.001259	0.002633
MST1	-0.197530768	6.29E-06	1.94E-05
MST4	0.120557697	0.006158	0.011369
MSTN	-0.259079715	2.41E-09	1.26E-08
MSTO1	0.027623469	0.531661	0.603178
MSTO2P	-0.217841558	5.99E-07	2.18E-06
MSX1	0.117375092	0.007667	0.013912
MSX2P1	-0.166272493	0.00015	0.000372
MSX2	0.000408262	0.992626	0.994633
MT1A	0.076464133	0.082994	0.120472
MT1B	0.134012499	0.002307	0.004599
MT1DP	-0.064152533	0.145998	0.199107
MT1E	-0.06552885	0.137525	0.188847
MT1F	-0.06208396	0.159474	0.215129
MT1G	0.169219656	0.000114	0.000287
MT1H	0.262459379	1.47E-09	7.92E-09
MT1IP	-0.113254088	0.010106	0.017927
MT1L	0.057196733	0.195007	0.256283
MT1M	-0.065593899	0.137135	0.188388
MT1X	0.178622561	4.57E-05	0.000123
MT2A	0.149368648	0.000672	0.001476
MT3	-0.091213472	0.038523	0.060363
MTA1	0.063821188	0.148096	0.201549
MTA2	0.244107871	2.01E-08	9.19E-08
MTA3	0.185703946	2.23E-05	6.30E-05
MTAP	0.10135284	0.021426	0.035496
MTBP	0.364993117	1.13E-17	2.09E-16
MTCH1	0.028037686	0.525523	0.597506
MTCH2	0.415461747	6.56E-23	2.65E-21
MTCP1NB	0.000878644	0.98413	0.988283
MTCP1	-0.139190659	0.001543	0.003171
MTDH	0.378975826	4.92E-19	1.12E-17
MTERFD1	0.369735014	3.97E-18	7.88E-17
MTERFD2	-0.053379463	0.226551	0.291941
MTERFD3	-0.104986994	0.017157	0.028973
MTERF	0.283537891	5.61E-11	3.74E-10
MTF1	-0.152930238	0.000497	0.001113
MTF2	0.083122645	0.059425	0.089342
MTFMT	0.020126757	0.648616	0.708716
MTFR1	0.481350661	3.16E-31	4.40E-29

MTG1	0.086624528	0.049444	0.075737
MTHFD1L	0.281601986	7.66E-11	5.01E-10
MTHFD1	0.395023348	1.11E-20	3.20E-19
MTHFD2L	0.087337682	0.047594	0.073093
MTHFD2	0.549524444	5.72E-42	7.14E-39
MTHFR	-0.2349561	6.86E-08	2.90E-07
MTHFSD	-0.158854958	0.000295	0.000691
MTHFS	0.161436252	0.000234	0.000559
MTIF2	0.334480759	6.30E-15	7.72E-14
MTIF3	-0.048539107	0.27155	0.341497
MTL5	0.387909042	6.13E-20	1.58E-18
MTM1	-0.22177314	3.69E-07	1.39E-06
MTMR10	-0.321281985	7.88E-14	8.10E-13
MTMR11	0.131850149	0.002718	0.005351
MTMR12	-0.156539009	0.000363	0.000833
MTMR14	-0.185809845	2.20E-05	6.24E-05
MTMR15	-0.117665473	0.007516	0.013658
MTMR1	-0.146147505	0.00088	0.001889
MTMR2	0.351005221	2.23E-16	3.37E-15
MTMR3	-0.344066838	9.30E-16	1.29E-14
MTMR4	-0.023259083	0.598457	0.663954
MTMR6	0.031316264	0.478246	0.552075
MTMR7	0.096325216	0.028835	0.046457
MTMR8	-0.229023602	1.48E-07	5.93E-07
MTMR9L	-0.263211133	1.31E-09	7.13E-09
MTMR9	-0.092040382	0.03679	0.057875
MTNR1A	0.017715653	0.688356	0.74493
MTNR1B	0.036367261	0.410183	0.484817
MTO1	-0.005954962	0.89276	0.916169
MTOR	-0.043507906	0.324418	0.397461
MTP18	0.300289294	3.42E-12	2.73E-11
MTPAP	0.349151193	3.28E-16	4.88E-15
MTRF1L	0.259963648	2.12E-09	1.11E-08
MTRF1	-0.043244671	0.327357	0.400662
MTRR	-0.145668915	0.000915	0.001958
MTR	-0.309909305	6.30E-13	5.70E-12
MTSS1L	-0.279466203	1.08E-10	6.89E-10
MTSS1	-0.206608288	2.26E-06	7.49E-06
MTTP	-0.038417061	0.384287	0.458931
MTUS1	-0.210193571	1.49E-06	5.08E-06
MTUS2	-0.099055356	0.024577	0.040183
MTVR2	-0.228232247	1.64E-07	6.52E-07
MTX1	0.248947249	1.03E-08	4.91E-08
MTX2	0.146917884	0.000825	0.001782

MTX3	-0.235509645	6.38E-08	2.70E-07
MUC12	0.000861302	0.984443	0.9883
MUC13	0.21297646	1.07E-06	3.75E-06
MUC15	-0.280391626	9.29E-11	6.01E-10
MUC16	0.076936468	0.081105	0.118063
MUC17	-0.016092482	0.71561	0.768598
MUC1	-0.281050904	8.36E-11	5.45E-10
MUC20	-0.180817081	3.67E-05	0.0001
MUC21	-0.241933825	2.70E-08	1.21E-07
MUC2	0.139226888	0.001538	0.003164
MUC4	0.040671581	0.356986	0.431509
MUC5B	0.036747796	0.405299	0.480066
MUC6	-0.022571573	0.609315	0.673729
MUC7	-0.042989846	0.330218	0.40367
MUCL1	0.160269147	0.00026	0.000615
MUDENG	0.293466239	1.09E-11	8.12E-11
MUL1	-0.046018474	0.297254	0.368512
MUM1L1	-0.076035062	0.084742	0.122705
MUM1	-0.302925412	2.16E-12	1.79E-11
MURC	0.200517878	4.52E-06	1.43E-05
MUS81	0.033086095	0.453722	0.527828
MUSK	-0.363964672	1.41E-17	2.57E-16
MUSTN1	-0.434328075	4.15E-25	2.37E-23
MUTED	0.187891334	1.77E-05	5.11E-05
MUTYH	-0.109785773	0.01267	0.02199
MUT	0.023023028	0.602175	0.667347
MVD	-0.049918495	0.258146	0.326902
MVK	-0.074369848	0.091805	0.131701
MVP	-0.310380649	5.79E-13	5.27E-12
MX1	-0.190102748	1.40E-05	4.12E-05
MX2	-0.127776134	0.003678	0.007062
MXD1	0.107565606	0.014598	0.025052
MXD3	0.069360307	0.115926	0.16223
MXD4	-0.270830275	4.15E-10	2.44E-09
MXI1	0.02417932	0.584061	0.650845
MXRA5	0.103924671	0.01832	0.030774
MXRA7	-0.049042502	0.266605	0.336123
MXRA8	-0.092714224	0.035427	0.055965
MYADML2	0.06355473	0.1498	0.20359
MYADML	0.055791914	0.20622	0.269178
MYADM	-0.098230517	0.025802	0.042011
MYBBP1A	0.039212401	0.374514	0.449407
MYBL1	0.212620519	1.12E-06	3.89E-06
MYBL2	0.448704841	7.04E-27	5.06E-25

MYBPC1	-0.013926813	0.752536	0.800345
MYBPC2	-0.263982721	1.17E-09	6.43E-09
MYBPC3	-0.226345227	2.08E-07	8.16E-07
MYBPHL	-0.361384405	2.47E-17	4.34E-16
MYBPH	-0.161979654	0.000223	0.000535
MYB	0.039999383	0.364995	0.43965
MYCBP2	-0.232317688	9.69E-08	4.00E-07
MYCBPAP	-0.277335134	1.51E-10	9.46E-10
MYCBP	0.166572528	0.000146	0.000362
MYCL1	-0.078037729	0.076835	0.112577
MYCNOS	0.152169468	0.00053	0.001183
MYCN	0.088334742	0.045104	0.069676
MYCT1	-0.154654254	0.000428	0.000972
MYC	0.217652996	6.12E-07	2.23E-06
MYD88	-0.092470104	0.035916	0.056665
MYEF2	-0.177778575	4.97E-05	0.000133
MYEOV2	0.165790146	0.000157	0.000387
MYEOV	0.378277912	5.77E-19	1.30E-17
MYF5	-0.016133897	0.71491	0.768011
MYF6	0.075718243	0.08605	0.124384
MYH10	-0.248550732	1.09E-08	5.17E-08
MYH11	-0.348152655	4.03E-16	5.92E-15
MYH13	0.108702087	0.013581	0.023443
MYH14	-0.266315528	8.24E-10	4.64E-09
MYH15	-0.085291141	0.053066	0.080715
MYH16	0.314068395	2.98E-13	2.82E-12
MYH1	-0.302961314	2.15E-12	1.78E-11
MYH2	-0.270194627	4.57E-10	2.67E-09
MYH3	-0.291653915	1.48E-11	1.08E-10
MYH4	-0.099848778	0.023446	0.038498
MYH6	0.053666823	0.224057	0.289206
MYH7B	-0.309944458	6.26E-13	5.67E-12
MYH7	-0.044438579	0.314166	0.38641
MYH8	-0.030162296	0.494617	0.567917
MYH9	-0.121484742	0.005772	0.010712
MYL10	0.08417625	0.056261	0.085051
MYL12A	0.057530542	0.19241	0.253352
MYL12B	0.08981831	0.041603	0.064763
MYL1	0.038726372	0.380468	0.455104
MYL2	-0.009706826	0.826064	0.861553
MYL3	-0.245163504	1.74E-08	8.01E-08
MYL4	-0.026205444	0.552945	0.622803
MYL5	-0.302666128	2.26E-12	1.87E-11
MYL6B	0.33854884	2.82E-15	3.66E-14

MYL6	0.169691133	0.000109	0.000276
MYL7	0.05532943	0.210011	0.273502
MYL9	-0.176987977	5.38E-05	0.000143
MYLIP	-0.291465214	1.53E-11	1.11E-10
MYLK2	0.164043072	0.000185	0.00045
MYLK3	-0.105882385	0.016227	0.027542
MYLK4	-0.274884601	2.22E-10	1.35E-09
MYLK	-0.16132631	0.000237	0.000564
MYLPF	-0.137099666	0.001818	0.003691
MYNN	0.208737105	1.77E-06	5.97E-06
MYO10	-0.01119421	0.799938	0.839811
MYO15A	-0.346680055	5.46E-16	7.87E-15
MYO15B	-0.325407231	3.62E-14	3.94E-13
MYO16	0.01119839	0.799865	0.839778
MYO18A	-0.224277715	2.70E-07	1.04E-06
MYO18B	0.040217373	0.362386	0.436979
MYO19	0.218537647	5.50E-07	2.02E-06
MYO1A	-0.109843765	0.012622	0.021916
MYO1B	-0.113025972	0.010259	0.018174
MYO1C	-0.138435763	0.001637	0.00335
MYO1D	-0.217381994	6.33E-07	2.30E-06
MYO1E	0.175271985	6.36E-05	0.000167
MYO1F	-0.302163711	2.47E-12	2.02E-11
MYO1G	-0.234913868	6.90E-08	2.91E-07
MYO1H	-0.115392764	0.008765	0.015714
MYO3A	-0.168064443	0.000127	0.000318
MYO3B	-0.059505674	0.177558	0.236476
MYO5A	-0.097136219	0.027508	0.044525
MYO5B	-0.015746927	0.72146	0.773717
MYO5C	-0.199226748	5.22E-06	1.63E-05
MYO6	-0.234044022	7.74E-08	3.23E-07
MYO7A	0.014882365	0.736168	0.786399
MYO7B	-0.12772712	0.003691	0.007086
MYO9A	-0.242308486	2.57E-08	1.15E-07
MYO9B	-0.279506315	1.07E-10	6.85E-10
MYOCD	-0.333593394	7.49E-15	9.02E-14
MYOC	-0.431475878	9.12E-25	4.90E-23
MYOD1	0.045268901	0.305201	0.376957
MYOF	-0.124523038	0.004654	0.008795
MYOG	-0.079586519	0.07114	0.105027
MYOM1	-0.136144133	0.001958	0.003955
MYOM2	-0.299718357	3.77E-12	3.00E-11
MYOM3	0.043155825	0.328353	0.401733
MYOT	-0.09502656	0.031073	0.049695

MYOZ1	-0.340494755	1.91E-15	2.54E-14
MYOZ2	-0.098351263	0.025619	0.041744
MYOZ3	-0.23053223	1.22E-07	4.97E-07
MYPN	0.098590464	0.025261	0.041204
MYPOP	0.063722547	0.148725	0.202267
MYRIP	-0.23893311	4.05E-08	1.77E-07
MYSM1	-0.146552356	0.000851	0.001831
MYST1	-0.214098519	9.40E-07	3.32E-06
MYST2	-0.183413197	2.82E-05	7.83E-05
MYST3	0.013723937	0.756026	0.803169
MYST4	-0.188105418	1.73E-05	5.01E-05
MYT1L	0.009829559	0.8239	0.859819
MYT1	-0.028765472	0.514826	0.587181
MZF1	-0.336601305	4.15E-15	5.22E-14
N4BP1	-0.317391441	1.62E-13	1.59E-12
N4BP2L1	-0.464965907	5.51E-29	5.19E-27
N4BP2L2	-0.283636855	5.52E-11	3.68E-10
N4BP2	0.157231785	0.000341	0.000788
N4BP3	-0.22042622	4.36E-07	1.63E-06
N6AMT1	-0.161438439	0.000234	0.000559
N6AMT2	0.104878781	0.017272	0.029141
NAA10	0.272521833	3.20E-10	1.91E-09
NAA11	0.081911811	0.063243	0.094512
NAA15	0.442971892	3.66E-26	2.43E-24
NAA16	-0.216432185	7.10E-07	2.55E-06
NAA20	0.300357381	3.38E-12	2.70E-11
NAA25	0.346568352	5.58E-16	8.03E-15
NAA30	0.331657128	1.09E-14	1.28E-13
NAA35	0.314689644	2.66E-13	2.54E-12
NAA38	0.240348559	3.35E-08	1.48E-07
NAA40	-0.047051232	0.286533	0.357504
NAA50	0.393070551	1.79E-20	4.97E-19
NAAA	-0.13895622	0.001572	0.003224
NAALAD2	-0.255653845	3.96E-09	2.00E-08
NAALADL1	-0.233716522	8.07E-08	3.37E-07
NAALADL2	-0.125907309	0.004213	0.008011
NAB1	0.092403934	0.036049	0.056848
NAB2	-0.165984129	0.000154	0.000381
NACA2	0.166794788	0.000143	0.000355
NACAD	-0.169665475	0.000109	0.000276
NACAP1	0.145882237	0.000899	0.001926
NACA	0.235180909	6.66E-08	2.81E-07
NACC1	0.066584097	0.131289	0.18113
NACC2	-0.031258078	0.479065	0.552924

NADK	-0.046402257	0.293239	0.364507
NADSYN1	-0.187011477	1.94E-05	5.56E-05
NAE1	0.273774505	2.63E-10	1.59E-09
NAF1	0.183282943	2.85E-05	7.93E-05
NAGA	-0.177246525	5.24E-05	0.000139
NAGK	-0.154241979	0.000443	0.001004
NAGLU	-0.165254648	0.000165	0.000405
NAGPA	-0.143998516	0.001049	0.002223
NAGS	-0.100074913	0.023132	0.038048
NAIF1	0.108451014	0.0138	0.023795
NAIP	-0.275568379	1.99E-10	1.22E-09
NALCN	-0.213544923	1.00E-06	3.53E-06
NAMPT	0.396408403	7.95E-21	2.35E-19
NANOG	-0.156722754	0.000357	0.000822
NANOS1	-0.04169356	0.345022	0.419228
NANOS2	0.016162022	0.714434	0.767687
NANOS3	0.020232998	0.646887	0.707213
NANP	0.208121312	1.90E-06	6.38E-06
NANS	0.163122804	0.000201	0.000486
NAP1L1	0.109390612	0.012995	0.022519
NAP1L2	-0.261374768	1.72E-09	9.20E-09
NAP1L3	-0.202034801	3.81E-06	1.22E-05
NAP1L4	0.001841258	0.966751	0.974503
NAP1L5	-0.249516942	9.48E-09	4.56E-08
NAP1L6	-0.040316123	0.361208	0.435927
NAPA	-0.359823349	3.46E-17	5.97E-16
NAPB	-0.208981754	1.72E-06	5.81E-06
NAPEPLD	-0.03614383	0.413067	0.487764
NAPG	0.038561451	0.382501	0.457077
NAPRT1	-0.128482787	0.003492	0.006735
NAPSA	-0.340255773	2.01E-15	2.66E-14
NAPSB	-0.399003276	4.21E-21	1.30E-19
NARFL	-0.148171852	0.000743	0.00162
NARF	0.069595408	0.114693	0.160729
NARG2	0.010636586	0.809708	0.847888
NARS2	0.247578265	1.24E-08	5.86E-08
NARS	0.284080255	5.14E-11	3.44E-10
NASP	-0.014718137	0.738973	0.788758
NAT10	0.140608713	0.001379	0.00286
NAT14	-0.152660926	0.000508	0.001138
NAT15	-0.266552036	7.95E-10	4.49E-09
NAT1	0.018526252	0.674893	0.732622
NAT2	0.002013547	0.963642	0.971993
NAT6	-0.25886847	2.49E-09	1.29E-08

NAT8B	0.004047831	0.926986	0.942932
NAT8L	0.163816502	0.000189	0.000458
NAT8	-0.077366416	0.079416	0.115925
NAT9	0.061357197	0.164425	0.220928
NAV1	0.130975066	0.002902	0.005682
NAV2	-0.065103023	0.140106	0.191915
NAV3	-0.042260188	0.3385	0.412582
NBAS	-0.04452773	0.313195	0.385287
NBEAL1	-0.234367928	7.41E-08	3.11E-07
NBEAL2	-0.250199566	8.62E-09	4.17E-08
NBEA	-0.204174817	2.99E-06	9.70E-06
NBL1	-0.055414732	0.209308	0.272657
NBLA00301	-0.150261875	0.000624	0.001376
NBN	0.362699464	1.86E-17	3.32E-16
NBPF10	-0.215687483	7.77E-07	2.77E-06
NBPF14	-0.236692388	5.46E-08	2.33E-07
NBPF15	-0.112570822	0.010571	0.018672
NBPF16	-0.105072089	0.017066	0.028843
NBPF1	-0.238638756	4.21E-08	1.83E-07
NBPF22P	0.105332903	0.016792	0.028417
NBPF3	-0.25399149	5.03E-09	2.51E-08
NBPF4	0.168650718	0.00012	0.000302
NBPF6	0.131646327	0.00276	0.005424
NBPF7	-0.160285167	0.00026	0.000615
NBPF9	-0.195341876	7.99E-06	2.43E-05
NBR1	-0.161083227	0.000242	0.000576
NBR2	-0.184179881	2.60E-05	7.29E-05
NCALD	-0.305204883	1.45E-12	1.24E-11
NCAM1	-0.169692314	0.000109	0.000276
NCAM2	-0.231863878	1.03E-07	4.22E-07
NCAN	-0.04579997	0.299556	0.371021
NCAPD2	0.393505186	1.61E-20	4.49E-19
NCAPD3	0.253033934	5.77E-09	2.85E-08
NCAPG2	0.411458166	1.85E-22	6.88E-21
NCAPG	0.530877898	8.74E-39	5.46E-36
NCAPH2	-0.108932944	0.013382	0.02312
NCAPH	0.48378139	1.44E-31	2.11E-29
NCBP1	0.319405715	1.12E-13	1.13E-12
NCBP2	0.189633403	1.48E-05	4.31E-05
NCCRP1	-0.005307538	0.90436	0.925047
NCDN	-0.292863761	1.21E-11	8.93E-11
NCEH1	0.012572436	0.775926	0.819663
NCF1B	-0.167276754	0.000137	0.00034
NCF1C	-0.170860864	9.75E-05	0.000249

NCF1	-0.168714961	0.00012	0.0003
NCF2	-0.162684416	0.000209	0.000505
NCF4	-0.293990981	1.00E-11	7.48E-11
NCK1	0.204079153	3.02E-06	9.80E-06
NCK2	-0.059894007	0.174741	0.233159
NCKAP1L	-0.228532149	1.58E-07	6.29E-07
NCKAP1	0.291405648	1.54E-11	1.12E-10
NCKAP5L	-0.301757437	2.65E-12	2.16E-11
NCKAP5	-0.150160595	0.000629	0.001386
NCKIPSD	-0.058040833	0.188488	0.248927
NCLN	0.081591093	0.064287	0.095886
NCL	0.247753267	1.21E-08	5.73E-08
NCOA1	-0.142814398	0.001155	0.00243
NCOA2	-0.092768103	0.03532	0.055818
NCOA3	0.060521141	0.170262	0.227903
NCOA4	0.035114047	0.426513	0.50114
NCOA5	0.040856283	0.354805	0.429392
NCOA6	0.038016768	0.389264	0.464126
NCOA7	-0.159798525	0.000272	0.00064
NCOR1	-0.052125448	0.237665	0.304321
NCOR2	-0.161581962	0.000231	0.000553
NCR1	0.054960338	0.213073	0.276948
NCR2	0.009536592	0.829067	0.863948
NCR3	-0.188536907	1.66E-05	4.80E-05
NCRNA00028	-0.017707617	0.68849	0.745035
NCRNA00029	0.005402285	0.902661	0.92384
NCRNA00032	-0.162090504	0.000221	0.00053
NCRNA00051	0.015321492	0.728685	0.780168
NCRNA00052	0.132872126	0.002516	0.004982
NCRNA00081	0.016779414	0.704029	0.759138
NCRNA00085	-0.311283341	4.93E-13	4.51E-12
NCRNA00086	-0.175191462	6.41E-05	0.000168
NCRNA00087	-0.325695607	3.43E-14	3.75E-13
NCRNA00092	-0.34528531	7.26E-16	1.02E-14
NCRNA00093	-0.109457256	0.01294	0.02243
NCRNA00094	-0.159842574	0.00027	0.000638
NCRNA00095	0.015292589	0.729177	0.780569
NCRNA00099	-0.018348583	0.677835	0.735257
NCRNA00105	-0.257981751	2.83E-09	1.46E-08
NCRNA00107	-0.243254659	2.26E-08	1.02E-07
NCRNA00110	-0.160511083	0.000255	0.000603
NCRNA00111	-0.074463822	0.091394	0.131243
NCRNA00112	0.020779039	0.638029	0.699288
NCRNA00113	-0.13441186	0.002237	0.004472

NCRNA00114	-0.084731909	0.054649	0.082909
NCRNA00115	-0.212275441	1.17E-06	4.05E-06
NCRNA00116	0.087801391	0.046422	0.071519
NCRNA00119	0.100325668	0.022788	0.037526
NCRNA00120	-0.029840667	0.499233	0.572237
NCRNA00152	0.265960413	8.69E-10	4.88E-09
NCRNA00157	0.004342223	0.921693	0.938741
NCRNA00158	-0.010368676	0.814413	0.852053
NCRNA00159	-0.008677645	0.844256	0.876669
NCRNA00160	-0.033973157	0.441702	0.516029
NCRNA00161	-0.120985924	0.005977	0.011065
NCRNA00162	0.057352619	0.193791	0.254864
NCRNA00164	-0.019350273	0.661313	0.720199
NCRNA00167	-0.045418941	0.303599	0.375376
NCRNA00169	-0.147877269	0.000762	0.001656
NCRNA00171	-0.200698987	4.43E-06	1.40E-05
NCRNA00173	-0.275665399	1.96E-10	1.21E-09
NCRNA00174	-0.354254886	1.13E-16	1.78E-15
NCRNA00175	-0.118416528	0.00714	0.01303
NCRNA00176	-0.085189638	0.05335	0.081111
NCRNA00181	-0.166196785	0.000151	0.000374
NCRNA00182	-0.311545482	4.70E-13	4.31E-12
NCRNA00183	-0.085632839	0.052117	0.079405
NCRNA00185	0.053423048	0.226171	0.29149
NCRNA00188	0.187881197	1.77E-05	5.12E-05
NCRNA00189	0.086140526	0.050734	0.07754
NCRNA00200	0.037588208	0.394636	0.469504
NCRNA00201	-0.336833373	3.96E-15	5.01E-14
NCRNA00202	-0.289373875	2.16E-11	1.54E-10
NCRNA00203	-0.166929393	0.000141	0.000351
NCRNA00204B	-0.241501971	2.86E-08	1.28E-07
NCRNA00207	0.064433015	0.14424	0.197025
NCRNA00219	-0.065206441	0.139476	0.19121
NCRNA00230B	0.125395969	0.004372	0.008295
NCRNA00235	-0.044375448	0.314855	0.387138
NCS1	0.073467683	0.095824	0.136877
NCSTN	0.096835581	0.027994	0.045244
NDC80	0.471264813	7.83E-30	8.28E-28
NDE1	0.093887105	0.033158	0.052664
NDEL1	-0.016241941	0.713084	0.766586
NDFIP1	-0.055518606	0.208455	0.27174
NDFIP2	0.219151445	5.10E-07	1.88E-06
NDNL2	0.030265709	0.493138	0.566388
NDN	-0.283900349	5.29E-11	3.54E-10

NDOR1	-0.098709598	0.025084	0.040939
NDP	0.036630911	0.406795	0.481553
NDRG1	0.119179825	0.006775	0.012419
NDRG2	-0.438948448	1.14E-25	7.10E-24
NDRG3	0.11089911	0.01179	0.020588
NDRG4	-0.036099446	0.413641	0.488327
NDST1	-0.19445855	8.80E-06	2.66E-05
NDST2	-0.361378027	2.48E-17	4.34E-16
NDST3	-0.014934304	0.735281	0.785715
NDST4	0.039371903	0.372572	0.447373
NDUFA10	0.048801208	0.268968	0.338632
NDUFA11	0.071915269	0.103067	0.14607
NDUFA12	0.355557524	8.59E-17	1.38E-15
NDUFA13	0.030444809	0.490582	0.563841
NDUFA1	0.14031318	0.001412	0.002922
NDUFA2	-0.035485838	0.421629	0.496262
NDUFA3	0.055609603	0.207709	0.270891
NDUFA4L2	0.04495717	0.308547	0.380458
NDUFA4	0.169233282	0.000114	0.000287
NDUFA5	0.116187234	0.008309	0.014974
NDUFA6	0.078910038	0.073584	0.108218
NDUFA7	0.057315962	0.194077	0.255194
NDUFA8	0.208113839	1.90E-06	6.38E-06
NDUFA9	0.419558381	2.25E-23	9.76E-22
NDUFAB1	0.278918275	1.17E-10	7.48E-10
NDUFAF1	-0.091687098	0.037522	0.05892
NDUFAF2	0.20554088	2.56E-06	8.40E-06
NDUFAF3	-0.057422966	0.193244	0.254317
NDUFAF4	0.294647671	8.95E-12	6.75E-11
NDUFB10	0.069770232	0.113782	0.159599
NDUFB11	0.152612337	0.00051	0.001142
NDUFB1	0.149382104	0.000672	0.001474
NDUFB2	0.097389836	0.027104	0.043931
NDUFB3	0.257308393	3.12E-09	1.60E-08
NDUFB4	0.2081366	1.90E-06	6.37E-06
NDUFB5	0.238954662	4.04E-08	1.76E-07
NDUFB6	0.227439913	1.81E-07	7.17E-07
NDUFB7	0.047163401	0.285384	0.356294
NDUFB8	0.18351713	2.79E-05	7.76E-05
NDUFB9	0.285813592	3.88E-11	2.65E-10
NDUFC1	0.122358128	0.005428	0.010132
NDUFC2	0.188937659	1.59E-05	4.61E-05
NDUFS1	0.334211512	6.64E-15	8.10E-14
NDUFS2	0.167786186	0.00013	0.000326

NDUFS3	0.124798386	0.004563	0.00863
NDUFS4	0.154926805	0.000418	0.00095
NDUFS5	0.121747703	0.005667	0.010538
NDUFS6	0.161137891	0.000241	0.000573
NDUFS7	-0.11359616	0.00988	0.017568
NDUFS8	0.116280218	0.008257	0.014892
NDUFV1	0.005041392	0.909135	0.928838
NDUFV2	0.239397988	3.80E-08	1.66E-07
NDUFV3	0.020915192	0.635828	0.697144
NEAT1	-0.362217833	2.07E-17	3.66E-16
NEBL	-0.084448778	0.055465	0.083977
NEB	0.061401445	0.16412	0.220578
NECAB1	-0.222860255	3.23E-07	1.23E-06
NECAB2	0.195582983	7.79E-06	2.37E-05
NECAB3	-0.17905528	4.38E-05	0.000118
NECAP1	0.226233008	2.11E-07	8.27E-07
NECAP2	-0.12007641	0.006368	0.011724
NEDD1	0.34549264	6.96E-16	9.83E-15
NEDD4L	-0.150096492	0.000632	0.001393
NEDD4	0.094800759	0.031477	0.050289
NEDD8	0.330988271	1.24E-14	1.44E-13
NEDD9	-0.301100736	2.97E-12	2.40E-11
NEFH	0.011665545	0.791704	0.83296
NEFL	-0.043034488	0.329716	0.403129
NEFM	0.003075717	0.944488	0.955918
NEGR1	-0.241373497	2.91E-08	1.30E-07
NEIL1	-0.420596454	1.71E-23	7.52E-22
NEIL2	-0.003201596	0.94222	0.954154
NEIL3	0.528373991	2.26E-38	1.26E-35
NEK10	-0.146889252	0.000827	0.001785
NEK11	-0.234286134	7.49E-08	3.14E-07
NEK1	-0.182278383	3.16E-05	8.72E-05
NEK2	0.58281384	3.53E-48	1.18E-44
NEK3	-0.025483018	0.563946	0.632947
NEK4	0.142018922	0.001231	0.002577
NEK5	-0.173718643	7.40E-05	0.000192
NEK6	0.074659253	0.090544	0.130136
NEK7	-0.061498728	0.163452	0.219798
NEK8	-0.390169635	3.58E-20	9.60E-19
NEK9	-0.164425258	0.000178	0.000435
NELF	0.087535351	0.047091	0.072421
NELL1	-0.263248074	1.30E-09	7.10E-09
NELL2	-0.227567195	1.79E-07	7.06E-07
NENF	-0.044124545	0.317601	0.390131

NEO1	-0.117686999	0.007505	0.013642
NES	-0.067829323	0.124214	0.172309
NET1	0.048848754	0.268501	0.338172
NETO1	0.100008186	0.023224	0.038184
NETO2	0.250784148	7.94E-09	3.85E-08
NEU1	-0.007095406	0.872384	0.900496
NEU2	0.148233663	0.00074	0.001612
NEU3	0.002784277	0.949741	0.960797
NEU4	0.001384813	0.97499	0.981225
NEURL1B	-0.069085307	0.117382	0.163958
NEURL2	0.051440026	0.2439	0.311307
NEURL3	-0.156887478	0.000352	0.00081
NEURL4	-0.194028538	9.22E-06	2.78E-05
NEURL	0.058697989	0.183525	0.243127
NEUROD1	0.065484829	0.13779	0.189198
NEUROD2	-0.117080568	0.007822	0.01417
NEUROD4	0.020680294	0.639627	0.700694
NEUROD6	0.020861618	0.636694	0.69794
NEUROG1	0.068119318	0.12261	0.170576
NEUROG2	0.139318204	0.001527	0.003143
NEUROG3	0.058336283	0.186245	0.246289
NEXN	-0.038698617	0.38081	0.45535
NF1P1	-0.032749274	0.458334	0.532163
NF1	-0.058613829	0.184155	0.243865
NF2	-0.043613456	0.323245	0.396357
NFAM1	-0.288499473	2.50E-11	1.75E-10
NFASC	-0.358882645	4.23E-17	7.17E-16
NFAT5	-0.293389588	1.11E-11	8.21E-11
NFATC1	-0.389483804	4.22E-20	1.12E-18
NFATC2IP	-0.229959928	1.31E-07	5.32E-07
NFATC2	-0.238754121	4.15E-08	1.81E-07
NFATC3	-0.320968252	8.35E-14	8.54E-13
NFATC4	-0.122452753	0.005392	0.010074
NFE2L1	-0.125737919	0.004265	0.008104
NFE2L2	-0.196317791	7.19E-06	2.20E-05
NFE2L3	-0.014656146	0.740032	0.789568
NFE2	-0.080811834	0.066884	0.099396
NFIA	-0.263710988	1.22E-09	6.67E-09
NFIB	-0.171639611	9.05E-05	0.000232
NFIC	-0.187673887	1.81E-05	5.22E-05
NFIL3	0.162038356	0.000222	0.000532
NFIX	-0.509688458	2.13E-35	6.70E-33
NFKB1	-0.1285072	0.003485	0.006725
NFKB2	-0.039642752	0.36929	0.444045

NFKBIA	-0.033350561	0.45012	0.524453
NFKBIB	0.015642584	0.72323	0.775282
NFKBID	-0.367823838	6.06E-18	1.17E-16
NFKBIE	-0.056199911	0.202916	0.265508
NFKBIL1	-0.02460733	0.57742	0.644919
NFKBIL2	0.218029658	5.85E-07	2.14E-06
NFKBIZ	-0.221528952	3.81E-07	1.43E-06
NFRKB	-0.105466598	0.016653	0.028199
NFS1	0.100480874	0.022578	0.037201
NFU1	0.229656224	1.37E-07	5.51E-07
NFX1	-0.074188086	0.092603	0.132704
NFXL1	0.362839975	1.80E-17	3.23E-16
NFYA	0.121191851	0.005892	0.010917
NFYB	0.206408575	2.32E-06	7.66E-06
NFYC	-0.221743602	3.71E-07	1.40E-06
NGB	-0.106360921	0.015748	0.026819
NGDN	0.235778641	6.16E-08	2.61E-07
NGEF	0.133517844	0.002395	0.004763
NGFRAP1	-0.078084823	0.076656	0.112357
NGFR	-0.284680173	4.66E-11	3.15E-10
NGF	-0.095089298	0.030962	0.049536
NGLY1	0.012958302	0.769241	0.814557
NGRN	0.084427552	0.055527	0.084049
NHEDC1	-0.081183766	0.065634	0.097756
NHEDC2	0.147772416	0.000769	0.001669
NHEG1	0.113482961	0.009954	0.017688
NHEJ1	0.099764265	0.023564	0.038673
NHLH1	-0.034128893	0.439611	0.513916
NHLH2	-0.124947154	0.004515	0.008546
NHLRC1	-0.092730945	0.035394	0.055925
NHLRC2	0.208011922	1.92E-06	6.45E-06
NHLRC3	-0.205614207	2.54E-06	8.33E-06
NHLRC4	-0.24413307	2.00E-08	9.16E-08
NHP2L1	0.10964678	0.012783	0.022171
NHP2	0.131503166	0.00279	0.005478
NHSL1	-0.08201979	0.062894	0.094062
NHSL2	-0.336281062	4.42E-15	5.54E-14
NHS	-0.21003316	1.52E-06	5.17E-06
NICN1	-0.413070219	1.22E-22	4.71E-21
NID1	0.056843783	0.197782	0.259519
NID2	0.167930328	0.000129	0.000322
NIF3L1	0.337557022	3.43E-15	4.39E-14
NINJ1	-0.200678641	4.44E-06	1.40E-05
NINJ2	-0.19765424	6.21E-06	1.92E-05

NINL	-0.171400587	9.26E-05	0.000237
NIN	0.039089126	0.376018	0.45078
NIP7	0.43484852	3.60E-25	2.06E-23
NIPA1	0.021244713	0.630515	0.692357
NIPA2	0.246383839	1.47E-08	6.85E-08
NIPAL1	0.133382128	0.00242	0.004809
NIPAL2	-0.002572777	0.953554	0.964215
NIPAL3	-0.383021847	1.93E-19	4.67E-18
NIPAL4	0.074814044	0.089876	0.129282
NIPBL	-0.132527864	0.002582	0.005103
NIPSNAP1	0.259354136	2.32E-09	1.21E-08
NIPSNAP3A	0.007002905	0.874034	0.901682
NIPSNAP3B	-0.336846017	3.95E-15	5.00E-14
NISCH	-0.498891214	9.20E-34	2.00E-31
NIT1	-0.047787112	0.279055	0.34957
NIT2	0.196764251	6.84E-06	2.10E-05
NKAIN1	0.216064334	7.42E-07	2.66E-06
NKAIN2	0.091664874	0.037568	0.058979
NKAIN3	0.177537047	5.09E-05	0.000136
NKAIN4	0.106342679	0.015766	0.026843
NKAPL	-0.16066245	0.000251	0.000596
NKAP	0.088102441	0.045674	0.070457
NKD1	-0.143224862	0.001117	0.002357
NKD2	-0.27223914	3.34E-10	1.99E-09
NKG7	0.053894465	0.222095	0.286977
NKIRAS1	-0.109423272	0.012968	0.022475
NKIRAS2	0.165554828	0.000161	0.000395
NKPD1	-0.003772835	0.931934	0.946712
NKRF	0.24685778	1.37E-08	6.43E-08
NKTR	-0.336608654	4.14E-15	5.22E-14
NKX1-2	0.207777227	1.98E-06	6.61E-06
NKX2-1	-0.279803058	1.02E-10	6.57E-10
NKX2-2	0.063467706	0.15036	0.204254
NKX2-3	0.149951334	0.00064	0.001409
NKX2-4	0.013459132	0.760589	0.807149
NKX2-5	0.138811814	0.00159	0.003258
NKX2-6	0.067665432	0.125128	0.173432
NKX2-8	-0.057600957	0.191865	0.252768
NKX3-1	-0.009780601	0.824763	0.86045
NKX3-2	0.053969389	0.221452	0.28622
NKX6-1	0.010594921	0.810439	0.848474
NKX6-2	-0.024762648	0.57502	0.642669
NKX6-3	-0.04401308	0.318827	0.39142
NLE1	0.29478273	8.75E-12	6.61E-11

NLGN1	-0.046054165	0.296879	0.368252
NLGN2	-0.096282904	0.028905	0.046563
NLGN3	-0.268597632	5.83E-10	3.36E-09
NLGN4X	0.024506841	0.578976	0.646188
NLGN4Y	0.153965661	0.000454	0.001026
NLK	0.010035907	0.820266	0.856652
NLN	0.346792413	5.33E-16	7.71E-15
NLRC3	-0.28950537	2.11E-11	1.51E-10
NLRC4	-0.248465726	1.10E-08	5.22E-08
NLRC5	-0.135312721	0.002088	0.0042
NLRP10	0.070282488	0.111147	0.156209
NLRP11	0.110147513	0.012378	0.021528
NLRP12	-0.058734599	0.183251	0.24278
NLRP13	0.074443881	0.091481	0.13134
NLRP14	-0.144060204	0.001044	0.002213
NLRP1	-0.437431286	1.75E-25	1.06E-23
NLRP2	-0.086088613	0.050874	0.077713
NLRP3	-0.295705965	7.49E-12	5.72E-11
NLRP4	0.001442841	0.973943	0.980417
NLRP5	0.138255422	0.001661	0.003395
NLRP6	-0.151425131	0.000565	0.001254
NLRP7	-0.017973405	0.684064	0.740847
NLRP8	0.152301903	0.000524	0.001171
NLRP9	-0.323880345	4.84E-14	5.14E-13
NLRX1	-0.174136829	7.11E-05	0.000185
NMBR	-0.242114045	2.64E-08	1.18E-07
NMB	0.24026514	3.38E-08	1.50E-07
NMD3	0.388883927	4.86E-20	1.27E-18
NME1-NME2	0.242322724	2.56E-08	1.15E-07
NME1	0.501738123	3.45E-34	8.38E-32
NME2P1	0.327679617	2.35E-14	2.62E-13
NME2	0.383376067	1.78E-19	4.33E-18
NME3	-0.202880191	3.46E-06	1.11E-05
NME4	0.182524025	3.08E-05	8.53E-05
NME5	-0.236009302	5.97E-08	2.54E-07
NME6	0.244782116	1.83E-08	8.42E-08
NME7	0.140250678	0.001419	0.002936
NMI	0.136464714	0.00191	0.003862
NMNAT1	-0.108489034	0.013766	0.023748
NMNAT2	-0.138668006	0.001608	0.003293
NMNAT3	-0.275946119	1.88E-10	1.16E-09
NMRAL1	-0.069976049	0.112718	0.15825
NMT1	0.074955299	0.089269	0.128507
NMT2	-0.078857298	0.073777	0.10847

NMUR1	-0.257326002	3.11E-09	1.59E-08
NMUR2	-0.184152301	2.61E-05	7.31E-05
NMU	0.1976507	6.21E-06	1.92E-05
NNAT	-0.241480488	2.87E-08	1.28E-07
NNMT	0.170291205	0.000103	0.000262
NNT	-0.057587528	0.191969	0.252855
NOB1	0.260808211	1.87E-09	9.93E-09
NOBOX	-0.025493466	0.563786	0.632839
NOC2L	0.116077358	0.008371	0.015074
NOC3L	0.279434352	1.08E-10	6.92E-10
NOC4L	0.15293702	0.000496	0.001113
NOD1	-0.443326238	3.31E-26	2.21E-24
NOD2	-0.205398318	2.60E-06	8.52E-06
NODAL	-0.09991958	0.023347	0.038364
NOG	-0.104391566	0.0178	0.029972
NOL10	0.351368863	2.07E-16	3.14E-15
NOL11	0.368219536	5.55E-18	1.08E-16
NOL12	-0.0576818	0.191241	0.252046
NOL3	-0.030659947	0.487521	0.560871
NOL4	0.066096605	0.134143	0.184748
NOL6	0.151913295	0.000542	0.001207
NOL7	0.324607826	4.22E-14	4.54E-13
NOL8	0.021352361	0.628783	0.691164
NOL9	0.062435021	0.157123	0.212287
NOLC1	0.390085139	3.65E-20	9.77E-19
NOM1	0.081830984	0.063505	0.094868
NOMO1	0.059901338	0.174688	0.233113
NOMO2	0.019436098	0.659904	0.719006
NOMO3	-0.044739066	0.310902	0.382937
NONO	0.204680202	2.82E-06	9.20E-06
NOP10	0.32203594	6.84E-14	7.10E-13
NOP14	0.271065025	4.00E-10	2.36E-09
NOP16	0.334104602	6.78E-15	8.25E-14
NOP2	0.368904528	4.77E-18	9.37E-17
NOP56	0.342964107	1.16E-15	1.59E-14
NOP58	0.314868823	2.57E-13	2.46E-12
NOS1AP	-0.177853044	4.93E-05	0.000132
NOS1	-0.084625447	0.054955	0.083309
NOS2	0.020575703	0.641322	0.702243
NOS3	-0.040251649	0.361977	0.436512
NOSIP	0.064092966	0.146374	0.19949
NOSTRIN	-0.220834472	4.15E-07	1.55E-06
NOTCH1	-0.253710715	5.24E-09	2.61E-08
NOTCH2NL	-0.08585058	0.05152	0.078598

NOTCH2	-0.173942101	7.25E-05	0.000188
NOTCH3	0.06004988	0.173619	0.231879
NOTCH4	-0.263052013	1.34E-09	7.29E-09
NOTO	-0.156600369	0.000361	0.000829
NOTUM	-0.096642548	0.028309	0.04571
NOVA1	-0.055492524	0.208669	0.271984
NOVA2	-0.173101835	7.86E-05	0.000203
NOV	-0.084776973	0.05452	0.082751
NOX1	-0.121202996	0.005887	0.010909
NOX3	0.009542999	0.828954	0.863909
NOX4	0.152633503	0.000509	0.00114
NOX5	0.107447251	0.014707	0.025225
NOXA1	-0.185636648	2.24E-05	6.34E-05
NOXO1	0.030380338	0.491501	0.5648
NPAS1	0.009291841	0.833389	0.867592
NPAS2	-0.083225012	0.059112	0.08891
NPAS3	-0.022367687	0.612552	0.676559
NPAS4	-0.088515263	0.044665	0.069057
NPAT	-0.05784754	0.189967	0.250548
NPBWR1	0.072648297	0.099594	0.141767
NPBWR2	-0.019750888	0.65475	0.714363
NPB	0.05844125	0.185452	0.245306
NPC1L1	0.054454264	0.217323	0.28163
NPC1	-0.115049951	0.008969	0.016052
NPC2	-0.313445646	3.33E-13	3.14E-12
NPDC1	-0.196130812	7.34E-06	2.24E-05
NPEPL1	-0.259049256	2.42E-09	1.26E-08
NPEPPS	0.137973437	0.001698	0.003467
NPFFR1	-0.234014769	7.76E-08	3.25E-07
NPFFR2	0.070001324	0.112587	0.158089
NPFF	-0.265452234	9.38E-10	5.24E-09
NPHP1	-0.22340437	3.02E-07	1.15E-06
NPHP3	-0.3082677	8.45E-13	7.48E-12
NPHP4	-0.285557891	4.04E-11	2.75E-10
NPHS1	-0.106235166	0.015873	0.026999
NPHS2	-0.009421666	0.831095	0.865475
NPIPL3	-0.292268847	1.34E-11	9.81E-11
NPIP	-0.359562999	3.66E-17	6.27E-16
NPLOC4	0.020216971	0.647148	0.707421
NPL	-0.121095331	0.005931	0.010984
NPM1	0.259782015	2.18E-09	1.14E-08
NPM2	-0.163272566	0.000198	0.00048
NPM3	0.37705044	7.64E-19	1.69E-17
NPNT	-0.254752295	4.51E-09	2.26E-08

NPPA	-0.185889866	2.18E-05	6.19E-05
NPPB	0.041912261	0.342495	0.416584
NPPC	-0.083709284	0.057646	0.086927
NPR1	-0.30766948	9.40E-13	8.27E-12
NPR2	-0.264322861	1.11E-09	6.14E-09
NPR3	-0.075296314	0.087819	0.126702
NPRL2	-0.048142555	0.27549	0.345886
NPRL3	-0.083313739	0.058841	0.088569
NPSR1	0.076592746	0.082477	0.119833
NPTN	0.166708579	0.000144	0.000358
NPTX1	-0.106690451	0.015426	0.026315
NPTX2	0.100870734	0.022056	0.036437
NPTXR	-0.231260548	1.11E-07	4.55E-07
NPVF	0.031869722	0.4705	0.544241
NPW	0.211413002	1.29E-06	4.45E-06
NPY1R	-0.160974874	0.000244	0.000581
NPY2R	-0.074154316	0.092752	0.132841
NPY5R	-0.075354694	0.087572	0.126373
NPY6R	-0.283091221	6.03E-11	4.00E-10
NPY	-0.036338683	0.410551	0.485195
NQO1	0.207490798	2.04E-06	6.81E-06
NQO2	0.114315011	0.00942	0.016803
NR0B1	0.216652022	6.91E-07	2.49E-06
NR0B2	-0.252930446	5.85E-09	2.89E-08
NR1D1	-0.101328228	0.021457	0.035542
NR1D2	-0.148334957	0.000733	0.001599
NR1H2	-0.194840937	8.44E-06	2.56E-05
NR1H3	-0.149387063	0.000671	0.001474
NR1H4	0.054265508	0.218924	0.283429
NR1I2	-0.070344304	0.110832	0.155822
NR1I3	0.073862929	0.094046	0.134578
NR2C1	-0.033912429	0.442519	0.516772
NR2C2AP	0.12745049	0.003766	0.007221
NR2C2	-0.255800646	3.88E-09	1.96E-08
NR2E1	-0.083117022	0.059443	0.089361
NR2E3	-0.229810646	1.34E-07	5.41E-07
NR2F1	-0.271462082	3.76E-10	2.23E-09
NR2F2	-0.097940163	0.026245	0.042674
NR2F6	0.128652834	0.003448	0.006658
NR3C1	-0.023410028	0.596084	0.662009
NR3C2	-0.475922272	1.80E-30	2.17E-28
NR4A1	-0.139887341	0.00146	0.003015
NR4A2	-0.046661116	0.290552	0.361638
NR4A3	-0.1561688	0.000375	0.000858

NR5A1	0.113494297	0.009947	0.017676
NR5A2	-0.10408467	0.01814	0.030501
NR6A1	-0.018163086	0.680912	0.738074
NRADDP	-0.237699342	4.77E-08	2.06E-07
NRAP	-0.199221559	5.22E-06	1.63E-05
NRARP	0.249551546	9.44E-09	4.54E-08
NRAS	0.400245151	3.10E-21	9.78E-20
NRBF2	0.323957773	4.77E-14	5.07E-13
NRBP1	0.212277271	1.17E-06	4.05E-06
NRBP2	-0.195579119	7.79E-06	2.37E-05
NRCAM	-0.095625807	0.030022	0.048215
NRD1	0.155370285	0.000402	0.000916
NRF1	-0.078477903	0.07518	0.110403
NRG1	-0.048884783	0.268147	0.337791
NRG2	-0.257894702	2.87E-09	1.48E-08
NRG3	-0.157629207	0.000329	0.000763
NRG4	0.027088742	0.539638	0.610533
NRGN	-0.281376125	7.94E-11	5.19E-10
NRIP1	0.245422181	1.68E-08	7.75E-08
NRIP2	-0.386764795	8.03E-20	2.03E-18
NRIP3	0.047035714	0.286692	0.357636
NRK	0.048218116	0.274737	0.345113
NRL	-0.093400951	0.034083	0.054007
NRM	0.11229444	0.010765	0.018984
NRN1L	-0.21949355	4.89E-07	1.81E-06
NRN1	-0.133894647	0.002328	0.004638
NRP1	-0.046812113	0.288992	0.360055
NRP2	0.02630936	0.551371	0.621416
NRSN1	0.007152517	0.871366	0.899724
NRSN2	-0.055127055	0.211686	0.275324
NRTN	-0.019669037	0.656089	0.715629
NRXN1	-0.143105246	0.001128	0.002377
NRXN2	-0.331534037	1.12E-14	1.31E-13
NRXN3	-0.214747597	8.70E-07	3.08E-06
NSA2	0.02166422	0.623778	0.686606
NSD1	-0.120725768	0.006086	0.011245
NSDHL	0.24046896	3.29E-08	1.46E-07
NSFL1C	0.032409751	0.46301	0.536968
NSF	0.13773615	0.00173	0.003526
NSL1	0.208675515	1.78E-06	6.01E-06
NSMAF	0.13264048	0.00256	0.005062
NSMCE1	-0.016904516	0.701928	0.757374
NSMCE2	0.28081919	8.68E-11	5.65E-10
NSMCE4A	0.046959688	0.287473	0.358453

NSUN2	0.224241845	2.72E-07	1.04E-06
NSUN3	0.07783189	0.077619	0.113544
NSUN4	-0.083822481	0.057307	0.086463
NSUN5P1	-0.210853206	1.38E-06	4.74E-06
NSUN5P2	-0.20056989	4.49E-06	1.42E-05
NSUN5	0.107018518	0.015111	0.025846
NSUN6	-0.09585362	0.029631	0.047648
NSUN7	-0.199020454	5.34E-06	1.67E-05
NT5C1A	-0.28294377	6.17E-11	4.09E-10
NT5C1B	-0.188140563	1.73E-05	4.99E-05
NT5C2	-0.000350886	0.993662	0.995305
NT5C3L	0.195583076	7.79E-06	2.37E-05
NT5C3	0.335561768	5.09E-15	6.33E-14
NT5C	0.047842724	0.278495	0.349029
NT5DC1	-0.148527556	0.000722	0.001576
NT5DC2	0.040652147	0.357216	0.431683
NT5DC3	0.155729148	0.000389	0.00089
NT5E	-0.039755963	0.367923	0.442562
NT5M	-0.020234341	0.646865	0.707213
NTAN1	0.029872915	0.498769	0.571869
NTF3	-0.157413737	0.000336	0.000776
NTF4	-0.355804425	8.15E-17	1.32E-15
NTHL1	-0.009637357	0.827289	0.8625
NTM	-0.051441649	0.243885	0.311307
NTN1	-0.172808536	8.09E-05	0.000208
NTN3	-0.110386161	0.012189	0.021233
NTN4	-0.286877648	3.26E-11	2.25E-10
NTN5	-0.315046406	2.49E-13	2.39E-12
NTNG1	-0.167564614	0.000133	0.000332
NTNG2	0.02518307	0.568544	0.636963
NTRK1	-0.273617087	2.70E-10	1.63E-09
NTRK2	-0.306119372	1.24E-12	1.07E-11
NTRK3	-0.368170992	5.61E-18	1.09E-16
NTSR1	0.186570644	2.03E-05	5.80E-05
NTSR2	-0.056145627	0.203354	0.265958
NTS	0.184882836	2.42E-05	6.81E-05
NUAK1	-0.027791636	0.529165	0.601065
NUAK2	-0.165704909	0.000158	0.00039
NUB1	-0.024694304	0.576075	0.643668
NUBP1	-0.190870398	1.29E-05	3.81E-05
NUBP2	-0.057963137	0.189082	0.249529
NUBPL	0.205215635	2.66E-06	8.69E-06
NUCB1	-0.272719267	3.10E-10	1.85E-09
NUCB2	0.026096793	0.554593	0.624167

NUCKS1	0.088851837	0.043857	0.067907
NUDCD1	0.459969335	2.51E-28	2.13E-26
NUDCD2	0.182961498	2.95E-05	8.19E-05
NUDCD3	-0.040310155	0.361279	0.43596
NUDC	-0.017560647	0.690942	0.747284
NUDT10	-0.065410631	0.138238	0.189734
NUDT11	0.086349652	0.050173	0.076801
NUDT12	-0.167589737	0.000133	0.000331
NUDT13	-0.155455062	0.000399	0.000909
NUDT14	-0.126122608	0.004148	0.007899
NUDT15	0.383361331	1.78E-19	4.34E-18
NUDT16L1	-0.186750284	2.00E-05	5.71E-05
NUDT16P1	0.054906393	0.213523	0.277479
NUDT16	-0.320291618	9.48E-14	9.62E-13
NUDT17	-0.111759971	0.011148	0.019591
NUDT18	-0.183870302	2.69E-05	7.51E-05
NUDT19	0.181099997	3.56E-05	9.76E-05
NUDT1	0.278765238	1.20E-10	7.65E-10
NUDT21	0.186606308	2.03E-05	5.78E-05
NUDT22	-0.032356914	0.46374	0.537596
NUDT2	0.212969779	1.08E-06	3.75E-06
NUDT3	0.303166887	2.08E-12	1.72E-11
NUDT4	0.074813241	0.089879	0.129282
NUDT5	0.321329622	7.81E-14	8.04E-13
NUDT6	0.070744917	0.10881	0.153312
NUDT7	-0.187970115	1.76E-05	5.07E-05
NUDT8	0.064410508	0.14438	0.197204
NUDT9P1	0.027510875	0.533336	0.604669
NUDT9	0.136788716	0.001863	0.003773
NUF2	0.532895157	4.04E-39	2.72E-36
NUFIP1	0.309609404	6.65E-13	5.99E-12
NUFIP2	-0.054671511	0.215492	0.279691
NUMA1	-0.293003587	1.18E-11	8.73E-11
NUMBL	0.055755305	0.206518	0.269497
NUMB	0.010452195	0.812945	0.850652
NUP107	0.319618483	1.07E-13	1.09E-12
NUP133	0.113972519	0.009637	0.01717
NUP153	0.211459275	1.29E-06	4.43E-06
NUP155	0.293077005	1.17E-11	8.63E-11
NUP160	0.11598973	0.00842	0.015152
NUP188	0.018898147	0.668751	0.726822
NUP205	0.321725497	7.25E-14	7.51E-13
NUP210L	-0.221478924	3.83E-07	1.44E-06
NUP210	0.034779001	0.430941	0.505557

NUP214	-0.106376868	0.015732	0.026797
NUP35	0.346493508	5.67E-16	8.13E-15
NUP37	0.531287221	7.48E-39	4.82E-36
NUP43	0.0642366	0.145469	0.198501
NUP50	0.128571579	0.003469	0.006697
NUP54	0.32155548	7.49E-14	7.74E-13
NUP62CL	0.155038579	0.000414	0.000941
NUP62	0.103118633	0.019248	0.032187
NUP85	0.187144643	1.92E-05	5.49E-05
NUP88	0.204755551	2.80E-06	9.13E-06
NUP93	0.219216579	5.06E-07	1.87E-06
NUP98	0.152148212	0.000531	0.001185
NUPL1	0.016536179	0.708122	0.762523
NUPL2	0.141168526	0.001318	0.002743
NUPR1	-0.220140852	4.52E-07	1.68E-06
NUS1	0.314452552	2.78E-13	2.64E-12
NUSAP1	0.485390995	8.49E-32	1.31E-29
NUTF2	0.30388153	1.83E-12	1.54E-11
NVL	-0.015060873	0.733123	0.783872
NWD1	-0.351764137	1.91E-16	2.91E-15
NXF1	-0.353608089	1.30E-16	2.03E-15
NXF2B	-0.160383812	0.000258	0.00061
NXF2	-0.111464263	0.011365	0.019949
NXF3	-0.232629719	9.30E-08	3.85E-07
NXF4	-0.075974432	0.084991	0.123021
NXF5	-0.124675217	0.004604	0.008703
NXNL1	-0.18063069	3.74E-05	0.000102
NXNL2	0.06428865	0.145143	0.198096
NXN	-0.191587647	1.20E-05	3.55E-05
NXPH1	0.095410819	0.030396	0.048732
NXPH2	0.013687948	0.756646	0.803647
NXPH3	-0.342527068	1.27E-15	1.73E-14
NXPH4	0.123794775	0.004903	0.009226
NXT1	0.234331806	7.45E-08	3.12E-07
NXT2	0.139051009	0.00156	0.003204
NYNRIN	-0.216598015	6.96E-07	2.50E-06
NYX	0.106516512	0.015595	0.026572
OAF	-0.010138242	0.818465	0.855129
OAS1	0.161321821	0.000237	0.000564
OAS2	-0.063048136	0.15308	0.20747
OAS3	0.104747765	0.017413	0.029356
OASL	0.082427283	0.061594	0.092337
OAT	0.166262841	0.00015	0.000372
OAZ1	0.00150978	0.972734	0.979546

OAZ2	-0.121665464	0.005699	0.010592
OAZ3	0.035905988	0.41615	0.490912
OBFC1	-0.135372365	0.002078	0.004182
OBFC2A	-0.03308918	0.45368	0.527828
OBFC2B	0.426442996	3.58E-24	1.79E-22
OBP2A	0.19049032	1.35E-05	3.96E-05
OBP2B	0.166372016	0.000149	0.000368
OBSCN	-0.24319785	2.27E-08	1.03E-07
OBSL1	-0.151160285	0.000578	0.001281
OC90	0.043352637	0.32615	0.399404
OCA2	-0.033907451	0.442586	0.51682
OCEL1	-0.105369024	0.016754	0.028358
OCIAD1	0.20699142	2.17E-06	7.19E-06
OCIAD2	0.143105501	0.001128	0.002377
OCLM	-0.046203284	0.295316	0.366655
OCLN	-0.286459421	3.49E-11	2.40E-10
OCM2	-0.012619219	0.775115	0.819177
OCM	-0.223416204	3.01E-07	1.15E-06
OCRL	0.124067402	0.004808	0.009064
ODAM	-0.241628935	2.81E-08	1.26E-07
ODC1	0.225037726	2.46E-07	9.51E-07
ODF1	-0.116668721	0.008043	0.014546
ODF2L	-0.046972571	0.28734	0.358333
ODF2	0.026192422	0.553142	0.62299
ODF3B	-0.254136691	4.93E-09	2.46E-08
ODF3L1	-0.158611516	0.000302	0.000705
ODF3L2	0.045300274	0.304865	0.376616
ODF3	-0.059667451	0.17638	0.235142
ODF4	-0.016738157	0.704723	0.759722
ODZ1	-0.034570929	0.433705	0.50835
ODZ2	-0.188740094	1.62E-05	4.70E-05
ODZ3	0.08575652	0.051777	0.07896
ODZ4	-0.086662212	0.049345	0.075591
OFD1	-0.33331233	7.91E-15	9.48E-14
OGDHL	0.024828399	0.574005	0.641761
OGDH	-0.11533528	0.008799	0.015771
OGFOD1	0.226451153	2.06E-07	8.07E-07
OGFOD2	-0.133563565	0.002387	0.004748
OGFRL1	-0.097979351	0.026185	0.04259
OGFR	-0.217013273	6.62E-07	2.39E-06
OGG1	-0.016040169	0.716494	0.769258
OGN	-0.314487618	2.76E-13	2.62E-12
OGT	-0.284471297	4.82E-11	3.25E-10
OIP5	0.524942749	8.21E-38	4.10E-35

OIT3	-0.140513561	0.001389	0.00288
OLA1	0.46689879	3.04E-29	2.96E-27
OLAH	0.159811364	0.000271	0.000639
OLFM1	-0.403234615	1.48E-21	4.93E-20
OLFM2	-0.077579917	0.078587	0.1148
OLFM3	0.032793801	0.457723	0.53167
OLFM4	0.06414296	0.146058	0.199155
OLFML1	-0.225448947	2.33E-07	9.06E-07
OLFML2A	-0.049336303	0.263747	0.332919
OLFML2B	0.102370959	0.020145	0.033553
OLFML3	-0.167464284	0.000134	0.000335
OLIG1	-0.267307953	7.09E-10	4.03E-09
OLIG2	0.053492477	0.225568	0.290875
OLIG3	0.025412766	0.565021	0.633905
OLR1	-0.263540997	1.25E-09	6.81E-09
OMA1	-0.174390254	6.94E-05	0.000181
OMD	-0.095052925	0.031026	0.049629
OMG	-0.140445113	0.001397	0.002894
OMP	0.063976355	0.147111	0.200378
ONECUT1	0.025335915	0.566199	0.634941
ONECUT2	0.025009086	0.57122	0.639352
ONECUT3	-0.02178613	0.621827	0.684887
OOEP	0.116519448	0.008125	0.014675
OPA1	0.255180643	4.24E-09	2.14E-08
OPA3	-0.03869282	0.380881	0.455408
OPALIN	-0.035292575	0.424164	0.498805
OPCML	-0.081588326	0.064296	0.095893
OPHN1	-0.249236929	9.86E-09	4.73E-08
OPLAH	-0.015102876	0.732407	0.783359
OPN1LW	0.001220599	0.977955	0.983719
OPN1MW	0.026680261	0.545772	0.616391
OPN1SW	0.090803223	0.039408	0.061586
OPN3	0.358768215	4.34E-17	7.33E-16
OPN4	0.021280236	0.629943	0.691906
OPN5	-0.049885052	0.258466	0.327224
OPRD1	0.045396897	0.303834	0.375632
OPRK1	-0.172374195	8.43E-05	0.000217
OPRL1	-0.149007353	0.000693	0.001519
OPRM1	-0.014290624	0.746291	0.795014
OPTC	0.079058422	0.073042	0.107489
OPTN	-0.003265656	0.941066	0.953493
OR10A2	0.001991321	0.964043	0.972264
OR10A3	-0.115114773	0.00893	0.015991
OR10A4	-0.038235028	0.386545	0.461325

OR10A5	0.029395953	0.505652	0.578401
OR10A6	-0.091434375	0.038053	0.059688
OR10AD1	-0.227930733	1.70E-07	6.76E-07
OR10G2	-0.083941915	0.056952	0.085992
OR10G3	0.031352544	0.477736	0.551519
OR10G4	-0.007034604	0.873468	0.901214
OR10G7	-0.068873302	0.118514	0.165343
OR10G8	-0.015840184	0.719879	0.772312
OR10H1	-0.018967128	0.667614	0.725892
OR10H2	-0.001642106	0.970346	0.977683
OR10H5	-0.004610155	0.916878	0.934885
OR10J1	-0.045594964	0.301727	0.373408
OR10J3	-0.006927922	0.875371	0.902741
OR10J5	0.033533275	0.44764	0.521898
OR10P1	0.034114181	0.439808	0.514117
OR10Q1	0.061262889	0.165076	0.221728
OR10S1	0.003834675	0.930821	0.945918
OR10V1	-0.150086661	0.000633	0.001394
OR10W1	0.052819253	0.231469	0.297608
OR11A1	-0.056746392	0.198552	0.260342
OR11G2	-0.013733795	0.755856	0.803106
OR11H12	-0.028535183	0.518199	0.590387
OR11H4	-0.056089245	0.203809	0.266413
OR11H6	0.01910244	0.665386	0.723992
OR12D2	0.006593434	0.881343	0.906934
OR13A1	-0.153867091	0.000458	0.001034
OR13C2	-0.110159554	0.012368	0.021514
OR13C3	0.038408295	0.384396	0.459033
OR13C5	-0.103515909	0.018786	0.03148
OR13C9	-0.086857339	0.048834	0.074865
OR13D1	0.007645392	0.862587	0.891949
OR13F1	-0.032296567	0.464574	0.538407
OR13G1	0.042397512	0.336931	0.410871
OR13H1	0.02180464	0.621531	0.684698
OR13J1	-0.199243879	5.21E-06	1.63E-05
OR14A16	-0.009704826	0.826099	0.861553
OR14C36	0.067460965	0.126276	0.174853
OR14I1	-0.013862822	0.753636	0.80122
OR1A2	-0.025117965	0.569545	0.637798
OR1B1	-0.108782283	0.013511	0.023328
OR1C1	-0.006350418	0.885686	0.910388
OR1D2	-0.018965474	0.667641	0.725892
OR1D4	-0.064378205	0.144582	0.197414
OR1E1	-0.059386994	0.178426	0.237457

OR1E2	-0.074334589	0.091959	0.131892
OR1F1	0.268891148	5.58E-10	3.22E-09
OR1F2P	0.157679345	0.000328	0.00076
OR1G1	-0.035829577	0.417143	0.49188
OR1J1	0.026849665	0.543224	0.613999
OR1J2	0.069050816	0.117566	0.164201
OR1J4	0.023109465	0.600812	0.666032
OR1K1	-0.106552818	0.01556	0.026518
OR1L1	-0.025946226	0.55688	0.626283
OR1L3	-0.012348095	0.779821	0.82284
OR1L4	-0.084138889	0.056371	0.085204
OR1L6	-0.021376397	0.628397	0.690929
OR1L8	-0.172216542	8.56E-05	0.00022
OR1M1	-0.037469629	0.39613	0.47102
OR1N1	-0.056218877	0.202764	0.265377
OR1N2	0.000809473	0.985379	0.988991
OR1Q1	-0.003461356	0.937542	0.950782
OR2A12	0.002290812	0.95864	0.968378
OR2A14	-0.013534744	0.759285	0.805894
OR2A1	-0.267869007	6.52E-10	3.73E-09
OR2A25	-0.044289518	0.315794	0.388149
OR2A2	-0.030187525	0.494256	0.567574
OR2A4	-0.129009725	0.003359	0.006501
OR2A5	-0.036268676	0.411454	0.486147
OR2A7	-0.110349085	0.012218	0.021282
OR2A9P	-0.134942642	0.002148	0.00431
OR2AE1	-0.050300047	0.254521	0.323069
OR2AG1	0.008570689	0.846152	0.878226
OR2AG2	0.020916335	0.63581	0.697144
OR2AK2	-0.026539044	0.5479	0.618271
OR2AT4	0.020121407	0.648704	0.708773
OR2B11	-0.145551844	0.000924	0.001976
OR2B2	0.016123244	0.71509	0.76808
OR2B3	-0.004059811	0.926771	0.94276
OR2B6	0.154863638	0.00042	0.000955
OR2C1	-0.148013453	0.000753	0.001639
OR2C3	0.06614445	0.13386	0.184397
OR2D2	-0.055455171	0.208976	0.272331
OR2D3	-0.067957717	0.123502	0.171541
OR2F1	0.019574347	0.657639	0.716955
OR2F2	0.047930234	0.277616	0.348139
OR2G2	-0.041158941	0.351248	0.42581
OR2G6	-0.016160837	0.714454	0.767687
OR2H1	0.018112365	0.681755	0.738786

OR2H2	-0.035878444	0.416508	0.491267
OR2J2	-0.057885808	0.189673	0.250244
OR2J3	-0.019855895	0.653034	0.712803
OR2K2	-0.107581927	0.014583	0.025028
OR2L13	-0.084453249	0.055452	0.083968
OR2L1P	0.004619811	0.916704	0.934804
OR2L2	-2.40E-05	0.999567	0.999867
OR2L3	-0.014049456	0.750429	0.798614
OR2M3	0.097569573	0.026821	0.043536
OR2M4	0.053889589	0.222137	0.287012
OR2S2	0.078080882	0.076671	0.11237
OR2T10	-0.143140331	0.001125	0.002371
OR2T2	-0.021435916	0.627441	0.690078
OR2T33	0.026029746	0.555611	0.625066
OR2T34	-6.45E-05	0.998834	0.999284
OR2T3	-0.076440688	0.083089	0.120595
OR2T4	0.023214226	0.599162	0.664561
OR2T5	-0.02555637	0.562824	0.632007
OR2T6	0.019349639	0.661323	0.720199
OR2T8	0.037245438	0.398964	0.473658
OR2V2	-0.018551963	0.674468	0.732296
OR2W1	0.016334105	0.711529	0.765366
OR2W3	-0.018792244	0.670498	0.728404
OR2W5	-0.016351062	0.711243	0.765141
OR2Z1	0.038790006	0.379685	0.454385
OR3A1	-0.054412383	0.217678	0.282016
OR3A2	-0.096966655	0.027781	0.044926
OR3A3	-0.069439287	0.115511	0.161739
OR3A4	-0.007686467	0.861856	0.891424
OR4A16	0.039766552	0.367796	0.442435
OR4A47	0.0163537	0.711198	0.765134
OR4C3	0.00218028	0.960634	0.969853
OR4C6	0.129022515	0.003356	0.006497
OR4D10	-0.11151858	0.011325	0.019882
OR4D1	-0.046265027	0.294671	0.366035
OR4D2	-0.041499916	0.347269	0.421625
OR4D5	-0.013582055	0.75847	0.805242
OR4D6	0.013301495	0.763309	0.809605
OR4E2	-0.096392262	0.028723	0.046303
OR4F21	-0.028706395	0.51569	0.587898
OR4F29	-0.065583165	0.137199	0.188438
OR4F4	0.043513602	0.324355	0.39745
OR4F5	-0.024451983	0.579826	0.646884
OR4F6	-0.06884255	0.118679	0.16555

OR4K17	-0.045426607	0.303517	0.375322
OR4K1	-0.015481934	0.725957	0.777622
OR4M2	-0.029948788	0.497679	0.570684
OR4N2	-0.074887936	0.089558	0.128867
OR4N3P	-0.062040657	0.159766	0.215463
OR4N4	-0.019250097	0.662958	0.721704
OR4X2	-0.028411604	0.520013	0.592191
OR51A7	-0.001259295	0.977257	0.983159
OR51B2	0.09969053	0.023668	0.038818
OR51B4	0.037701522	0.393211	0.468106
OR51B5	0.061745009	0.161769	0.217784
OR51B6	-0.047079522	0.286243	0.357232
OR51E1	0.342119182	1.38E-15	1.87E-14
OR51E2	0.141452737	0.001289	0.002688
OR51F2	0.019342425	0.661441	0.720259
OR51G2	0.004117299	0.925737	0.942092
OR51I1	-0.150542505	0.000609	0.001345
OR51I2	-0.038254671	0.386301	0.461061
OR51M1	-0.010834628	0.806235	0.845026
OR51Q1	-0.014773694	0.738023	0.788098
OR51T1	0.026599279	0.546992	0.617385
OR52A1	0.042754752	0.332872	0.406591
OR52A4	0.12729748	0.003809	0.007294
OR52A5	0.05178924	0.240709	0.307824
OR52B2	0.021433415	0.627481	0.690078
OR52B4	0.025476524	0.564045	0.633023
OR52B6	-0.147081613	0.000814	0.00176
OR52D1	-0.107145991	0.01499	0.025674
OR52E2	0.17720647	5.26E-05	0.00014
OR52E4	0.024385083	0.580864	0.647825
OR52E6	0.081614872	0.064209	0.095784
OR52E8	0.063204321	0.152063	0.206232
OR52H1	-0.142042797	0.001229	0.002573
OR52I1	0.059209315	0.17973	0.238923
OR52I2	0.014322567	0.745743	0.794475
OR52K1	-0.049722729	0.26002	0.328859
OR52K2	-0.156343995	0.000369	0.000846
OR52L1	-0.005576166	0.899544	0.921584
OR52M1	-0.006538407	0.882326	0.907759
OR52N1	-0.048745194	0.269518	0.339283
OR52N2	-0.084178716	0.056253	0.085046
OR52N4	-0.324183173	4.57E-14	4.87E-13
OR52N5	0.016993486	0.700434	0.755955
OR52R1	-0.012186779	0.782625	0.825232

OR52W1	-0.044754595	0.310734	0.382801
OR56A1	0.057768444	0.190574	0.25125
OR56A3	0.103220284	0.019129	0.032001
OR56A4	0.044996396	0.308124	0.380055
OR56A5	0.002806941	0.949333	0.960481
OR56B1	-0.304060477	1.78E-12	1.49E-11
OR56B4	-0.005950299	0.892844	0.916169
OR5A1	-0.094582451	0.031872	0.050834
OR5A2	-0.039169869	0.375032	0.449895
OR5AC2	0.017526558	0.691511	0.747667
OR5AK2	-0.143805582	0.001066	0.002255
OR5AN1	-0.050271577	0.25479	0.323287
OR5AU1	-0.114749292	0.009151	0.016356
OR5B12	0.002385006	0.956941	0.967101
OR5B21	0.012432165	0.778361	0.82169
OR5B2	-0.04322696	0.327555	0.40088
OR5B3	0.08339222	0.058602	0.088223
OR5C1	-0.110631194	0.011997	0.020921
OR5E1P	-0.081404265	0.064902	0.09671
OR5H1	0.006042728	0.891189	0.914947
OR5H2	-0.049140666	0.265647	0.335001
OR5H6	-0.023830072	0.589505	0.655669
OR5K1	-0.14804559	0.000751	0.001636
OR5K2	-0.250031386	8.82E-09	4.26E-08
OR5M11	0.033918321	0.44244	0.51671
OR5M1	0.046504593	0.292175	0.363455
OR5P2	-0.100815549	0.022129	0.036552
OR5P3	-0.10402641	0.018206	0.0306
OR5T2	-0.006293715	0.8867	0.911086
OR5T3	-0.038568155	0.382418	0.457027
OR5V1	0.030125943	0.495138	0.56839
OR6A2	-0.025552032	0.562891	0.632046
OR6B1	0.067081485	0.128427	0.177548
OR6B2	0.034981913	0.428256	0.502819
OR6B3	-0.006191921	0.88852	0.912581
OR6C2	-0.04210068	0.340328	0.414482
OR6C3	-0.018771037	0.670848	0.728666
OR6C6	0.047039689	0.286651	0.357607
OR6C70	-0.052136377	0.237567	0.304253
OR6C75	0.026426924	0.549593	0.619901
OR6C76	-0.088284085	0.045228	0.069836
OR6F1	0.119911749	0.006441	0.011849
OR6K3	-0.187683925	1.81E-05	5.22E-05
OR6K6	0.02004788	0.649902	0.70981

OR6M1	0.012579576	0.775802	0.819638
OR6N1	-0.113676351	0.009828	0.017481
OR6N2	-0.022158867	0.615875	0.679479
OR6S1	-0.104911879	0.017237	0.029086
OR6T1	-0.023372819	0.596669	0.66247
OR6V1	-0.021382957	0.628292	0.690851
OR6W1P	-0.002038138	0.963198	0.971706
OR7A5	0.044808136	0.310155	0.382158
OR7C1	-0.025431831	0.56473	0.633613
OR7D2	-0.065125077	0.139971	0.191784
OR7D4	-0.013723439	0.756035	0.803169
OR7E156P	0.046091225	0.29649	0.367838
OR7E24	-0.072979093	0.098058	0.139799
OR7E37P	-0.146450328	0.000858	0.001846
OR7E5P	0.057631841	0.191627	0.252471
OR7E91P	0.067316964	0.127089	0.17589
OR7G2	-0.047211287	0.284895	0.355794
OR7G3	-0.040194537	0.362659	0.437256
OR8A1	0.037322738	0.397986	0.472664
OR8B12	-0.009807794	0.824284	0.860072
OR8B2	0.104741804	0.017419	0.029365
OR8B3	0.048582074	0.271126	0.34107
OR8B4	0.041384201	0.348616	0.423081
OR8D1	-0.002448504	0.955796	0.966139
OR8D2	0.076979031	0.080937	0.117856
OR8G2	-0.000155828	0.997185	0.998134
OR8G5	0.04847092	0.272225	0.342216
OR8K3	0.051412487	0.244152	0.311554
OR8S1	0.135227095	0.002102	0.004224
OR9A2	-0.058049616	0.188421	0.248871
OR9A4	-0.034407316	0.435886	0.510427
OR9G4	-0.14754983	0.000783	0.001698
OR9G9	0.034230678	0.438247	0.512471
OR9K2	0.049767227	0.259593	0.328423
OR9Q1	0.038070294	0.388596	0.463441
ORAI1	0.071456682	0.105288	0.148791
ORAI2	-0.051540911	0.242975	0.310305
ORAI3	-0.161169638	0.00024	0.000571
ORAOV1	-0.046118578	0.296203	0.367528
ORC1L	0.473587688	3.78E-30	4.34E-28
ORC2L	0.259327867	2.33E-09	1.22E-08
ORC3L	-0.010531143	0.811559	0.849574
ORC4L	0.19016432	1.39E-05	4.09E-05
ORC5L	0.381003695	3.08E-19	7.25E-18

ORC6L	0.442025707	4.80E-26	3.12E-24
ORM1	-0.076914677	0.081191	0.118163
ORM2	-0.06046941	0.170628	0.228311
ORMDL1	-0.155498311	0.000397	0.000906
ORMDL2	0.313032352	3.59E-13	3.37E-12
ORMDL3	-0.429481065	1.57E-24	8.08E-23
OS9	-0.131583015	0.002773	0.005447
OSBP2	-0.163457569	0.000195	0.000472
OSBPL10	0.145080925	0.00096	0.002048
OSBPL11	-0.06138806	0.164212	0.220687
OSBPL1A	-0.120184804	0.00632	0.011644
OSBPL2	-0.113053336	0.010241	0.018145
OSBPL3	-0.009075707	0.837209	0.87068
OSBPL5	-0.079059085	0.07304	0.107489
OSBPL6	-0.228559201	1.57E-07	6.27E-07
OSBPL7	-0.356741218	6.69E-17	1.10E-15
OSBPL8	0.196739811	6.86E-06	2.10E-05
OSBPL9	-0.003163379	0.942909	0.95461
OSBP	0.035393254	0.422842	0.497397
OSCAR	-0.250765071	7.96E-09	3.86E-08
OSCP1	-0.227244151	1.86E-07	7.34E-07
OSGEPL1	-0.00103472	0.981312	0.986121
OSGEP	-0.008455558	0.848193	0.880117
OSGIN1	0.129980763	0.003126	0.006084
OSGIN2	0.255958948	3.79E-09	1.92E-08
OSMR	0.025496199	0.563744	0.632827
OSM	-0.065926402	0.13515	0.185981
OSR1	-0.298991046	4.27E-12	3.36E-11
OSR2	0.094017846	0.032913	0.052323
OST4	0.178158799	4.79E-05	0.000128
OSTBETA	0.031109457	0.481158	0.554988
OSTCL	0.209564502	1.61E-06	5.45E-06
OSTC	0.416595355	4.88E-23	2.00E-21
OSTF1	0.029328065	0.506636	0.579327
OSTM1	-0.063798081	0.148243	0.201708
OSTN	0.035256132	0.424642	0.49928
OSTalpha	-0.060538043	0.170142	0.227783
OTC	-0.303984871	1.80E-12	1.51E-11
OTOA	-0.239679359	3.66E-08	1.61E-07
OTOF	-0.010683307	0.808888	0.84716
OTOL1	-0.057064476	0.196044	0.257509
OTOP1	-0.022607011	0.608754	0.673294
OTOP2	0.09151359	0.037886	0.059468
OTOP3	0.061326357	0.164637	0.221199

OTOR	0.03919923	0.374674	0.449519
OTOS	0.048080136	0.276114	0.34656
OTP	0.034383737	0.4362	0.510706
OTUB1	0.116312403	0.008239	0.014861
OTUB2	0.287219051	3.08E-11	2.14E-10
OTUD1	-0.275403741	2.04E-10	1.25E-09
OTUD3	-0.345754803	6.59E-16	9.35E-15
OTUD4	-0.023877032	0.588772	0.654999
OTUD5	-0.196111178	7.35E-06	2.24E-05
OTUD6A	0.012695391	0.773794	0.818208
OTUD6B	0.364646263	1.22E-17	2.24E-16
OTUD7A	-0.198255576	5.81E-06	1.80E-05
OTUD7B	-0.122430841	0.005401	0.010087
OTX1	0.162683474	0.000209	0.000505
OTX2	0.088260381	0.045286	0.069914
OVCA2	0.24174917	2.77E-08	1.24E-07
OVCH1	-0.280779283	8.73E-11	5.68E-10
OVCH2	-0.26973263	4.91E-10	2.85E-09
OVGP1	-0.176391288	5.70E-05	0.00015
OVOL1	-0.016396632	0.710474	0.76452
OVOL2	-0.044962377	0.308491	0.380413
OXA1L	0.142353667	0.001199	0.002513
OXCT1	-0.114970153	0.009017	0.016128
OXCT2	-0.070952948	0.107771	0.151977
OXER1	-0.398525951	4.74E-21	1.45E-19
OXGR1	-0.015792081	0.720694	0.773062
OXNAD1	0.152156323	0.000531	0.001185
OXR1	-0.073291106	0.096626	0.137925
OXSM	0.182267302	3.17E-05	8.73E-05
OXSR1	0.116227104	0.008287	0.014938
OXTR	-0.015966518	0.71774	0.770514
OXT	-0.062312293	0.157942	0.213307
P2RX1	-0.229822073	1.34E-07	5.40E-07
P2RX2	-0.329569065	1.64E-14	1.87E-13
P2RX3	-0.055153999	0.211463	0.275087
P2RX4	-0.143540407	0.001089	0.002301
P2RX5	0.058462463	0.185293	0.245157
P2RX6	-0.257936412	2.85E-09	1.47E-08
P2RX7	-0.264826846	1.03E-09	5.72E-09
P2RY10	-0.08998766	0.041218	0.064235
P2RY11	-0.193267692	1.00E-05	3.00E-05
P2RY12	-0.300749249	3.16E-12	2.54E-11
P2RY13	-0.267054657	7.37E-10	4.18E-09
P2RY14	-0.214602842	8.85E-07	3.13E-06

P2RY1	-0.059617972	0.17674	0.235606
P2RY2	-0.150228278	0.000625	0.001379
P2RY4	-0.05927389	0.179255	0.238371
P2RY6	0.108396366	0.013848	0.023868
P2RY8	-0.20172503	3.94E-06	1.26E-05
P4HA1	0.3214024	7.70E-14	7.94E-13
P4HA2	0.089275771	0.042856	0.066506
P4HA3	0.189147518	1.55E-05	4.52E-05
P4HB	0.154368899	0.000438	0.000994
P4HTM	-0.273099823	2.92E-10	1.76E-09
P704P	0.129033215	0.003353	0.006493
PA2G4P4	0.351194261	2.15E-16	3.25E-15
PA2G4	0.492957538	6.89E-33	1.24E-30
PAAF1	0.068089739	0.122773	0.170719
PABPC1L2A	-0.095634983	0.030006	0.048194
PABPC1L2B	-0.111833477	0.011094	0.019504
PABPC1L	-0.259308764	2.33E-09	1.22E-08
PABPC1P2	0.136658452	0.001881	0.003808
PABPC1	0.285500775	4.08E-11	2.78E-10
PABPC3	0.294108656	9.80E-12	7.35E-11
PABPC4L	-0.028637515	0.516699	0.58888
PABPC4	-0.011788018	0.789568	0.83115
PABPC5	-0.100598583	0.022419	0.03697
PABPN1L	-0.103919217	0.018326	0.030782
PABPN1	0.004895609	0.911751	0.93094
PACRGL	0.137791416	0.001722	0.003512
PACRG	-0.226712092	1.99E-07	7.82E-07
PACS1	0.1530015	0.000494	0.001107
PACS2	-0.108029493	0.014175	0.024393
PACSIN1	-0.000579662	0.98953	0.99231
PACSIN2	-0.140660192	0.001373	0.002849
PACSIN3	-0.089095617	0.043279	0.067069
PADI1	0.027203613	0.53792	0.608692
PADI2	-0.272972981	2.98E-10	1.79E-09
PADI3	0.039232426	0.37427	0.449195
PADI4	-0.040704676	0.356595	0.43114
PADI6	-0.060649289	0.169357	0.226838
PAEP	0.27073895	4.21E-10	2.47E-09
PAF1	-0.035474518	0.421777	0.496392
PAFAH1B1	-0.097374375	0.027129	0.04396
PAFAH1B2	0.207628016	2.01E-06	6.71E-06
PAFAH1B3	0.248188365	1.14E-08	5.41E-08
PAFAH2	-0.005094794	0.908176	0.928191
PAG1	-0.268270082	6.13E-10	3.52E-09

PAGE1	0.094192641	0.032587	0.051876
PAGE2B	0.049005728	0.266964	0.336555
PAGE2	0.093144588	0.03458	0.054721
PAGE3	-0.059113648	0.180436	0.23967
PAGE4	0.045310107	0.30476	0.376556
PAGE5	0.022126858	0.616386	0.679967
PAH	0.203302755	3.30E-06	1.06E-05
PAICS	0.525317605	7.13E-38	3.66E-35
PAIP1	0.265718379	9.02E-10	5.05E-09
PAIP2B	-0.109806717	0.012653	0.021965
PAIP2	0.018992676	0.667193	0.725642
PAK1IP1	0.489947499	1.89E-32	3.22E-30
PAK1	0.159945389	0.000268	0.000632
PAK2	0.261343378	1.73E-09	9.23E-09
PAK3	0.024425363	0.580239	0.647236
PAK4	0.040952161	0.353676	0.428311
PAK6	-0.238015842	4.58E-08	1.98E-07
PAK7	-0.250667893	8.07E-09	3.91E-08
PALB2	0.145426452	0.000933	0.001995
PALLD	0.089087635	0.043298	0.067088
PALM2-AKAP2	-0.027373925	0.535376	0.60657
PALM2	-0.065182204	0.139623	0.191372
PALM3	-0.282236169	6.92E-11	4.56E-10
PALMD	-0.266110682	8.50E-10	4.78E-09
PALM	-0.294429518	9.29E-12	6.99E-11
PAMR1	-0.158127625	0.000315	0.000733
PAM	-0.124932041	0.00452	0.008553
PAN2	-0.260879881	1.85E-09	9.84E-09
PAN3	-0.172636071	8.22E-05	0.000212
PANK1	0.142284492	0.001205	0.002527
PANK2	-0.006376414	0.885221	0.910034
PANK3	0.218797058	5.33E-07	1.96E-06
PANK4	-0.124396674	0.004697	0.008869
PANX1	0.250378128	8.40E-09	4.07E-08
PANX2	-0.019095839	0.665494	0.724071
PANX3	0.047656504	0.280372	0.351023
PAOX	-0.254639537	4.59E-09	2.29E-08
PAPD4	-0.109753695	0.012696	0.02203
PAPD5	0.100050473	0.023166	0.038101
PAPD7	-0.117962014	0.007366	0.013413
PAPLN	-0.355401455	8.88E-17	1.42E-15
PAPL	0.074146172	0.092788	0.132883
PAPOLA	0.298200294	4.89E-12	3.82E-11
PAPOLB	0.033372933	0.449816	0.52419

PAPOLG	0.071252727	0.106288	0.150066
PAPPA2	-0.006789006	0.877851	0.904598
PAPPA	-0.012789484	0.772163	0.816744
PAPSS1	-0.142471689	0.001187	0.002492
PAPSS2	-0.19628929	7.21E-06	2.20E-05
PAQR3	0.220462259	4.34E-07	1.62E-06
PAQR4	0.059334462	0.178811	0.237906
PAQR5	-0.032863823	0.456763	0.530863
PAQR6	-0.116656213	0.00805	0.014556
PAQR7	-0.029300747	0.507032	0.579647
PAQR8	-0.224726055	2.56E-07	9.87E-07
PAQR9	0.232939073	8.94E-08	3.71E-07
PAR-SN	-0.168414796	0.000123	0.000308
PAR1	-0.152039491	0.000536	0.001195
PAR4	-0.08428507	0.055942	0.084633
PAR5	-0.18946278	1.50E-05	4.38E-05
PARD3B	-0.310408232	5.77E-13	5.25E-12
PARD3	0.245955629	1.56E-08	7.23E-08
PARD6A	-0.06576661	0.136101	0.187135
PARD6B	-0.18043463	3.81E-05	0.000104
PARD6G	0.082114578	0.06259	0.093648
PARG	0.228947965	1.50E-07	5.99E-07
PARK2	-0.185627315	2.24E-05	6.35E-05
PARK7	0.129910955	0.003142	0.006113
PARL	0.22370353	2.91E-07	1.11E-06
PARM1	-0.25573497	3.92E-09	1.98E-08
PARN	-0.174946506	6.57E-05	0.000172
PARP10	-0.217488317	6.25E-07	2.27E-06
PARP11	-0.134219298	0.002271	0.004533
PARP12	0.041479517	0.347506	0.421838
PARP14	-0.224017589	2.79E-07	1.07E-06
PARP15	-0.309674901	6.57E-13	5.92E-12
PARP16	-0.209725125	1.58E-06	5.35E-06
PARP1	0.172637798	8.22E-05	0.000212
PARP2	0.229879026	1.33E-07	5.37E-07
PARP3	-0.306435812	1.17E-12	1.01E-11
PARP4	-0.110678571	0.01196	0.020866
PARP6	-0.149456899	0.000667	0.001466
PARP8	-0.219164906	5.09E-07	1.88E-06
PARP9	0.013567198	0.758726	0.805419
PARS2	0.137718166	0.001732	0.00353
PART1	0.064364762	0.144666	0.197513
PARVA	-0.098794061	0.024959	0.040749
PARVB	0.118044186	0.007324	0.013347

PARVG	-0.334347693	6.46E-15	7.90E-14
PASD1	0.098880155	0.024833	0.040562
PASK	-0.136957775	0.001838	0.003728
PATE1	0.0455296	0.302421	0.374105
PATE2	0.213277302	1.04E-06	3.63E-06
PATE3	-0.020502282	0.642512	0.703277
PATE4	0.054377862	0.21797	0.28234
PATL1	0.237753194	4.74E-08	2.04E-07
PATL2	-0.329299969	1.72E-14	1.96E-13
PATZ1	-0.102650525	0.019806	0.033039
PAWR	0.438845287	1.18E-25	7.29E-24
PAX1	-0.060837043	0.168038	0.225298
PAX2	-0.04268031	0.333715	0.407496
PAX3	0.049505756	0.262108	0.331081
PAX4	0.059431623	0.178099	0.237117
PAX5	-0.148794677	0.000706	0.001544
PAX6	-0.216904549	6.71E-07	2.42E-06
PAX7	-0.108483089	0.013772	0.023755
PAX8	0.067909168	0.123771	0.171813
PAX9	0.198549441	5.62E-06	1.75E-05
PAXIP1	0.152539247	0.000514	0.001149
PBK	0.546685267	1.80E-41	1.91E-38
PBLD	-0.093107675	0.034652	0.054826
PBOV1	-0.226107432	2.15E-07	8.39E-07
PBRM1	-0.090938558	0.039114	0.061184
PBX1	-0.088944486	0.043636	0.067571
PBX2	-0.146524355	0.000853	0.001835
PBX3	-0.112700096	0.010482	0.018526
PBX4	-0.322737102	6.00E-14	6.27E-13
PBXIP1	-0.410163917	2.57E-22	9.41E-21
PCA3	-0.060509148	0.170347	0.227996
PCBD1	0.072297375	0.101245	0.14384
PCBD2	-0.176613183	5.58E-05	0.000147
PCBP1	0.248762148	1.05E-08	5.03E-08
PCBP2	0.32426597	4.50E-14	4.80E-13
PCBP3	-0.19437693	8.88E-06	2.68E-05
PCBP4	-0.090825472	0.039359	0.061525
PCCA	-0.14844793	0.000727	0.001586
PCCB	0.210972135	1.36E-06	4.67E-06
PCDH10	-0.160865427	0.000247	0.000586
PCDH11X	-0.303714514	1.89E-12	1.58E-11
PCDH11Y	-0.091372821	0.038184	0.05986
PCDH12	-0.126234606	0.004115	0.007841
PCDH15	-0.306179645	1.22E-12	1.06E-11

PCDH17	-0.128496651	0.003488	0.006729
PCDH18	-0.089382009	0.042608	0.066142
PCDH19	-0.175266032	6.37E-05	0.000167
PCDH1	-0.107535191	0.014626	0.025096
PCDH20	-0.396368451	8.03E-21	2.37E-19
PCDH7	0.032822799	0.457325	0.531283
PCDH8	-0.085843456	0.05154	0.078621
PCDH9	-0.178674831	4.55E-05	0.000122
PCDHA10	-0.206344621	2.33E-06	7.71E-06
PCDHA11	0.013197435	0.765106	0.810952
PCDHA12	-0.115995043	0.008417	0.015148
PCDHA13	-0.031162052	0.480417	0.554197
PCDHA1	0.132584928	0.002571	0.005082
PCDHA2	-0.026501524	0.548467	0.61877
PCDHA3	-0.17131642	9.33E-05	0.000239
PCDHA4	-0.025198701	0.568304	0.636766
PCDHA5	0.03059879	0.48839	0.561676
PCDHA6	-0.011272055	0.798576	0.83891
PCDHA7	0.020117503	0.648767	0.708803
PCDHA8	-0.075888249	0.085346	0.123526
PCDHA9	-0.030584183	0.488598	0.561851
PCDHAC1	-0.01326413	0.763954	0.810032
PCDHAC2	-0.171171757	9.46E-05	0.000242
PCDHB10	-0.037047383	0.401478	0.476105
PCDHB11	0.008609644	0.845461	0.877692
PCDHB12	-0.085639211	0.0521	0.079391
PCDHB13	0.059129489	0.180319	0.239562
PCDHB14	-0.050148841	0.255953	0.324537
PCDHB15	-0.117047197	0.007839	0.0142
PCDHB16	-0.046945846	0.287615	0.358563
PCDHB17	0.06085564	0.167908	0.225154
PCDHB18	-0.089447411	0.042456	0.065912
PCDHB19P	-0.057077543	0.195941	0.257392
PCDHB1	-0.216779849	6.81E-07	2.45E-06
PCDHB2	0.133968908	0.002314	0.004613
PCDHB3	0.01957285	0.657663	0.716955
PCDHB4	-0.192642001	1.07E-05	3.19E-05
PCDHB5	0.085933632	0.051294	0.078288
PCDHB6	0.033071973	0.453915	0.527983
PCDHB7	-0.110642797	0.011988	0.02091
PCDHB8	0.172372458	8.43E-05	0.000217
PCDHB9	-0.04403802	0.318552	0.391179
PCDHGA10	-0.123064419	0.005164	0.00968
PCDHGA11	-0.103491137	0.018814	0.031517

PCDHGA12	-0.151878739	0.000543	0.00121
PCDHGA1	0.128566142	0.00347	0.006699
PCDHGA2	-0.054776667	0.214609	0.278727
PCDHGA3	-0.076783949	0.081711	0.118799
PCDHGA4	-0.057043798	0.196206	0.257689
PCDHGA5	-0.168376824	0.000123	0.000309
PCDHGA6	-0.20205334	3.80E-06	1.21E-05
PCDHGA7	-0.040368246	0.360587	0.435335
PCDHGA8	0.035643154	0.419573	0.494249
PCDHGA9	-0.16248894	0.000213	0.000513
PCDHGB1	0.15315818	0.000487	0.001094
PCDHGB2	-0.071996972	0.102675	0.145562
PCDHGB3	-0.078163613	0.076359	0.11198
PCDHGB4	0.041416215	0.348243	0.42268
PCDHGB5	0.058986444	0.181377	0.240728
PCDHGB6	-0.173587794	7.50E-05	0.000194
PCDHGB7	-0.219902112	4.65E-07	1.73E-06
PCDHGB8P	-0.100036637	0.023185	0.038129
PCDHGC3	-0.192982178	1.03E-05	3.08E-05
PCDHGC4	-0.095461313	0.030308	0.048607
PCDHGC5	-0.038908761	0.378227	0.452993
PCDP1	-0.450295066	4.43E-27	3.28E-25
PCF11	-0.314363156	2.82E-13	2.68E-12
PCGEM1	0.018283131	0.67892	0.736234
PCGF1	0.069347839	0.115992	0.162311
PCGF2	-0.088101479	0.045677	0.070457
PCGF3	-0.162937096	0.000204	0.000494
PCGF5	0.13312813	0.002467	0.004892
PCGF6	0.351487099	2.02E-16	3.07E-15
PCID2	0.087609075	0.046905	0.072167
PCIF1	-0.044400306	0.314584	0.386876
PCK1	0.227021459	1.91E-07	7.53E-07
PCK2	0.034265651	0.437779	0.512044
PCLO	-0.13778255	0.001724	0.003514
PCM1	-0.099325759	0.024186	0.039596
PCMT1	0.196184378	7.29E-06	2.23E-05
PCMTD1	-0.04647666	0.292465	0.363735
PCMTD2	-0.153800125	0.000461	0.00104
PCNAP1	-0.039095711	0.375938	0.450738
PCNA	0.381699631	2.62E-19	6.27E-18
PCNP	0.164133688	0.000183	0.000446
PCNT	-0.171114482	9.52E-05	0.000243
PCNXL2	-0.146728068	0.000838	0.001807
PCNXL3	0.001665057	0.969931	0.977413

PCNX	-0.101577835	0.021137	0.035061
PCOLCE2	0.063289473	0.151511	0.205656
PCOLCE	0.011123957	0.801167	0.840704
PCOTH	-0.059923971	0.174525	0.232948
PCP2	-0.307039482	1.05E-12	9.18E-12
PCP4L1	-0.228373167	1.61E-07	6.41E-07
PCP4	0.029561529	0.503257	0.575979
PCSK1N	-0.173388462	7.65E-05	0.000198
PCSK1	0.299538319	3.89E-12	3.08E-11
PCSK2	-0.204293897	2.95E-06	9.58E-06
PCSK4	-0.220875738	4.13E-07	1.54E-06
PCSK5	-0.219859587	4.68E-07	1.74E-06
PCSK6	0.054028335	0.220947	0.285696
PCSK7	-0.237070551	5.19E-08	2.22E-07
PCSK9	0.000204454	0.996307	0.997535
PCTP	-0.040827859	0.35514	0.429667
PCYOX1L	-0.329068684	1.80E-14	2.05E-13
PCYOX1	-0.076538677	0.082694	0.120105
PCYT1A	0.122821132	0.005254	0.009833
PCYT1B	-0.170515896	0.000101	0.000256
PCYT2	-0.072319065	0.101142	0.143756
PC	-0.082177525	0.062388	0.093396
PDAP1	0.232835193	9.06E-08	3.76E-07
PDCD10	0.313380688	3.37E-13	3.17E-12
PDCD11	0.224640129	2.58E-07	9.97E-07
PDCD1LG2	0.013114133	0.766545	0.812133
PDCD1	-0.053224273	0.227906	0.293517
PDCD2L	0.334205234	6.65E-15	8.10E-14
PDCD2	0.286887087	3.25E-11	2.25E-10
PDCD4	-0.13340225	0.002417	0.004802
PDCD5	0.315373939	2.35E-13	2.26E-12
PDCD6IP	-0.006793677	0.877767	0.904558
PDCD6	0.194396284	8.86E-06	2.67E-05
PDCD7	-0.053925335	0.22183	0.286671
PDCL2	0.036682308	0.406137	0.480945
PDCL3	0.332001428	1.02E-14	1.20E-13
PDCL	0.169129228	0.000115	0.00029
PDC	0.027689601	0.530679	0.602203
PDDC1	-0.214250471	9.23E-07	3.26E-06
PDE10A	0.221109322	4.01E-07	1.50E-06
PDE11A	-0.084409166	0.05558	0.084124
PDE12	0.178577771	4.59E-05	0.000123
PDE1A	-0.090104166	0.040956	0.06385
PDE1B	-0.291417576	1.54E-11	1.12E-10

PDE1C	-0.039107531	0.375794	0.450646
PDE2A	-0.191038692	1.27E-05	3.75E-05
PDE3A	0.130229488	0.003068	0.005982
PDE3B	-0.050654649	0.251183	0.319298
PDE4A	-0.187430858	1.86E-05	5.34E-05
PDE4B	-0.106913742	0.015211	0.025994
PDE4C	-0.343153434	1.12E-15	1.54E-14
PDE4DIP	-0.188894227	1.59E-05	4.63E-05
PDE4D	0.058747846	0.183152	0.242682
PDE5A	-0.224352587	2.68E-07	1.03E-06
PDE6A	0.083197705	0.059195	0.089029
PDE6B	-0.276100442	1.83E-10	1.13E-09
PDE6C	-0.024442495	0.579974	0.646976
PDE6D	0.126455682	0.004049	0.007725
PDE6G	-0.158443817	0.000306	0.000714
PDE6H	-0.022264779	0.614189	0.678067
PDE7A	-0.102001269	0.020602	0.034248
PDE7B	-0.202022873	3.81E-06	1.22E-05
PDE8A	-0.154957433	0.000417	0.000947
PDE8B	-0.301994542	2.54E-12	2.08E-11
PDE9A	-0.188401897	1.68E-05	4.86E-05
PDF	0.29589428	7.25E-12	5.55E-11
PDGFA	-0.119930199	0.006433	0.011836
PDGFB	0.006609966	0.881048	0.906747
PDGFC	-0.059016048	0.181158	0.240469
PDGFD	-0.14749691	0.000787	0.001704
PDGFRA	-0.056476539	0.200699	0.262898
PDGFRB	-0.114535428	0.009282	0.016579
PDGFRL	0.079420335	0.071734	0.105841
PDHA1	0.057803404	0.190306	0.250962
PDHA2	0.032563218	0.460893	0.534823
PDHB	0.154396716	0.000437	0.000992
PDHX	0.302087265	2.50E-12	2.05E-11
PDIA2	0.061401965	0.164116	0.220578
PDIA3P	0.195293296	8.04E-06	2.44E-05
PDIA3	0.188824206	1.61E-05	4.66E-05
PDIA4	0.358549783	4.55E-17	7.65E-16
PDIA5	0.093636866	0.033631	0.053359
PDIA6	0.311519429	4.72E-13	4.33E-12
PDIK1L	-0.251795202	6.88E-09	3.36E-08
PDILT	-0.054456119	0.217308	0.281628
PDK1	0.269423237	5.15E-10	2.99E-09
PDK2	-0.449551588	5.51E-27	4.02E-25
PDK3	0.038025625	0.389154	0.464022

PDK4	-0.117489623	0.007607	0.013811
PDLIM1	-0.165787873	0.000157	0.000387
PDLIM2	-0.306788705	1.10E-12	9.57E-12
PDLIM3	-0.024502157	0.579049	0.646233
PDLIM4	-0.073191266	0.097083	0.138527
PDLIM5	0.117811885	0.007442	0.013534
PDLIM7	0.046524106	0.291972	0.363225
PDP1	0.027525432	0.533119	0.604458
PDP2	-0.06452925	0.14364	0.196273
PDPK1	-0.314537293	2.73E-13	2.60E-12
PDPN	-0.025120669	0.569503	0.637798
PDPR	-0.2287877	1.53E-07	6.10E-07
PDRG1	0.279665946	1.04E-10	6.70E-10
PDS5A	0.107106893	0.015027	0.025729
PDS5B	0.00378064	0.931794	0.946617
PDSS1	0.456872611	6.36E-28	5.17E-26
PDSS2	-0.012891496	0.770397	0.81535
PDX1	0.125605255	0.004306	0.008177
PDXDC1	-0.052166575	0.237295	0.303983
PDXDC2	-0.302969989	2.15E-12	1.78E-11
PDXK	-0.11049797	0.012101	0.021091
PDXP	0.165786961	0.000157	0.000387
PDYN	-0.085464219	0.052583	0.080055
PDZD11	0.266661623	7.82E-10	4.42E-09
PDZD2	-0.402018112	2.00E-21	6.54E-20
PDZD3	-0.018952228	0.667859	0.726042
PDZD4	-0.387692664	6.45E-20	1.65E-18
PDZD7	-0.054492712	0.216998	0.281336
PDZD8	0.157343079	0.000338	0.000781
PDZD9	-0.063269091	0.151643	0.205745
PDZK1IP1	-0.111578215	0.011281	0.01981
PDZK1P1	-0.222694241	3.30E-07	1.25E-06
PDZK1	-0.035273233	0.424418	0.499045
PDZRN3	-0.216768397	6.82E-07	2.46E-06
PDZRN4	-0.183094492	2.91E-05	8.08E-05
PEA15	-0.161835861	0.000226	0.000541
PEAR1	-0.215364448	8.08E-07	2.87E-06
PEBP1	-0.116845356	0.007947	0.014382
PEBP4	-0.355298456	9.08E-17	1.45E-15
PECAM1	-0.243070411	2.31E-08	1.05E-07
PECI	0.050364128	0.253915	0.322423
PECR	-0.034673547	0.432341	0.507019
PEF1	-0.212886326	1.09E-06	3.78E-06
PEG10	-0.071480422	0.105172	0.148658

PEG3AS	-0.065871635	0.135475	0.186339
PEG3	-0.215933265	7.54E-07	2.69E-06
PELI1	-0.126416399	0.004061	0.007744
PELI2	-0.163625513	0.000192	0.000466
PELI3	-0.143410906	0.001101	0.002324
PELO	0.131966058	0.002694	0.00531
PELP1	0.043581614	0.323599	0.396693
PEMT	0.029559227	0.50329	0.575979
PENK	-0.352098772	1.78E-16	2.73E-15
PEPD	-0.09112187	0.038719	0.060628
PER1	-0.239648081	3.68E-08	1.61E-07
PER2	-0.173225866	7.77E-05	0.000201
PER3	-0.430907969	1.07E-24	5.62E-23
PER4	-0.036595441	0.40725	0.482006
PERP	0.189667205	1.47E-05	4.30E-05
PES1	0.190815866	1.30E-05	3.83E-05
PET112L	0.155759704	0.000388	0.000888
PEX10	-8.20E-05	0.99852	0.99912
PEX11A	-0.095730641	0.029842	0.047948
PEX11B	-0.134089663	0.002293	0.004576
PEX11G	-0.270020515	4.70E-10	2.74E-09
PEX12	-0.02777338	0.529435	0.601236
PEX13	0.129609319	0.003213	0.006239
PEX14	-0.118888086	0.006913	0.012645
PEX16	-0.114328427	0.009411	0.01679
PEX19	-0.023121462	0.600623	0.665923
PEX1	0.056419413	0.201155	0.263462
PEX26	0.091021964	0.038934	0.060926
PEX2	0.165527065	0.000161	0.000396
PEX3	-0.003677224	0.933655	0.948147
PEX5L	-0.141079442	0.001328	0.002762
PEX5	0.180661272	3.73E-05	0.000102
PEX6	-0.037003391	0.402038	0.476684
PEX7	0.007412596	0.866731	0.895633
PF4V1	-0.01339749	0.761652	0.808063
PF4	0.116025675	0.0084	0.015122
PFAS	0.04196962	0.341834	0.416031
PFDN1	0.117540496	0.007581	0.013771
PFDN2	0.33175744	1.07E-14	1.26E-13
PFDN4	0.407151628	5.52E-22	1.94E-20
PFDN5	0.112069957	0.010924	0.01924
PFDN6	0.289336001	2.17E-11	1.54E-10
PFKFB1	-0.121105674	0.005927	0.010977
PFKFB2	-0.217823002	6.00E-07	2.19E-06

PFKFB3	-0.143934051	0.001055	0.002233
PFKFB4	0.158586144	0.000303	0.000706
PFKL	-0.236806225	5.37E-08	2.30E-07
PFKM	0.069192881	0.116811	0.163308
PFKP	0.362964606	1.76E-17	3.15E-16
PFN1	0.248990554	1.02E-08	4.89E-08
PFN2	0.410923761	2.12E-22	7.79E-21
PFN3	0.021308277	0.629492	0.691653
PFN4	-0.031601528	0.474245	0.547937
PGA3	-0.155806978	0.000387	0.000884
PGA4	-0.074719463	0.090284	0.129827
PGA5	-0.120474126	0.006194	0.011429
PGAM1	0.415523359	6.46E-23	2.62E-21
PGAM2	-0.088377957	0.044999	0.069526
PGAM4	0.380748641	3.27E-19	7.65E-18
PGAM5	0.502491163	2.66E-34	6.73E-32
PGAP1	-0.13490556	0.002154	0.004321
PGAP2	-0.013098805	0.76681	0.812328
PGAP3	-0.32591297	3.29E-14	3.60E-13
PGBD1	0.037324697	0.397961	0.472663
PGBD2	-0.174986053	6.54E-05	0.000171
PGBD3	0.022736041	0.60671	0.671442
PGBD4	-0.109195137	0.013159	0.022785
PGBD5	-0.161687905	0.000229	0.000548
PGCP	-0.29111519	1.62E-11	1.17E-10
PGC	-0.228907883	1.50E-07	6.02E-07
PGD	0.27764419	1.44E-10	9.04E-10
PGF	0.135678799	0.00203	0.004091
PGGT1B	0.005079652	0.908448	0.92842
PGK1	0.368394705	5.34E-18	1.04E-16
PGK2	0.140302434	0.001413	0.002925
PGLS	-0.023661475	0.592142	0.658126
PGLYRP1	-0.09639222	0.028723	0.046303
PGLYRP2	-0.02325747	0.598482	0.663954
PGLYRP3	-0.061116835	0.166087	0.222967
PGLYRP4	-0.115814713	0.00852	0.015308
PGM1	0.136832732	0.001856	0.003763
PGM2L1	0.169420766	0.000112	0.000282
PGM2	0.2196205	4.82E-07	1.79E-06
PGM3	0.386619989	8.31E-20	2.10E-18
PGM5P2	-0.200984258	4.29E-06	1.36E-05
PGM5	-0.294906967	8.57E-12	6.49E-11
PGPEP1L	-0.075436586	0.087228	0.12594
PGPEP1	-0.454912055	1.14E-27	9.08E-26

PGP	0.167157527	0.000138	0.000344
PGRMC1	-0.101362476	0.021413	0.035478
PGRMC2	0.091881198	0.037118	0.058355
PGR	-0.320897579	8.46E-14	8.64E-13
PGS1	-0.33991957	2.14E-15	2.83E-14
PHACTR1	-0.400956793	2.60E-21	8.32E-20
PHACTR2	-0.186661792	2.01E-05	5.75E-05
PHACTR3	-0.15349361	0.000473	0.001064
PHACTR4	-0.180409635	3.82E-05	0.000104
PHAX	0.107456073	0.014699	0.025213
PHB2	0.360247727	3.16E-17	5.49E-16
PHB	0.300663809	3.20E-12	2.57E-11
PHC1	-0.219408537	4.94E-07	1.83E-06
PHC2	-0.033942749	0.442111	0.516446
PHC3	-0.288627879	2.44E-11	1.72E-10
PHEX	0.042426891	0.336596	0.410553
PHF10	-0.084646383	0.054894	0.083224
PHF11	-0.130418336	0.003025	0.005904
PHF12	-0.238312936	4.40E-08	1.91E-07
PHF13	-0.027761739	0.529608	0.601398
PHF14	0.210651738	1.41E-06	4.84E-06
PHF15	-0.275522915	2.01E-10	1.23E-09
PHF16	0.129491301	0.003241	0.00629
PHF17	0.032187097	0.46609	0.539914
PHF19	0.14598532	0.000892	0.001911
PHF1	-0.344892078	7.86E-16	1.10E-14
PHF20L1	0.199910196	4.83E-06	1.52E-05
PHF20	-0.02099115	0.634602	0.696028
PHF21A	-0.24955992	9.43E-09	4.53E-08
PHF21B	-0.084991056	0.05391	0.081894
PHF23	0.249612744	9.36E-09	4.50E-08
PHF2	-0.303482707	1.96E-12	1.64E-11
PHF3	-0.089954435	0.041294	0.064337
PHF5A	0.279796496	1.02E-10	6.57E-10
PHF6	0.191558901	1.20E-05	3.56E-05
PHF7	-0.270171547	4.59E-10	2.68E-09
PHF8	-0.004899662	0.911679	0.930914
PHGDH	0.107870157	0.014319	0.024611
PHGR1	0.109039284	0.013291	0.022979
PHIP	-0.193632111	9.62E-06	2.89E-05
PHKA1	0.312881594	3.69E-13	3.45E-12
PHKA2	-0.255175668	4.25E-09	2.14E-08
PHKB	-0.233965943	7.81E-08	3.26E-07
PHKG1	-0.286850719	3.27E-11	2.26E-10

PHKG2	-0.223615153	2.94E-07	1.12E-06
PHLDA1	0.025895917	0.557646	0.627108
PHLDA2	0.081095264	0.06593	0.098124
PHLDA3	-0.270582869	4.31E-10	2.53E-09
PHLDB1	-0.326875099	2.74E-14	3.03E-13
PHLDB2	0.028326201	0.521269	0.593277
PHLDB3	-0.15575726	0.000388	0.000888
PHLPP1	0.064506905	0.143779	0.19645
PHLPP2	-0.033012818	0.454724	0.528769
PHOSPHO1	-0.174260309	7.02E-05	0.000183
PHOSPHO2	-0.024585708	0.577755	0.645149
PHOX2A	0.10148227	0.021259	0.035255
PHOX2B	0.055932932	0.205074	0.267874
PHPT1	0.040281827	0.361617	0.436287
PHRF1	-0.171455825	9.21E-05	0.000236
PHTF1	-0.073723896	0.094668	0.135382
PHTF2	0.140964866	0.00134	0.002784
PHYHD1	-0.239513992	3.74E-08	1.64E-07
PHYHIPL	-0.018187024	0.680515	0.737723
PHYHIP	-0.118401793	0.007147	0.013041
PHYH	0.040270858	0.361747	0.436341
PI15	0.259054181	2.42E-09	1.26E-08
PI16	-0.368757958	4.93E-18	9.65E-17
PI3	0.206710774	2.24E-06	7.40E-06
PI4K2A	-0.079231788	0.072413	0.106701
PI4K2B	0.111380287	0.011427	0.020046
PI4KAP1	-0.240443006	3.30E-08	1.46E-07
PI4KAP2	-0.150641805	0.000604	0.001335
PI4KA	-0.273613303	2.70E-10	1.63E-09
PI4KB	-0.168771595	0.000119	0.000299
PIAS1	-0.10921982	0.013139	0.022755
PIAS2	0.040276027	0.361686	0.436293
PIAS3	-0.079943097	0.069879	0.103337
PIAS4	-0.025314421	0.566529	0.635168
PIBF1	-0.089714251	0.041841	0.065098
PICALM	0.136441664	0.001913	0.003869
PICK1	-0.112240952	0.010803	0.019047
PID1	-0.264096895	1.15E-09	6.33E-09
PIF1	0.286025955	3.74E-11	2.56E-10
PIGA	-0.149809125	0.000648	0.001426
PIGB	-0.037319478	0.398027	0.472685
PIGC	0.259401132	2.30E-09	1.20E-08
PIGF	0.229886256	1.33E-07	5.36E-07
PIGG	-0.003754118	0.932271	0.947006

PIGH	0.095558443	0.030139	0.048391
PIGK	0.094894796	0.031308	0.050043
PIGL	-0.132524076	0.002583	0.005104
PIGM	0.017698355	0.688645	0.745162
PIGN	0.116350049	0.008218	0.014826
PIGO	0.104085105	0.01814	0.030501
PIGP	0.004580095	0.917418	0.935196
PIGQ	-0.345232001	7.34E-16	1.03E-14
PIGR	-0.412857753	1.29E-22	4.94E-21
PIGS	-0.095119096	0.030909	0.04946
PIGT	0.065696431	0.13652	0.187596
PIGU	0.197186195	6.53E-06	2.01E-05
PIGV	-0.133131319	0.002467	0.004892
PIGW	0.199681198	4.96E-06	1.55E-05
PIGX	0.159105535	0.000289	0.000677
PIGY	0.059534605	0.177347	0.236289
PIGZ	-0.124478593	0.004669	0.008821
PIH1D1	0.09380061	0.033321	0.052909
PIH1D2	-0.16781509	0.00013	0.000325
PIK3AP1	-0.064095091	0.14636	0.199485
PIK3C2A	-0.027801244	0.529022	0.601033
PIK3C2B	-0.212331236	1.16E-06	4.02E-06
PIK3C2G	0.042233171	0.338809	0.412909
PIK3C3	0.058820748	0.182609	0.242041
PIK3CA	0.07994528	0.069872	0.103337
PIK3CB	0.006898205	0.875902	0.903072
PIK3CD	-0.307043937	1.05E-12	9.17E-12
PIK3CG	-0.179929988	4.01E-05	0.000109
PIK3IP1	-0.475602379	2.00E-30	2.39E-28
PIK3R1	-0.380787987	3.24E-19	7.60E-18
PIK3R2	-0.048818902	0.268794	0.338463
PIK3R3	-0.207144853	2.13E-06	7.07E-06
PIK3R4	0.039830791	0.367022	0.441661
PIK3R5	-0.302452239	2.35E-12	1.93E-11
PIK3R6	-0.333839729	7.14E-15	8.64E-14
PIKFYVE	-0.202153734	3.76E-06	1.20E-05
PILRA	-0.162978891	0.000204	0.000492
PILRB	-0.271374678	3.82E-10	2.25E-09
PIM1	-0.038717651	0.380575	0.455178
PIM2	-0.050597276	0.251721	0.31986
PIM3	-0.01483472	0.736981	0.787111
PIN1L	0.068032426	0.123089	0.171068
PIN1	-0.114009627	0.009613	0.01713
PIN4	0.03167232	0.473255	0.546977

PINK1	-0.278734526	1.21E-10	7.69E-10
PINX1	0.190835795	1.30E-05	3.83E-05
PION	-0.371479519	2.69E-18	5.45E-17
PIP4K2A	-0.009434875	0.830862	0.865368
PIP4K2B	-0.246277173	1.49E-08	6.94E-08
PIP4K2C	0.180302471	3.86E-05	0.000105
PIP5K1A	0.155553773	0.000395	0.000902
PIP5K1B	-0.325433273	3.61E-14	3.92E-13
PIP5K1C	-0.235600062	6.30E-08	2.67E-07
PIP5K1P1	0.053090238	0.229081	0.294878
PIP5KL1	-0.217875982	5.96E-07	2.17E-06
PIPOX	0.004941942	0.91092	0.930376
PIPSL	0.243096192	2.31E-08	1.04E-07
PIP	-0.176233333	5.79E-05	0.000153
PIRT	-0.038730619	0.380415	0.455096
PIR	0.13497694	0.002143	0.004301
PISD	-0.130724671	0.002957	0.005783
PITPNA	-0.142605992	0.001175	0.002468
PITPNB	0.274944887	2.19E-10	1.34E-09
PITPNC1	0.352204169	1.74E-16	2.67E-15
PITPNM1	-0.020364747	0.644745	0.705257
PITPNM2	0.005688399	0.897534	0.919913
PITPNM3	-0.3364875	4.24E-15	5.34E-14
PITRM1	0.132698102	0.002549	0.005044
PITX1	0.277224645	1.54E-10	9.61E-10
PITX2	0.203596171	3.19E-06	1.03E-05
PITX3	0.267151563	7.26E-10	4.13E-09
PIWIL1	0.053252398	0.22766	0.293219
PIWIL2	0.024382875	0.580898	0.647827
PIWIL3	0.027041711	0.540343	0.611218
PIWIL4	-0.161614077	0.000231	0.000551
PJA1	-0.019639303	0.656575	0.715964
PJA2	-0.096409923	0.028694	0.046267
PKD1L1	-0.085246908	0.053189	0.080895
PKD1L2	0.106606932	0.015507	0.026437
PKD1L3	-0.186294714	2.09E-05	5.96E-05
PKD1	-0.384317962	1.43E-19	3.55E-18
PKD2L1	-0.092921007	0.035018	0.055357
PKD2L2	-0.147917009	0.000759	0.001651
PKD2	-0.309707219	6.54E-13	5.90E-12
PKDCC	-0.075762335	0.085867	0.124155
PKDREJ	-0.195973529	7.46E-06	2.27E-05
PKHD1L1	-0.240147613	3.44E-08	1.52E-07
PKHD1	-0.179070218	4.37E-05	0.000118

PKIA	-0.049650757	0.260712	0.32953
PKIB	0.199238647	5.21E-06	1.63E-05
PKIG	0.040860981	0.354749	0.429351
PKLR	-0.003999173	0.927862	0.943486
PKM2	0.278370621	1.28E-10	8.12E-10
PKMYT1	0.382257462	2.31E-19	5.55E-18
PKN1	-0.028326797	0.52126	0.593277
PKN2	0.24398432	2.04E-08	9.32E-08
PKN3	-0.012427419	0.778443	0.821733
PKNOX1	-0.029103224	0.509901	0.582322
PKNOX2	-0.352295253	1.71E-16	2.63E-15
PKP1	0.059449815	0.177966	0.236972
PKP2	0.248118287	1.15E-08	5.46E-08
PKP3	0.156842814	0.000353	0.000813
PKP4	-0.069416632	0.11563	0.161872
PL-5283	0.33690519	3.91E-15	4.95E-14
PLA1A	-0.211486853	1.28E-06	4.42E-06
PLA2G10	-0.20840889	1.84E-06	6.19E-06
PLA2G12A	-0.019452439	0.659637	0.718754
PLA2G12B	-0.149291632	0.000677	0.001485
PLA2G15	-0.236394141	5.68E-08	2.42E-07
PLA2G16	-0.216433963	7.10E-07	2.55E-06
PLA2G1B	-0.311439207	4.79E-13	4.39E-12
PLA2G2A	-0.08657351	0.049579	0.075937
PLA2G2C	-0.072035664	0.10249	0.14532
PLA2G2D	-0.103994316	0.018242	0.030658
PLA2G2F	0.029760618	0.500385	0.573327
PLA2G3	-0.236088805	5.91E-08	2.51E-07
PLA2G4A	0.18088504	3.64E-05	9.95E-05
PLA2G4C	-0.272868812	3.03E-10	1.81E-09
PLA2G4D	-0.069770795	0.113779	0.159599
PLA2G4E	-0.154973395	0.000416	0.000946
PLA2G4F	-0.418508997	2.96E-23	1.27E-21
PLA2G5	-0.103687202	0.018589	0.031184
PLA2G6	-0.272879327	3.03E-10	1.81E-09
PLA2G7	-0.042502232	0.335738	0.409641
PLA2R1	-0.101126688	0.021719	0.035926
PLAA	0.200444185	4.55E-06	1.44E-05
PLAC1L	0.06319643	0.152115	0.206284
PLAC1	0.228451459	1.59E-07	6.35E-07
PLAC2	-0.101880813	0.020753	0.034476
PLAC4	-0.001889026	0.965889	0.97388
PLAC8L1	-0.038833699	0.379148	0.453851
PLAC8	-0.189854449	1.44E-05	4.22E-05

PLAC9	-0.322259642	6.56E-14	6.83E-13
PLAG1	-0.002841561	0.948708	0.959995
PLAGL1	-0.165358274	0.000164	0.000402
PLAGL2	-0.075369984	0.087508	0.126299
PLAT	-0.130529478	0.003	0.005858
PLAUR	0.179907527	4.02E-05	0.000109
PLAU	0.094591419	0.031856	0.050816
PLB1	-0.233047494	8.81E-08	3.66E-07
PLBD1	-0.024764944	0.574984	0.642665
PLBD2	0.078962889	0.07339	0.107973
PLCB1	0.17173176	8.97E-05	0.00023
PLCB2	-0.346212601	6.00E-16	8.58E-15
PLCB3	0.009960332	0.821596	0.857773
PLCB4	-0.165217414	0.000166	0.000407
PLCD1	-0.389605914	4.09E-20	1.09E-18
PLCD3	0.140514222	0.001389	0.00288
PLCD4	-0.186690673	2.01E-05	5.74E-05
PLCE1	-0.248854822	1.04E-08	4.97E-08
PLCG1	-0.250218831	8.59E-09	4.15E-08
PLCG2	-0.31559216	2.25E-13	2.18E-12
PLCH1	-0.234746673	7.05E-08	2.97E-07
PLCH2	-0.381273253	2.90E-19	6.87E-18
PLCL1	-0.304765171	1.57E-12	1.33E-11
PLCL2	-0.197757541	6.14E-06	1.90E-05
PLCXD1	-0.10413395	0.018086	0.030418
PLCXD2	0.025336901	0.566184	0.634941
PLCXD3	-0.210484785	1.44E-06	4.93E-06
PLCZ1	-0.007816689	0.859539	0.889495
PLD1	0.184270241	2.58E-05	7.23E-05
PLD2	-0.299196822	4.13E-12	3.26E-11
PLD3	-0.236399934	5.67E-08	2.42E-07
PLD4	-0.416775361	4.66E-23	1.92E-21
PLD5	-0.153621419	0.000468	0.001054
PLD6	-0.11768145	0.007508	0.013645
PLDN	0.080455944	0.068098	0.101012
PLEC	0.011622097	0.792462	0.833626
PLEK2	0.31847584	1.33E-13	1.32E-12
PLEKHA1	0.17589174	5.99E-05	0.000158
PLEKHA2	-0.216470909	7.07E-07	2.54E-06
PLEKHA3	0.076386688	0.083308	0.120864
PLEKHA4	-0.288750336	2.39E-11	1.69E-10
PLEKHA5	0.030114814	0.495297	0.568541
PLEKHA6	-0.059567561	0.177107	0.236001
PLEKHA7	-0.223811364	2.87E-07	1.10E-06

PLEKHA8	0.178064718	4.83E-05	0.000129
PLEKHA9	0.169047751	0.000116	0.000292
PLEKHB1	-0.213626773	9.94E-07	3.49E-06
PLEKHB2	0.14623823	0.000873	0.001875
PLEKHF1	-0.09478887	0.031498	0.050319
PLEKHF2	0.080861329	0.066716	0.099176
PLEKHG1	-0.303472517	1.97E-12	1.64E-11
PLEKHG2	0.177738423	4.99E-05	0.000133
PLEKHG3	-0.000767613	0.986135	0.989601
PLEKHG4B	-0.159432883	0.000281	0.00066
PLEKHG4	-0.197743695	6.15E-06	1.90E-05
PLEKHG5	-0.077512564	0.078848	0.115155
PLEKHG6	0.046802289	0.289093	0.360137
PLEKHG7	-0.252850335	5.92E-09	2.93E-08
PLEKHH1	0.142616511	0.001174	0.002466
PLEKHH2	-0.460762589	1.98E-28	1.72E-26
PLEKHH3	-0.251038801	7.66E-09	3.73E-08
PLEKHJ1	0.010894863	0.805179	0.84416
PLEKHM1P	-0.484816534	1.02E-31	1.56E-29
PLEKHM1	-0.41482645	7.74E-23	3.08E-21
PLEKHM2	-0.279589627	1.06E-10	6.77E-10
PLEKHM3	-0.300366935	3.37E-12	2.70E-11
PLEKHN1	-0.101770024	0.020893	0.034696
PLEKHO1	-0.023423206	0.595877	0.661884
PLEKHO2	-0.160571367	0.000253	0.000601
PLEK	-0.156813216	0.000354	0.000815
PLGLA	0.016626418	0.706603	0.761297
PLGLB2	-0.250093422	8.75E-09	4.22E-08
PLG	-0.030875156	0.484469	0.558163
PLIN1	-0.264144609	1.14E-09	6.28E-09
PLIN2	0.201919561	3.86E-06	1.23E-05
PLIN3	0.260883425	1.85E-09	9.84E-09
PLIN4	-0.202630142	3.56E-06	1.14E-05
PLIN5	-0.383039807	1.92E-19	4.66E-18
PLK1S1	-0.202230069	3.73E-06	1.19E-05
PLK1	0.526595323	4.42E-38	2.32E-35
PLK2	-0.036108262	0.413527	0.488237
PLK3	-0.270433425	4.41E-10	2.58E-09
PLK4	0.474742807	2.62E-30	3.08E-28
PLK5P	-0.034972155	0.428385	0.50294
PLLP	-0.377671305	6.63E-19	1.48E-17
PLN	0.017250804	0.696122	0.75195
PLOD1	0.17696245	5.39E-05	0.000143
PLOD2	0.362008763	2.16E-17	3.82E-16

PLOD3	0.204166204	2.99E-06	9.71E-06
PLP1	-0.251594571	7.08E-09	3.46E-08
PLP2	0.194349472	8.90E-06	2.68E-05
PLRG1	0.181549797	3.41E-05	9.35E-05
PLS1	0.076534701	0.08271	0.120119
PLS3	-0.059930366	0.174479	0.232902
PLSCR1	0.102542205	0.019937	0.033225
PLSCR2	0.058126232	0.187838	0.248215
PLSCR3	-0.071702187	0.104094	0.147291
PLSCR4	-0.291221038	1.59E-11	1.15E-10
PLSCR5	0.07944689	0.071639	0.105716
PLTP	0.079023492	0.073169	0.107664
PLUNC	0.123763301	0.004914	0.009243
PLVAP	0.027246565	0.537278	0.608103
PLXDC1	-0.308872746	7.59E-13	6.77E-12
PLXDC2	-0.191001873	1.28E-05	3.76E-05
PLXNA1	-0.075791494	0.085746	0.123998
PLXNA2	-0.346713426	5.42E-16	7.83E-15
PLXNA3	-0.110639048	0.011991	0.020913
PLXNA4	-0.080786392	0.06697	0.099509
PLXNB1	-0.349175337	3.26E-16	4.86E-15
PLXNB2	-0.355327824	9.02E-17	1.44E-15
PLXNB3	-0.150491896	0.000612	0.001351
PLXNC1	-0.187310031	1.88E-05	5.40E-05
PLXND1	-0.2927688	1.23E-11	9.06E-11
PM20D1	-0.264293299	1.12E-09	6.16E-09
PM20D2	0.189575192	1.48E-05	4.34E-05
PMAIP1	0.261786153	1.62E-09	8.69E-09
PMCHL1	0.042180567	0.339412	0.413492
PMCHL2	-0.005533224	0.900314	0.922088
PMCH	0.029139914	0.509367	0.581851
PMEPA1	0.031169751	0.480308	0.554104
PMF1	-0.060216046	0.17243	0.23046
PMFBP1	-0.081632807	0.064151	0.095715
PML	-0.14167747	0.001266	0.002644
PMM1	-0.227151587	1.88E-07	7.42E-07
PMM2	0.176541487	5.62E-05	0.000148
PMP22	-0.110690378	0.011951	0.020852
PMP2	-0.148047851	0.000751	0.001636
PMPCA	0.093332779	0.034214	0.054203
PMPCB	0.145442789	0.000932	0.001992
PMS1	0.111250019	0.011525	0.020193
PMS2CL	-0.046475925	0.292473	0.363735
PMS2L11	-0.274281923	2.43E-10	1.48E-09

PMS2L1	0.234174687	7.60E-08	3.18E-07
PMS2L2	-0.146897915	0.000827	0.001784
PMS2L3	-0.153075087	0.00049	0.001101
PMS2L4	-0.103963669	0.018276	0.030711
PMS2L5	0.109056563	0.013277	0.022956
PMS2	0.020062707	0.64966	0.709585
PMVK	-0.150743203	0.000599	0.001324
PNCK	0.050834526	0.249502	0.317435
PNKD	-0.096533321	0.028489	0.045963
PNKP	-0.064377896	0.144584	0.197414
PNLDC1	-0.122194112	0.005491	0.010244
PNLIPRP1	-0.057380557	0.193574	0.254667
PNLIPRP2	-0.006344459	0.885792	0.910388
PNLIPRP3	0.032546012	0.46113	0.535005
PNLIP	-0.004696929	0.915319	0.933724
PNMA1	0.330005312	1.50E-14	1.73E-13
PNMA2	-0.333146095	8.18E-15	9.77E-14
PNMA3	-0.199102349	5.29E-06	1.65E-05
PNMA5	-0.072306613	0.101201	0.143819
PNMA6A	-0.147630564	0.000778	0.001687
PNMAL1	-0.218537016	5.50E-07	2.02E-06
PNMAL2	-0.402703096	1.69E-21	5.58E-20
PNMT	-0.236910152	5.30E-08	2.27E-07
PNN	-0.01318401	0.765338	0.811112
PNO1	0.467381935	2.62E-29	2.58E-27
PNOC	-0.051840358	0.240244	0.307269
PNPLA1	0.162206803	0.000218	0.000525
PNPLA2	-0.286475933	3.48E-11	2.39E-10
PNPLA3	-0.001471093	0.973433	0.980151
PNPLA4	-0.097599235	0.026775	0.043471
PNPLA5	0.116490417	0.008141	0.014703
PNPLA6	-0.213228523	1.04E-06	3.65E-06
PNPLA7	-0.371892411	2.45E-18	5.01E-17
PNPLA8	0.15955535	0.000278	0.000653
PNPO	0.112579324	0.010565	0.018664
PNPT1	0.357475319	5.72E-17	9.50E-16
PNP	0.425091191	5.15E-24	2.48E-22
PNRC1	-0.119951365	0.006423	0.011821
PNRC2	-0.185886171	2.18E-05	6.20E-05
POC1A	0.460908609	1.89E-28	1.66E-26
POC1B	0.378425217	5.58E-19	1.26E-17
POC5	-0.07433344	0.091964	0.131892
PODNL1	-0.065301821	0.138896	0.190533
PODN	-0.343566347	1.03E-15	1.42E-14

PODXL2	-0.070571756	0.10968	0.154387
PODXL	-0.115676331	0.0086	0.015437
POF1B	-0.004799658	0.913474	0.932366
POFUT1	0.271158788	3.94E-10	2.32E-09
POFUT2	-0.184744045	2.46E-05	6.91E-05
POGK	0.005366728	0.903299	0.92434
POGZ	-0.136899898	0.001846	0.003744
POLA1	0.077994351	0.077	0.112769
POLA2	0.32026038	9.53E-14	9.67E-13
POLB	0.114424901	0.009351	0.016688
POLD1	0.036375139	0.410082	0.484754
POLD2	0.279496332	1.07E-10	6.86E-10
POLD3	0.183538288	2.78E-05	7.75E-05
POLD4	0.093306603	0.034265	0.054266
POLDIP2	0.222331691	3.45E-07	1.30E-06
POLDIP3	-0.194483133	8.78E-06	2.65E-05
POLE2	0.576475742	6.10E-47	1.52E-43
POLE3	0.297728451	5.31E-12	4.13E-11
POLE4	0.19570493	7.68E-06	2.34E-05
POLE	0.126741127	0.003966	0.00758
POLG2	-0.006989131	0.874279	0.901847
POLG	-0.147716648	0.000772	0.001676
POLH	-0.304821974	1.55E-12	1.32E-11
POLI	-0.216096211	7.39E-07	2.65E-06
POLK	-0.167666985	0.000132	0.000329
POLL	-0.121267365	0.005861	0.010865
POLM	-0.180103136	3.94E-05	0.000107
POLN	-0.196947642	6.71E-06	2.06E-05
POLQ	0.360391246	3.06E-17	5.32E-16
POLR1A	0.158387092	0.000308	0.000718
POLR1B	0.305169439	1.46E-12	1.25E-11
POLR1C	0.243841301	2.08E-08	9.50E-08
POLR1D	0.131325394	0.002827	0.005546
POLR1E	0.171730117	8.97E-05	0.00023
POLR2A	-0.080202527	0.068974	0.102167
POLR2B	0.213625027	9.94E-07	3.49E-06
POLR2C	-0.028203075	0.523082	0.595069
POLR2D	0.286415854	3.51E-11	2.42E-10
POLR2E	-0.069779129	0.113736	0.159567
POLR2F	0.181763904	3.33E-05	9.16E-05
POLR2G	0.246461838	1.45E-08	6.78E-08
POLR2H	0.250131355	8.70E-09	4.20E-08
POLR2I	0.072259452	0.101425	0.144034
POLR2J2	-0.046953897	0.287532	0.358505

POLR2J3	-0.122547241	0.005356	0.010011
POLR2J4	-0.181615086	3.38E-05	9.29E-05
POLR2J	0.195552229	7.81E-06	2.38E-05
POLR2K	0.338418734	2.89E-15	3.75E-14
POLR2L	-0.052993988	0.229927	0.295877
POLR3A	0.232683595	9.24E-08	3.83E-07
POLR3B	0.128172087	0.003572	0.006876
POLR3C	0.162020125	0.000222	0.000533
POLR3D	0.222087815	3.55E-07	1.34E-06
POLR3E	0.033014187	0.454705	0.528769
POLR3F	0.181088153	3.57E-05	9.77E-05
POLR3GL	-0.32326554	5.43E-14	5.71E-13
POLR3G	0.4331823	5.70E-25	3.18E-23
POLR3H	-0.139213979	0.00154	0.003167
POLR3K	0.179328981	4.26E-05	0.000115
POLRMT	-0.046707487	0.290072	0.361221
POM121C	-0.021292832	0.629741	0.691836
POM121L10P	-0.27040666	4.43E-10	2.59E-09
POM121L1P	-0.295613426	7.60E-12	5.81E-11
POM121L2	-0.065933498	0.135108	0.185936
POM121L4P	-0.205985931	2.43E-06	8.01E-06
POM121L8P	-0.300118992	3.52E-12	2.81E-11
POM121L9P	-0.348930461	3.43E-16	5.09E-15
POM121	-0.073064613	0.097664	0.139287
POMC	-0.011394908	0.796429	0.836962
POMGNT1	-0.112047678	0.01094	0.019263
POMP	0.332429853	9.40E-15	1.11E-13
POMT1	-0.271422037	3.79E-10	2.24E-09
POMT2	0.058956611	0.181598	0.240958
POMZP3	-0.080805122	0.066907	0.099422
PON1	-0.194416706	8.84E-06	2.67E-05
PON2	0.02268355	0.607541	0.672064
PON3	-0.118480628	0.007109	0.012975
POP1	0.39076218	3.11E-20	8.39E-19
POP4	0.147603127	0.00078	0.001691
POP5	0.167515476	0.000134	0.000333
POP7	0.375524325	1.08E-18	2.34E-17
POPDC2	-0.302865804	2.19E-12	1.81E-11
POPDC3	0.298985436	4.28E-12	3.36E-11
PORCN	-0.012588399	0.775649	0.81952
POR	-0.058563489	0.184533	0.2443
POSTN	0.224856559	2.51E-07	9.72E-07
POT1	0.154531819	0.000432	0.000981
POTEA	0.000854458	0.984567	0.988375

POTEB	-0.047168497	0.285332	0.356251
POTEC	-0.012838252	0.771319	0.816023
POTED	0.055302941	0.21023	0.273733
POTEE	0.139226817	0.001539	0.003164
POTEF	0.144255351	0.001027	0.002182
POTEG	0.066244203	0.133274	0.183627
POTEH	0.067947981	0.123556	0.171574
POU1F1	-0.02876828	0.514785	0.587168
POU2AF1	-0.084666664	0.054836	0.083149
POU2F1	0.037587573	0.394644	0.469504
POU2F2	-0.088995807	0.043515	0.067403
POU2F3	-0.318581223	1.30E-13	1.30E-12
POU3F1	-0.279775177	1.03E-10	6.59E-10
POU3F2	-0.024933058	0.572391	0.640519
POU3F3	-0.026901463	0.542446	0.613189
POU3F4	0.035473598	0.421789	0.496392
POU4F1	0.078154336	0.076394	0.112004
POU4F2	-0.03080784	0.485422	0.559003
POU4F3	-0.004703435	0.915202	0.933701
POU5F1B	-0.197330603	6.43E-06	1.98E-05
POU5F1	-0.241281867	2.95E-08	1.31E-07
POU5F2	-0.154267867	0.000442	0.001002
POU6F1	-0.373778836	1.60E-18	3.37E-17
POU6F2	0.103019335	0.019365	0.032361
PP14571	0.03101264	0.482525	0.55628
PPA1	0.213638331	9.93E-07	3.49E-06
PPA2	0.162518555	0.000212	0.000512
PPAN-P2RY11	-0.125660306	0.004289	0.008147
PPAN	0.139761597	0.001475	0.003044
PPAP2A	-0.044758951	0.310687	0.382767
PPAP2B	-0.222019932	3.58E-07	1.35E-06
PPAP2C	0.059410852	0.178251	0.237256
PPAPDC1A	0.265992625	8.65E-10	4.86E-09
PPAPDC1B	0.01691578	0.701738	0.75724
PPAPDC2	0.044657082	0.31179	0.383764
PPAPDC3	-0.118698379	0.007003	0.012798
PPARA	-0.211752156	1.24E-06	4.29E-06
PPARD	-0.044658355	0.311776	0.383764
PPARGC1A	0.021243956	0.630527	0.692357
PPARGC1B	-0.181945106	3.27E-05	9.00E-05
PPARG	0.01417642	0.748249	0.796719
PPAT	0.460746649	1.99E-28	1.72E-26
PPBPL2	0.010961574	0.80401	0.843196
PPBP	-0.007313623	0.868494	0.897223

PPCDC	0.026356817	0.550653	0.620852
PPCS	0.093763336	0.033391	0.053
PPDPF	-0.146388283	0.000862	0.001854
PPEF1	0.250769894	7.95E-09	3.86E-08
PPEF2	-0.129777889	0.003173	0.00617
PPFIA1	0.085228642	0.053241	0.080957
PPFIA2	-0.171311757	9.34E-05	0.000239
PPFIA3	0.0459531	0.297942	0.36925
PPFIA4	-0.107249977	0.014892	0.025521
PPFIBP1	0.091185003	0.038584	0.060443
PPFIBP2	-0.454959047	1.12E-27	8.99E-26
PPHLN1	0.286787617	3.31E-11	2.28E-10
PPIAL4C	0.373320637	1.78E-18	3.69E-17
PPIAL4D	0.062775464	0.154868	0.209567
PPIAL4E	0.042774967	0.332643	0.406361
PPIAL4G	0.312956519	3.64E-13	3.41E-12
PPIA	0.343199917	1.11E-15	1.52E-14
PPIB	0.217804987	6.01E-07	2.19E-06
PPIC	0.047480049	0.282159	0.35284
PPID	0.408477636	3.95E-22	1.42E-20
PPIEL	-0.3279824	2.22E-14	2.48E-13
PPIE	-0.029201017	0.508479	0.58097
PPIF	0.411390059	1.88E-22	6.99E-21
PPIG	0.054853564	0.213965	0.277926
PPIH	0.219680472	4.78E-07	1.77E-06
PPIL1	0.494573198	4.00E-33	7.68E-31
PPIL2	-0.159495893	0.000279	0.000656
PPIL3	0.092013897	0.036844	0.057947
PPIL4	0.013653353	0.757241	0.804182
PPIL5	0.559414652	9.67E-44	1.38E-40
PPIL6	-0.308256351	8.47E-13	7.49E-12
PPIP5K1	-0.207671265	2.00E-06	6.69E-06
PPIP5K2	0.029279867	0.507335	0.579927
PPL	-0.311601822	4.65E-13	4.28E-12
PPM1A	0.106375253	0.015734	0.026797
PPM1B	-0.016403852	0.710352	0.76443
PPM1D	-0.057858477	0.189883	0.25047
PPM1E	0.006842949	0.876888	0.903838
PPM1F	-0.157304038	0.000339	0.000783
PPM1G	0.363015326	1.74E-17	3.12E-16
PPM1H	-0.007721196	0.861238	0.890877
PPM1J	-0.150437956	0.000614	0.001356
PPM1K	-0.128764447	0.00342	0.006609
PPM1L	-0.098924283	0.024768	0.040469

PPM1M	-0.409220339	3.27E-22	1.18E-20
PPM1N	-0.032113176	0.467115	0.540913
PPME1	0.257069105	3.23E-09	1.65E-08
PPOX	-0.239776069	3.61E-08	1.59E-07
PPP1CA	0.235646181	6.27E-08	2.65E-07
PPP1CB	0.225828789	2.23E-07	8.66E-07
PPP1CC	0.449273945	5.97E-27	4.32E-25
PPP1R10	-0.157046161	0.000347	0.0008
PPP1R11	0.177933905	4.89E-05	0.000131
PPP1R12A	0.233584372	8.21E-08	3.43E-07
PPP1R12B	-0.398174287	5.16E-21	1.57E-19
PPP1R12C	-0.325639423	3.47E-14	3.78E-13
PPP1R13B	-0.288960694	2.31E-11	1.64E-10
PPP1R13L	-0.146052685	0.000887	0.001902
PPP1R14A	-0.127642001	0.003714	0.007127
PPP1R14B	0.353353447	1.37E-16	2.12E-15
PPP1R14C	-0.270386966	4.44E-10	2.60E-09
PPP1R14D	0.148352198	0.000732	0.001597
PPP1R15A	-0.253097514	5.72E-09	2.83E-08
PPP1R15B	0.210898474	1.37E-06	4.71E-06
PPP1R16A	0.004691915	0.915409	0.933769
PPP1R16B	-0.218962729	5.22E-07	1.92E-06
PPP1R1A	-0.030487238	0.489977	0.563275
PPP1R1B	-0.318930978	1.22E-13	1.22E-12
PPP1R1C	0.012846747	0.771172	0.815981
PPP1R2P1	0.048818371	0.268799	0.338463
PPP1R2P3	0.153322884	0.00048	0.00108
PPP1R2P9	-0.027801818	0.529014	0.601033
PPP1R2	0.141307004	0.001304	0.002717
PPP1R3A	-0.036320213	0.410789	0.485447
PPP1R3B	0.081055082	0.066064	0.098295
PPP1R3C	-0.123569166	0.004982	0.009364
PPP1R3D	-0.115601267	0.008643	0.015509
PPP1R3E	-0.323557024	5.14E-14	5.44E-13
PPP1R3F	-0.100367286	0.022732	0.037436
PPP1R3G	0.15741312	0.000336	0.000776
PPP1R7	-0.124708362	0.004593	0.008684
PPP1R8	0.229825996	1.34E-07	5.40E-07
PPP1R9A	-0.219573435	4.85E-07	1.80E-06
PPP1R9B	-0.23972028	3.64E-08	1.60E-07
PPP2CA	0.284837094	4.54E-11	3.07E-10
PPP2CB	-0.106990426	0.015137	0.025882
PPP2R1A	0.107935205	0.01426	0.024525
PPP2R1B	0.316740244	1.83E-13	1.78E-12

PPP2R2A	0.078282759	0.07591	0.111393
PPP2R2B	-0.241085899	3.03E-08	1.35E-07
PPP2R2C	0.190947373	1.28E-05	3.78E-05
PPP2R2D	0.137137236	0.001813	0.003681
PPP2R3A	-0.094586621	0.031864	0.050826
PPP2R3B	-0.035585301	0.420328	0.494964
PPP2R3C	0.234766807	7.03E-08	2.96E-07
PPP2R4	0.003108696	0.943894	0.955462
PPP2R5A	-0.235977485	6.00E-08	2.55E-07
PPP2R5B	-0.195946194	7.48E-06	2.28E-05
PPP2R5C	0.04013043	0.363425	0.437995
PPP2R5D	0.133163219	0.002461	0.004881
PPP2R5E	0.306028011	1.26E-12	1.08E-11
PPP3CA	-0.081920224	0.063216	0.094485
PPP3CB	0.089505834	0.042321	0.065737
PPP3CC	-0.140884091	0.001349	0.002801
PPP3R1	0.28322023	5.90E-11	3.92E-10
PPP3R2	-0.140234609	0.00142	0.00294
PPP4C	0.23690328	5.31E-08	2.27E-07
PPP4R1L	-0.13123919	0.002845	0.005578
PPP4R1	0.026486861	0.548688	0.618985
PPP4R2	0.294080386	9.85E-12	7.38E-11
PPP4R4	-0.191781299	1.17E-05	3.48E-05
PPP5C	0.055849167	0.205754	0.268675
PPP6C	0.01452898	0.742208	0.791424
PPPDE1	0.05735774	0.193751	0.254833
PPPDE2	0.221690795	3.73E-07	1.40E-06
PPRC1	0.081531225	0.064484	0.096146
PPT1	-0.104453323	0.017733	0.02987
PPT2	0.151690108	0.000552	0.001229
PPTC7	0.202599862	3.57E-06	1.15E-05
PPWD1	-0.234895962	6.92E-08	2.92E-07
PPY2	0.120174486	0.006324	0.01165
PPYR1	-0.161950189	0.000224	0.000536
PPY	0.034781789	0.430905	0.505557
PQBP1	0.090084375	0.041	0.063909
PQLC1	-0.158861836	0.000295	0.000691
PQLC2	-0.059729521	0.17593	0.234589
PQLC3	-0.041137996	0.351494	0.42603
PRAC	0.022730867	0.606792	0.671461
PRAF2	-0.083491601	0.058301	0.087789
PRAM1	-0.372019411	2.38E-18	4.87E-17
PRAMEF10	0.065263301	0.13913	0.190802
PRAMEF11	0.05882478	0.182579	0.242017

PRAMEF12	0.048489411	0.272042	0.34205
PRAMEF13	-0.046335054	0.29394	0.365264
PRAMEF14	-0.071880366	0.103235	0.146227
PRAMEF16	-0.060653671	0.169326	0.226812
PRAMEF18	-0.058661194	0.1838	0.243443
PRAMEF1	0.050624629	0.251464	0.319554
PRAMEF20	0.034310386	0.43718	0.511554
PRAMEF22	-0.026181558	0.553307	0.62307
PRAMEF2	0.026603576	0.546927	0.617347
PRAMEF4	0.049993626	0.25743	0.326125
PRAMEF5	0.0406842	0.356837	0.431381
PRAMEF6	0.049870391	0.258606	0.32736
PRAMEF8	0.008265752	0.851561	0.882649
PRAMEF9	0.022254906	0.614346	0.678128
PRAME	0.246070817	1.53E-08	7.13E-08
PRAP1	0.21041255	1.45E-06	4.96E-06
PRB1	-0.114995199	0.009002	0.016105
PRB2	-0.136402474	0.001919	0.003879
PRB3	-0.04515544	0.306416	0.378165
PRB4	-0.109791354	0.012665	0.021984
PRC1	0.493029721	6.73E-33	1.23E-30
PRCC	0.187852544	1.78E-05	5.13E-05
PRCD	-0.373791944	1.60E-18	3.37E-17
PRCP	0.069103153	0.117287	0.163867
PRDM10	0.013354323	0.762397	0.80881
PRDM11	-0.169823392	0.000108	0.000273
PRDM12	0.159551885	0.000278	0.000653
PRDM13	0.087708864	0.046654	0.07182
PRDM14	0.019773301	0.654383	0.714042
PRDM15	-0.162059426	0.000221	0.000532
PRDM16	-0.420930432	1.56E-23	6.93E-22
PRDM1	0.000750174	0.98645	0.989867
PRDM2	-0.400434249	2.96E-21	9.38E-20
PRDM4	0.241652157	2.81E-08	1.25E-07
PRDM5	-0.184529708	2.51E-05	7.05E-05
PRDM6	-0.367032946	7.22E-18	1.38E-16
PRDM7	0.050981307	0.248136	0.315987
PRDM8	-0.071191266	0.106591	0.150461
PRDM9	0.190615165	1.33E-05	3.91E-05
PRDX1	0.23343733	8.37E-08	3.49E-07
PRDX2	0.112064809	0.010928	0.019245
PRDX3	0.381137807	2.99E-19	7.05E-18
PRDX4	0.331897658	1.04E-14	1.22E-13
PRDX5	-0.069666976	0.114319	0.160256

PRDX6	0.250719637	8.01E-09	3.88E-08
PRDXDD1P	-0.166413103	0.000148	0.000367
PREB	0.312225072	4.16E-13	3.85E-12
PRELID1	0.221849061	3.66E-07	1.38E-06
PRELID2	-0.090811052	0.039391	0.061564
PRELP	-0.28270534	6.41E-11	4.24E-10
PREPL	-0.197421923	6.37E-06	1.96E-05
PREP	0.022026192	0.617991	0.681361
PREX1	-0.256216276	3.65E-09	1.85E-08
PREX2	-0.357554451	5.62E-17	9.35E-16
PRF1	-0.066307143	0.132904	0.183194
PRG1	0.05683923	0.197818	0.259532
PRG2	-0.15196324	0.00054	0.001202
PRG3	0.001415966	0.974428	0.980807
PRG4	-0.387849433	6.21E-20	1.60E-18
PRH1	0.00957891	0.82832	0.863365
PRH2	-0.171018375	9.60E-05	0.000245
PRHOXNB	0.012350255	0.779783	0.82284
PRIC285	-0.184566734	2.50E-05	7.03E-05
PRICKLE1	-0.263227188	1.31E-09	7.12E-09
PRICKLE2	-0.333769513	7.24E-15	8.75E-14
PRICKLE3	0.073396005	0.096149	0.137314
PRICKLE4	-0.482828578	1.96E-31	2.80E-29
PRIM1	0.382713294	2.07E-19	5.01E-18
PRIM2	0.198797981	5.47E-06	1.71E-05
PRIMA1	-0.184748667	2.46E-05	6.90E-05
PRINS	-0.118907551	0.006903	0.012629
PRKAA1	0.036668403	0.406315	0.481099
PRKAA2	0.165010381	0.000169	0.000414
PRKAB1	-0.071207152	0.106512	0.150361
PRKAB2	0.137996811	0.001695	0.003462
PRKACA	-0.227387002	1.83E-07	7.21E-07
PRKACB	0.057041399	0.196225	0.257697
PRKACG	0.000725135	0.986903	0.990272
PRKAG1	0.286504389	3.46E-11	2.39E-10
PRKAG2	-0.015758425	0.721265	0.773633
PRKAG3	-0.113370181	0.010029	0.017804
PRKAR1A	-0.171640477	9.05E-05	0.000232
PRKAR1B	0.084006322	0.056761	0.085736
PRKAR2A	0.040865122	0.3547	0.429318
PRKAR2B	-0.154082601	0.00045	0.001017
PRKCA	-0.11248863	0.010628	0.01876
PRKCB	-0.295518497	7.73E-12	5.89E-11
PRKCDBP	-0.038202417	0.386951	0.461781

PRKCD	-0.305889075	1.29E-12	1.11E-11
PRKCE	-0.38372633	1.64E-19	4.02E-18
PRKCG	0.043592532	0.323477	0.396593
PRKCH	-0.217220403	6.45E-07	2.34E-06
PRKCI	0.014386254	0.744652	0.793522
PRKCQ	-0.215964339	7.51E-07	2.69E-06
PRKCSH	-0.024461059	0.579686	0.646835
PRKCZ	-0.244113227	2.01E-08	9.18E-08
PRKD1	-0.162195282	0.000219	0.000526
PRKD2	-0.217538506	6.21E-07	2.26E-06
PRKD3	0.049150621	0.265551	0.3349
PRKDC	0.284843228	4.54E-11	3.07E-10
PRKG1	-0.115048713	0.00897	0.016052
PRKG2	0.038994405	0.377177	0.451925
PRKRA	0.216526634	7.02E-07	2.52E-06
PRKRIP1	-0.057247817	0.194608	0.255792
PRKRIR	0.239549725	3.73E-08	1.63E-07
PRKX	-0.031187155	0.480063	0.553917
PRKY	0.110063919	0.012445	0.021638
PRLHR	0.057099505	0.195769	0.257216
PRLH	0.006399913	0.884801	0.909743
PRLR	-0.087983735	0.045968	0.07088
PRL	0.052987338	0.229986	0.295929
PRM1	-0.067647164	0.12523	0.173525
PRM2	-0.062495461	0.156721	0.21183
PRM3	0.106640347	0.015474	0.026391
PRMT10	-0.123355983	0.005058	0.009496
PRMT1	0.310347911	5.83E-13	5.29E-12
PRMT2	-0.37426724	1.44E-18	3.05E-17
PRMT3	0.391574674	2.56E-20	7.02E-19
PRMT5	0.42373194	7.42E-24	3.46E-22
PRMT6	0.005336391	0.903843	0.924706
PRMT7	-0.186224658	2.11E-05	6.00E-05
PRMT8	-0.301286701	2.88E-12	2.33E-11
PRND	-0.136746057	0.001869	0.003785
PRNP	-0.098001736	0.026151	0.042538
PRO0611	-0.16160061	0.000231	0.000552
PRO0628	-0.143176979	0.001122	0.002365
PRO1768	-0.050205331	0.255417	0.323919
PROCA1	-0.248519046	1.09E-08	5.19E-08
PROCR	0.039884792	0.366372	0.441121
PROC	-0.012886683	0.77048	0.815381
PRODH2	0.100929625	0.021978	0.036324
PRODH	-0.315871166	2.14E-13	2.07E-12

PROK1	-0.156650479	0.000359	0.000826
PROK2	0.017636239	0.689681	0.746041
PROKR1	-0.094621902	0.0318	0.05074
PROKR2	-0.011244936	0.79905	0.839188
PROL1	0.069507438	0.115153	0.161306
PROM1	-0.155415132	0.0004	0.000912
PROM2	-0.162372102	0.000215	0.000518
PROP1	-0.105220899	0.016909	0.028599
PROS1	-0.173069521	7.89E-05	0.000204
PROSC	-0.014931351	0.735332	0.785727
PROX1	0.006513338	0.882774	0.908053
PROX2	-0.058872825	0.182221	0.24164
PROZ	0.017253847	0.696071	0.751936
PRPF18	0.027857246	0.528192	0.600336
PRPF19	0.280989955	8.45E-11	5.51E-10
PRPF31	-0.061851005	0.161048	0.216931
PRPF38A	0.006855927	0.876656	0.903693
PRPF38B	-0.21543978	8.00E-07	2.85E-06
PRPF39	0.000545793	0.990142	0.992724
PRPF3	-0.069828276	0.113481	0.159255
PRPF40A	0.32897719	1.83E-14	2.08E-13
PRPF40B	-0.024219159	0.583441	0.6503
PRPF4B	-0.065706429	0.13646	0.187537
PRPF4	0.412524324	1.40E-22	5.33E-21
PRPF6	-0.097818843	0.026433	0.042947
PRPF8	-0.104849667	0.017303	0.029186
PRPH2	0.024861213	0.573498	0.641435
PRPH	-0.106880793	0.015242	0.026037
PRPS1L1	0.029154022	0.509162	0.581684
PRPS1	0.184141662	2.61E-05	7.32E-05
PRPS2	0.209289583	1.66E-06	5.61E-06
PRPSAP1	0.11307468	0.010226	0.018121
PRPSAP2	0.149145443	0.000685	0.001503
PRR11	0.43677283	2.11E-25	1.24E-23
PRR12	-0.208491099	1.82E-06	6.13E-06
PRR13	0.174426921	6.91E-05	0.00018
PRR14	-0.134773454	0.002176	0.004362
PRR15L	-0.29985886	3.68E-12	2.93E-11
PRR15	0.004742195	0.914506	0.933229
PRR16	0.163789385	0.000189	0.000459
PRR18	-0.089364178	0.04265	0.066202
PRR19	0.266099395	8.51E-10	4.78E-09
PRR22	-0.166961865	0.000141	0.00035
PRR23A	-0.030193228	0.494175	0.567513

PRR23C	-0.033899378	0.442695	0.516917
PRR24	-0.237817942	4.70E-08	2.03E-07
PRR25	-0.122093555	0.00553	0.010311
PRR3	-0.013052325	0.767614	0.813136
PRR4	-0.041352907	0.348981	0.423472
PRR5-ARHGAP8	-0.201782465	3.92E-06	1.25E-05
PRR5L	-0.087491783	0.047202	0.07258
PRR5	-0.093369749	0.034143	0.054094
PRR7	0.210586588	1.42E-06	4.87E-06
PRRC1	0.09520331	0.03076	0.049249
PRRG1	-0.012364149	0.779542	0.822643
PRRG2	-0.192729568	1.06E-05	3.16E-05
PRRG3	-0.149686702	0.000655	0.00144
PRRG4	-0.030084776	0.495728	0.568904
PRRT1	-0.089183804	0.043071	0.066794
PRRT2	-0.385131871	1.18E-19	2.94E-18
PRRT3	-0.333951266	6.99E-15	8.47E-14
PRRT4	-0.106309512	0.015799	0.026889
PRRX1	0.054496118	0.21697	0.281317
PRRX2	0.002077529	0.962488	0.971136
PRSS12	-0.326388085	3.01E-14	3.31E-13
PRSS16	-0.146620219	0.000846	0.001823
PRSS1	0.056672996	0.199134	0.260969
PRSS21	-0.022415517	0.611792	0.675944
PRSS22	-0.098769851	0.024995	0.0408
PRSS23	0.205990993	2.43E-06	8.00E-06
PRSS27	-0.13838523	0.001644	0.003362
PRSS30P	-0.079160541	0.072671	0.10701
PRSS33	-0.100916892	0.021995	0.036346
PRSS35	-0.160622313	0.000252	0.000598
PRSS36	-0.153237827	0.000484	0.001087
PRSS37	-0.118140547	0.007276	0.013264
PRSS38	0.018072272	0.682421	0.739388
PRSS3	0.216113502	7.38E-07	2.64E-06
PRSS41	0.029664054	0.501777	0.574682
PRSS42	0.073552549	0.09544	0.136387
PRSS45	-0.202612495	3.57E-06	1.14E-05
PRSS48	-0.020064301	0.649634	0.709585
PRSS50	-0.139007572	0.001565	0.003213
PRSS53	-0.043544951	0.324006	0.397071
PRSS54	-0.067765647	0.124569	0.172728
PRSS55	-0.014554159	0.741777	0.791049
PRSS8	-0.245893931	1.57E-08	7.29E-08
PRSSL1	-0.123057819	0.005166	0.009683

PRTFDC1	0.313150964	3.52E-13	3.30E-12
PRTG	-0.179350411	4.25E-05	0.000115
PRTN3	-0.038375949	0.384796	0.459429
PRUNE2	-0.093483917	0.033924	0.053776
PRUNE	0.09787609	0.026344	0.042817
PRX	-0.408182355	4.26E-22	1.52E-20
PRY2	-0.035833457	0.417093	0.49185
PSAPL1	-0.059122434	0.180371	0.239616
PSAP	-0.109174678	0.013177	0.022805
PSAT1	0.371854468	2.47E-18	5.04E-17
PSCA	0.010735351	0.807975	0.84647
PSD2	-0.09622517	0.029002	0.046697
PSD3	-0.069469058	0.115354	0.161554
PSD4	-0.324369552	4.41E-14	4.72E-13
PSD	-0.182447928	3.11E-05	8.59E-05
PSEN1	0.200464295	4.54E-06	1.43E-05
PSEN2	-0.080206808	0.068959	0.102153
PSENEN	0.156427502	0.000366	0.000841
PSG10	0.044220002	0.316555	0.388917
PSG11	0.055495354	0.208645	0.271971
PSG1	0.142746832	0.001161	0.002442
PSG2	0.086295505	0.050318	0.076991
PSG3	0.218729045	5.37E-07	1.97E-06
PSG4	0.084019208	0.056723	0.085698
PSG5	0.094525781	0.031975	0.050978
PSG6	0.196025958	7.42E-06	2.26E-05
PSG7	0.084386746	0.055646	0.084216
PSG8	0.129029984	0.003354	0.006494
PSG9	0.07364016	0.095045	0.135861
PSIMCT-1	0.165908057	0.000155	0.000383
PSIP1	-0.016191182	0.713942	0.767422
PSKH1	-0.14707071	0.000815	0.001761
PSKH2	0.036065651	0.414079	0.488728
PSMA1	0.335335975	5.32E-15	6.58E-14
PSMA2	0.362337961	2.01E-17	3.57E-16
PSMA3	0.434785481	3.66E-25	2.09E-23
PSMA4	0.348377917	3.85E-16	5.66E-15
PSMA5	0.37706209	7.62E-19	1.69E-17
PSMA6	0.35405283	1.18E-16	1.85E-15
PSMA7	0.319357534	1.13E-13	1.14E-12
PSMA8	-0.049460816	0.262542	0.331545
PSMB10	-0.104497382	0.017685	0.029794
PSMB11	0.009585814	0.828198	0.863313
PSMB1	0.188372554	1.68E-05	4.88E-05

PSMB2	0.238882143	4.08E-08	1.78E-07
PSMB3	0.259715672	2.20E-09	1.15E-08
PSMB4	0.260564102	1.94E-09	1.03E-08
PSMB5	0.475346286	2.17E-30	2.58E-28
PSMB6	0.274949905	2.19E-10	1.34E-09
PSMB7	0.32870118	1.93E-14	2.19E-13
PSMB8	0.070403282	0.110533	0.155422
PSMB9	0.060464983	0.170659	0.228331
PSMC1	0.253271987	5.58E-09	2.77E-08
PSMC2	0.304974572	1.51E-12	1.29E-11
PSMC3IP	0.298493851	4.65E-12	3.64E-11
PSMC3	0.178347741	4.70E-05	0.000126
PSMC4	0.310172002	6.01E-13	5.45E-12
PSMC5	0.096833376	0.027997	0.045246
PSMC6	0.530774868	9.09E-39	5.51E-36
PSMD10	0.197633519	6.22E-06	1.92E-05
PSMD11	0.386400532	8.75E-20	2.20E-18
PSMD12	0.425906206	4.14E-24	2.02E-22
PSMD13	0.264888738	1.02E-09	5.67E-09
PSMD14	0.487108011	4.83E-32	7.69E-30
PSMD1	0.328291743	2.09E-14	2.35E-13
PSMD2	0.28410517	5.12E-11	3.43E-10
PSMD3	0.241503471	2.86E-08	1.28E-07
PSMD4	0.236750169	5.41E-08	2.31E-07
PSMD5	0.15908576	0.000289	0.000678
PSMD6	0.259853535	2.15E-09	1.13E-08
PSMD7	0.389381863	4.32E-20	1.14E-18
PSMD8	0.232660289	9.27E-08	3.84E-07
PSMD9	0.33765933	3.36E-15	4.31E-14
PSME1	0.151470235	0.000563	0.001251
PSME2	0.227865168	1.72E-07	6.81E-07
PSME3	0.368894021	4.78E-18	9.38E-17
PSME4	0.248527854	1.09E-08	5.18E-08
PSMF1	-0.032797599	0.457671	0.53164
PSMG1	0.277180061	1.55E-10	9.67E-10
PSMG2	0.126734297	0.003968	0.007583
PSMG3	0.246984653	1.35E-08	6.33E-08
PSMG4	0.09056421	0.039931	0.06237
PSORS1C1	-0.129717565	0.003187	0.006194
PSORS1C2	-0.156650668	0.000359	0.000826
PSORS1C3	-0.240515068	3.27E-08	1.45E-07
PSPC1	0.150207103	0.000626	0.001381
PSPH	0.287179785	3.10E-11	2.15E-10
PSPN	-0.021705903	0.623111	0.685993

PSRC1	0.356847006	6.54E-17	1.08E-15
PSTK	0.147231352	0.000804	0.001739
PSTPIP1	-0.261850589	1.60E-09	8.61E-09
PSTPIP2	-0.122748331	0.005281	0.009878
PTAFR	-0.0768832	0.081316	0.118302
PTAR1	-0.214683769	8.76E-07	3.11E-06
PTBP1	0.2347163	7.08E-08	2.98E-07
PTBP2	0.013168181	0.765611	0.811318
PTCD1	0.19420684	9.04E-06	2.72E-05
PTCD2	0.126014628	0.004181	0.007955
PTCD3	0.23462716	7.17E-08	3.01E-07
PTCH1	-0.408454491	3.97E-22	1.42E-20
PTCH2	-0.358911318	4.21E-17	7.14E-16
PTCHD1	-0.238741419	4.15E-08	1.81E-07
PTCHD2	-0.188667607	1.63E-05	4.73E-05
PTCHD3	0.005328491	0.903984	0.924758
PTCRA	-0.263622775	1.23E-09	6.74E-09
PTDSS1	0.36733101	6.76E-18	1.30E-16
PTDSS2	0.107180107	0.014957	0.025623
PTENP1	0.036438991	0.40926	0.483955
PTEN	-0.07409909	0.092996	0.133153
PTER	0.182176929	3.20E-05	8.80E-05
PTF1A	0.010065267	0.819749	0.856247
PTGDR	-0.160347367	0.000259	0.000612
PTGDS	-0.392061531	2.28E-20	6.27E-19
PTGER1	-0.135086361	0.002125	0.004268
PTGER2	-0.06797567	0.123403	0.171444
PTGER3	-0.129535092	0.003231	0.006272
PTGER4	-0.304168046	1.74E-12	1.47E-11
PTGES2	0.196522726	7.03E-06	2.15E-05
PTGES3	0.455654872	9.14E-28	7.34E-26
PTGES	0.14693789	0.000824	0.001779
PTGFRN	0.003326361	0.939973	0.952699
PTGFR	-0.243984238	2.04E-08	9.32E-08
PTGIR	-0.321479388	7.59E-14	7.83E-13
PTGIS	-0.093661962	0.033584	0.053292
PTGR1	0.235781435	6.15E-08	2.61E-07
PTGR2	0.074481215	0.091318	0.131163
PTGS1	-0.201409338	4.09E-06	1.30E-05
PTGS2	0.056134895	0.20344	0.266054
PTH1R	-0.350766325	2.35E-16	3.53E-15
PTH2R	0.047841267	0.27851	0.349029
PTH2	0.080771432	0.067021	0.099577
PTHLH	0.237468071	4.92E-08	2.12E-07

PTH	0.009193119	0.835133	0.86882
PTK2B	-0.432153999	7.57E-25	4.11E-23
PTK2	0.08952789	0.04227	0.065668
PTK6	-0.020489918	0.642713	0.703458
PTK7	-0.32522096	3.75E-14	4.07E-13
PTMA	0.236155868	5.86E-08	2.49E-07
PTMS	0.058586191	0.184363	0.244123
PTN	-0.212960302	1.08E-06	3.75E-06
PTOV1	0.030454979	0.490437	0.563739
PTP4A1	0.252002203	6.68E-09	3.27E-08
PTP4A2	0.193789234	9.46E-06	2.84E-05
PTP4A3	-0.053992173	0.221257	0.286052
PTPDC1	0.03708061	0.401056	0.475636
PTPLAD1	0.188695366	1.63E-05	4.72E-05
PTPLAD2	-0.251127276	7.56E-09	3.69E-08
PTPLA	0.102607576	0.019858	0.033117
PTPLB	0.169325768	0.000113	0.000285
PTPMT1	0.037189243	0.399677	0.474334
PTPN11	0.256869044	3.33E-09	1.69E-08
PTPN12	0.15980173	0.000271	0.00064
PTPN13	-0.404491387	1.08E-21	3.66E-20
PTPN14	0.000719725	0.987	0.99032
PTPN18	-0.411683255	1.74E-22	6.53E-21
PTPN1	-0.112147406	0.010869	0.019154
PTPN20A	0.02062954	0.640449	0.701518
PTPN20B	0.036124796	0.413313	0.488026
PTPN21	-0.300030369	3.57E-12	2.85E-11
PTPN22	-0.173226384	7.77E-05	0.000201
PTPN23	-0.169388421	0.000112	0.000283
PTPN2	0.0760401	0.084721	0.122692
PTPN3	-0.260004083	2.11E-09	1.11E-08
PTPN4	-0.097048808	0.027649	0.044737
PTPN5	-0.114488394	0.009312	0.016624
PTPN6	-0.262129847	1.54E-09	8.31E-09
PTPN7	-0.124359195	0.004709	0.008892
PTPN9	-0.001151231	0.979208	0.984676
PTPRA	-0.187477519	1.85E-05	5.32E-05
PTPRB	-0.223663213	2.92E-07	1.12E-06
PTPRCAP	-0.151575982	0.000558	0.00124
PTPRC	-0.193935085	9.31E-06	2.80E-05
PTPRD	-0.244736487	1.84E-08	8.47E-08
PTPRE	-0.255394661	4.11E-09	2.07E-08
PTPRF	-0.121505815	0.005763	0.0107
PTPRG	-0.136904294	0.001846	0.003743

PTPRH	0.176451629	5.67E-05	0.00015
PTPRJ	-0.258885281	2.48E-09	1.29E-08
PTPRK	-0.110146943	0.012378	0.021528
PTPRM	-0.19311215	1.02E-05	3.04E-05
PTPRN2	-0.124914188	0.004526	0.00856
PTPRN	0.263989495	1.17E-09	6.42E-09
PTPRO	-0.201014172	4.27E-06	1.36E-05
PTPRQ	-0.223555958	2.96E-07	1.13E-06
PTPRR	0.00839981	0.849182	0.880823
PTPRS	-0.259950642	2.12E-09	1.12E-08
PTPRT	-0.281851017	7.36E-11	4.83E-10
PTPRU	-0.297348268	5.66E-12	4.39E-11
PTPRVP	-0.177530514	5.09E-05	0.000136
PTPRZ1	-0.205672201	2.52E-06	8.28E-06
PTRF	-0.176651797	5.56E-05	0.000147
PTRH1	-0.109836355	0.012629	0.021925
PTRH2	0.327979553	2.22E-14	2.48E-13
PTS	0.330330147	1.41E-14	1.63E-13
PTTG1IP	-0.080824882	0.06684	0.099344
PTTG1	0.349742078	2.90E-16	4.33E-15
PTTG2	0.158526305	0.000304	0.00071
PTTG3P	0.261922578	1.59E-09	8.53E-09
PTX3	-0.002184795	0.960552	0.96982
PTX4	-0.124546258	0.004647	0.008781
PUF60	0.332176439	9.88E-15	1.16E-13
PUM1	-0.073261642	0.096761	0.138107
PUM2	-0.112937145	0.01032	0.018271
PURA	-0.153051986	0.000491	0.001103
PURB	0.038491319	0.383368	0.458025
PURG	-0.116519346	0.008125	0.014675
PUS10	-0.311047645	5.14E-13	4.70E-12
PUS1	0.301923135	2.58E-12	2.10E-11
PUS3	0.230773745	1.18E-07	4.82E-07
PUS7L	0.368231174	5.54E-18	1.08E-16
PUS7	0.376542737	8.58E-19	1.88E-17
PUSL1	0.120890233	0.006017	0.011133
PVALB	-0.237350037	5.00E-08	2.15E-07
PVRIG	-0.260335131	2.01E-09	1.06E-08
PVRL1	-0.009774901	0.824863	0.86051
PVRL2	0.132152409	0.002657	0.005241
PVRL3	-0.23097311	1.15E-07	4.70E-07
PVRL4	-0.189949008	1.43E-05	4.18E-05
PVR	0.30347557	1.97E-12	1.64E-11
PVT1	0.114802792	0.009118	0.016301

PWP1	0.465542014	4.61E-29	4.39E-27
PWP2	-0.05208022	0.238073	0.304765
PWRN1	-0.119369207	0.006687	0.012273
PWRN2	-0.018219435	0.679977	0.737197
PWWP2A	-0.230956851	1.16E-07	4.71E-07
PWWP2B	-0.063297133	0.151461	0.205611
PXDNL	0.058834854	0.182504	0.241934
PXDN	0.021707238	0.623089	0.685993
PXK	-0.138748928	0.001598	0.003274
PXMP2	0.201586717	4.01E-06	1.28E-05
PXMP4	-0.270938458	4.08E-10	2.40E-09
PXN	0.035628582	0.419763	0.494377
PXT1	-0.221379079	3.88E-07	1.45E-06
PYCARD	-0.19832319	5.76E-06	1.79E-05
PYCR1	0.376892259	7.92E-19	1.75E-17
PYCR2	-0.193168591	1.01E-05	3.03E-05
PYCRL	0.249741278	9.19E-09	4.42E-08
PYDC1	-0.031921322	0.469782	0.543529
PYGB	-0.01525639	0.729793	0.781066
PYGL	0.369399165	4.28E-18	8.47E-17
PYGM	-0.363262696	1.65E-17	2.96E-16
PYGO1	0.036523707	0.408171	0.482838
PYGO2	0.016579838	0.707387	0.761936
PYHIN1	-0.151622982	0.000555	0.001235
PYROXD1	0.13602888	0.001976	0.003988
PYROXD2	-0.339081392	2.54E-15	3.31E-14
PYY2	0.054046074	0.220796	0.285574
PYY	0.099141325	0.024452	0.039989
PZP	-0.208295533	1.86E-06	6.26E-06
ProSAPiP1	-0.222590656	3.34E-07	1.27E-06
QARS	-0.02755814	0.532632	0.604146
QDPR	0.033893379	0.442776	0.516981
QKI	-0.061890806	0.160779	0.216626
QPCTL	-0.000224407	0.995947	0.997294
QPCT	0.175319707	6.33E-05	0.000166
QPRT	0.10185599	0.020784	0.034525
QRFPR	-0.009461079	0.8304	0.865111
QRFP	-0.016518442	0.708421	0.762762
QRICH1	-0.072766445	0.099044	0.141083
QRICH2	-0.20082038	4.37E-06	1.38E-05
QRSL1	0.121650095	0.005705	0.010603
QSER1	0.153714068	0.000464	0.001047
QSOX1	-0.074725508	0.090258	0.129799
QSOX2	0.160785073	0.000249	0.00059

QTRT1	-0.16046155	0.000256	0.000606
QTRTD1	0.126757567	0.003961	0.007573
R3HCC1	-0.050577864	0.251903	0.320051
R3HDM1	0.400141634	3.18E-21	1.00E-19
R3HDM2	-0.088220672	0.045383	0.070048
R3HDML	0.063968432	0.147161	0.200426
RAB10	0.480451661	4.23E-31	5.60E-29
RAB11A	0.176361049	5.72E-05	0.000151
RAB11B	-0.246806235	1.38E-08	6.48E-08
RAB11FIP1	-0.343273937	1.09E-15	1.50E-14
RAB11FIP2	-0.210919173	1.37E-06	4.70E-06
RAB11FIP3	-0.342290915	1.33E-15	1.81E-14
RAB11FIP4	-0.346901393	5.21E-16	7.55E-15
RAB11FIP5	-0.076830273	0.081527	0.118571
RAB12	-0.07648925	0.082893	0.120342
RAB13	0.054743009	0.214891	0.279039
RAB14	-0.055054358	0.21229	0.276002
RAB15	0.031043993	0.482082	0.555893
RAB17	-0.315252711	2.40E-13	2.30E-12
RAB18	0.242366908	2.55E-08	1.15E-07
RAB19	-0.055714032	0.206855	0.269901
RAB1A	0.270304537	4.50E-10	2.63E-09
RAB1B	0.07753303	0.078769	0.115048
RAB20	-0.217138409	6.52E-07	2.36E-06
RAB21	0.103906381	0.018341	0.030803
RAB22A	0.117246945	0.007734	0.014022
RAB23	0.207727287	1.99E-06	6.65E-06
RAB24	-0.233739225	8.05E-08	3.36E-07
RAB25	-0.115935289	0.008451	0.015199
RAB26	0.027844823	0.528376	0.600511
RAB27A	-0.209685447	1.58E-06	5.37E-06
RAB27B	0.03548907	0.421587	0.496241
RAB28	-0.002174508	0.960738	0.969909
RAB2A	0.326973475	2.69E-14	2.98E-13
RAB2B	-0.02458638	0.577745	0.645149
RAB30	-0.087289232	0.047718	0.073255
RAB31	0.067320248	0.12707	0.175879
RAB32	0.049650359	0.260715	0.32953
RAB33A	-0.070711531	0.108977	0.153516
RAB33B	-0.041045835	0.352575	0.427185
RAB34	0.065590214	0.137157	0.188405
RAB35	0.232780827	9.12E-08	3.78E-07
RAB36	-0.288471524	2.51E-11	1.76E-10
RAB37	-0.342070219	1.39E-15	1.89E-14

RAB38	-0.122726704	0.005289	0.009891
RAB39B	-0.042280699	0.338265	0.412321
RAB39	0.006346399	0.885758	0.910388
RAB3A	-0.105208309	0.016923	0.028617
RAB3B	0.289380634	2.16E-11	1.53E-10
RAB3C	-0.03304463	0.454289	0.528325
RAB3D	0.063476604	0.150302	0.20419
RAB3GAP1	-0.093991432	0.032962	0.052381
RAB3GAP2	0.120784898	0.006061	0.011202
RAB3IL1	-0.089132871	0.043191	0.066949
RAB3IP	0.057734128	0.190838	0.251565
RAB40AL	-0.246366515	1.47E-08	6.86E-08
RAB40A	-0.229326088	1.43E-07	5.73E-07
RAB40B	-0.225232377	2.40E-07	9.30E-07
RAB40C	-0.197263416	6.48E-06	2.00E-05
RAB41	-0.146794292	0.000834	0.001798
RAB42	0.003232668	0.941661	0.953778
RAB43	-0.253057034	5.75E-09	2.85E-08
RAB4A	-0.026004653	0.555992	0.625425
RAB4B	-0.265208478	9.73E-10	5.43E-09
RAB5A	0.077152812	0.080251	0.116982
RAB5B	-0.093283852	0.034309	0.054323
RAB5C	0.072993361	0.097992	0.139715
RAB6A	0.322654632	6.09E-14	6.36E-13
RAB6B	0.001143687	0.979344	0.98471
RAB6C	0.137098033	0.001818	0.003691
RAB7A	0.115485535	0.008711	0.015623
RAB7L1	-0.169690948	0.000109	0.000276
RAB8A	-0.03283439	0.457166	0.531146
RAB8B	-0.047478066	0.282179	0.352843
RAB9A	0.041405068	0.348373	0.422812
RAB9BP1	0.022560508	0.609491	0.673848
RAB9B	-0.203032808	3.40E-06	1.09E-05
RABAC1	0.025091003	0.569959	0.63812
RABEP1	-0.112168116	0.010854	0.01913
RABEP2	-0.297485009	5.53E-12	4.30E-11
RABEPK	0.335948679	4.72E-15	5.88E-14
RABGAP1L	-0.169648363	0.000109	0.000277
RABGAP1	-0.196332555	7.18E-06	2.19E-05
RABGEF1	0.147004787	0.000819	0.00177
RABGGTA	-0.028374911	0.520553	0.59263
RABGGTB	0.182550146	3.08E-05	8.51E-05
RABIF	0.200292307	4.63E-06	1.46E-05
RABL2A	-0.417225832	4.14E-23	1.73E-21

RABL2B	-0.345433756	7.04E-16	9.94E-15
RABL3	0.116463148	0.008156	0.014729
RABL5	0.06145132	0.163777	0.220206
RAC1	0.182906483	2.97E-05	8.23E-05
RAC2	-0.140849864	0.001352	0.002808
RAC3	0.194242851	9.01E-06	2.71E-05
RACGAP1P	0.383746703	1.63E-19	4.01E-18
RACGAP1	0.528668833	2.02E-38	1.16E-35
RAD17	-0.113895324	0.009686	0.017251
RAD18	0.333088648	8.27E-15	9.85E-14
RAD1	0.014501854	0.742672	0.791793
RAD21L1	0.196503966	7.04E-06	2.16E-05
RAD21	0.360101933	3.26E-17	5.65E-16
RAD23A	0.097535771	0.026874	0.043615
RAD23B	0.344833276	7.96E-16	1.11E-14
RAD50	0.043219959	0.327634	0.400952
RAD51AP1	0.499272844	8.07E-34	1.81E-31
RAD51AP2	0.003384461	0.938926	0.951928
RAD51C	0.118402182	0.007147	0.013041
RAD51L1	-0.029522827	0.503816	0.576433
RAD51L3	0.04318755	0.327997	0.401322
RAD51	0.515953471	2.25E-36	8.47E-34
RAD52	0.024037273	0.586272	0.652836
RAD54B	0.46762355	2.43E-29	2.40E-27
RAD54L2	-0.092529373	0.035797	0.056499
RAD54L	0.394902245	1.15E-20	3.28E-19
RAD9A	-0.087536693	0.047088	0.072421
RAD9B	-0.057796876	0.190356	0.250995
RADIL	-0.244288546	1.96E-08	8.98E-08
RAE1	0.304378471	1.68E-12	1.42E-11
RAET1E	0.187888187	1.77E-05	5.11E-05
RAET1G	0.114655716	0.009208	0.016457
RAET1K	-0.045500235	0.302733	0.374445
RAET1L	0.059144005	0.180212	0.239468
RAF1	-0.076829299	0.081531	0.118571
RAG1AP1	0.172628046	8.23E-05	0.000212
RAG1	0.051991196	0.238877	0.305677
RAG2	0.003743309	0.932466	0.947155
RAGE	-0.101703589	0.020977	0.034824
RAI14	-0.127288292	0.003811	0.007298
RAI1	-0.230191526	1.28E-07	5.17E-07
RAI2	-0.433708789	4.93E-25	2.78E-23
RALA	0.353503256	1.32E-16	2.07E-15
RALBP1	0.159201221	0.000286	0.000672

RALB	0.179430801	4.22E-05	0.000114
RALGAPA1	-0.046525181	0.291961	0.363225
RALGAPA2	-0.234389682	7.39E-08	3.10E-07
RALGAPB	0.036718633	0.405672	0.480423
RALGDS	-0.36468722	1.21E-17	2.22E-16
RALGPS1	-0.469051765	1.56E-29	1.61E-27
RALGPS2	0.185745876	2.22E-05	6.28E-05
RALYL	0.046728794	0.289852	0.361014
RALY	-0.05684656	0.19776	0.259508
RAMP1	0.053878118	0.222236	0.287099
RAMP2	-0.237438463	4.94E-08	2.13E-07
RAMP3	-0.307059237	1.05E-12	9.15E-12
RANBP10	-0.229000949	1.49E-07	5.95E-07
RANBP17	-0.244297542	1.96E-08	8.97E-08
RANBP1	0.418855397	2.70E-23	1.17E-21
RANBP2	-0.076731852	0.081919	0.119075
RANBP3L	-0.246238878	1.50E-08	6.97E-08
RANBP3	-0.266188893	8.40E-10	4.72E-09
RANBP6	-0.022072947	0.617245	0.680802
RANBP9	-0.03489007	0.42947	0.504126
RANGAP1	0.173301953	7.71E-05	0.000199
RANGRF	0.050790582	0.249912	0.317905
RAN	0.569847355	1.12E-45	2.25E-42
RAP1A	-0.070337024	0.110869	0.155863
RAP1B	0.143817892	0.001065	0.002253
RAP1GAP2	0.120955461	0.00599	0.011085
RAP1GAP	-0.339363392	2.40E-15	3.14E-14
RAP1GDS1	0.194457207	8.80E-06	2.66E-05
RAP2A	0.199619276	4.99E-06	1.56E-05
RAP2B	-0.022425205	0.611638	0.675849
RAP2C	0.184527489	2.51E-05	7.05E-05
RAPGEF1	-0.190589418	1.33E-05	3.92E-05
RAPGEF2	-0.258264412	2.72E-09	1.40E-08
RAPGEF3	-0.370662148	3.23E-18	6.47E-17
RAPGEF4	-0.21960144	4.83E-07	1.79E-06
RAPGEF5	-0.354604167	1.05E-16	1.66E-15
RAPGEF6	-0.093573347	0.033752	0.053539
RAPGEFL1	-0.074049259	0.093217	0.133459
RAPH1	-0.168316231	0.000124	0.000311
RAPSN	-0.182610704	3.06E-05	8.46E-05
RARA	-0.302661958	2.27E-12	1.87E-11
RARB	-0.010610806	0.81016	0.848226
RARG	-0.051836983	0.240275	0.307289
RARRES1	0.050011866	0.257256	0.326002

RARRES2	-0.08182648	0.063519	0.094876
RARRES3	-0.224999003	2.47E-07	9.55E-07
RARS2	0.070377878	0.110662	0.155593
RARS	0.333107635	8.24E-15	9.83E-14
RASA1	-0.096009517	0.029366	0.047244
RASA2	-0.095584894	0.030093	0.048321
RASA3	-0.091499779	0.037915	0.059504
RASA4P	-0.223029659	3.16E-07	1.20E-06
RASA4	-0.157659461	0.000329	0.000761
RASAL1	0.052206961	0.236931	0.303576
RASAL2	0.076826988	0.08154	0.118575
RASAL3	-0.289180378	2.23E-11	1.58E-10
RASD1	-0.01846558	0.675897	0.733513
RASD2	-0.020374533	0.644586	0.705181
RASEF	-0.100567426	0.022461	0.037033
RASGEF1A	-0.016297205	0.712151	0.765871
RASGEF1B	-0.252144826	6.55E-09	3.21E-08
RASGEF1C	0.127951092	0.003631	0.006978
RASGRF1	-0.425922629	4.12E-24	2.01E-22
RASGRF2	-0.014633788	0.740415	0.789849
RASGRP1	-0.204450741	2.90E-06	9.43E-06
RASGRP2	-0.430582069	1.16E-24	6.06E-23
RASGRP3	-0.119590271	0.006586	0.012095
RASGRP4	-0.36921374	4.46E-18	8.78E-17
RASIP1	-0.158269345	0.000311	0.000725
RASL10A	-0.236017252	5.97E-08	2.54E-07
RASL10B	0.020582417	0.641213	0.702168
RASL11A	-0.284582554	4.74E-11	3.20E-10
RASL11B	-0.045026047	0.307805	0.379708
RASL12	-0.273754108	2.64E-10	1.60E-09
RASSF10	-0.150279234	0.000623	0.001374
RASSF1	-0.12259085	0.00534	0.009982
RASSF2	-0.351959504	1.83E-16	2.80E-15
RASSF3	-0.030154173	0.494734	0.567959
RASSF4	-0.2250907	2.44E-07	9.45E-07
RASSF5	-0.427203976	2.92E-24	1.47E-22
RASSF6	0.033601455	0.446717	0.521035
RASSF7	-0.270093586	4.64E-10	2.71E-09
RASSF8	-0.105551341	0.016565	0.028072
RASSF9	0.064775974	0.142112	0.194424
RAVER1	-0.052810664	0.231545	0.297676
RAVER2	-0.205111195	2.69E-06	8.78E-06
RAX2	-0.022240751	0.614571	0.678329
RAX	0.05806841	0.188278	0.248735

RB1CC1	0.164199738	0.000182	0.000444
RB1	0.106602888	0.015511	0.026442
RBAK	-0.112886117	0.010354	0.01832
RBBP4	0.11588455	0.00848	0.015247
RBBP5	0.167603854	0.000133	0.000331
RBBP6	-0.129394184	0.003265	0.006331
RBBP7	0.213642465	9.92E-07	3.49E-06
RBBP8	0.155907589	0.000383	0.000877
RBBP9	-0.052362435	0.235536	0.302108
RBCK1	-0.009701084	0.826165	0.861553
RBKS	-0.105461758	0.016658	0.028205
RBL1	0.243567791	2.16E-08	9.83E-08
RBL2	-0.342351197	1.32E-15	1.79E-14
RBM10	-0.048297421	0.273947	0.344229
RBM11	-0.198340573	5.75E-06	1.79E-05
RBM12B	-0.020764203	0.638269	0.699513
RBM12	0.240053162	3.48E-08	1.54E-07
RBM14	0.010963664	0.803973	0.843196
RBM15B	0.015239986	0.730072	0.781276
RBM15	0.091004902	0.038971	0.060974
RBM16	-0.094753911	0.031561	0.050399
RBM17	0.222529248	3.36E-07	1.28E-06
RBM18	0.135102895	0.002122	0.004264
RBM19	0.023369137	0.596727	0.662484
RBM20	-0.138107647	0.00168	0.003433
RBM22	-0.077184799	0.080126	0.116842
RBM23	0.107231056	0.014909	0.025549
RBM24	-0.011218249	0.799517	0.839546
RBM25	-0.091876021	0.037129	0.058367
RBM26	0.030957129	0.483309	0.557051
RBM27	0.192559019	1.08E-05	3.22E-05
RBM28	0.314382869	2.81E-13	2.67E-12
RBM33	-0.311405468	4.82E-13	4.41E-12
RBM34	0.269411728	5.15E-10	2.99E-09
RBM38	0.016616207	0.706775	0.761405
RBM39	-0.20901275	1.71E-06	5.79E-06
RBM3	0.121135267	0.005915	0.010957
RBM41	0.019814646	0.653708	0.713421
RBM42	0.057677751	0.191272	0.252071
RBM43	-0.307142846	1.03E-12	9.02E-12
RBM44	-0.162309919	0.000216	0.00052
RBM45	0.210043292	1.52E-06	5.17E-06
RBM46	0.018863339	0.669325	0.727367
RBM47	-0.028557207	0.517876	0.590087

RBM4B	-0.059912779	0.174605	0.233025
RBM4	0.182745292	3.02E-05	8.36E-05
RBM5	-0.373266414	1.80E-18	3.73E-17
RBM6	-0.304951221	1.52E-12	1.29E-11
RBM7	0.218779186	5.34E-07	1.96E-06
RBM8A	0.183058942	2.92E-05	8.11E-05
RBM9	-0.075815001	0.085649	0.123902
RBMS1	-0.094603749	0.031833	0.050785
RBMS2	-0.254724664	4.53E-09	2.27E-08
RBMS3	-0.278125438	1.33E-10	8.42E-10
RBMX2	0.132882184	0.002514	0.004979
RBMXL1	0.097044079	0.027656	0.044746
RBMXL2	-0.233539465	8.26E-08	3.44E-07
RBMXL3	-0.023894494	0.588499	0.654842
RBMX	0.092235601	0.036391	0.057328
RBMY1A1	0.072460513	0.100475	0.142888
RBMY1A3P	0.022914934	0.603881	0.668867
RBMY1B	0.131214083	0.002851	0.005587
RBMY1E	-0.014041803	0.75056	0.798684
RBMY1F	0.028948341	0.512157	0.584436
RBMY1J	0.125863111	0.004227	0.008034
RBMY2EP	0.163877087	0.000188	0.000456
RBMY2FP	0.198022553	5.96E-06	1.85E-05
RBP1	0.010976567	0.803747	0.843013
RBP2	-0.169378014	0.000112	0.000283
RBP3	0.04658772	0.291312	0.362539
RBP4	-0.07802882	0.076869	0.11261
RBP5	-0.328743039	1.92E-14	2.17E-13
RBP7	-0.006392611	0.884932	0.90983
RBPJL	0.006513425	0.882772	0.908053
RBPJ	0.064898809	0.141356	0.193509
RBPMS2	0.12304442	0.005171	0.009691
RBPMS	-0.39497682	1.13E-20	3.23E-19
RBX1	0.100008887	0.023223	0.038184
RC3H1	-0.232765119	9.14E-08	3.79E-07
RC3H2	0.086878435	0.048779	0.074786
RCAN1	-0.235178604	6.66E-08	2.81E-07
RCAN2	-0.285717407	3.94E-11	2.69E-10
RCAN3	0.077830166	0.077625	0.113544
RCBTB1	-0.06657805	0.131324	0.181153
RCBTB2	-0.320175533	9.68E-14	9.81E-13
RCC1	0.22294335	3.19E-07	1.22E-06
RCC2	0.124989549	0.004501	0.008522
RCCD1	0.199645212	4.98E-06	1.56E-05

RCE1	0.161502217	0.000233	0.000556
RCHY1	0.072102038	0.102173	0.144922
RCL1	0.13123178	0.002847	0.00558
RCN1	0.179772109	4.07E-05	0.00011
RCN2	0.201933003	3.85E-06	1.23E-05
RCN3	0.015271284	0.729539	0.780915
RCOR1	0.043894501	0.320134	0.39288
RCOR2	0.301064364	2.99E-12	2.41E-11
RCOR3	-0.243235484	2.26E-08	1.03E-07
RCSD1	-0.248405045	1.11E-08	5.26E-08
RCVRN	-0.176602832	5.58E-05	0.000148
RD3	-0.1789296	4.43E-05	0.000119
RDBP	0.280028357	9.85E-11	6.35E-10
RDH10	0.188109161	1.73E-05	5.00E-05
RDH11	0.32966487	1.61E-14	1.84E-13
RDH12	0.112868021	0.010367	0.01834
RDH13	-0.145542262	0.000925	0.001977
RDH14	0.133397138	0.002418	0.004804
RDH16	0.01811601	0.681694	0.738761
RDH5	-0.047610302	0.280839	0.351497
RDH8	0.136831825	0.001856	0.003763
RDM1	0.210699309	1.41E-06	4.82E-06
RDX	0.270363377	4.46E-10	2.61E-09
REC8	-0.166148221	0.000152	0.000375
RECK	-0.166361252	0.000149	0.000369
RECQL4	0.30017705	3.49E-12	2.78E-11
RECQL5	-0.263917921	1.18E-09	6.48E-09
RECQL	0.260345567	2.00E-09	1.06E-08
REEP1	-0.261853401	1.60E-09	8.61E-09
REEP2	0.075454311	0.087153	0.125852
REEP3	0.251724767	6.95E-09	3.40E-08
REEP4	0.047971109	0.277206	0.34769
REEP5	-0.151617497	0.000556	0.001236
REEP6	-0.039817564	0.367181	0.441802
REG1A	-0.013470132	0.760399	0.80699
REG1B	0.092163271	0.036538	0.057516
REG1P	-0.052129576	0.237628	0.304293
REG3A	-0.019221814	0.663423	0.722092
REG3G	0.014738913	0.738618	0.788564
REG4	0.021917298	0.61973	0.682939
RELA	0.106034	0.016074	0.027301
RELB	0.051727736	0.241269	0.308422
RELL1	-0.116112922	0.008351	0.015043
RELL2	-0.129545365	0.003228	0.006268

RELN	-0.007103657	0.872237	0.900391
RELT	-0.061510793	0.163369	0.219701
REL	-0.245516812	1.65E-08	7.65E-08
REM1	-0.282056025	7.12E-11	4.68E-10
REM2	-0.054254028	0.219022	0.283537
RENBP	-0.280745852	8.78E-11	5.70E-10
REN	-0.248248264	1.13E-08	5.37E-08
REP15	0.084221067	0.056129	0.084903
REPIN1	0.011993837	0.785982	0.828073
REPS1	0.151651051	0.000554	0.001233
REPS2	-0.382799415	2.03E-19	4.91E-18
RER1	-0.027718846	0.530245	0.601881
RERE	-0.33798464	3.15E-15	4.06E-14
RERGL	-0.110923935	0.011771	0.020562
RERG	-0.285573708	4.03E-11	2.75E-10
RESP18	0.015351917	0.728167	0.779697
REST	0.029068507	0.510406	0.582705
RETNLB	0.177969885	4.88E-05	0.00013
RETN	-0.217888645	5.95E-07	2.17E-06
RETSAT	-0.055612981	0.207681	0.270873
RET	0.037647486	0.39389	0.468775
REV1	-0.43851789	1.29E-25	7.97E-24
REV3L	-0.326519368	2.93E-14	3.23E-13
REXO1L1	0.001645769	0.97028	0.977665
REXO1	-0.105532711	0.016584	0.0281
REXO2	0.079284861	0.072222	0.106481
REXO4	0.137815876	0.001719	0.003506
RFC1	0.023316571	0.597553	0.663107
RFC2	0.351341478	2.08E-16	3.16E-15
RFC3	0.43962989	9.45E-26	5.96E-24
RFC4	0.369319058	4.35E-18	8.61E-17
RFC5	0.403331819	1.44E-21	4.83E-20
RFESD	-0.072706334	0.099324	0.141432
RFFL	0.217569997	6.19E-07	2.25E-06
RFK	0.308492351	8.12E-13	7.22E-12
RFNG	-0.03879546	0.379618	0.454332
RFPL1S	-0.053956175	0.221566	0.286348
RFPL1	0.053499264	0.225509	0.290823
RFPL2	-0.166242742	0.000151	0.000373
RFPL3S	-0.254971586	4.37E-09	2.20E-08
RFPL3	-0.036911703	0.403206	0.477866
RFPL4A	-0.071802833	0.103608	0.146686
RFPL4B	0.049272037	0.26437	0.333643
RFT1	0.183796018	2.71E-05	7.56E-05

RFTN1	-0.300068673	3.55E-12	2.83E-11
RFTN2	-0.113604026	0.009875	0.01756
RFWD2	0.191396513	1.22E-05	3.62E-05
RFWD3	0.303729461	1.88E-12	1.57E-11
RFX1	-0.277905669	1.38E-10	8.70E-10
RFX2	-0.399023788	4.19E-21	1.30E-19
RFX3	0.012209248	0.782234	0.824864
RFX4	-0.05521683	0.210942	0.274535
RFX5	-0.193354174	9.92E-06	2.97E-05
RFX6	0.145848818	0.000902	0.001931
RFX7	-0.03387339	0.443045	0.517265
RFX8	0.206603602	2.26E-06	7.49E-06
RFXANK	-0.025242945	0.567625	0.636183
RFXAP	-0.249851233	9.05E-09	4.36E-08
RG9MTD1	0.343793653	9.83E-16	1.36E-14
RG9MTD2	0.312278812	4.12E-13	3.82E-12
RG9MTD3	-0.302504333	2.33E-12	1.91E-11
RGAG1	-0.025212582	0.568091	0.636598
RGAG4	-0.378969717	4.92E-19	1.12E-17
RGL1	-0.348975781	3.40E-16	5.04E-15
RGL2	-0.243651387	2.14E-08	9.73E-08
RGL3	-0.307472679	9.73E-13	8.54E-12
RGL4	-0.220218711	4.48E-07	1.67E-06
RGMA	-0.404352232	1.12E-21	3.78E-20
RGMB	-0.278459549	1.26E-10	8.02E-10
RGNEF	-0.253233673	5.61E-09	2.78E-08
RGN	-0.287002095	3.19E-11	2.21E-10
RGP1	0.108063644	0.014144	0.024342
RGPD1	-0.151624965	0.000555	0.001235
RGPD3	-0.195414732	7.93E-06	2.41E-05
RGPD4	-0.244690555	1.85E-08	8.52E-08
RGPD5	-0.200318475	4.62E-06	1.46E-05
RGPD6	-0.282304973	6.84E-11	4.51E-10
RGPD8	-0.012912967	0.770025	0.815043
RGR	-0.032991097	0.455021	0.529053
RGS10	0.029635195	0.502193	0.575004
RGS11	-0.348837864	3.50E-16	5.18E-15
RGS12	-0.280756129	8.77E-11	5.70E-10
RGS13	-0.288543366	2.48E-11	1.74E-10
RGS14	-0.116552999	0.008106	0.014647
RGS16	-0.159041462	0.000291	0.000681
RGS17	0.113505495	0.00994	0.017664
RGS18	-0.159784936	0.000272	0.00064
RGS19	-0.082980767	0.059863	0.089945

RGS1	-0.116257559	0.00827	0.014913
RGS20	0.26492347	1.02E-09	5.64E-09
RGS21	-0.051133712	0.246723	0.314408
RGS22	-0.269950125	4.75E-10	2.77E-09
RGS2	0.002322665	0.958065	0.967864
RGS3	-0.297631962	5.39E-12	4.19E-11
RGS4	0.147064993	0.000815	0.001762
RGS5	-0.172352315	8.45E-05	0.000217
RGS6	-0.24127531	2.95E-08	1.31E-07
RGS7BP	-0.243693858	2.12E-08	9.68E-08
RGS7	-0.040182345	0.362804	0.437378
RGS8	-0.021117127	0.63257	0.694181
RGS9BP	-0.03653527	0.408022	0.48272
RGS9	-0.33194261	1.03E-14	1.21E-13
RGSL1	-0.001175686	0.978766	0.984331
RHAG	0.028755039	0.514979	0.587322
RHBDD1	0.011105037	0.801498	0.840875
RHBDD2	-0.122035793	0.005553	0.010347
RHBDD3	0.04031027	0.361277	0.43596
RHBDF1	-0.233444249	8.37E-08	3.49E-07
RHBDF2	-0.040278291	0.361659	0.436287
RHBDL1	-0.111023824	0.011695	0.020447
RHBDL2	-0.042907284	0.331149	0.404708
RHBDL3	-0.304699992	1.59E-12	1.35E-11
RHBG	0.116087645	0.008365	0.015067
RHCE	-0.12999224	0.003123	0.00608
RHCG	0.176774229	5.49E-05	0.000145
RHD	-0.225980562	2.18E-07	8.51E-07
RHEBL1	0.098917337	0.024778	0.040479
RHEB	0.236964153	5.26E-08	2.25E-07
RHOA	0.055480751	0.208765	0.272083
RHOBTB1	0.032239926	0.465358	0.539191
RHOBTB2	-0.321888499	7.03E-14	7.30E-13
RHOBTB3	0.123060848	0.005165	0.009681
RHOB	-0.057864367	0.189838	0.250427
RHOC	0.123072943	0.005161	0.009675
RHOD	0.103442519	0.01887	0.0316
RHOF	0.214145095	9.35E-07	3.30E-06
RHOG	-0.085621967	0.052147	0.079439
RHOH	-0.010507617	0.811972	0.849811
RHOJ	-0.220912203	4.11E-07	1.54E-06
RHOQ	0.064873731	0.14151	0.193707
RHOT1	-0.00448364	0.919151	0.936582
RHOT2	-0.255585663	4.00E-09	2.02E-08

RHOU	-0.072679481	0.099449	0.14159
RHOV	0.280750914	8.77E-11	5.70E-10
RHOXF1	-0.179752819	4.08E-05	0.000111
RHOXF2B	-0.038935764	0.377896	0.452732
RHO	-0.052797971	0.231658	0.297779
RHPN1	-0.183444445	2.81E-05	7.81E-05
RHPN2	0.230639279	1.20E-07	4.90E-07
RIBC1	-0.311603817	4.65E-13	4.28E-12
RIBC2	0.037377927	0.397288	0.472088
RIC3	-0.433443814	5.30E-25	2.99E-23
RIC8A	0.035627227	0.419781	0.494377
RIC8B	0.073373906	0.096249	0.137446
RICH2	-0.378787247	5.14E-19	1.17E-17
RICTOR	0.011247592	0.799004	0.839186
RIF1	0.182065942	3.23E-05	8.90E-05
RILPL1	-0.064579557	0.143328	0.195926
RILPL2	-0.440494305	7.40E-26	4.70E-24
RILP	-0.214254431	9.22E-07	3.26E-06
RIMBP2	-0.088414419	0.04491	0.069403
RIMBP3C	-0.187685937	1.81E-05	5.22E-05
RIMBP3	-0.213511536	1.01E-06	3.54E-06
RIMKLA	-0.175870798	6.00E-05	0.000158
RIMKLB	0.024529396	0.578627	0.645906
RIMS1	-0.163278481	0.000198	0.00048
RIMS2	0.183831567	2.70E-05	7.54E-05
RIMS3	-0.267872423	6.51E-10	3.72E-09
RIMS4	-0.199998251	4.79E-06	1.51E-05
RIN1	-0.043786036	0.321332	0.394205
RIN2	-0.102297794	0.020235	0.033674
RIN3	-0.263451427	1.27E-09	6.90E-09
RING1	-0.166034738	0.000154	0.000379
RINL	-0.138682545	0.001606	0.003289
RINT1	0.292080244	1.38E-11	1.01E-10
RIOK1	0.391133615	2.84E-20	7.75E-19
RIOK2	0.040847386	0.35491	0.429493
RIOK3	0.042367099	0.337278	0.411244
RIPK1	-0.101003257	0.021881	0.036178
RIPK2	0.417278188	4.09E-23	1.71E-21
RIPK3	-0.339276892	2.44E-15	3.19E-14
RIPK4	-0.238975246	4.02E-08	1.76E-07
RIPPLY1	-0.10074756	0.02222	0.03669
RIPPLY2	0.112823772	0.010397	0.018387
RIT1	0.041545348	0.346741	0.421138
RIT2	0.045511216	0.302617	0.374324

RLBP1	0.092215285	0.036432	0.057375
RLF	-0.022809917	0.605541	0.670371
RLIM	0.062457583	0.156973	0.212113
RLN1	0.011985137	0.786133	0.828146
RLN2	-0.071140386	0.106842	0.150763
RLN3	0.015142803	0.731727	0.782796
RLTPR	-0.197874751	6.06E-06	1.88E-05
RMI1	0.319049282	1.19E-13	1.20E-12
RMND1	0.079400155	0.071807	0.10594
RMND5A	-0.000402634	0.992727	0.994633
RMND5B	-0.229151272	1.46E-07	5.85E-07
RMRP	-0.009757294	0.825174	0.860752
RMST	-0.153249953	0.000483	0.001086
RNASE10	0.177498114	5.11E-05	0.000136
RNASE11	0.092255261	0.036351	0.057274
RNASE13	-0.2637504	1.21E-09	6.63E-09
RNASE1	-0.402896916	1.61E-21	5.34E-20
RNASE2	0.040663943	0.357076	0.43154
RNASE3	0.057944124	0.189227	0.249688
RNASE4	-0.144539451	0.001004	0.002135
RNASE6	-0.200241973	4.66E-06	1.47E-05
RNASE7	-0.103476043	0.018832	0.031543
RNASE8	-0.098939135	0.024746	0.040437
RNASE9	-0.061646204	0.162442	0.218573
RNASEH1	0.379624537	4.24E-19	9.78E-18
RNASEH2A	0.370011347	3.73E-18	7.41E-17
RNASEH2B	0.060495301	0.170445	0.228081
RNASEH2C	-0.107876056	0.014313	0.024604
RNASEK	0.003704069	0.933172	0.947728
RNASEL	-0.394547155	1.25E-20	3.55E-19
RNASEN	0.04934622	0.263651	0.332819
RNASET2	-0.085398316	0.052767	0.080303
RND1	0.056108566	0.203653	0.266258
RND2	-0.005942007	0.892992	0.916233
RND3	0.098055764	0.026068	0.042406
RNF103	-0.053510294	0.225413	0.290718
RNF10	0.018860505	0.669371	0.727378
RNF111	-0.017878649	0.685641	0.742474
RNF112	-0.152732564	0.000505	0.001131
RNF113A	0.034671456	0.432369	0.507022
RNF113B	0.007510576	0.864986	0.894014
RNF114	0.010741471	0.807868	0.846402
RNF115	0.243512751	2.18E-08	9.90E-08
RNF11	-0.022883182	0.604383	0.669312

RNF121	0.152397116	0.00052	0.001162
RNF122	-0.111299946	0.011487	0.020136
RNF123	-0.159879851	0.00027	0.000636
RNF125	-0.188451246	1.67E-05	4.84E-05
RNF126P1	-0.067833556	0.124191	0.172288
RNF126	0.070624446	0.109415	0.154078
RNF128	-0.092036721	0.036797	0.057882
RNF130	-0.295862454	7.29E-12	5.58E-11
RNF133	-0.213742221	9.81E-07	3.45E-06
RNF135	-0.153790329	0.000461	0.00104
RNF138P1	0.076438626	0.083098	0.120595
RNF138	0.157063424	0.000346	0.000799
RNF139	0.184717871	2.46E-05	6.92E-05
RNF13	-0.020311034	0.645618	0.706096
RNF141	-0.090691583	0.039652	0.061962
RNF144A	-0.013731557	0.755895	0.803106
RNF144B	-0.32487884	4.01E-14	4.33E-13
RNF145	-0.185649819	2.24E-05	6.33E-05
RNF146	-0.336659657	4.10E-15	5.17E-14
RNF148	-0.099562907	0.023848	0.039081
RNF149	0.144167365	0.001035	0.002195
RNF14	0.103511194	0.018791	0.031486
RNF150	-0.129758984	0.003177	0.006178
RNF151	0.008895973	0.84039	0.873334
RNF152	-0.038485473	0.38344	0.458082
RNF157	0.041521609	0.347017	0.421396
RNF160	0.027908615	0.527432	0.59954
RNF165	-0.239399268	3.80E-08	1.66E-07
RNF166	-0.267340878	7.06E-10	4.02E-09
RNF167	-0.063621853	0.149369	0.203074
RNF168	0.000909416	0.983574	0.987775
RNF169	-0.039583666	0.370005	0.444824
RNF170	0.004657031	0.916036	0.934312
RNF175	-0.405766797	7.83E-22	2.70E-20
RNF17	-0.083869733	0.057167	0.086264
RNF180	-0.162151579	0.00022	0.000528
RNF181	0.050655339	0.251177	0.319298
RNF182	-0.101747518	0.020921	0.034741
RNF183	0.123415849	0.005037	0.009458
RNF185	-0.144986098	0.000968	0.002063
RNF186	0.22820551	1.65E-07	6.54E-07
RNF187	0.036524539	0.40816	0.482838
RNF19A	-0.10943402	0.012959	0.022462
RNF19B	-0.125573783	0.004316	0.008193

RNF207	-0.22546365	2.33E-07	9.05E-07
RNF208	-0.148973717	0.000695	0.001523
RNF20	-0.078162351	0.076363	0.11198
RNF212	-0.047893163	0.277988	0.348562
RNF213	-0.052220728	0.236807	0.303456
RNF214	0.049687387	0.26036	0.329184
RNF215	-0.04864479	0.270507	0.340384
RNF216L	0.108944919	0.013372	0.023107
RNF216	-0.003607969	0.934902	0.948955
RNF217	-0.051202378	0.246088	0.313759
RNF219	0.1544955	0.000434	0.000984
RNF220	-0.071151412	0.106787	0.150707
RNF222	-0.084685292	0.054783	0.08308
RNF24	0.160075224	0.000265	0.000625
RNF25	0.014336945	0.745497	0.794338
RNF26	0.174692335	6.74E-05	0.000176
RNF2	0.309587598	6.68E-13	6.01E-12
RNF31	-0.016559199	0.707734	0.762146
RNF32	-0.053867623	0.222326	0.287182
RNF34	0.348990106	3.39E-16	5.03E-15
RNF38	-0.244608179	1.87E-08	8.61E-08
RNF39	-0.180535043	3.77E-05	0.000103
RNF40	-0.082465816	0.061472	0.092176
RNF41	0.192393472	1.10E-05	3.27E-05
RNF43	-0.138893779	0.001579	0.003239
RNF44	-0.153671823	0.000466	0.00105
RNF4	0.153261047	0.000483	0.001085
RNF5P1	0.009450271	0.83059	0.86513
RNF5	-0.043092112	0.329068	0.402499
RNF6	0.124941836	0.004517	0.008547
RNF7	0.268206133	6.19E-10	3.55E-09
RNF8	0.08520025	0.05332	0.081071
RNFT1	-0.015573335	0.724405	0.776376
RNFT2	0.207026588	2.16E-06	7.16E-06
RNGTT	0.146216839	0.000875	0.001878
RNH1	-0.255721125	3.93E-09	1.98E-08
RNLS	-0.003617674	0.934727	0.948826
RNMTL1	0.156810123	0.000354	0.000815
RNMT	-0.016254827	0.712867	0.766476
RNPC3	-0.407843352	4.64E-22	1.64E-20
RNPEPL1	-0.17403564	7.18E-05	0.000187
RNPEP	-0.008007074	0.856155	0.886647
RNPS1	0.059283327	0.179186	0.238317
RNU11	-0.016280867	0.712427	0.766044

RNU4ATAC	-0.041283453	0.349792	0.424405
RNU6ATAC	0.037227621	0.39919	0.473869
ROBLD3	0.07112914	0.106897	0.15083
ROBO1	0.005687728	0.897546	0.919913
ROBO2	-0.426568837	3.46E-24	1.74E-22
ROBO3	-0.26052019	1.95E-09	1.03E-08
ROBO4	-0.248671612	1.07E-08	5.09E-08
ROCK1	-0.048235565	0.274563	0.344916
ROCK2	0.053185087	0.228249	0.29394
ROD1	0.206182378	2.38E-06	7.84E-06
ROGDI	-0.41679942	4.63E-23	1.91E-21
ROM1	-0.28849753	2.50E-11	1.75E-10
ROMO1	0.189789053	1.45E-05	4.25E-05
ROPN1B	-0.191479606	1.21E-05	3.59E-05
ROPN1L	-0.099791013	0.023527	0.038618
ROPN1	-0.084455445	0.055446	0.083965
ROR1	-0.11566951	0.008604	0.015443
ROR2	-0.043840286	0.320732	0.393542
RORA	-0.396012326	8.76E-21	2.56E-19
RORB	-0.10270119	0.019745	0.032946
RORC	-0.224525914	2.62E-07	1.01E-06
ROS1	-0.337725972	3.32E-15	4.25E-14
RP1-177G6.2	-0.260469169	1.97E-09	1.04E-08
RP1L1	0.055298945	0.210263	0.273758
RP1	-0.122233367	0.005476	0.010218
RP2	0.140733106	0.001365	0.002833
RP9P	0.142254345	0.001208	0.002533
RP9	0.106484578	0.015626	0.026623
RPA1	0.066649354	0.130911	0.18067
RPA2	-0.058501511	0.184999	0.244831
RPA3	0.253202503	5.63E-09	2.79E-08
RPA4	-0.182170908	3.20E-05	8.81E-05
RPAIN	-0.087925854	0.046112	0.071084
RPAP1	-0.022942783	0.603442	0.668491
RPAP2	0.333329805	7.89E-15	9.46E-14
RPAP3	0.335516039	5.14E-15	6.38E-14
RPE65	0.075845971	0.085521	0.123734
RPE	0.420782025	1.63E-23	7.19E-22
RPF1	0.226083549	2.15E-07	8.41E-07
RPF2	0.353554663	1.31E-16	2.05E-15
RPGRIP1L	0.060562045	0.169973	0.227587
RPGRIP1	-0.212746651	1.10E-06	3.84E-06
RPGR	-0.164234853	0.000181	0.000443
RPH3AL	0.019981208	0.650989	0.710842

RPH3A	-0.038734087	0.380373	0.455072
RPIA	0.111694919	0.011195	0.019669
RPL10A	0.096549155	0.028463	0.045929
RPL10L	0.174183636	7.08E-05	0.000184
RPL10	0.075563724	0.086695	0.125215
RPL11	-0.071359359	0.105764	0.149379
RPL12	0.124404606	0.004694	0.008865
RPL13AP17	-0.355396735	8.89E-17	1.42E-15
RPL13AP20	0.078807282	0.073961	0.108717
RPL13AP3	0.051764682	0.240932	0.308031
RPL13AP6	0.123958831	0.004846	0.009131
RPL13A	0.056806476	0.198076	0.259821
RPL13P5	0.026297008	0.551558	0.621591
RPL13	0.069224641	0.116643	0.163107
RPL14	0.130638224	0.002976	0.005817
RPL15	0.026986478	0.541171	0.611886
RPL17	0.133803044	0.002344	0.004667
RPL18A	0.131358126	0.00282	0.005533
RPL18	0.092840099	0.035178	0.055597
RPL19P12	0.120107409	0.006354	0.011701
RPL19	0.093623748	0.033656	0.053391
RPL21P44	-0.181136387	3.55E-05	9.72E-05
RPL21	0.151280755	0.000572	0.001269
RPL22L1	0.253239485	5.60E-09	2.78E-08
RPL22	0.0776476	0.078326	0.11446
RPL23AP32	-0.148737865	0.000709	0.001551
RPL23AP53	-0.198103999	5.91E-06	1.83E-05
RPL23AP64	-0.259114882	2.40E-09	1.25E-08
RPL23AP7	0.179601516	4.14E-05	0.000112
RPL23AP82	0.050769409	0.25011	0.318136
RPL23A	0.206387858	2.32E-06	7.67E-06
RPL23P8	-0.000219134	0.996042	0.997339
RPL23	0.089904747	0.041406	0.064497
RPL24	0.100755105	0.02221	0.036679
RPL26L1	0.211210078	1.32E-06	4.55E-06
RPL26	0.133735517	0.002356	0.00469
RPL27A	0.141677825	0.001266	0.002644
RPL27	0.250894786	7.81E-09	3.80E-08
RPL28	-0.003649549	0.934153	0.948388
RPL29P2	0.075174614	0.088334	0.127363
RPL29	0.136397082	0.00192	0.00388
RPL30	0.22803763	1.68E-07	6.67E-07
RPL31P11	0.117005589	0.007861	0.014237
RPL31	0.1588257	0.000296	0.000693

RPL32P3	-0.30667033	1.12E-12	9.76E-12
RPL32	0.126199397	0.004125	0.007859
RPL34	0.028052266	0.525308	0.597329
RPL35A	0.209429966	1.63E-06	5.53E-06
RPL35	0.158223576	0.000313	0.000727
RPL36AL	0.295544218	7.69E-12	5.87E-11
RPL36A	0.158886543	0.000295	0.000689
RPL36	0.094975264	0.031164	0.049825
RPL37A	0.141191695	0.001316	0.002739
RPL37	0.10568033	0.016433	0.027873
RPL38	0.185461682	2.28E-05	6.45E-05
RPL39L	0.239862333	3.57E-08	1.57E-07
RPL39	0.213173306	1.05E-06	3.67E-06
RPL3L	0.042425787	0.336609	0.410553
RPL3	0.0416868	0.3451	0.419298
RPL41	0.205021812	2.71E-06	8.87E-06
RPL4	0.193219844	1.01E-05	3.01E-05
RPL5	0.157740749	0.000326	0.000757
RPL6	0.224365881	2.67E-07	1.03E-06
RPL7A	0.16019917	0.000262	0.000619
RPL7L1	0.338436178	2.88E-15	3.73E-14
RPL7	0.222560854	3.35E-07	1.27E-06
RPL8	0.246295174	1.49E-08	6.92E-08
RPL9	0.132739568	0.002541	0.005029
RPLP0P2	0.2489083	1.03E-08	4.94E-08
RPLP0	0.338653104	2.76E-15	3.59E-14
RPLP1	0.096950495	0.027807	0.044961
RPLP2	0.086045217	0.050991	0.077868
RPN1	0.260481359	1.96E-09	1.04E-08
RPN2	0.256264409	3.63E-09	1.84E-08
RPP14	0.19812177	5.89E-06	1.83E-05
RPP21	0.102350047	0.020171	0.033584
RPP25	0.223269246	3.07E-07	1.17E-06
RPP30	0.31246235	3.98E-13	3.70E-12
RPP38	0.250516981	8.24E-09	3.99E-08
RPP40	0.367813738	6.07E-18	1.18E-16
RPPH1	0.063281172	0.151565	0.205709
RPRD1A	0.215069811	8.37E-07	2.97E-06
RPRD1B	0.180083354	3.95E-05	0.000107
RPRD2	-0.106548734	0.015564	0.026523
RPRML	-0.09240546	0.036046	0.056848
RPRM	-0.06114089	0.16592	0.222772
RPS10P7	-0.223229375	3.08E-07	1.18E-06
RPS10	0.24269779	2.43E-08	1.10E-07

RPS11	0.100223689	0.022927	0.037746
RPS12	0.113471426	0.009962	0.017698
RPS13	0.117220109	0.007748	0.014045
RPS14	0.077155542	0.080241	0.116975
RPS15AP10	-0.288442324	2.52E-11	1.77E-10
RPS15A	0.159555508	0.000278	0.000653
RPS15	0.057077488	0.195941	0.257392
RPS16	0.231190577	1.12E-07	4.58E-07
RPS17	0.201683225	3.96E-06	1.26E-05
RPS18	0.217202498	6.47E-07	2.34E-06
RPS19BP1	0.048199039	0.274927	0.345243
RPS19	0.176116649	5.86E-05	0.000154
RPS20	0.083187822	0.059225	0.089061
RPS21	0.186674859	2.01E-05	5.75E-05
RPS23	0.069035148	0.117649	0.164296
RPS24	0.151454584	0.000563	0.001252
RPS25	0.126056506	0.004168	0.007933
RPS26P11	0.163348937	0.000197	0.000477
RPS26	0.288423686	2.53E-11	1.77E-10
RPS27A	0.230656442	1.20E-07	4.89E-07
RPS27L	-0.02180735	0.621487	0.684688
RPS27	0.034137641	0.439493	0.513839
RPS28	-0.120869902	0.006026	0.011147
RPS29	0.29627665	6.79E-12	5.22E-11
RPS2P32	0.030878821	0.484417	0.558135
RPS2	0.149985587	0.000638	0.001405
RPS3A	0.157726694	0.000327	0.000757
RPS3	0.241393231	2.91E-08	1.29E-07
RPS4X	0.036725979	0.405578	0.48034
RPS4Y1	0.173027875	7.92E-05	0.000204
RPS4Y2	0.043792944	0.321256	0.39416
RPS5	0.157494749	0.000333	0.000771
RPS6KA1	-0.389124452	4.59E-20	1.20E-18
RPS6KA2	-0.355144028	9.38E-17	1.49E-15
RPS6KA3	-0.186620043	2.02E-05	5.78E-05
RPS6KA4	0.035251888	0.424698	0.499316
RPS6KA5	-0.216110948	7.38E-07	2.65E-06
RPS6KA6	-0.061083215	0.166321	0.223186
RPS6KB1	0.153698964	0.000465	0.001048
RPS6KB2	0.153130709	0.000488	0.001096
RPS6KC1	0.208335282	1.85E-06	6.24E-06
RPS6KL1	-0.08106035	0.066047	0.098276
RPS6	0.134611182	0.002204	0.00441
RPS7	0.266865978	7.58E-10	4.29E-09

RPS8	0.119176197	0.006777	0.012421
RPS9	0.012885077	0.770508	0.815381
RPSAP52	0.149863886	0.000645	0.001419
RPSAP58	0.200846755	4.35E-06	1.38E-05
RPSAP9	0.178488599	4.63E-05	0.000124
RPSA	0.197786433	6.12E-06	1.89E-05
RPTN	-0.000404917	0.992686	0.994633
RPTOR	-0.014329004	0.745633	0.794441
RPUSD1	0.12251882	0.005367	0.01003
RPUSD2	0.134184962	0.002277	0.004544
RPUSD3	0.140543459	0.001386	0.002874
RPUSD4	0.163771835	0.000189	0.00046
RQCD1	0.273253553	2.85E-10	1.72E-09
RRAD	-0.309870057	6.35E-13	5.74E-12
RRAGA	0.086266082	0.050397	0.077096
RRAGB	-0.087383706	0.047476	0.072929
RRAGC	0.038920906	0.378078	0.452841
RRAGD	-0.17913168	4.34E-05	0.000117
RRAS2	0.007039415	0.873382	0.901201
RRAS	-0.128377702	0.003519	0.006784
RRBP1	-0.181055371	3.58E-05	9.80E-05
RREB1	-0.004634514	0.91644	0.93463
RRH	-0.067849201	0.124104	0.172215
RRM1	0.32502623	3.90E-14	4.22E-13
RRM2B	-0.225413818	2.34E-07	9.10E-07
RRM2	0.508480491	3.26E-35	9.88E-33
RRN3P1	-0.282294804	6.85E-11	4.52E-10
RRN3P2	-0.228031802	1.68E-07	6.68E-07
RRN3P3	-0.298855134	4.38E-12	3.44E-11
RRN3	0.047400103	0.282971	0.353745
RRP12	0.199124281	5.27E-06	1.65E-05
RRP15	0.210948781	1.37E-06	4.69E-06
RRP1B	0.181638186	3.38E-05	9.27E-05
RRP1	0.092262711	0.036336	0.057267
RRP7A	0.114239317	0.009467	0.016881
RRP7B	-0.169179016	0.000114	0.000288
RRP8	0.05192018	0.23952	0.306402
RRP9	0.194748069	8.53E-06	2.58E-05
RRS1	0.358398698	4.70E-17	7.89E-16
RS1	-0.336402725	4.31E-15	5.42E-14
RSAD1	-0.214910727	8.53E-07	3.03E-06
RSAD2	-0.137888803	0.001709	0.003487
RSBN1L	-0.078704256	0.07434	0.109226
RSBN1	-0.178467578	4.64E-05	0.000125

RSC1A1	-0.034684641	0.432194	0.506906
RSF1	0.002221798	0.959885	0.969244
RSL1D1	0.239167055	3.92E-08	1.71E-07
RSL24D1	0.275668748	1.96E-10	1.21E-09
RSPH10B2	-0.236129629	5.88E-08	2.50E-07
RSPH1	-0.312278053	4.12E-13	3.82E-12
RSPH3	-0.066821493	0.129917	0.17946
RSPH4A	-0.243216353	2.27E-08	1.03E-07
RSPH6A	0.083763567	0.057483	0.086702
RSPH9	-0.142187395	0.001215	0.002546
RSPO1	-0.396580859	7.62E-21	2.26E-19
RSPO2	-0.363814275	1.46E-17	2.65E-16
RSPO3	0.232964131	8.91E-08	3.70E-07
RSPO4	-0.270276896	4.52E-10	2.64E-09
RSPRY1	0.121813752	0.00564	0.010495
RSRC1	0.35405303	1.18E-16	1.85E-15
RSRC2	0.01175851	0.790082	0.831517
RSU1	-0.019404262	0.660427	0.71947
RTBDN	0.037164916	0.399985	0.47456
RTCD1	0.277396508	1.49E-10	9.38E-10
RTDR1	-0.155695794	0.000391	0.000892
RTEL1	-0.154108083	0.000449	0.001015
RTF1	-0.002655026	0.952071	0.962862
RTKN2	-0.020479323	0.642885	0.703607
RTKN	0.207872804	1.96E-06	6.55E-06
RTL1	0.145643733	0.000917	0.001961
RTN1	-0.354151528	1.16E-16	1.82E-15
RTN2	-0.091849213	0.037185	0.05844
RTN3	0.065285146	0.138998	0.190646
RTN4IP1	0.098522363	0.025362	0.041353
RTN4RL1	-0.389009253	4.72E-20	1.24E-18
RTN4RL2	-0.083634404	0.05787	0.087213
RTN4R	-0.229024507	1.48E-07	5.93E-07
RTN4	0.186384569	2.07E-05	5.91E-05
RTP1	-0.134974123	0.002143	0.004301
RTP2	-0.086928946	0.048647	0.074602
RTP3	0.102655538	0.0198	0.033032
RTP4	-0.133861381	0.002334	0.004648
RTTN	0.067799882	0.124378	0.1725
RUFY1	-0.197310133	6.45E-06	1.99E-05
RUFY2	-0.02391739	0.588142	0.654517
RUFY3	-0.292201722	1.35E-11	9.92E-11
RUFY4	-0.280449916	9.21E-11	5.96E-10
RUNDC1	-0.045736063	0.300232	0.371742

RUNDC2A	-0.14955745	0.000662	0.001455
RUNDC2C	-0.372663762	2.06E-18	4.24E-17
RUNDC3A	0.060019742	0.173836	0.232137
RUNDC3B	-0.100522605	0.022521	0.037126
RUNX1T1	-0.236401422	5.67E-08	2.42E-07
RUNX1	-0.271766305	3.59E-10	2.13E-09
RUNX2	0.070331072	0.1109	0.155894
RUNX3	-0.247293309	1.29E-08	6.08E-08
RUSC1	0.050523025	0.252418	0.320606
RUSC2	-0.123727752	0.004926	0.009264
RUVBL1	0.407929961	4.54E-22	1.61E-20
RUVBL2	0.240425768	3.31E-08	1.47E-07
RWDD1	0.109663727	0.01277	0.02215
RWDD2A	0.113159003	0.01017	0.018032
RWDD2B	0.025320927	0.566429	0.635092
RWDD3	0.015739002	0.721594	0.77382
RWDD4A	0.228732254	1.54E-07	6.14E-07
RXFP1	-0.275136328	2.13E-10	1.31E-09
RXFP2	-0.169362224	0.000112	0.000284
RXFP3	0.011427018	0.795868	0.836504
RXFP4	-0.201909602	3.86E-06	1.23E-05
RXRA	-0.345943106	6.34E-16	9.03E-15
RXRB	-0.164967291	0.00017	0.000416
RXRG	-0.349391308	3.12E-16	4.66E-15
RYBP	-0.022720309	0.606959	0.671532
RYK	0.055816248	0.206022	0.268968
RYR1	-0.305114448	1.48E-12	1.26E-11
RYR2	-0.293448438	1.10E-11	8.14E-11
RYR3	-0.186667467	2.01E-05	5.75E-05
S100A10	0.093949396	0.033041	0.052493
S100A11	0.107255949	0.014886	0.025514
S100A12	0.152832421	0.000501	0.001122
S100A13	-0.049580231	0.26139	0.330278
S100A14	-0.007816334	0.859546	0.889495
S100A16	0.198079993	5.92E-06	1.84E-05
S100A1	-0.194585251	8.68E-06	2.62E-05
S100A2	0.082425936	0.061598	0.092337
S100A3	0.018403649	0.676923	0.734386
S100A4	-0.032854172	0.456895	0.530986
S100A5	0.057559098	0.192189	0.253111
S100A6	0.024857937	0.573549	0.641456
S100A7A	0.077315855	0.079613	0.11617
S100A7	0.138929455	0.001575	0.00323
S100A8	0.116403725	0.008189	0.01478

S100A9	0.106624687	0.01549	0.02641
S100B	-0.248884423	1.04E-08	4.95E-08
S100G	0.033486864	0.448269	0.52248
S100PBP	-0.182694292	3.03E-05	8.39E-05
S100P	0.275945857	1.88E-10	1.16E-09
S100Z	-0.301924869	2.58E-12	2.10E-11
S1PR1	-0.22833245	1.62E-07	6.44E-07
S1PR2	-0.214674814	8.77E-07	3.11E-06
S1PR3	0.143787993	0.001067	0.002258
S1PR4	-0.290615034	1.76E-11	1.27E-10
S1PR5	-0.024843805	0.573767	0.641628
SAA1	0.123490078	0.00501	0.009412
SAA2	0.11446966	0.009323	0.01664
SAA3P	-0.03231557	0.464311	0.538196
SAA4	0.058989581	0.181354	0.240713
SAAL1	0.291342713	1.56E-11	1.13E-10
SAC3D1	0.220295017	4.43E-07	1.65E-06
SACM1L	-0.180791848	3.68E-05	0.0001
SACS	0.211775406	1.24E-06	4.27E-06
SAE1	0.261996917	1.57E-09	8.46E-09
SAFB2	-0.203301534	3.30E-06	1.06E-05
SAFB	-0.096255483	0.028951	0.046634
SAGE1	0.021040284	0.633809	0.695388
SAG	0.017873312	0.68573	0.74253
SALL1	-0.055059426	0.212248	0.275965
SALL2	-0.241687095	2.79E-08	1.25E-07
SALL3	0.075106908	0.088622	0.127704
SALL4	-0.026740591	0.544864	0.615512
SAMD10	-0.115874798	0.008486	0.015256
SAMD11	-0.196194273	7.28E-06	2.22E-05
SAMD12	-0.280271713	9.47E-11	6.13E-10
SAMD13	0.091794262	0.037299	0.058601
SAMD14	-0.128238338	0.003555	0.006846
SAMD1	0.173171684	7.81E-05	0.000202
SAMD3	-0.179370593	4.24E-05	0.000115
SAMD4A	-0.084237348	0.056082	0.084838
SAMD4B	0.024563043	0.578106	0.645469
SAMD5	-0.123415966	0.005037	0.009458
SAMD7	0.04471888	0.31112	0.383136
SAMD8	0.224883115	2.51E-07	9.69E-07
SAMD9L	-0.134334597	0.002251	0.004496
SAMD9	-0.072888931	0.098475	0.140334
SAMHD1	-0.145863538	0.0009	0.001929
SAMM50	0.076691642	0.08208	0.1193

SAMSN1	-0.068230209	0.122001	0.16978
SAP130	0.053854599	0.222438	0.287308
SAP18	0.145054344	0.000962	0.002053
SAP30BP	-0.064608707	0.143147	0.195706
SAP30L	-0.320951955	8.38E-14	8.56E-13
SAP30	0.197674229	6.19E-06	1.91E-05
SAPS1	-0.039428586	0.371884	0.446777
SAPS2	-0.365024773	1.12E-17	2.07E-16
SAPS3	0.209108227	1.69E-06	5.73E-06
SAR1A	0.180755696	3.69E-05	0.000101
SAR1B	0.185348774	2.31E-05	6.52E-05
SARDH	-0.230275096	1.26E-07	5.12E-07
SARM1	-0.448915253	6.63E-27	4.78E-25
SARNP	0.315466809	2.31E-13	2.22E-12
SARS2	0.077626733	0.078407	0.114561
SARS	0.003920475	0.929278	0.944637
SART1	-0.012658354	0.774436	0.818801
SART3	0.126118788	0.004149	0.0079
SASH1	-0.259067592	2.42E-09	1.26E-08
SASH3	-0.223808507	2.87E-07	1.10E-06
SASS6	0.345011097	7.67E-16	1.08E-14
SAT1	-0.084365521	0.055707	0.084303
SAT2	-0.060102867	0.173239	0.231418
SATB1	-0.335103449	5.57E-15	6.88E-14
SATB2	-0.00457852	0.917446	0.935196
SATL1	0.022879905	0.604435	0.669332
SAV1	0.258295967	2.70E-09	1.40E-08
SBDSP1	0.08557577	0.052275	0.079627
SBDS	0.060724719	0.168826	0.226219
SBF1P1	-0.026469047	0.548957	0.619219
SBF1	-0.224738006	2.55E-07	9.85E-07
SBF2	0.034070722	0.440391	0.514708
SBK1	-0.18524426	2.33E-05	6.58E-05
SBK2	0.112052429	0.010937	0.019259
SBNO1	0.212833255	1.09E-06	3.81E-06
SBNO2	-0.048278245	0.274138	0.344447
SBSN	0.226358664	2.08E-07	8.15E-07
SC4MOL	0.170815086	9.79E-05	0.00025
SC5DL	0.002964058	0.946501	0.95781
SC65	0.298462551	4.68E-12	3.66E-11
SCAF1	-0.079255738	0.072327	0.106597
SCAI	-0.348444142	3.80E-16	5.59E-15
SCAMP1	0.187095245	1.93E-05	5.52E-05
SCAMP2	-0.260268024	2.03E-09	1.07E-08

SCAMP3	0.015866665	0.719431	0.771914
SCAMP4	-0.195100432	8.21E-06	2.49E-05
SCAMP5	-0.042498214	0.335784	0.409671
SCAND1	0.019568434	0.657735	0.716995
SCAND2	-0.274203507	2.46E-10	1.50E-09
SCAND3	-0.104207953	0.018003	0.030293
SCAPER	-0.307189506	1.02E-12	8.95E-12
SCAP	-0.145221129	0.000949	0.002026
SCARA3	-0.324290582	4.48E-14	4.78E-13
SCARA5	-0.20309198	3.38E-06	1.09E-05
SCARB1	0.202638011	3.56E-06	1.14E-05
SCARB2	-0.016196709	0.713848	0.767366
SCARF1	-0.40152664	2.26E-21	7.31E-20
SCARF2	-0.165500829	0.000161	0.000397
SCARNA10	0.026174318	0.553417	0.623159
SCARNA11	-0.0356882	0.418985	0.493691
SCARNA12	0.110094242	0.01242	0.021597
SCARNA15	-0.009033871	0.837949	0.871115
SCARNA16	-0.015076753	0.732852	0.783706
SCARNA17	-0.0943929	0.032218	0.051337
SCARNA18	0.085139714	0.05349	0.081312
SCARNA1	-0.010196721	0.817436	0.854367
SCARNA20	0.079131371	0.072777	0.107142
SCARNA21	0.030924746	0.483767	0.557483
SCARNA22	-0.038480019	0.383508	0.458082
SCARNA2	-0.06276234	0.154955	0.20967
SCARNA3	0.104214046	0.017996	0.030284
SCARNA4	-0.092189703	0.036484	0.057444
SCARNA5	-0.042108887	0.340234	0.414392
SCARNA6	-0.065999031	0.134719	0.18544
SCARNA7	-0.187073987	1.93E-05	5.53E-05
SCARNA9L	0.041212983	0.350616	0.425172
SCARNA9	-0.103758901	0.018508	0.03106
SCCPDH	0.201396772	4.09E-06	1.30E-05
SCD5	-0.261428438	1.71E-09	9.13E-09
SCD	0.238040109	4.56E-08	1.97E-07
SCEL	-0.078257138	0.076006	0.11151
SCFD1	0.373042128	1.89E-18	3.91E-17
SCFD2	0.075209147	0.088188	0.12716
SCG2	0.064975849	0.140883	0.192888
SCG3	0.116374344	0.008205	0.014808
SCG5	0.157069416	0.000346	0.000799
SCGB1A1	-0.280287467	9.45E-11	6.11E-10
SCGB1C1	0.052720094	0.232348	0.298507

SCGB1D1	-0.012821352	0.771611	0.816289
SCGB1D2	-0.036541469	0.407943	0.482683
SCGB2A1	-0.278874376	1.18E-10	7.53E-10
SCGB2A2	0.015251346	0.729878	0.781111
SCGB3A1	-0.420442817	1.78E-23	7.81E-22
SCGB3A2	-0.305299454	1.43E-12	1.22E-11
SCGBL	-0.234917833	6.90E-08	2.91E-07
SCGN	-0.063212691	0.152009	0.206172
SCHIP1	0.155588844	0.000394	0.0009
SCIN	-0.072267734	0.101386	0.144009
SCLT1	0.228969052	1.49E-07	5.97E-07
SCLY	0.034349115	0.436663	0.511051
SCMH1	-0.276060127	1.84E-10	1.14E-09
SCML1	-0.085010389	0.053856	0.081823
SCML2	0.239192198	3.91E-08	1.71E-07
SCML4	-0.266258562	8.31E-10	4.68E-09
SCN10A	-0.191265597	1.24E-05	3.66E-05
SCN11A	-0.398063658	5.31E-21	1.61E-19
SCN1A	-0.365702472	9.67E-18	1.82E-16
SCN1B	-0.093995939	0.032954	0.052376
SCN2A	-0.044270609	0.316001	0.38838
SCN2B	-0.351043127	2.22E-16	3.35E-15
SCN3A	0.085715748	0.051889	0.079088
SCN3B	-0.100790175	0.022163	0.036605
SCN4A	0.062931153	0.153846	0.208403
SCN4B	-0.463987598	7.43E-29	6.90E-27
SCN5A	-0.111248282	0.011526	0.020193
SCN7A	-0.367650159	6.30E-18	1.21E-16
SCN8A	0.022870399	0.604585	0.669422
SCN9A	-0.004596958	0.917115	0.935079
SCNM1	0.214311373	9.16E-07	3.24E-06
SCNN1A	-0.055771136	0.206389	0.269364
SCNN1B	-0.359159489	3.99E-17	6.79E-16
SCNN1D	-0.294067374	9.87E-12	7.39E-11
SCNN1G	-0.185643261	2.24E-05	6.34E-05
SCO1	0.366818516	7.56E-18	1.44E-16
SCO2	-0.026334558	0.55099	0.621161
SCOC	0.226493921	2.05E-07	8.03E-07
SCP2	-0.201641836	3.98E-06	1.27E-05
SCPEP1	-0.272427122	3.24E-10	1.93E-09
SCRG1	-0.162364175	0.000215	0.000518
SCRIB	0.064131884	0.146128	0.199223
SCRN1	-0.079776195	0.070467	0.104133
SCRN2	-0.28417751	5.06E-11	3.39E-10

SCRN3	-0.047619394	0.280747	0.351404
SCRT1	-0.029236885	0.507959	0.580474
SCRT2	0.049222595	0.26485	0.334164
SCTR	-0.428757739	1.91E-24	9.81E-23
SCT	0.012738668	0.773044	0.817545
SCUBE1	-0.240139937	3.44E-08	1.52E-07
SCUBE2	-0.271245032	3.89E-10	2.30E-09
SCUBE3	-0.13041063	0.003027	0.005906
SCXB	-0.065335786	0.138691	0.190278
SCYL1	0.026284389	0.551749	0.621666
SCYL2	0.281086746	8.32E-11	5.42E-10
SCYL3	-0.153303428	0.000481	0.001081
SDAD1	0.277815278	1.40E-10	8.82E-10
SDC1	-0.318458825	1.33E-13	1.33E-12
SDC2	-0.088164299	0.045522	0.07024
SDC3	-0.26491493	1.02E-09	5.65E-09
SDC4P	-0.166407914	0.000148	0.000367
SDC4	-0.228739987	1.54E-07	6.14E-07
SDCBP2	0.101381825	0.021388	0.035442
SDCBP	0.025533345	0.563176	0.632332
SDCCAG1	0.139211493	0.00154	0.003167
SDCCAG3	0.226751313	1.98E-07	7.78E-07
SDCCAG8	-0.155979795	0.000381	0.000871
SDF2L1	0.183452909	2.81E-05	7.81E-05
SDF2	0.08958934	0.042128	0.065484
SDF4	-0.055026776	0.21252	0.276265
SDHAF1	0.026365348	0.550524	0.620741
SDHAF2	0.221997909	3.59E-07	1.36E-06
SDHAP1	-0.231614967	1.06E-07	4.36E-07
SDHAP2	-0.183557212	2.78E-05	7.74E-05
SDHAP3	-0.328249271	2.11E-14	2.37E-13
SDHA	0.087459107	0.047285	0.072685
SDHB	0.215823027	7.64E-07	2.73E-06
SDHC	0.239051602	3.98E-08	1.74E-07
SDHD	0.145703802	0.000912	0.001953
SDK1	-0.130529559	0.003	0.005858
SDK2	-0.303475349	1.97E-12	1.64E-11
SDPR	-0.366334624	8.41E-18	1.59E-16
SDR16C5	-0.128035225	0.003608	0.00694
SDR39U1	-0.133832592	0.002339	0.004657
SDR42E1	-0.161535255	0.000232	0.000555
SDR9C7	0.054703879	0.21522	0.279374
SDSL	0.08244993	0.061522	0.09224
SDS	0.10395755	0.018283	0.03072

SEBOX	-0.074298764	0.092116	0.132082
SEC11A	0.177189892	5.27E-05	0.00014
SEC11C	0.050347438	0.254073	0.322603
SEC13	0.253851633	5.13E-09	2.56E-08
SEC14L1	-0.164107044	0.000184	0.000447
SEC14L2	0.067952427	0.123531	0.171552
SEC14L3	-0.337893474	3.21E-15	4.13E-14
SEC14L4	-0.245426756	1.67E-08	7.75E-08
SEC14L5	-0.241598244	2.83E-08	1.26E-07
SEC16A	-0.040459451	0.359502	0.434182
SEC16B	-0.122403512	0.005411	0.010103
SEC1	-0.168362464	0.000124	0.00031
SEC22A	0.100676627	0.022314	0.036822
SEC22B	0.1475195	0.000785	0.001702
SEC22C	-0.050442413	0.253177	0.321526
SEC23A	0.394244561	1.34E-20	3.80E-19
SEC23B	0.140655549	0.001374	0.00285
SEC23IP	0.24852888	1.09E-08	5.18E-08
SEC24A	0.249565546	9.42E-09	4.53E-08
SEC24B	0.0643023	0.145057	0.197993
SEC24C	-0.047107166	0.28596	0.356945
SEC24D	0.159237777	0.000286	0.00067
SEC31A	0.054571265	0.216335	0.280677
SEC31B	-0.399239396	3.97E-21	1.24E-19
SEC61A1	0.195605367	7.77E-06	2.36E-05
SEC61A2	0.046199604	0.295355	0.366657
SEC61B	0.240335074	3.35E-08	1.48E-07
SEC61G	0.391072111	2.89E-20	7.86E-19
SEC62	-0.003642824	0.934274	0.948462
SEC63	-0.094568867	0.031897	0.050865
SECISBP2L	-0.243091701	2.31E-08	1.04E-07
SECISBP2	-0.156691643	0.000358	0.000823
SECTM1	-0.046309111	0.29421	0.365532
SEH1L	0.324894276	3.99E-14	4.32E-13
SEL1L2	-0.129088046	0.003339	0.006469
SEL1L3	-0.151993937	0.000538	0.001199
SEL1L	0.137624106	0.001745	0.003553
SELENBP1	-0.441494567	5.58E-26	3.61E-24
SELE	-0.100656258	0.022342	0.036864
SELK	0.077898459	0.077365	0.11322
SELL	-0.182429567	3.11E-05	8.60E-05
SELM	-0.026806294	0.543876	0.614597
SELO	-0.252818412	5.95E-09	2.94E-08
SELPLG	-0.247645788	1.23E-08	5.81E-08

SELP		-0.298310541	4.80E-12	3.75E-11
SELS		0.228711834	1.54E-07	6.16E-07
SELT		0.216518875	7.03E-07	2.53E-06
SELV		0.128246992	0.003553	0.006842
SEMA3A		0.186399648	2.07E-05	5.90E-05
SEMA3B		-0.164070955	0.000184	0.000449
SEMA3C		0.053361035	0.226711	0.292129
SEMA3D		-0.077926576	0.077257	0.113097
SEMA3E		-0.05228728	0.23621	0.302884
SEMA3F		0.068635512	0.119794	0.166965
SEMA3G		-0.218871381	5.28E-07	1.94E-06
SEMA4A		-0.378218799	5.85E-19	1.32E-17
SEMA4B		0.202654657	3.55E-06	1.14E-05
SEMA4C		-0.105903231	0.016206	0.027509
SEMA4D		-0.234289176	7.49E-08	3.14E-07
SEMA4F		-0.141326932	0.001302	0.002713
SEMA4G		0.046161355	0.295755	0.367063
SEMA5A		-0.181056012	3.58E-05	9.80E-05
SEMA5B		0.082740585	0.060609	0.091004
SEMA6A		-0.166508743	0.000147	0.000364
SEMA6B		0.065860652	0.135541	0.186403
SEMA6C		-0.218267646	5.68E-07	2.08E-06
SEMA6D		-0.304263839	1.71E-12	1.45E-11
SEMA7A		0.107868999	0.01432	0.024611
SEMG1		0.006583368	0.881523	0.907026
SEMG2		0.073880755	0.093966	0.134484
SENP1		0.294921514	8.55E-12	6.47E-11
SENP2		0.116422774	0.008178	0.014764
SENP3		0.225695306	2.26E-07	8.80E-07
SENP5		0.09607359	0.029257	0.047081
SENP6		-0.091807971	0.03727	0.05857
SENP7		-0.266585756	7.91E-10	4.47E-09
SENP8		-0.145468413	0.00093	0.001988
	15-Sep	0.224445823	2.65E-07	1.02E-06
SEPHS1		0.342618678	1.25E-15	1.70E-14
SEPHS2		0.221935998	3.62E-07	1.36E-06
SEPN1		-0.31872829	1.27E-13	1.27E-12
SEPP1		-0.309041785	7.36E-13	6.59E-12
SEPSECS		-0.186897203	1.97E-05	5.63E-05
	10-Sep	0.111991575	0.01098	0.01932
	11-Sep	0.10086212	0.022067	0.036453
	12-Sep	-0.041167593	0.351147	0.425713
	14-Sep	0.092962711	0.034936	0.055241
	1-Sep	-0.242123667	2.63E-08	1.18E-07

	2-Sep	0.230981012	1.15E-07	4.70E-07
	3-Sep	-0.107196453	0.014942	0.025601
	4-Sep	-0.404267458	1.14E-21	3.85E-20
	5-Sep	-0.098149032	0.025926	0.042193
	6-Sep	-0.20567164	2.52E-06	8.28E-06
SEPT7L		-0.070434597	0.110374	0.155221
SEPT7P2		-0.257024703	3.25E-09	1.66E-08
	7-Sep	0.17908003	4.37E-05	0.000118
	8-Sep	-0.039270748	0.373803	0.448715
	9-Sep	-0.10161776	0.021086	0.034985
SEPW1		-0.257353024	3.10E-09	1.59E-08
SEPX1		0.236071306	5.92E-08	2.52E-07
SERAC1		-0.000605895	0.989056	0.991885
SERBP1		0.320822903	8.58E-14	8.75E-13
SERF1A		0.064173018	0.145869	0.198951
SERF2		0.069554945	0.114904	0.161014
SERGEF		-0.139140902	0.001549	0.003183
SERHL2		-0.153632041	0.000467	0.001053
SERHL		-0.061782773	0.161512	0.217467
SERINC1		-0.205429848	2.59E-06	8.49E-06
SERINC2		-0.085754224	0.051784	0.078963
SERINC3		0.087754287	0.04654	0.071673
SERINC4		-0.195781398	7.62E-06	2.32E-05
SERINC5		-0.174351297	6.96E-05	0.000182
SERP1		0.08074492	0.067111	0.099696
SERP2		-0.224276788	2.70E-07	1.04E-06
SERPINA10		0.02340908	0.596099	0.662009
SERPINA11		-0.004303154	0.922395	0.939265
SERPINA12		0.045273267	0.305154	0.376926
SERPINA13		0.05609202	0.203786	0.266402
SERPINA1		-0.112087881	0.010911	0.019221
SERPINA3		0.121512022	0.005761	0.010697
SERPINA4		0.080401563	0.068285	0.101252
SERPINA5		0.103855924	0.018398	0.030887
SERPINA6		-0.086111923	0.050811	0.07764
SERPINA7		-0.040283908	0.361592	0.436285
SERPINA9		-0.135467164	0.002063	0.004154
SERPINB10		-0.221177047	3.98E-07	1.49E-06
SERPINB11		-0.060074618	0.173442	0.231673
SERPINB12		0.089477022	0.042388	0.06581
SERPINB13		-0.049718351	0.260062	0.328891
SERPINB1		0.104857228	0.017295	0.029175
SERPINB2		-0.032553116	0.461032	0.534923
SERPINB3		0.1390429	0.001561	0.003205

SERPINB4	0.176384725	5.71E-05	0.000151
SERPINB5	0.334902972	5.80E-15	7.14E-14
SERPINB6	-0.044941892	0.308711	0.380614
SERPINB7	0.180842714	3.66E-05	9.99E-05
SERPINB8	0.046425798	0.292994	0.364247
SERPINB9	-0.056115598	0.203596	0.266205
SERPINC1	-0.124214884	0.004758	0.008979
SERPIND1	-0.210240476	1.48E-06	5.06E-06
SERPINE1	0.161420621	0.000235	0.00056
SERPINE2	0.112808586	0.010407	0.018404
SERPINE3	-0.058958905	0.181581	0.240951
SERPINF1	0.007072772	0.872788	0.900866
SERPINF2	-0.052007472	0.23873	0.305508
SERPING1	-0.200530496	4.51E-06	1.42E-05
SERPINH1	0.100945736	0.021957	0.036292
SERPINI1	0.0959482	0.02947	0.047396
SERPINI2	-0.27463672	2.30E-10	1.41E-09
SERTAD1	-0.003463942	0.937495	0.950782
SERTAD2	-0.112133405	0.010879	0.019169
SERTAD3	0.066532785	0.131587	0.181466
SERTAD4	-0.232197049	9.84E-08	4.06E-07
SESN1	-0.37687949	7.94E-19	1.75E-17
SESN2	-0.207495991	2.04E-06	6.81E-06
SESN3	-0.171910361	8.82E-05	0.000226
SESTD1	-0.170099557	0.000105	0.000266
SETBP1	-0.201045158	4.26E-06	1.35E-05
SETD1A	-0.118631881	0.007035	0.012854
SETD1B	-0.167458569	0.000135	0.000335
SETD2	-0.173733752	7.39E-05	0.000192
SETD3	0.033709855	0.445251	0.519537
SETD4	-0.255600599	3.99E-09	2.01E-08
SETD5	-0.048138368	0.275532	0.345916
SETD6	0.024692256	0.576107	0.643668
SETD7	0.07186677	0.1033	0.146282
SETD8	0.213084098	1.06E-06	3.71E-06
SETDB1	0.004140657	0.925317	0.94176
SETDB2	-0.421853844	1.22E-23	5.53E-22
SETMAR	-0.040909443	0.354178	0.428816
SETX	-0.18758907	1.83E-05	5.26E-05
SET	0.322095579	6.77E-14	7.03E-13
SEZ6L2	-0.217067583	6.58E-07	2.38E-06
SEZ6L	-0.141622488	0.001271	0.002655
SEZ6	-0.068627605	0.119837	0.167013
SF1	-0.096379891	0.028744	0.046325

SF3A1	-0.108195685	0.014026	0.024156
SF3A2	-0.022582333	0.609145	0.673652
SF3A3	0.132387148	0.00261	0.005152
SF3B14	0.356961089	6.38E-17	1.05E-15
SF3B1	-0.100101901	0.023095	0.037996
SF3B2	0.161470355	0.000234	0.000558
SF3B3	0.145493735	0.000928	0.001985
SF3B4	0.182960859	2.95E-05	8.19E-05
SF3B5	0.12795951	0.003628	0.006974
SF4	-0.171770182	8.94E-05	0.000229
SFI1	-0.452767961	2.15E-27	1.65E-25
SFMBT1	0.005032776	0.909289	0.928901
SFMBT2	-0.025049353	0.5706	0.63873
SFN	0.093128108	0.034612	0.054767
SFPQ	0.210697798	1.41E-06	4.82E-06
SFRP1	-0.033067059	0.453982	0.52803
SFRP2	0.168800167	0.000119	0.000298
SFRP4	-0.046804406	0.289071	0.360132
SFRP5	-0.197570008	6.26E-06	1.93E-05
SFRS11	-0.202495233	3.62E-06	1.16E-05
SFRS12IP1	0.087652608	0.046795	0.072027
SFRS12	-0.252834803	5.93E-09	2.93E-08
SFRS13A	0.103807317	0.018453	0.030971
SFRS13B	0.151890377	0.000543	0.001209
SFRS14	-0.228891974	1.51E-07	6.03E-07
SFRS15	-0.154067325	0.00045	0.001018
SFRS16	-0.154116309	0.000448	0.001014
SFRS17A	-0.237109203	5.16E-08	2.22E-07
SFRS18	-0.396781848	7.26E-21	2.16E-19
SFRS1	0.251147353	7.54E-09	3.68E-08
SFRS2B	-0.121734419	0.005672	0.010547
SFRS2IP	0.011314528	0.797834	0.838262
SFRS2	0.073825388	0.094214	0.134789
SFRS3	0.243627541	2.14E-08	9.76E-08
SFRS4	-0.308090832	8.72E-13	7.70E-12
SFRS5	-0.335423443	5.23E-15	6.48E-14
SFRS6	-0.101168764	0.021664	0.035856
SFRS7	0.17833218	4.70E-05	0.000126
SFRS8	-0.087407241	0.047416	0.072854
SFRS9	0.45416069	1.42E-27	1.12E-25
SFT2D1	0.130624094	0.002979	0.005822
SFT2D2	-0.022985569	0.602766	0.667891
SFT2D3	-0.116254395	0.008272	0.014914
SFTA1P	-0.418385767	3.06E-23	1.31E-21

SFTA2	-0.222924922	3.20E-07	1.22E-06
SFTA3	-0.297071676	5.94E-12	4.59E-11
SFTPA1	-0.294858625	8.64E-12	6.53E-11
SFTPA2	-0.296345738	6.72E-12	5.16E-11
SFTPB	-0.286063058	3.72E-11	2.55E-10
SFTPC	-0.33311703	8.22E-15	9.82E-14
SFTPD	-0.372100475	2.34E-18	4.79E-17
SFXN1	0.352069689	1.79E-16	2.74E-15
SFXN2	-0.139921032	0.001456	0.003007
SFXN3	-0.230410106	1.24E-07	5.04E-07
SFXN4	0.347984511	4.17E-16	6.10E-15
SFXN5	-0.018142899	0.681248	0.738357
SGCA	-0.388871327	4.88E-20	1.27E-18
SGCB	0.005776803	0.89595	0.91856
SGCD	-0.214420896	9.04E-07	3.20E-06
SGCE	-0.017594279	0.690381	0.746717
SGCG	-0.316904302	1.77E-13	1.73E-12
SGCZ	0.048930216	0.267702	0.337316
SGEF	-0.238014799	4.58E-08	1.98E-07
SGIP1	-0.163188652	0.0002	0.000483
SGK196	0.093082854	0.0347	0.05489
SGK1	-0.262944938	1.36E-09	7.40E-09
SGK223	-0.173900119	7.28E-05	0.000189
SGK269	-0.314394976	2.81E-13	2.66E-12
SGK2	0.000865345	0.98437	0.9883
SGK3	-0.02332981	0.597345	0.662913
SGK494	-0.208332901	1.85E-06	6.24E-06
SGMS1	0.021048518	0.633676	0.69528
SGMS2	-0.172684532	8.18E-05	0.000211
SGOL1	0.532868733	4.08E-39	2.72E-36
SGOL2	0.459522474	2.88E-28	2.42E-26
SGPL1	0.15126002	0.000573	0.001271
SGPP1	0.23226696	9.75E-08	4.02E-07
SGPP2	-0.186885571	1.97E-05	5.63E-05
SGSH	-0.341645167	1.52E-15	2.04E-14
SGSM1	-0.333201523	8.09E-15	9.67E-14
SGSM2	-0.345144044	7.47E-16	1.05E-14
SGSM3	-0.292796453	1.22E-11	9.02E-11
SGTA	0.116604365	0.008078	0.014603
SGTB	0.064566775	0.143407	0.196022
SH2B1	-0.415606242	6.32E-23	2.57E-21
SH2B2	0.050064243	0.256757	0.325453
SH2B3	-0.207998691	1.93E-06	6.46E-06
SH2D1A	-0.099587602	0.023813	0.039034

SH2D1B	0.069797812	0.113639	0.159465
SH2D2A	0.091786064	0.037316	0.058623
SH2D3A	-0.062039255	0.159775	0.215463
SH2D3C	-0.355583201	8.55E-17	1.38E-15
SH2D4A	-0.077670871	0.078237	0.114346
SH2D4B	-0.144230702	0.001029	0.002185
SH2D5	0.272308329	3.30E-10	1.97E-09
SH2D6	-0.018401129	0.676965	0.734392
SH2D7	-0.04121373	0.350607	0.425172
SH3BGRL2	-0.189535758	1.49E-05	4.35E-05
SH3BGRL3	0.007118796	0.871967	0.900205
SH3BGRL	-0.215211153	8.23E-07	2.92E-06
SH3BGR	-0.236140247	5.87E-08	2.50E-07
SH3BP1	-0.064675477	0.142733	0.195207
SH3BP2	-0.08834261	0.045085	0.069652
SH3BP4	-0.063816922	0.148123	0.201572
SH3BP5L	-0.07020412	0.111547	0.156705
SH3BP5	-0.246481716	1.45E-08	6.76E-08
SH3D19	-0.117885816	0.007404	0.013476
SH3D20	-0.209493548	1.62E-06	5.49E-06
SH3GL1	0.120867094	0.006027	0.011148
SH3GL2	-0.152043542	0.000536	0.001195
SH3GL3	-0.183039045	2.93E-05	8.13E-05
SH3GLB1	0.096565988	0.028435	0.045891
SH3GLB2	-0.212459924	1.14E-06	3.96E-06
SH3KBP1	-0.0086514	0.844721	0.877015
SH3PXD2A	-0.177403872	5.16E-05	0.000137
SH3PXD2B	0.129473194	0.003246	0.006298
SH3RF1	-0.213829423	9.70E-07	3.42E-06
SH3RF2	-0.130932738	0.002911	0.005699
SH3RF3	-0.194674286	8.60E-06	2.60E-05
SH3TC1	-0.270817484	4.16E-10	2.44E-09
SH3TC2	-0.008940299	0.839605	0.8727
SH3YL1	-0.160391147	0.000257	0.00061
SHANK1	0.018954001	0.66783	0.726042
SHANK2	-0.136702709	0.001875	0.003796
SHANK3	-0.285128012	4.33E-11	2.94E-10
SHARPIN	0.046676032	0.290397	0.361536
SHBG	-0.067530966	0.125882	0.174368
SHB	-0.053879885	0.222221	0.287099
SHC1	0.231696973	1.05E-07	4.31E-07
SHC2	-0.271373013	3.82E-10	2.25E-09
SHC3	-0.269828646	4.84E-10	2.81E-09
SHC4	-0.096283876	0.028904	0.046563

SHCBP1	0.454080066	1.46E-27	1.14E-25
SHD	0.069732457	0.113979	0.159829
SHE	-0.258659833	2.56E-09	1.33E-08
SHFM1	0.264644885	1.06E-09	5.87E-09
SHF	-0.172829273	8.07E-05	0.000208
SHH	-0.325693397	3.43E-14	3.75E-13
SHISA2	-0.230060267	1.30E-07	5.25E-07
SHISA3	-0.282830177	6.29E-11	4.16E-10
SHISA4	-0.153566686	0.00047	0.001058
SHISA5	-0.023418589	0.59595	0.661916
SHISA6	-0.215554387	7.89E-07	2.81E-06
SHISA7	-0.097511541	0.026912	0.043663
SHISA9	-0.052452409	0.234731	0.301181
SHKBP1	0.04402527	0.318693	0.391327
SHMT1	-0.020836046	0.637107	0.698316
SHMT2	0.396777963	7.27E-21	2.16E-19
SHOC2	0.127882568	0.003649	0.007011
SHOX2	0.168167143	0.000126	0.000315
SHOX	-0.05114748	0.246595	0.314306
SHPK	0.018926443	0.668284	0.726434
SHPRH	-0.12543812	0.004358	0.008271
SHQ1	0.180466646	3.80E-05	0.000103
SHROOM1	-0.1526808	0.000507	0.001136
SHROOM2	-0.228381576	1.61E-07	6.40E-07
SHROOM3	-0.307152	1.03E-12	9.01E-12
SHROOM4	-0.432404644	7.06E-25	3.86E-23
SIAE	-0.081532564	0.064479	0.096146
SIAH1	0.104950054	0.017196	0.02903
SIAH2	0.143087051	0.00113	0.00238
SIAH3	-0.248058296	1.16E-08	5.50E-08
SIDT1	-0.229036595	1.48E-07	5.93E-07
SIDT2	-0.322922232	5.79E-14	6.06E-13
SIGIRR	-0.247673498	1.23E-08	5.79E-08
SIGLEC10	-0.026853517	0.543166	0.613969
SIGLEC11	-0.223959149	2.81E-07	1.08E-06
SIGLEC12	0.062210491	0.158624	0.214112
SIGLEC14	-0.112222422	0.010816	0.019067
SIGLEC15	-0.074936414	0.08935	0.128614
SIGLEC16	-0.226381025	2.08E-07	8.13E-07
SIGLEC1	-0.258417919	2.66E-09	1.38E-08
SIGLEC5	-0.179340373	4.25E-05	0.000115
SIGLEC6	-0.311718498	4.56E-13	4.19E-12
SIGLEC7	-0.107291673	0.014853	0.025465
SIGLEC8	-0.283079069	6.04E-11	4.01E-10

SIGLEC9	-0.144038879	0.001046	0.002216
SIGLECP3	-0.398770509	4.46E-21	1.37E-19
SIGMAR1	0.323942298	4.78E-14	5.08E-13
SIK1	0.037233852	0.399111	0.473804
SIK2	-0.006898806	0.875891	0.903072
SIK3	-0.142374829	0.001197	0.002509
SIKE1	0.060782843	0.168418	0.225793
SIL1	-0.041894068	0.342705	0.416717
SILV	-0.012042153	0.785141	0.827405
SIM1	0.095092493	0.030956	0.049531
SIM2	0.014389633	0.744594	0.793522
SIN3A	-0.063386076	0.150886	0.204927
SIN3B	-0.222509888	3.37E-07	1.28E-06
SIP1	0.335200594	5.47E-15	6.75E-14
SIPA1L1	0.016653399	0.706149	0.761054
SIPA1L2	-0.196824619	6.80E-06	2.09E-05
SIPA1L3	-0.157861218	0.000323	0.000749
SIPA1	-0.147395836	0.000793	0.001718
SIRPA	-0.135430332	0.002069	0.004165
SIRPB1	-0.151013324	0.000585	0.001296
SIRPB2	-0.209255927	1.66E-06	5.64E-06
SIRPD	-0.063638832	0.149261	0.20294
SIRPG	-0.052797423	0.231663	0.297779
SIRT1	0.033764334	0.444515	0.51877
SIRT2	-0.259066315	2.42E-09	1.26E-08
SIRT3	-0.241597507	2.83E-08	1.26E-07
SIRT4	-0.077163297	0.08021	0.116948
SIRT5	-0.022043617	0.617713	0.68113
SIRT6	0.006721602	0.879054	0.905371
SIRT7	0.015224914	0.730328	0.781509
SIT1	-0.126206366	0.004123	0.007856
SIVA1	0.063739694	0.148616	0.202146
SIX1	-0.069497313	0.115206	0.161369
SIX2	-0.037199807	0.399543	0.474232
SIX3	-0.031460468	0.476221	0.549928
SIX4	0.08352301	0.058206	0.087653
SIX5	-0.089737187	0.041788	0.065032
SIX6	-0.012546167	0.776382	0.820034
SI	0.026762631	0.544532	0.6152
SKA1	0.50743407	4.72E-35	1.33E-32
SKA2	0.240084089	3.47E-08	1.53E-07
SKA3	0.558187014	1.62E-43	2.15E-40
SKAP1	-0.051764877	0.240931	0.308031
SKAP2	0.128880979	0.003391	0.006556

SKIL	0.14168401	0.001265	0.002644
SKINTL	-0.291023936	1.64E-11	1.19E-10
SKIV2L2	0.113448489	0.009977	0.017722
SKIV2L	-0.091508637	0.037896	0.05948
SKI	-0.316978933	1.75E-13	1.71E-12
SKP1	0.123236439	0.005101	0.009572
SKP2	0.280587553	9.01E-11	5.84E-10
SLA2	-0.054506635	0.216881	0.28122
SLAIN1	-0.201096095	4.23E-06	1.34E-05
SLAIN2	0.002336666	0.957813	0.967689
SLAMF1	-0.159214566	0.000286	0.000672
SLAMF6	-0.118027398	0.007333	0.013361
SLAMF7	-0.005593157	0.89924	0.921319
SLAMF8	-0.06854343	0.120293	0.167613
SLAMF9	0.25607881	3.73E-09	1.89E-08
SLA	-0.18098883	3.60E-05	9.86E-05
SLBP	0.339323015	2.42E-15	3.16E-14
SLC10A1	-0.078944716	0.073457	0.108047
SLC10A2	-0.215024648	8.41E-07	2.99E-06
SLC10A3	0.004729872	0.914727	0.933311
SLC10A4	0.04888579	0.268138	0.337791
SLC10A5	-0.059826207	0.17523	0.233781
SLC10A6	-0.004009931	0.927668	0.943385
SLC10A7	-0.075691784	0.08616	0.124507
SLC11A1	-0.13164735	0.002759	0.005424
SLC11A2	-0.128079155	0.003597	0.006919
SLC12A1	-0.081140642	0.065778	0.097942
SLC12A2	-0.053713732	0.223652	0.288745
SLC12A3	-0.099305703	0.024215	0.039637
SLC12A4	-0.415514932	6.47E-23	2.62E-21
SLC12A5	-0.179800974	4.06E-05	0.00011
SLC12A6	-0.243162931	2.28E-08	1.04E-07
SLC12A7	-0.03243159	0.462708	0.53668
SLC12A8	0.340721312	1.83E-15	2.43E-14
SLC12A9	-0.262680564	1.42E-09	7.68E-09
SLC13A1	-0.030822598	0.485213	0.558859
SLC13A2	-0.167949109	0.000128	0.000321
SLC13A3	-0.010526952	0.811632	0.849589
SLC13A4	-0.174057396	7.16E-05	0.000186
SLC13A5	0.101281643	0.021518	0.035639
SLC14A1	-0.291449527	1.53E-11	1.11E-10
SLC14A2	0.028518814	0.518439	0.590627
SLC15A1	0.099030436	0.024613	0.040233
SLC15A2	-0.421448804	1.36E-23	6.13E-22

SLC15A3	-0.295211061	8.14E-12	6.18E-11
SLC15A4	0.21186283	1.23E-06	4.24E-06
SLC16A10	-0.000479579	0.991338	0.993574
SLC16A11	-0.386747031	8.06E-20	2.04E-18
SLC16A12	-0.22557087	2.30E-07	8.93E-07
SLC16A13	0.056191538	0.202984	0.265578
SLC16A14	0.267294023	7.11E-10	4.04E-09
SLC16A1	0.266194684	8.39E-10	4.72E-09
SLC16A2	-0.133567936	0.002386	0.004747
SLC16A3	0.16065161	0.000252	0.000597
SLC16A4	-0.037459222	0.396261	0.471148
SLC16A5	-0.238260965	4.43E-08	1.92E-07
SLC16A6	-0.095650223	0.02998	0.048162
SLC16A7	-0.257358106	3.10E-09	1.59E-08
SLC16A8	-0.084862697	0.054275	0.082423
SLC16A9	-0.047859542	0.278326	0.348854
SLC17A1	-0.024223701	0.583371	0.650257
SLC17A2	0.053478688	0.225687	0.290941
SLC17A3	-0.106470899	0.01564	0.026644
SLC17A4	0.053812937	0.222796	0.287752
SLC17A5	0.005995276	0.892039	0.915588
SLC17A6	0.015355694	0.728103	0.77967
SLC17A7	-0.05674836	0.198537	0.260339
SLC17A8	-0.173661016	7.45E-05	0.000193
SLC17A9	-0.049910173	0.258226	0.326982
SLC18A1	-0.206990704	2.17E-06	7.19E-06
SLC18A2	-0.37025871	3.53E-18	7.04E-17
SLC18A3	-0.089540268	0.042241	0.065634
SLC19A1	0.037509722	0.395624	0.470475
SLC19A2	0.081106095	0.065893	0.098092
SLC19A3	-0.144137525	0.001037	0.0022
SLC1A1	-0.204072308	3.03E-06	9.80E-06
SLC1A2	-0.233206643	8.63E-08	3.59E-07
SLC1A3	-0.150230731	0.000625	0.001379
SLC1A4	-0.067171568	0.127914	0.176912
SLC1A5	0.157186902	0.000343	0.000791
SLC1A6	0.14651508	0.000853	0.001836
SLC1A7	-0.298997722	4.27E-12	3.36E-11
SLC20A1	0.210927505	1.37E-06	4.70E-06
SLC20A2	-0.141581776	0.001275	0.002663
SLC22A10	-0.135064607	0.002128	0.004275
SLC22A11	-0.047361261	0.283366	0.35415
SLC22A12	-2.04E-06	0.999963	0.999963
SLC22A13	-0.003447988	0.937782	0.950913

SLC22A14	-0.056062937	0.204021	0.266648
SLC22A15	-0.334092889	6.79E-15	8.26E-14
SLC22A16	-0.130861324	0.002927	0.005727
SLC22A17	-0.34108276	1.70E-15	2.28E-14
SLC22A18AS	0.01737117	0.694108	0.750058
SLC22A18	-0.179728791	4.09E-05	0.000111
SLC22A1	-0.093487754	0.033916	0.053769
SLC22A20	-0.187113063	1.92E-05	5.51E-05
SLC22A23	-0.198648353	5.56E-06	1.73E-05
SLC22A24	-0.050102061	0.256398	0.325017
SLC22A25	-0.008193654	0.852841	0.883701
SLC22A2	0.012491459	0.777331	0.82082
SLC22A3	-0.334095207	6.79E-15	8.26E-14
SLC22A4	-0.135337492	0.002084	0.004193
SLC22A5	-0.203831823	3.11E-06	1.01E-05
SLC22A6	-0.039123317	0.375601	0.450441
SLC22A7	0.001680296	0.969656	0.977185
SLC22A8	-0.051637334	0.242093	0.309317
SLC22A9	0.049665257	0.260572	0.329411
SLC23A1	-0.10603985	0.016068	0.027294
SLC23A2	-0.065689345	0.136563	0.187641
SLC23A3	-0.20769412	2.00E-06	6.67E-06
SLC24A1	-0.189026885	1.57E-05	4.58E-05
SLC24A2	0.114483091	0.009315	0.016628
SLC24A3	-0.1563252	0.00037	0.000848
SLC24A4	-0.306665474	1.12E-12	9.76E-12
SLC24A5	-0.187561099	1.83E-05	5.28E-05
SLC24A6	-0.028409118	0.52005	0.592192
SLC25A10	0.218109719	5.79E-07	2.12E-06
SLC25A11	0.094017783	0.032913	0.052323
SLC25A12	-0.035544711	0.420859	0.495472
SLC25A13	0.281935762	7.26E-11	4.77E-10
SLC25A14	-0.130103331	0.003097	0.006033
SLC25A15	0.376644304	8.38E-19	1.84E-17
SLC25A16	-0.059081421	0.180674	0.239938
SLC25A17	0.130103477	0.003097	0.006033
SLC25A18	-0.051491831	0.243424	0.310819
SLC25A19	0.046670376	0.290456	0.361587
SLC25A1	0.045267442	0.305216	0.376957
SLC25A20	-0.039427497	0.371897	0.446777
SLC25A21	0.317451412	1.60E-13	1.58E-12
SLC25A22	0.052025368	0.238568	0.305341
SLC25A23	-0.315997369	2.09E-13	2.03E-12
SLC25A24	0.148645864	0.000715	0.001561

SLC25A25	-0.15037296	0.000618	0.001364
SLC25A26	-0.011758581	0.790081	0.831517
SLC25A27	-0.398786154	4.44E-21	1.36E-19
SLC25A28	-0.198030506	5.95E-06	1.85E-05
SLC25A29	-0.38354467	1.71E-19	4.17E-18
SLC25A2	-0.121848438	0.005627	0.010473
SLC25A30	-0.213893746	9.63E-07	3.39E-06
SLC25A31	-0.056290937	0.202185	0.264706
SLC25A32	0.432096534	7.69E-25	4.16E-23
SLC25A33	0.13966663	0.001486	0.003064
SLC25A34	-0.356047173	7.75E-17	1.26E-15
SLC25A35	-0.143198631	0.00112	0.002361
SLC25A36	-0.187057994	1.93E-05	5.54E-05
SLC25A37	-0.011398397	0.796368	0.836942
SLC25A38	-0.095530325	0.030188	0.048459
SLC25A39	0.216803391	6.79E-07	2.45E-06
SLC25A3	0.370766653	3.15E-18	6.33E-17
SLC25A40	0.180754855	3.69E-05	0.000101
SLC25A41	-0.201484659	4.05E-06	1.29E-05
SLC25A42	-0.403466377	1.39E-21	4.68E-20
SLC25A43	0.15769209	0.000328	0.000759
SLC25A44	-0.029358431	0.506196	0.578958
SLC25A45	-0.265391151	9.47E-10	5.29E-09
SLC25A46	0.002380007	0.957031	0.967144
SLC25A4	-0.227896444	1.71E-07	6.79E-07
SLC25A5	0.244041832	2.03E-08	9.26E-08
SLC25A6	0.074376634	0.091775	0.131668
SLC26A10	-0.045197123	0.305969	0.377699
SLC26A11	-0.303508441	1.96E-12	1.63E-11
SLC26A1	-0.113532385	0.009922	0.017638
SLC26A2	0.066310197	0.132887	0.183182
SLC26A3	-0.010831399	0.806291	0.845026
SLC26A4	0.056786041	0.198238	0.259982
SLC26A5	-0.397997187	5.39E-21	1.63E-19
SLC26A6	-0.075802833	0.085699	0.123948
SLC26A7	-0.071639992	0.104396	0.147665
SLC26A8	-0.240254765	3.39E-08	1.50E-07
SLC26A9	-0.321263013	7.91E-14	8.12E-13
SLC27A1	-0.519829264	5.46E-37	2.26E-34
SLC27A2	0.122359645	0.005428	0.010132
SLC27A3	-0.355041671	9.58E-17	1.52E-15
SLC27A4	0.268196405	6.20E-10	3.56E-09
SLC27A5	0.112735247	0.010458	0.018486
SLC27A6	-0.140582795	0.001382	0.002865

SLC28A1	0.034260685	0.437845	0.512092
SLC28A2	0.067652341	0.125201	0.173509
SLC28A3	-0.159043236	0.000291	0.000681
SLC29A1	-0.200037822	4.77E-06	1.50E-05
SLC29A2	0.04556996	0.301992	0.373667
SLC29A3	-0.151455456	0.000563	0.001252
SLC29A4	0.006762963	0.878316	0.90489
SLC2A10	-0.030826767	0.485154	0.558856
SLC2A11	-0.276605359	1.69E-10	1.05E-09
SLC2A12	-0.203481481	3.24E-06	1.04E-05
SLC2A13	-0.114480676	0.009316	0.016629
SLC2A14	0.073242426	0.096849	0.138213
SLC2A1	0.360430415	3.04E-17	5.29E-16
SLC2A2	0.088377473	0.045	0.069526
SLC2A3	0.065568911	0.137285	0.188542
SLC2A4RG	-0.002410998	0.956472	0.966774
SLC2A4	-0.233070538	8.78E-08	3.65E-07
SLC2A5	0.123238815	0.0051	0.009572
SLC2A6	-0.049210071	0.264972	0.334276
SLC2A7	0.193531431	9.73E-06	2.92E-05
SLC2A8	-0.116077523	0.008371	0.015074
SLC2A9	-0.210471173	1.44E-06	4.93E-06
SLC30A10	0.23709576	5.17E-08	2.22E-07
SLC30A1	0.105309154	0.016817	0.028452
SLC30A2	-0.150922525	0.00059	0.001305
SLC30A3	-0.170791951	9.81E-05	0.00025
SLC30A4	-0.057280428	0.194354	0.255525
SLC30A5	0.121796671	0.005647	0.010507
SLC30A6	0.269130618	5.38E-10	3.11E-09
SLC30A7	0.139031521	0.001562	0.003207
SLC30A8	-0.071345229	0.105833	0.149459
SLC30A9	0.107963331	0.014234	0.024483
SLC31A1	0.070057313	0.1123	0.157718
SLC31A2	-0.110824429	0.011848	0.020678
SLC32A1	-0.019983422	0.650953	0.710841
SLC33A1	0.274175078	2.47E-10	1.50E-09
SLC34A1	0.071813495	0.103557	0.146624
SLC34A2	-0.393950341	1.44E-20	4.06E-19
SLC34A3	-0.03148783	0.475838	0.549517
SLC35A1	-0.081892629	0.063305	0.094595
SLC35A2	0.235896324	6.06E-08	2.57E-07
SLC35A3	0.100709669	0.02227	0.036761
SLC35A4	-0.206237628	2.36E-06	7.80E-06
SLC35A5	-0.064448793	0.144142	0.196929

SLC35B1	0.199299562	5.17E-06	1.62E-05
SLC35B2	0.213390694	1.02E-06	3.59E-06
SLC35B3	0.053096771	0.229023	0.294823
SLC35B4	0.101934111	0.020686	0.034376
SLC35C1	0.065809211	0.135847	0.186812
SLC35C2	0.029400262	0.50559	0.578362
SLC35D1	0.004899619	0.911679	0.930914
SLC35D2	0.077292745	0.079703	0.116285
SLC35D3	0.05682476	0.197932	0.259665
SLC35E1	-0.136541585	0.001899	0.003841
SLC35E2	-0.330195139	1.45E-14	1.67E-13
SLC35E3	0.235154393	6.69E-08	2.82E-07
SLC35E4	0.066669151	0.130796	0.180525
SLC35F1	-0.004097289	0.926097	0.942266
SLC35F2	0.202298301	3.70E-06	1.18E-05
SLC35F3	-0.073904676	0.09386	0.134341
SLC35F4	0.192691	1.06E-05	3.17E-05
SLC35F5	0.177022052	5.36E-05	0.000142
SLC36A1	-0.109463177	0.012935	0.022424
SLC36A2	0.015935512	0.718265	0.770994
SLC36A3	-0.075357965	0.087559	0.126363
SLC36A4	0.261940705	1.58E-09	8.51E-09
SLC37A1	-0.125688163	0.00428	0.008131
SLC37A2	-0.271516139	3.73E-10	2.21E-09
SLC37A3	0.203512611	3.22E-06	1.04E-05
SLC37A4	0.013898783	0.753018	0.80073
SLC38A10	-0.247509833	1.26E-08	5.91E-08
SLC38A11	-0.003983747	0.928139	0.94372
SLC38A1	0.178009468	4.86E-05	0.00013
SLC38A2	0.299999623	3.59E-12	2.86E-11
SLC38A3	-0.067677832	0.125059	0.173348
SLC38A4	0.082796663	0.060434	0.090755
SLC38A5	-0.005488602	0.901114	0.922671
SLC38A6	0.022795284	0.605772	0.67059
SLC38A7	0.168753249	0.000119	0.000299
SLC38A8	0.174693936	6.73E-05	0.000176
SLC38A9	0.176881277	5.43E-05	0.000144
SLC39A10	0.134333388	0.002251	0.004496
SLC39A11	0.085899839	0.051386	0.078421
SLC39A12	-0.086102734	0.050836	0.077666
SLC39A13	-0.265002607	1.00E-09	5.58E-09
SLC39A14	0.346697581	5.44E-16	7.85E-15
SLC39A1	0.210684308	1.41E-06	4.82E-06
SLC39A2	-0.071047372	0.107302	0.151351

SLC39A3	0.027028627	0.540539	0.61131
SLC39A4	0.1044426	0.017744	0.029888
SLC39A5	0.144767014	0.000985	0.002098
SLC39A6	0.188983972	1.58E-05	4.59E-05
SLC39A7	0.106262778	0.015845	0.026954
SLC39A8	-0.250785106	7.93E-09	3.85E-08
SLC39A9	0.248975645	1.02E-08	4.90E-08
SLC3A1	-0.023938385	0.587814	0.654275
SLC3A2	0.272768747	3.08E-10	1.84E-09
SLC40A1	-0.24569673	1.61E-08	7.48E-08
SLC41A1	-0.433892527	4.69E-25	2.65E-23
SLC41A2	0.169703579	0.000109	0.000276
SLC41A3	-0.123701744	0.004935	0.00928
SLC43A1	-0.102017855	0.020582	0.034217
SLC43A2	-0.193666415	9.59E-06	2.88E-05
SLC43A3	-0.115259237	0.008844	0.015846
SLC44A1	0.316179923	2.02E-13	1.97E-12
SLC44A2	-0.32229814	6.51E-14	6.79E-13
SLC44A3	-0.14258362	0.001177	0.002472
SLC44A4	-0.264978434	1.01E-09	5.60E-09
SLC44A5	0.071699016	0.10411	0.147303
SLC45A1	0.023617557	0.59283	0.658744
SLC45A2	-0.030953099	0.483366	0.557085
SLC45A3	-0.067612891	0.125422	0.173767
SLC45A4	0.084128881	0.0564	0.085242
SLC46A1	-0.268060937	6.33E-10	3.63E-09
SLC46A2	-0.327916752	2.25E-14	2.51E-13
SLC46A3	-0.253299643	5.55E-09	2.75E-08
SLC47A1	-0.3508515	2.31E-16	3.47E-15
SLC47A2	-0.024196429	0.583795	0.650585
SLC48A1	-0.126641808	0.003995	0.007629
SLC4A10	0.006109633	0.889992	0.913858
SLC4A11	-0.052671629	0.232778	0.298983
SLC4A1AP	0.152435806	0.000518	0.001158
SLC4A1	-0.090900286	0.039197	0.061304
SLC4A2	-0.024011428	0.586675	0.653139
SLC4A3	-0.067264329	0.127387	0.176257
SLC4A4	-0.266136306	8.47E-10	4.76E-09
SLC4A5	-0.117508418	0.007597	0.013799
SLC4A7	0.01754725	0.691166	0.747404
SLC4A8	-0.157582259	0.000331	0.000766
SLC4A9	-0.102648464	0.019808	0.033041
SLC5A10	0.156657036	0.000359	0.000826
SLC5A11	0.074158079	0.092736	0.132836

SLC5A12	0.172325956	8.47E-05	0.000218
SLC5A1	-0.186899999	1.97E-05	5.63E-05
SLC5A2	-0.305244195	1.44E-12	1.23E-11
SLC5A3	-0.006322579	0.886184	0.910602
SLC5A4	-0.287849	2.78E-11	1.94E-10
SLC5A5	0.05610725	0.203663	0.266258
SLC5A6	0.261337523	1.73E-09	9.23E-09
SLC5A7	-0.237676646	4.79E-08	2.06E-07
SLC5A8	-0.09519577	0.030773	0.049259
SLC5A9	-0.29704626	5.96E-12	4.61E-11
SLC6A10P	0.213178818	1.05E-06	3.67E-06
SLC6A11	-0.009196864	0.835067	0.86882
SLC6A12	-0.145413601	0.000934	0.001997
SLC6A13	-0.16936671	0.000112	0.000284
SLC6A14	-0.061049885	0.166553	0.223456
SLC6A15	0.155752884	0.000389	0.000888
SLC6A16	-0.323369976	5.33E-14	5.61E-13
SLC6A17	0.213625518	9.94E-07	3.49E-06
SLC6A18	-0.158562305	0.000303	0.000708
SLC6A19	0.039146405	0.375319	0.450157
SLC6A1	-0.182462331	3.10E-05	8.58E-05
SLC6A20	-0.27996027	9.95E-11	6.42E-10
SLC6A2	-0.073477624	0.095779	0.136823
SLC6A3	-0.123761593	0.004914	0.009243
SLC6A4	-0.259317677	2.33E-09	1.22E-08
SLC6A5	0.014911978	0.735662	0.785979
SLC6A6	-0.073014445	0.097895	0.139597
SLC6A7	-0.211084212	1.34E-06	4.62E-06
SLC6A8	0.210727632	1.40E-06	4.80E-06
SLC6A9	-0.138487184	0.001631	0.003337
SLC7A10	-0.187448503	1.86E-05	5.33E-05
SLC7A11	0.284266639	4.98E-11	3.34E-10
SLC7A13	0.047987832	0.277038	0.347567
SLC7A14	-0.013800179	0.754714	0.801979
SLC7A1	0.315868386	2.14E-13	2.07E-12
SLC7A2	0.129899535	0.003144	0.006117
SLC7A3	-0.059119867	0.18039	0.239625
SLC7A4	-0.243806011	2.09E-08	9.54E-08
SLC7A5P1	0.093401412	0.034082	0.054007
SLC7A5P2	-0.055736701	0.20667	0.269677
SLC7A5	0.356569721	6.93E-17	1.14E-15
SLC7A6OS	0.051703555	0.241489	0.308684
SLC7A6	-0.270531003	4.34E-10	2.55E-09
SLC7A7	-0.052057999	0.238274	0.305002

SLC7A8	-0.243281894	2.25E-08	1.02E-07
SLC7A9	0.06919521	0.116799	0.163302
SLC8A1	-0.172450014	8.37E-05	0.000215
SLC8A2	-0.060869721	0.16781	0.225037
SLC8A3	-0.32772423	2.33E-14	2.60E-13
SLC9A10	-0.114872278	0.009076	0.016227
SLC9A11	-0.303951411	1.81E-12	1.52E-11
SLC9A1	-0.297167306	5.84E-12	4.52E-11
SLC9A2	-0.059105335	0.180497	0.239736
SLC9A3R1	-0.069740478	0.113937	0.159782
SLC9A3R2	-0.204148751	3.00E-06	9.72E-06
SLC9A3	-0.185011196	2.39E-05	6.73E-05
SLC9A4	-0.127318316	0.003803	0.007285
SLC9A5	-0.30549522	1.38E-12	1.18E-11
SLC9A6	-0.137764257	0.001726	0.003518
SLC9A7	0.095786107	0.029746	0.047818
SLC9A8	-0.21137258	1.30E-06	4.47E-06
SLC9A9	-0.254624946	4.60E-09	2.30E-08
SLCO1A2	0.067200587	0.127749	0.176709
SLCO1B1	0.136235147	0.001944	0.003928
SLCO1B3	0.087469639	0.047258	0.072649
SLCO1C1	-0.088961212	0.043597	0.067515
SLCO2A1	-0.225869809	2.21E-07	8.62E-07
SLCO2B1	-0.221790139	3.69E-07	1.39E-06
SLCO3A1	-0.366197466	8.67E-18	1.64E-16
SLCO4A1	0.120569515	0.006153	0.01136
SLCO4C1	-0.26882273	5.64E-10	3.25E-09
SLCO5A1	0.107392641	0.014758	0.02531
SLCO6A1	0.100717006	0.02226	0.036751
SLED1	-0.160762179	0.000249	0.000591
SLFN11	-0.070859913	0.108234	0.152577
SLFN12L	-0.180024294	3.97E-05	0.000108
SLFN12	-0.26869359	5.75E-10	3.31E-09
SLFN13	-0.160560194	0.000254	0.000601
SLFN14	-0.321148199	8.08E-14	8.27E-13
SLFN5	-0.1251702	0.004443	0.008418
SLFNL1	-0.424615027	5.86E-24	2.79E-22
SLIT1	-0.161431661	0.000234	0.00056
SLIT2	-0.228473085	1.59E-07	6.33E-07
SLIT3	-0.324551144	4.26E-14	4.58E-13
SLITRK1	0.088996584	0.043513	0.067403
SLITRK2	-0.13557507	0.002046	0.004122
SLITRK3	-0.250681715	8.05E-09	3.90E-08
SLITRK4	-0.014736016	0.738667	0.788574

SLITRK5	-0.052569365	0.233688	0.300054
SLITRK6	-0.094313097	0.032365	0.051542
SLK	0.102361555	0.020157	0.033566
SLMAP	0.103552292	0.018744	0.031423
SLMO1	0.06182842	0.161202	0.217108
SLMO2	0.389880439	3.84E-20	1.02E-18
SLN	-0.088699263	0.044222	0.06843
SLPI	-0.24985769	9.04E-09	4.36E-08
SLTM	-0.164760623	0.000173	0.000423
SLU7	-0.17656049	5.61E-05	0.000148
SLURP1	0.03999222	0.365081	0.439726
SMAD1	0.046390178	0.293365	0.36464
SMAD2	0.100636331	0.022368	0.036902
SMAD3	-0.049268892	0.264401	0.33366
SMAD4	-0.010336124	0.814985	0.852563
SMAD5OS	-0.098845979	0.024883	0.040637
SMAD5	0.037815894	0.391776	0.466676
SMAD6	-0.369424419	4.25E-18	8.43E-17
SMAD7	-0.268073368	6.32E-10	3.62E-09
SMAD9	-0.171884344	8.84E-05	0.000227
SMAGP	0.210144909	1.50E-06	5.11E-06
SMAP1	0.146314741	0.000868	0.001864
SMAP2	-0.279786987	1.02E-10	6.58E-10
SMARCA1	0.08734241	0.047582	0.07308
SMARCA2	-0.424089292	6.74E-24	3.17E-22
SMARCA4	-0.180859448	3.65E-05	9.98E-05
SMARCA5	0.04756209	0.281327	0.351998
SMARCAD1	0.158073706	0.000317	0.000736
SMARCAL1	0.210536588	1.43E-06	4.90E-06
SMARCB1	0.175377202	6.30E-05	0.000165
SMARCC1	0.168095857	0.000127	0.000317
SMARCC2	-0.093492831	0.033906	0.053762
SMARCD1	0.12963262	0.003207	0.00623
SMARCD2	0.031943644	0.469471	0.543232
SMARCD3	-0.356595878	6.90E-17	1.13E-15
SMARCE1	0.142063198	0.001227	0.002569
SMC1A	0.075748061	0.085927	0.124214
SMC1B	0.086247284	0.050447	0.077152
SMC2	0.343249594	1.10E-15	1.51E-14
SMC3	0.218300209	5.66E-07	2.07E-06
SMC4	0.178744717	4.52E-05	0.000121
SMC5	-0.061980365	0.160173	0.21594
SMC6	0.128839136	0.003401	0.006575
SMCHD1	0.031432763	0.47661	0.550314

SMCP	0.005514982	0.900641	0.922376
SMCR5	-0.253476899	5.42E-09	2.69E-08
SMCR7L	0.144318945	0.001022	0.002172
SMCR7	-0.254882094	4.43E-09	2.22E-08
SMCR8	-0.109529172	0.01288	0.022331
SMEK1	0.092298347	0.036263	0.057159
SMEK2	0.221452025	3.84E-07	1.44E-06
SMEK3P	0.102958486	0.019437	0.032471
SMG1	-0.158567355	0.000303	0.000707
SMG5	0.081111672	0.065875	0.098071
SMG6	-0.260247019	2.03E-09	1.07E-08
SMG7	0.045725345	0.300345	0.371836
SMN1	0.045648321	0.301161	0.372754
SMN2	0.238600582	4.23E-08	1.84E-07
SMNDC1	0.439469545	9.88E-26	6.19E-24
SMOC1	0.127135817	0.003854	0.007375
SMOC2	-0.148847329	0.000703	0.001538
SMOX	0.29832546	4.79E-12	3.74E-11
SMO	-0.007877762	0.858453	0.888549
SMPD1	-0.264262648	1.12E-09	6.18E-09
SMPD2	-0.111458532	0.011369	0.019953
SMPD3	-0.232133414	9.92E-08	4.09E-07
SMPD4	0.087631904	0.046847	0.072085
SMPDL3A	-0.056602165	0.199697	0.261689
SMPDL3B	-0.0428055	0.332298	0.405964
SMPX	-0.154320142	0.00044	0.000998
SMR3A	-0.111822451	0.011102	0.019516
SMR3B	-0.082542984	0.061228	0.091859
SMS	0.352875904	1.51E-16	2.34E-15
SMTNL1	-0.036087142	0.413801	0.488428
SMTNL2	-0.193625221	9.63E-06	2.89E-05
SMTN	0.082052661	0.062789	0.093918
SMU1	0.197706979	6.17E-06	1.91E-05
SMUG1	0.281206081	8.16E-11	5.33E-10
SMURF1	0.025502205	0.563653	0.632795
SMURF2	-0.054855076	0.213952	0.277926
SMYD1	0.036845391	0.404052	0.478759
SMYD2	0.14509849	0.000959	0.002046
SMYD3	0.065314657	0.138819	0.19044
SMYD4	-0.299333635	4.03E-12	3.18E-11
SMYD5	0.135593122	0.002043	0.004117
SNAI1	0.196675086	6.91E-06	2.12E-05
SNAI2	0.186783984	1.99E-05	5.69E-05
SNAI3	-0.33794455	3.18E-15	4.09E-14

SNAP23	0.049725489	0.259994	0.328859
SNAP25	-0.024553821	0.578249	0.645592
SNAP29	-0.021745892	0.622471	0.685469
SNAP47	0.082686184	0.060779	0.091232
SNAP91	-0.085572883	0.052283	0.079633
SNAPC1	0.329781247	1.57E-14	1.81E-13
SNAPC2	-0.061764544	0.161636	0.217619
SNAPC3	0.142477945	0.001187	0.002491
SNAPC4	-0.166777667	0.000143	0.000356
SNAPC5	0.162792853	0.000207	0.0005
SNAPIN	0.111592123	0.011271	0.019794
SNAR-B2	-0.014507502	0.742575	0.791732
SNAR-G1	0.036447598	0.409149	0.483866
SNCAIP	-0.097484779	0.026954	0.043727
SNCA	-0.216046792	7.44E-07	2.66E-06
SNCB	0.113475303	0.009959	0.017695
SNCG	0.191280902	1.24E-05	3.66E-05
SND1	0.244446043	1.92E-08	8.80E-08
SNED1	-0.405658818	8.05E-22	2.77E-20
SNF8	0.30255527	2.31E-12	1.90E-11
SNHG10	-0.045021148	0.307858	0.37975
SNHG11	-0.026034292	0.555542	0.625059
SNHG12	-0.23744253	4.94E-08	2.13E-07
SNHG1	0.141671149	0.001266	0.002646
SNHG3-RCC1	0.101690427	0.020993	0.034846
SNHG3	-0.050626517	0.251447	0.319554
SNHG4	0.153192974	0.000485	0.001091
SNHG5	0.018446936	0.676206	0.733728
SNHG6	0.301030027	3.01E-12	2.42E-11
SNHG7	-0.019802775	0.653902	0.713594
SNHG8	-0.004244049	0.923458	0.940203
SNHG9	-0.158287557	0.000311	0.000724
SNIP1	-0.041933894	0.342246	0.416386
SNN	-0.189715106	1.46E-05	4.28E-05
SNORA10	0.034715825	0.43178	0.50645
SNORA11D	-0.041910215	0.342519	0.416584
SNORA11E	-0.042621666	0.334381	0.408134
SNORA11	-0.018312785	0.678429	0.73578
SNORA12	0.073484537	0.095747	0.136797
SNORA13	-0.048473166	0.272203	0.34221
SNORA14A	0.052675304	0.232745	0.29896
SNORA14B	-0.008378076	0.849568	0.881158
SNORA15	0.010081723	0.819459	0.855989
SNORA16A	-0.036498059	0.4085	0.483171

SNORA16B	-0.023783131	0.590239	0.656339
SNORA18	0.099142479	0.02445	0.039989
SNORA1	-0.016529128	0.708241	0.762609
SNORA20	0.074892024	0.089541	0.128851
SNORA21	-0.07017036	0.11172	0.156926
SNORA22	0.001411955	0.9745	0.980831
SNORA23	0.061820323	0.161257	0.217167
SNORA24	-0.046961374	0.287455	0.358453
SNORA25	-0.049898148	0.258341	0.327086
SNORA26	0.014027011	0.750814	0.798854
SNORA27	0.028345707	0.520982	0.593051
SNORA28	0.041011023	0.352984	0.427602
SNORA29	-0.038047841	0.388876	0.463719
SNORA2A	0.048983441	0.267182	0.336775
SNORA2B	0.014615777	0.740723	0.790136
SNORA31	-0.017847187	0.686165	0.7428
SNORA32	0.06092461	0.167426	0.224568
SNORA34	0.047053668	0.286508	0.357504
SNORA36A	-0.059983439	0.174097	0.232424
SNORA37	-0.07288643	0.098487	0.14034
SNORA38B	0.072223461	0.101596	0.144236
SNORA38	-0.024239144	0.583131	0.650026
SNORA39	-0.226174926	2.13E-07	8.32E-07
SNORA3	0.041558546	0.346588	0.420977
SNORA40	0.094629419	0.031787	0.05073
SNORA41	0.055940343	0.205014	0.267814
SNORA42	0.017467534	0.692497	0.748514
SNORA44	-0.018813296	0.67015	0.728106
SNORA45	-0.0640764	0.146478	0.199605
SNORA46	-0.043322366	0.326488	0.39977
SNORA47	-0.002028228	0.963377	0.971788
SNORA48	0.003838208	0.930758	0.945901
SNORA49	0.016601898	0.707015	0.761577
SNORA4	-0.023108874	0.600822	0.666032
SNORA50	-0.048164674	0.275269	0.34563
SNORA51	0.07054787	0.109801	0.154513
SNORA52	-0.005461047	0.901608	0.923082
SNORA53	0.090444222	0.040196	0.06275
SNORA54	-0.026645387	0.546297	0.616832
SNORA55	-0.051024774	0.247732	0.315554
SNORA56	4.21E-05	0.99924	0.99959
SNORA57	-0.037913245	0.390558	0.465308
SNORA58	0.008635799	0.844998	0.877256
SNORA59B	-0.015907951	0.718732	0.771442

SNORA5A	0.050335094	0.25419	0.322669
SNORA5B	0.014336987	0.745496	0.794338
SNORA5C	0.021672948	0.623639	0.686528
SNORA61	0.037392543	0.397103	0.471981
SNORA62	-0.052606692	0.233355	0.299666
SNORA63	-0.03098406	0.482929	0.556645
SNORA64	-0.008223962	0.852303	0.883372
SNORA65	0.042192218	0.339278	0.413397
SNORA66	-0.009287155	0.833471	0.867633
SNORA67	-0.031506399	0.475577	0.54925
SNORA68	-0.001329929	0.975981	0.982173
SNORA6	0.076380419	0.083333	0.120884
SNORA70B	-0.049064272	0.266392	0.335876
SNORA70	-0.023935535	0.587859	0.654275
SNORA71A	0.013297326	0.763381	0.809639
SNORA71B	0.029686599	0.501452	0.574385
SNORA71C	0.010718882	0.808264	0.846729
SNORA71D	0.081947419	0.063128	0.094382
SNORA72	-0.076965871	0.080989	0.117902
SNORA74A	0.118216786	0.007238	0.013196
SNORA74B	-0.021307513	0.629505	0.691653
SNORA75	-0.011157515	0.80058	0.840352
SNORA76	0.094677327	0.0317	0.050608
SNORA77	-0.007956722	0.85705	0.887326
SNORA78	-0.011130055	0.80106	0.84068
SNORA79	0.027435895	0.534453	0.60566
SNORA7B	-0.196320811	7.18E-06	2.20E-05
SNORA80	0.061899584	0.160719	0.216575
SNORA81	-0.060740358	0.168716	0.226117
SNORA84	-0.04538263	0.303986	0.375762
SNORA8	-0.037341736	0.397745	0.472511
SNORA9	-0.067662803	0.125143	0.17344
SNORD10	-0.095259918	0.03066	0.049101
SNORD115-26	-0.014239781	0.747162	0.795604
SNORD116-20	-0.179867705	4.04E-05	0.000109
SNORD116-28	-0.257213299	3.16E-09	1.62E-08
SNORD116-4	-0.130234277	0.003067	0.00598
SNORD15A	0.086847218	0.04886	0.074899
SNORD15B	0.072944151	0.09822	0.139999
SNORD17	0.125906887	0.004213	0.008011
SNORD1C	-0.07329424	0.096612	0.137925
SNORD22	0.004794976	0.913558	0.932404
SNORD89	-0.007116263	0.872012	0.900205
SNORD94	-0.0829533	0.059948	0.090066

SNORD97	-0.027775575	0.529403	0.601233
SNPH	-0.123294151	0.00508	0.009535
SNRK	-0.199790854	4.90E-06	1.54E-05
SNRNP200	0.033091325	0.453651	0.527828
SNRNP25	0.163931322	0.000187	0.000454
SNRNP27	0.255880815	3.84E-09	1.94E-08
SNRNP35	-0.07091905	0.10794	0.152183
SNRNP40	0.221110173	4.01E-07	1.50E-06
SNRNP48	0.127165381	0.003845	0.007362
SNRNP70	-0.23976775	3.62E-08	1.59E-07
SNRPA1	0.453878965	1.55E-27	1.21E-25
SNRPA	0.174003919	7.20E-05	0.000187
SNRPB2	0.277759157	1.41E-10	8.89E-10
SNRPB	0.338464467	2.87E-15	3.72E-14
SNRPC	0.346633129	5.51E-16	7.93E-15
SNRPD1	0.471377016	7.56E-30	8.04E-28
SNRPD2	0.29328684	1.13E-11	8.35E-11
SNRPD3	0.15441647	0.000437	0.00099
SNRPE	0.376210282	9.25E-19	2.03E-17
SNRPF	0.43785324	1.56E-25	9.46E-24
SNRPG	0.373499514	1.71E-18	3.57E-17
SNRPN	-0.161343846	0.000236	0.000563
SNTA1	-0.02559492	0.562235	0.631594
SNTB1	-0.085765541	0.051753	0.078928
SNTB2	-0.108867891	0.013438	0.023211
SNTG1	0.024544333	0.578396	0.645684
SNTG2	-0.206523828	2.29E-06	7.56E-06
SNTN	-0.282891473	6.22E-11	4.12E-10
SNUPN	0.216629733	6.93E-07	2.49E-06
SNURF	-0.017079078	0.698999	0.754568
SNW1	0.320137525	9.75E-14	9.88E-13
SNX10	0.033615307	0.446529	0.520938
SNX11	0.092704897	0.035446	0.055981
SNX12	0.169050243	0.000116	0.000292
SNX13	-0.102487093	0.020004	0.033331
SNX14	-0.019217198	0.663498	0.722135
SNX15	0.030622972	0.488046	0.561378
SNX16	0.161980711	0.000223	0.000535
SNX17	0.10595129	0.016157	0.027436
SNX18	-0.256498122	3.51E-09	1.78E-08
SNX19	-0.12523535	0.004422	0.008381
SNX1	-0.363638307	1.52E-17	2.74E-16
SNX20	-0.263770533	1.21E-09	6.62E-09
SNX21	-0.096655277	0.028288	0.04568

SNX22	-0.071223589	0.106431	0.150258
SNX24	-0.17845981	4.65E-05	0.000125
SNX25	-0.365886585	9.28E-18	1.75E-16
SNX27	-0.044567603	0.312762	0.384849
SNX29	-0.396534264	7.71E-21	2.28E-19
SNX2	-0.146374214	0.000863	0.001856
SNX30	-0.409021642	3.44E-22	1.24E-20
SNX31	-0.081827492	0.063516	0.094876
SNX32	-0.049711412	0.260129	0.328955
SNX33	-0.183769183	2.72E-05	7.58E-05
SNX3	0.096979744	0.02776	0.044899
SNX4	0.105820055	0.01629	0.027638
SNX5	0.040834859	0.355057	0.429643
SNX6	0.356409628	7.17E-17	1.17E-15
SNX7	0.158443807	0.000306	0.000714
SNX8	0.046933026	0.287747	0.358683
SNX9	0.128233793	0.003556	0.006847
SOAT1	0.082496691	0.061374	0.092043
SOAT2	-0.175311166	6.34E-05	0.000166
SOBP	-0.217872095	5.96E-07	2.17E-06
SOCS1	-0.186065461	2.14E-05	6.09E-05
SOCS2	-0.111165121	0.011588	0.020288
SOCS3	0.006020103	0.891594	0.915268
SOCS4	0.3344136	6.38E-15	7.81E-14
SOCS5	-0.023161026	0.6	0.665343
SOCS6	0.100088227	0.023114	0.038024
SOCS7	0.013675893	0.756853	0.803825
SOD1	0.122949792	0.005206	0.009751
SOD2	0.129842564	0.003158	0.006142
SOD3	-0.302666515	2.26E-12	1.87E-11
SOHLH1	0.09616226	0.029108	0.046848
SOHLH2	0.074182733	0.092627	0.132728
SOLH	-0.097465532	0.026985	0.043763
SON	-0.253924096	5.08E-09	2.53E-08
SORBS1	-0.362801731	1.82E-17	3.25E-16
SORBS2	-0.197948481	6.01E-06	1.86E-05
SORBS3	-0.345378938	7.12E-16	1.00E-14
SORCS1	-0.312607786	3.88E-13	3.61E-12
SORCS2	-0.335471595	5.18E-15	6.43E-14
SORCS3	-0.085043452	0.053762	0.081693
SORD	0.324018376	4.71E-14	5.02E-13
SORL1	-0.211779983	1.24E-06	4.27E-06
SORT1	-0.313657614	3.21E-13	3.03E-12
SOS1	-0.016153961	0.71457	0.76777

SOS2	0.028331274	0.521194	0.593259
SOSTDC1	-0.326787629	2.79E-14	3.08E-13
SOST	-0.00205156	0.962956	0.971511
SOX10	-0.239961283	3.53E-08	1.55E-07
SOX11	-0.03892425	0.378037	0.452819
SOX12	0.097588408	0.026792	0.043492
SOX13	-0.320526761	9.07E-14	9.24E-13
SOX14	0.034777024	0.430968	0.505557
SOX15	0.112926252	0.010327	0.018276
SOX17	-0.248791214	1.05E-08	5.01E-08
SOX18	-0.157535384	0.000332	0.000769
SOX1	-0.135312213	0.002088	0.0042
SOX21	0.119218035	0.006757	0.01239
SOX2OT	-0.010327136	0.815143	0.852683
SOX2	-0.016706832	0.70525	0.760208
SOX30	0.059523747	0.177426	0.236379
SOX3	-0.036095313	0.413695	0.488343
SOX4	0.114930977	0.009041	0.016167
SOX5	-0.1806146	3.74E-05	0.000102
SOX6	-0.29086953	1.69E-11	1.22E-10
SOX7	-0.223106439	3.13E-07	1.19E-06
SOX8	-0.341916045	1.44E-15	1.94E-14
SOX9	-0.108184146	0.014036	0.024171
SP100	-0.136575779	0.001894	0.003832
SP110	-0.140694863	0.001369	0.002841
SP140L	-0.044015845	0.318796	0.391407
SP140	-0.118713531	0.006996	0.012787
SP1	0.029781912	0.500078	0.573009
SP2	-0.203253861	3.32E-06	1.07E-05
SP3	0.115076134	0.008953	0.016027
SP4	-0.121943992	0.005589	0.01041
SP5	0.049354343	0.263572	0.33274
SP6	0.151060282	0.000583	0.001291
SP7	-0.004836755	0.912808	0.931876
SP8	0.083627899	0.05789	0.087236
SP9	0.127820519	0.003666	0.00704
SPA17	0.034778094	0.430954	0.505557
SPACA1	-0.044586133	0.31256	0.384625
SPACA3	-0.064999713	0.140737	0.192741
SPACA4	-0.259550376	2.25E-09	1.18E-08
SPACA5	-0.194093863	9.15E-06	2.76E-05
SPAG11A	0.085098597	0.053606	0.081475
SPAG11B	0.067552638	0.12576	0.174211
SPAG16	-0.050656142	0.251169	0.319298

SPAG17	-0.289053904	2.28E-11	1.61E-10
SPAG1	-0.006706366	0.879326	0.905511
SPAG4	0.172517338	8.32E-05	0.000214
SPAG5	0.435476552	3.02E-25	1.76E-23
SPAG6	-0.199844859	4.87E-06	1.53E-05
SPAG7	-0.027454773	0.534171	0.605376
SPAG8	-0.36248908	1.95E-17	3.46E-16
SPAG9	-0.229148771	1.46E-07	5.85E-07
SPAM1	-0.039871854	0.366528	0.441228
SPANXA2	0.222392707	3.42E-07	1.30E-06
SPANXB2	0.230818728	1.18E-07	4.79E-07
SPANXC	0.242060711	2.65E-08	1.19E-07
SPANXE	0.231397807	1.09E-07	4.48E-07
SPANXN1	0.029784547	0.50004	0.572998
SPANXN2	0.055811967	0.206057	0.268983
SPANXN3	0.109917495	0.012563	0.02182
SPANXN4	0.094806128	0.031467	0.050277
SPANXN5	0.055579232	0.207957	0.27118
SPARCL1	-0.346808614	5.31E-16	7.69E-15
SPARC	0.067802695	0.124362	0.17249
SPAST	0.226175741	2.13E-07	8.32E-07
SPATA12	-0.006124888	0.889719	0.913625
SPATA13	-0.19709722	6.60E-06	2.03E-05
SPATA16	0.060621166	0.169555	0.227058
SPATA17	-0.074894631	0.089529	0.128844
SPATA18	-0.352237207	1.73E-16	2.66E-15
SPATA19	-0.203148549	3.36E-06	1.08E-05
SPATA1	-0.080322876	0.068557	0.10161
SPATA20	-0.205740628	2.50E-06	8.23E-06
SPATA21	0.051412174	0.244155	0.311554
SPATA22	0.006400979	0.884782	0.909743
SPATA24	-0.147793315	0.000767	0.001667
SPATA2L	-0.050000641	0.257363	0.326109
SPATA2	0.036553486	0.407789	0.482557
SPATA3	0.014015743	0.751008	0.799018
SPATA4	-0.167413255	0.000135	0.000336
SPATA5L1	0.234890644	6.92E-08	2.92E-07
SPATA5	0.234733037	7.07E-08	2.97E-07
SPATA6	-0.389002837	4.73E-20	1.24E-18
SPATA7	-0.085939411	0.051278	0.07827
SPATA8	0.076319258	0.083581	0.121191
SPATA9	-0.163153669	0.0002	0.000485
SPATC1	-0.194899032	8.39E-06	2.54E-05
SPATS1	-0.189773161	1.45E-05	4.25E-05

SPATS2L	0.053489066	0.225597	0.290881
SPATS2	0.45870262	3.68E-28	3.06E-26
SPC24	0.389299344	4.40E-20	1.16E-18
SPC25	0.478858869	7.06E-31	8.93E-29
SPCS1	0.178490173	4.63E-05	0.000124
SPCS2	0.213556647	1.00E-06	3.52E-06
SPCS3	0.213287103	1.04E-06	3.63E-06
SPDEF	0.020667321	0.639837	0.700886
SPDYA	-0.058038409	0.188507	0.248935
SPDYC	0.157389745	0.000337	0.000778
SPDYE1	-0.244869228	1.81E-08	8.33E-08
SPDYE2	-0.069095531	0.117328	0.163905
SPDYE3	0.122846032	0.005244	0.009817
SPDYE4	-0.094190683	0.032591	0.051878
SPDYE5	-0.20803567	1.92E-06	6.44E-06
SPDYE6	-0.18595219	2.17E-05	6.16E-05
SPDYE7P	-0.216837857	6.76E-07	2.44E-06
SPDYE8P	-0.204506791	2.88E-06	9.37E-06
SPEF1	-0.216862971	6.74E-07	2.43E-06
SPEF2	-0.329425991	1.68E-14	1.92E-13
SPEG	-0.153060558	0.000491	0.001102
SPEM1	0.081317888	0.065188	0.097114
SPEN	-0.259911262	2.13E-09	1.12E-08
SPERT	0.097400739	0.027087	0.04391
SPESP1	0.077044412	0.080678	0.117536
SPG11	-0.232834171	9.06E-08	3.76E-07
SPG20	-0.087455001	0.047295	0.07269
SPG21	0.066062634	0.134343	0.184973
SPG7	-0.207921582	1.94E-06	6.51E-06
SPHAR	-0.173495137	7.57E-05	0.000196
SPHK1	0.273695727	2.67E-10	1.61E-09
SPHK2	-0.156497746	0.000364	0.000836
SPHKAP	-0.135600182	0.002042	0.004115
SPI1	-0.247961973	1.18E-08	5.57E-08
SPIB	-0.23400761	7.77E-08	3.25E-07
SPIC	-0.049418736	0.262949	0.332016
SPIN1	0.168647528	0.00012	0.000302
SPIN2A	0.067841696	0.124145	0.172261
SPIN2B	-0.064794062	0.142001	0.194285
SPIN3	-0.14598436	0.000892	0.001911
SPIN4	0.26779402	6.59E-10	3.77E-09
SPINK13	-0.203257412	3.32E-06	1.07E-05
SPINK14	-0.068693437	0.119481	0.166576
SPINK1	-0.043047807	0.329566	0.402996

SPINK2	-0.21931724	5.00E-07	1.85E-06
SPINK4	0.087456117	0.047292	0.07269
SPINK5	-0.326717957	2.82E-14	3.12E-13
SPINK6	0.075671045	0.086247	0.124622
SPINK7	-0.062840029	0.154444	0.209077
SPINK8	-0.010248194	0.816531	0.85361
SPINK9	-0.048006357	0.276852	0.347378
SPINLW1	-0.214159011	9.33E-07	3.29E-06
SPINT1	-0.059366263	0.178577	0.237628
SPINT2	0.078793954	0.07401	0.108765
SPINT3	-0.016307714	0.711974	0.765722
SPINT4	-0.029578373	0.503014	0.575745
SPIRE1	-0.082160923	0.062441	0.093454
SPIRE2	-0.074421267	0.09158	0.131442
SPNS1	-0.078268606	0.075963	0.111463
SPNS2	-0.226279256	2.10E-07	8.23E-07
SPNS3	-0.360880485	2.76E-17	4.82E-16
SPN	-0.357805206	5.33E-17	8.89E-16
SPO11	-0.026273159	0.551919	0.621823
SPOCD1	0.128662752	0.003446	0.006654
SPOCK1	0.262464235	1.47E-09	7.92E-09
SPOCK2	-0.257822091	2.90E-09	1.49E-08
SPOCK3	-0.009454503	0.830516	0.86513
SPON1	-0.188867718	1.60E-05	4.64E-05
SPON2	-0.048240874	0.27451	0.344871
SPOPL	0.137507914	0.001761	0.003581
SPOP	-0.090334835	0.040439	0.063099
SPP1	0.166186748	0.000152	0.000374
SPP2	0.050251571	0.254979	0.323466
SPPL2A	0.050109366	0.256328	0.324971
SPPL2B	-0.199893373	4.84E-06	1.52E-05
SPPL3	0.029657795	0.501867	0.574696
SPRED1	-0.159616496	0.000276	0.00065
SPRED2	-0.253452144	5.43E-09	2.70E-08
SPRED3	0.168732269	0.000119	0.0003
SPRN	-0.130423691	0.003024	0.005902
SPRR1A	-0.065399455	0.138306	0.189801
SPRR1B	-0.011280996	0.79842	0.83879
SPRR2A	-0.024286656	0.582392	0.649311
SPRR2B	-0.016364954	0.711008	0.765053
SPRR2C	-0.040763108	0.355904	0.430436
SPRR2D	-0.005235833	0.905646	0.926221
SPRR2E	0.059171116	0.180012	0.23925
SPRR2F	-0.023385449	0.59647	0.662347

SPRR2G	-0.089288055	0.042827	0.066467
SPRR3	-0.049861021	0.258695	0.327453
SPRR4	-0.012121252	0.783764	0.82626
SPRY1	-0.308833165	7.64E-13	6.81E-12
SPRY2	-0.275698602	1.95E-10	1.20E-09
SPRY3	-0.081100625	0.065912	0.098112
SPRY4	-0.311758768	4.52E-13	4.17E-12
SPRYD3	-0.117839857	0.007427	0.013514
SPRYD4	0.154697706	0.000426	0.000968
SPRYD5	-0.199226968	5.22E-06	1.63E-05
SPR	-0.003463019	0.937512	0.950782
SPSB1	-0.094124072	0.032715	0.052054
SPSB2	-0.025817284	0.558843	0.628278
SPSB3	-0.357656532	5.50E-17	9.17E-16
SPSB4	-0.082246675	0.062167	0.0931
SPTA1	0.021899084	0.620021	0.683185
SPTAN1	-0.212815717	1.09E-06	3.81E-06
SPTBN1	-0.183387213	2.82E-05	7.85E-05
SPTBN2	-0.020692559	0.639428	0.700553
SPTBN4	-0.164295487	0.00018	0.00044
SPTBN5	-0.290482147	1.80E-11	1.29E-10
SPTB	-0.075165909	0.088371	0.127397
SPTLC1	0.220053043	4.57E-07	1.70E-06
SPTLC2	-0.109189257	0.013164	0.022786
SPTLC3	-0.347937166	4.21E-16	6.16E-15
SPTY2D1	0.22388948	2.84E-07	1.09E-06
SPZ1	0.034801036	0.430649	0.505361
SQLE	0.250780342	7.94E-09	3.85E-08
SQRDL	0.129372022	0.00327	0.00634
SQSTM1	-0.086399412	0.050041	0.076604
SR140	0.106096121	0.016011	0.027205
SRA1	0.038480408	0.383503	0.458082
SRBD1	0.015314067	0.728811	0.780261
SRCAP	-0.146296252	0.000869	0.001867
SRCIN1	-0.261273816	1.75E-09	9.32E-09
SRCRB4D	0.084338778	0.055785	0.084402
SRC	0.000439297	0.992065	0.994163
SRD5A1	0.129937595	0.003136	0.006101
SRD5A2	-0.104438887	0.017749	0.029892
SRD5A3	0.012142997	0.783386	0.825904
SREBF1	-0.107075389	0.015057	0.025771
SREBF2	-0.1378097	0.00172	0.003508
SRFBP1	0.156185068	0.000374	0.000857
SRF	-0.025855822	0.558256	0.62775

SRGAP1	0.268847861	5.62E-10	3.24E-09
SRGAP2	-0.183842778	2.70E-05	7.53E-05
SRGAP3	-0.408861207	3.58E-22	1.29E-20
SRGN	0.041051244	0.352511	0.427134
SRI	0.0714373	0.105383	0.148914
SRL	-0.369185646	4.48E-18	8.83E-17
SRMS	0.10867011	0.013608	0.023487
SRM	0.313920878	3.06E-13	2.89E-12
SRP14	0.079825765	0.070292	0.103897
SRP19	0.248458065	1.10E-08	5.23E-08
SRP54	0.264294796	1.12E-09	6.16E-09
SRP68	0.121180578	0.005896	0.010925
SRP72	0.407356709	5.24E-22	1.85E-20
SRP9	0.282225058	6.93E-11	4.56E-10
SRPK1	0.443241816	3.39E-26	2.26E-24
SRPK2	0.238544882	4.26E-08	1.85E-07
SRPK3	-0.027705763	0.530439	0.601999
SRPRB	0.352211657	1.74E-16	2.67E-15
SRPR	0.035819558	0.417274	0.491976
SRPX2	0.114965582	0.00902	0.016131
SRPX	-0.134668867	0.002194	0.004396
SRRD	0.271395207	3.80E-10	2.25E-09
SRRM1	-0.162129607	0.00022	0.000528
SRRM2	-0.299180979	4.14E-12	3.26E-11
SRRM3	-0.068097761	0.122729	0.170674
SRRM4	-0.018885656	0.668957	0.727007
SRRM5	-0.145388978	0.000936	0.002
SRRT	0.040385511	0.360381	0.43514
SRR	-0.073482613	0.095756	0.1368
SRXN1	0.325322114	3.68E-14	4.00E-13
SRY	0.074551897	0.09101	0.130777
SS18L1	-0.123230585	0.005103	0.009575
SS18L2	0.105926454	0.016182	0.027476
SS18	-0.059281076	0.179203	0.238317
SSBP1	0.335951565	4.72E-15	5.88E-14
SSBP2	-0.301491011	2.78E-12	2.25E-11
SSBP3	-0.193011344	1.03E-05	3.07E-05
SSBP4	-0.202271509	3.71E-06	1.19E-05
SSB	0.358879413	4.24E-17	7.17E-16
SSC5D	-0.26289518	1.37E-09	7.45E-09
SSFA2	0.005134026	0.907472	0.927661
SSH1	-0.05698934	0.196634	0.2582
SSH2	-0.193371044	9.90E-06	2.96E-05
SSH3	-0.133169974	0.00246	0.004879

SSNA1	0.071509238	0.105032	0.148491
SSPN	-0.098597261	0.025251	0.041191
SSPO	-0.269896468	4.79E-10	2.79E-09
SSR1	0.376124901	9.43E-19	2.06E-17
SSR2	0.166639889	0.000145	0.00036
SSR3	0.373086619	1.88E-18	3.87E-17
SSR4	0.073738625	0.094602	0.135306
SSRP1	0.301759345	2.65E-12	2.16E-11
SSSCA1	0.118826998	0.006942	0.012693
SSTR1	-0.183412684	2.82E-05	7.83E-05
SSTR2	0.242442949	2.52E-08	1.14E-07
SSTR3	-0.152922203	0.000497	0.001114
SSTR4	-0.215705976	7.75E-07	2.77E-06
SSTR5	-0.020552814	0.641693	0.702547
SST	0.03204328	0.468086	0.54181
SSU72	0.021006633	0.634352	0.695792
SSX1	0.076840801	0.081485	0.11853
SSX2IP	0.310090697	6.10E-13	5.53E-12
SSX2	0.064029776	0.146773	0.199952
SSX3	0.038543905	0.382718	0.457303
SSX4	0.068532394	0.120353	0.167673
SSX5	0.021629381	0.624337	0.687069
SSX6	0.106336983	0.015772	0.026848
SSX7	0.005924887	0.893299	0.916497
SSX8	0.038561042	0.382506	0.457077
ST13	0.097377433	0.027124	0.043956
ST14	-0.012163571	0.783028	0.825614
ST18	-0.049627116	0.260939	0.329729
ST20	0.014842893	0.736842	0.787004
ST3GAL1	-0.124468495	0.004673	0.008827
ST3GAL2	-0.147597702	0.00078	0.001691
ST3GAL3	-0.22725958	1.86E-07	7.32E-07
ST3GAL4	0.172493376	8.34E-05	0.000214
ST3GAL5	-0.379271288	4.60E-19	1.05E-17
ST3GAL6	-0.368465803	5.26E-18	1.03E-16
ST5	-0.365021902	1.12E-17	2.07E-16
ST6GAL1	-0.186051031	2.15E-05	6.10E-05
ST6GAL2	-0.03869042	0.38091	0.455416
ST6GALNAC1	-0.203416189	3.26E-06	1.05E-05
ST6GALNAC2	-0.267463937	6.93E-10	3.95E-09
ST6GALNAC3	0.058500345	0.185007	0.244831
ST6GALNAC4	-0.116023444	0.008401	0.015123
ST6GALNAC5	0.016072912	0.71594	0.768825
ST6GALNAC6	-0.401351329	2.36E-21	7.62E-20

ST7L	-0.197604251	6.24E-06	1.93E-05
ST7OT1	-0.21599181	7.49E-07	2.68E-06
ST7OT2	-0.07474856	0.090158	0.129665
ST7OT3	-0.127307059	0.003806	0.007289
ST7OT4	0.127514723	0.003749	0.007191
ST7	-0.11304505	0.010246	0.018153
ST8SIA1	-0.312201652	4.18E-13	3.87E-12
ST8SIA2	-0.08950202	0.04233	0.065741
ST8SIA3	-0.019275932	0.662533	0.721281
ST8SIA4	-0.131966293	0.002694	0.00531
ST8SIA5	-0.11207509	0.010921	0.019235
ST8SIA6	-0.173808965	7.34E-05	0.000191
STAB1	-0.158603199	0.000302	0.000705
STAB2	-0.078762891	0.074124	0.108925
STAC2	-0.243819301	2.09E-08	9.52E-08
STAC3	-0.198470683	5.67E-06	1.76E-05
STAC	-0.199051516	5.32E-06	1.66E-05
STAG1	0.112410694	0.010683	0.018848
STAG2	0.067523051	0.125926	0.174405
STAG3L1	-0.130683888	0.002966	0.005798
STAG3L2	-0.110051133	0.012455	0.021652
STAG3L3	-0.255722786	3.92E-09	1.98E-08
STAG3L4	0.124295976	0.004731	0.008929
STAG3	-0.057380819	0.193572	0.254667
STAM2	0.010007914	0.820758	0.857122
STAMBPL1	-0.027730977	0.530064	0.601745
STAMBP	0.274483534	2.36E-10	1.44E-09
STAM	0.27546326	2.02E-10	1.24E-09
STAP1	-0.24407595	2.02E-08	9.23E-08
STAP2	-0.069204876	0.116747	0.163242
STARD10	0.080062687	0.069461	0.10282
STARD13	-0.201244301	4.16E-06	1.32E-05
STARD3NL	0.192382688	1.10E-05	3.27E-05
STARD3	-0.06659329	0.131236	0.181069
STARD4	0.07791611	0.077297	0.113138
STARD5	-0.248584316	1.08E-08	5.15E-08
STARD6	0.056733127	0.198657	0.260429
STARD7	0.174484544	6.87E-05	0.000179
STARD8	-0.228815116	1.52E-07	6.08E-07
STAR	-0.208076376	1.91E-06	6.41E-06
STAT1	0.134384437	0.002242	0.004479
STAT2	-0.195313888	8.02E-06	2.43E-05
STAT3	-0.181418125	3.45E-05	9.46E-05
STAT4	-0.156207764	0.000373	0.000856

STAT5A	-0.302458323	2.35E-12	1.93E-11
STAT5B	-0.261756775	1.63E-09	8.72E-09
STAT6	-0.305036477	1.50E-12	1.27E-11
STATH	-0.08259658	0.06106	0.091626
STAU1	0.167809587	0.00013	0.000325
STAU2	0.229851211	1.33E-07	5.38E-07
STBD1	0.003824726	0.931	0.945955
STC1	0.326542084	2.92E-14	3.21E-13
STC2	0.359466058	3.74E-17	6.40E-16
STEAP1	0.278126337	1.33E-10	8.42E-10
STEAP2	0.107727471	0.014449	0.024807
STEAP3	-0.199889348	4.84E-06	1.52E-05
STEAP4	-0.224092179	2.77E-07	1.06E-06
STH	-0.038145218	0.387663	0.462493
STIL	0.390873927	3.03E-20	8.18E-19
STIM1	-0.135949387	0.001988	0.004012
STIM2	-0.102895613	0.019512	0.032593
STIP1	0.394411712	1.29E-20	3.66E-19
STK10	-0.230237637	1.27E-07	5.15E-07
STK11IP	-0.197784238	6.12E-06	1.89E-05
STK11	-0.278436262	1.27E-10	8.04E-10
STK16	-0.029228974	0.508073	0.580539
STK17A	0.026402608	0.549961	0.620176
STK17B	-0.073077171	0.097606	0.139215
STK19	-0.147799409	0.000767	0.001666
STK24	0.151775153	0.000548	0.00122
STK25	0.000947643	0.982884	0.987231
STK31	0.075025884	0.088968	0.128137
STK32A	-0.245670533	1.62E-08	7.50E-08
STK32B	-0.122722045	0.005291	0.009893
STK32C	0.052839801	0.231288	0.297393
STK33	-0.211600595	1.26E-06	4.36E-06
STK35	0.064099674	0.146331	0.199459
STK36	-0.289303202	2.19E-11	1.55E-10
STK38L	0.259195953	2.37E-09	1.24E-08
STK38	0.00209189	0.962229	0.971071
STK39	-0.129451141	0.003251	0.006307
STK3	0.201203712	4.18E-06	1.33E-05
STK40	-0.228604722	1.56E-07	6.24E-07
STK4	-0.104201932	0.01801	0.030302
STL	-0.259083682	2.41E-09	1.26E-08
STMN1	0.320310467	9.44E-14	9.59E-13
STMN2	-0.097603072	0.026769	0.043468
STMN3	-0.095987472	0.029403	0.047297

STMN4	-0.070535677	0.109862	0.154578
STOML1	-0.14386397	0.001061	0.002245
STOML2	0.424281279	6.41E-24	3.03E-22
STOML3	-0.179249162	4.29E-05	0.000116
STOM	-0.20948042	1.62E-06	5.50E-06
STON1-GTF2A1L	0.055135547	0.211616	0.275269
STON1	-0.099808297	0.023503	0.038582
STON2	0.003650261	0.93414	0.948388
STOX1	0.030750826	0.486231	0.559644
STOX2	-0.145508516	0.000927	0.001983
STRA13	0.214340497	9.13E-07	3.23E-06
STRA6	0.121531719	0.005753	0.010684
STRA8	-0.013907666	0.752865	0.800632
STRADA	-0.305472302	1.39E-12	1.19E-11
STRADB	-0.038860971	0.378813	0.453641
STRAP	0.513472242	5.50E-36	1.80E-33
STRBP	0.115658674	0.00861	0.015453
STRC	-0.207921202	1.94E-06	6.51E-06
STRN3	0.307896346	9.03E-13	7.96E-12
STRN4	0.041959284	0.341954	0.416132
STRN	0.131574232	0.002775	0.00545
STS	-0.20657999	2.27E-06	7.51E-06
STT3A	0.218368158	5.61E-07	2.06E-06
STT3B	0.154206596	0.000445	0.001007
STUB1	-0.079202503	0.072519	0.106841
STX10	-0.078332671	0.075723	0.111143
STX11	-0.212774781	1.10E-06	3.83E-06
STX12	-0.186967373	1.95E-05	5.59E-05
STX16	-0.185285827	2.32E-05	6.56E-05
STX17	-0.193696786	9.56E-06	2.87E-05
STX18	0.230872415	1.17E-07	4.76E-07
STX19	-0.217932485	5.92E-07	2.16E-06
STX1A	0.134664896	0.002194	0.004396
STX1B	-0.104026471	0.018206	0.0306
STX2	0.046576378	0.29143	0.362663
STX3	-0.046403465	0.293227	0.364507
STX4	-0.159027783	0.000291	0.000681
STX5	0.086301555	0.050302	0.076974
STX6	-0.125947152	0.004201	0.00799
STX7	-0.048553958	0.271404	0.341355
STX8	0.098342284	0.025633	0.04176
STXBP1	-0.327431074	2.47E-14	2.74E-13
STXBP2	-0.20771896	1.99E-06	6.65E-06
STXBP3	0.035113073	0.426525	0.50114

STXBP4	-0.105471875	0.016647	0.028192
STXBP5L	0.163872571	0.000188	0.000456
STXBP5	0.148411207	0.000729	0.00159
STXBP6	-0.197206977	6.52E-06	2.01E-05
STYK1	0.169818797	0.000108	0.000273
STYXL1	0.15897617	0.000292	0.000684
STYX	0.480688995	3.92E-31	5.26E-29
SUB1	0.144237608	0.001029	0.002184
SUCLA2	0.209842128	1.55E-06	5.28E-06
SUCLG1	0.140335781	0.001409	0.002918
SUCLG2	0.155464575	0.000399	0.000909
SUCNR1	-0.099437946	0.024026	0.039347
SUDS3	0.259276356	2.34E-09	1.22E-08
SUFU	-0.22959515	1.38E-07	5.55E-07
SUGT1L1	-0.39418943	1.36E-20	3.84E-19
SUGT1P1	-0.061516859	0.163327	0.21966
SUGT1	0.281241362	8.11E-11	5.30E-10
SULF1	0.220958585	4.09E-07	1.53E-06
SULF2	0.06811678	0.122624	0.170576
SULT1A1	-0.333106387	8.24E-15	9.83E-14
SULT1A2	-0.233492442	8.31E-08	3.46E-07
SULT1A3	-0.013626644	0.757701	0.804512
SULT1B1	-0.045091379	0.307103	0.378936
SULT1C2	-0.334071179	6.82E-15	8.29E-14
SULT1C3	-0.002138957	0.961379	0.97041
SULT1C4	-0.281290111	8.05E-11	5.26E-10
SULT1E1	-0.177985723	4.87E-05	0.00013
SULT2A1	0.022421986	0.61169	0.675868
SULT2B1	0.114631067	0.009223	0.016481
SULT4A1	-0.093012206	0.034839	0.055096
SULT6B1	0.043664679	0.322676	0.395748
SUMF1	-0.023177104	0.599747	0.665099
SUMF2	0.015040579	0.733469	0.784156
SUMO1P1	-0.094995103	0.031129	0.049776
SUMO1P3	0.228220502	1.64E-07	6.53E-07
SUMO1	0.242165506	2.62E-08	1.17E-07
SUMO2	0.255496706	4.05E-09	2.04E-08
SUMO3	0.065420664	0.138177	0.189664
SUMO4	-0.058068055	0.188281	0.248735
SUN1	-0.033795118	0.4441	0.518346
SUN2	-0.250907259	7.80E-09	3.80E-08
SUN3	0.084823179	0.054388	0.082575
SUOX	-0.206182607	2.38E-06	7.84E-06
SUPT16H	0.258353438	2.68E-09	1.39E-08

SUPT3H	0.16948057	0.000111	0.000281
SUPT4H1	0.148377568	0.000731	0.001595
SUPT5H	-0.01772765	0.688156	0.744795
SUPT6H	-0.180142115	3.93E-05	0.000107
SUPT7L	-0.021117914	0.632557	0.694181
SUPV3L1	0.304236653	1.72E-12	1.45E-11
SURF1	-0.022701976	0.607249	0.671778
SURF2	0.005889993	0.893923	0.917
SURF4	0.198750694	5.50E-06	1.71E-05
SURF6	-0.155080106	0.000412	0.000938
SUSD1	-0.058619628	0.184112	0.243823
SUSD2	-0.469187902	1.50E-29	1.55E-27
SUSD3	-0.196578349	6.98E-06	2.14E-05
SUSD4	-0.378070273	6.05E-19	1.36E-17
SUSD5	0.064541341	0.143565	0.196184
SUV39H1	0.300357927	3.38E-12	2.70E-11
SUV39H2	0.468014409	2.15E-29	2.14E-27
SUV420H1	0.078901512	0.073615	0.108256
SUV420H2	-0.164798771	0.000172	0.000422
SUZ12P	-0.139809496	0.001469	0.003033
SUZ12	0.209792914	1.56E-06	5.31E-06
SV2A	0.156992537	0.000349	0.000803
SV2B	-0.081166782	0.065691	0.097819
SV2C	-0.16681246	0.000143	0.000355
SVEP1	-0.30151157	2.77E-12	2.25E-11
SVIL	-0.149912097	0.000642	0.001414
SVIP	-0.012942793	0.769509	0.814672
SVOPL	-0.046445044	0.292794	0.364043
SVOP	0.054595537	0.216131	0.280466
SWAP70	-0.191013374	1.27E-05	3.76E-05
SYAP1	0.177432006	5.14E-05	0.000137
SYBU	-0.212654797	1.12E-06	3.88E-06
SYCE1L	-0.086961718	0.048562	0.074482
SYCE1	-0.07130506	0.106031	0.149713
SYCE2	0.228213585	1.64E-07	6.53E-07
SYCN	-0.061363444	0.164382	0.220885
SYCP1	-0.024481571	0.579368	0.646553
SYCP2L	0.074420093	0.091585	0.131442
SYCP2	-0.103810167	0.018449	0.030968
SYCP3	-0.165060495	0.000168	0.000412
SYDE1	-0.075876044	0.085396	0.123572
SYDE2	-0.187298035	1.89E-05	5.41E-05
SYF2	-0.194962175	8.33E-06	2.52E-05
SYK	-0.207534624	2.03E-06	6.78E-06

SYMPK	-0.059588898	0.176951	0.235834
SYN1	-0.046905439	0.28803	0.35897
SYN2	-0.094055303	0.032843	0.052226
SYN3	-0.084202669	0.056183	0.084966
SYNCRIP	0.207546285	2.03E-06	6.77E-06
SYNC	-0.206735695	2.23E-06	7.39E-06
SYNE1	-0.414049251	9.47E-23	3.72E-21
SYNE2	-0.200863207	4.35E-06	1.38E-05
SYNGAP1	-0.256017221	3.76E-09	1.91E-08
SYNGR1	-0.280546882	9.07E-11	5.88E-10
SYNGR2	-0.085789219	0.051688	0.078837
SYNGR3	0.102778437	0.019652	0.032805
SYNGR4	0.171554676	9.12E-05	0.000234
SYNJ1	-0.373770137	1.61E-18	3.37E-17
SYNJ2BP	-0.079262817	0.072301	0.106567
SYNJ2	0.118341487	0.007177	0.01309
SYNM	-0.198117842	5.90E-06	1.83E-05
SYNPO2L	-0.051524641	0.243124	0.310455
SYNPO2	-0.279636118	1.05E-10	6.73E-10
SYNPO	-0.353271408	1.39E-16	2.16E-15
SYNPR	-0.007674262	0.862073	0.89151
SYNRG	-0.207395648	2.07E-06	6.88E-06
SYPL1	0.137053352	0.001825	0.003702
SYPL2	-0.091416584	0.038091	0.059723
SYP	-0.216211115	7.29E-07	2.62E-06
SYS1-DBNDD2	-0.065477365	0.137835	0.189247
SYS1	-0.06923238	0.116602	0.163073
SYT10	-0.020753533	0.638442	0.699626
SYT11	-0.104363223	0.017832	0.030017
SYT12	0.01919972	0.663786	0.72229
SYT13	0.161056416	0.000242	0.000577
SYT14L	-0.108995206	0.013329	0.02304
SYT14	0.057924613	0.189376	0.249868
SYT15	-0.423224769	8.49E-24	3.91E-22
SYT16	-0.049237496	0.264706	0.334003
SYT17	-0.120059035	0.006375	0.011737
SYT1	0.160357096	0.000258	0.000611
SYT2	-0.289781456	2.02E-11	1.44E-10
SYT3	-0.167035582	0.00014	0.000348
SYT4	-0.029494409	0.504227	0.57687
SYT5	0.164503266	0.000177	0.000433
SYT6	-0.141198607	0.001315	0.002738
SYT7	0.116823356	0.007959	0.014402
SYT8	-0.255689265	3.94E-09	1.99E-08

SYT9	-0.008014759	0.856018	0.886579
SYTL1	-0.32528628	3.71E-14	4.02E-13
SYTL2	-0.226221186	2.12E-07	8.28E-07
SYTL3	-0.167861531	0.00013	0.000324
SYTL4	-0.091461012	0.037997	0.059609
SYTL5	-0.033344431	0.450203	0.524519
SYVN1	-0.109676045	0.012759	0.022137
TAAR1	0.028588685	0.517414	0.589594
TAAR6	0.015455254	0.726411	0.777983
TAAR8	0.084808085	0.054431	0.082628
TAAR9	0.003060061	0.944771	0.956156
TAB1	-0.172070045	8.68E-05	0.000223
TAB2	0.058538123	0.184723	0.244504
TAB3	-0.140033664	0.001443	0.002983
TAC1	0.11200313	0.010972	0.019311
TAC3	0.044986265	0.308233	0.380119
TAC4	-0.067783009	0.124472	0.172618
TACC1	-0.312591877	3.89E-13	3.62E-12
TACC2	0.034038482	0.440824	0.515154
TACC3	0.385385676	1.11E-19	2.79E-18
TACO1	0.367739969	6.17E-18	1.19E-16
TACR1	-0.150761498	0.000598	0.001322
TACR2	-0.061217252	0.165391	0.222107
TACR3	0.151009332	0.000585	0.001296
TACSTD2	-0.131640019	0.002761	0.005426
TADA1	0.174024412	7.19E-05	0.000187
TADA2A	0.135683399	0.002029	0.00409
TADA2B	-0.141605307	0.001273	0.002658
TADA3	-0.070436357	0.110365	0.155219
TAF10	0.137641656	0.001743	0.003549
TAF11	0.192500979	1.09E-05	3.24E-05
TAF12	0.053349439	0.226813	0.292241
TAF13	0.315544636	2.27E-13	2.19E-12
TAF15	-0.082337962	0.061877	0.092706
TAF1A	0.316334752	1.97E-13	1.91E-12
TAF1B	0.128330252	0.003531	0.006805
TAF1C	-0.276381324	1.75E-10	1.09E-09
TAF1D	0.266809485	7.65E-10	4.33E-09
TAF1L	-0.105478676	0.01664	0.028182
TAF1	-0.257344178	3.10E-09	1.59E-08
TAF2	0.323329036	5.37E-14	5.65E-13
TAF3	0.001238178	0.977638	0.983493
TAF4B	-0.03735725	0.397549	0.472314
TAF4	0.116586449	0.008088	0.014618

TAF5L	0.168755172	0.000119	0.000299
TAF5	0.293699938	1.05E-11	7.83E-11
TAF6L	-0.157253377	0.000341	0.000787
TAF6	0.148340708	0.000733	0.001599
TAF7L	0.021491987	0.626541	0.689267
TAF7	0.069776725	0.113749	0.159574
TAF8	-0.110216219	0.012323	0.021445
TAF9B	-0.044945656	0.308671	0.380588
TAF9	0.285938009	3.80E-11	2.60E-10
TAGAP	-0.242865713	2.38E-08	1.08E-07
TAGLN2	-0.020339941	0.645148	0.705621
TAGLN3	0.091459364	0.038	0.05961
TAGLN	-0.022877164	0.604478	0.669343
TAL1	-0.333676418	7.37E-15	8.90E-14
TAL2	0.033601806	0.446712	0.521035
TALDO1	0.169400736	0.000112	0.000283
TANC1	-0.244401741	1.93E-08	8.85E-08
TANC2	-0.123099953	0.005151	0.009658
TANK	0.027003445	0.540916	0.611667
TAOK1	0.075250063	0.088014	0.126929
TAOK2	-0.329808458	1.56E-14	1.80E-13
TAOK3	-0.1545906	0.00043	0.000977
TAP1	0.115550952	0.008673	0.01556
TAP2	0.073317122	0.096508	0.137795
TAPBPL	0.050997707	0.247983	0.315813
TAPBP	-0.171103259	9.53E-05	0.000243
TAPT1	-0.423365239	8.18E-24	3.78E-22
TARBP1	-0.135297449	0.00209	0.004204
TARBP2	0.265007859	1.00E-09	5.58E-09
TARDBP	0.061867105	0.160939	0.216828
TARM1	0.024458291	0.579729	0.646847
TARP	-0.145294939	0.000944	0.002014
TARS2	0.063043404	0.153111	0.20747
TARSL2	-0.184288153	2.58E-05	7.22E-05
TARS	0.405710532	7.94E-22	2.74E-20
TAS1R1	-0.14688933	0.000827	0.001785
TAS1R2	-0.126772236	0.003957	0.007565
TAS1R3	0.118398624	0.007149	0.013042
TAS2R10	-0.191438141	1.22E-05	3.60E-05
TAS2R13	-0.04164715	0.34556	0.419805
TAS2R14	-0.159754177	0.000273	0.000642
TAS2R19	-0.008509908	0.847229	0.879299
TAS2R1	0.01920316	0.663729	0.722268
TAS2R20	-0.04628968	0.294413	0.365761

TAS2R30	0.048076988	0.276145	0.346578
TAS2R31	-0.013278753	0.763701	0.809893
TAS2R38	-0.031059666	0.481861	0.55567
TAS2R39	0.050266645	0.254837	0.323326
TAS2R3	-0.095607325	0.030054	0.048263
TAS2R40	0.030193879	0.494165	0.567513
TAS2R41	-0.074682869	0.090442	0.130026
TAS2R42	-0.011794467	0.789455	0.831076
TAS2R43	-0.064857891	0.141607	0.1938
TAS2R46	0.00411351	0.925805	0.942113
TAS2R4	-0.240582381	3.24E-08	1.44E-07
TAS2R50	-0.036368368	0.410169	0.484817
TAS2R5	-0.128101431	0.003591	0.006908
TAS2R60	0.039685696	0.368771	0.443475
TAS2R7	0.024831763	0.573953	0.641761
TAS2R8	-0.039855002	0.36673	0.441445
TAS2R9	0.042927076	0.330925	0.40446
TASP1	-0.014959922	0.734844	0.785374
TATDN1	0.324377121	4.40E-14	4.72E-13
TATDN2	0.00682967	0.877125	0.903989
TATDN3	0.049779852	0.259472	0.328291
TAT	0.08067023	0.067365	0.100036
TAX1BP1	0.090145396	0.040863	0.06372
TAX1BP3	-0.027644717	0.531345	0.602857
TAZ	-0.047569135	0.281256	0.351931
TBC1D10A	-0.236811435	5.37E-08	2.30E-07
TBC1D10B	0.014005704	0.75118	0.799159
TBC1D10C	-0.258350388	2.68E-09	1.39E-08
TBC1D12	-0.042652205	0.334034	0.40781
TBC1D13	-0.151449827	0.000564	0.001252
TBC1D14	-0.05978524	0.175526	0.234113
TBC1D15	0.111045875	0.011679	0.020424
TBC1D16	0.049784675	0.259426	0.328274
TBC1D17	-0.453003738	2.00E-27	1.55E-25
TBC1D19	-0.021435042	0.627455	0.690078
TBC1D1	-0.024711116	0.575816	0.643415
TBC1D20	-0.253405941	5.47E-09	2.71E-08
TBC1D22A	-0.243941424	2.05E-08	9.38E-08
TBC1D22B	-0.02617175	0.553456	0.623168
TBC1D23	0.151487928	0.000562	0.001249
TBC1D24	-0.220229761	4.47E-07	1.66E-06
TBC1D25	-0.152748608	0.000504	0.00113
TBC1D26	-0.109650213	0.012781	0.022168
TBC1D28	0.068893893	0.118404	0.1652

TBC1D29	-0.116980358	0.007875	0.01426
TBC1D2B	-0.320881083	8.49E-14	8.66E-13
TBC1D2	-0.156950915	0.00035	0.000806
TBC1D3B	-0.219396227	4.95E-07	1.83E-06
TBC1D3C	-0.106274989	0.015833	0.026936
TBC1D3G	-0.072837594	0.098713	0.140643
TBC1D3H	-0.224374559	2.67E-07	1.03E-06
TBC1D3P2	0.035551473	0.42077	0.495397
TBC1D3	-0.177081489	5.33E-05	0.000141
TBC1D4	-0.128907543	0.003384	0.006544
TBC1D5	-0.146727425	0.000838	0.001807
TBC1D7	0.266440941	8.09E-10	4.56E-09
TBC1D8B	-0.141382843	0.001296	0.002702
TBC1D8	-0.194384201	8.87E-06	2.68E-05
TBC1D9B	-0.269000908	5.49E-10	3.17E-09
TBC1D9	-0.265504978	9.31E-10	5.21E-09
TBCA	0.252142573	6.55E-09	3.21E-08
TBCB	0.089615919	0.042067	0.065404
TBCCD1	0.120839826	0.006038	0.011165
TBCC	-0.001196921	0.978383	0.983995
TBCD	-0.257257925	3.14E-09	1.61E-08
TBCEL	-0.20340177	3.26E-06	1.05E-05
TBCE	0.284305823	4.95E-11	3.32E-10
TBCK	-0.127015114	0.003888	0.007437
TBK1	0.210377858	1.46E-06	4.98E-06
TBKBP1	-0.088421411	0.044893	0.069383
TBL1XR1	0.142791725	0.001157	0.002433
TBL1X	0.127501787	0.003752	0.007196
TBL1Y	-0.016168144	0.714331	0.767687
TBL2	0.321803642	7.15E-14	7.41E-13
TBL3	-0.103756169	0.018511	0.031063
TBPL1	0.169776174	0.000108	0.000274
TBPL2	0.043272725	0.327043	0.400302
TBP	0.133110579	0.002471	0.004897
TBR1	0.088645041	0.044352	0.068606
TBRG1	-0.269556332	5.04E-10	2.93E-09
TBRG4	0.387128518	7.37E-20	1.88E-18
TBX10	0.169101431	0.000115	0.00029
TBX15	0.111268551	0.011511	0.020171
TBX18	0.089664573	0.041955	0.065255
TBX19	-0.285528236	4.06E-11	2.77E-10
TBX1	-0.06706089	0.128544	0.177686
TBX20	0.046817252	0.288939	0.360012
TBX21	-0.103731157	0.018539	0.031108

TBX22	-0.101609171	0.021097	0.034997
TBX2	-0.356654934	6.81E-17	1.12E-15
TBX3	-0.224183945	2.74E-07	1.05E-06
TBX4	-0.388558604	5.25E-20	1.37E-18
TBX5	-0.314034077	3.00E-13	2.84E-12
TBX6	-0.17642993	5.68E-05	0.00015
TBXA2R	0.029011944	0.51123	0.583512
TBXAS1	-0.119838499	0.006474	0.011906
TC2N	0.100707546	0.022273	0.036763
TCAM1P	0.050803153	0.249794	0.317776
TCAP	-0.197916212	6.03E-06	1.87E-05
TCEA1	0.27284634	3.04E-10	1.82E-09
TCEA2	-0.210242818	1.48E-06	5.06E-06
TCEA3	-0.355546601	8.61E-17	1.38E-15
TCEAL1	-0.047802814	0.278897	0.349416
TCEAL2	-0.356492028	7.05E-17	1.15E-15
TCEAL3	-0.29918328	4.14E-12	3.26E-11
TCEAL4	-0.253430802	5.45E-09	2.71E-08
TCEAL5	-0.232147424	9.91E-08	4.08E-07
TCEAL6	-0.312106025	4.25E-13	3.93E-12
TCEAL7	-0.127566863	0.003734	0.007165
TCEAL8	-0.10799142	0.014209	0.024445
TCEANC	-0.266324729	8.23E-10	4.63E-09
TCEB1	0.428396042	2.11E-24	1.07E-22
TCEB2	-0.008738246	0.843183	0.875807
TCEB3B	0.092716421	0.035423	0.055962
TCEB3C	-0.044380787	0.314797	0.38709
TCEB3	-0.268899236	5.57E-10	3.22E-09
TCERG1L	-0.052913735	0.230635	0.296649
TCERG1	0.12420593	0.004761	0.008983
TCF12	0.090122236	0.040915	0.063797
TCF15	-0.051106366	0.246976	0.314711
TCF19	0.17051886	0.000101	0.000256
TCF20	-0.115236063	0.008858	0.015868
TCF21	-0.401918642	2.05E-21	6.69E-20
TCF23	-0.061025139	0.166725	0.223672
TCF25	-0.191226032	1.25E-05	3.68E-05
TCF3	0.106894262	0.015229	0.026017
TCF4	-0.186415848	2.07E-05	5.89E-05
TCF7L1	-0.25192943	6.75E-09	3.31E-08
TCF7L2	-0.17559896	6.16E-05	0.000162
TCF7	-0.126023726	0.004178	0.007951
TCFL5	0.002389859	0.956853	0.967062
TCHHL1	-0.001512929	0.972677	0.979538

TCHH	-0.160717495	0.00025	0.000593
TCHP	-0.022261495	0.614241	0.678088
TCIRG1	-0.217699449	6.09E-07	2.22E-06
TCL1A	-0.096384259	0.028736	0.046317
TCL1B	-0.010247587	0.816541	0.85361
TCL6	0.013128113	0.766304	0.811964
TCN1	0.047752338	0.279405	0.349943
TCN2	-0.131255409	0.002842	0.005573
TCOF1	0.13336924	0.002423	0.004812
TCP10L2	-0.007126757	0.871825	0.900152
TCP10L	-0.068982829	0.117928	0.164629
TCP10	0.048898244	0.268016	0.337668
TCP11L1	0.047781463	0.279112	0.34962
TCP11L2	-0.082099667	0.062638	0.093706
TCP11	-0.026263481	0.552066	0.621953
TCP1	0.363828647	1.46E-17	2.64E-16
TCTA	-0.217788221	6.02E-07	2.19E-06
TCTE1	-0.263075709	1.34E-09	7.27E-09
TCTE3	-0.147680088	0.000775	0.001681
TCTEX1D1	-0.23115252	1.13E-07	4.61E-07
TCTEX1D2	0.06111519	0.166099	0.222967
TCTEX1D4	-0.332414244	9.43E-15	1.12E-13
TCTN1	-0.101096775	0.021758	0.035985
TCTN2	-0.101715231	0.020962	0.034803
TCTN3	0.207964617	1.93E-06	6.48E-06
TDGF1	-0.014080366	0.749898	0.798177
TDGF3	0.016793851	0.703787	0.758917
TDG	0.436474049	2.29E-25	1.35E-23
TDH	-0.136220218	0.001947	0.003932
TDO2	0.146105182	0.000883	0.001895
TDP1	0.200127587	4.72E-06	1.49E-05
TDP2	0.243638689	2.14E-08	9.75E-08
TDRD10	-0.354603604	1.05E-16	1.66E-15
TDRD12	0.094611207	0.03182	0.050767
TDRD1	-0.066716531	0.130522	0.180184
TDRD3	-0.213489034	1.01E-06	3.55E-06
TDRD5	0.100927262	0.021981	0.036326
TDRD6	-0.296322547	6.74E-12	5.18E-11
TDRD7	0.023849934	0.589195	0.655434
TDRD9	-0.050040884	0.256979	0.325714
TDRG1	0.116638788	0.008059	0.014572
TDRKH	-0.021347345	0.628864	0.691214
TEAD1	0.047392678	0.283046	0.353817
TEAD2	0.001212192	0.978107	0.983767

TEAD3	-0.112362302	0.010717	0.018903
TEAD4	0.417065733	4.32E-23	1.79E-21
TECPR1	-0.182843398	2.99E-05	8.27E-05
TECPR2	-0.140537446	0.001387	0.002875
TECRL	0.041543079	0.346767	0.421144
TECR	-0.106185369	0.015922	0.027073
TECTA	-0.37073135	3.18E-18	6.38E-17
TECTB	0.027419125	0.534702	0.605909
TEC	-0.050554181	0.252126	0.320313
TEDDM1	-0.226274106	2.10E-07	8.23E-07
TEF	-0.477243832	1.18E-30	1.46E-28
TEKT1	-0.202085339	3.79E-06	1.21E-05
TEKT2	-0.250994127	7.70E-09	3.75E-08
TEKT3	-0.280138705	9.68E-11	6.25E-10
TEKT4	-0.264522642	1.08E-09	5.97E-09
TEKT5	-0.151958331	0.00054	0.001203
TEK	-0.269092203	5.41E-10	3.13E-09
TELO2	-0.169515848	0.000111	0.00028
TENC1	-0.443821209	2.88E-26	1.94E-24
TEP1	-0.259975377	2.11E-09	1.11E-08
TEPP	-0.391582711	2.55E-20	7.02E-19
TERC	0.117794864	0.00745	0.013548
TERF1	0.242213962	2.60E-08	1.17E-07
TERF2IP	-0.298835256	4.39E-12	3.44E-11
TERF2	-0.018771189	0.670845	0.728666
TERT	0.159295529	0.000284	0.000667
TESC	0.034371413	0.436365	0.510811
TESK1	-0.071411754	0.105508	0.149069
TESK2	-0.308404745	8.25E-13	7.32E-12
TES	0.212984427	1.07E-06	3.75E-06
TET1	0.15764769	0.000329	0.000762
TET2	-0.305099368	1.48E-12	1.26E-11
TET3	-0.072213852	0.101641	0.14427
TEX101	0.061098284	0.166216	0.223065
TEX10	0.398850937	4.37E-21	1.35E-19
TEX11	0.076527038	0.082741	0.120138
TEX12	-0.07570354	0.086112	0.124445
TEX13A	0.204567933	2.86E-06	9.31E-06
TEX13B	-0.021788169	0.621794	0.684887
TEX14	-0.1280271	0.003611	0.006943
TEX15	0.203916296	3.08E-06	9.97E-06
TEX19	0.281105318	8.29E-11	5.41E-10
TEX261	-0.107087969	0.015045	0.025755
TEX264	-0.081115048	0.065863	0.098062

TEX2	-0.148873207	0.000701	0.001535
TEX9	-0.16198331	0.000223	0.000535
TFAMP1	0.034035667	0.440862	0.515168
TFAM	0.449789305	5.14E-27	3.76E-25
TFAP2A	0.088319788	0.045141	0.069722
TFAP2B	-0.040564838	0.35825	0.432802
TFAP2C	-0.12837521	0.003519	0.006785
TFAP2D	-0.007597109	0.863446	0.892653
TFAP2E	-0.21915843	5.10E-07	1.88E-06
TFAP4	0.058921292	0.181861	0.241242
TFB1M	0.073970904	0.093565	0.133938
TFB2M	0.294103384	9.81E-12	7.35E-11
TFCP2L1	-0.18491132	2.42E-05	6.80E-05
TFCP2	0.198319326	5.77E-06	1.79E-05
TFDP1	0.205159565	2.67E-06	8.73E-06
TFDP2	0.117853459	0.007421	0.013503
TFDP3	-0.052337299	0.235761	0.302367
TFE3	-0.061323173	0.164659	0.221213
TFEB	-0.440851678	6.69E-26	4.27E-24
TFEC	-0.136179936	0.001953	0.003944
TFF1	0.20782874	1.97E-06	6.58E-06
TFF2	0.030517158	0.489551	0.562818
TFF3	0.010418863	0.813531	0.85122
TFG	0.328375873	2.06E-14	2.32E-13
TFIP11	0.02581476	0.558881	0.628286
TFPI2	0.05105413	0.24746	0.315247
TFPI	-0.078502797	0.075087	0.110275
TFPT	-0.010662482	0.809254	0.847499
TFR2	0.15244933	0.000517	0.001157
TFRC	0.183477915	2.80E-05	7.79E-05
TF	0.092432288	0.035992	0.056767
TGDS	0.100493145	0.022561	0.037179
TGFA	0.001848765	0.966616	0.974416
TGFB1I1	-0.103669394	0.01861	0.031215
TGFB1	-0.237872874	4.66E-08	2.01E-07
TGFB2	-0.175846322	6.02E-05	0.000158
TGFB3	-0.018434615	0.67641	0.73391
TGFBI	0.126633721	0.003997	0.007632
TGFBR1	0.137726768	0.001731	0.003528
TGFBR2	-0.252567354	6.16E-09	3.03E-08
TGFBR3	-0.387657865	6.50E-20	1.66E-18
TGFBRAP1	-0.007388371	0.867163	0.895939
TGIF1	0.248517732	1.09E-08	5.19E-08
TGIF2LX	0.121821113	0.005637	0.010491

TGIF2LY	0.154236399	0.000444	0.001005
TGIF2	0.069747662	0.1139	0.159741
TGM1	-0.240031762	3.49E-08	1.54E-07
TGM2	-0.005208457	0.906137	0.926628
TGM3	0.075126353	0.088539	0.127603
TGM4	0.170290212	0.000103	0.000262
TGM5	0.061988545	0.160117	0.21588
TGM6	0.048384191	0.273085	0.34319
TGM7	-0.046551099	0.291692	0.362944
TGOLN2	-0.16467842	0.000174	0.000426
TGS1	0.242452326	2.52E-08	1.14E-07
TG	-0.155433367	0.0004	0.000911
TH1L	0.147792669	0.000767	0.001667
THADA	-0.078898221	0.073627	0.108266
THAP10	0.023030195	0.602062	0.667296
THAP11	0.050273311	0.254774	0.323287
THAP1	0.286528895	3.45E-11	2.38E-10
THAP2	-0.06891806	0.118275	0.165054
THAP3	-0.035010613	0.427877	0.502481
THAP4	0.13056399	0.002993	0.005844
THAP5	0.120857974	0.006031	0.011154
THAP6	-0.037861872	0.391201	0.466046
THAP7	-0.068948319	0.118113	0.16484
THAP8	-0.099221825	0.024336	0.039813
THAP9	-0.037453946	0.396328	0.471199
THBD	-0.184128557	2.62E-05	7.33E-05
THBS1	0.032591451	0.460504	0.534403
THBS2	0.132631058	0.002562	0.005066
THBS3	-0.220227947	4.47E-07	1.66E-06
THBS4	-0.273812594	2.62E-10	1.58E-09
THEG	0.074390683	0.091713	0.131589
THEM4	-0.034371294	0.436367	0.510811
THEM5	0.122167408	0.005502	0.010259
THEMIS	-0.094499479	0.032023	0.051043
THG1L	-0.026166441	0.553536	0.623223
THNSL1	0.092124162	0.036618	0.057627
THNSL2	-0.128956485	0.003372	0.006522
THOC1	0.007033677	0.873485	0.901214
THOC2	-0.067428649	0.126458	0.175093
THOC3	0.204315191	2.94E-06	9.56E-06
THOC4	0.374688504	1.31E-18	2.80E-17
THOC5	0.11892651	0.006894	0.01262
THOC6	0.017103358	0.698592	0.75421
THOC7	0.186538576	2.04E-05	5.82E-05

THOP1	0.30583394	1.30E-12	1.12E-11
THPO	0.172679947	8.19E-05	0.000211
THRAP3	-0.107346815	0.014801	0.025379
THRA	-0.397553529	6.01E-21	1.80E-19
THRB	-0.257010765	3.26E-09	1.66E-08
THRSP	-0.045740129	0.300189	0.371712
THSD1P1	-0.303088312	2.10E-12	1.75E-11
THSD1	-0.320197488	9.64E-14	9.78E-13
THSD4	-0.133204621	0.002453	0.004867
THSD7A	0.01313102	0.766254	0.811953
THSD7B	-0.316289151	1.98E-13	1.93E-12
THTPA	-0.039059967	0.376375	0.451153
THUMPD1	-0.217507024	6.23E-07	2.26E-06
THUMPD2	0.104996489	0.017146	0.028963
THUMPD3	0.242290684	2.57E-08	1.16E-07
THY1	0.11796837	0.007363	0.013409
THYN1	-0.158085834	0.000316	0.000735
TH	0.162044894	0.000222	0.000532
TIA1	-0.156296962	0.000371	0.00085
TIAF1	-0.164130364	0.000183	0.000447
TIAL1	0.252364074	6.35E-09	3.12E-08
TIAM1	-0.252835589	5.93E-09	2.93E-08
TIAM2	-0.098497161	0.0254	0.041411
TICAM1	0.037218186	0.39931	0.473983
TICAM2	0.015008786	0.734011	0.784567
TIE1	-0.180038674	3.97E-05	0.000108
TIFAB	-0.163289604	0.000198	0.00048
TIFA	0.193930934	9.32E-06	2.80E-05
TIGD1	-0.081443056	0.064774	0.096533
TIGD2	-0.055267049	0.210527	0.274012
TIGD3	-0.004815217	0.913195	0.932176
TIGD4	-0.167741217	0.000131	0.000327
TIGD5	0.196733334	6.87E-06	2.11E-05
TIGD6	-0.204939216	2.74E-06	8.94E-06
TIGD7	-0.093146271	0.034576	0.05472
TIGIT	-0.084687305	0.054777	0.083078
TIMD4	-0.118716213	0.006995	0.012786
TIMELESS	0.357312974	5.92E-17	9.83E-16
TIMM10	0.300486939	3.30E-12	2.65E-11
TIMM13	0.141507465	0.001283	0.002678
TIMM16	0.169664798	0.000109	0.000276
TIMM17A	0.367307474	6.79E-18	1.31E-16
TIMM17B	0.1865566	2.04E-05	5.81E-05
TIMM22	0.197098738	6.60E-06	2.03E-05

TIMM44	0.19281502	1.05E-05	3.14E-05
TIMM50	0.256959225	3.28E-09	1.67E-08
TIMM8A	0.441180987	6.10E-26	3.90E-24
TIMM8B	0.291548695	1.51E-11	1.10E-10
TIMM9	0.466775937	3.16E-29	3.06E-27
TIMP1	0.113517677	0.009932	0.017652
TIMP2	-0.077923541	0.077269	0.113105
TIMP3	-0.161865937	0.000225	0.00054
TIMP4	0.046228724	0.29505	0.366393
TINAGL1	-0.155531889	0.000396	0.000904
TINAG	0.127470508	0.003761	0.007211
TINF2	0.121133563	0.005916	0.010957
TIPARP	-0.054741006	0.214908	0.279042
TIPIN	0.365564353	9.96E-18	1.87E-16
TIPRL	0.270695478	4.24E-10	2.49E-09
TIRAP	-0.068474465	0.120667	0.168076
TJAP1	-0.194483605	8.78E-06	2.65E-05
TJP1	-0.068998161	0.117846	0.164531
TJP2	-0.131244818	0.002844	0.005576
TJP3	-0.185198294	2.34E-05	6.61E-05
TK1	0.383606965	1.68E-19	4.12E-18
TK2	-0.30278942	2.22E-12	1.83E-11
TKTL1	0.065017017	0.140631	0.192609
TKTL2	0.079125012	0.0728	0.107168
TKT	0.088808381	0.04396	0.068052
TLCD1	0.163982183	0.000186	0.000452
TLE1	0.05735143	0.1938	0.254864
TLE2	-0.316075125	2.06E-13	2.00E-12
TLE3	-0.083228512	0.059101	0.0889
TLE4	-0.39097468	2.95E-20	8.01E-19
TLE6	-0.132062716	0.002675	0.005273
TLK1	0.282134539	7.03E-11	4.62E-10
TLK2	0.10117613	0.021655	0.035846
TLL1	-0.156599623	0.000361	0.000829
TLL2	0.183759348	2.72E-05	7.59E-05
TLN1	-0.189035566	1.57E-05	4.57E-05
TLN2	-0.146763698	0.000836	0.001802
TLR10	-0.237827831	4.69E-08	2.03E-07
TLR1	-0.04698467	0.287216	0.3582
TLR2	-0.446559609	1.31E-26	9.16E-25
TLR3	-0.215249606	8.19E-07	2.91E-06
TLR4	-0.137611032	0.001747	0.003556
TLR5	-0.433413408	5.35E-25	3.00E-23
TLR6	0.080511498	0.067907	0.100745

TLR7	-0.270149033	4.61E-10	2.69E-09
TLR8	-0.131883911	0.002711	0.00534
TLR9	-0.075170608	0.088351	0.127378
TLX1NB	0.028688951	0.515946	0.588156
TLX1	0.054731631	0.214987	0.279108
TLX2	0.027471629	0.53392	0.60516
TLX3	0.087550882	0.047052	0.072372
TM2D1	0.151938883	0.000541	0.001205
TM2D2	0.123186896	0.005119	0.009604
TM2D3	-0.059073416	0.180733	0.239985
TM4SF18	-0.021705436	0.623118	0.685993
TM4SF19	0.219291425	5.02E-07	1.85E-06
TM4SF1	0.014677002	0.739676	0.789268
TM4SF20	0.209441908	1.63E-06	5.52E-06
TM4SF4	-0.084353808	0.055741	0.084347
TM4SF5	0.094220793	0.032535	0.051801
TM6SF1	-0.23021025	1.27E-07	5.16E-07
TM6SF2	0.028775527	0.514679	0.587081
TM7SF2	-0.101915547	0.02071	0.034412
TM7SF3	0.040186241	0.362758	0.437349
TM7SF4	-0.205226345	2.65E-06	8.68E-06
TM9SF1	0.255017596	4.34E-09	2.18E-08
TM9SF2	-0.000482956	0.991277	0.993568
TM9SF3	0.191126392	1.26E-05	3.71E-05
TM9SF4	0.101248355	0.021561	0.035703
TMBIM1	-0.07905758	0.073045	0.107489
TMBIM4	-0.075794723	0.085733	0.123987
TMBIM6	0.149990613	0.000638	0.001405
TMC1	0.071393609	0.105596	0.149174
TMC2	-0.177178627	5.28E-05	0.00014
TMC3	-0.049995148	0.257415	0.326125
TMC4	-0.314071643	2.98E-13	2.82E-12
TMC5	-0.119490797	0.006631	0.012174
TMC6	-0.252232666	6.46E-09	3.17E-08
TMC7	0.076322147	0.083569	0.121183
TMC8	-0.33500048	5.69E-15	7.01E-14
TMCC1	-0.005811985	0.89532	0.918103
TMCC2	-0.27547492	2.02E-10	1.24E-09
TMCC3	0.004223669	0.923824	0.940433
TMCO1	0.162801264	0.000207	0.0005
TMCO2	-0.121694866	0.005688	0.010573
TMCO3	0.037167683	0.39995	0.47456
TMCO4	-0.188897389	1.59E-05	4.63E-05
TMCO5A	-0.081700322	0.06393	0.095425

TMCO6	-0.090854513	0.039296	0.061431
TMCO7	-0.035948392	0.415599	0.490349
TMED10P1	0.277536936	1.46E-10	9.18E-10
TMED10	0.260598699	1.93E-09	1.02E-08
TMED1	0.110638501	0.011991	0.020913
TMED2	0.472328689	5.61E-30	6.23E-28
TMED3	0.117447219	0.007629	0.013849
TMED4	0.002242234	0.959516	0.968929
TMED5	0.169265495	0.000113	0.000286
TMED6	-0.020722258	0.638948	0.700103
TMED7-TICAM2	0.174894368	6.60E-05	0.000173
TMED7	0.236819581	5.36E-08	2.29E-07
TMED8	0.022369739	0.61252	0.676559
TMED9	0.033287749	0.450974	0.525356
TMEFF1	0.291205844	1.59E-11	1.16E-10
TMEFF2	-0.208716785	1.77E-06	5.98E-06
TMEM100	-0.267091366	7.33E-10	4.16E-09
TMEM101	-0.006736359	0.878791	0.905219
TMEM102	-0.078961252	0.073396	0.107974
TMEM104	-0.084810013	0.054425	0.082626
TMEM105	-0.134915338	0.002153	0.004319
TMEM106A	-0.110659237	0.011975	0.02089
TMEM106B	0.212629366	1.12E-06	3.89E-06
TMEM106C	0.309852717	6.37E-13	5.75E-12
TMEM107	0.022578525	0.609205	0.673664
TMEM108	-0.291615673	1.49E-11	1.09E-10
TMEM109	-0.231369803	1.10E-07	4.49E-07
TMEM110	-0.203376989	3.27E-06	1.06E-05
TMEM111	-0.052254856	0.236501	0.30316
TMEM114	-0.124115403	0.004792	0.009036
TMEM115	-0.163917921	0.000187	0.000454
TMEM116	-0.139085992	0.001556	0.003195
TMEM117	0.251914374	6.76E-09	3.31E-08
TMEM119	-0.261023443	1.81E-09	9.65E-09
TMEM11	0.146008524	0.00089	0.001908
TMEM120A	-0.031193308	0.479976	0.553849
TMEM120B	0.000625264	0.988706	0.991633
TMEM121	-0.039228533	0.374317	0.449222
TMEM123	0.117343787	0.007683	0.013936
TMEM125	-0.366065225	8.93E-18	1.69E-16
TMEM126A	0.267926467	6.46E-10	3.70E-09
TMEM126B	0.135912663	0.001993	0.004023
TMEM127	-0.03571576	0.418625	0.493364
TMEM128	-0.07514802	0.088447	0.127493

TMEM129	-0.225453443	2.33E-07	9.06E-07
TMEM130	-0.420116675	1.94E-23	8.48E-22
TMEM131	-0.042287225	0.338191	0.412281
TMEM132A	0.166907885	0.000142	0.000352
TMEM132B	-0.116408656	0.008186	0.014776
TMEM132C	-0.371519258	2.67E-18	5.40E-17
TMEM132D	-0.269684086	4.94E-10	2.87E-09
TMEM132E	-0.359157815	3.99E-17	6.79E-16
TMEM133	-0.207641784	2.01E-06	6.70E-06
TMEM134	-0.087304303	0.047679	0.073201
TMEM135	0.211503307	1.28E-06	4.41E-06
TMEM136	-0.156201119	0.000374	0.000856
TMEM138	-0.057407875	0.193361	0.254438
TMEM139	-0.075122414	0.088556	0.127618
TMEM140	-0.052666824	0.232821	0.299018
TMEM141	0.190248379	1.38E-05	4.06E-05
TMEM143	-0.274218416	2.46E-10	1.49E-09
TMEM144	-0.016250812	0.712935	0.766507
TMEM145	0.076343411	0.083483	0.121066
TMEM146	-0.206712528	2.24E-06	7.40E-06
TMEM147	0.202753472	3.51E-06	1.13E-05
TMEM149	-0.139955949	0.001452	0.002999
TMEM14A	0.277954581	1.37E-10	8.64E-10
TMEM14B	0.292671547	1.25E-11	9.21E-11
TMEM14C	0.210344105	1.47E-06	5.00E-06
TMEM14E	-0.125022724	0.00449	0.008504
TMEM150A	-0.243004349	2.33E-08	1.06E-07
TMEM150B	-0.061311677	0.164739	0.221305
TMEM150C	-0.06105806	0.166496	0.223395
TMEM151A	-0.066343002	0.132694	0.18293
TMEM151B	-0.310913733	5.27E-13	4.80E-12
TMEM154	-0.208178064	1.89E-06	6.34E-06
TMEM155	-0.135802794	0.00201	0.004055
TMEM156	0.126662203	0.003989	0.00762
TMEM158	0.245028963	1.77E-08	8.15E-08
TMEM159	-0.202455228	3.63E-06	1.16E-05
TMEM160	-0.040673567	0.356962	0.431507
TMEM161A	-0.012624208	0.775028	0.819167
TMEM161B	-0.065286957	0.138987	0.190644
TMEM163	-0.346905965	5.21E-16	7.55E-15
TMEM164	-0.175736935	6.08E-05	0.00016
TMEM165	0.167216099	0.000138	0.000342
TMEM167A	0.1188049	0.006952	0.012711
TMEM167B	-0.123032658	0.005176	0.009698

TMEM168	-0.020045618	0.649938	0.709811
TMEM169	-0.128015707	0.003614	0.006948
TMEM170A	-0.027713328	0.530326	0.60194
TMEM170B	-0.199304086	5.17E-06	1.62E-05
TMEM171	0.158860477	0.000295	0.000691
TMEM173	-0.449962665	4.88E-27	3.59E-25
TMEM174	0.01019281	0.817505	0.854392
TMEM175	-0.411152143	2.00E-22	7.39E-21
TMEM176A	0.030788903	0.485691	0.559184
TMEM176B	0.021909876	0.619848	0.683032
TMEM177	0.231218263	1.12E-07	4.57E-07
TMEM178	-0.213428066	1.02E-06	3.57E-06
TMEM179B	0.061417679	0.164008	0.220472
TMEM179	-0.086246602	0.050449	0.077152
TMEM17	0.067334052	0.126992	0.175796
TMEM180	-0.047023133	0.286821	0.357742
TMEM181	-0.095500811	0.030239	0.048528
TMEM182	0.259619727	2.23E-09	1.17E-08
TMEM183A	0.274511315	2.35E-10	1.43E-09
TMEM184A	-0.030160507	0.494643	0.567917
TMEM184B	0.036872969	0.4037	0.478399
TMEM184C	0.111272324	0.011508	0.020168
TMEM185A	-0.105907921	0.016201	0.027503
TMEM185B	0.318722327	1.27E-13	1.27E-12
TMEM186	0.079470678	0.071554	0.105606
TMEM187	-0.047297136	0.284019	0.354855
TMEM188	0.032358637	0.463716	0.537596
TMEM189-UBE2V1	0.113896393	0.009685	0.017251
TMEM189	0.199547119	5.03E-06	1.58E-05
TMEM18	0.09996726	0.023281	0.038271
TMEM190	-0.214579858	8.87E-07	3.14E-06
TMEM191A	-0.129529532	0.003232	0.006274
TMEM192	0.016903873	0.701938	0.757374
TMEM194A	0.208856026	1.74E-06	5.89E-06
TMEM194B	0.209367436	1.64E-06	5.56E-06
TMEM195	0.109779378	0.012675	0.021998
TMEM196	-0.041968306	0.34185	0.416031
TMEM198	-0.108622876	0.01365	0.023552
TMEM199	0.197049407	6.63E-06	2.04E-05
TMEM19	0.092086282	0.036696	0.05774
TMEM200A	-0.041835475	0.343381	0.417488
TMEM200B	0.017641854	0.689587	0.74598
TMEM200C	0.02704016	0.540366	0.611218
TMEM201	-0.033502729	0.448054	0.52232

TMEM202	0.08053871	0.067814	0.100621
TMEM203	0.13584168	0.002004	0.004044
TMEM204	-0.261357298	1.73E-09	9.21E-09
TMEM205	-0.079641475	0.070945	0.104769
TMEM206	0.379102793	4.78E-19	1.09E-17
TMEM207	0.015732538	0.721704	0.773896
TMEM208	0.115916037	0.008462	0.015218
TMEM209	0.161910684	0.000224	0.000538
TMEM20	0.00323992	0.94153	0.953697
TMEM211	-0.083789525	0.057406	0.086598
TMEM212	-0.212995743	1.07E-06	3.74E-06
TMEM213	-0.212105569	1.19E-06	4.12E-06
TMEM214	0.032305009	0.464457	0.538334
TMEM215	-0.017863425	0.685894	0.742668
TMEM216	0.157622003	0.00033	0.000763
TMEM217	-0.183013598	2.93E-05	8.15E-05
TMEM218	-0.024973659	0.571765	0.6399
TMEM219	-0.120408997	0.006222	0.011479
TMEM220	-0.300541455	3.27E-12	2.63E-11
TMEM222	-0.200075178	4.75E-06	1.49E-05
TMEM223	0.27397728	2.55E-10	1.55E-09
TMEM229A	0.021284539	0.629874	0.691906
TMEM229B	-0.122760271	0.005276	0.009872
TMEM22	0.23760457	4.83E-08	2.08E-07
TMEM231	-0.19945257	5.09E-06	1.59E-05
TMEM232	-0.283174531	5.95E-11	3.95E-10
TMEM233	-0.08766933	0.046753	0.071968
TMEM25	-0.275329221	2.07E-10	1.27E-09
TMEM26	0.072418333	0.100674	0.14313
TMEM27	-0.224748865	2.55E-07	9.84E-07
TMEM2	0.053542151	0.225137	0.290381
TMEM30A	-0.000129027	0.997669	0.998519
TMEM30B	-0.07793058	0.077242	0.113091
TMEM30C	0.02809237	0.524715	0.596689
TMEM31	-0.010914736	0.804831	0.843839
TMEM33	0.301905906	2.58E-12	2.11E-11
TMEM35	-0.090253713	0.04062	0.063357
TMEM37	-0.289662124	2.06E-11	1.47E-10
TMEM38A	-0.050216744	0.255309	0.323802
TMEM38B	0.299905523	3.65E-12	2.91E-11
TMEM39A	0.203980934	3.06E-06	9.90E-06
TMEM39B	-0.169636882	0.00011	0.000277
TMEM40	0.002542418	0.954102	0.964623
TMEM41A	0.078883351	0.073681	0.108338

TMEM41B	-0.05510119	0.211901	0.275568
TMEM42	-0.057429146	0.193196	0.25427
TMEM43	-0.218984975	5.21E-07	1.92E-06
TMEM44	-0.03625076	0.411685	0.486391
TMEM45A	0.167324294	0.000136	0.000339
TMEM45B	0.02866378	0.516314	0.588542
TMEM47	-0.13112243	0.00287	0.00562
TMEM48	0.432575036	6.74E-25	3.70E-23
TMEM49	0.077987227	0.077027	0.112798
TMEM50A	-0.059804414	0.175388	0.23396
TMEM50B	-0.210761527	1.40E-06	4.78E-06
TMEM51	-0.044714115	0.311172	0.383152
TMEM52	0.045495189	0.302787	0.374466
TMEM53	-0.074513175	0.091179	0.130982
TMEM54	-0.06405751	0.146598	0.19974
TMEM55A	0.139464621	0.00151	0.003111
TMEM55B	0.098398816	0.025548	0.041634
TMEM56	-0.161626303	0.00023	0.000551
TMEM57	-0.227438774	1.81E-07	7.17E-07
TMEM59L	-0.100371752	0.022726	0.037429
TMEM59	-0.13200193	0.002687	0.005297
TMEM5	0.316004696	2.09E-13	2.02E-12
TMEM60	0.049699506	0.260243	0.329074
TMEM61	-0.038058778	0.38874	0.463584
TMEM62	0.055372333	0.209658	0.273059
TMEM63A	-0.360140927	3.23E-17	5.61E-16
TMEM63B	-0.291019983	1.64E-11	1.19E-10
TMEM63C	-0.22010717	4.54E-07	1.69E-06
TMEM64	0.014715944	0.73901	0.788758
TMEM65	0.327690779	2.35E-14	2.62E-13
TMEM66	-0.135672103	0.002031	0.004093
TMEM67	-0.020371017	0.644643	0.705184
TMEM68	0.209409152	1.64E-06	5.54E-06
TMEM69	0.205801441	2.48E-06	8.17E-06
TMEM70	0.317260905	1.66E-13	1.63E-12
TMEM71	-0.120973559	0.005982	0.011072
TMEM72	-0.028289723	0.521806	0.593786
TMEM74	0.154232392	0.000444	0.001005
TMEM79	0.065873241	0.135466	0.186339
TMEM80	-0.26860154	5.83E-10	3.36E-09
TMEM81	0.013854516	0.753779	0.801326
TMEM82	0.105384882	0.016738	0.028333
TMEM84	-0.006971218	0.874599	0.90213
TMEM85	0.260324746	2.01E-09	1.06E-08

TMEM86A	-0.160094689	0.000264	0.000624
TMEM86B	-0.193654387	9.60E-06	2.88E-05
TMEM87A	-0.149020446	0.000692	0.001517
TMEM87B	0.106142233	0.015965	0.027138
TMEM88B	-0.078059235	0.076753	0.112474
TMEM88	-0.276091074	1.83E-10	1.14E-09
TMEM89	-0.100170761	0.023	0.037853
TMEM8A	-0.048563625	0.271308	0.341256
TMEM8B	-0.465472963	4.71E-29	4.46E-27
TMEM8C	-0.099895424	0.023381	0.038407
TMEM90A	-0.373373366	1.76E-18	3.66E-17
TMEM90B	0.012378814	0.779287	0.822451
TMEM91	-0.345630607	6.76E-16	9.57E-15
TMEM92	-0.072161553	0.10189	0.144581
TMEM93	0.284893081	4.50E-11	3.05E-10
TMEM95	0.04631383	0.294161	0.365516
TMEM97	0.04335318	0.326144	0.399404
TMEM98	-0.288556962	2.47E-11	1.74E-10
TMEM99	-0.006664982	0.880065	0.906113
TMEM9B	-0.139101624	0.001554	0.003192
TMEM9	0.026586209	0.547189	0.617573
TMF1	-0.017318703	0.694986	0.750926
TMIE	0.020373378	0.644605	0.705181
TMIGD1	0.089972865	0.041252	0.064277
TMIGD2	-0.009846734	0.823597	0.859593
TMLHE	0.054739177	0.214923	0.279044
TMOD1	-0.165105207	0.000167	0.000411
TMOD2	-0.260901999	1.85E-09	9.82E-09
TMOD3	0.157575257	0.000331	0.000766
TMOD4	-0.147338738	0.000797	0.001725
TMPO	0.440738409	6.91E-26	4.40E-24
TMPPE	-0.01858884	0.673858	0.731697
TMPRSS11A	0.040665565	0.357057	0.43154
TMPRSS11BNL	-0.012237585	0.781741	0.824562
TMPRSS11B	-0.021306542	0.62952	0.691653
TMPRSS11D	0.01996024	0.651331	0.711138
TMPRSS11F	0.076919501	0.081172	0.118144
TMPRSS12	-0.024714744	0.57576	0.643388
TMPRSS13	-0.057590855	0.191943	0.252838
TMPRSS15	0.081639627	0.064128	0.095699
TMPRSS2	-0.480673348	3.94E-31	5.26E-29
TMPRSS3	-0.171840388	8.88E-05	0.000228
TMPRSS4	-0.096198867	0.029046	0.046756
TMPRSS5	-0.229581016	1.38E-07	5.56E-07

TMPRSS6	-0.082398264	0.061686	0.092447
TMPRSS7	-0.16767901	0.000132	0.000329
TMPRSS9	-0.077220545	0.079986	0.116654
TMSB10	0.286375373	3.54E-11	2.43E-10
TMSB15A	0.010105951	0.819033	0.855633
TMSB15B	0.015618207	0.723643	0.775684
TMSB4Y	0.133671094	0.002368	0.004713
TMSL3	-0.133368123	0.002423	0.004812
TMTC1	0.009724944	0.825744	0.861294
TMTC2	-0.159176192	0.000287	0.000674
TMTC3	0.378703953	5.23E-19	1.19E-17
TMTC4	0.033604978	0.446669	0.521035
TMUB1	0.037610916	0.39435	0.469294
TMUB2	-0.169018755	0.000116	0.000292
TMX1	0.438270432	1.38E-25	8.52E-24
TMX2	0.261463978	1.70E-09	9.08E-09
TMX3	-0.017724048	0.688216	0.744819
TMX4	-0.077281926	0.079745	0.11633
TNC	-0.015299486	0.729059	0.780485
TNFAIP1	0.109116025	0.013226	0.022881
TNFAIP2	-0.096215543	0.029018	0.046715
TNFAIP3	-0.077096751	0.080472	0.11727
TNFAIP6	0.225202253	2.41E-07	9.33E-07
TNFAIP8L1	0.063732046	0.148665	0.202198
TNFAIP8L2	-0.227832941	1.73E-07	6.84E-07
TNFAIP8L3	-0.087838199	0.04633	0.071388
TNFAIP8	-0.111309206	0.01148	0.020128
TNFRSF10A	0.088236284	0.045345	0.069994
TNFRSF10B	-0.029522879	0.503815	0.576433
TNFRSF10C	-0.208151605	1.89E-06	6.36E-06
TNFRSF10D	-0.009392682	0.831607	0.865918
TNFRSF11A	-0.104388846	0.017803	0.029974
TNFRSF11B	-0.021370832	0.628487	0.690989
TNFRSF12A	0.035225502	0.425045	0.499665
TNFRSF13B	-0.311837333	4.46E-13	4.11E-12
TNFRSF13C	-0.155589479	0.000394	0.0009
TNFRSF14	-0.402241897	1.89E-21	6.22E-20
TNFRSF17	-0.02734533	0.535803	0.606847
TNFRSF18	0.01612774	0.715014	0.76804
TNFRSF19	-0.200766186	4.39E-06	1.39E-05
TNFRSF1A	0.193393997	9.87E-06	2.96E-05
TNFRSF1B	-0.241600262	2.83E-08	1.26E-07
TNFRSF21	0.123878426	0.004874	0.009177
TNFRSF25	-0.132134924	0.00266	0.005247

TNFRSF4	-0.126633415	0.003997	0.007632
TNFRSF6B	0.043878817	0.320307	0.393044
TNFRSF8	-0.02752879	0.533069	0.604435
TNFRSF9	0.062795293	0.154738	0.209404
TNFSF10	-0.231208793	1.12E-07	4.57E-07
TNFSF11	0.194232719	9.02E-06	2.72E-05
TNFSF12-TNFSF13	-0.097514575	0.026908	0.043658
TNFSF12	-0.379585797	4.27E-19	9.85E-18
TNFSF13B	-0.061257975	0.165109	0.221758
TNFSF13	-0.334453645	6.33E-15	7.76E-14
TNFSF14	-0.1703733	0.000102	0.00026
TNFSF15	-0.167165333	0.000138	0.000344
TNFSF18	-0.059243188	0.179481	0.238655
TNFSF4	0.167425274	0.000135	0.000336
TNFSF8	-0.160261989	0.00026	0.000616
TNFSF9	0.09088551	0.039229	0.061345
TNF	-0.136826364	0.001857	0.003764
TNIK	-0.306955607	1.07E-12	9.30E-12
TNIP1	-0.151640982	0.000555	0.001234
TNIP2	0.100749055	0.022218	0.036689
TNIP3	0.049068767	0.266348	0.335852
TNK1	-0.107105119	0.015028	0.025729
TNK2	-0.313242054	3.46E-13	3.25E-12
TNKS1BP1	-0.278609887	1.23E-10	7.84E-10
TNKS2	-0.046138633	0.295993	0.367313
TNKS	-0.113822892	0.009733	0.017326
TNMD	0.009423232	0.831068	0.865475
TNNC1	-0.349125409	3.30E-16	4.90E-15
TNNC2	-0.078640822	0.074575	0.109563
TNNI1	-0.175784151	6.05E-05	0.000159
TNNI2	-0.293580474	1.07E-11	7.98E-11
TNNI3K	-0.34105867	1.71E-15	2.29E-14
TNNI3	0.07452134	0.091143	0.13094
TNNT1	0.166977811	0.000141	0.00035
TNNT2	-0.258375602	2.67E-09	1.38E-08
TNNT3	-0.257400136	3.08E-09	1.58E-08
TNN	-0.304008876	1.79E-12	1.51E-11
TNP1	0.017495187	0.692035	0.748102
TNP2	-0.021970441	0.618881	0.682192
TNPO1	0.286796108	3.30E-11	2.28E-10
TNPO2	-0.001610978	0.970908	0.978101
TNPO3	0.186876061	1.97E-05	5.64E-05
TNRC18	-0.188891828	1.60E-05	4.63E-05
TNRC6A	-0.273889298	2.59E-10	1.57E-09

TNRC6B	-0.267996947	6.39E-10	3.66E-09
TNRC6C	-0.369259488	4.41E-18	8.70E-17
TNR	-0.221428265	3.86E-07	1.45E-06
TNS1	-0.480667058	3.95E-31	5.26E-29
TNS3	-0.082600836	0.061047	0.091613
TNS4	0.144745753	0.000987	0.002101
TNXB	-0.465604261	4.53E-29	4.33E-27
TOB1	-0.147048382	0.000816	0.001764
TOB2	-0.133587084	0.002383	0.004741
TOE1	-0.08982282	0.041593	0.064753
TOLLIP	-0.280586318	9.01E-11	5.84E-10
TOM1L1	0.053576377	0.22484	0.290073
TOM1L2	-0.453163718	1.91E-27	1.48E-25
TOM1	-0.200132312	4.72E-06	1.48E-05
TOMM20L	0.139288289	0.001531	0.00315
TOMM20	0.207630043	2.01E-06	6.71E-06
TOMM22	0.29062995	1.75E-11	1.27E-10
TOMM34	0.179271987	4.28E-05	0.000116
TOMM40L	0.137602537	0.001748	0.003558
TOMM40	0.3648283	1.17E-17	2.16E-16
TOMM5	0.421286887	1.42E-23	6.35E-22
TOMM6	0.110927509	0.011769	0.020562
TOMM70A	0.356092912	7.67E-17	1.25E-15
TOMM7	0.000233726	0.995778	0.997175
TOP1MT	0.054974585	0.212954	0.276812
TOP1P1	0.074414003	0.091611	0.131471
TOP1P2	0.112465286	0.010645	0.018786
TOP1	0.159535451	0.000278	0.000654
TOP2A	0.473148283	4.34E-30	4.90E-28
TOP2B	-0.12384211	0.004886	0.009197
TOP3A	0.071792949	0.103656	0.146734
TOP3B	-0.127418074	0.003775	0.007237
TOPBP1	0.282203887	6.95E-11	4.58E-10
TOPORS	0.009472951	0.83019	0.864983
TOR1AIP1	-0.125259053	0.004415	0.008368
TOR1AIP2	0.056722881	0.198738	0.260518
TOR1A	0.182700632	3.03E-05	8.39E-05
TOR1B	0.09457297	0.031889	0.050858
TOR2A	-0.130868633	0.002925	0.005725
TOR3A	0.063597154	0.149528	0.203262
TOX2	-0.217000876	6.63E-07	2.40E-06
TOX3	-0.146616356	0.000846	0.001823
TOX4	0.125386167	0.004375	0.008299
TOX	-0.301268482	2.89E-12	2.34E-11

TP53AIP1	-0.123837882	0.004888	0.009199
TP53BP1	-0.023644119	0.592414	0.658318
TP53BP2	-0.005489579	0.901096	0.922671
TP53I11	-0.181719983	3.35E-05	9.20E-05
TP53I13	-0.115698628	0.008587	0.015416
TP53I3	0.148088853	0.000749	0.001631
TP53INP1	-0.253903775	5.10E-09	2.54E-08
TP53INP2	0.05916894	0.180028	0.239255
TP53RK	0.218913033	5.25E-07	1.93E-06
TP53TG1	-0.131462182	0.002798	0.005493
TP53TG3B	0.052586628	0.233534	0.299876
TP53TG5	-0.051402739	0.244242	0.311644
TP53	-0.101975305	0.020635	0.034296
TP63	-0.199369013	5.13E-06	1.61E-05
TP73	-0.226403812	2.07E-07	8.11E-07
TPBG	0.070768477	0.108692	0.153157
TPCN1	-0.126654245	0.003991	0.007623
TPCN2	-0.007317406	0.868427	0.897199
TPD52L1	0.080363142	0.068418	0.101434
TPD52L2	0.270577695	4.31E-10	2.53E-09
TPD52L3	-0.038856635	0.378866	0.453661
TPD52	0.272360209	3.28E-10	1.95E-09
TPH1	-0.071469812	0.105224	0.148721
TPH2	0.06800389	0.123247	0.171263
TPI1P2	0.116453732	0.008161	0.014735
TPI1P3	0.159510117	0.000279	0.000655
TPI1	0.454455976	1.30E-27	1.03E-25
TPK1	-0.186749507	2.00E-05	5.71E-05
TPM1	-0.041636327	0.345685	0.419932
TPM2	0.098920739	0.024773	0.040474
TPM3	0.372210427	2.28E-18	4.68E-17
TPM4	0.166721497	0.000144	0.000357
TPMT	0.137773869	0.001725	0.003516
TPO	-0.046469142	0.292543	0.3638
TPP1	-0.250802572	7.91E-09	3.85E-08
TPP2	-0.151017112	0.000585	0.001295
TPPP2	-0.184983155	2.40E-05	6.75E-05
TPPP3	-0.33579399	4.86E-15	6.05E-14
TPPP	-0.383772879	1.62E-19	3.99E-18
TPRA1	-0.137984689	0.001696	0.003465
TPRG1L	-0.259937531	2.13E-09	1.12E-08
TPRG1	-0.217770966	6.04E-07	2.20E-06
TPRKB	0.381204699	2.94E-19	6.97E-18
TPRN	0.036775111	0.40495	0.479709

TPRX1	0.005401739	0.902671	0.92384
TPRXL	-0.008815362	0.841817	0.874772
TPR	-0.064754164	0.142247	0.194595
TPSAB1	-0.344833018	7.96E-16	1.11E-14
TPSB2	-0.351816775	1.89E-16	2.88E-15
TPSD1	-0.174937655	6.58E-05	0.000172
TPSG1	-0.167685739	0.000132	0.000329
TPST1	-0.023884644	0.588653	0.654914
TPST2	-0.00903516	0.837927	0.871115
TPT1	0.041431726	0.348063	0.422486
TPTE2P1	-0.118624649	0.007039	0.012859
TPTE2P3	-0.085721865	0.051872	0.079074
TPTE2	0.005850111	0.894637	0.917591
TPTE	0.106031523	0.016076	0.027301
TPX2	0.513965018	4.61E-36	1.56E-33
TRA2A	-0.048706498	0.269899	0.339698
TRA2B	0.255169226	4.25E-09	2.14E-08
TRABD	-0.113153661	0.010173	0.018036
TRADD	-0.224410957	2.66E-07	1.02E-06
TRAF1	-0.230761272	1.19E-07	4.83E-07
TRAF2	0.102716291	0.019727	0.032921
TRAF3IP1	-0.046102574	0.296371	0.367714
TRAF3IP2	-0.116025989	0.0084	0.015122
TRAF3IP3	-0.284186432	5.05E-11	3.39E-10
TRAF3	0.074673292	0.090483	0.130058
TRAF4	0.109188653	0.013165	0.022786
TRAF5	-0.126747673	0.003964	0.007577
TRAF6	-0.151940529	0.000541	0.001205
TRAF7	0.138704046	0.001603	0.003284
TRAFD1	-0.163489874	0.000194	0.000471
TRAIP	0.36723104	6.91E-18	1.32E-16
TRAK1	-0.173751175	7.38E-05	0.000192
TRAK2	-0.27039997	4.43E-10	2.59E-09
TRAM1L1	-0.140065456	0.00144	0.002976
TRAM1	0.004185186	0.924516	0.941089
TRAM2	0.036910127	0.403226	0.477866
TRANK1	-0.414539265	8.34E-23	3.30E-21
TRAP1	0.204102963	3.01E-06	9.77E-06
TRAPPC10	-0.213610746	9.96E-07	3.50E-06
TRAPPC1	-0.03875497	0.380116	0.454819
TRAPPC2L	0.089231574	0.042959	0.066641
TRAPPC2P1	0.046089142	0.296512	0.367843
TRAPPC2	-0.158055717	0.000317	0.000737
TRAPPC3	0.01198217	0.786185	0.828156

TRAPPC4	0.098849191	0.024878	0.040633
TRAPPC5	0.026831923	0.543491	0.614231
TRAPPC6A	-0.184907891	2.42E-05	6.80E-05
TRAPPC6B	0.138444397	0.001636	0.003348
TRAPPC9	0.051193241	0.246172	0.313847
TRAT1	-0.144180584	0.001034	0.002193
TRDMT1	-0.144344419	0.00102	0.002168
TRDN	0.131168501	0.00286	0.005604
TREH	-0.095643211	0.029992	0.048175
TREM1	0.064383421	0.14455	0.197394
TREM2	-0.230888399	1.17E-07	4.75E-07
TREML1	-0.391037313	2.91E-20	7.91E-19
TREML2P1	0.006608692	0.88107	0.906747
TREML2	-0.231146299	1.13E-07	4.61E-07
TREML3	0.244138614	2.00E-08	9.16E-08
TREML4	0.009074784	0.837226	0.87068
TRERF1	0.077170373	0.080182	0.116916
TREX1	-0.276370456	1.76E-10	1.09E-09
TREX2	-0.225633064	2.28E-07	8.87E-07
TRHDE	-0.148790536	0.000706	0.001544
TRHR	0.003819701	0.931091	0.945999
TRH	-0.073804586	0.094307	0.134913
TRIAP1	0.368859823	4.82E-18	9.44E-17
TRIB1	-0.079335466	0.072039	0.106245
TRIB2	-0.182402754	3.12E-05	8.62E-05
TRIB3	0.304218458	1.73E-12	1.46E-11
TRIL	-0.205170708	2.67E-06	8.73E-06
TRIM10	0.215254777	8.18E-07	2.91E-06
TRIM11	0.041913445	0.342482	0.416584
TRIM13	-0.17492407	6.58E-05	0.000172
TRIM14	-0.202605575	3.57E-06	1.15E-05
TRIM15	0.27735242	1.50E-10	9.43E-10
TRIM16L	0.26284235	1.39E-09	7.51E-09
TRIM16	0.268750231	5.70E-10	3.28E-09
TRIM17	-0.124975144	0.004506	0.008529
TRIM21	-0.041952581	0.342031	0.416201
TRIM22	-0.389732112	3.97E-20	1.06E-18
TRIM23	-0.199811817	4.89E-06	1.53E-05
TRIM24	-0.077926957	0.077256	0.113097
TRIM25	-0.008337121	0.850294	0.881793
TRIM26	-0.185431807	2.29E-05	6.47E-05
TRIM27	0.121942545	0.005589	0.01041
TRIM28	0.177501538	5.11E-05	0.000136
TRIM29	-0.062201847	0.158682	0.214176

TRIM2	-0.220948721	4.09E-07	1.53E-06
TRIM31	0.142892368	0.001148	0.002416
TRIM32	-0.008173952	0.853191	0.884017
TRIM33	-0.048837935	0.268607	0.338285
TRIM34	-0.13465627	0.002196	0.004398
TRIM35	-0.225932289	2.20E-07	8.56E-07
TRIM36	-0.085127511	0.053525	0.081358
TRIM37	0.182118181	3.21E-05	8.85E-05
TRIM38	-0.306843964	1.09E-12	9.48E-12
TRIM39	-0.249011894	1.02E-08	4.87E-08
TRIM3	-0.226730042	1.99E-07	7.80E-07
TRIM40	0.236925413	5.29E-08	2.27E-07
TRIM41	-0.328575059	1.98E-14	2.24E-13
TRIM42	0.010800466	0.806833	0.845496
TRIM43	-0.005451921	0.901771	0.923202
TRIM44	-0.019732301	0.655054	0.714615
TRIM45	-0.210221014	1.49E-06	5.07E-06
TRIM46	-0.054030403	0.22093	0.285692
TRIM47	-0.065718163	0.13639	0.187456
TRIM48	-0.022193328	0.615327	0.679023
TRIM49L	0.017440651	0.692946	0.748925
TRIM49	-0.09701804	0.027698	0.044807
TRIM4	-0.127946592	0.003632	0.00698
TRIM50	-0.284751459	4.61E-11	3.11E-10
TRIM52	-0.404768738	1.01E-21	3.43E-20
TRIM53	-0.163032185	0.000203	0.00049
TRIM54	-0.103550032	0.018746	0.031424
TRIM55	-0.0596746	0.176328	0.235105
TRIM56	-0.113418015	0.009997	0.017753
TRIM58	-0.071490914	0.105121	0.148598
TRIM59	0.320895654	8.47E-14	8.64E-13
TRIM5	-0.172452584	8.37E-05	0.000215
TRIM6-TRIM34	0.059194934	0.179836	0.239048
TRIM60	-0.015344318	0.728297	0.779794
TRIM61	-0.120870982	0.006025	0.011147
TRIM62	-0.297503066	5.51E-12	4.28E-11
TRIM63	-0.315292991	2.38E-13	2.29E-12
TRIM64	0.096850738	0.027969	0.045212
TRIM65	-0.038180501	0.387224	0.462079
TRIM66	-0.389315119	4.39E-20	1.16E-18
TRIM67	-0.201309579	4.13E-06	1.31E-05
TRIM68	-0.148361159	0.000732	0.001597
TRIM69	0.060456985	0.170716	0.228383
TRIM6	0.149163233	0.000684	0.001501

TRIM71	-0.274593723	2.32E-10	1.42E-09
TRIM72	0.045560846	0.302089	0.373764
TRIM74	-0.223790379	2.87E-07	1.10E-06
TRIM77	-0.012556216	0.776208	0.819893
TRIM78P	-0.265076165	9.93E-10	5.53E-09
TRIM7	0.018037677	0.682996	0.73985
TRIM8	-0.144953457	0.00097	0.002068
TRIM9	0.143574441	0.001086	0.002296
TRIML1	-0.016424945	0.709997	0.764171
TRIML2	0.148256943	0.000738	0.001609
TRIOBP	-0.288606904	2.45E-11	1.73E-10
TRIO	-0.023225874	0.598979	0.664395
TRIP10	-0.057419802	0.193269	0.254332
TRIP11	0.013404503	0.761531	0.807977
TRIP12	0.127161034	0.003847	0.007363
TRIP13	0.447073855	1.13E-26	7.98E-25
TRIP4	0.154428738	0.000436	0.00099
TRIP6	-0.166571488	0.000146	0.000362
TRIT1	0.163364378	0.000197	0.000476
TRMT112	0.173654028	7.45E-05	0.000193
TRMT11	0.020223682	0.647039	0.70734
TRMT12	0.298730746	4.47E-12	3.50E-11
TRMT1	-0.051781618	0.240778	0.307893
TRMT2A	-0.066582446	0.131299	0.181131
TRMT2B	-0.10516309	0.01697	0.028692
TRMT5	0.344500461	8.51E-16	1.19E-14
TRMT61A	0.056165386	0.203194	0.265837
TRMT61B	0.257810851	2.90E-09	1.49E-08
TRMT6	0.27013646	4.61E-10	2.69E-09
TRMU	-0.01507098	0.732951	0.783769
TRNAU1AP	-0.199956255	4.81E-06	1.51E-05
TRNP1	0.093497638	0.033897	0.053752
TRNT1	0.208010626	1.92E-06	6.45E-06
TROAP	0.450534125	4.13E-27	3.08E-25
TROVE2	0.092616351	0.035623	0.056237
TRO	-0.076903919	0.081234	0.118217
TRPA1	0.292029362	1.39E-11	1.02E-10
TRPC1	-0.003227329	0.941757	0.953782
TRPC2	-0.329666693	1.61E-14	1.84E-13
TRPC3	-0.021267926	0.630141	0.692048
TRPC4AP	0.044739813	0.310894	0.382937
TRPC4	0.120809848	0.006051	0.011186
TRPC5	-0.122341993	0.005434	0.010142
TRPC6	-0.262352661	1.49E-09	8.05E-09

TRPC7	0.069894486	0.113139	0.158796
TRPM1	-0.138714127	0.001602	0.003282
TRPM2	0.048384133	0.273085	0.34319
TRPM3	-0.095294182	0.0306	0.049025
TRPM4	-0.251978457	6.70E-09	3.28E-08
TRPM5	-0.080253931	0.068795	0.101926
TRPM6	-0.099580756	0.023823	0.039046
TRPM7	-0.17265198	8.21E-05	0.000211
TRPM8	0.079335136	0.07204	0.106245
TRPS1	0.067206097	0.127717	0.176678
TRPT1	0.132223282	0.002642	0.005214
TRPV1	-0.157407109	0.000336	0.000777
TRPV2	-0.180097446	3.94E-05	0.000107
TRPV3	0.126421864	0.004059	0.007742
TRPV4	-0.175721632	6.09E-05	0.00016
TRPV5	-0.11537433	0.008776	0.015732
TRPV6	-0.374340775	1.41E-18	3.01E-17
TRRAP	-0.003976895	0.928262	0.943749
TRUB1	0.319238442	1.15E-13	1.16E-12
TRUB2	0.2631817	1.32E-09	7.16E-09
TRY6	0.048210005	0.274817	0.345172
TSC1	-0.297386811	5.62E-12	4.37E-11
TSC22D1	-0.05931092	0.178983	0.238089
TSC22D2	0.143293218	0.001111	0.002345
TSC22D3	-0.332474606	9.32E-15	1.10E-13
TSC22D4	-0.173290648	7.72E-05	0.0002
TSC2	-0.312052813	4.29E-13	3.97E-12
TSEN15	0.247070017	1.33E-08	6.26E-08
TSEN2	0.071177683	0.106658	0.150545
TSEN34	-0.003553906	0.935875	0.949702
TSEN54	-0.047526365	0.281689	0.352341
TSFM	0.263662981	1.23E-09	6.71E-09
TSG101	0.217492804	6.25E-07	2.27E-06
TSG1	-0.009297154	0.833295	0.86754
TSGA10IP	-0.239958613	3.53E-08	1.55E-07
TSGA10	-0.228602131	1.56E-07	6.24E-07
TSGA13	0.025559232	0.562781	0.631994
TSGA14	0.167986745	0.000128	0.00032
TSHB	-0.076306931	0.083631	0.121237
TSHR	0.009216067	0.834728	0.868646
TSHZ1	-0.133275578	0.00244	0.004844
TSHZ2	-0.090819808	0.039372	0.061539
TSHZ3	-0.069303698	0.116225	0.162591
TSIX	-0.202752428	3.51E-06	1.13E-05

TSKS	-0.075420253	0.087296	0.126011
TSKU	0.257883354	2.87E-09	1.48E-08
TSLP	-0.329180494	1.76E-14	2.01E-13
TSNARE1	-0.218843267	5.30E-07	1.95E-06
TSNAX-DISC1	0.134822162	0.002168	0.004347
TSNAXIP1	-0.26489134	1.02E-09	5.67E-09
TSNAX	0.245752756	1.60E-08	7.43E-08
TSN	0.333380846	7.81E-15	9.38E-14
TSPAN10	-0.054464781	0.217234	0.281551
TSPAN11	-0.059671514	0.176351	0.235119
TSPAN12	-0.045729404	0.300302	0.371806
TSPAN13	0.019349643	0.661323	0.720199
TSPAN14	-0.044324021	0.315417	0.387757
TSPAN15	0.002342444	0.957708	0.967682
TSPAN16	-0.024470676	0.579537	0.646705
TSPAN17	-0.044926295	0.308879	0.380775
TSPAN18	-0.101471335	0.021273	0.035275
TSPAN19	-0.168794678	0.000119	0.000298
TSPAN1	-0.028276487	0.522001	0.59394
TSPAN2	-0.106907359	0.015217	0.026003
TSPAN31	-0.08712834	0.048131	0.07385
TSPAN32	-0.436333145	2.38E-25	1.40E-23
TSPAN33	-0.066052695	0.134402	0.185041
TSPAN3	-0.248424828	1.11E-08	5.25E-08
TSPAN4	-0.31029677	5.88E-13	5.34E-12
TSPAN5	0.096139866	0.029145	0.046905
TSPAN6	0.097302146	0.027243	0.044124
TSPAN7	-0.208539919	1.81E-06	6.10E-06
TSPAN8	-0.004330813	0.921898	0.938902
TSPAN9	-0.278079563	1.34E-10	8.47E-10
TSPO2	0.125757137	0.004259	0.008093
TSPO	-0.079664201	0.070864	0.104665
TSPY1	0.030771731	0.485934	0.559432
TSPY2	0.099752054	0.023581	0.038698
TSPY3	0.050937192	0.248546	0.316429
TSPY4	0.113301929	0.010074	0.017877
TSPYL1	-0.263453537	1.27E-09	6.90E-09
TSPYL2	-0.378185194	5.90E-19	1.33E-17
TSPYL3	-0.093487668	0.033916	0.053769
TSPYL4	-0.23897483	4.02E-08	1.76E-07
TSPYL5	-0.045420042	0.303587	0.375376
TSPYL6	-0.121422383	0.005797	0.010756
TSR1	0.395060845	1.10E-20	3.18E-19
TSR2	-0.053491331	0.225578	0.290875

TSSC1	0.351415261	2.05E-16	3.11E-15
TSSC4	-0.007743254	0.860845	0.890564
TSSK1B	-0.012970971	0.769021	0.814368
TSSK3	-0.366390002	8.31E-18	1.58E-16
TSSK4	-0.240327215	3.36E-08	1.48E-07
TSSK6	-0.12971832	0.003187	0.006194
TSTA3	0.065627857	0.136931	0.188121
TSTD1	-0.23998208	3.52E-08	1.55E-07
TSTD2	-0.065918354	0.135198	0.186034
TST	-0.093935112	0.033068	0.052528
TTBK1	-0.117286588	0.007713	0.013986
TTBK2	-0.25399005	5.03E-09	2.51E-08
TTC12	-0.262892596	1.38E-09	7.45E-09
TTC13	-0.058191876	0.187339	0.247621
TTC14	-0.275346453	2.06E-10	1.27E-09
TTC15	-0.116662532	0.008046	0.014551
TTC16	-0.354653432	1.04E-16	1.65E-15
TTC17	-0.04956837	0.261504	0.330401
TTC18	-0.394334355	1.32E-20	3.73E-19
TTC19	-0.161402651	0.000235	0.000561
TTC1	0.003862758	0.930316	0.945548
TTC21A	-0.422992881	9.04E-24	4.15E-22
TTC21B	-0.166172009	0.000152	0.000375
TTC22	-0.099393895	0.024089	0.039446
TTC23L	-0.363972998	1.41E-17	2.57E-16
TTC23	-0.210683719	1.41E-06	4.82E-06
TTC24	-0.093273187	0.03433	0.054347
TTC25	-0.290973846	1.66E-11	1.20E-10
TTC26	0.059148899	0.180176	0.239436
TTC27	0.258332214	2.69E-09	1.39E-08
TTC28	-0.227795591	1.73E-07	6.87E-07
TTC29	-0.197495859	6.32E-06	1.95E-05
TTC30A	-0.043136831	0.328566	0.401969
TTC30B	-0.060024927	0.173799	0.232103
TTC31	-0.272566594	3.18E-10	1.90E-09
TTC32	0.01085535	0.805871	0.844753
TTC33	0.054465116	0.217232	0.281551
TTC35	0.118873203	0.00692	0.012655
TTC36	-0.06024096	0.172252	0.230275
TTC37	-0.082217485	0.062261	0.093225
TTC38	-0.11996817	0.006416	0.011808
TTC39A	-0.01153468	0.793988	0.834923
TTC39B	-0.032371158	0.463543	0.537461
TTC39C	0.17658753	5.59E-05	0.000148

TTC3	-0.234153481	7.62E-08	3.19E-07
TTC4	0.221955131	3.61E-07	1.36E-06
TTC5	0.216734336	6.85E-07	2.47E-06
TTC7A	-0.141677867	0.001266	0.002644
TTC7B	0.014635035	0.740393	0.789849
TTC8	0.228315105	1.62E-07	6.45E-07
TTC9B	0.148866645	0.000701	0.001535
TTC9C	0.25357088	5.34E-09	2.66E-08
TTC9	0.032774201	0.457992	0.531872
TTF1	0.008093755	0.854615	0.885218
TTF2	0.247110498	1.33E-08	6.22E-08
TTK	0.501716026	3.48E-34	8.38E-32
TTLL10	-0.272821709	3.05E-10	1.83E-09
TTLL11	-0.177331029	5.20E-05	0.000138
TTLL12	0.233435341	8.38E-08	3.49E-07
TTLL13	-0.106903408	0.015221	0.026007
TTLL1	-0.321788215	7.17E-14	7.43E-13
TTLL2	-0.218972379	5.22E-07	1.92E-06
TTLL3	-0.405265051	8.89E-22	3.05E-20
TTLL4	0.119335804	0.006703	0.012296
TTLL5	0.084548684	0.055176	0.083607
TTLL6	-0.242017544	2.67E-08	1.20E-07
TTLL7	0.046699149	0.290158	0.361306
TTLL8	0.086698985	0.049248	0.075448
TTLL9	-0.344801585	8.01E-16	1.12E-14
TTL	0.297009511	6.00E-12	4.63E-11
TTN	-0.29398392	1.00E-11	7.48E-11
TTPAL	0.225620898	2.28E-07	8.88E-07
TTPA	0.003710686	0.933053	0.947655
TTR	-0.05129286	0.245253	0.312855
TTTY10	-0.069013897	0.117763	0.164432
TTTY14	0.046001903	0.297428	0.368682
TTTY15	0.161734294	0.000228	0.000546
TTTY16	0.016294371	0.712199	0.765881
TTTY1B	0.04454715	0.312984	0.385075
TTTY2	-0.015135675	0.731848	0.782853
TTTY4C	-0.01265135	0.774557	0.818886
TTTY5	-0.058115117	0.187922	0.248294
TTTY6B	0.000805887	0.985444	0.989007
TTTY6	-0.022407906	0.611913	0.67604
TTTY7	0.025394611	0.5653	0.634181
TTTY8	-0.09814862	0.025926	0.042193
TTTY9B	-0.039205502	0.374598	0.449454
TTYH1	0.093558883	0.03378	0.053574

TTYH2	-0.33386739	7.10E-15	8.60E-14
TTYH3	0.011448148	0.795499	0.836248
TUBA1A	-0.043074919	0.329261	0.402672
TUBA1B	0.431012127	1.04E-24	5.49E-23
TUBA1C	0.563315599	1.86E-44	3.10E-41
TUBA3C	0.110297099	0.012259	0.021346
TUBA3D	-0.068953833	0.118083	0.16481
TUBA3E	0.019730256	0.655087	0.714615
TUBA4A	0.164519569	0.000177	0.000432
TUBA4B	-0.229357456	1.42E-07	5.71E-07
TUBA8	-0.17799145	4.87E-05	0.00013
TUBAL3	0.191610577	1.20E-05	3.54E-05
TUBB1	-0.218514624	5.51E-07	2.02E-06
TUBB2A	0.094757217	0.031555	0.050394
TUBB2B	0.042095874	0.340383	0.414498
TUBB2C	0.10521774	0.016913	0.028602
TUBB3	0.374063205	1.50E-18	3.19E-17
TUBB4Q	-0.080616052	0.06755	0.100273
TUBB4	0.144908953	0.000974	0.002075
TUBB6	0.072614771	0.099751	0.14196
TUBB8	-0.1113088	0.011481	0.020128
TUBBP5	-0.134176385	0.002278	0.004546
TUBB	0.345765323	6.58E-16	9.33E-15
TUBD1	0.079937382	0.0699	0.103355
TUBE1	-0.051234456	0.245792	0.313442
TUBG1	0.307701888	9.34E-13	8.23E-12
TUBG2	-0.276147859	1.82E-10	1.13E-09
TUBGCP2	-0.08617992	0.050628	0.077396
TUBGCP3	-0.074247586	0.092341	0.132357
TUBGCP4	0.0914153	0.038094	0.059723
TUBGCP5	-0.033108366	0.453418	0.527635
TUBGCP6	-0.504142877	1.50E-34	3.89E-32
TUB	-0.344704207	8.17E-16	1.14E-14
TUFM	0.122726115	0.005289	0.009891
TUFT1	0.119834257	0.006475	0.011908
TUG1	-0.068487183	0.120598	0.167992
TULP1	0.095271833	0.030639	0.049076
TULP2	0.003181281	0.942586	0.954428
TULP3	0.080065353	0.069451	0.102814
TULP4	-0.220268149	4.45E-07	1.66E-06
TUSC1	-0.144855342	0.000978	0.002083
TUSC2	-0.003214999	0.941979	0.953958
TUSC3	0.030356475	0.491842	0.565094
TUSC5	-0.036924372	0.403044	0.477735

TUT1	-0.089177719	0.043086	0.066808
TWF1	0.497742506	1.36E-33	2.90E-31
TWF2	-0.041787055	0.34394	0.418142
TWIST1	0.168432332	0.000123	0.000308
TWIST2	-0.008311131	0.850756	0.882134
TWISTNB	0.279458854	1.08E-10	6.90E-10
TWSG1	0.1783941	4.68E-05	0.000125
TXK	-0.222461262	3.39E-07	1.29E-06
TXLNA	-0.177286824	5.22E-05	0.000139
TXLNB	-0.159325028	0.000283	0.000666
TXLNG	-0.069323493	0.11612	0.162468
TXN2	0.036989254	0.402218	0.476869
TXNDC11	-0.23789105	4.65E-08	2.01E-07
TXNDC12	0.181965303	3.27E-05	8.98E-05
TXNDC15	-0.111622497	0.011248	0.019758
TXNDC16	0.014284282	0.746399	0.79506
TXNDC17	0.123936535	0.004853	0.009144
TXNDC2	0.156144701	0.000376	0.00086
TXNDC3	-0.202057935	3.80E-06	1.21E-05
TXNDC5	0.149018297	0.000693	0.001517
TXNDC6	-0.294113987	9.80E-12	7.35E-11
TXNDC9	0.392131072	2.24E-20	6.18E-19
TXNIP	-0.347600774	4.52E-16	6.58E-15
TXNL1	0.28342497	5.71E-11	3.80E-10
TXNL4A	0.216808763	6.78E-07	2.45E-06
TXNL4B	0.148784598	0.000706	0.001545
TXNRD1	0.356744282	6.68E-17	1.10E-15
TXNRD2	-0.110491813	0.012106	0.021097
TXNRD3IT1	-0.052914552	0.230627	0.296649
TXN	0.271959385	3.49E-10	2.07E-09
TYK2	-0.348091823	4.08E-16	5.98E-15
TYMP	-7.63E-05	0.998622	0.999165
TYMS	0.377752644	6.51E-19	1.45E-17
TYRO3	0.075140733	0.088478	0.127524
TYROBP	-0.170577323	0.0001	0.000255
TYRP1	-0.254065289	4.98E-09	2.48E-08
TYR	-0.079976857	0.069761	0.103211
TYSND1	0.061952032	0.160364	0.216184
TYW1B	-0.107990805	0.01421	0.024445
TYW1	0.051913587	0.23958	0.306459
TYW3	0.001428919	0.974194	0.980621
T	-0.226955862	1.93E-07	7.59E-07
U2AF1L4	-0.158887955	0.000295	0.000689
U2AF1	-0.025976624	0.556418	0.625833

U2AF2	0.181892497	3.29E-05	9.05E-05
UACA	-0.013812686	0.754499	0.801917
UAP1L1	-0.234499109	7.29E-08	3.06E-07
UAP1	0.313464668	3.32E-13	3.13E-12
UBA1	0.02365039	0.592316	0.658245
UBA2	0.373304693	1.79E-18	3.70E-17
UBA3	0.151912764	0.000542	0.001207
UBA52	0.067264491	0.127386	0.176257
UBA5	0.133350899	0.002426	0.004817
UBA6	0.291388857	1.55E-11	1.12E-10
UBA7	-0.446746536	1.24E-26	8.71E-25
UBAC1	0.10502593	0.017115	0.028916
UBAC2	0.144892708	0.000975	0.002078
UBAP1	0.105505322	0.016613	0.02814
UBAP2L	0.039766764	0.367793	0.442435
UBAP2	0.116751313	0.007998	0.014469
UBASH3A	-0.191395732	1.22E-05	3.62E-05
UBASH3B	-0.046337505	0.293914	0.365255
UBB	0.044082201	0.318067	0.390655
UBC	0.117054139	0.007836	0.014194
UBD	0.054212272	0.219377	0.283905
UBE2A	0.125303846	0.004401	0.008344
UBE2B	0.032629496	0.459981	0.53392
UBE2CBP	0.122876454	0.005233	0.009799
UBE2C	0.487268585	4.58E-32	7.38E-30
UBE2D1	0.351832253	1.88E-16	2.87E-15
UBE2D2	0.173348268	7.68E-05	0.000199
UBE2D3	0.152453165	0.000517	0.001157
UBE2D4	-0.014050996	0.750402	0.798614
UBE2DNL	-0.044674935	0.311597	0.383604
UBE2E1	0.263505884	1.26E-09	6.85E-09
UBE2E2	0.040820542	0.355226	0.429727
UBE2E3	0.228532371	1.58E-07	6.29E-07
UBE2F	0.323642468	5.06E-14	5.36E-13
UBE2G1	0.294426946	9.29E-12	6.99E-11
UBE2G2	-0.373576835	1.68E-18	3.51E-17
UBE2H	0.134214576	0.002271	0.004534
UBE2I	0.118218565	0.007238	0.013196
UBE2J1	0.122809338	0.005258	0.009841
UBE2J2	0.0683155	0.121534	0.169177
UBE2K	0.402859902	1.62E-21	5.38E-20
UBE2L3	0.187529113	1.84E-05	5.29E-05
UBE2L6	0.018046473	0.68285	0.739732
UBE2MP1	0.288826864	2.36E-11	1.67E-10

UBE2M	0.219180628	5.08E-07	1.88E-06
UBE2NL	0.250574327	8.17E-09	3.96E-08
UBE2N	0.439533212	9.71E-26	6.10E-24
UBE2O	-0.00359166	0.935195	0.94906
UBE2Q1	0.09105044	0.038872	0.060844
UBE2Q2P1	-0.182260305	3.17E-05	8.73E-05
UBE2Q2	0.202142408	3.76E-06	1.20E-05
UBE2QL1	-0.036548536	0.407852	0.482604
UBE2R2	0.323174463	5.53E-14	5.80E-13
UBE2S	0.37001753	3.73E-18	7.41E-17
UBE2T	0.535250838	1.63E-39	1.25E-36
UBE2U	-0.165471959	0.000162	0.000398
UBE2V1	0.230342852	1.25E-07	5.08E-07
UBE2V2	0.441186645	6.09E-26	3.90E-24
UBE2W	0.245218024	1.72E-08	7.96E-08
UBE2Z	0.171946173	8.79E-05	0.000225
UBE3A	0.13464792	0.002197	0.004401
UBE3B	-0.057976621	0.188978	0.249426
UBE3C	0.254881785	4.43E-09	2.22E-08
UBE4A	-0.01322429	0.764642	0.810632
UBE4B	-0.142826693	0.001154	0.002427
UBFD1	0.182455404	3.11E-05	8.58E-05
UBIAD1	-0.010268533	0.816173	0.853359
UBL3	-0.22676283	1.98E-07	7.77E-07
UBL4A	0.307600057	9.51E-13	8.36E-12
UBL4B	-0.032047445	0.468028	0.541803
UBL5	0.131469266	0.002797	0.005491
UBL7	0.01846199	0.675957	0.733537
UBLCP1	-0.023338836	0.597203	0.662829
UBN1	-0.146404806	0.000861	0.001852
UBN2	-0.22042432	4.36E-07	1.63E-06
UBOX5	-0.176909542	5.42E-05	0.000143
UBP1	-0.08573024	0.051849	0.079045
UBQLN1	0.258139261	2.77E-09	1.43E-08
UBQLN2	0.068574255	0.120126	0.167404
UBQLN3	-0.107368028	0.014781	0.025347
UBQLN4	0.152998566	0.000494	0.001108
UBQLNL	-0.343062708	1.14E-15	1.56E-14
UBR1	-0.056736217	0.198633	0.260429
UBR2	-0.052982889	0.230025	0.29596
UBR3	-0.13727176	0.001794	0.003645
UBR4	-0.121402331	0.005805	0.010769
UBR5	0.141097534	0.001326	0.002758
UBR7	0.084074289	0.056561	0.085472

UBTD1	-0.333552335	7.55E-15	9.08E-14
UBTD2	0.025585715	0.562376	0.631717
UBTFL1	-0.046884771	0.288243	0.359212
UBTF	-0.023344123	0.59712	0.662829
UBXN10	-0.178439195	4.65E-05	0.000125
UBXN11	-0.380731318	3.28E-19	7.67E-18
UBXN1	-0.038511567	0.383118	0.457753
UBXN2A	0.315527988	2.28E-13	2.20E-12
UBXN2B	0.091495755	0.037924	0.059513
UBXN4	0.181231306	3.52E-05	9.64E-05
UBXN6	-0.186095076	2.14E-05	6.08E-05
UBXN7	0.089020081	0.043457	0.06733
UBXN8	0.041720423	0.344711	0.418926
UCA1	0.104983693	0.01716	0.028977
UCHL1	0.2582082	2.74E-09	1.41E-08
UCHL3	0.359958577	3.36E-17	5.82E-16
UCHL5	0.412500238	1.41E-22	5.35E-21
UCK1	-0.140019205	0.001445	0.002986
UCK2	0.472455453	5.39E-30	6.02E-28
UCKL1AS	-0.248888944	1.04E-08	4.95E-08
UCKL1	-0.060980095	0.167039	0.224063
UCMA	0.000795794	0.985626	0.98914
UCN2	0.153098697	0.000489	0.001099
UCN3	-0.176480022	5.65E-05	0.000149
UCN	-0.10672972	0.015388	0.026254
UCP1	0.072437698	0.100582	0.143011
UCP2	-0.04974545	0.259802	0.328666
UCP3	-0.317676383	1.54E-13	1.52E-12
UEVLD	0.225318762	2.37E-07	9.20E-07
UFC1	0.067584508	0.125581	0.173976
UFD1L	0.263761612	1.21E-09	6.62E-09
UFM1	0.05100747	0.247893	0.315738
UFSP1	0.119247056	0.006744	0.012367
UFSP2	-0.093467478	0.033955	0.053822
UGCG	-0.047642543	0.280513	0.351155
UGDH	0.351124389	2.18E-16	3.29E-15
UGGT1	0.232949203	8.93E-08	3.70E-07
UGGT2	0.166396757	0.000149	0.000368
UGP2	0.287349349	3.02E-11	2.10E-10
UGT1A10	-0.03624642	0.411741	0.486426
UGT1A1	0.144297868	0.001024	0.002175
UGT1A3	0.244032123	2.03E-08	9.27E-08
UGT1A4	0.104994832	0.017148	0.028963
UGT1A5	0.090374883	0.04035	0.062965

UGT1A6	0.066728219	0.130455	0.180104
UGT1A7	0.063122195	0.152598	0.206886
UGT1A8	-0.023581317	0.593397	0.659301
UGT1A9	0.103279951	0.019059	0.031902
UGT2A1	0.037593539	0.394569	0.46948
UGT2A3	0.012024017	0.785457	0.827651
UGT2B10	0.127189177	0.003839	0.007349
UGT2B11	0.152106597	0.000533	0.001189
UGT2B15	-0.058343277	0.186192	0.246236
UGT2B28	0.122853862	0.005241	0.009812
UGT2B4	0.148032865	0.000752	0.001637
UGT2B7	0.08697609	0.048525	0.074437
UGT3A1	0.170751729	9.85E-05	0.000251
UGT3A2	0.008043049	0.855516	0.886105
UGT8	0.007237464	0.869851	0.898439
UHMK1	0.100192223	0.022971	0.037808
UHRF1BP1L	0.243388182	2.22E-08	1.01E-07
UHRF1BP1	0.082001046	0.062955	0.094145
UHRF1	0.436609845	2.20E-25	1.30E-23
UHRF2	0.148731473	0.000709	0.001551
UIMC1	-0.001787776	0.967716	0.97539
ULBP1	-0.116777149	0.007984	0.014446
ULBP2	-0.010776988	0.807245	0.845838
ULBP3	-0.111725763	0.011173	0.019631
ULK1	0.005116122	0.907794	0.927895
ULK2	-0.274929669	2.20E-10	1.35E-09
ULK3	-0.27382447	2.61E-10	1.58E-09
ULK4	-0.046880193	0.28829	0.359249
UMODL1	0.001464884	0.973545	0.980214
UMOD	0.068736438	0.11925	0.166299
UMPS	0.306420548	1.17E-12	1.01E-11
UNC119B	-0.096567389	0.028433	0.045891
UNC119	-0.032000912	0.468675	0.542373
UNC13A	0.001693407	0.96942	0.976996
UNC13B	-0.254297229	4.82E-09	2.41E-08
UNC13C	-0.114081768	0.009567	0.017051
UNC13D	-0.016943574	0.701272	0.756777
UNC45A	-0.103894688	0.018354	0.03082
UNC45B	-0.351777785	1.90E-16	2.90E-15
UNC50	0.079873899	0.070123	0.103662
UNC5A	-0.039911691	0.366049	0.440758
UNC5B	-0.082157381	0.062453	0.093464
UNC5CL	-0.109337894	0.013039	0.022593
UNC5C	-0.162412602	0.000214	0.000516

UNC5D	0.199909539	4.83E-06	1.52E-05
UNC80	-0.136472157	0.001909	0.003861
UNC93A	0.060845567	0.167979	0.225234
UNC93B1	-0.178147086	4.79E-05	0.000128
UNG	0.442287277	4.45E-26	2.91E-24
UNKL	-0.191319859	1.23E-05	3.64E-05
UNK	-0.10652586	0.015586	0.026559
UOX	0.103583392	0.018708	0.031368
UPB1	-0.263282757	1.30E-09	7.07E-09
UPF0639	-0.156997359	0.000348	0.000803
UPF1	-0.217028296	6.61E-07	2.39E-06
UPF2	0.020589678	0.641095	0.702148
UPF3A	-0.186835457	1.98E-05	5.66E-05
UPF3B	0.049993111	0.257434	0.326125
UPK1A	0.014471233	0.743196	0.792257
UPK1B	0.122426881	0.005402	0.010089
UPK2	0.082892067	0.060137	0.09033
UPK3A	-0.17764804	5.04E-05	0.000134
UPK3BL	-0.086164886	0.050668	0.077452
UPK3B	-0.206618833	2.26E-06	7.48E-06
UPP1	0.120762439	0.006071	0.011217
UPP2	-0.109940034	0.012544	0.021794
UPRT	-0.222097405	3.55E-07	1.34E-06
UQCC	0.005291601	0.904646	0.925292
UQCR10	-0.014322466	0.745745	0.794475
UQCR11	0.114496104	0.009307	0.016617
UQCRB	0.238512674	4.28E-08	1.86E-07
UQCRC1	0.204206764	2.98E-06	9.67E-06
UQCRC2	0.182257613	3.17E-05	8.74E-05
UQCRFS1	0.272966228	2.98E-10	1.79E-09
UQCRHL	0.310943174	5.24E-13	4.78E-12
UQCRH	0.285258233	4.24E-11	2.88E-10
UQCRQ	0.19471714	8.56E-06	2.59E-05
URB1	0.039543764	0.370488	0.445298
URB2	0.417232445	4.14E-23	1.73E-21
URGCP	-0.094110112	0.032741	0.052091
URM1	0.147417	0.000792	0.001715
UROC1	-0.129273586	0.003294	0.006385
UROD	-0.078563476	0.074862	0.10996
UROS	0.059918406	0.174565	0.232986
USE1	-0.05414869	0.219919	0.284532
USF1	-0.071756912	0.10383	0.146948
USF2	-0.114581701	0.009254	0.016533
USH1C	0.046184611	0.295512	0.366829

USH1G	-0.078030788	0.076861	0.112607
USH2A	-0.079951647	0.069849	0.103319
USHBP1	-0.364803156	1.18E-17	2.17E-16
USMG5	0.352671831	1.58E-16	2.44E-15
USO1	0.222584996	3.34E-07	1.27E-06
USP10	0.266634321	7.85E-10	4.44E-09
USP11	-0.12839879	0.003513	0.006775
USP12	-0.072331965	0.101081	0.143679
USP13	-0.134770102	0.002177	0.004363
USP14	0.363355483	1.61E-17	2.91E-16
USP15	0.208257954	1.87E-06	6.28E-06
USP16	-0.030633226	0.4879	0.561243
USP17L2	-0.160542677	0.000254	0.000602
USP17L6P	0.009100716	0.836767	0.870384
USP17	0.023126494	0.600544	0.665872
USP18	0.050274101	0.254766	0.323287
USP19	-0.231314546	1.10E-07	4.52E-07
USP1	0.275839492	1.91E-10	1.18E-09
USP20	-0.364473706	1.26E-17	2.32E-16
USP21	-0.05749609	0.192677	0.25367
USP22	-0.078017598	0.076911	0.112664
USP24	-0.109596634	0.012825	0.022238
USP25	-0.105275857	0.016852	0.028506
USP26	-0.040051668	0.364369	0.439026
USP27X	-0.208292112	1.86E-06	6.26E-06
USP28	-0.00114481	0.979324	0.98471
USP29	-0.026153886	0.553727	0.623402
USP2	-0.315738089	2.20E-13	2.12E-12
USP30	-0.050036928	0.257017	0.325741
USP31	0.070738294	0.108843	0.153348
USP32	0.124312971	0.004725	0.008919
USP33	0.092500673	0.035854	0.056572
USP34	-0.170659849	9.94E-05	0.000253
USP35	0.006783301	0.877952	0.904656
USP36	-0.159741223	0.000273	0.000643
USP37	0.104462696	0.017722	0.029856
USP38	0.116554445	0.008105	0.014647
USP39	0.245302988	1.70E-08	7.87E-08
USP3	0.193616661	9.64E-06	2.89E-05
USP40	-0.153952994	0.000455	0.001027
USP42	0.086204871	0.050561	0.077316
USP43	-0.043659467	0.322734	0.395774
USP44	-0.332716605	8.89E-15	1.06E-13
USP45	0.035456401	0.422015	0.49657

USP46	0.041037467	0.352673	0.427278
USP47	-0.113835142	0.009725	0.017315
USP48	-0.230995463	1.15E-07	4.69E-07
USP49	-0.155720243	0.00039	0.00089
USP4	-0.306011681	1.26E-12	1.09E-11
USP50	0.035237598	0.424886	0.499507
USP51	-0.24822827	1.14E-08	5.38E-08
USP53	-0.231832688	1.03E-07	4.24E-07
USP54	-0.339237207	2.46E-15	3.21E-14
USP5	0.247861621	1.20E-08	5.65E-08
USP6NL	0.007835928	0.859197	0.889273
USP6	-0.169705884	0.000109	0.000276
USP7	-0.00666338	0.880094	0.906113
USP8	-0.051668831	0.241806	0.309029
USP9X	0.00754753	0.864328	0.893473
USP9Y	0.132474455	0.002593	0.005121
USPL1	-0.096842959	0.027982	0.045229
UST	-0.247209255	1.31E-08	6.15E-08
UTF1	-0.137519091	0.001759	0.003579
UTP11L	0.281528707	7.75E-11	5.07E-10
UTP14A	0.281707954	7.53E-11	4.93E-10
UTP14C	-0.015854878	0.71963	0.772087
UTP15	0.242970653	2.35E-08	1.06E-07
UTP18	0.325658326	3.46E-14	3.77E-13
UTP20	0.252498766	6.22E-09	3.06E-08
UTP23	0.264365011	1.10E-09	6.10E-09
UTP3	-0.009992831	0.821024	0.857306
UTP6	0.309112826	7.27E-13	6.51E-12
UTRN	-0.371673939	2.57E-18	5.23E-17
UTS2D	-0.110186965	0.012346	0.021481
UTS2R	0.003118786	0.943712	0.955327
UTS2	-0.062599422	0.156031	0.211026
UTY	0.128711723	0.003433	0.006632
UVRAG	-0.149068614	0.00069	0.001511
UXS1	-0.145206723	0.00095	0.002028
UXT	0.204434482	2.90E-06	9.44E-06
VAC14	-0.039773671	0.36771	0.442385
VAMP1	-0.284353272	4.91E-11	3.30E-10
VAMP2	-0.420612387	1.70E-23	7.50E-22
VAMP3	-0.117496931	0.007603	0.013806
VAMP4	-0.058561094	0.184551	0.244308
VAMP5	0.022402153	0.612005	0.676104
VAMP7	0.085664097	0.052031	0.079298
VAMP8	-0.158018226	0.000318	0.000739

VANGL1	0.204826371	2.78E-06	9.05E-06
VANGL2	-0.177374811	5.17E-05	0.000138
VAPA	0.101373403	0.021399	0.035458
VAPB	0.274262895	2.44E-10	1.48E-09
VARS2	-0.05743528	0.193149	0.254224
VARS	0.257459726	3.05E-09	1.56E-08
VASH1	-0.280090023	9.75E-11	6.29E-10
VASH2	0.06869699	0.119462	0.166561
VASN	-0.162395189	0.000215	0.000517
VASP	0.144561134	0.001002	0.002132
VAT1L	0.04719718	0.285039	0.355952
VAT1	0.040319638	0.361166	0.435902
VAV1	-0.250157552	8.67E-09	4.19E-08
VAV2	-8.47E-05	0.998471	0.99912
VAV3	-0.131141751	0.002866	0.005613
VAX1	0.186539467	2.04E-05	5.82E-05
VAX2	0.096227786	0.028998	0.046693
VBP1	0.3440099	9.41E-16	1.30E-14
VCAM1	-0.000378483	0.993164	0.994906
VCAN	0.184696486	2.47E-05	6.94E-05
VCL	0.045144134	0.306537	0.378284
VCPIP1	0.22339338	3.02E-07	1.15E-06
VCP	0.228638854	1.56E-07	6.21E-07
VCX2	0.070541731	0.109832	0.154546
VCX3A	0.07689858	0.081255	0.118239
VCX3B	0.062661007	0.155624	0.210518
VCX	0.090112903	0.040936	0.063824
VCY	0.070552763	0.109776	0.154489
VDAC1	0.434032337	4.51E-25	2.56E-23
VDAC2	0.466652369	3.28E-29	3.17E-27
VDAC3	0.328507461	2.01E-14	2.26E-13
VDR	-0.194926431	8.36E-06	2.53E-05
VEGFA	0.082650013	0.060892	0.091389
VEGFB	-0.137046321	0.001826	0.003704
VEGFC	0.261237287	1.76E-09	9.36E-09
VENTXP1	0.044200388	0.31677	0.389157
VENTXP7	0.189411767	1.51E-05	4.40E-05
VENTX	-0.308687037	7.84E-13	6.99E-12
VEPH1	-0.323407529	5.29E-14	5.57E-13
VEZF1	-0.103591105	0.018699	0.031356
VEZT	0.167688445	0.000132	0.000329
VGF	0.195534671	7.83E-06	2.38E-05
VGLL1	-0.186834264	1.98E-05	5.66E-05
VGLL2	0.064695701	0.142608	0.195063

VGLL3	-0.024968246	0.571849	0.639948
VGLL4	-0.099390341	0.024094	0.039451
VHLL	-0.048859687	0.268394	0.33808
VHL	0.006081946	0.890488	0.91432
VIL1	0.193859314	9.39E-06	2.82E-05
VILL	-0.231212351	1.12E-07	4.57E-07
VIM	-0.191380323	1.23E-05	3.62E-05
VIPAR	0.112512487	0.010612	0.018736
VIPR1	-0.483591331	1.53E-31	2.23E-29
VIPR2	-0.022730669	0.606795	0.671461
VIP	-0.264778533	1.04E-09	5.76E-09
VIT	-0.149413724	0.00067	0.001471
VKORC1L1	0.05659413	0.199761	0.261756
VKORC1	-0.0005211	0.990588	0.993072
VLDLR	-0.028304687	0.521586	0.593569
VMA21	0.165742523	0.000158	0.000389
VMAC	-0.379585025	4.28E-19	9.85E-18
VMO1	-0.082556381	0.061186	0.091802
VN1R1	-0.233350158	8.47E-08	3.53E-07
VN1R2	-0.01260107	0.775429	0.819331
VN1R4	-0.075978664	0.084973	0.123005
VN1R5	-0.164112063	0.000184	0.000447
VNN1	0.021018481	0.634161	0.695659
VNN2	-0.134660293	0.002195	0.004398
VNN3	-0.05585977	0.205668	0.26858
VOPP1	0.112433191	0.010667	0.018822
VPRBP	-0.029993811	0.497032	0.570126
VPREB1	-0.00899886	0.838569	0.871714
VPREB3	-0.08236714	0.061784	0.092581
VPS11	-0.259811264	2.17E-09	1.14E-08
VPS13A	-0.253518545	5.38E-09	2.68E-08
VPS13B	-0.128796345	0.003412	0.006595
VPS13C	-0.347081569	5.02E-16	7.29E-15
VPS13D	-0.372491114	2.14E-18	4.40E-17
VPS16	-0.142901038	0.001147	0.002414
VPS18	0.039450591	0.371617	0.446521
VPS24	-0.029537902	0.503598	0.576264
VPS25	0.225844032	2.22E-07	8.65E-07
VPS26A	0.316514627	1.90E-13	1.85E-12
VPS26B	-0.189975738	1.42E-05	4.17E-05
VPS28	-0.038010739	0.389339	0.464161
VPS29	0.303680538	1.90E-12	1.59E-11
VPS33A	0.365820969	9.42E-18	1.77E-16
VPS33B	0.089479333	0.042382	0.065807

VPS35	0.191485392	1.21E-05	3.58E-05
VPS36	-0.07259546	0.099842	0.142048
VPS37A	0.192377649	1.10E-05	3.27E-05
VPS37B	-0.047543729	0.281513	0.352165
VPS37C	0.050896482	0.248924	0.31683
VPS37D	0.067687511	0.125005	0.173285
VPS39	-0.277372146	1.50E-10	9.41E-10
VPS41	-0.086750517	0.049113	0.075253
VPS45	0.04844929	0.272439	0.342464
VPS4A	-0.047549018	0.28146	0.35212
VPS4B	0.09547803	0.030279	0.04857
VPS52	-0.045229783	0.305619	0.377361
VPS53	-0.196862782	6.77E-06	2.08E-05
VPS54	0.087962083	0.046022	0.070951
VPS72	0.243813403	2.09E-08	9.53E-08
VPS8	-0.20043991	4.56E-06	1.44E-05
VRK1	0.426253498	3.77E-24	1.87E-22
VRK2	0.339699771	2.24E-15	2.95E-14
VRK3	-0.120521974	0.006173	0.011396
VSIG10L	0.06324452	0.151802	0.205934
VSIG10	-0.056063165	0.204019	0.266648
VSIG1	-0.070613598	0.109469	0.154122
VSIG2	-0.343391493	1.07E-15	1.47E-14
VSIG4	-0.071635826	0.104416	0.147665
VSIG8	-0.183239174	2.87E-05	7.97E-05
VSNL1	-0.011105281	0.801494	0.840875
VSTM1	-0.085390293	0.052789	0.080325
VSTM2A	-0.050641951	0.251302	0.319429
VSTM2B	-0.021847511	0.620845	0.684029
VSTM2L	-0.0307308	0.486515	0.559939
VSX1	0.013583834	0.758439	0.805242
VSX2	-0.04568293	0.300794	0.372346
VTA1	0.305961107	1.27E-12	1.09E-11
VTCN1	-0.163614478	0.000192	0.000466
VTI1A	0.223799854	2.87E-07	1.10E-06
VTI1B	0.283667231	5.49E-11	3.66E-10
VTN	0.040473206	0.359338	0.434037
VWA1	-0.176947026	5.40E-05	0.000143
VWA2	-0.347666433	4.46E-16	6.50E-15
VWA3A	-0.35121968	2.14E-16	3.23E-15
VWA3B	-0.192268379	1.11E-05	3.31E-05
VWA5A	-0.280489759	9.15E-11	5.93E-10
VWA5B1	-0.263684352	1.22E-09	6.69E-09
VWA5B2	-0.083934986	0.056973	0.08601

VWC2L	-0.021247651	0.630468	0.692357
VWC2	-0.254903922	4.42E-09	2.22E-08
VWCE	-0.224443429	2.65E-07	1.02E-06
VWDE	0.065385392	0.138391	0.189905
VWF	-0.312993729	3.62E-13	3.39E-12
WAC	0.125616735	0.004302	0.008171
WAPAL	0.143212142	0.001118	0.002359
WARS2	0.120588919	0.006145	0.011347
WARS	0.146249745	0.000872	0.001874
WASF1	0.215007154	8.43E-07	2.99E-06
WASF2	-0.101385343	0.021384	0.035438
WASF3	-0.227114082	1.89E-07	7.45E-07
WASH2P	-0.299000639	4.27E-12	3.36E-11
WASH3P	-0.259258702	2.35E-09	1.23E-08
WASH5P	-0.213645132	9.92E-07	3.49E-06
WASH7P	-0.29719494	5.81E-12	4.50E-11
WASL	0.138281878	0.001657	0.003388
WAS	-0.257651312	2.97E-09	1.52E-08
WBP11P1	0.25888506	2.48E-09	1.29E-08
WBP11	0.309468005	6.82E-13	6.13E-12
WBP1	-0.119825825	0.006479	0.011913
WBP2NL	-0.23843552	4.33E-08	1.88E-07
WBP2	-0.303284365	2.03E-12	1.69E-11
WBP4	0.009336739	0.832595	0.866857
WBP5	0.044655894	0.311803	0.383764
WBSCR16	0.124034722	0.00482	0.009085
WBSCR17	-0.365390696	1.03E-17	1.93E-16
WBSCR22	0.164518959	0.000177	0.000432
WBSCR26	-0.186999507	1.94E-05	5.57E-05
WBSCR27	-0.042820366	0.33213	0.405783
WBSCR28	-0.009928831	0.822151	0.858263
WDFY1	0.035589361	0.420275	0.494931
WDFY2	-0.196390849	7.13E-06	2.18E-05
WDFY3	-0.256093073	3.72E-09	1.89E-08
WDFY4	-0.202218193	3.73E-06	1.19E-05
WDHD1	0.461910341	1.40E-28	1.25E-26
WDR11	-0.084019167	0.056723	0.085698
WDR12	0.490257271	1.70E-32	2.96E-30
WDR13	-0.167618293	0.000133	0.00033
WDR16	-0.209754972	1.57E-06	5.33E-06
WDR17	-0.187868681	1.78E-05	5.12E-05
WDR18	0.121002241	0.00597	0.011053
WDR19	-0.317364514	1.63E-13	1.60E-12
WDR1	0.118108438	0.007292	0.013292

WDR20	0.105639117	0.016475	0.027935
WDR24	-0.192453558	1.09E-05	3.25E-05
WDR25	0.019996592	0.650738	0.710646
WDR26	-0.044810542	0.310129	0.38215
WDR27	-0.300514272	3.29E-12	2.64E-11
WDR31	-0.086320614	0.050251	0.076902
WDR33	-0.185089121	2.37E-05	6.68E-05
WDR34	0.072138401	0.102	0.144697
WDR35	-0.135676305	0.00203	0.004092
WDR36	0.140769779	0.001361	0.002826
WDR37	-0.30719098	1.02E-12	8.95E-12
WDR38	-0.190431748	1.36E-05	3.98E-05
WDR3	0.303737781	1.88E-12	1.57E-11
WDR41	-0.000911615	0.983535	0.987775
WDR43	0.514130086	4.34E-36	1.52E-33
WDR44	0.074471164	0.091362	0.131207
WDR45L	0.177160073	5.28E-05	0.00014
WDR45	-0.052368623	0.23548	0.302065
WDR46	0.062929399	0.153857	0.208403
WDR47	-0.172906636	8.01E-05	0.000207
WDR48	-0.130715444	0.002959	0.005786
WDR49	-0.241587935	2.83E-08	1.26E-07
WDR4	0.178532143	4.61E-05	0.000124
WDR52	-0.365853048	9.35E-18	1.76E-16
WDR53	0.209891661	1.55E-06	5.26E-06
WDR54	-0.079671989	0.070836	0.104632
WDR55	-0.046500259	0.29222	0.363488
WDR59	-0.15066889	0.000602	0.001333
WDR5B	-0.218995768	5.20E-07	1.92E-06
WDR5	0.223085241	3.14E-07	1.20E-06
WDR60	-0.164454917	0.000178	0.000434
WDR61	0.065807217	0.135859	0.186815
WDR62	0.217090427	6.56E-07	2.37E-06
WDR63	-0.261071871	1.80E-09	9.59E-09
WDR64	-0.004773399	0.913946	0.932752
WDR65	-0.291118545	1.62E-11	1.17E-10
WDR66	0.042284506	0.338222	0.412293
WDR67	0.433051862	5.91E-25	3.29E-23
WDR69	0.01589076	0.719023	0.771642
WDR6	-0.273195151	2.88E-10	1.73E-09
WDR70	0.074261409	0.09228	0.132279
WDR72	0.135556426	0.002049	0.004127
WDR73	-0.001141166	0.97939	0.98471
WDR74	0.24462878	1.87E-08	8.59E-08

WDR75	0.359310657	3.86E-17	6.59E-16
WDR76	0.3462399	5.97E-16	8.54E-15
WDR77	0.156510108	0.000364	0.000835
WDR78	-0.159949422	0.000268	0.000632
WDR7	-0.17764843	5.04E-05	0.000134
WDR81	-0.28445523	4.83E-11	3.26E-10
WDR82	0.029596865	0.502747	0.575515
WDR83	-0.166361686	0.000149	0.000369
WDR85	-0.046242184	0.294909	0.366241
WDR86	-0.123874599	0.004875	0.009178
WDR87	-0.06670377	0.130596	0.180261
WDR88	-0.017965269	0.6842	0.740954
WDR89	0.185902906	2.18E-05	6.19E-05
WDR8	-0.071934778	0.102973	0.145964
WDR90	-0.263845305	1.19E-09	6.55E-09
WDR91	-0.389221405	4.49E-20	1.18E-18
WDR92	-0.023654397	0.592253	0.658212
WDR93	-0.248405479	1.11E-08	5.26E-08
WDSUB1	-0.03315696	0.452755	0.527033
WDTC1	-0.479239827	6.25E-31	8.01E-29
WDYHV1	0.343727718	9.96E-16	1.37E-14
WEE1	-0.030373482	0.491599	0.564847
WEE2	-0.126192721	0.004127	0.007862
WFDC10A	0.034583525	0.433538	0.508184
WFDC10B	-0.001859094	0.966429	0.974326
WFDC11	-0.030929256	0.483703	0.557441
WFDC12	-0.117950328	0.007372	0.013423
WFDC13	-0.063920146	0.147467	0.200775
WFDC1	-0.189360646	1.52E-05	4.43E-05
WFDC2	-0.365756111	9.55E-18	1.80E-16
WFDC3	0.028818637	0.514049	0.586395
WFDC5	-0.144691255	0.000991	0.00211
WFDC6	-0.230617314	1.21E-07	4.91E-07
WFDC8	-0.049538611	0.261791	0.330743
WFDC9	-0.014718771	0.738962	0.788758
WFIKKN1	-0.206271997	2.35E-06	7.77E-06
WFIKKN2	-0.157915311	0.000321	0.000746
WFS1	-0.218206196	5.73E-07	2.09E-06
WHAMML1	-0.258426463	2.65E-09	1.37E-08
WHAMML2	-0.220531556	4.31E-07	1.61E-06
WHAMM	-0.324969709	3.94E-14	4.26E-13
WHSC1L1	-0.036446674	0.409161	0.483866
WHSC1	0.197225193	6.51E-06	2.00E-05
WHSC2	0.068383981	0.12116	0.168716

WIBG	0.274172574	2.48E-10	1.50E-09
WIF1	-0.256676455	3.42E-09	1.74E-08
WIPF1	-0.108399529	0.013845	0.023865
WIPF2	-0.025074072	0.57022	0.638375
WIPF3	-0.03545308	0.422058	0.496592
WIPI1	-0.041903118	0.342601	0.416615
WIPI2	0.052262235	0.236434	0.303095
WISP1	0.124651976	0.004612	0.008716
WISP2	-0.107029734	0.0151	0.02583
WISP3	0.066387859	0.132432	0.182581
WIT1	0.134955347	0.002146	0.004306
WIZ	-0.0842123	0.056155	0.084929
WLS	-0.305414527	1.40E-12	1.20E-11
WNK1	-0.154022359	0.000452	0.001021
WNK2	-0.003675917	0.933679	0.948147
WNK3	0.022328422	0.613177	0.677099
WNK4	-0.02251874	0.610153	0.674432
WNT10A	-0.247457638	1.26E-08	5.95E-08
WNT10B	-0.088194086	0.045449	0.070138
WNT11	-0.333648729	7.41E-15	8.94E-14
WNT16	0.067988086	0.123334	0.171361
WNT1	-0.116001443	0.008414	0.015144
WNT2B	-0.373802713	1.60E-18	3.36E-17
WNT2	-0.02395123	0.587614	0.654148
WNT3A	-0.269871675	4.80E-10	2.80E-09
WNT3	-0.009269507	0.833783	0.867867
WNT4	-0.194071412	9.18E-06	2.76E-05
WNT5A	-0.244130563	2.00E-08	9.16E-08
WNT5B	-0.056316175	0.201982	0.264476
WNT6	-0.073029672	0.097825	0.139507
WNT7A	-0.084523076	0.05525	0.0837
WNT7B	-0.158995657	0.000292	0.000683
WNT8A	-0.051664395	0.241846	0.309061
WNT8B	-0.036742833	0.405362	0.480113
WNT9A	-0.01206015	0.784828	0.827162
WNT9B	-0.136098595	0.001965	0.003968
WRAP53	0.110217706	0.012322	0.021444
WRB	-0.150002783	0.000637	0.001404
WRNIP1	0.225471376	2.33E-07	9.04E-07
WRN	0.096188521	0.029064	0.046781
WSB1	-0.200848001	4.35E-06	1.38E-05
WSB2	0.311224538	4.98E-13	4.55E-12
WSCD1	-0.10889407	0.013415	0.023174
WSCD2	-0.305610178	1.35E-12	1.16E-11

WT1	0.157716352	0.000327	0.000758
WTAP	0.128742538	0.003426	0.006619
WTIP	-0.250596314	8.15E-09	3.95E-08
WWC1	-0.306609039	1.13E-12	9.85E-12
WWC2	-0.163617001	0.000192	0.000466
WWC3	-0.295361673	7.94E-12	6.04E-11
WWOX	-0.161112678	0.000241	0.000574
WWP1	0.165436619	0.000162	0.000399
WWP2	-0.372973159	1.92E-18	3.96E-17
WWTR1	0.072948096	0.098201	0.139983
XAB2	-0.252571177	6.16E-09	3.03E-08
XAF1	-0.235739198	6.19E-08	2.62E-07
XAGE1D	0.00458135	0.917395	0.935196
XAGE2	-0.047648401	0.280454	0.351103
XAGE3	-0.071879292	0.10324	0.146227
XAGE5	-0.016766241	0.704251	0.759336
XBP1	0.08994548	0.041314	0.064364
XCL1	0.066276759	0.133083	0.183389
XCL2	0.032146129	0.466658	0.540509
XCR1	-0.265504025	9.31E-10	5.21E-09
XDH	-0.02026929	0.646297	0.706684
XG	0.070811184	0.108478	0.152888
XIAP	-0.032960562	0.455438	0.529446
XIRP1	0.191447087	1.22E-05	3.60E-05
XIRP2	0.054406327	0.217729	0.282046
XIST	-0.224295717	2.70E-07	1.04E-06
XKR3	0.007695495	0.861695	0.891304
XKR4	-0.207577589	2.02E-06	6.75E-06
XKR5	-0.070934523	0.107863	0.15209
XKR6	-0.213765149	9.78E-07	3.44E-06
XKR7	-0.039144065	0.375347	0.450165
XKR8	-0.139086372	0.001556	0.003195
XKR9	-0.024012135	0.586664	0.653139
XKRX	-0.16763236	0.000132	0.00033
XKRY2	0.09039776	0.040299	0.062901
XK	-0.05276178	0.231978	0.29807
XPA	-0.235236637	6.61E-08	2.80E-07
XPC	-0.385916469	9.80E-20	2.46E-18
XPNPEP1	0.162001218	0.000223	0.000534
XPNPEP2	-0.119127331	0.0068	0.012461
XPNPEP3	0.063788228	0.148306	0.20178
XPO1	0.276796034	1.64E-10	1.02E-09
XPO4	0.05811547	0.18792	0.248294
XPO5	0.297095738	5.91E-12	4.57E-11

XPO6	0.063949579	0.147281	0.200562
XPO7	0.048272513	0.274195	0.344497
XPOT	0.462596174	1.13E-28	1.03E-26
XPR1	0.041944182	0.342127	0.416293
XRCC1	-0.108008074	0.014194	0.024424
XRCC2	0.373642423	1.65E-18	3.46E-17
XRCC3	0.155796308	0.000387	0.000885
XRCC4	0.215495185	7.95E-07	2.83E-06
XRCC5	0.314715295	2.65E-13	2.53E-12
XRCC6BP1	0.140933144	0.001343	0.002791
XRCC6	0.212517932	1.13E-06	3.94E-06
XRN1	-0.022757802	0.606365	0.671135
XRN2	0.147513619	0.000785	0.001702
XRRA1	0.024107324	0.585181	0.651831
XYLB	0.17127222	9.37E-05	0.00024
XYLT1	-0.01186068	0.788301	0.830079
XYLT2	-0.256772953	3.37E-09	1.72E-08
YAF2	0.252584191	6.15E-09	3.03E-08
YAP1	-0.069510509	0.115137	0.161295
YARS2	0.472270382	5.71E-30	6.28E-28
YARS	0.219676505	4.78E-07	1.78E-06
YBX1	0.275872199	1.90E-10	1.17E-09
YBX2	0.234454881	7.33E-08	3.08E-07
YDJC	0.24676025	1.39E-08	6.52E-08
YEATS2	0.100713236	0.022265	0.036756
YEATS4	0.285990362	3.77E-11	2.58E-10
YES1	0.292586124	1.27E-11	9.33E-11
YIF1A	0.234157038	7.62E-08	3.19E-07
YIF1B	0.16766854	0.000132	0.000329
YIPF1	0.0887176	0.044178	0.068373
YIPF2	0.045993545	0.297516	0.368768
YIPF3	-0.057592839	0.191928	0.252834
YIPF4	0.245982447	1.55E-08	7.21E-08
YIPF5	0.161408301	0.000235	0.000561
YIPF6	0.320377943	9.32E-14	9.48E-13
YIPF7	-0.056200226	0.202914	0.265508
YJEFN3	-0.182919898	2.96E-05	8.22E-05
YKT6	0.334689545	6.04E-15	7.43E-14
YLPM1	-0.011120311	0.801231	0.840727
YME1L1	0.359003515	4.13E-17	7.01E-16
YOD1	-0.139700844	0.001482	0.003057
YPEL1	-0.357858314	5.27E-17	8.81E-16
YPEL2	-0.285351469	4.18E-11	2.84E-10
YPEL3	-0.351565794	1.99E-16	3.03E-15

YPEL4	-0.122403959	0.005411	0.010103
YPEL5	-0.154371252	0.000438	0.000994
YRDC	0.35865869	4.44E-17	7.49E-16
YSK4	-0.23619791	5.82E-08	2.48E-07
YTHDC1	-0.12050115	0.006182	0.011409
YTHDC2	-0.293177382	1.15E-11	8.49E-11
YTHDF1	0.09225986	0.036341	0.057267
YTHDF2	-0.012839724	0.771293	0.816023
YTHDF3	0.31298334	3.62E-13	3.39E-12
YWHAB	0.226862484	1.95E-07	7.68E-07
YWHAE	0.290118489	1.91E-11	1.37E-10
YWHAG	0.431132696	1.00E-24	5.32E-23
YWHAH	0.050229193	0.255191	0.323673
YWHAQ	0.380021736	3.87E-19	8.97E-18
YWHAZ	0.492963963	6.88E-33	1.24E-30
YY1AP1	-0.117197625	0.00776	0.014064
YY1	0.3842561	1.45E-19	3.59E-18
YY2	-0.043303383	0.3267	0.39998
ZACN	-0.013118372	0.766472	0.812099
ZADH2	-0.016703156	0.705312	0.760233
ZAK	0.15421206	0.000444	0.001007
ZAN	0.021766603	0.622139	0.685179
ZAP70	-0.268354478	6.05E-10	3.48E-09
ZAR1L	0.022230032	0.614742	0.678453
ZAR1	-0.051538731	0.242995	0.310311
ZBBX	-0.199807687	4.89E-06	1.53E-05
ZBED1	0.098087955	0.026019	0.042333
ZBED2	-0.049215107	0.264923	0.334235
ZBED3	-0.179834273	4.05E-05	0.00011
ZBED4	0.081942085	0.063145	0.094394
ZBED5	-0.28578578	3.89E-11	2.66E-10
ZBP1	-0.112546283	0.010588	0.018698
ZBTB10	0.201763346	3.93E-06	1.25E-05
ZBTB11	-0.028819501	0.514037	0.586395
ZBTB12	0.054445162	0.2174	0.281711
ZBTB16	-0.294152327	9.73E-12	7.31E-11
ZBTB17	-0.177044175	5.35E-05	0.000142
ZBTB1	0.020873959	0.636494	0.697759
ZBTB20	-0.148083537	0.000749	0.001631
ZBTB22	-0.288505011	2.49E-11	1.75E-10
ZBTB24	-0.139383881	0.00152	0.00313
ZBTB25	0.019081697	0.665727	0.724245
ZBTB26	-0.024157706	0.584397	0.651111
ZBTB2	0.237153065	5.13E-08	2.20E-07

ZBTB32	-0.16509325	0.000168	0.000411
ZBTB33	0.022159607	0.615864	0.679479
ZBTB34	-0.279689628	1.04E-10	6.68E-10
ZBTB37	-0.074177851	0.092648	0.13274
ZBTB38	0.137952524	0.001701	0.003472
ZBTB39	0.046123439	0.296152	0.367488
ZBTB3	-0.271182852	3.93E-10	2.32E-09
ZBTB40	-0.313017747	3.60E-13	3.37E-12
ZBTB41	0.179135443	4.34E-05	0.000117
ZBTB42	-0.059418237	0.178197	0.237216
ZBTB43	-0.341316746	1.62E-15	2.18E-14
ZBTB44	-0.100569891	0.022458	0.03703
ZBTB45	-0.08251141	0.061328	0.09198
ZBTB46	-0.369285411	4.38E-18	8.67E-17
ZBTB47	-0.348614811	3.67E-16	5.41E-15
ZBTB48	-0.3563224	7.31E-17	1.19E-15
ZBTB49	-0.237462341	4.93E-08	2.12E-07
ZBTB4	-0.43071297	1.12E-24	5.89E-23
ZBTB5	-0.050845874	0.249396	0.317329
ZBTB6	0.115329426	0.008803	0.015776
ZBTB7A	-0.164492985	0.000177	0.000433
ZBTB7B	-0.092705611	0.035444	0.055981
ZBTB7C	-0.355359289	8.96E-17	1.43E-15
ZBTB8A	-0.073668973	0.094915	0.135688
ZBTB8B	0.031722152	0.472559	0.546331
ZBTB8OS	0.069401454	0.11571	0.161972
ZBTB9	0.137080756	0.001821	0.003695
ZC3H10	-0.007488042	0.865388	0.894383
ZC3H11A	-0.148724575	0.00071	0.001552
ZC3H12A	-0.082089388	0.062671	0.093748
ZC3H12B	-0.263557088	1.25E-09	6.80E-09
ZC3H12C	-0.065228227	0.139343	0.191041
ZC3H12D	-0.260710725	1.90E-09	1.01E-08
ZC3H13	-0.05646234	0.200812	0.26303
ZC3H14	0.051445695	0.243848	0.3113
ZC3H15	0.501905448	3.26E-34	8.14E-32
ZC3H18	-0.010276558	0.816032	0.853314
ZC3H3	0.075754074	0.085902	0.124195
ZC3H4	-0.207374584	2.07E-06	6.90E-06
ZC3H6	-0.338518886	2.84E-15	3.68E-14
ZC3H7A	-0.207655113	2.01E-06	6.70E-06
ZC3H7B	-0.297686206	5.34E-12	4.16E-11
ZC3H8	0.335443137	5.21E-15	6.46E-14
ZC3HAV1L	0.177492221	5.11E-05	0.000136

ZC3HAV1	0.048813044	0.268851	0.338507
ZC3HC1	0.309681833	6.57E-13	5.92E-12
ZC4H2	-0.072663935	0.099521	0.141683
ZCCHC10	0.043482083	0.324706	0.397783
ZCCHC11	-0.155428801	0.0004	0.000911
ZCCHC12	-0.024868984	0.573379	0.641373
ZCCHC13	-0.034478287	0.434939	0.509558
ZCCHC14	-0.21054432	1.43E-06	4.90E-06
ZCCHC16	-0.09926653	0.024271	0.039723
ZCCHC17	0.093379937	0.034123	0.054067
ZCCHC18	-0.257636608	2.98E-09	1.53E-08
ZCCHC24	-0.295631453	7.58E-12	5.79E-11
ZCCHC2	-0.030793806	0.485621	0.559136
ZCCHC3	-0.086965532	0.048552	0.074473
ZCCHC4	0.130835062	0.002933	0.005737
ZCCHC5	-0.052277999	0.236293	0.302953
ZCCHC6	-0.046252354	0.294803	0.366154
ZCCHC7	0.282138801	7.03E-11	4.62E-10
ZCCHC8	0.181843382	3.31E-05	9.09E-05
ZCCHC9	0.180399792	3.82E-05	0.000104
ZCRB1	0.226081518	2.16E-07	8.41E-07
ZCWPW1	-0.317401904	1.62E-13	1.59E-12
ZCWPW2	-0.291832267	1.44E-11	1.05E-10
ZDBF2	0.029952976	0.497618	0.570648
ZDHHC11	-0.319536919	1.09E-13	1.10E-12
ZDHHC12	0.046310462	0.294196	0.365532
ZDHHC13	-0.040520139	0.358781	0.43339
ZDHHC14	-0.115713059	0.008579	0.015402
ZDHHC15	-0.431239162	9.73E-25	5.20E-23
ZDHHC16	-0.067150623	0.128033	0.177061
ZDHHC17	-0.106163281	0.015944	0.027107
ZDHHC18	0.083298887	0.058886	0.088631
ZDHHC19	-0.119719682	0.006527	0.011997
ZDHHC1	-0.380773787	3.25E-19	7.62E-18
ZDHHC20	0.137133742	0.001813	0.003682
ZDHHC21	-0.077994379	0.076999	0.112769
ZDHHC22	0.01143601	0.795711	0.836383
ZDHHC23	-0.061604698	0.162726	0.21891
ZDHHC24	0.022760673	0.60632	0.671122
ZDHHC2	-0.141050262	0.001331	0.002767
ZDHHC3	-0.168567752	0.000121	0.000304
ZDHHC4	0.059138045	0.180256	0.239494
ZDHHC5	0.048982687	0.267189	0.336775
ZDHHC6	0.210781268	1.39E-06	4.77E-06

ZDHHC7	-0.295550716	7.69E-12	5.87E-11
ZDHHC8P1	-0.337486725	3.48E-15	4.45E-14
ZDHHC8	-0.237803336	4.71E-08	2.03E-07
ZDHHC9	-0.068965751	0.11802	0.164744
ZEB1	-0.183424858	2.81E-05	7.83E-05
ZEB2	-0.22168193	3.74E-07	1.40E-06
ZER1	-0.351597921	1.97E-16	3.01E-15
ZFAND1	0.314463018	2.77E-13	2.63E-12
ZFAND2A	0.271586078	3.69E-10	2.19E-09
ZFAND2B	-0.24357752	2.16E-08	9.82E-08
ZFAND3	-0.051848682	0.240169	0.307192
ZFAND5	-0.128566677	0.00347	0.006699
ZFAND6	0.05667299	0.199134	0.260969
ZFATAS	0.083229417	0.059098	0.0889
ZFAT	0.068069261	0.122886	0.170845
ZFC3H1	-0.095749914	0.029809	0.047899
ZFHX3	-0.168989194	0.000116	0.000293
ZFHX4	0.017841398	0.686261	0.742864
ZFP106	-0.20528126	2.64E-06	8.63E-06
ZFP112	-0.138997084	0.001567	0.003215
ZFP14	-0.246301872	1.48E-08	6.92E-08
ZFP161	-0.133262491	0.002442	0.004848
ZFP1	0.079736082	0.070609	0.104312
ZFP28	-0.153915698	0.000456	0.00103
ZFP2	-0.37379401	1.60E-18	3.37E-17
ZFP30	0.041032647	0.35273	0.42732
ZFP36L1	-0.245264535	1.71E-08	7.91E-08
ZFP36L2	-0.19146002	1.21E-05	3.59E-05
ZFP36	-0.27699676	1.59E-10	9.93E-10
ZFP37	-0.067855928	0.124066	0.172187
ZFP3	-0.400747122	2.74E-21	8.74E-20
ZFP41	-0.047909915	0.27782	0.348373
ZFP42	0.12816034	0.003575	0.006881
ZFP57	0.011134481	0.800983	0.840643
ZFP62	-0.1705598	0.0001	0.000255
ZFP64	0.263285603	1.30E-09	7.06E-09
ZFP82	-0.030000834	0.496931	0.570089
ZFP90	-0.258505576	2.62E-09	1.36E-08
ZFP91-CNTF	0.087600288	0.046927	0.072186
ZFP91	0.12670706	0.003976	0.007597
ZFP92	-0.232106532	9.96E-08	4.10E-07
ZFPL1	0.106762296	0.015356	0.02621
ZFPM1	-0.234886933	6.92E-08	2.92E-07
ZFPM2	-0.152219383	0.000528	0.001179

ZFR2	-0.135003871	0.002138	0.004293
ZFR	0.188356094	1.69E-05	4.88E-05
ZFX	-0.114251465	0.00946	0.01687
ZFYVE16	-0.180709975	3.71E-05	0.000101
ZFYVE19	-0.025722451	0.560288	0.62962
ZFYVE1	-0.149634148	0.000658	0.001446
ZFYVE20	-0.353435827	1.34E-16	2.09E-15
ZFYVE21	-0.065278486	0.139038	0.190688
ZFYVE26	-0.196939452	6.71E-06	2.06E-05
ZFYVE27	-0.182287926	3.16E-05	8.72E-05
ZFYVE28	-0.241644207	2.81E-08	1.25E-07
ZFYVE9	-0.056395098	0.20135	0.263699
ZFY	0.162136186	0.00022	0.000528
ZG16B	0.012919586	0.769911	0.814964
ZG16	0.161262114	0.000238	0.000567
ZGLP1	-0.302779192	2.22E-12	1.83E-11
ZGPAT	-0.143984216	0.00105	0.002225
ZHX1	0.096402269	0.028706	0.046284
ZHX2	-0.133790898	0.002346	0.004671
ZHX3	-0.131612139	0.002767	0.005435
ZIC1	0.029402732	0.505554	0.578354
ZIC2	0.037013228	0.401913	0.476564
ZIC3	0.063378877	0.150933	0.204976
ZIC4	0.062485298	0.156789	0.211893
ZIC5	0.042641166	0.334159	0.407938
ZIK1	-0.139957029	0.001452	0.002999
ZIM2	-0.070241425	0.111357	0.156482
ZIM3	0.003293597	0.940563	0.953201
ZKSCAN1	0.059236041	0.179534	0.238694
ZKSCAN2	-0.079719687	0.070667	0.10439
ZKSCAN3	-0.013696389	0.7565	0.803578
ZKSCAN4	-0.032106771	0.467204	0.540985
ZKSCAN5	0.139549674	0.0015	0.003091
ZMAT1	-0.47437394	2.95E-30	3.44E-28
ZMAT2	-0.03583537	0.417068	0.491849
ZMAT3	-0.100615046	0.022397	0.036943
ZMAT4	0.168290389	0.000124	0.000312
ZMAT5	-0.112116588	0.010891	0.019186
ZMIZ1	-0.254686858	4.55E-09	2.28E-08
ZMIZ2	-0.100455906	0.022611	0.03725
ZMPSTE24	0.219321975	5.00E-07	1.85E-06
ZMYM1	0.010709883	0.808422	0.846805
ZMYM2	0.065905249	0.135275	0.186128
ZMYM3	-0.225416008	2.34E-07	9.10E-07

ZMYM4	-0.056205267	0.202873	0.265486
ZMYM5	-0.088171827	0.045503	0.070217
ZMYM6	-0.339715947	2.23E-15	2.94E-14
ZMYND10	-0.265904932	8.77E-10	4.91E-09
ZMYND11	-0.088705906	0.044206	0.06841
ZMYND12	-0.283016678	6.10E-11	4.05E-10
ZMYND15	-0.415078518	7.25E-23	2.90E-21
ZMYND17	-0.069918448	0.113015	0.158642
ZMYND19	0.180829576	3.66E-05	0.0001
ZMYND8	-0.027965003	0.526597	0.598693
ZNF100	-0.122918029	0.005218	0.009771
ZNF101	-0.062699055	0.155372	0.21022
ZNF107	-0.008493779	0.847515	0.879505
ZNF10	-0.213138039	1.05E-06	3.68E-06
ZNF114	0.049788577	0.259389	0.328268
ZNF117	-0.245825687	1.59E-08	7.36E-08
ZNF121	0.004707104	0.915136	0.933681
ZNF124	0.037658679	0.39375	0.468657
ZNF12	-0.014541171	0.741999	0.791244
ZNF131	0.187699764	1.81E-05	5.21E-05
ZNF132	-0.223160465	3.11E-07	1.19E-06
ZNF133	-0.130150813	0.003086	0.006014
ZNF134	-0.087273355	0.047758	0.073306
ZNF135	-0.146355775	0.000865	0.001859
ZNF136	-0.257497407	3.04E-09	1.56E-08
ZNF137	-0.222606173	3.33E-07	1.27E-06
ZNF138	0.148629641	0.000716	0.001563
ZNF140	0.148275642	0.000737	0.001607
ZNF141	-0.1935252	9.73E-06	2.92E-05
ZNF142	-0.106201065	0.015907	0.027052
ZNF143	0.292841569	1.21E-11	8.96E-11
ZNF146	0.215965419	7.51E-07	2.69E-06
ZNF148	0.107924447	0.01427	0.024537
ZNF14	-0.212041109	1.20E-06	4.15E-06
ZNF154	-0.359703177	3.55E-17	6.11E-16
ZNF155	-0.160305875	0.000259	0.000614
ZNF157	-0.068264579	0.121812	0.16953
ZNF160	-0.325155626	3.80E-14	4.12E-13
ZNF165	0.061742819	0.161784	0.217789
ZNF167	-0.300224625	3.46E-12	2.76E-11
ZNF169	-0.258003338	2.82E-09	1.45E-08
ZNF16	0.111458452	0.011369	0.019953
ZNF174	-0.062370358	0.157554	0.212826
ZNF175	-0.226013969	2.17E-07	8.48E-07

ZNF177	-0.206038038	2.42E-06	7.96E-06
ZNF17	-0.273192186	2.88E-10	1.73E-09
ZNF180	-0.021312659	0.629422	0.691653
ZNF181	-0.145190535	0.000952	0.002031
ZNF182	-0.150040247	0.000635	0.0014
ZNF184	-0.057987123	0.188898	0.249336
ZNF185	0.059863329	0.174962	0.233439
ZNF187	-0.117864881	0.007415	0.013494
ZNF189	-0.20671113	2.24E-06	7.40E-06
ZNF18	-0.193862154	9.39E-06	2.82E-05
ZNF192	-0.109885244	0.012589	0.021863
ZNF193	-0.07737705	0.079374	0.115873
ZNF195	0.055955472	0.204891	0.267688
ZNF197	-0.173507744	7.56E-05	0.000196
ZNF19	-0.28974007	2.03E-11	1.45E-10
ZNF200	0.162379629	0.000215	0.000518
ZNF202	-0.018523876	0.674932	0.732625
ZNF204P	-0.374582074	1.34E-18	2.86E-17
ZNF205	-0.137102331	0.001818	0.00369
ZNF207	0.199090671	5.29E-06	1.65E-05
ZNF208	-0.090086189	0.040996	0.063908
ZNF20	-0.193203391	1.01E-05	3.02E-05
ZNF211	-0.418172764	3.23E-23	1.37E-21
ZNF212	-0.110393051	0.012183	0.021225
ZNF213	-0.222539333	3.36E-07	1.27E-06
ZNF214	-0.251868089	6.81E-09	3.33E-08
ZNF215	0.01248428	0.777456	0.820908
ZNF217	-0.051314132	0.245057	0.312625
ZNF219	-0.272907068	3.01E-10	1.80E-09
ZNF221	-0.031088198	0.481458	0.555293
ZNF222	-0.011536848	0.79395	0.834923
ZNF223	-0.104928891	0.017219	0.029061
ZNF224	-0.356516886	7.01E-17	1.15E-15
ZNF225	-0.180900177	3.64E-05	9.94E-05
ZNF226	-0.17146906	9.20E-05	0.000235
ZNF227	-0.046005779	0.297387	0.368654
ZNF229	0.07222141	0.101605	0.144239
ZNF22	0.141955195	0.001238	0.00259
ZNF230	-0.169972636	0.000106	0.000269
ZNF232	0.04524862	0.305418	0.377159
ZNF233	-0.075616208	0.086475	0.124917
ZNF234	-0.090643265	0.039758	0.062113
ZNF235	-0.113345919	0.010045	0.017828
ZNF236	-0.107792522	0.014389	0.02472

ZNF238	-0.276384752	1.75E-10	1.09E-09
ZNF239	0.218503738	5.52E-07	2.02E-06
ZNF23	-0.232266045	9.76E-08	4.02E-07
ZNF248	-0.213785693	9.76E-07	3.43E-06
ZNF24	-0.02525003	0.567516	0.636097
ZNF250	-0.113877077	0.009698	0.01727
ZNF251	-0.053055177	0.229389	0.295237
ZNF252	0.106750769	0.015367	0.026224
ZNF253	-0.146074638	0.000885	0.001899
ZNF254	-0.131677965	0.002753	0.005414
ZNF256	-0.058852342	0.182373	0.241794
ZNF257	-0.010236139	0.816743	0.853731
ZNF259	0.381184313	2.96E-19	6.99E-18
ZNF25	-0.329425854	1.68E-14	1.92E-13
ZNF260	0.117322297	0.007694	0.013954
ZNF263	-0.164783874	0.000173	0.000422
ZNF264	-0.306062126	1.25E-12	1.08E-11
ZNF266	-0.306632148	1.13E-12	9.82E-12
ZNF267	0.116229313	0.008286	0.014937
ZNF268	0.065056853	0.140387	0.192288
ZNF26	0.04389234	0.320157	0.392885
ZNF271	0.042957288	0.330585	0.404093
ZNF273	0.041213896	0.350605	0.425172
ZNF274	-0.079904484	0.070015	0.10351
ZNF275	-0.136028933	0.001976	0.003988
ZNF276	-0.337243686	3.65E-15	4.64E-14
ZNF277	0.124168679	0.004774	0.009004
ZNF280A	0.258619942	2.58E-09	1.34E-08
ZNF280B	0.053673504	0.223999	0.289156
ZNF280C	0.112125233	0.010885	0.019177
ZNF280D	-0.09095373	0.039081	0.061138
ZNF281	0.133991391	0.002311	0.004605
ZNF282	-0.0486187	0.270764	0.340637
ZNF283	0.037731801	0.392831	0.46771
ZNF284	-0.046027482	0.297159	0.368509
ZNF285	0.009470012	0.830242	0.864992
ZNF286A	0.160735684	0.00025	0.000592
ZNF286B	0.027020919	0.540654	0.611406
ZNF287	-0.217511253	6.23E-07	2.26E-06
ZNF28	0.126970008	0.003901	0.007461
ZNF292	-0.134143997	0.002284	0.004557
ZNF295	-0.190558032	1.34E-05	3.93E-05
ZNF296	-0.072210491	0.101657	0.144282
ZNF2	-0.144404969	0.001015	0.002157

ZNF300	0.150465581	0.000613	0.001353
ZNF302	-0.23482329	6.98E-08	2.94E-07
ZNF304	-0.153676328	0.000466	0.00105
ZNF30	-0.037524786	0.395435	0.470333
ZNF311	-0.159299597	0.000284	0.000667
ZNF317	-0.090879741	0.039241	0.061359
ZNF318	-0.083909408	0.057049	0.086105
ZNF319	-0.205922863	2.45E-06	8.06E-06
ZNF320	-0.078112506	0.076552	0.112228
ZNF321	-0.221884905	3.64E-07	1.37E-06
ZNF322A	-0.104411222	0.017779	0.029941
ZNF322B	-0.099546779	0.023871	0.039116
ZNF323	-0.083979843	0.05684	0.085842
ZNF324B	-0.25634135	3.59E-09	1.82E-08
ZNF324	-0.294198553	9.66E-12	7.26E-11
ZNF326	0.078176254	0.076311	0.111924
ZNF329	-0.107031792	0.015098	0.025828
ZNF32	0.016761289	0.704334	0.759343
ZNF330	0.047367253	0.283305	0.354096
ZNF331	-0.198915899	5.40E-06	1.68E-05
ZNF333	-0.333317765	7.91E-15	9.47E-14
ZNF334	-0.252607618	6.13E-09	3.02E-08
ZNF335	-0.137822919	0.001718	0.003505
ZNF337	-0.340811179	1.79E-15	2.40E-14
ZNF33A	-0.232227256	9.80E-08	4.04E-07
ZNF33B	-0.196270791	7.22E-06	2.21E-05
ZNF341	-0.103122691	0.019243	0.032182
ZNF343	-0.044607474	0.312329	0.384379
ZNF345	-0.361723914	2.30E-17	4.04E-16
ZNF346	-0.216669227	6.90E-07	2.48E-06
ZNF347	-0.210971654	1.36E-06	4.67E-06
ZNF34	-0.125085292	0.00447	0.008467
ZNF350	-0.131252299	0.002843	0.005574
ZNF354A	-0.12981557	0.003164	0.006153
ZNF354B	-0.312723137	3.80E-13	3.54E-12
ZNF354C	-0.179709952	4.10E-05	0.000111
ZNF358	-0.142107618	0.001223	0.002561
ZNF35	0.063719721	0.148743	0.202278
ZNF362	-0.381911078	2.50E-19	5.99E-18
ZNF365	-0.015089963	0.732627	0.783517
ZNF366	-0.311537367	4.71E-13	4.32E-12
ZNF367	0.312643178	3.86E-13	3.59E-12
ZNF37A	-0.103100185	0.01927	0.032221
ZNF37B	-0.317724135	1.52E-13	1.51E-12

ZNF382	-0.144924614	0.000973	0.002073
ZNF383	-0.095649116	0.029982	0.048162
ZNF384	0.17537462	6.30E-05	0.000165
ZNF385A	-0.116929343	0.007902	0.014304
ZNF385B	-0.310053391	6.14E-13	5.56E-12
ZNF385D	-0.154160028	0.000446	0.00101
ZNF389	-0.169481429	0.000111	0.000281
ZNF391	-0.115799875	0.008529	0.015322
ZNF394	-0.117117219	0.007802	0.014139
ZNF395	-0.171231629	9.41E-05	0.00024
ZNF396	-0.274232584	2.45E-10	1.49E-09
ZNF397OS	-0.222451083	3.40E-07	1.29E-06
ZNF397	-0.225665806	2.27E-07	8.83E-07
ZNF398	-0.010510763	0.811917	0.849798
ZNF3	0.111570691	0.011286	0.019818
ZNF404	-0.151044665	0.000583	0.001293
ZNF407	-0.17393397	7.25E-05	0.000188
ZNF408	0.01606193	0.716126	0.768946
ZNF410	0.350351764	2.56E-16	3.83E-15
ZNF414	-0.162651859	0.00021	0.000506
ZNF415	-0.106276334	0.015832	0.026936
ZNF416	-0.042520063	0.335535	0.409418
ZNF417	-0.268520577	5.90E-10	3.39E-09
ZNF418	-0.246101047	1.53E-08	7.10E-08
ZNF419	-0.221808051	3.68E-07	1.39E-06
ZNF41	-0.097440267	0.027025	0.043823
ZNF420	0.029660621	0.501826	0.574682
ZNF423	-0.268761403	5.69E-10	3.28E-09
ZNF425	-0.200937758	4.31E-06	1.37E-05
ZNF426	-0.007793923	0.859944	0.889801
ZNF428	-0.079397326	0.071817	0.105947
ZNF429	-0.277906993	1.38E-10	8.70E-10
ZNF430	-0.078463879	0.075232	0.110472
ZNF431	-0.232367285	9.63E-08	3.98E-07
ZNF432	-0.075877732	0.08539	0.123571
ZNF433	-0.181423997	3.45E-05	9.46E-05
ZNF434	-0.14103541	0.001333	0.002769
ZNF436	-0.081065663	0.066029	0.098257
ZNF438	-0.222172167	3.52E-07	1.33E-06
ZNF439	-0.315610558	2.25E-13	2.17E-12
ZNF43	-0.254354613	4.78E-09	2.39E-08
ZNF440	-0.239726521	3.64E-08	1.60E-07
ZNF441	-0.352142766	1.76E-16	2.70E-15
ZNF442	-0.255894798	3.83E-09	1.94E-08

ZNF443	-0.085371254	0.052842	0.080387
ZNF444	-0.293535246	1.08E-11	8.04E-11
ZNF445	-0.198811861	5.46E-06	1.70E-05
ZNF446	-0.308275482	8.44E-13	7.48E-12
ZNF449	-0.180788804	3.68E-05	0.0001
ZNF44	-0.340820418	1.79E-15	2.39E-14
ZNF451	-0.079246708	0.072359	0.106637
ZNF454	-0.200050842	4.76E-06	1.50E-05
ZNF45	-0.091112488	0.038739	0.06065
ZNF460	0.04328588	0.326896	0.400147
ZNF461	-0.110924419	0.011771	0.020562
ZNF462	-0.316722651	1.83E-13	1.79E-12
ZNF467	-0.087643539	0.046818	0.072051
ZNF468	0.190833365	1.30E-05	3.83E-05
ZNF469	0.004671641	0.915773	0.934092
ZNF470	-0.206956608	2.17E-06	7.21E-06
ZNF471	-0.285089916	4.36E-11	2.96E-10
ZNF473	0.157515648	0.000333	0.00077
ZNF474	-0.211389979	1.30E-06	4.46E-06
ZNF479	0.085892432	0.051406	0.078441
ZNF480	0.099322207	0.024191	0.039601
ZNF483	-0.244412283	1.93E-08	8.84E-08
ZNF484	-0.116122529	0.008345	0.015035
ZNF485	0.196326309	7.18E-06	2.20E-05
ZNF486	-0.128279964	0.003544	0.006827
ZNF487	-0.008284326	0.851231	0.882444
ZNF488	0.116633445	0.008062	0.014576
ZNF48	0.130184992	0.003078	0.006
ZNF490	-0.346035285	6.23E-16	8.87E-15
ZNF491	-0.292582626	1.27E-11	9.34E-11
ZNF492	0.062187112	0.15878	0.21428
ZNF493	-0.37521801	1.16E-18	2.50E-17
ZNF496	-0.025604996	0.562081	0.631492
ZNF497	-0.272509362	3.20E-10	1.91E-09
ZNF498	-0.116532322	0.008118	0.014665
ZNF500	-0.364079837	1.38E-17	2.52E-16
ZNF501	-0.05647732	0.200692	0.262898
ZNF502	-0.160948646	0.000245	0.000582
ZNF503	-0.117503139	0.0076	0.013801
ZNF506	-0.190831591	1.30E-05	3.83E-05
ZNF507	0.015594829	0.72404	0.776026
ZNF510	-0.135255365	0.002097	0.004215
ZNF511	0.248126158	1.15E-08	5.46E-08
ZNF512B	-0.12376502	0.004913	0.009243

ZNF512	-0.300672071	3.20E-12	2.57E-11
ZNF513	-0.193580412	9.68E-06	2.90E-05
ZNF514	-0.272350554	3.28E-10	1.96E-09
ZNF516	-0.179801102	4.06E-05	0.00011
ZNF517	-0.113205435	0.010139	0.01798
ZNF518A	0.05029088	0.254607	0.323138
ZNF518B	0.026722039	0.545143	0.615785
ZNF519	-0.038306948	0.385652	0.460369
ZNF521	-0.24612494	1.52E-08	7.08E-08
ZNF524	-0.099663046	0.023707	0.038872
ZNF525	0.060464071	0.170666	0.228331
ZNF526	0.040935612	0.35387	0.428521
ZNF527	-0.095432953	0.030357	0.048678
ZNF528	-0.175442376	6.26E-05	0.000164
ZNF529	-0.189570625	1.48E-05	4.34E-05
ZNF530	0.020147716	0.648275	0.708498
ZNF532	0.016288486	0.712299	0.765947
ZNF534	0.044238919	0.316347	0.388734
ZNF536	-0.2776472	1.44E-10	9.04E-10
ZNF540	-0.468862612	1.66E-29	1.69E-27
ZNF541	-0.174031036	7.18E-05	0.000187
ZNF542	-0.054579313	0.216268	0.280607
ZNF543	-0.121498911	0.005766	0.010703
ZNF544	0.161694457	0.000229	0.000548
ZNF546	-0.34648494	5.68E-16	8.14E-15
ZNF547	-0.261933508	1.59E-09	8.52E-09
ZNF548	-0.210720808	1.40E-06	4.80E-06
ZNF549	-0.184906093	2.42E-05	6.80E-05
ZNF550	-0.145914926	0.000897	0.001921
ZNF551	-0.067523154	0.125926	0.174405
ZNF552	-0.295481185	7.78E-12	5.93E-11
ZNF554	-0.36283381	1.81E-17	3.23E-16
ZNF555	0.013010281	0.768341	0.813777
ZNF556	0.095388968	0.030434	0.048782
ZNF557	-0.238146663	4.50E-08	1.95E-07
ZNF558	-0.071882912	0.103223	0.146227
ZNF559	-0.202472742	3.63E-06	1.16E-05
ZNF560	0.083842686	0.057247	0.086379
ZNF561	-0.067939268	0.123604	0.171605
ZNF562	0.015532443	0.725099	0.776995
ZNF563	-0.319126061	1.18E-13	1.18E-12
ZNF564	-0.286341679	3.56E-11	2.44E-10
ZNF565	-0.068861532	0.118577	0.165419
ZNF566	-0.125386755	0.004374	0.008299

ZNF567	0.015490039	0.72582	0.777558
ZNF568	-0.187417816	1.86E-05	5.35E-05
ZNF569	-0.043752703	0.321701	0.394609
ZNF570	0.007520095	0.864817	0.893886
ZNF571	-0.211408347	1.29E-06	4.45E-06
ZNF572	0.017065377	0.699228	0.754776
ZNF573	-0.174900437	6.60E-05	0.000173
ZNF574	-0.024845909	0.573734	0.641627
ZNF575	-0.234789324	7.01E-08	2.95E-07
ZNF576	0.098463907	0.02545	0.041489
ZNF577	-0.228202864	1.65E-07	6.54E-07
ZNF578	-0.085978749	0.051171	0.078119
ZNF579	-0.063613754	0.149421	0.203131
ZNF57	-0.116448208	0.008164	0.014739
ZNF580	-0.212228508	1.17E-06	4.07E-06
ZNF581	0.126785802	0.003953	0.007558
ZNF582	-0.188476835	1.67E-05	4.83E-05
ZNF583	-0.09663761	0.028317	0.045719
ZNF584	-0.125852543	0.00423	0.008039
ZNF585A	0.036637134	0.406716	0.481487
ZNF585B	-0.055626278	0.207572	0.270749
ZNF586	-0.155546815	0.000396	0.000903
ZNF587	-0.205314644	2.63E-06	8.60E-06
ZNF589	-0.381964035	2.47E-19	5.93E-18
ZNF592	-0.141678572	0.001266	0.002644
ZNF593	0.094003453	0.03294	0.052358
ZNF594	-0.258481506	2.63E-09	1.36E-08
ZNF595	-0.037976321	0.389769	0.464507
ZNF596	-0.251060956	7.63E-09	3.72E-08
ZNF597	-0.171893125	8.83E-05	0.000226
ZNF598	0.126661824	0.003989	0.00762
ZNF599	-0.267825467	6.56E-10	3.75E-09
ZNF600	-0.125435672	0.004359	0.008272
ZNF605	0.084663008	0.054847	0.083158
ZNF606	-0.163266854	0.000198	0.00048
ZNF607	-0.000327283	0.994088	0.995682
ZNF608	-0.431889519	8.14E-25	4.40E-23
ZNF609	-0.151155692	0.000578	0.001282
ZNF610	0.003154978	0.94306	0.954715
ZNF611	-0.131188357	0.002856	0.005597
ZNF613	0.008413549	0.848938	0.880658
ZNF614	0.074431857	0.091533	0.131397
ZNF615	-0.140732898	0.001365	0.002833
ZNF616	0.030331037	0.492205	0.565446

ZNF618	-0.126855486	0.003933	0.007521
ZNF619	-0.099014593	0.024636	0.040267
ZNF620	-0.047977253	0.277144	0.347634
ZNF621	-0.207407782	2.06E-06	6.87E-06
ZNF622	0.165290265	0.000165	0.000404
ZNF623	0.188322208	1.69E-05	4.90E-05
ZNF624	-0.074549107	0.091022	0.130785
ZNF625	-0.220642577	4.25E-07	1.59E-06
ZNF626	-0.166955119	0.000141	0.00035
ZNF627	0.053316782	0.227097	0.29257
ZNF628	-0.090665088	0.03971	0.062043
ZNF629	-0.274480975	2.36E-10	1.44E-09
ZNF630	-0.082197555	0.062324	0.093307
ZNF638	-0.171357094	9.30E-05	0.000238
ZNF639	0.308641062	7.91E-13	7.04E-12
ZNF641	-0.095246918	0.030683	0.049134
ZNF642	0.029786935	0.500006	0.572992
ZNF643	0.285278817	4.23E-11	2.87E-10
ZNF644	-0.012309507	0.780491	0.823417
ZNF645	-0.002542564	0.954099	0.964623
ZNF646	-0.216405403	7.12E-07	2.56E-06
ZNF648	0.01396145	0.751941	0.799883
ZNF649	-0.022965558	0.603082	0.668167
ZNF652	-0.216321022	7.20E-07	2.58E-06
ZNF653	-0.211085964	1.34E-06	4.62E-06
ZNF654	-0.149124157	0.000686	0.001505
ZNF655	-0.132643748	0.00256	0.005062
ZNF658	-0.332321868	9.60E-15	1.13E-13
ZNF660	-0.263455064	1.26E-09	6.90E-09
ZNF662	-0.329554846	1.64E-14	1.88E-13
ZNF664	0.055385678	0.209548	0.272951
ZNF665	-0.16719505	0.000138	0.000343
ZNF667	-0.111319674	0.011472	0.020119
ZNF668	0.038666837	0.381201	0.455709
ZNF669	0.067054116	0.128583	0.177728
ZNF670	0.125310401	0.004399	0.008341
ZNF671	-0.354739909	1.02E-16	1.62E-15
ZNF672	-0.184052568	2.64E-05	7.38E-05
ZNF673	-0.15512144	0.000411	0.000935
ZNF674	-0.071786376	0.103688	0.146768
ZNF675	-0.016858995	0.702692	0.757982
ZNF676	0.040351513	0.360786	0.435523
ZNF677	-0.137956374	0.0017	0.003471
ZNF678	-0.000958503	0.982688	0.987133

ZNF679	0.087140668	0.048099	0.073807
ZNF680	0.076771279	0.081762	0.118855
ZNF681	0.090320775	0.040471	0.063143
ZNF682	-0.338719907	2.72E-15	3.54E-14
ZNF683	-0.044388	0.314718	0.387017
ZNF684	-0.106755518	0.015363	0.026219
ZNF687	-0.074119735	0.092905	0.133031
ZNF688	-0.263237745	1.31E-09	7.11E-09
ZNF689	-0.022353773	0.612774	0.676729
ZNF691	-0.174213457	7.06E-05	0.000184
ZNF692	-0.183546801	2.78E-05	7.74E-05
ZNF695	0.32678789	2.79E-14	3.08E-13
ZNF696	0.049836785	0.258927	0.327705
ZNF697	0.215267783	8.17E-07	2.91E-06
ZNF699	-0.007901875	0.858025	0.888197
ZNF69	-0.24612236	1.52E-08	7.08E-08
ZNF700	-0.30285975	2.19E-12	1.81E-11
ZNF701	0.02096255	0.635063	0.696496
ZNF702P	0.020170755	0.6479	0.708127
ZNF703	-0.013025952	0.76807	0.813533
ZNF704	-0.283453596	5.68E-11	3.78E-10
ZNF705A	-0.041923196	0.342369	0.416511
ZNF705D	-0.029706991	0.501158	0.574147
ZNF706	0.296973948	6.03E-12	4.66E-11
ZNF707	-0.111481335	0.011352	0.019929
ZNF708	-0.198130401	5.89E-06	1.83E-05
ZNF709	-0.311010144	5.17E-13	4.73E-12
ZNF70	-0.254123754	4.94E-09	2.46E-08
ZNF710	-0.049785606	0.259417	0.328274
ZNF711	-0.069393587	0.115751	0.162019
ZNF713	-0.111601827	0.011263	0.019783
ZNF714	-0.029077472	0.510276	0.582596
ZNF716	0.039999258	0.364997	0.43965
ZNF717	-0.085399457	0.052763	0.080303
ZNF718	0.03030696	0.492549	0.565776
ZNF71	0.02729483	0.536557	0.607459
ZNF720	-0.120180845	0.006322	0.011646
ZNF721	-0.131768559	0.002734	0.005381
ZNF727	-0.098129314	0.025956	0.042237
ZNF732	-0.062579021	0.156166	0.21118
ZNF735	0.012446249	0.778116	0.821475
ZNF737	-0.280546362	9.07E-11	5.88E-10
ZNF738	-0.054222955	0.219286	0.283842
ZNF740	0.024077023	0.585653	0.652256

ZNF746	0.086764619	0.049076	0.075211
ZNF747	-0.139327278	0.001526	0.003142
ZNF749	0.038356403	0.385039	0.459691
ZNF74	-0.030162654	0.494612	0.567917
ZNF750	-0.381158376	2.97E-19	7.03E-18
ZNF75A	-0.072063285	0.102358	0.145143
ZNF75D	-0.304548857	1.63E-12	1.38E-11
ZNF761	0.057252861	0.194569	0.255774
ZNF763	-0.436823608	2.08E-25	1.23E-23
ZNF764	-0.168229987	0.000125	0.000313
ZNF765	0.18337718	2.83E-05	7.86E-05
ZNF766	-0.113077376	0.010225	0.018119
ZNF767	-0.354225672	1.14E-16	1.79E-15
ZNF768	-0.012363589	0.779552	0.822643
ZNF76	-0.159381535	0.000282	0.000663
ZNF770	0.122174814	0.005499	0.010256
ZNF771	0.101424343	0.021333	0.035361
ZNF772	-0.030020598	0.496648	0.56984
ZNF773	-0.1472423	0.000803	0.001738
ZNF774	-0.169512191	0.000111	0.00028
ZNF775	-0.039295001	0.373508	0.448389
ZNF776	-0.257182125	3.18E-09	1.62E-08
ZNF777	0.045834928	0.299187	0.370586
ZNF778	-0.208603574	1.80E-06	6.06E-06
ZNF77	-0.064664671	0.1428	0.195258
ZNF780A	-0.170061571	0.000105	0.000267
ZNF780B	-0.276676583	1.67E-10	1.04E-09
ZNF781	-0.247160129	1.32E-08	6.18E-08
ZNF782	-0.272108584	3.41E-10	2.03E-09
ZNF784	-0.300396205	3.36E-12	2.69E-11
ZNF785	-0.235671365	6.24E-08	2.65E-07
ZNF786	-0.047384952	0.283125	0.353893
ZNF787	-0.039944684	0.365652	0.440333
ZNF788	-0.106089715	0.016018	0.027213
ZNF789	-0.22942365	1.41E-07	5.66E-07
ZNF790	-0.196888419	6.75E-06	2.07E-05
ZNF791	-0.169559842	0.00011	0.000279
ZNF792	-0.105681596	0.016431	0.027873
ZNF793	-0.109634666	0.012793	0.022186
ZNF799	-0.123180811	0.005121	0.009607
ZNF79	-0.219046552	5.17E-07	1.91E-06
ZNF7	0.08188264	0.063337	0.094625
ZNF800	0.097473065	0.026973	0.043754
ZNF804A	-0.13187079	0.002714	0.005345

ZNF804B	0.02618298	0.553285	0.62307
ZNF805	-0.195417541	7.93E-06	2.41E-05
ZNF808	-0.224626769	2.59E-07	9.98E-07
ZNF80	-0.209801942	1.56E-06	5.30E-06
ZNF813	0.167055282	0.00014	0.000347
ZNF814	-0.294977336	8.47E-12	6.42E-11
ZNF815	-0.211507483	1.28E-06	4.41E-06
ZNF816A	-0.060667582	0.169228	0.226727
ZNF81	-0.043423623	0.325357	0.398532
ZNF821	-0.113935108	0.009661	0.017211
ZNF823	-0.072930515	0.098283	0.140079
ZNF826	-0.076188255	0.084114	0.121885
ZNF827	-0.067813897	0.1243	0.172416
ZNF828	0.043507423	0.324424	0.397461
ZNF829	-0.05904506	0.180943	0.2402
ZNF830	-0.163973776	0.000186	0.000452
ZNF831	-0.271785347	3.58E-10	2.12E-09
ZNF833	-0.054808114	0.214345	0.278402
ZNF835	-0.232080076	9.99E-08	4.11E-07
ZNF836	-0.262496579	1.46E-09	7.89E-09
ZNF837	-0.213241712	1.04E-06	3.64E-06
ZNF839	-0.203909257	3.08E-06	9.98E-06
ZNF83	-0.301094863	2.97E-12	2.40E-11
ZNF841	-0.162100195	0.000221	0.00053
ZNF843	-0.210460236	1.45E-06	4.94E-06
ZNF844	-0.174050792	7.17E-05	0.000186
ZNF845	0.086022047	0.051054	0.077958
ZNF846	-0.364820084	1.17E-17	2.16E-16
ZNF84	-0.059900358	0.174695	0.233113
ZNF853	-0.177113088	5.31E-05	0.000141
ZNF85	-0.107769734	0.01441	0.024751
ZNF860	0.041479415	0.347508	0.421838
ZNF862	-0.403520293	1.38E-21	4.62E-20
ZNF876P	0.026112843	0.554349	0.623998
ZNF878	0.030999416	0.482712	0.556459
ZNF879	-0.162874845	0.000206	0.000497
ZNF880	-0.080920025	0.066518	0.098919
ZNF883	0.152356303	0.000522	0.001166
ZNF8	0.022577386	0.609223	0.673664
ZNF90	0.027571705	0.532431	0.603951
ZNF91	-0.219405972	4.95E-07	1.83E-06
ZNF92	0.222394665	3.42E-07	1.30E-06
ZNF93	0.071142773	0.10683	0.150757
ZNF98	0.049289451	0.264201	0.33345

ZNF99	0.018080886	0.682278	0.739273
ZNFX1	-0.165783534	0.000157	0.000387
ZNHIT1	0.121415318	0.0058	0.01076
ZNHIT2	-0.156182685	0.000374	0.000857
ZNHIT3	0.212543782	1.13E-06	3.93E-06
ZNHIT6	-0.006211095	0.888177	0.912416
ZNRD1	0.24918366	9.94E-09	4.77E-08
ZNRF1	0.11299246	0.010282	0.018211
ZNRF2	0.099870742	0.023415	0.038451
ZNRF3	-0.126261946	0.004106	0.007826
ZNRF4	-0.021558719	0.62547	0.688203
ZP1	0.053173985	0.228346	0.294046
ZP2	-0.037153042	0.400136	0.47471
ZP3	0.265681658	9.07E-10	5.08E-09
ZP4	0.137963436	0.001699	0.00347
ZPBP2	-0.020888139	0.636265	0.697547
ZPBP	-0.098799166	0.024952	0.04074
ZPLD1	0.006380737	0.885144	0.910002
ZRANB1	-0.037368043	0.397413	0.472208
ZRANB2	-0.130641257	0.002976	0.005816
ZRANB3	0.328921315	1.85E-14	2.10E-13
ZRSR2	-0.416766876	4.67E-23	1.92E-21
ZSCAN10	-0.034058834	0.440551	0.514864
ZSCAN12P1	-4.04E-06	0.999927	0.999963
ZSCAN12	-0.087030474	0.048384	0.074226
ZSCAN16	-0.009201999	0.834976	0.868793
ZSCAN18	-0.281735333	7.50E-11	4.91E-10
ZSCAN1	-0.152221869	0.000528	0.001178
ZSCAN20	0.029754968	0.500466	0.573388
ZSCAN21	0.0873891	0.047463	0.072919
ZSCAN22	-0.211025635	1.35E-06	4.65E-06
ZSCAN23	-0.188826772	1.61E-05	4.66E-05
ZSCAN29	0.00484922	0.912584	0.931695
ZSCAN2	0.101247677	0.021562	0.035703
ZSCAN4	-0.303610201	1.92E-12	1.60E-11
ZSCAN5A	-0.024621523	0.577201	0.644746
ZSCAN5B	0.016070844	0.715975	0.768825
ZSWIM1	0.010620592	0.809989	0.848135
ZSWIM2	-0.005151317	0.907162	0.927486
ZSWIM3	0.055309711	0.210174	0.273678
ZSWIM4	-0.188148234	1.72E-05	4.99E-05
ZSWIM5	-0.256811387	3.35E-09	1.71E-08
ZSWIM6	-0.160372966	0.000258	0.00061
ZSWIM7	-0.20424972	2.96E-06	9.62E-06

ZUFSP	0.201571131	4.01E-06	1.28E-05
ZW10	0.177448803	5.14E-05	0.000137
ZWILCH	0.488751438	2.81E-32	4.71E-30
ZWINT	0.473542847	3.83E-30	4.37E-28
ZXDA	-0.123436562	0.005029	0.009446
ZXDB	0.139156426	0.001547	0.003179
ZXDC	-0.339450507	2.36E-15	3.09E-14
ZYG11A	0.214886572	8.55E-07	3.03E-06
ZYG11B	-0.16951401	0.000111	0.00028
ZYX	-0.064245419	0.145414	0.198439
ZZEF1	-0.254717287	4.53E-09	2.27E-08
ZZZ3	0.203488396	3.23E-06	1.04E-05
psiTPTE22	-0.023907929	0.58829	0.654645
tAKR	0.008953003	0.83938	0.872512

Table S5. Gene Ontology term annotation of *GNPNAT1* co-expressed genes.

Gene set	Description	Size	Leading edge number	ES	NES	P-value	FDR
GO:0007059	chromosome segregation	262	85	0.74971	2.849	0	0
GO:0034470	ncRNA processing	312	154	0.71009	2.6894	0	0
GO:0006413	translational initiation	184	104	0.66586	2.435	0	0
GO:0032200	telomere organization	136	46	0.67666	2.3491	0	0
GO:0031123	RNA 3'-end processing	111	52	0.68192	2.3313	0	0
GO:0006310	DNA recombination	233	77	0.60539	2.2731	0	0
GO:0009112	nucleobase metabolic process	34	14	0.7619	2.1489	0	0
GO:0033108	mitochondrial respiratory chain complex assembly	68	36	0.68415	2.1399	0	0
GO:0150076	neuroinflammatory response	48	21	-0.57991	-1.6325	0.0031646	0.029177
GO:0061512	protein localization to cilium	43	17	-0.5943	-1.6577	0.0063694	0.030139
GO:0031589	cell-substrate adhesion	316	98	-0.479	-1.6975	0	0.027081
GO:0003007	heart morphogenesis	244	68	-0.50052	-1.7366	0	0.02549
GO:0010959	regulation of metal ion transport	337	128	-0.50996	-1.8346	0	0.013466

ES: Enrichment score; NES: Normalized enrichment score; FDR: false discovery rate.

Table S6. KEGG annotation of *GNPNAT1* co-expressed genes.

Gene set	Description	Size	Leading edge number	ES	NES	P-value	FDR
hsa04110	Cell cycle	118	55	0.76543	2.6389	0	0
hsa03010	Ribosome	131	89	0.74412	2.6123	0	0
hsa03050	Proteasome	44	32	0.86007	2.5697	0	0
hsa03013	RNA transport	158	69	0.67655	2.4154	0	0
hsa03008	Ribosome biogenesis in eukaryotes	70	39	0.74878	2.4056	0	0
hsa03040	Spliceosome	115	58	0.68504	2.3566	0	0
hsa00970	Aminoacyl-tRNA biosynthesis	43	18	0.77167	2.2124	0	0
hsa00240	Pyrimidine metabolism	96	46	0.65944	2.1756	0	0
hsa03440	Homologous recombination	34	14	0.75421	2.1113	0	0
hsa00190	Oxidative phosphorylation	104	51	0.57244	1.9409	0	0.000248
hsa03060	Protein export	22	13	0.77027	1.9233	0	0.000351
hsa01200	Carbon metabolism	110	44	0.55506	1.9035	0	0.000441
hsa03022	Basal transcription factors	44	25	0.6588	1.8791	0	0.001092
hsa03420	Nucleotide excision repair	45	17	0.61686	1.8175	0	0.002235
hsa04115	p53 signaling pathway	69	15	0.5676	1.8235	0	0.002245
hsa04973	Carbohydrate digestion and absorption	41	12	-0.6892	-1.8584	0	0.002294
hsa04141	Protein processing in endoplasmic reticulum	159	62	0.50812	1.8241	0	0.002347
hsa00270	Cysteine and methionine metabolism	44	14	0.62552	1.8072	0	0.002368
hsa05320	Autoimmune thyroid disease	48	19	-0.61176	-1.7008	0	0.010171
hsa04658	Th1 and Th2 cell differentiation	90	34	-0.55284	-1.6925	0	0.010274
hsa02010	ABC transporters	44	15	-0.60645	-1.6961	0.003021	0.010288
hsa04971	Gastric acid secretion	75	24	-0.55232	-1.6554	0	0.013878

KEGG: Kyoto Encyclopedia of Genes and Genomes; ES: Enrichment score; NES: Normalized enrichment score; FDR: false discovery rate.

Table S7. Kinases enrichment of *GNPNAT1* co-expressed genes.

Gene Set	Description	Size	Leading Edge Number	ES	NES	P-value	FDR
Kinase_CDK1	cyclin dependent kinase 1	258	84	0.67047	2.5367	0	0
Kinase_PLK1	polo like kinase 1	90	30	0.74433	2.4679	0	0
Kinase_AURKB	aurora kinase B	87	34	0.6792	2.2754	0	0
Kinase_CDK2	cyclin dependent kinase 2	278	90	0.57619	2.1616	0	0
Kinase_ATM	ATM serine/threonine kinase	123	38	0.6072	2.0821	0	0
Kinase_ATR	ATR serine/threonine kinase	66	24	0.65819	2.0789	0	0
Kinase_CHEK1	checkpoint kinase 1	130	40	0.56538	1.9821	0	0.00066623
Kinase_AURKA	aurora kinase A	46	14	0.68672	1.9916	0	0.00076141
Kinase_PLK3	polo like kinase 3	20	11	0.77708	1.8952	0	0.0020727
Kinase_CSNK2A1	casein kinase 2 alpha 1	256	78	0.49962	1.8711	0	0.0042639
Kinase_TTK	TTK protein kinase	12	8	0.84184	1.8412	0	0.0067835
Kinase_CHEK2	checkpoint kinase 2	27	10	0.70356	1.8163	0.0052356	0.0082169
Kinase_SYK	spleen associated tyrosine kinase	35	19	-0.65904	-1.778	0	0.010932
Kinase_WEE1	WEE1 G2 checkpoint kinase	5	5	0.9652	1.7038	0	0.03769
Kinase_PLK4	polo like kinase 4	5	2	0.90366	1.7081	0.0042735	0.038949
Kinase_CDC7	cell division cycle 7	4	3	0.95485	1.6884	0	0.042994
Kinase_STK3	serine/threonine kinase 3	7	3	0.83503	1.6498	0.02551	0.059961
Kinase_RPS6KA4	ribosomal protein S6 kinase A4	19	13	0.66566	1.6415	0.015544	0.063958
Kinase_NEK1	NIMA related kinase 1	4	3	0.98045	1.6215	0	0.069555
Kinase_BUB1	BUB1 mitotic checkpoint serine/threonine kinase	5	3	0.94506	1.6154	0.0045045	0.070938
Kinase_EIF2AK4	eukaryotic translation initiation factor 2 alpha kinase 4	4	2	0.96894	1.6221	0	0.072514
Kinase_STK38	serine/threonine kinase 38	4	1	0.95461	1.6063	0.0093897	0.072532
Kinase_EIF2AK3	eukaryotic translation initiation factor 2 alpha kinase 3	3	1	0.98673	1.5761	0	0.095015
Kinase_HCK	HCK proto-oncogene, Src family tyrosine kinase	23	12	-0.68051	-1.648	0.0034602	0.11712
Kinase_NTRK2	neurotrophic receptor tyrosine kinase 2	11	5	-0.70097	-1.4497	0.045139	0.27533
Kinase_SGK1	serum/glucocorticoid regulated	21	11	-0.60539	-1.4341	0.045603	0.28713

	kinase 1						
Kinase_ERBB4	erb-b2 receptor tyrosine kinase 4	8	5	-0.76061	-1.4528	0.049822	0.28864
Kinase_TGFBR1	transforming growth factor beta receptor 1	8	2	-0.74807	-1.4398	0.052632	0.28924
Kinase_IGF1R	insulin like growth factor 1 receptor	11	5	-0.68588	-1.4263	0.064516	0.29378
Kinase_PIK3CD	phosphatidylinositol-4,5-bisphosphate 3-kinase catalytic subunit delta	8	2	-0.7832	-1.4877	0.030405	0.29906
Kinase_PIK3CA	phosphatidylinositol-4,5-bisphosphate 3-kinase catalytic subunit alpha	10	2	-0.73312	-1.4549	0.03125	0.30708
Kinase_NTRK1	neurotrophic receptor tyrosine kinase 1	12	5	-0.6878	-1.4632	0.056667	0.31061
Kinase_FGFR2	fibroblast growth factor receptor 2	3	1	-0.94093	-1.4131	0.013699	0.31242
Kinase_MARK2	microtubule affinity regulating kinase 2	15	9	-0.67924	-1.4945	0.044828	0.31545
Kinase_MAPK10	mitogen-activated protein kinase 10	25	10	-0.55941	-1.4065	0.055738	0.31597
Kinase_FYN	FYN proto-oncogene, Src family tyrosine kinase	66	27	-0.48442	-1.4709	0.0065359	0.32135
Kinase_BTK	Bruton tyrosine kinase	9	6	-0.77359	-1.5312	0.023333	0.34942
Kinase_FGR	FGR proto-oncogene, Src family tyrosine kinase	12	8	-0.73717	-1.5501	0.0096463	0.3571
Kinase_PDGFRB	platelet derived growth factor receptor beta	15	7	-0.68585	-1.4968	0.035587	0.361
Kinase_IKBKB	inhibitor of nuclear factor kappa B kinase subunit beta	28	12	-0.58196	-1.5098	0.026846	0.37448
Kinase_FES	FES proto-oncogene, tyrosine kinase	7	2	-0.7306	-1.3393	0.11888	0.38214
Kinase_TEC	tec protein tyrosine kinase	5	5	-0.79491	-1.3565	0.066225	0.38953
Kinase_STK39	serine/threonine kinase 39	8	3	-0.69023	-1.3394	0.11348	0.39684
Kinase_PRKAA2	protein kinase AMP-activated catalytic subunit alpha 2	19	10	-0.5789	-1.3458	0.099379	0.40622
Kinase_ULK1	unc-51 like autophagy activating	13	4	-0.61895	-1.3406	0.13423	0.40871

kinase 1

ES: Enrichment score; NES: Normalized enrichment score; FDR: false discovery rate.

Table S8. miRNA enrichment of *GNPNAT1* co-expressed genes.

Gene Set	Size	Leading Edge Number	ES	NES	P Value	FDR
GGGGCCC,MIR-296	67	27	-0.53575	-1.5696	0.003236	0.099967
CCTGTGA,MIR-513	118	47	-0.49612	-1.579	0	0.11175
AGCGCTT,MIR-518F,MIR-518E,MIR-518A	16	7	-0.6863	-1.5396	0.021277	0.12257
GAGCCTG,MIR-484	98	40	-0.52405	-1.6427	0	0.13212
CCCAGAG,MIR-326	141	49	-0.48085	-1.5866	0	0.13432
ATGCTGG,MIR-338	104	29	-0.47556	-1.5142	0.008876	0.14281
GGCCAGT,MIR-193A,MIR-193B	83	18	-0.46871	-1.4438	0.019293	0.14694
AGCTCCT,MIR-28	83	26	-0.4777	-1.4704	0.012539	0.14993
GAGCTGG,MIR-337	147	57	-0.44985	-1.4901	0.002959	0.15046
ACAGGGT,MIR-10A,MIR-10B	119	33	-0.44766	-1.4478	0.015244	0.15099
AGGGCAG,MIR-18A	130	43	-0.43821	-1.4333	0.003135	0.15235
CTTTGCA,MIR-527	222	63	-0.41297	-1.4271	0	0.15362
CTCTATG,MIR-368	37	9	-0.53838	-1.4211	0.02589	0.15377
GCACCTT,MIR-18A,MIR-18B	112	39	-0.46746	-1.4981	0	0.15469
CAGCACT,MIR-512-3P	143	51	-0.4443	-1.4738	0	0.16008
CCAGGGG,MIR-331	83	28	-0.47059	-1.4481	0.006452	0.1621
AGCGCAG,MIR-191	12	7	-0.68025	-1.4095	0.063091	0.16341
TCTCTCC,MIR-185	114	39	-0.45045	-1.4532	0.00304	0.16625
CAGGGTC,MIR-504	79	29	-0.52202	-1.5925	0.009146	0.18386
GTGCAAA,MIR-507	119	51	-0.43213	-1.3813	0.011905	0.20731
GTGCCAA,MIR-96	284	112	-0.3795	-1.3413	0.01061	0.25597
TTTGCAC,MIR-19A,MIR-19B	479	141	-0.36687	-1.3419	0.002639	0.26548
TACTTGA,MIR-26A,MIR-26B	285	69	0.38921	1.4956	0	0.71897
ATGCACG,MIR-517B	15	5	0.28921	0.66046	0.94059	0.98373
GCTTGAA,MIR-498	103	15	0.26173	0.87707	0.81609	0.99454
AAGGGAT,MIR-188	70	9	0.24721	0.7806	0.92857	0.99566
GTAAACC,MIR-299-5P	47	9	0.22451	0.67417	0.97549	0.9979
ATTCTTT,MIR-186	252	61	0.32798	1.2361	0.014085	1
AGTCTTA,MIR-499	68	20	0.40477	1.2974	0.078652	1
ATCATGA,MIR-433	106	32	0.36913	1.2244	0.10119	1
CTACTAG,MIR-325	16	2	0.52349	1.2443	0.17	1

GACTGTT,MIR-212,MIR-132	148	27	0.31388	1.1232	0.17647	1
ATTACAT,MIR-380-3P	95	19	0.33349	1.1142	0.20879	1
TTTTGAG,MIR-373	210	47	0.29139	1.0753	0.22656	1
AGTCTAG,MIR-151	21	6	0.47234	1.1714	0.22749	1
GAGACTG,MIR-452	91	23	0.34122	1.1031	0.24859	1
GTTTGTT,MIR-495	236	40	0.28087	1.0457	0.26761	1
AGTCAGC,MIR-345	55	7	0.35046	1.075	0.26961	1
CTGTTAC,MIR-194	97	15	0.31593	1.0553	0.28962	1
GGGATGC,MIR-324-5P	46	5	0.36286	1.075	0.30151	1
CAGGTCC,MIR-492	59	11	0.33185	1.044	0.30769	1
TATCTGG,MIR-488	56	12	0.34901	1.0577	0.32836	1
TGTTTAC,MIR-30A-5P,MIR-30C,MIR-30D,MIR-30B,MIR-30E-5P	545	108	0.25394	1.0193	0.34513	1

ES: Enrichment score; NES: Normalized enrichment score; FDR: false discovery rate.

Table S9. Transcription factor enrichment of *GNPNAT1* co-expressed genes.

Gene Set	Size	Leading Edge Number	ES	NES	P Value	FDR
V$E2F1_Q6	213	85	0.58802	2.1908	0	0
V$E2F_Q6	211	81	0.58691	2.1879	0	0
V$E2F_Q4	212	81	0.58132	2.1486	0	0
V$E2F4DP1_01	220	82	0.57572	2.147	0	0
V$E2F1DP1_01	218	82	0.57679	2.1346	0	0
V$E2F1DP2_01	218	82	0.57679	2.1346	0	0
V$E2F4DP2_01	218	82	0.57679	2.1346	0	0
V$E2F_02	218	82	0.57647	2.1316	0	0
V$ELK1_02	226	71	0.56863	2.1061	0	0
V$E2F_Q4_01	215	77	0.55898	2.0761	0	0
SGCGSSAAA_V$E2F1DP2_01	155	62	0.58061	2.0718	0	0
V$E2F_Q3	204	63	0.55659	2.0536	0	0
V$E2F1_Q3	225	75	0.54693	2.0251	0	0
V$E2F_Q6_01	219	64	0.53911	2.0245	0	0
V$E2F1DP1RB_01	210	61	0.54487	2.0224	0	0
V$E2F_Q3_01	215	68	0.53792	2.0191	0	0
SCGGAAGY_V$ELK1_02	1076	335	0.47868	1.9809	0	0
V$E2F1_Q6_01	218	65	0.52208	1.9579	0	0
V$E2F1_Q4_01	210	66	0.52356	1.9547	0	0
V$GABP_B	242	82	0.50985	1.9044	0	0
KRCTCNNNNMANAGC_UNKNOWN	63	36	0.543	1.7574	0	0.000696
TGTYNNNNNRGCARM_UNKNOWN	81	36	-0.61058	-1.8355	0	0.006511
V$PPARA_01	36	7	-0.6473	-1.7538	0.003115	0.007054
V$GATA_Q6	185	54	-0.51439	-1.761	0	0.007235
V$RP58_01	194	62	-0.499	-1.708	0	0.008372
V$LMO2COM_02	231	70	-0.49473	-1.7245	0	0.008682
V$DBP_Q6	227	55	-0.49461	-1.7136	0	0.009043
V$MYOGENIN_Q6	235	69	-0.48486	-1.6961	0	0.009496
V$MEF2_02	207	56	-0.51582	-1.7669	0	0.010852
V$GATA3_01	227	69	-0.48082	-1.6586	0	0.011069
V$LHX3_01	209	69	-0.49163	-1.6742	0	0.011334

GATAAGR_V$GATA_C	277	75	-0.46642	-1.6531	0	0.011641
V$GATA1_04	233	65	-0.46829	-1.6351	0	0.015012
V$CHX10_01	208	72	-0.46828	-1.5957	0	0.016564
V$STAT5B_01	226	85	-0.45942	-1.5992	0	0.017243
TGCCAAR_V$NF1_Q6	660	221	-0.42193	-1.5823	0	0.017659
V$ELF1_Q6	220	87	-0.4586	-1.5891	0	0.017689
GCANCTGNY_V$MYOD_Q6	854	246	-0.41636	-1.5797	0	0.017835
TGTTTGY_V$HNF3_Q6	670	212	-0.41815	-1.5764	0	0.018087
V$GATA1_03	233	60	-0.45754	-1.5999	0	0.018129
TGGNNNNNNKCCAR_UNKNOWN	387	127	-0.43491	-1.5737	0	0.018231

ES: Enrichment score; NES: Normalized enrichment score; FDR: false discovery rate.

Table S10. Correlation of *GNPNAT1* with various immune signatures in TCGA-LUAD cohort.

Marker gene	Class	None adjusted		Tumor purity adjusted	
		Correlation coefficient	P-value	Correlation coefficient	P-value
CCL1	chemokine	0.00726001	0.869449575	0.026240141	0.561074131
CCL2	chemokine	-0.010385082	0.814124382	0.00954098	0.832644447
CCL3	chemokine	0.08135977	0.06504917	0.099139627	0.027729363
CCL4	chemokine	0.118895541	0.006908972	0.143692075	0.001379056
CCL5	chemokine	-0.006329612	0.886057864	0.002025595	0.964217788
CCL7	chemokine	0.229806297	1.34066E-07	0.244728004	3.71349E-08
CCL8	chemokine	0.210411881	1.45414E-06	0.234422643	1.39915E-07
CCL11	chemokine	0.142288335	0.001205068	0.162478444	0.000291715
CCL13	chemokine	-0.118175487	0.007258943	-0.098556383	0.028663677
CCL14	chemokine	-0.350637739	2.41068E-16	-0.364383658	6.31456E-17
CCL15	chemokine	-0.033324942	0.450467858	-0.036831128	0.414508865
CCL16	chemokine	-0.295335243	7.9706E-12	-0.299076519	1.2059E-11
CCL17	chemokine	-0.324823736	4.04763E-14	-0.322871423	2.00552E-13
CCL18	chemokine	0.001151238	0.979207669	0.013760089	0.760547108
CCL19	chemokine	-0.257321049	3.11497E-09	-0.268727387	1.33228E-09
CCL20	chemokine	0.198770888	5.48569E-06	0.202417602	5.89927E-06
CCL21	chemokine	0.034893517	0.429424738	0.060456904	0.180186929
CCL22	chemokine	-0.151805229	0.000546851	-0.136603105	0.002368592
CCL23	chemokine	-0.210853136	1.38068E-06	-0.216373758	1.2368E-06
CCL24	chemokine	0.119992733	0.006404714	0.136747	0.002343299
CCL25	chemokine	0.068744928	0.119203957	0.06909134	0.125519058
CCL26	chemokine	0.283871586	5.31326E-11	0.305921866	3.85338E-12
CCL27	chemokine	-0.070380743	0.110647187	-0.078117715	0.083141814
CCL28	chemokine	-0.124816893	0.004557323	-0.12687554	0.004782156
CX3CL1	chemokine	-0.234032114	7.74709E-08	-0.215322073	1.39648E-06
CXCL1	chemokine	0.054878662	0.213755123	0.058421457	0.195327028
CXCL2	chemokine	-0.09518732	0.030788063	-0.094465995	0.036006669
CXCL3	chemokine	0.063423511	0.150644576	0.063758311	0.157506085
CXCL5	chemokine	0.22390853	2.83233E-07	0.233329045	1.60495E-07
CXCL6	chemokine	0.067032322	0.128707584	0.08269717	0.066556432

CXCL9	chemokine	0.108025722	0.01417814	0.136052568	0.00246768
CXCL10	chemokine	0.170026307	0.000105578	0.202906563	5.595E-06
CXCL11	chemokine	0.11115271	0.011597741	0.142961375	0.001459783
CXCL12	chemokine	-0.104803372	0.017352947	-0.106300582	0.018227825
CXCL13	chemokine	-0.049925633	0.25807815	-0.0438504	0.331233278
CXCL14	chemokine	-0.208097062	1.90508E-06	-0.204598539	4.65357E-06
CXCL16	chemokine	-0.239951185	3.53032E-08	-0.24848784	2.25438E-08
CXCL17	chemokine	-0.298408407	4.72354E-12	-0.30268508	6.63351E-12
XCL1	chemokine	0.060958665	0.167188085	0.059455867	0.187520548
XCL2	chemokine	-0.002366917	0.957267043	0.005028703	0.911320885
CCR1	receptor	0.046578486	0.291407824	0.070124247	0.119952443
CCR2	receptor	-0.126005908	0.004183227	-0.10866265	0.01579021
CCR3	receptor	-0.061571912	0.162950169	-0.065222955	0.148163591
CCR4	receptor	-0.165325115	0.000164119	-0.15938535	0.000381325
CCR5	receptor	-0.042019262	0.34126331	-0.020718103	0.646307371
CCR6	receptor	-0.292803364	1.22084E-11	-0.310366219	1.80781E-12
CCR7	receptor	-0.234376194	7.40532E-08	-0.254513392	9.95942E-09
CCR8	receptor	0.024334179	0.58165415	0.051650028	0.252346001
CCR9	receptor	-0.143919863	0.001055904	-0.136089473	0.002460921
CCR10	receptor	0.055566207	0.208064074	0.048477536	0.282699296
CXCR1	receptor	0.005255914	0.905285921	0.021328476	0.636626583
CXCR2	receptor	-0.123412987	0.005037755	-0.108151298	0.016292214
CXCR3	receptor	-0.05976949	0.175640467	-0.049466377	0.272984733
CXCR4	receptor	-0.116377952	0.008202789	-0.126882409	0.004779861
CXCR5	receptor	-0.173059991	7.8926E-05	-0.18290316	4.40175E-05
CXCR6	receptor	-0.012282814	0.780955099	0.000276221	0.995118937
XCR1	receptor	-0.146952422	0.000822961	-0.142056286	0.001565789
CX3CR1	receptor	-0.348212224	3.98246E-16	-0.344080593	3.78787E-15
B2M	MHC	-0.056671804	0.19914373	-0.031129613	0.490444406
HLA-A	MHC	-0.104668387	0.017498539	-0.111300147	0.013410492
HLA-B	MHC	-0.130606423	0.002983253	-0.125640271	0.005211473
HLA-C	MHC	-0.116810679	0.007966078	-0.11528808	0.010411324
HLA-DMA	MHC	-0.412114548	1.55898E-22	-0.42592914	3.80215E-23

HLA-DMB	MHC	-0.272775786	3.07419E-10	-0.277879857	3.42206E-10
HLA-DOA	MHC	-0.311670172	4.59523E-13	-0.323608459	1.75608E-13
HLA-DOB	MHC	-0.305610234	1.35236E-12	-0.337938396	1.23419E-14
HLA-DPA1	MHC	-0.322698168	6.04266E-14	-0.332833127	3.22971E-14
HLA-DPB1	MHC	-0.406600879	6.34826E-22	-0.42746317	2.55772E-23
HLA-DQA1	MHC	-0.255072648	4.3094E-09	-0.269006083	1.27921E-09
HLA-DQA2	MHC	-0.166544176	0.000146544	-0.168321656	0.000173561
HLA-DQB1	MHC	-0.316720154	1.83382E-13	-0.329182705	6.35557E-14
HLA-DRA	MHC	-0.313277459	3.43679E-13	-0.328215756	7.59228E-14
HLA-DRB1	MHC	-0.373626478	1.66056E-18	-0.394736916	7.8592E-20
HLA-E	MHC	-0.194520555	8.74078E-06	-0.197130285	1.03738E-05
HLA-F	MHC	-0.205298348	2.63017E-06	-0.209536436	2.69427E-06
HLA-G	MHC	-0.04930086	0.264090483	-0.039689348	0.379208259
TAP1	MHC	0.173871586	7.29548E-05	0.200178651	7.5058E-06
TAP2	MHC	0.185187177	2.34746E-05	0.206324227	3.85026E-06
TAPBP	MHC	-0.023477206	0.595029902	-0.02848124	0.528100111
CD160	Immunoinhibitor	-0.134322425	0.002252797	-0.129137991	0.004077731
CD96	Immunoinhibitor	-0.104191955	0.018020976	-0.099478154	0.027199168
CSF1R	Immunoinhibitor	-0.132456897	0.00259616	-0.122263442	0.006567752
IDO1	Immunoinhibitor	0.018820465	0.670031894	0.030207154	0.503394812
IL10	Immunoinhibitor	-0.048486718	0.272068667	-0.029773772	0.509540652
IL10RB	Immunoinhibitor	-0.016138636	0.714829421	-0.010172175	0.821752567
KIR2DL1	Immunoinhibitor	-0.006249376	0.887492582	-0.010350957	0.818673629
KIR2DL3	Immunoinhibitor	0.073300603	0.096583122	0.081901626	0.069225006
LAG3	Immunoinhibitor	0.023124209	0.600579954	0.035102602	0.436766263
LGALS9	Immunoinhibitor	-0.28253979	6.58678E-11	-0.282669134	1.64655E-10
TGFB1	Immunoinhibitor	-0.141645182	0.001269026	-0.132281618	0.003254539
TGFBR1	Immunoinhibitor	0.231506362	1.07658E-07	0.243138268	4.57476E-08
TIGIT	Immunoinhibitor	-0.00085192	0.984612826	0.021730387	0.630285567
BTNL2	Immunostimulator	-0.047216059	0.284846319	-0.042875153	0.342110617
CD27	Immunostimulator	-0.149323133	0.00067503	-0.158832393	0.000399828
CD28	Immunostimulator	-0.107557417	0.014605216	-0.096985809	0.031315074
CD40	Immunostimulator	-0.15948441	0.000279313	-0.156506711	0.000487181

CD40LG	Immunostimulator	-0.290524358	1.78556E-11	-0.304475474	4.91607E-12
CD48	Immunostimulator	-0.203917499	3.07882E-06	-0.214222934	1.5844E-06
CD70	Immunostimulator	0.00271091	0.951063775	0.020035481	0.657205151
CXCL12	Immunostimulator	-0.104803372	0.017352947	-0.106300582	0.018227825
CXCR4	Immunostimulator	-0.116377952	0.008202789	-0.126882409	0.004779861
ICOS	Immunostimulator	-0.057842547	0.190005066	-0.046130139	0.306686479
IL6	Immunostimulator	0.211213641	1.32332E-06	0.212190585	1.99753E-06
IL6R	Immunostimulator	-0.170211328	0.000103735	-0.16877885	0.000166529
LTA	Immunostimulator	-0.128307552	0.003536979	-0.126828771	0.004797811
MICB	Immunostimulator	0.131802423	0.0027275	0.145087022	0.001236233
RAET1E	Immunostimulator	0.197819552	6.0939E-06	0.206883316	3.61974E-06
TMIGD2	Immunostimulator	-0.041161112	0.351223025	-0.045053752	0.31812263
ULBP1	Immunostimulator	-0.023284016	0.45538838	-0.031559248	0.321942881
ADRM1	Activated CD8 T cell	0.185338333	2.31108E-05	0.170378475	0.000143973
AHSA1	Activated CD8 T cell	0.459213252	3.15644E-28	0.455829198	1.14998E-26
C1GALT1C1	Activated CD8 T cell	0.211208765	1.32408E-06	0.231995882	1.89546E-07
CCT6B	Activated CD8 T cell	-0.310151836	6.03639E-13	-0.302812113	6.49442E-12
CD37	Activated CD8 T cell	-0.306266584	1.20459E-12	-0.34250078	5.14548E-15
CD3D	Activated CD8 T cell	-0.067648935	0.125220413	-0.064485389	0.152814126
CD3E	Activated CD8 T cell	-0.113400541	0.010008738	-0.118441174	0.008477977
CD3G	Activated CD8 T cell	0.011751079	0.790211786	0.038171111	0.397724027
CD69	Activated CD8 T cell	-0.226452252	2.05649E-07	-0.246576931	2.90826E-08
CD8A	Activated CD8 T cell	0.049499497	0.262168594	0.06581037	0.14453753
CETN3	Activated CD8 T cell	-0.09067163	0.039695335	-0.092941202	0.039126127
CSE1L	Activated CD8 T cell	0.513339269	5.76963E-36	0.510044763	5.26621E-34
GEMIN6	Activated CD8 T cell	0.30490375	1.53124E-12	0.304648254	4.77544E-12
GNLY	Activated CD8 T cell	0.085496592	0.052493604	0.098735229	0.028374347
GPT2	Activated CD8 T cell	0.247779409	1.20906E-08	0.234312349	1.41869E-07
GZMA	Activated CD8 T cell	0.060100524	0.173256264	0.07955218	0.077621122
GZMH	Activated CD8 T cell	0.012403394	0.778860244	0.022812473	0.613348779
GZMK	Activated CD8 T cell	-0.094990307	0.031137534	-0.089499189	0.047016962
IL2RB	Activated CD8 T cell	0.08831152	0.045160901	0.117980016	0.008739105
LCK	Activated CD8 T cell	-0.070965642	0.107707771	-0.062814737	0.163756356

MPZL1	Activated CD8 T cell	0.210065473	1.51441E-06	0.231565983	1.99951E-07
NKG7	Activated CD8 T cell	0.038752195	0.380149963	0.053705871	0.233930068
PIK3IP1	Activated CD8 T cell	-0.411361772	1.89133E-22	-0.414205067	7.36456E-22
PTRH2	Activated CD8 T cell	0.363665176	1.50841E-17	0.358683705	2.0535E-16
TIMM13	Activated CD8 T cell	0.129903419	0.003143579	0.122315947	0.006544444
ZAP70	Activated CD8 T cell	-0.206287842	2.3479E-06	-0.226843288	3.57194E-07
ACTN4	Central memory CD8 T cell	0.122436494	0.005398356	0.123536168	0.006023254
ADAM12	Central memory CD8 T cell	0.330360349	1.40421E-14	0.360035835	1.55572E-16
ADCY9	Central memory CD8 T cell	-0.241765242	2.76308E-08	-0.238435492	8.40743E-08
F13A1	Central memory CD8 T cell	-0.06178789	0.16147702	-0.037147231	0.410512124
FCER1G	Central memory CD8 T cell	-0.024607404	0.577419277	-0.012481505	0.782208867
FCGR3B	Central memory CD8 T cell	0.137622661	0.001745243	0.154690361	0.000567409
FGF7	Central memory CD8 T cell	-0.023430205	0.595767547	-0.013167559	0.770563622
FKBP4	Central memory CD8 T cell	0.44842168	7.64734E-27	0.450483699	5.19688E-26
GLUD1	Central memory CD8 T cell	0.224032584	2.7887E-07	0.238756899	8.06816E-08
GM2A	Central memory CD8 T cell	-0.016211379	0.713600438	-0.008877311	0.844131316
GUSB	Central memory CD8 T cell	-0.132711272	0.002546687	-0.145226321	0.001222745
IL1RN	Central memory CD8 T cell	0.045207964	0.305852918	0.060401795	0.180585051
NOL11	Central memory CD8 T cell	0.450853872	3.76543E-27	0.443566958	3.51828E-25
NTRK1	Central memory CD8 T cell	-0.204110157	3.0121E-06	-0.215219655	1.41304E-06
RARA	Central memory CD8 T cell	-0.144863755	0.000977582	-0.154086424	0.000596693
RNF128	Central memory CD8 T cell	-0.041278522	0.349849629	-0.047089623	0.296724129
SIGLEC1	Central memory CD8 T cell	-0.137209964	0.0018024	-0.134923498	0.00268282
TNFRSF11A	Central memory CD8 T cell	0.019366873	0.661040206	0.036622795	0.417155484
TOX4	Central memory CD8 T cell	0.259823129	2.16272E-09	0.259796997	4.78129E-09
UBA52	Central memory CD8 T cell	-0.053604912	0.22459259	-0.047186188	0.295733572
ULBP1	Central memory CD8 T cell	-0.023284016	0.45538838	-0.031559248	0.321942881
ACAP1	Effector memeory CD8 T cell	-0.220814758	4.15931E-07	-0.25102603	1.60211E-08
APOL3	Effector memeory CD8 T cell	-0.063890806	0.147653537	-0.043404049	0.336183645
ARHGAP10	Effector memeory CD8 T cell	0.05012941	0.256137784	0.056966837	0.206702841
ATP10D	Effector memeory CD8 T cell	0.021900492	0.619998083	0.041097006	0.362521983
C3AR1	Effector memeory CD8 T cell	-0.023535542	0.594114965	-0.004585353	0.919111135
CCR5	Effector memeory CD8 T cell	-0.042019262	0.34126331	-0.020718103	0.646307371

CD160	Effector memeory CD8 T cell	-0.134322425	0.002252797	-0.129137991	0.004077731
CD55	Effector memeory CD8 T cell	-0.094219653	0.032537355	-0.084515138	0.060774418
CFLAR	Effector memeory CD8 T cell	-4.85832E-05	0.999122447	0.025534876	0.571652633
CMKLR1	Effector memeory CD8 T cell	-0.030241828	0.493479412	-0.014255076	0.752210011
DAPP1	Effector memeory CD8 T cell	-0.048724142	0.26972522	-0.032745756	0.468192762
FCRL6	Effector memeory CD8 T cell	-0.218109599	5.79388E-07	-0.22300804	5.66966E-07
FLT3LG	Effector memeory CD8 T cell	-0.313307374	3.4182E-13	-0.329930377	5.53681E-14
GZMM	Effector memeory CD8 T cell	-0.132212446	0.002644526	-0.14373754	0.001374172
HAPLN3	Effector memeory CD8 T cell	0.119046562	0.006837517	0.156787409	0.000475768
HLA-DMB	Effector memeory CD8 T cell	-0.272775786	3.07419E-10	-0.277879857	3.42206E-10
HLA-DPA1	Effector memeory CD8 T cell	-0.322698168	6.04266E-14	-0.332833127	3.22971E-14
HLA-DPB1	Effector memeory CD8 T cell	-0.406600879	6.34826E-22	-0.42746317	2.55772E-23
IFI16	Effector memeory CD8 T cell	0.035334193	0.423617011	0.060427965	0.180395908
LIME1	Effector memeory CD8 T cell	-0.189950393	1.42639E-05	-0.194487564	1.36786E-05
LTK	Effector memeory CD8 T cell	0.010036301	0.82025873	0.003217133	0.943198149
NFKBIA	Effector memeory CD8 T cell	0.004293202	0.922573948	-0.007213461	0.873069874
SETD7	Effector memeory CD8 T cell	0.241597144	2.82677E-08	0.259995467	4.64981E-09
SIK1	Effector memeory CD8 T cell	0.060313354	0.171736187	0.039570827	0.380634378
TRIB2	Effector memeory CD8 T cell	-0.052916137	0.230613391	-0.037310871	0.408452094
AIM2	Activated CD4 T cell	0.102917895	0.01948552	0.129155261	0.004072736
BIRC3	Activated CD4 T cell	0.045633178	0.301321438	0.066680298	0.139292623
BRIP1	Activated CD4 T cell	0.460278349	2.2902E-28	0.462780774	1.55359E-27
CCL20	Activated CD4 T cell	0.198770888	5.48569E-06	0.202417602	5.89927E-06
CCL4	Activated CD4 T cell	0.118895541	0.006908972	0.143692075	0.001379056
CCL5	Activated CD4 T cell	-0.006329612	0.886057864	0.002025595	0.964217788
CCNB1	Activated CD4 T cell	0.621200617	2.7366E-56	0.622066839	3.8745E-54
CCR7	Activated CD4 T cell	-0.234376194	7.40532E-08	-0.254513392	9.95942E-09
DUSP2	Activated CD4 T cell	-0.109996594	0.012498866	-0.118952547	0.008196567
ESCO2	Activated CD4 T cell	0.558429138	1.46115E-43	0.557986016	1.08773E-41
ETS1	Activated CD4 T cell	0.008235296	0.852101573	0.030931395	0.493212036
EXO1	Activated CD4 T cell	0.592980001	3.2024E-50	0.593980516	2.44704E-48
EXOC6	Activated CD4 T cell	0.285144346	4.32237E-11	0.2939331	2.7866E-11
IARS	Activated CD4 T cell	0.485040166	9.52592E-32	0.500537842	1.27396E-32

ITK	Activated CD4 T cell	-0.11427183	0.009446827	-0.117550163	0.008988934
KIF11	Activated CD4 T cell	0.569204147	1.48621E-45	0.569812156	8.75613E-44
KNTC1	Activated CD4 T cell	0.360134442	3.23758E-17	0.359080283	1.89318E-16
NUF2	Activated CD4 T cell	0.558814265	1.24373E-43	0.554592371	4.18895E-41
PRC1	Activated CD4 T cell	0.562126898	3.08385E-44	0.55662616	1.87058E-41
PSAT1	Activated CD4 T cell	0.432598781	6.6958E-25	0.425003199	4.82535E-23
RGS1	Activated CD4 T cell	-0.112870164	0.010365144	-0.121803353	0.006775194
RTKN2	Activated CD4 T cell	0.091858321	0.037165688	0.084789106	0.059940159
SAMSN1	Activated CD4 T cell	-0.046929023	0.287787879	-0.032771213	0.467846777
SELL	Activated CD4 T cell	-0.135036329	0.002132765	-0.126896273	0.004775231
TRAT1	Activated CD4 T cell	-0.110013562	0.012485205	-0.106070255	0.018482235
ABHD3	Central memory CD4 T cell	0.202437731	3.64057E-06	0.202674082	5.73775E-06
AHNAK	Central memory CD4 T cell	-0.050264529	0.254856762	-0.039505956	0.381416326
ANXA2P2	Central memory CD4 T cell	0.124305715	0.004727243	0.14519131	0.001226123
AQP3	Central memory CD4 T cell	-0.250915537	7.78956E-09	-0.251355733	1.53216E-08
ATHL1	Central memory CD4 T cell	-0.113645343	0.009847936	-0.121309807	0.00700423
BMI1	Central memory CD4 T cell	0.169496109	0.000111029	0.18131552	5.13822E-05
BZW2	Central memory CD4 T cell	0.447444734	1.01486E-26	0.447571152	1.16899E-25
CD63	Central memory CD4 T cell	-0.150980021	0.000586716	-0.149867254	0.000843787
COL4A1	Central memory CD4 T cell	0.196340756	7.16872E-06	0.20210429	6.10247E-06
CYLD	Central memory CD4 T cell	-0.046820569	0.288904617	-0.025960838	0.565252183
ELMO2	Central memory CD4 T cell	0.008554777	0.846433823	0.00690497	0.878455178
FYN	Central memory CD4 T cell	-0.115879647	0.008483131	-0.110124157	0.014429446
GLIPR1	Central memory CD4 T cell	0.006575032	0.881671785	0.028312288	0.530551224
GSS	Central memory CD4 T cell	0.243284022	2.24718E-08	0.234936718	1.31144E-07
IFITM2	Central memory CD4 T cell	-0.113125072	0.010192467	-0.102703475	0.02257022
ITGB1	Central memory CD4 T cell	0.425660122	4.42406E-24	0.431312434	9.37391E-24
ITGB2	Central memory CD4 T cell	-0.179725777	4.09301E-05	-0.174952537	9.4275E-05
KLF5	Central memory CD4 T cell	0.065715578	0.136405699	0.059856239	0.184561484
LSP1	Central memory CD4 T cell	-0.224205349	2.72902E-07	-0.240934634	6.09389E-08
NDUFB9	Central memory CD4 T cell	0.228608149	1.56323E-07	0.22274969	5.84717E-07
PKM2	Central memory CD4 T cell	0.247773573	1.21004E-08	0.248241086	2.33002E-08
SFXN3	Central memory CD4 T cell	-0.077549764	0.078703878	-0.058623376	0.193784818

SIRPG	Central memory CD4 T cell	-0.001232635	0.977737945	0.018815643	0.676860202
SMAD4	Central memory CD4 T cell	0.137040494	0.001826364	0.141467325	0.001638518
STX4	Central memory CD4 T cell	-0.081946678	0.06313017	-0.082344626	0.067728397
TRADD	Central memory CD4 T cell	-0.230466518	1.23143E-07	-0.233630998	1.54538E-07
VIM	Central memory CD4 T cell	-0.146186176	0.000876848	-0.143134763	0.001440244
XRCC6	Central memory CD4 T cell	0.2627806	1.39811E-09	0.266561845	1.82415E-09
ATM	Effector memeory CD4 T cell	-0.018623057	0.673292148	-0.013225941	0.769574961
CASP3	Effector memeory CD4 T cell	0.290347501	1.83876E-11	0.29487158	2.39465E-11
CASQ1	Effector memeory CD4 T cell	-0.322641759	6.107E-14	-0.330795631	4.7178E-14
CD300E	Effector memeory CD4 T cell	0.105207615	0.016923258	0.115905695	0.010004394
DARS	Effector memeory CD4 T cell	0.448763739	6.92446E-27	0.460231897	3.25397E-27
DOCK9	Effector memeory CD4 T cell	-0.110998831	0.011714266	-0.09836667	0.028973353
EXOSC9	Effector memeory CD4 T cell	0.249108639	1.00424E-08	0.251253471	1.55353E-08
EZH2	Effector memeory CD4 T cell	0.42127108	1.42823E-23	0.414807394	6.34229E-22
GDE1	Effector memeory CD4 T cell	-0.13961624	0.001491832	-0.147627427	0.001010558
IL34	Effector memeory CD4 T cell	-0.352515713	1.62945E-16	-0.373356669	9.39938E-18
NCOA4	Effector memeory CD4 T cell	0.122236362	0.005475069	0.133366698	0.003007515
NEFL	Effector memeory CD4 T cell	-0.137128759	0.001813847	-0.128682875	0.00421139
PDGFRL	Effector memeory CD4 T cell	0.094917127	0.031268207	0.09971293	0.026836608
PTGS1	Effector memeory CD4 T cell	-0.097823247	0.026425896	-0.086367055	0.055317503
REPS1	Effector memeory CD4 T cell	0.296085512	7.01888E-12	0.290952312	4.49343E-11
SCG2	Effector memeory CD4 T cell	0.085293386	0.053059309	0.091615427	0.042021548
SDPR	Effector memeory CD4 T cell	-0.341958093	1.42437E-15	-0.335892901	1.81846E-14
SIGLEC14	Effector memeory CD4 T cell	-0.045539875	0.302311932	-0.035481993	0.431823099
SIGLEC6	Effector memeory CD4 T cell	-0.193322739	9.94902E-06	-0.18546341	3.4204E-05
TAL1	Effector memeory CD4 T cell	-0.232224612	9.80786E-08	-0.231550886	2.00326E-07
TFEC	Effector memeory CD4 T cell	-0.07499941	0.089080705	-0.06313352	0.161624211
TIPIN	Effector memeory CD4 T cell	0.403794332	1.28448E-21	0.399141559	2.80624E-20
TPK1	Effector memeory CD4 T cell	-0.198273894	5.79581E-06	-0.190941562	1.97092E-05
UQCRB	Effector memeory CD4 T cell	0.172261846	8.52452E-05	0.174186376	0.00010128
USP9Y	Effector memeory CD4 T cell	0.089892228	0.041434844	0.085306777	0.058389641
WIPF1	Effector memeory CD4 T cell	0.001405399	0.974618703	0.021601826	0.632310977
ZCRB1	Effector memeory CD4 T cell	0.274339291	2.41194E-10	0.271796078	8.4942E-10

B3GAT1	T follicular helper cell	-0.31931883	1.13525E-13	-0.309452885	2.11423E-12
CDK5R1	T follicular helper cell	0.291901857	1.41955E-11	0.29811958	1.41104E-11
PDCD1	T follicular helper cell	0.001973946	0.964356667	0.012209177	0.786845118
BCL6	T follicular helper cell	-0.143155347	0.001123542	-0.141548788	0.001628277
CD200	T follicular helper cell	0.055884194	0.205469353	0.07694824	0.087873669
CD83	T follicular helper cell	-0.295600545	7.62047E-12	-0.302507651	6.83267E-12
CD84	T follicular helper cell	-0.038596034	0.382074246	-0.019300518	0.669020258
FGF2	T follicular helper cell	-0.096426631	0.028665807	-0.088389501	0.049829588
GPR18	T follicular helper cell	-0.124586587	0.004633184	-0.117369887	0.009095589
CEBPA	T follicular helper cell	-0.377753592	6.50717E-19	-0.380292599	2.06894E-18
CECR1	T follicular helper cell	-0.173164801	7.81298E-05	-0.177511568	7.40427E-05
CLEC10A	T follicular helper cell	-0.264222459	1.12803E-09	-0.276982574	3.91863E-10
CLEC4A	T follicular helper cell	-0.153419498	0.00047604	-0.143634496	0.001385265
CSF1R	T follicular helper cell	-0.132456897	0.00259616	-0.122263442	0.006567752
CTSS	T follicular helper cell	-0.084149661	0.056338837	-0.071172408	0.114502581
DPP4	T follicular helper cell	-0.158586847	0.000302613	-0.138602014	0.002038586
LRRC32	T follicular helper cell	-0.00593629	0.893094425	0.003077624	0.945657442
MC5R	T follicular helper cell	-0.249645603	9.3141E-09	-0.243279166	4.49123E-08
MICA	T follicular helper cell	-0.075912522	0.08524588	-0.077237195	0.086684836
NCAM1	T follicular helper cell	-0.155904977	0.000383497	-0.162570411	0.00028938
NCR2	T follicular helper cell	0.058347427	0.186160541	0.055747779	0.216599655
NRP1	T follicular helper cell	0.071022788	0.107423922	0.084770265	0.059997227
PDCD1LG2	T follicular helper cell	0.067111092	0.128257962	0.098415218	0.028893834
PDCD6	T follicular helper cell	0.159864199	0.000269968	0.149950664	0.000838098
PRDX1	T follicular helper cell	0.21188568	1.22244E-06	0.203192835	5.42387E-06
RAE1	T follicular helper cell	0.389248049	4.45859E-20	0.379016136	2.74101E-18
RAET1E	T follicular helper cell	0.197819552	6.0939E-06	0.206883316	3.61974E-06
SIGLEC7	T follicular helper cell	-0.018550797	0.67448708	-0.002184029	0.961421159
SIGLEC9	T follicular helper cell	-0.067877048	0.123949122	-0.056980314	0.206595284
TYRO3	T follicular helper cell	0.163288485	0.000197958	0.183489463	4.15594E-05
CHST12	T follicular helper cell	-0.070440405	0.110344497	-0.089289071	0.047539068
CLIC3	T follicular helper cell	-0.318557437	1.30714E-13	-0.333486958	2.85819E-14
IVNS1ABP	T follicular helper cell	0.131051272	0.002885682	0.137634751	0.002192631

LGMN	T follicular helper cell	-0.016305207	0.712016324	-0.008339228	0.853468978
ACP5	Gamma delta T cell	-0.220518866	4.31376E-07	-0.234189124	1.44083E-07
AQP9	Gamma delta T cell	0.155373194	0.00040176	0.171695219	0.000127592
BTN3A2	Gamma delta T cell	-0.077441309	0.079124257	-0.08087167	0.072809378
C1orf54	Gamma delta T cell	-0.143843024	0.001062529	-0.152111496	0.000702534
CARD8	Gamma delta T cell	-0.186658291	2.01536E-05	-0.195663679	1.21001E-05
CCL18	Gamma delta T cell	0.001151238	0.979207669	0.013760089	0.760547108
CD209	Gamma delta T cell	0.118091279	0.00730089	0.141054555	0.001691322
CD33	Gamma delta T cell	-0.22657762	2.02411E-07	-0.223426426	5.39312E-07
CD36	Gamma delta T cell	-0.15182574	0.000545893	-0.148676077	0.000929016
CDK5	Gamma delta T cell	0.127382887	0.003784856	0.119792993	0.007752139
IL10RB	Gamma delta T cell	-0.016138636	0.714829421	-0.010172175	0.821752567
KLRF1	Gamma delta T cell	-0.07416063	0.092724322	-0.083924687	0.062605037
LGALS1	Gamma delta T cell	0.075819437	0.085630614	0.086289963	0.055536255
MAPK7	Gamma delta T cell	-0.014955946	0.734912097	-0.02758725	0.541134851
KLHL7	Gamma delta T cell	0.286120667	3.68682E-11	0.290264967	5.01284E-11
KRT80	Gamma delta T cell	0.31172877	4.54696E-13	0.311414268	1.50949E-12
LAMC1	Gamma delta T cell	0.330740405	1.30475E-14	0.336572157	1.59936E-14
LCORL	Gamma delta T cell	0.142507164	0.001183992	0.152628759	0.000673247
LMNB1	Gamma delta T cell	0.498912291	9.13302E-34	0.495766432	6.07285E-32
MEIS3P1	Gamma delta T cell	-0.065244547	0.139243941	-0.07596971	0.091997236
MPL	Gamma delta T cell	-0.166118919	0.000152464	-0.164691692	0.00024012
FABP1	Gamma delta T cell	-0.033936407	0.442196345	-0.022466984	0.618734632
FABP5	Gamma delta T cell	0.010234105	0.816778395	0.013601752	0.763219891
FADD	Gamma delta T cell	0.381637667	2.66161E-19	0.391663727	1.59766E-19
MFAP3L	Gamma delta T cell	0.064109827	0.146267309	0.06638294	0.141068747
MINPP1	Gamma delta T cell	0.34564891	6.73755E-16	0.359245387	1.83013E-16
RPS24	Gamma delta T cell	0.058295654	0.186552121	0.058288805	0.19634506
RPS7	Gamma delta T cell	0.168759194	0.000119046	0.167226406	0.000191556
RPS9	Gamma delta T cell	-0.205474978	2.57751E-06	-0.203317731	5.35078E-06
DBNL	Gamma delta T cell	-0.036546776	0.407874663	-0.029592276	0.512126055
CCL13	Gamma delta T cell	-0.118175487	0.007258943	-0.098556383	0.028663677
CD70	Type 1 T helper cell	0.00271091	0.951063775	0.020035481	0.657205151

TBX21	Type 1 T helper cell	-0.076596593	0.082461072	-0.073392801	0.103600467
ADAM8	Type 1 T helper cell	-0.088332912	0.045108654	-0.087352175	0.052584678
AHCYL2	Type 1 T helper cell	-0.09007387	0.041023797	-0.091372147	0.042572025
ALCAM	Type 1 T helper cell	-0.060399842	0.171121322	-0.078201387	0.082811309
B3GALNT1	Type 1 T helper cell	0.190122455	1.40063E-05	0.199228595	8.30672E-06
BBS12	Type 1 T helper cell	0.037696894	0.393269347	0.049906005	0.268739702
BST1	Type 1 T helper cell	-0.032561437	0.460917688	-0.0103281	0.8190671
CD151	Type 1 T helper cell	-0.120287281	0.006275088	-0.12615066	0.005030047
CD47	Type 1 T helper cell	-0.156294167	0.000370625	-0.160713077	0.000340106
CD48	Type 1 T helper cell	-0.203917499	3.07882E-06	-0.214222934	1.5844E-06
CD52	Type 1 T helper cell	-0.316601281	1.87428E-13	-0.346119085	2.54461E-15
CD53	Type 1 T helper cell	-0.10160092	0.021107313	-0.092904717	0.039203487
CD59	Type 1 T helper cell	-0.2287557	1.53401E-07	-0.208250014	3.11047E-06
CD6	Type 1 T helper cell	-0.136903793	0.001845907	-0.140814644	0.001722726
CD68	Type 1 T helper cell	-0.011963597	0.786508551	0.000563654	0.990039956
CD7	Type 1 T helper cell	0.034204611	0.438595764	0.046876495	0.298918183
CD96	Type 1 T helper cell	-0.104191955	0.018020976	-0.099478154	0.027199168
CFHR3	Type 1 T helper cell	0.111489107	0.011346569	0.102085262	0.023400504
CHRM3	Type 1 T helper cell	0.016248576	0.712972287	0.016013538	0.72283052
CLEC7A	Type 1 T helper cell	-0.107087442	0.015045142	-0.101602153	0.024067611
COL23A1	Type 1 T helper cell	-0.15752175	0.000332614	-0.155726056	0.000520275
COL4A4	Type 1 T helper cell	-0.272957254	2.98907E-10	-0.27445112	5.72809E-10
COL5A3	Type 1 T helper cell	0.167551418	0.000133373	0.175881667	8.63921E-05
DAB1	Type 1 T helper cell	-0.007684187	0.861896277	-0.000437375	0.992271298
DLEU7	Type 1 T helper cell	0.120602817	0.006138843	0.118378968	0.00851279
DOC2B	Type 1 T helper cell	0.102641848	0.019816168	0.108125305	0.016318101
EMP1	Type 1 T helper cell	-0.021147628	0.632078467	0.001906275	0.966324294
F12	Type 1 T helper cell	0.321081568	8.1784E-14	0.327150116	9.22897E-14
FURIN	Type 1 T helper cell	0.202425476	3.64561E-06	0.198087144	9.37682E-06
GAB3	Type 1 T helper cell	-0.298129684	4.95436E-12	-0.3224831	2.15058E-13
GATM	Type 1 T helper cell	-0.076378717	0.083339936	-0.067579559	0.134025688
GFPT2	Type 1 T helper cell	0.233728896	8.06085E-08	0.274003873	6.12299E-10
GPR25	Type 1 T helper cell	-0.125868796	0.004224902	-0.124570036	0.005611171

GREM2	Type 1 T helper cell	-0.150287961	0.000622212	-0.138031406	0.002128218
HAVCR1	Type 1 T helper cell	0.165547174	0.000160777	0.172152982	0.00012232
HSD11B1	Type 1 T helper cell	0.050670239	0.25103709	0.07547957	0.094120205
HUNK	Type 1 T helper cell	-0.085822179	0.051597691	-0.089216364	0.047720859
IGF2	Type 1 T helper cell	-0.074864377	0.08965939	-0.074818673	0.097044576
RCSD1	Type 1 T helper cell	-0.180430585	3.81311E-05	-0.190035893	2.16133E-05
RYR1	Type 1 T helper cell	-0.184253289	2.58455E-05	-0.182900536	4.40288E-05
SAV1	Type 1 T helper cell	0.342096544	1.38518E-15	0.348746847	1.51716E-15
SELE	Type 1 T helper cell	-0.047104469	0.285987476	-0.033797558	0.454016005
SELP	Type 1 T helper cell	-0.233008835	8.85591E-08	-0.224345364	4.83052E-07
SH3KBP1	Type 1 T helper cell	0.104321889	0.017877162	0.123876028	0.005884886
SIT1	Type 1 T helper cell	-0.128873768	0.003392559	-0.138079277	0.002120562
SLC35B3	Type 1 T helper cell	0.171233157	9.40943E-05	0.175779356	8.72287E-05
SIGLEC10	Type 1 T helper cell	0.038241455	0.386465605	0.050424823	0.263788432
SKAP1	Type 1 T helper cell	-0.053127074	0.228757492	-0.049049939	0.277047792
THUMPD2	Type 1 T helper cell	0.181846043	3.3048E-05	0.173966801	0.000103376
TIGIT	Type 1 T helper cell	-0.00085192	0.984612826	0.021730387	0.630285567
ZEB2	Type 1 T helper cell	-0.086465621	0.049864639	-0.076022951	0.09176896
ENC1	Type 1 T helper cell	-0.07618557	0.08412536	-0.067191224	0.136280996
FAM134B	Type 1 T helper cell	-0.144353411	0.001019237	-0.14761443	0.001011609
FBXO30	Type 1 T helper cell	0.377379359	7.08803E-19	0.384178344	8.72044E-19
FCGR2C	Type 1 T helper cell	-0.039345033	0.372898946	-0.026560457	0.556301
STAC	Type 1 T helper cell	-0.141385442	0.001295731	-0.132706421	0.003155714
LTC4S	Type 1 T helper cell	-0.466885019	3.05236E-29	-0.477501592	1.91975E-29
MAN1B1	Type 1 T helper cell	0.040032534	0.364597876	0.041386788	0.359144699
MDH1	Type 1 T helper cell	0.299698769	3.78487E-12	0.309323351	2.16161E-12
MMD	Type 1 T helper cell	0.337370522	3.56245E-15	0.349934396	1.19906E-15
RGS16	Type 1 T helper cell	-0.08552756	0.052407834	-0.093041833	0.038913424
IL12A	Type 1 T helper cell	-0.018406146	0.676881573	-0.030401309	0.500654198
P2RX5	Type 1 T helper cell	0.081366754	0.065026054	0.098117773	0.029383987
CD97	Type 1 T helper cell	-0.225527282	2.31135E-07	-0.217191724	1.12488E-06
ITGB4	Type 1 T helper cell	0.029711409	0.501094051	0.039845077	0.377339419
ICAM3	Type 1 T helper cell	-0.191757597	1.17688E-05	-0.216801238	1.17704E-06

METRNL	Type 1 T helper cell	-0.075088847	0.088699076	-0.080497732	0.074147621
TNFRSF1A	Type 1 T helper cell	0.211424047	1.29091E-06	0.211666973	2.11964E-06
IRF1	Type 1 T helper cell	0.101576673	0.02113824	0.129286076	0.004035077
HTR2B	Type 1 T helper cell	-0.078246182	0.076047523	-0.071420416	0.113241937
CALD1	Type 1 T helper cell	0.126827565	0.003941184	0.151902461	0.0007147
MOCOS	Type 1 T helper cell	0.355772115	8.2108E-17	0.380826151	1.83876E-18
TRAF3IP2	Type 1 T helper cell	-0.053710995	0.223675325	-0.053688448	0.23408205
TLR8	Type 1 T helper cell	-0.044699993	0.311325098	-0.024908475	0.58112678
TRAF1	Type 1 T helper cell	-0.139309278	0.001528511	-0.135270258	0.002614995
DUSP14	Type 1 T helper cell	0.329461252	1.67006E-14	0.327669917	8.39151E-14
IL17A	Type 17 T helper cell	0.017946952	0.684504377	0.022848764	0.612784245
IL17RA	Type 17 T helper cell	0.004605303	0.916964955	0.016198543	0.719762247
C2CD4A	Type 17 T helper cell	0.164037964	0.000184809	0.171728298	0.000127204
C2CD4B	Type 17 T helper cell	-0.10002772	0.023197239	-0.100892384	0.025077501
CA2	Type 17 T helper cell	-0.110600062	0.012021066	-0.114491532	0.01095779
CCDC65	Type 17 T helper cell	-0.166453511	0.000147788	-0.162858181	0.000282184
CEACAM3	Type 17 T helper cell	-0.046630972	0.290863874	-0.035397644	0.4329193
IL17C	Type 17 T helper cell	0.214947456	8.49018E-07	0.216582663	1.20724E-06
IL17F	Type 17 T helper cell	-0.123008283	0.005184484	-0.107515199	0.016936155
IL17RC	Type 17 T helper cell	-0.165818675	0.000156778	-0.188031084	2.64676E-05
IL17RE	Type 17 T helper cell	-0.140849489	0.001352475	-0.167272658	0.000190762
IL23A	Type 17 T helper cell	0.080320194	0.068565886	0.083789629	0.063030095
ILDR1	Type 17 T helper cell	-0.139933524	0.001454771	-0.149796178	0.000848662
LONRF3	Type 17 T helper cell	0.095205945	0.0307552	0.085161304	0.058821965
SH2D6	Type 17 T helper cell	-0.076676667	0.082139961	-0.091716718	0.041794131
TNIP2	Type 17 T helper cell	0.147770274	0.000768837	0.146175186	0.001134417
ABCA1	Type 17 T helper cell	0.086525713	0.049705301	0.102712032	0.022558909
ABCB1	Type 17 T helper cell	-0.112213283	0.01082214	-0.09665171	0.031905383
ADAMTS12	Type 17 T helper cell	0.304510911	1.64052E-12	0.334735253	2.26158E-14
ANK1	Type 17 T helper cell	-0.041607976	0.346014008	-0.030837368	0.494527809
ANKRD22	Type 17 T helper cell	-0.057382367	0.193559729	-0.036892198	0.413734935
B3GALT2	Type 17 T helper cell	-0.270924923	4.08878E-10	-0.274544142	5.64912E-10
CAMTA1	Type 17 T helper cell	-0.085759892	0.051768091	-0.102942523	0.022256081

CCR9	Type 17 T helper cell	-0.143919863	0.001055904	-0.136089473	0.002460921
CD40	Type 17 T helper cell	-0.15948441	0.000279313	-0.156506711	0.000487181
GPR44	Type 17 T helper cell	-0.325956405	3.26516E-14	-0.32121344	2.70031E-13
IFT80	Type 17 T helper cell	-0.174369326	6.95064E-05	-0.183591813	4.11439E-05
ASB2	Type 2 T helper cell	-0.164506005	0.000177018	-0.176829464	7.89933E-05
CSRP2	Type 2 T helper cell	0.210394398	1.45712E-06	0.207963421	3.21123E-06
DAPK1	Type 2 T helper cell	-0.214959904	8.47752E-07	-0.219694685	8.39563E-07
DLC1	Type 2 T helper cell	-0.3475889	4.52765E-16	-0.348793553	1.50321E-15
DNAJC12	Type 2 T helper cell	0.168577288	0.000121107	0.172689433	0.000116402
DUSP6	Type 2 T helper cell	-0.05497227	0.212973775	-0.045176445	0.316805184
GNAI1	Type 2 T helper cell	0.047140928	0.2856143	0.047629523	0.29121427
LAMP3	Type 2 T helper cell	-0.2789941	1.16061E-10	-0.292349309	3.59443E-11
NRP2	Type 2 T helper cell	0.137059734	0.001823629	0.167362564	0.000189227
OSBPL1A	Type 2 T helper cell	-0.01260906	0.775290858	-0.008279103	0.85451366
PDE4B	Type 2 T helper cell	-0.034698394	0.432010967	-0.019017451	0.673592864
PHLDA1	Type 2 T helper cell	0.088758916	0.044078764	0.105449705	0.019183145
PLA2G4A	Type 2 T helper cell	0.19438623	8.86897E-06	0.190428148	2.07681E-05
RAB27B	Type 2 T helper cell	0.11541468	0.008752389	0.130066767	0.003816808
RBMS3	Type 2 T helper cell	-0.19867131	5.54653E-06	-0.183364046	4.20741E-05
RNF125	Type 2 T helper cell	-0.066676039	0.130756517	-0.058273747	0.196460867
TMPRSS3	Type 2 T helper cell	-0.139153469	0.001547443	-0.141655425	0.00161496
GATA3	Type 2 T helper cell	-0.040137607	0.363339413	-0.029517743	0.513189736
BIRC5	Type 2 T helper cell	0.522804476	1.81952E-37	0.516478039	5.75684E-35
CDC25C	Type 2 T helper cell	0.530469535	1.02135E-38	0.530477655	3.94394E-37
CDC7	Type 2 T helper cell	0.380314227	3.61426E-19	0.376319965	4.94519E-18
CENPF	Type 2 T helper cell	0.542539588	9.40876E-41	0.540255398	1.05516E-38
CXCR6	Type 2 T helper cell	-0.012282814	0.780955099	0.000276221	0.995118937
DHFR	Type 2 T helper cell	0.320726608	8.73823E-14	0.326781502	9.87195E-14
EVI5	Type 2 T helper cell	0.057244306	0.194635619	0.05969889	0.185720284
GSTA4	Type 2 T helper cell	-0.125966374	0.004195205	-0.150849685	0.000778997
HELLS	Type 2 T helper cell	0.468757967	1.71009E-29	0.474365404	4.98368E-29
IL26	Type 2 T helper cell	-0.03735097	0.397628662	-0.023549606	0.601927183
LAIR2	Type 2 T helper cell	-0.015196305	0.730815506	-0.006521142	0.885163421

CCL3L1	Regulatory T cell	0.042277531	0.338301475	0.057790794	0.200201612
CD72	Regulatory T cell	-0.055516218	0.208474119	-0.050542474	0.262674429
CLEC5A	Regulatory T cell	-0.073311779	0.096532172	-0.067856194	0.132436724
FOXP3	Regulatory T cell	-0.044059801	0.31831278	-0.038125991	0.398282482
ITGA4	Regulatory T cell	-0.083208352	0.059162615	-0.072105627	0.109815582
L1CAM	Regulatory T cell	0.036864305	0.403810514	0.048547264	0.28200671
LIPA	Regulatory T cell	-0.101559939	0.021159607	-0.090004482	0.045781059
LRP1	Regulatory T cell	-0.023345997	0.597090142	-0.015226372	0.735934733
LRRC42	Regulatory T cell	0.426662501	3.37698E-24	0.426744115	3.08083E-23
MARCO	Regulatory T cell	-0.171257053	9.38793E-05	-0.172978354	0.000113327
MMP12	Regulatory T cell	0.31093233	5.24763E-13	0.320071005	3.31107E-13
MNDA	Regulatory T cell	-0.159803228	0.000271449	-0.150857995	0.00077847
MRC1	Regulatory T cell	-0.155610487	0.000393513	-0.14428273	0.001316824
MS4A6A	Regulatory T cell	-0.128231995	0.003556667	-0.11864245	0.008366202
PELO	Regulatory T cell	0.197300385	6.45242E-06	0.221409651	6.85701E-07
PLEK	Regulatory T cell	-0.078875392	0.073710667	-0.061567484	0.172301805
PRSS23	Regulatory T cell	0.241829765	2.739E-08	0.271498548	8.87528E-10
PTGIR	Regulatory T cell	-0.223126893	3.12272E-07	-0.24408904	4.03894E-08
ST8SIA4	Regulatory T cell	-0.056881116	0.197486779	-0.04075886	0.366487912
STAB1	Regulatory T cell	-0.078016313	0.076916136	-0.081921193	0.069158333
ADAM28	Activated B cell	-0.059105139	0.180498802	-0.052491656	0.244688551
CD180	Activated B cell	-0.077198041	0.080073795	-0.061202965	0.174861006
CD79B	Activated B cell	-0.164578046	0.000175846	-0.178609749	6.66821E-05
BLK	Activated B cell	-0.248750085	1.05591E-08	-0.273769446	6.34043E-10
CD19	Activated B cell	-0.145187265	0.000951997	-0.163293283	0.000271621
MS4A1	Activated B cell	-0.200370968	4.59128E-06	-0.216517331	1.21641E-06
TNFRSF17	Activated B cell	-0.084548569	0.055176396	-0.079770657	0.076807027
GNG7	Activated B cell	-0.444367255	2.46013E-26	-0.448261032	9.6544E-26
MICAL3	Activated B cell	0.169056008	0.000115753	0.180801777	5.40048E-05
SPIB	Activated B cell	-0.217740442	6.05998E-07	-0.235532299	1.21645E-07
HLA-DOB	Activated B cell	-0.305610234	1.35236E-12	-0.337938396	1.23419E-14
PNOC	Activated B cell	-0.053871553	0.222292128	-0.052577291	0.243918631
FCRL2	Activated B cell	-0.142562468	0.00117872	-0.147652194	0.001008559

BACH2	Activated B cell	0.039022215	0.376836692	0.059541611	0.186883914
CR2	Activated B cell	-0.145503291	0.000927602	-0.145566621	0.001190364
TCL1A	Activated B cell	-0.08782015	0.046375043	-0.076275514	0.090692241
AKNA	Activated B cell	-0.209966418	1.53208E-06	-0.210391747	2.44763E-06
ARHGAP25	Activated B cell	-0.184905337	2.41674E-05	-0.198829012	8.66735E-06
CCL21	Activated B cell	0.034893517	0.429424738	0.060456904	0.180186929
CD27	Activated B cell	-0.149323133	0.00067503	-0.158832393	0.000399828
CD38	Activated B cell	0.02732701	0.536076322	0.038610517	0.392310153
CLEC17A	Activated B cell	-0.160977918	0.000244222	-0.169615147	0.000154353
CLEC9A	Activated B cell	-0.314705545	2.65107E-13	-0.319536245	3.64158E-13
CLECL1	Activated B cell	-0.240805442	3.14634E-08	-0.253567012	1.13388E-08
CD22	Immature B cell	-0.310895212	5.28274E-13	-0.338122362	1.19174E-14
CYBB	Immature B cell	-0.06094032	0.167316125	-0.038808142	0.389889827
FAM129C	Immature B cell	-0.242348956	2.55252E-08	-0.259526846	4.96607E-09
FCRL1	Immature B cell	-0.253629164	5.29917E-09	-0.27284271	7.27595E-10
FCRL3	Immature B cell	-0.1743678	6.95167E-05	-0.178684354	6.6208E-05
FCRL5	Immature B cell	0.01421156	0.747646498	0.02045217	0.65054406
FCRLA	Immature B cell	-0.117263088	0.00772514	-0.131374698	0.003474988
HDAC9	Immature B cell	-0.062139877	0.159097701	-0.051718633	0.251715637
HLA-DQA1	Immature B cell	-0.255072648	4.3094E-09	-0.269006083	1.27921E-09
HVCN1	Immature B cell	-0.189227535	1.53965E-05	-0.193674705	1.48819E-05
KIAA0226	Immature B cell	0.094023036	0.032902979	0.101475158	0.024245679
NCF1	Immature B cell	-0.126326227	0.004087313	-0.131243697	0.003507927
NCF1B	Immature B cell	-0.126386008	0.004069634	-0.142701686	0.001489503
P2RY10	Immature B cell	-0.042233275	0.33880783	-0.028254603	0.531389417
SP100	Immature B cell	-0.00871678	0.843562818	0.012315618	0.785032103
TXNIP	Immature B cell	-0.297475304	5.54046E-12	-0.287639344	7.59233E-11
STAP1	Immature B cell	-0.234002244	7.77747E-08	-0.254316383	1.02324E-08
TAGAP	Immature B cell	-0.165757924	0.000157664	-0.17032314	0.000144703
ZCCHC2	Immature B cell	0.101756424	0.020909903	0.100587408	0.025522544
AICDA	Memory B cell	-0.027342948	0.535838478	-0.015063864	0.738649763
CCNA2	Memory B cell	0.614489762	8.63061E-55	0.6159704	7.89084E-53
CDKN3	Memory B cell	0.626487491	1.70037E-57	0.626434205	4.29175E-55

CLCN5	Memory B cell	-0.029403042	0.505549307	-0.031873917	0.480126748
ENPP1	Memory B cell	0.282606202	6.51676E-11	0.288888857	6.2346E-11
FCER1A	Memory B cell	-0.422285394	1.09103E-23	-0.418385691	2.59399E-22
FCRL4	Memory B cell	-0.121032846	0.005957438	-0.132304455	0.003249155
MYC	Memory B cell	0.28653582	3.44509E-11	0.291232246	4.29728E-11
RUNX2	Memory B cell	0.13790151	0.00170757	0.147268332	0.001039966
SORL1	Memory B cell	-0.033100199	0.453529867	-0.034311846	0.447172972
SOX5	Memory B cell	-0.061245179	0.165197935	-0.070984663	0.115464205
STAT5A	Memory B cell	-0.152015112	0.000537121	-0.141050787	0.001691811
STAT5B	Memory B cell	-0.040946215	0.353745601	-0.032921121	0.465812246
TLR9	Memory B cell	-0.00906758	0.837353097	-0.005749705	0.898670389
AKT3	Natural killer cell	0.109731236	0.012714219	0.131009225	0.003567591
AXL	Natural killer cell	-0.059021238	0.181119367	-0.045552429	0.312790215
BST2	Natural killer cell	-0.03027508	0.493004115	-0.020127924	0.65572501
CDH2	Natural killer cell	0.193565407	9.69206E-06	0.206689128	3.69827E-06
CRTAM	Natural killer cell	-0.112042014	0.01094419	-0.102473762	0.022875705
CSF2RA	Natural killer cell	-0.132769255	0.002535531	-0.124793033	0.005525684
CTSZ	Natural killer cell	-0.11538081	0.008772298	-0.103566811	0.021453503
CXCL1	Natural killer cell	0.054878662	0.213755123	0.058421457	0.195327028
CYTH1	Natural killer cell	-0.151204572	0.000575609	-0.154937023	0.000555837
DAXX	Natural killer cell	0.185089874	2.37116E-05	0.167196047	0.000192079
DGKH	Natural killer cell	0.143814164	0.001065026	0.165471602	0.00022407
DLL4	Natural killer cell	0.02283495	0.60514519	0.019197869	0.670677032
DPYD	Natural killer cell	-0.101193805	0.021631825	-0.081725659	0.069826948
ERBB3	Natural killer cell	0.012777608	0.7723692	0.002403333	0.957550831
F11R	Natural killer cell	0.029207699	0.508382326	0.021506153	0.633820031
FAM27A	Natural killer cell	-0.121268915	0.005859916	-0.110225621	0.014338924
FAM49A	Natural killer cell	-0.162553891	0.000211692	-0.161709385	0.000311947
FASLG	Natural killer cell	0.064262078	0.14530955	0.088203094	0.050315589
FCGR1A	Natural killer cell	-0.008899032	0.840335425	0.005550397	0.902164971
FN1	Natural killer cell	0.187791514	1.79049E-05	0.209698092	2.6459E-06
FSTL1	Natural killer cell	0.111077944	0.011654229	0.140452534	0.001771138
FUCA1	Natural killer cell	-0.337875901	3.22265E-15	-0.333993736	2.59937E-14

GBP3	Natural killer cell	0.035330327	0.423667756	0.062778073	0.164002926
GLS2	Natural killer cell	-0.274097724	2.50431E-10	-0.289035919	6.0913E-11
GRB2	Natural killer cell	0.135540388	0.002051568	0.143379612	0.001413061
LST1	Natural killer cell	-0.316208003	2.01447E-13	-0.327676244	8.38179E-14
BCL2	Natural killer cell	-0.141910197	0.001242302	-0.137340305	0.002241594
CDC5L	Natural killer cell	0.382630117	2.11399E-19	0.376040226	5.25576E-18
FGF18	Natural killer cell	-0.225215546	2.40391E-07	-0.224134285	4.95453E-07
FUT5	Natural killer cell	0.093100877	0.034664948	0.092039663	0.041075983
FZR1	Natural killer cell	0.066104374	0.134096683	0.061932054	0.169770287
IGFBP5	Natural killer cell	0.148289315	0.000736217	0.159028692	0.000393166
KANK2	Natural killer cell	-0.198812615	5.46039E-06	-0.201142337	6.76891E-06
LDB3	Natural killer cell	-0.260034951	2.09656E-09	-0.260016819	4.63588E-09
ABAT	CD56bright natural killer cell	-0.200204133	4.67759E-06	-0.200370561	7.35321E-06
C11orf75	CD56bright natural killer cell	-0.071394894	0.105590056	-0.059868861	0.184468758
C5orf15	CD56bright natural killer cell	0.223202628	3.09337E-07	0.235223115	1.2649E-07
CDHR1	CD56bright natural killer cell	-0.214793471	8.64838E-07	-0.226397559	3.77056E-07
DCAF12	CD56bright natural killer cell	0.174059773	7.16325E-05	0.185428019	3.43242E-05
DYNLL1	CD56bright natural killer cell	0.467684182	2.38487E-29	0.467764799	3.59464E-28
GPR137B	CD56bright natural killer cell	0.073162383	0.097215074	0.086442486	0.055104161
HCP5	CD56bright natural killer cell	-0.024241623	0.583092	-0.017843817	0.692678491
HDGFRP2	CD56bright natural killer cell	-0.00629245	0.886722324	-0.02163604	0.631771682
KRT86	CD56bright natural killer cell	0.09334784	0.034185371	0.077796538	0.084420364
MLST8	CD56bright natural killer cell	0.052167361	0.237287465	0.045818066	0.309973817
ELMOD3	CD56bright natural killer cell	-0.22946841	1.40013E-07	-0.231678217	1.97183E-07
ENTPD5	CD56bright natural killer cell	0.306550798	1.14562E-12	0.312603118	1.22917E-12
FAM119A	CD56bright natural killer cell	0.225222917	2.40168E-07	0.219013082	9.09494E-07
FAM179A	CD56bright natural killer cell	-0.283286022	5.84049E-11	-0.29578415	2.06536E-11
CLIC2	CD56bright natural killer cell	-0.148548747	0.000720397	-0.14464535	0.001279909
COX7A2L	CD56bright natural killer cell	0.30498814	1.50871E-12	0.307555725	2.92181E-12
CREB3L4	CD56bright natural killer cell	0.070726548	0.108901806	0.06529056	0.147742777
CSF1	CD56bright natural killer cell	-0.099928445	0.023334931	-0.093665922	0.037616051
CSNK2A2	CD56bright natural killer cell	0.186195911	2.11461E-05	0.195948489	1.17448E-05
CSTA	CD56bright natural killer cell	-0.119586685	0.006587347	-0.117471412	0.009035387

CSTB	CD56bright natural killer cell	0.024948408	0.572154246	0.009141336	0.839557448
CTPS	CD56bright natural killer cell	0.417179283	4.19387E-23	0.41035877	1.89889E-21
CTSD	CD56bright natural killer cell	-0.298753228	4.45249E-12	-0.307560696	2.91934E-12
FST	CD56bright natural killer cell	0.210941093	1.36647E-06	0.216107129	1.27554E-06
GATA2	CD56bright natural killer cell	-0.068400877	0.121067799	-0.068246963	0.130217021
GMPR	CD56bright natural killer cell	-0.35769963	5.4522E-17	-0.353555309	5.81527E-16
HDC	CD56bright natural killer cell	-0.288890092	2.34028E-11	-0.295241939	2.25526E-11
HEY1	CD56bright natural killer cell	0.007227875	0.870022307	0.014260021	0.752126856
HOXA1	CD56bright natural killer cell	0.204380191	2.9209E-06	0.21451523	1.53218E-06
HS2ST1	CD56bright natural killer cell	0.290432933	1.81287E-11	0.297377613	1.59319E-11
HS3ST1	CD56bright natural killer cell	-0.001773288	0.967977885	0.01158679	0.797469012
BCL11B	CD56bright natural killer cell	-0.093009599	0.034843645	-0.080341557	0.07471244
CDH3	CD56bright natural killer cell	0.162098324	0.000220652	0.171294737	0.000132379
MYL6B	CD56bright natural killer cell	0.347112476	4.9932E-16	0.33671257	1.55741E-14
NAA16	CD56bright natural killer cell	-0.056323332	0.201924731	-0.056398791	0.211272976
CYP27B1	CD56bright natural killer cell	0.08091927	0.066520764	0.086223969	0.055724086
EIF3M	CD56bright natural killer cell	0.336917686	3.89663E-15	0.346023859	2.59251E-15
CYP27A1	CD56dim natural killer cell	-0.354072784	1.17529E-16	-0.361142647	1.23826E-16
DDX55	CD56dim natural killer cell	0.320689432	8.79899E-14	0.31016715	1.87065E-12
DYRK2	CD56dim natural killer cell	0.250254573	8.55006E-09	0.260081316	4.59403E-09
RPL37A	CD56dim natural killer cell	-0.023203191	0.599336127	-0.027730633	0.539033537
NOTCH3	CD56dim natural killer cell	0.238805482	4.11705E-08	0.234070426	1.46248E-07
AKR7A3	CD56dim natural killer cell	0.178022161	4.85197E-05	0.162870024	0.000281891
GPRC5C	CD56dim natural killer cell	-0.273971572	2.55392E-10	-0.287679027	7.54508E-11
GRIN1	CD56dim natural killer cell	0.088986079	0.04353772	0.103309292	0.02178147
HLA-E	CD56dim natural killer cell	-0.194520555	8.74078E-06	-0.197130285	1.03738E-05
PORCN	CD56dim natural killer cell	0.086433071	0.049951125	0.079245012	0.078777538
PSMC4	CD56dim natural killer cell	0.365818987	9.422E-18	0.371781789	1.31877E-17
UPP1	CD56dim natural killer cell	0.095765355	0.02978203	0.108816045	0.015642289
IL21R	CD56dim natural killer cell	-0.022542616	0.609774644	0.000436085	0.992294093
CCR2	Myeloid derived suppressor cell	-0.126005908	0.004183227	-0.10866265	0.01579021
CD14	Myeloid derived suppressor cell	-0.026168348	0.55350726	-0.009628322	0.831135319
CD2	Myeloid derived suppressor cell	-0.107850239	0.01433687	-0.106346476	0.0181775

CD86	Myeloid derived suppressor cell	-0.04899258	0.267092336	-0.03779769	0.402360015
CXCR4	Myeloid derived suppressor cell	-0.116377952	0.008202789	-0.126882409	0.004779861
FCGR2A	Myeloid derived suppressor cell	-0.023157682	0.600052672	-0.005030384	0.911291358
FCGR2B	Myeloid derived suppressor cell	-0.035664655	0.41929202	-0.024005937	0.594904776
FCGR3A	Myeloid derived suppressor cell	0.13324885	0.002444952	0.154307022	0.000585837
FERMT3	Myeloid derived suppressor cell	-0.159723153	0.000273404	-0.169277435	0.000159166
GPSM3	Myeloid derived suppressor cell	-0.224258759	2.71082E-07	-0.22923698	2.66603E-07
IL18BP	Myeloid derived suppressor cell	-0.019217784	0.663488841	-0.005371054	0.905311108
IL4R	Myeloid derived suppressor cell	0.009972168	0.821387911	0.018153193	0.687627902
ITGAL	Myeloid derived suppressor cell	-0.193864637	9.38392E-06	-0.206867732	3.62598E-06
ITGAM	Myeloid derived suppressor cell	-0.146296872	0.000868867	-0.133815697	0.002910383
PARVG	Myeloid derived suppressor cell	-0.280986796	8.45001E-11	-0.312980793	1.15129E-12
PSAP	Myeloid derived suppressor cell	-0.031924187	0.46974196	-0.019968948	0.658271271
PTGER2	Myeloid derived suppressor cell	-0.037277654	0.398556301	-0.019850992	0.660163087
PTGES2	Myeloid derived suppressor cell	0.25723632	3.15348E-09	0.250126732	1.80898E-08
S100A8	Myeloid derived suppressor cell	0.068947766	0.118115662	0.084388759	0.061162476
S100A9	Myeloid derived suppressor cell	0.07815868	0.076377225	0.08521516	0.058661607
BTN2A2	Natural killer T cell	-0.230938865	1.1586E-07	-0.235052921	1.29236E-07
CD101	Natural killer T cell	-0.154557602	0.000431349	-0.153312412	0.000636275
CD109	Natural killer T cell	0.276427391	1.7402E-10	0.30199651	7.43963E-12
CNPY3	Natural killer T cell	-0.024314762	0.581955659	-0.042366227	0.347876303
CNPY4	Natural killer T cell	0.070649717	0.109287709	0.067004805	0.137373976
CREB1	Natural killer T cell	0.301205055	2.91827E-12	0.318084215	4.71061E-13
CRTC2	Natural killer T cell	0.047308467	0.283903642	0.032117112	0.476781287
CRTC3	Natural killer T cell	0.102491935	0.019997772	0.110946978	0.013709626
CSF2	Natural killer T cell	-0.17971755	4.09639E-05	-0.167497405	0.000186947
KLRC1	Natural killer T cell	0.087978847	0.045979954	0.093999474	0.036937827
FUT4	Natural killer T cell	0.21106591	1.34655E-06	0.22387276	5.11242E-07
ICAM2	Natural killer T cell	-0.208925222	1.7302E-06	-0.211150217	2.24713E-06
IL32	Natural killer T cell	0.045405592	0.303741241	0.066051658	0.143067884
LAMP2	Natural killer T cell	0.104659077	0.01750862	0.11763824	0.008937232
LILRB5	Natural killer T cell	-0.05768933	0.191183229	-0.054092088	0.230579144
KLRG1	Natural killer T cell	-0.158877555	0.000294875	-0.156580402	0.00048416

HSPA4	Natural killer T cell	0.37862194	5.3339E-19	0.390909927	1.89918E-19
HSPB6	Natural killer T cell	-0.24175588	2.76659E-08	-0.240447926	6.48987E-08
ISM2	Natural killer T cell	0.178439327	4.6547E-05	0.183002929	4.35897E-05
ITIH2	Natural killer T cell	-0.080487954	0.067988061	-0.089958581	0.045892191
KDM4C	Natural killer T cell	-0.038981932	0.37732985	-0.033273247	0.461052591
KIR2DS4	Natural killer T cell	0.029627934	0.502298036	0.045317699	0.315292857
KIRREL3	Natural killer T cell	-0.049147605	0.265579875	-0.040217199	0.372896706
SDCBP	Natural killer T cell	0.160017021	0.000266291	0.178685189	6.62027E-05
NFATC2IP	Natural killer T cell	-0.06164829	0.162428055	-0.064836517	0.150586554
MICB	Natural killer T cell	0.131802423	0.0027275	0.145087022	0.001236233
KIR2DL1	Natural killer T cell	-0.006249376	0.887492582	-0.010350957	0.818673629
KIR2DL3	Natural killer T cell	0.073300603	0.096583122	0.081901626	0.069225006
KIR3DL1	Natural killer T cell	0.057520882	0.192484687	0.066124983	0.14262354
KIR3DL2	Natural killer T cell	0.033200522	0.452161576	0.049856632	0.269214179
NCR1	Natural killer T cell	0.118123245	0.007284941	0.138526549	0.002050239
FOSL1	Natural killer T cell	0.277072766	1.57231E-10	0.290637208	4.72468E-11
TSLP	Natural killer T cell	-0.323068458	5.6365E-14	-0.320021207	3.34056E-13
SLC7A7	Natural killer T cell	0.01877992	0.670700998	0.034793299	0.440820179
SPP1	Natural killer T cell	0.202169254	3.75248E-06	0.208117786	3.15657E-06
TREM2	Natural killer T cell	-0.26249692	1.4582E-09	-0.268964974	1.28691E-09
UBASH3A	Natural killer T cell	-0.121251166	0.005867198	-0.114982066	0.010618336
YBX2	Natural killer T cell	0.257617299	2.98387E-09	0.254925049	9.41152E-09
CCDC88A	Natural killer T cell	0.13325992	0.002442897	0.138987632	0.001979983
CLEC1A	Natural killer T cell	-0.238918857	4.05503E-08	-0.254950954	9.37803E-09
THBD	Natural killer T cell	-0.164611961	0.000175297	-0.163966832	0.000255995
PDPN	Natural killer T cell	0.030938421	0.483573789	0.04044379	0.370207366
VCAM1	Natural killer T cell	0.109343312	0.013034933	0.144076424	0.001338261
EMR1	Natural killer T cell	-0.059522317	0.177436531	-0.053866006	0.232536549
ABCD1	Activated dendritic cell	0.09067378	0.039690622	0.08268309	0.066602917
C1QC	Activated dendritic cell	-0.013055007	0.767567694	-0.003394793	0.940067115
CAPG	Activated dendritic cell	-0.117600401	0.007549759	-0.10740045	0.01705466
CCL3L3	Activated dendritic cell	-0.060873088	0.167785982	-0.064388395	0.153433846
CD207	Activated dendritic cell	-0.378712017	5.22484E-19	-0.373530046	9.05439E-18

CD302	Activated dendritic cell	-0.383483821	1.7329E-19	-0.388408295	3.36014E-19
ATP5B	Activated dendritic cell	0.494471022	4.13873E-33	0.493598781	1.22463E-31
ATP5L	Activated dendritic cell	0.105327687	0.016797434	0.094789564	0.035372621
ATP6V1A	Activated dendritic cell	0.192387865	1.10009E-05	0.195882709	1.1826E-05
BCL2L1	Activated dendritic cell	0.033460971	0.448620223	0.036100211	0.423837834
C1QB	Activated dendritic cell	-0.052614534	0.233285491	-0.045399409	0.314420198
SNURF	Activated dendritic cell	0.023303328	0.597760816	0.034571588	0.443739235
SPCS3	Activated dendritic cell	0.230430718	1.23712E-07	0.252175201	1.37088E-08
CCNA1	Activated dendritic cell	-0.085455262	0.052608255	-0.081525188	0.070517927
CEACAM8	Activated dendritic cell	-0.278987042	1.16191E-10	-0.272304369	7.87965E-10
NOS2	Activated dendritic cell	0.052822176	0.231443516	0.04191297	0.353062854
SRA1	Activated dendritic cell	-0.038551976	0.382618224	-0.03149772	0.485326848
TNFRSF6B	Activated dendritic cell	0.021193795	0.631334803	0.036963872	0.41282769
TREM1	Activated dendritic cell	0.050777026	0.250038363	0.047050556	0.297125496
TREML1	Activated dendritic cell	-0.380833398	3.20602E-19	-0.400662096	1.95959E-20
RHOA	Activated dendritic cell	0.1025562	0.019919744	0.116474412	0.009642237
SLC25A37	Activated dendritic cell	0.061099293	0.16620904	0.061302326	0.174160628
TNFSF14	Activated dendritic cell	-0.111580258	0.011279345	-0.114641396	0.010853077
TREML4	Activated dendritic cell	0.004398468	0.920681694	0.003135631	0.944634816
VNN2	Activated dendritic cell	-0.107700883	0.014473195	-0.100391079	0.025812631
XPO6	Activated dendritic cell	0.229041537	1.4789E-07	0.236495897	1.07665E-07
CLEC4C	Activated dendritic cell	0.012752935	0.772796679	0.030470545	0.499678791
TNFAIP2	Activated dendritic cell	0.012487075	0.777407378	0.030815894	0.494828576
UBD	Activated dendritic cell	0.080410244	0.068255222	0.099394807	0.027328888
ACTR3	Activated dendritic cell	0.445476455	1.78981E-26	0.46724462	4.19267E-28
RAB1A	Activated dendritic cell	0.348198087	3.99408E-16	0.359303346	1.80848E-16
SLA	Activated dendritic cell	-0.12032725	0.006257681	-0.121519146	0.006906251
HLA-DQA2	Activated dendritic cell	-0.166544176	0.000146544	-0.168321656	0.000173561
SIGLEC5	Activated dendritic cell	-0.078249742	0.076034135	-0.070921853	0.115787327
SLAMF9	Activated dendritic cell	0.224373828	2.672E-07	0.218027163	1.02062E-06
CBX6	Plasmacytoid dendritic cell	-0.031001808	0.482677796	-0.018341187	0.684565646
DAB2	Plasmacytoid dendritic cell	-0.098062911	0.026057081	-0.086156686	0.055916129
DDX17	Plasmacytoid dendritic cell	-0.148866471	0.000701452	-0.147346621	0.001033488

HIGD1A	Plasmacytoid dendritic cell	0.189509155	1.49455E-05	0.192073779	1.75523E-05
IDH3A	Plasmacytoid dendritic cell	0.167658248	0.000132043	0.173418275	0.000108792
IL3RA	Plasmacytoid dendritic cell	-0.219670368	4.78786E-07	-0.211519986	2.15518E-06
MAGED1	Plasmacytoid dendritic cell	0.169075113	0.000115544	0.169471943	0.000156377
NUCB2	Plasmacytoid dendritic cell	0.033549923	0.447414339	0.03898862	0.387687433
OFD1	Plasmacytoid dendritic cell	-0.15653291	0.000362929	-0.158610327	0.000407491
OGT	Plasmacytoid dendritic cell	-0.159043559	0.00029054	-0.149062662	0.000900521
PDIA4	Plasmacytoid dendritic cell	0.414632345	8.1392E-23	0.418924484	2.26516E-22
SERTAD2	Plasmacytoid dendritic cell	0.101332834	0.021451447	0.10795163	0.016491994
SIRPA	Plasmacytoid dendritic cell	-0.026919499	0.542175386	-0.013106726	0.771594176
TMED2	Plasmacytoid dendritic cell	0.513454841	5.53479E-36	0.525422125	2.44981E-36
ENG	Plasmacytoid dendritic cell	-0.241943844	2.69693E-08	-0.23409283	1.45837E-07
FCAR	Plasmacytoid dendritic cell	0.108530588	0.013730067	0.117968767	0.008745563
IGF1	Plasmacytoid dendritic cell	0.023244262	0.598689794	0.040385265	0.370900823
ITGA2B	Plasmacytoid dendritic cell	-0.20735165	2.07686E-06	-0.21974759	8.34356E-07
GABARAP	Plasmacytoid dendritic cell	-0.227584568	1.78122E-07	-0.237495364	9.48073E-08
GPX1	Plasmacytoid dendritic cell	-0.147208093	0.000805674	-0.154299339	0.000586212
KRT23	Plasmacytoid dendritic cell	-0.003963405	0.928505135	-0.010482725	0.816406131
PROK2	Plasmacytoid dendritic cell	0.044191516	0.316866812	0.056087773	0.213805875
RALB	Plasmacytoid dendritic cell	0.265747012	8.97728E-10	0.294294857	2.62862E-11
RETNLB	Plasmacytoid dendritic cell	0.245539014	1.64917E-08	0.241110731	5.95646E-08
RNF141	Plasmacytoid dendritic cell	0.029008973	0.511272893	0.041112594	0.362339801
SEC14L1	Plasmacytoid dendritic cell	0.027731665	0.530054253	0.038477576	0.393943377
SEPX1	Plasmacytoid dendritic cell	0.170288419	0.000102977	0.17114548	0.000134206
EMP3	Plasmacytoid dendritic cell	-0.095099115	0.030944109	-0.086347038	0.055374235
CD300LF	Plasmacytoid dendritic cell	-0.25575356	3.90723E-09	-0.261785215	3.61222E-09
ABTB1	Plasmacytoid dendritic cell	-0.461113049	1.77969E-28	-0.459902102	3.57899E-27
KLHL21	Plasmacytoid dendritic cell	-0.059541992	0.177293064	-0.058793299	0.19249391
PHRF1	Plasmacytoid dendritic cell	0.040131282	0.363415096	0.033807575	0.45388216
ACADM	Immature dendritic cell	0.1427457	0.001161406	0.135982364	0.002480584
AHCYL1	Immature dendritic cell	0.068584666	0.120069342	0.064987134	0.149638632
ALDH1A2	Immature dendritic cell	-0.079472275	0.071548306	-0.074949091	0.096461831
ALDH3A2	Immature dendritic cell	-0.091693898	0.037507804	-0.116233329	0.009794311

ALDH9A1	Immature dendritic cell	-0.002978732	0.946236146	-0.00159863	0.971756585
ALOX15	Immature dendritic cell	-0.209378989	1.64101E-06	-0.193507931	1.51409E-05
AMT	Immature dendritic cell	-0.33081205	1.28679E-14	-0.330556841	4.93114E-14
ARL1	Immature dendritic cell	0.350197152	2.64169E-16	0.358015856	2.35414E-16
ATIC	Immature dendritic cell	0.375688725	1.04155E-18	0.37681573	4.43853E-18
ATP5A1	Immature dendritic cell	0.135822707	0.002007336	0.141058952	0.001690751
CAPZA1	Immature dendritic cell	0.353003734	1.47114E-16	0.365459772	5.04076E-17
LILRA5	Immature dendritic cell	0.076712121	0.081998106	0.100325408	0.025910297
RDX	Immature dendritic cell	0.39458066	1.23967E-20	0.398068955	3.61123E-20
RRAGD	Immature dendritic cell	-0.074778017	0.090031068	-0.071606156	0.112304965
TACSTD2	Immature dendritic cell	-0.035289036	0.424210025	-0.039725206	0.378777439
INPP5F	Immature dendritic cell	0.121213655	0.005882615	0.135233319	0.002622145
RAB38	Immature dendritic cell	-0.045926874	0.298217701	-0.036884919	0.413827136
PLAU	Immature dendritic cell	0.149679296	0.000655067	0.168440373	0.000171709
CSF3R	Immature dendritic cell	-0.212954555	1.07702E-06	-0.215982636	1.29402E-06
SLC18A2	Immature dendritic cell	-0.267192874	7.21946E-10	-0.273210567	6.88956E-10
AMPD2	Immature dendritic cell	-0.000731823	0.986781764	-0.001412398	0.975045662
CLTB	Immature dendritic cell	-0.056787508	0.198226552	-0.056589571	0.209730031
C1orf162	Immature dendritic cell	-0.237265012	5.05629E-08	-0.253772671	1.10241E-08
AIF1	Macrophage	-0.164087821	0.000183964	-0.161466083	0.000318615
CCL1	Macrophage	0.00726001	0.869449575	0.026240141	0.561074131
CCL14	Macrophage	-0.350637739	2.41068E-16	-0.364383658	6.31456E-17
CCL23	Macrophage	-0.210853136	1.38068E-06	-0.216373758	1.2368E-06
CCL26	Macrophage	0.283871586	5.31326E-11	0.305921866	3.85338E-12
CD300LB	Macrophage	-0.200777213	4.38742E-06	-0.199020635	8.49258E-06
CNR1	Macrophage	-0.307848627	9.10315E-13	-0.30757201	2.91373E-12
CNR2	Macrophage	-0.203667942	3.16736E-06	-0.222884436	5.75393E-07
EIF1	Macrophage	0.101423814	0.021334117	0.089881433	0.046079487
EIF4A1	Macrophage	0.431235728	9.73622E-25	0.433958588	4.66643E-24
FPR1	Macrophage	0.023200642	0.599376248	0.046803648	0.29967058
FPR2	Macrophage	0.092949906	0.034960931	0.11483197	0.0107212
FRAT2	Macrophage	0.127721432	0.003692331	0.122535377	0.006447838
GPR27	Macrophage	-0.088156615	0.045540744	-0.090762031	0.043979355

GPR77	Macrophage	-0.016865847	0.702577011	-0.028659966	0.525513478
RNASE2	Macrophage	0.046065379	0.296761416	0.071735773	0.111654725
MS4A2	Macrophage	-0.300673414	3.19932E-12	-0.292059043	3.76545E-11
BASP1	Macrophage	0.255860082	3.84771E-09	0.2511112	1.58375E-08
IGSF6	Macrophage	-0.15150903	0.000560861	-0.138241923	0.002094738
HK3	Macrophage	-0.105390236	0.016732213	-0.102041017	0.023460928
VNN1	Macrophage	0.012789863	0.772156882	0.021995429	0.62611873
FES	Macrophage	-0.160163781	0.000262804	-0.158466914	0.000412513
NPL	Macrophage	-0.070482137	0.110133157	-0.071592539	0.11237345
FZD2	Macrophage	-0.09907288	0.024551189	-0.100598969	0.025505551
FAM198B	Macrophage	0.162178535	0.000219049	0.179580968	6.0753E-05
HNMT	Macrophage	-0.259190318	2.37265E-09	-0.248321293	2.30517E-08
SLC15A3	Macrophage	-0.195394657	7.94875E-06	-0.204537293	4.68484E-06
CD4	Macrophage	-0.172260393	8.52572E-05	-0.173715551	0.000105824
TXNDC3	Macrophage	-0.227427164	1.81724E-07	-0.244920022	3.62074E-08
FRMD4A	Macrophage	-0.121295666	0.005848955	-0.122852639	0.006310421
CRYBB1	Macrophage	-0.118479772	0.007109159	-0.107739399	0.016706693
HRH1	Macrophage	0.139166383	0.001545866	0.160483698	0.000346916
WNT5B	Macrophage	-0.00330937	0.940278946	0.002921574	0.948408938
GIPR	Eosinophil	-0.316430895	1.9338E-13	-0.312993344	1.14879E-12
LRMP	Eosinophil	-0.213444951	1.016E-06	-0.22788186	3.14743E-07
FOSB	Eosinophil	-0.209305844	1.65508E-06	-0.213616115	1.69829E-06
RRP12	Eosinophil	0.280515718	9.11046E-11	0.268067704	1.46654E-09
GPR183	Eosinophil	-0.111455057	0.011371772	-0.109299303	0.01518419
NR4A3	Eosinophil	-0.065643757	0.136835561	-0.073017714	0.105382093
ST3GAL6	Eosinophil	-0.355742771	8.26196E-17	-0.3515185	8.7468E-16
DEPDC5	Eosinophil	-0.059548274	0.177247279	-0.06987678	0.121268226
PDE6C	Eosinophil	-0.003253986	0.941276592	0.006258897	0.889751494
PKD2L2	Eosinophil	-0.072159565	0.101899267	-0.07124145	0.114150534
GPR65	Eosinophil	-0.122297289	0.005451611	-0.113267759	0.011846905
IL5RA	Eosinophil	-0.154380052	0.000438054	-0.150701209	0.000788484
P2RY14	Eosinophil	-0.187892986	1.77156E-05	-0.189416548	2.30146E-05
DACH1	Eosinophil	-0.228716253	1.54177E-07	-0.228491894	2.92118E-07

DAPK2	Eosinophil	-0.455230438	1.0372E-27	-0.467688116	3.67718E-28
EMR3	Eosinophil	-0.144449134	0.001011302	-0.149151717	0.000894073
ADAMTS3	Mast cell	0.032389242	0.463292996	0.046244426	0.305488383
CPA3	Mast cell	-0.24865852	1.06951E-08	-0.236571259	1.0664E-07
CMA1	Mast cell	-0.244959279	1.78623E-08	-0.231468373	2.02388E-07
CTSG	Mast cell	-0.304242469	1.71954E-12	-0.291562556	4.07656E-11
ARHGAP15	Mast cell	-0.256721271	3.39773E-09	-0.278932313	2.91737E-10
CPM	Mast cell	-0.100989809	0.021898875	-0.112698767	0.012281596
FCN1	Mast cell	-0.232780371	9.12362E-08	-0.213641091	1.69346E-06
FTL	Mast cell	-0.10174351	0.020926236	-0.120069637	0.007610631
HSPA6	Mast cell	-0.053059999	0.229346491	-0.050240043	0.26554461
ITGA9	Mast cell	-0.198697355	5.53056E-06	-0.183953116	3.97081E-05
RNASE3	Mast cell	0.027421307	0.534669928	0.043596318	0.334045443
S100A4	Mast cell	-0.131397021	0.002811863	-0.12083318	0.00723197
SIGLEC8	Mast cell	-0.217657195	6.12159E-07	-0.225138101	4.39091E-07
SLC6A4	Mast cell	-0.230377702	1.2456E-07	-0.234842785	1.32706E-07
PTGS2	Mast cell	0.112653604	0.010513878	0.103789572	0.021173268
EGR3	Mast cell	-0.160416888	0.00025689	-0.160771964	0.000338378
PILRA	Mast cell	-0.083488339	0.058310717	-0.076990839	0.087697585
ASGR2	Monocyte	-0.009754818	0.825217488	0.001889262	0.96662468
CFP	Monocyte	-0.288581863	2.46231E-11	-0.290391793	4.91279E-11
ASGR1	Monocyte	-0.122652172	0.005316768	-0.120472142	0.007408865
CD1D	Monocyte	-0.223615493	2.93801E-07	-0.22035824	7.7646E-07
UPK3A	Monocyte	-0.159879862	0.000269589	-0.159380468	0.000381485
ACTG1	Monocyte	0.292063464	1.38174E-11	0.30123239	8.44637E-12
ANXA5	Monocyte	0.066548739	0.13149469	0.08664222	0.054542544
ATP6V1B2	Monocyte	-0.041821327	0.343544402	-0.028436802	0.528744255
CFL1	Monocyte	0.359021508	4.11079E-17	0.357645445	2.53911E-16
DAZAP2	Monocyte	0.163073811	0.000201883	0.162208251	0.000298679
CTBS	Monocyte	0.202909863	3.45148E-06	0.211498233	2.16049E-06
EMR4P	Monocyte	-0.282419368	6.71562E-11	-0.302429545	6.92218E-12
HIVEP2	Monocyte	0.159801208	0.000271498	0.17828119	6.88086E-05
MARCKSL1	Monocyte	0.273078179	2.93363E-10	0.270489137	1.02961E-09

MBP	Monocyte	-0.011622812	0.792449193	0.009504944	0.833267279
MMP15	Monocyte	-0.096826586	0.028008277	-0.102948664	0.02224806
PNPLA6	Monocyte	-0.150132136	0.000630474	-0.152954584	0.000655385
TMBIM6	Monocyte	0.200696423	4.42726E-06	0.2033064	5.35737E-06
PQBP1	Monocyte	0.01737092	0.694112175	0.003538296	0.937538716
TEX264	Monocyte	-0.229408059	1.41101E-07	-0.226800419	3.5906E-07
IKZF1	Monocyte	-0.15643381	0.000366105	-0.157127573	0.00046227
CREB5	Neutrophil	-0.001715612	0.969018873	0.022628259	0.616217946
CDA	Neutrophil	0.192793215	1.05324E-05	0.210914069	2.30781E-06
CHST15	Neutrophil	0.152732571	0.000505061	0.169445252	0.000156757
S100A12	Neutrophil	0.122565766	0.00534932	0.138261104	0.002091712
APOBEC3A	Neutrophil	0.048520587	0.271733525	0.052299194	0.246425143
CASP5	Neutrophil	0.143738898	0.001071566	0.173334311	0.000109644
MMP25	Neutrophil	-0.02799615	0.526136813	-0.021333195	0.636551983
HAL	Neutrophil	0.137373288	0.001779579	0.129653659	0.003930937
C1orf183	Neutrophil	-0.225480373	2.32506E-07	-0.222891226	5.74927E-07
FFAR2	Neutrophil	0.089593884	0.042117527	0.101219434	0.024607704
MAK	Neutrophil	-0.093932418	0.033072665	-0.096176779	0.032760875
CXCR1	Neutrophil	0.005255914	0.905285921	0.021328476	0.636626583
STEAP4	Neutrophil	-0.17379669	7.34875E-05	-0.177011199	7.76447E-05
MGAM	Neutrophil	-0.11652866	0.008119645	-0.125898528	0.005118947
BTNL8	Neutrophil	-0.210231523	1.48523E-06	-0.219812283	8.28032E-07
CXCR2	Neutrophil	-0.123412987	0.005037755	-0.108151298	0.016292214
TNFRSF10C	Neutrophil	-0.179865073	4.03619E-05	-0.175817815	8.69133E-05
VNN3	Neutrophil	-0.053907954	0.221979382	-0.051658519	0.252267919

TCGA: The Cancer Genome Atlas; LUAD: lung adenocarcinoma.
